# Contraceptive discontinuation, switching, abandonment and their reproductive consequences: An analysis of 1,539,071 episodes of reversible method use contributed from 61 countries that participated in DHS: Population base-analysis

**DOI:** 10.1371/journal.pgph.0005174

**Published:** 2025-10-31

**Authors:** Mohamed M. Ali, John Cleland

**Affiliations:** 1 Department of Sexual, Reproductive, Maternal, Child, Adolescent Health and Ageing: Advancing Life Course Health and Reproduction (LHR), World Health Organization, Geneva, Switzerland; 2 London School of Hygiene and Tropical Medicine, London, United Kingdom; Jhpiego, UNITED STATES OF AMERICA

## Abstract

As contraceptive uptake rises in a country, discontinuation of use of a contraceptive method because of dissatisfaction and the ability and willingness to switch promptly to an alternative following such discontinuation become increasingly important determinants of the prevention of unintended pregnancy. About one third of unintended births in low- and middle-income countries are the result of discontinuation or accidental pregnancy while using a method. Examination of the dynamics of contraceptive use continuation and switching can reveal how well contraceptive services are meeting the family planning needs of women and couples. We performed a population-based analysis of 1,539,071 episodes of use of reversible contraceptive methods from calendar data collected in 149 demographic and health surveys in 61 countries. Cause-specific discontinuation rates in the presence of competing events, switching to an alternative method within three months of discontinuation, and conception rates after cause-specific discontinuation, were computed using the cumulative incidence method. Competing risks marginal models were used to produce sub-hazard ratios of discontinuation, switching and abandonment. Meta-analysis was used to estimate the method-specific pooled cumulative incidence rates for discontinuation, switching and abandonment. We found that discontinuation because of dissatisfaction was highest in socio-economically developed countries with low unmet need for contraception. Conversely, switching within three months of discontinuation was much higher in these countries, with the net result that contraceptive abandonment, defined as discontinuation but no switching, was 31% lower in most developed societies than in the least developed. Women who switched were five times less likely to experience pregnancy within 12 months than abandoners. In conclusion, high discontinuation rates should be regarded as an unfortunate inevitability, which may be resistant to improvements in the quality of services. Increases in the ability and willingness to switch is a more plausible pathway to the reduction of unintended pregnancy.

## Introduction

Abandonment of contraceptive use can be defined as ceasing use of a method without switching promptly to an alternative one while still in need of protection against an unintended pregnancy. This definition excludes stopping use because of the desire for a child or because of no further need for protection (e.g. absence of a partner or menopause). Nearly all contraceptive abandonment stems from dissatisfaction with the method and is intentional; inability to obtain a supply, or cost, are rarely cited as reasons, as shown in the publicly accessible data base, StatCompiler. Abandonment has serious health implications. In low- and middle-income countries (LMICs) past contraceptive users, in distinction to never users, comprise 38% of unmet need for contraception, broadly defined as the proportion of married women who are not using a method despite a self-reported desire to delay pregnancy for two or more years [1]. Modelling of data from 2015 indicated that reduction of unmet need by 90% would prevent 67,000 maternal deaths globally, with large downstream effects on stillbirths, neonatal and child deaths [2]. Moreover, increased contraceptive use was estimated to be the most cost-effective way of reducing maternal deaths in 74 LMICs [3]. Reduction of unmet need for birth spacing would also have substantial direct benefits for perinatal, infant and child survival because of the risks posed by short inter-birth intervals, particularly in less developed countries where unmet need remains high [4,5].

Owing in large measure to collection of relevant data by the Demographic and Health Surveys (DHSs), much evidence has accumulated on contraceptive discontinuation and switching in LMICs. Most publications are single site studies but, since 2010, several multi-country analyses have been published [6–14]. The following robust generalisations may be drawn. Persistence of use is much longer for users of long-acting reversible methods (LARCs), IUDs and implants, than short-acting modern ones (SARCs). Both discontinuation and switching vary markedly between countries for reasons that are poorly understood. Cessation of use because of dissatisfaction with the method is dominated by side effects (e.g. disruption of menstrual bleeding) and health concerns (e.g. fears about long-term fertility impairment). Most studies have found positive associations between women’s education or economic status and switching. Associations with age, parity, urban-rural residence and motivation (i.e. spacing versus limitation) have been more mixed. Persistency of use may also be influenced by the quality of services, particularly counselling, but evidence is weak and results are mixed [15–17].

Reproductive consequences of contraceptive discontinuation have attracted little attention with only three multi-country studies [9,14,18]. Typically, between 10–15% of women experience pregnancy within 12 months of stopping a method because of dissatisfaction, with wide variation between countries of 5–27%. The majority of pregnancies are classified by mothers as unintended (i.e. mistimed or unwanted). About one-third of all unintended births in LMICs are the result of method-related discontinuation or accidental pregnancy while using a method [18].

We add to this evidence-base in the following main ways. First, in contrast to most studies that examine discontinuation and switching as separate outcomes, we combine them to derive an additional composite measure of abandonment, defined as failure to switch within three months following method-related discontinuation. Second, we examine trends in discontinuation, switching and abandonment for countries with three or more surveys. Third, in analyses of predictors, in addition to the conventional individual and household factors, we go beyond all previous multi-country analyses by including three country-level factors: the Human Development Index (HDI); unmet need for family planning as an indicator of societal access to, and acceptability of, contraception; and method-skewness as a proxy for access to a range of methods, defined as the percentage of current contraceptive users who rely on the most commonly used method. HDI and unmet need for family planning were negatively correlated (*ρ*(56)= -0.4833, *P-value = 0.0002*), but neither was correlated with skewness. We hypothesise that low HDI, high unmet need and high method-skewness will be positively related to the level of contraceptive abandonment. Fourth, we document the conception rates of switchers and abandoners over a longer time period than used in previous studies in order to assess the extent to which the effect of switching persists beyond the conventional 12-month span of time. Disaggregation of estimates between these two categories will reveal the degree of advantage in avoiding pregnancy that switchers enjoy over abandoners. To our knowledge, no previous analysis has compared switchers and abandoners in terms of conception rates.

## Methods

### Data source

The Demographic and Health Survey (DHS) is the major data source for contraceptive use dynamic. From 1984 to date, the DHS programme had eight phases (DHS-I to DHS-VIII) collecting data from more than 400 surveys in more than 90 countries [19]. The contraceptive calendar takes the form of a grid in which contraceptive status is recorded for each calendar month The exact length of the period covered by the calendar varies depending on the defined recall period and duration of the survey data collection. In most surveys, the period covered by the calendar includes the months up to the month of interview in the year of interview, plus the five calendar years preceding the year of interview. Specifically, interviewers are trained to enter on the monthly grid all live births (and current pregnancy if any), ascertained earlier in the interview. These reproductive events are used as anchor-points in the determination of dates of starting and stopping the use of specific contraceptive methods over the calendar period. Any pregnancy loss detected at this stage of detailed questioning is entered into the calendar, but no attempt is made to distinguish induced from spontaneous abortions. Reasons for stopping method-use are entered into the calendar. Monthly calendars of contraceptive use have been found to yield more complete and accurate data than other retrospective formats [20–22]. Calendar data for the three months prior to the interview date were omitted to avoid the problem of under-reporting of first trimester pregnancies [23].

The data are third-party and are not owned or collected by the authors, authors have access to anonymised individual records and do not have any special access privileges, the last time a data file was downloaded was on 23 September 2024.

### Ethical review

Procedures and questionnaires for standard DHS surveys have been reviewed and approved by ICF Institutional Review Board (IRB). Additionally, country-specific DHS survey protocols are reviewed by the ICF IRB and typically by an IRB in the host country. ICF IRB ensures that the survey complies with the U.S. Department of Health and Human Services regulations for the protection of human subjects (45 CFR 46), while the host country IRB ensures that the survey complies with laws and norms of the nation.

### Statistical analysis

We performed a population-based analysis of DHS. The unit of analysis for discontinuation of specific methods is an episode of contraceptive use, defined as a period of uninterrupted use (in months) that may or may not have ended. We have retained contraceptive episodes that started between month 63 and 4 prior to interview date. The episodes used in this paper were contributed by all women regardless of their marital status at the time of use (S1 Table).

The full sample comprised 149 surveys with 100 or more method-specific episodes of reversible methods from 61 countries. The total number of episodes was 1,539,071, distributed as follows: oral contraceptives 375,253, injectables 299,471, condoms 266,454, withdrawal 177,008, periodic abstinence 167,022, IUDs 127,999, implants 54,100, lactational amenorrhea method (LAM) 33,762 with the remaining 37,992 episodes comprised of other modern methods (13,707), and other traditional methods (24,285). The episodes of LAM, other modern methods and other traditional methods were excluded from all analyses.

We analyse four main outcomes: 1) cause-specific discontinuation within 12 months of starting a method; 2) switching to an alternative method within three months of discontinuation; 3) abandonment, defined as discontinuation within 12 months for method-related reasons without switching within three months; and 4) rate of conception within 12 and 24 months of discontinuation.

The main analysis was performed on most recent surveys with 100 or more method-specific episodes from 56 countries, excluding a few countries whose most recent survey was conducted before the year 2000. The total number of episodes for the reduced sample was 610,191. The number of surveys represented in the full and reduced sample varied by method as follows; oral contraceptives 144 surveys (most recent survey 54 countries), condom 136 (50), injectable 129 (49), withdrawal 113 (40), IUDs 111 (38), periodic abstinence 109 (35), and implants 64 (33).

The analysis of switching was restricted to surveys with 100 or more method-specific episodes discontinued for method-related reasons, comprising 141 surveys from 57 countries in the full sample, and 52 countries for the reduced sample. The number of surveys represented in the full and reduced sample is as follows: oral contraceptives 125 surveys (most recent survey 43 countries), condom 63 (22), injectable 115 (43), withdrawal 49 (16), IUDs 54 (14), periodic abstinence 29 (7), and implants 29 (21). The total number of episodes in the reduced sample is 127,493.

The analysis of reproductive consequences was based on surveys with 100 or more episodes discontinued for method-related reasons, regardless of the method discontinued. The number of surveys included was 147 from 60 countries in the full sample and from 55 countries in the reduced sample, after excluding two surveys *Moldova* (2005) and *Türkiye* (2003/4). The comparison of conception rates among switchers and abandoners was further restricted to episodes that started 39 months before interview date in order to compute the cumulative incidence of conceptions at 24 months in addition to 12 months and was based on 140 surveys from 57 countries in the full sample and from 51 countries for the reduced sample of most recent surveys.

The list of methods extracted, and grouping, is tabulated in S2 Table. Women were asked to give the primary single reason for each discontinued episode. Reasons were grouped into broad categories: became pregnant while using; method-related reasons (including side effects and health concerns), wanted to become pregnant/no need, and other/not stated (S3 Table). Method-related reasons are our main focus of interest. They imply some degree of dissatisfaction with the method. Moreover, it can be assumed that most women who stop use for this type of reason remain at risk of an unwelcome pregnancy. Because most women who stop use for this reason do so within the first 12 months of use, we use a 12-month measure of discontinuation [9]. Following method-related discontinuation, the focus of attention shifts to switching. We report switching to any method within three months. The three-month criterion was chosen because most women who switch do so within this span [9,14].

We then estimate the cumulative incidence of conception at 12- and 24-months following cause-specific discontinuation, comparing switchers and abandoners. Conceptions were classified as wanted or unwanted indirectly, based on a comparison of the actual number of living children at time of conception and the total desired number. If the actual number at time of conception plus one was greater than the desired number, the conception was defined as unwanted. All other conceptions, including non-numeric answers to the question of desired number, were defined as wanted. This approach is a departure from all previous analyses that have used a mother’s retrospective classification of current pregnancies and live births as wanted at that time, mistimed, or unwanted. Both approaches are vulnerable to rationalisation and provide lower-bound estimates of undesired conceptions. Our approach has the advantage of permitting the classification of conceptions that did not end in live births but the disadvantage of an inability to identify mistimed births or current pregnancies.

### Covariates

In addition to the three country-specific factors described above, we extracted the following individual/household covariates on the basis that they have been shown to exert an influence on contraceptive behaviour: rural or urban residence; household wealth score re-coded into tertiles (poor, middle, and rich); women’s years of education; age at start of each episode in years (<25, 25–34, and ≥35 years); number of living children at start of each episode (0–1, 2–3, and 4+); and motivation for use (i.e. using the method to limit births or to space them) which was derived indirectly by comparing desired and actual number of living children at start of method use.

### Statistical methods

Cause-specific discontinuation rates at 12 months in the presence of competing events, the status of women at three months after having discontinued for method-related reasons, and conception rates at 12 and 24 months after cause-specific discontinuation, with their 95% confidence intervals, were computed using the cumulative incidence method [24–26]. Competing risks marginal models using Fine and Gray proportional sub-hazards models that take into account the hierarchical nature of the data (clustering of episodes withing sampled clusters and surveys) were used to produce sub-hazard ratios of method-related reasons at 12 months, switching at three months following discontinuation due to method-related reasons and conception rates at 12 months with their 95% confidence intervals [27].

Random-effects meta-regression was used to estimate the method-specific pooled cumulative incidence rates for method related discontinuation at 12 months, switching at three months, and rates of abandonment at 12 months, weighted by the inverse of the standard errors of the country-specific point estimates and summarized using forest plots. For countries with three or more surveys, method specific trends were modelled using linear regression with the mid-calendar period as a predictor. 12- month conception rates vs 12-month method-related discontinuation rates were plotted using a scatter plot to illustrate diversity between countries. All analyses were restricted to methods with at least 100 method-specific episodes per survey. Much of the analysis is restricted to the most recent survey for 56 countries, excluding surveys conducted prior to the year 2000.

### Data quality assessment

We assessed the possibility that data quality might deteriorate with time elapsed since start of episodes and date of interview, because of greater omission of short episodes at longer durations. Specifically, we estimated the overall discontinuation rates using two time periods: 4–39 months and 4–63 months before the survey date, by comparing the point estimates of the shorter period with the 95%CI of the longer period. The results, summarised in S4 Table, provided no evidence of an association between omission and time elapsed.

## Results

The full data set comprises 149 surveys conducted in 61 countries between 1990 and 2023. Sub-Saharan Africa is overrepresented with 29 countries, followed by 13 countries in Central, South and South-East Asia, 10 in North Africa, Western Asia and Europe and nine in Latin America. In sub-Saharan Africa and Latin America, survey respondents were all women aged 15–49 regardless of marital status whereas in other regions many surveys were restricted to ever-married women (S5 Table).

The 56 most recent surveys encompassed a wide range of national circumstances. The proportion of women with secondary or higher education varied from 12% to 100% and the HDI from 0.35 to 0.84. Contraceptive prevalence ranged from 7% to 79%, unmet need from 4%-29% and method skewness from 18%-91%. (S6 Table). Contraceptive prevalence was lower in the sub-Saharan African countries and unmet need higher than elsewhere, but method skewness varied little between regions. (S1 Fig).

### 1-Discontinuation

Probabilities of discontinuation for any reason by the end of the first year of use varied widely between methods. Oral contraceptive users were most prone to stopping with a median value of 48.0 per hundred episodes followed by injectables (45.0%), condoms (39.3%), withdrawal (37.1%) and periodic abstinence (29.8%). Probabilities for users of the two LARCs were much lower, at 15.5% for IUDs and 14.5% for implants (S7 Table and S2 Fig). For all modern methods, except condoms, dissatisfaction with the method, largely side effects or health concerns, accounted for about half or more of all discontinuations. Accidental pregnancy while using the methods was most common for the two traditional methods (periodic abstinence 7.9%, withdrawal 7.7%) followed by condoms (2.8%). The proportions of women who stopped in order to get pregnant or who had no further need for protection ranged from 11.4% to 15.8%, with the marked exceptions of LARCs (3.3-3.4%). Results for all surveys with sufficient method-specific episodes are shown in S8 Table.

Trends in method-related discontinuation by month 12 were examined for countries with three or more surveys. (S9 Table). Of 105 trends, statistically significant (*P ≤ 0.05*) declines were observed in 40 cases versus significant increases in 25 cases (S3 Fig). The most pronounced method-specific result was for condoms with significant declines in 12 countries and only two significant increases, followed IUDs with 7 declines and three increases. For other methods, declines and increases were more balanced.

Discontinuation because of dissatisfaction for the four most commonly used modern methods was generally higher for women in the Latin American countries than in other regions. Discontinuation of injectables in the sub-Saharan countries, where this is the most commonly used method, was lower than in other regions (Table 1). Country variations were pronounced within regions (S4 Fig).

**Table 1:** Pooled overall and region-specific 12-month culminative incidence of discontinuation for method-related reasons, switching within three months and abandonment: most recent survey from 56 countries (discontinuation and abandonment), and 52 (switching).

	Discontinuation				Switching				Abandonment			
	Rate	95%CI			Rate	95%CI			Rate	95%CI		
**Oral contraceptives**												
Sub-Saharan Africa	23.4	(19.9	–	26.8)	35.8	(30.1	–	41.5)	14.9	(12.7	–	17.0)
North Africa Western Asia and Europe	18.5	(14.9	–	22.0)	56.2	(44.7	–	67.8)	7.8	(4.8	–	10.8)
Central, South & Southeast Asia	18.5	(15.9	–	21.2)	52.7	(42.1	–	63.3)	8.7	(6.3	–	11.2)
Latin America & Caribbean	30.8	(22.2	–	39.5)	53.4	(38.8	–	68.0)	13.1	(10.3	–	15.9)
*Overall*	*22.3*	*(20.0*	–	*24.6)*	*44.7*	*(39.6*	–	*49.8)*	*12.3*	*(10.8*	–	*13.9)*
**IUDs**												
Sub-Saharan Africa	12.8	(4.5	–	21.1)					8.2	(2.5	–	13.9)
North Africa Western Asia and Europe	5.9	(3.0	–	8.8)	57.2	(42.2	–	72.1)	2.9	(2.1	–	3.8)
Central, South & Southeast Asia	11.3	(7.9	–	14.6)	44.3	(24.8	–	63.7)	6.7	(4.0	–	9.4)
Latin America & Caribbean	14.7	(10.2	–	19.1)					5.2	(2.0	–	8.4)
*Overall*	*11.0*	*(7.7*	–	*14.3)*	*51.5*	*(41.8*	*–*	*61.2)*	*6.3*	*(3.9*	–	*8.7)*
**Injectables**												
Sub-Saharan Africa	23.5	(20.7	–	26.3)	24.8	(20.6	–	29.0)	17.8	(15.3	–	20.3)
North Africa Western Asia and Europe	33.3	(25.3	–	41.3)					14.7	(11.5	–	17.9)
Central, South & Southeast Asia	26.9	(23.1	–	30.6)	50.9	(41.7	–	60.1)	14.2	(10.4	–	17.9)
Latin America & Caribbean	33.1	(24.4	–	41.8)	56.6	(45.7	–	67.6)	13.4	(8.5	–	18.4)
*Overall*	*26.1*	(23.8	–	28.5)	*35.2*	(29.9	–	40.5)	*16.4*	(14.6	–	18.2)
**Condom**												
Sub-Saharan Africa	11.1	(8.6	–	13.6)	49.7	(41.7	–	57.8)	5.5	(4.4	–	6.6)
North Africa Western Asia and Europe	12.3	(6.9	–	17.6)					2.5	(1.0	–	3.9)
Central, South & Southeast Asia	11.7	(9.0	–	14.4)	60.0	(43.2	–	76.8)	5.1	(3.3	–	7.0)
Latin America & Caribbean	20.2	(15.4	–	25.1)	70.1	(65.2	–	74.9)	6.0	(4.3	–	7.7)
*Overall*	*12.4*	*(10.5*	–	*14.2)*	*60.3*	*(53.1*	–	*67.6)*	*4.9*	*(4.1*	–	*5.7)*
**Implants**												
Sub-Saharan Africa	10.1	(7.1	–	13.0)	26.1	(21.2	–	31.0)	7.6	(5.5	–	9.7)
North Africa Western Asia and Europe												
Central, South & Southeast Asia	8.5	(6.5	–	10.6)					3.5	(2.5	–	4.5)
Latin America & Caribbean												
*Overall*	*10.5*	*(8.2*	–	*12.8)*	*32.0*	*(25.2*	*–*	*38.8)*	*6.6*	*(5.0*	–	*8.2)*
**Periodic abstinence/rhythm**												
Sub-Saharan Africa	4.9	(3.7	–	6.2)					2.2	(1.4	–	2.9)
North Africa Western Asia and Europe	8.0	(4.4	–	11.6)					0.5	(0.2	–	0.8)
Central, South & Southeast Asia	8.2	(2.7	–	13.7)					2.3	(0.5	–	4.1)
Latin America & Caribbean	13.1	(6.9	–	19.3)					3.2	(0.7	–	5.7)
*Overall*	*7.5*	*(5.6*	–	*9.5)*	*70.8*	*(62.4*	*–*	*79.2)*	*2.1*	*(1.4*	–	*2.7)*
**Withdrawal**												
Sub-Saharan Africa	11.4	(7.8	–	14.9)					4.3	(3.0	–	5.6)
North Africa Western Asia and Europe	7.3	(3.5	–	11.2)					1.1	(0.5	–	1.7)
Central, South & Southeast Asia	8.3	(6.2	–	10.3)	72.4	(60.8	–	83.9)	2.2	(1.0	–	3.3)
Latin America & Caribbean	18.6	(11.2	–	26.1)	80.5	(72.0	–	88.9)	3.3	(1.1	–	5.5)
*Overall*	*10.5*	*(8.4*	–	*12.7)*	*76.9*	*(70.3*	*–*	*83.4)*	*2.7*	*(2.0*	–	*3.4)*

Note: region-specific pooled estimated was suppressed for region with fewer than 5 surveys.

The method’s *overall* estimates are weighted average of the region’s estimates. The region-specific weights are the inverse of the the variance (i.e. the information).

### 2-Switching

Following method-related discontinuation, it is reasonable to assume that most, but not all, women will still be at risk of an unintended pregnancy with a need to switch to an alternative method. The status of women three months after discontinuation has three possibilities: already pregnant; switched to another method (or very rarely to the same type of method); and at risk of pregnancy. Typically, between 8–11% were pregnant but switching probabilities ranged from about 30% for those who stopped using injectables or implants to 74% or more for prior users of traditional methods who predominantly adopted SARCs (S10 Table). Results for all surveys that met the inclusion criteria are shown in (S11 Table).

Trends in such switching within three months of stopping specific methods were estimated for 57 countries (S12 Table). The overall impression was that there had been no improvement in switching probabilities with an equal balance between statistically significant increases and decreases. (S5 Fig).

Women in sub-Saharan countries had much lower probabilities of switching than elsewhere (Table 1). As with discontinuation, wide inter-country variability was observed (S6 Fig). For the two most commonly used methods we checked whether high discontinuation was offset by high switching at the country level, but the correlations were low (for oral contraceptives *ρ*(43)= 0.0586, *P-value = *0.7089, and for injectables *ρ*(43)= 0.2189, *P-value =* *0.1584*).

### 3-Abandonment

The cumulative incidence of abandonment was highest for injectables (16.4 per 100 episodes) followed by oral contraceptives (12.3) but much lower for other methods (Table 1 and S13 Table). For oral contraceptives, IUDs and injectables, rates were highest in the countries of sub-Saharan Africa. Results for all surveys with sufficient method-specific episodes are shown in S14 Table and for most recent surveys in S7 Fig.

Trends in abandonment were examined for countries with three or more surveys (S15 Table). Of 105 trends, statistically significant (*P ≤ 0.05*) declines were observed in 27 cases versus significant increases in 21 cases (S8 Fig). The most pronounced method-specific result was for oral contraceptives with significant declines in 8 countries and only one significant increase, followed with seven declines and three increases for condoms. Abandonment increased among users of traditional methods.

#### Predictors of method-related discontinuation, switching and abandonment.

Table 2 shows the results of the marginal competing risk models expressed as sub-hazard ratios (sHRs). Relative to oral contraceptives, the adjusted sHR of discontinuation for injectables was 22% higher but 57% lower for implants, 43% lower for IUDs, and 24% lower for condoms. Differences between socio-demographic groups and between limiters and spacers were small (<10%). Among the country-level factors, associations between discontinuation and method skewness and unmet need were unclear with lower sHRs in the medium group than in the high or low groups. Discontinuation was highest in high HDI, low unmet need and low skewness settings.

**Table 2:** Adjusted sub-hazard ratios (sHRs) of 12-month method-related discontinuation, switching within three months and abandonment: most recent survey for 56 countries (discontinuation and abandonment), and 52 (switching).

	Discontinuation				Switching				Abandonment			
	sHR	95% CIs			sHR	95% CIs			sHR	95% CIs		
**Method**												
Pill	1.00				1.00				1.00			
IUD	**0.57**	**(0.54**	–	**0.60)**	0.95	(0.90	–	1.00)	**0.65**	**(0.61**	–	**0.70)**
Injectables	**1.22**	**(1.18**	–	**1.25)**	**0.88**	**(0.86**	–	**0.90)**	**1.37**	**(1.32**	–	**1.42)**
Condom	**0.76**	**(0.72**	–	**0.80)**	1.00	(0.97	–	1.04)	**0.77**	**(0.73**	–	**0.80)**
Implants	**0.43**	**(0.41**	–	**0.46)**	**0.70**	**(0.66**	–	**0.75)**	**0.57**	**(0.53**	–	**0.60)**
Periodic abstinence	**0.45**	**(0.43**	–	**0.48)**	**1.14**	**(1.09**	–	**1.18)**	**0.40**	**(0.38**	–	**0.43)**
Withdrawal	**0.48**	**(0.46**	–	**0.50)**	**1.30**	**(1.26**	–	**1.35)**	**0.38**	**(0.36**	–	**0.40)**
**Place of residence**												
Rural	1.00				1.00				1.00			
Urban	0.97	(0.93	–	1.01)	0.98	(0.95	–	1.01)	0.99	(0.95	–	1.03)
**Household wealth**												
Poor	1.00				1.00				1.00			
Middle	**1.04**	**(1.00**	–	**1.07)**	**1.11**	**(1.08**	–	**1.15)**	**0.96**	**(0.93**	–	**0.99)**
Rich	**1.05**	**(1.01**	–	**1.10)**	**1.18**	**(1.14**	–	**1.22)**	**0.93**	**(0.89**	–	**0.97)**
**Women’s years of schooling**	**0.98**	**(0.97**	–	**1.00)**	**1.04**	**(1.02**	–	**1.05)**	**0.95**	**(0.94**	–	**0.97)**
**Age group**												
35+	1.00								1.00			
25-34	**0.92**	**(0.88**	–	**0.95)**	**1.08**	**(1.05**	–	**1.12)**	**0.87**	**(0.83**	–	**0.90)**
15-24	0.96	(0.92	–	1.00)	**1.07**	**(1.03**	–	**1.11)**	**0.89**	**(0.85**	–	**0.94)**
**No of living children**												
0-1	1.00								1.00			
2	0.98	(0.95	–	1.01)	**1.05**	**(1.02**	–	**1.09)**	**0.92**	**(0.89**	–	**0.95)**
3+	**0.91**	**(0.87**	–	**0.95)**	0.98	(0.94	–	1.03)	**0.89**	**(0.84**	–	**0.94)**
**Motivation**												
Spacer	1.00								1.00			
Limiter	**1.06**	**(1.03**	–	**1.09)**	**1.12**	**(1.09**	–	**1.15)**	**0.96**	**(0.93**	–	**0.99)**
**Survey specific**												
**HDI**												
Low (<0.55)	1.00								1.00			
Medium (0.55-0.69)	1.00	(0.96	–	1.04)	0.95	(0.91	–	1.00)	**1.07**	**(1.03**	–	**1.12)**
High (0.7+)	**1.06**	**(1.00**	–	**1.11)**	**1.42**	**(1.34**	–	**1.50)**	**0.69**	**(0.65**	–	**0.74)**
**Unmet need for contraception**												
High: (16.1-29.0)	1.00								1.00			
Medium: (11.1-16.0)	**0.80**	**(0.77**	–	**0.84)**	**1.44**	**(1.35**	–	**1.54)**	**0.69**	**(0.65**	–	**0.73)**
Low: (04.0-11.0)	**1.20**	**(1.15**	–	**1.25)**	**1.98**	**(1.86**	–	**2.11)**	**0.78**	**(0.74**	–	**0.82)**
**Method skewness**												
High: (47.1-99.0)	1.00								1.00			
Medium: (35.1-47.0)	**0.75**	**(0.72**	–	**0.78)**	**1.22**	**(1.18**	–	**1.27)**	**0.69**	**(0.66**	–	**0.71)**
Low: (17.0-35.0)	**1.14**	**(1.10**	–	**1.18)**	**1.28**	**(1.24**	–	**1.32)**	0.97	(0.93	–	1.01)

Country-level factors were the strongest predictors of switching. The sHRs were 1.42 (95%CI:1.34-1.49) for women living in high versus low HDI countries, 1.28 (95%CI:1.24-1.32) for low versus high method-skewness and 1.98 [1.86-2.11] for low versus high unmet need settings. Among the individual factors, the probability of switching increased by 4% for each additional year of schooling, was 18% higher for women living in rich relative to poor households and was 12% higher for limiters versus spacers. Relative to women who stopped use of oral contraceptives, those who discontinued implant use were much less likely to switch with an sHR of 0.70 (95%CI:0.66-0.77) (Table 2).

Injectable users were much more likely to abandon contraception than users of other methods with a sHR of 1.37 (95%CI:1.32-1.42) relative to users of oral contraceptives; this reflected high discontinuation combined with low switching. Abandonment for LARC users was 35–43% lower than for oral contraceptive users because of low discontinuation while the lowest rates were for traditional method users with low discontinuation and high switching. Abandonment increased with age but fell with number of living children. Education was strongly associated with a 6% decline for each year of schooling but the association with motivation was modest.

Relative to low HDI settings, abandonment was slightly higher for those living in medium HDI settings but much lower in high settings with an sHR ratio of 0.78 (94%CI: 0.65-0.74). Abandonment was lowest in settings with medium skewness. For reasons that are unclear, method-related discontinuation was lower in this group of countries than in countries with high or low skewness and switching was similar to that in the low skewness group. Finally, abandonment was about 30% lower in countries with medium or low unmet need than in high unmet countries.

### 4-Reproductive consequences and conception rates

**Fig 1:**
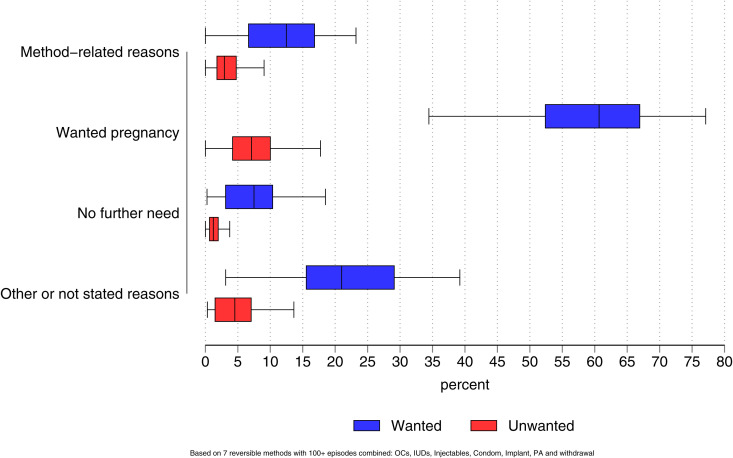
Medians and inter-quartile ranges of 12- month cumulative incidence of wanted and unwanted conceptions following specified reason for discontinuation: most recent survey for 55 countries.

The cumulative 12-month incidence of conception varied markedly by the four major reasons for discontinuation. Following method-related discontinuation, the median rate of a wanted conception was 12.5 per 100 discontinuations with an interquartile range of 11.9 (S16 Table and S9 Fig). The median rate of an unwanted conception (in excess of desired number of children) was 2.9, implying that about 19% of conceptions were unwanted. As expected, conception rates were highest when discontinuation was motivated by desire for a child with median rates of 60.7 and 7.1 for wanted and unwanted conceptions, respectively, implying that about 10% of conceptions were unwanted. Conception rates were lowest when the reason was “no further need” and intermediate for the small number of cases when reason was unstated or unclassified (Fig 1).

**Fig 2:**
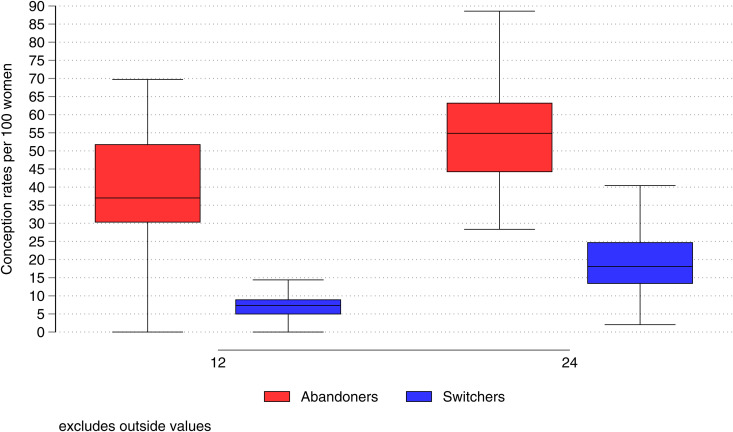
Medians and inter-quartile ranges of 12- and 24-month cumulative incidence of conceptions for switchers and abandoners, following method-related discontinuation: most recent survey for 51 countries.

To assess the efficacy of switching in avoidance of conception, we compared 12- and 24-month conception rates between switchers and abandoners following method-related discontinuation. At 12- and 24-months, the median conception rates for abandoners were 37.0 and 54.8, respectively. The corresponding rates for switchers were 7.3 and 18.1 (Fig 2 and S10 Fig). Country variations were pronounced (S17 Table)

#### Country variations.

**Fig 3:**
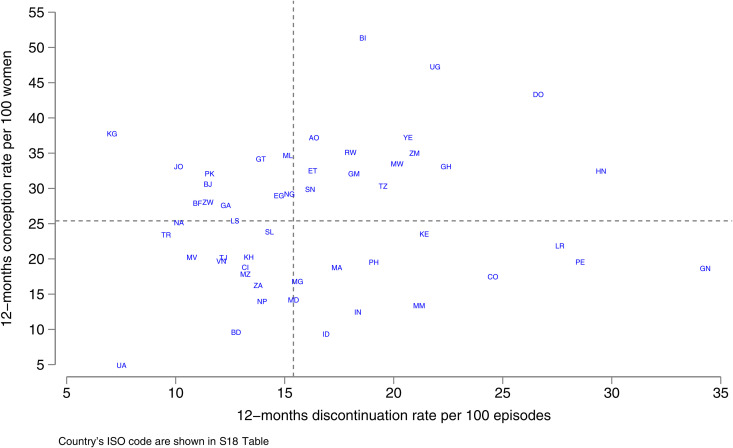
Conception rates following method-related discontinuation vs. method-related discontinuation: most recent survey for 51 countries.

We summarised country variations in a scatterplot of 12-month method-related discontinuation and 12-month conception rates following such discontinuations Wide variation was found on both axes (Fig 3). The ranking of countries are shown in (S18 Table). Of the 14 countries with higher than median discontinuation and conception, 11 were in sub-Saharan Africa, together with Dominican Republic, Yemen and Honduras. Most of the countries with lower than median rates were in Europe and Asia, with three in sub-Saharan Africa.

### Limitations

While clinical trials are the main source of information on the properties of contraceptive methods and their safety and efficacy, evidence from population-based surveys, such as the DHS, is invaluable in revealing the real-life contraceptive experiences of women, and couples, and how these vary by individual and national characteristics. These survey data also have limitations, not least in the severe recall demands imposed on women. Individual reliability or consistency of reporting has been assessed in several recent longitudinal studies. In an analysis of nine populations in seven countries, current reproductive status (pregnant, no use, current use) at baseline was compared with retrospective calendar data for the same month collected one to two years later. Kappa values ranged from 0.60 to 0.81 indicating moderate to excellent agreement. Kappas were similar when comparing method-specific use, though higher for LARCs than for other methods [28]. A comparison of overlapping calendar data, collected one year apart in Burkina Faso, Kenya and Uganda, showed that dates of discontinuation were reported with low precision. Only 25% to 36% of discontinuations reported at round 1 were reported at round 2 within two months either side of the round 1 date. The implications for length of use before discontinuation are unclear. When events matched, consistency of reporting of broad reason for stopping (contraceptive failure, method-related, other including desire for pregnancy) was moderate to high, ranging from 66% to 82% [29]. In urban Kenya, an overlap of 33 months between two rounds of calendar data collection revealed a modest aggregate difference in all cause 12-month discontinuation (34% versus 39%) and a larger difference in switching (12% versus 17%) [30]. Our conclusion from this evidence is that retrospective calendar data do not suffer from severe biases that would invalidate our results though imprecision of recall implies that interpretation should be cautious.

Other limitations should be noted. Reliance on a single main cause of discontinuation does insufficient justice to complexities of motivation. Induced abortions are undoubtedly prone to underreporting and thus our estimated conception rates are downwardly biased. The identification of unwanted conceptions is unsatisfactory because of post factum rationalisation whereby desired number of children is adjusted to correspond to a new conception. We also acknowledge that our 12-month measure of discontinuation and our three-month measure of switching do not capture all discontinuations or switchings. Finally, it should be noted that permanent methods, dominant in India and some Latin American countries, were omitted from analyses.

## Discussion

Our results suggest that high rates of discontinuation because of dissatisfaction with the method, particularly for the two most commonly used hormonal methods, oral contraceptives and injectables, should be regarded as an unfortunate inevitability and unlikely to decline in the future. The reason for this interpretation is that such cessation of use was found to be highest in the most advanced countries, with high HDI values, and with low levels of unmet need and method-skewness. Rather than protecting against dissatisfaction that prompts discontinuation, high standards of living, education and health, together with societal endorsement of modern contraception, appear to increase it. To the extent that anticipatory counselling about side-effects is better in more developed countries, improvements in such counselling may have little effect in reducing discontinuation.

The apparent inevitability of discontinuation while still at risk of an unintended pregnancy underscores the importance of switching to an alternative method. Indeed, discontinuation followed by prompt switching should be viewed positively as a reflection of women’s ability to stop a method that is found to be unsatisfactory and to choose an alternative. Typically, about half or more women who stop use of a method within 12 months of starting because of dissatisfaction switched within three months, though rates were lower for users of injectables and implants. In contrast to results for discontinuation, high HDI values, low unmet need and skewness were strongly related to switching as were individual factors of women’s length of schooling and household wealth. Thus, progress in contraceptive protection is driven by improvements in willingness and ability to try another method when the earlier method is rejected. The absence of any marked improvement in switching over time in countries with three or more surveys was surprising and acts as a caveat.

Rates of contraceptive abandonment, defined here as discontinuation without switching, was 31% lower in high than in low HDI countries and 22% lower in low than high unmet need settings. Rates also varied by method even after adjustment for other predictors. They were lowest for users of traditional methods though the high failure rates for these methods should be born in mind. They were also low for LARCs because of low discontinuation. They were highest for injectable users, a reflection of high discontinuation and low switching. This result is of concern because injectables are the most commonly used method in sub-Saharan Africa where unmet need for family planning is greatest.

Our results confirm the high efficacy of switching in avoidance of future pregnancy. At 12 months following method-related discontinuation, abandoners were typically five times more likely to experience conception than switchers and, at 24 months, three times more likely. These large differences underscore the need for contraceptive counselling to alert women to the possibility of switching. We found that women living in countries with low method skewness were 28% more likely to switch than those in high skewness settings, a result that supports measures to widen the range of readily available and acceptable methods. Our results further confirm the much-acclaimed benefit of IUDs and implants in providing long term protection against pregnancy. One possible reason for this benefit is that women with a particularly strong motivation for pregnancy-avoidance may choose LARCs but a randomized trial in USA suggested that it is the characteristics of the method rather than those of the users that is the main reason [31]. Discontinuation of implants or IUDs requires the effort of a facility visit to remove the device whereas discontinuation of oral contraceptives or injectables demands nothing more than inertia. However, care must be exercised to avoid over-zealous promotion of LARCs that runs the risk of limiting women’s freedom of choice.

Further insights into the dynamics of contraceptive behaviour are unlikely to emerge from cross-sectional surveys such as DHS and, in any case, the future of such surveys is uncertain following the decision to terminate the DHS program. Longitudinal investigations to ascertain the barriers to switching that women may face is a potentially fruitful focus for future research, particularly in sub-Saharan Africa where the number of women of reproductive age is set to double in the next few decades and where switching rates are low and unmet need for family planning remains high.

## Supporting information

S1 Fig Human Development Index (HDI) for the most recent surveys since 2000. (PDF)

S2 Fig Contraceptive prevalence for the most recent surveys since 2000. (PDF)

S3 Fig Unmet need for family planning for the most recent surveys since 2000. (PDF)

S4 Fig Method skewness for the most recent surveys since 2000. (PDF)

S4 Fig Cumulative incidence of method-related discontinuation, at 12 months per 100 episodes with 95%CIs: Oral contraceptives. (PDF)

S5 Fig Trends in switching to any method at 3 months with 95%CB“, following method related discontinuation. (PDF)

S6 Fig Cumulative incidence of switching to any method, within 3 months following method-related discontinuation with 95%CIs: Oral contraceptives. (PDF)

S7 Fig Cumulative incidence of abandonment of method, within 3 months following method-related discontinuation with 95%CIs: Oral contraceptives. (PDF)

S8 Fig Trends in 12 months method abandonment with 95%CB. (PDF)

S9 Fig Reproductive consequences at 12 months following Discontinuation for method-related reasons. (PDF)

S10 Fig Reproductive consequences at 12 months following Discontinuation for method-related reasons, by country. (PDF)

S1 Table Survey samples, number of women and episodes, and percentage of women with 1,2,3,4,5 + episodes reported in the calendar during months 4–63 prior to survey date. (PDF)

S2 Table Grouping of reported contraceptive methods. (PDF)

S3 Table Grouping of reported reasons for discontinuations. (PDF)

S4 Table Comparison of all causes discontinuation at 12 months between 3 and 5 years analysis periods. (PDF)

S5 Table Number of method-specific episodes reported in the calendar during months 4–63 prior to survey date. (PDF)

S6 Table Education level, Human Development Index (HDI), unmet need for family planning, and method skewness. (PDF)

S7 Table Medians of discontinuation rates at 12 months, by method and reason. (PDF)

S8 Table 12-month overall estimated probabilities of discontinuation and reason-specific estimated cumulative incidence of discontinuation per 100 episodes of use: Oral contraceptives (OCs). (PDF)

S9 Table Trends in 12-month method-related discontinuations. (PDF)

S10 Table Medians of switching rates within 3 months following method related discontinuation, by method and status. (PDF)

S11 Table Status at 3 months following method related discontinuation: Oral contraceptives (OCs). (PDF)

S12 Table Trends in switching to any method at 3 months following method-related discontinuations. (PDF)

S13 Table Medians of 12-month abandonment following method-related discontinuation, by method. (PDF)

S14 Table 12-month cumulative incidence of abandonment following method-related discontinuation per 100 episodes of use: Oral contraceptives (OCs). (PDF)

S15 Table Trends in 12-month method abandonment. (PDF)

S16 Table Cumulative incidence of wanted and unwanted conceptions at 12 months following discontinuation. (PDF)

S17 Table Conception rates at 12 and 24 months, by switchers and abandoners, After method-related discontinuation within 12 months of use. (PDF)

S18 Table Ranking countries by 12-month conception rates and 12-month discontinuation for method-related reasons. (PDF)
