# Supplementary material for: Contraceptive discontinuation, switching, abandonment and their reproductive consequences: An analysis of 1,539,071 episodes of reversible method use contributed from 61 countries that participated in DHS: Population base-analysis
Source: PLOS Glob Public Health. 2025 Oct 31;5(10):e0005174. doi: 10.1371/journal.pgph.0005174 (PMC12578211; doi:10.1371/journal.pgph.0005174)

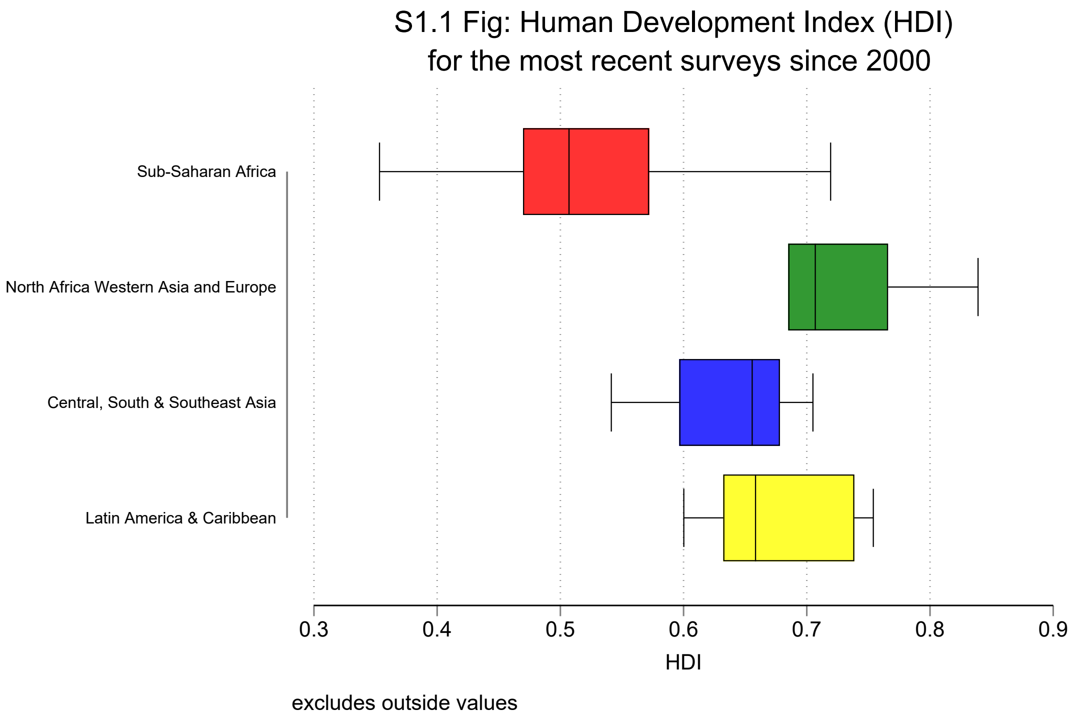
S1.1 Fig: Human Development Index (HDI)
for the most recent surveys since 2000
Sub-Saharan Africa
North Africa Western Asia and Europe
Central, South & Southeast Asia
Latin America & Caribbean
0.3
0.4
0.5
0.6
0.7
0.8
0.9
HDI
excludes outside values

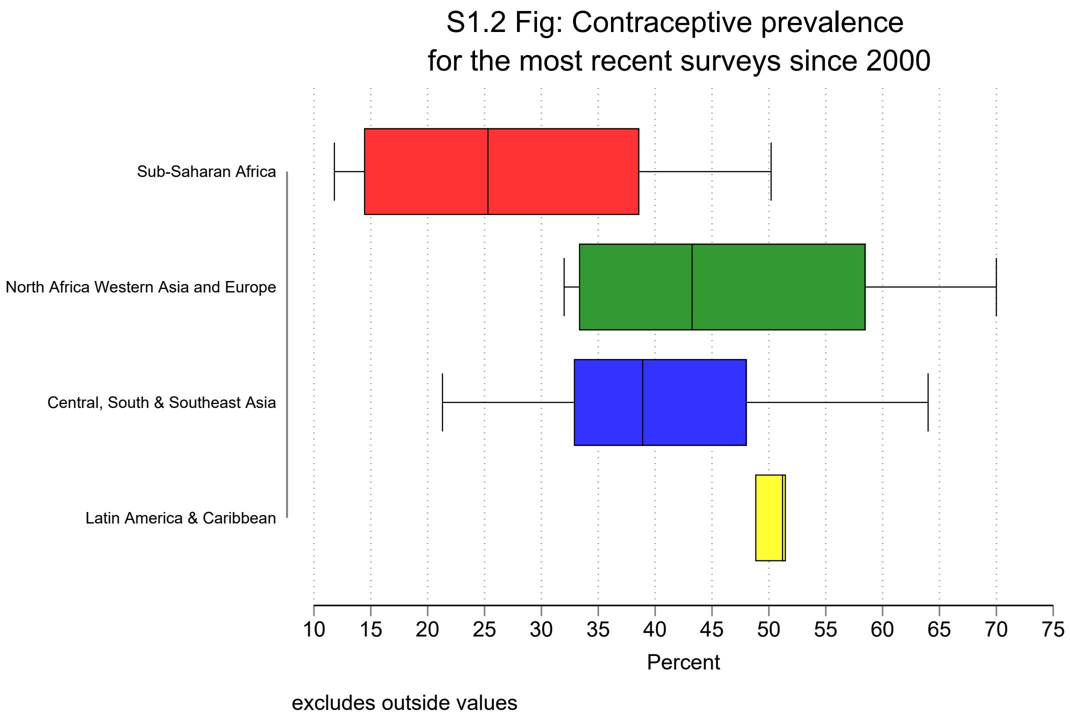

S1.2 Fig: Contraceptive prevalence
for the most recent surveys since 2000
Sub-Saharan Africa
North Africa Western Asia and Europe
Central, South & Southeast Asia
Latin America & Caribbean
10
15
20
25
30
35
40
45
50
55
60
65
70
75
Percent
excludes outside values

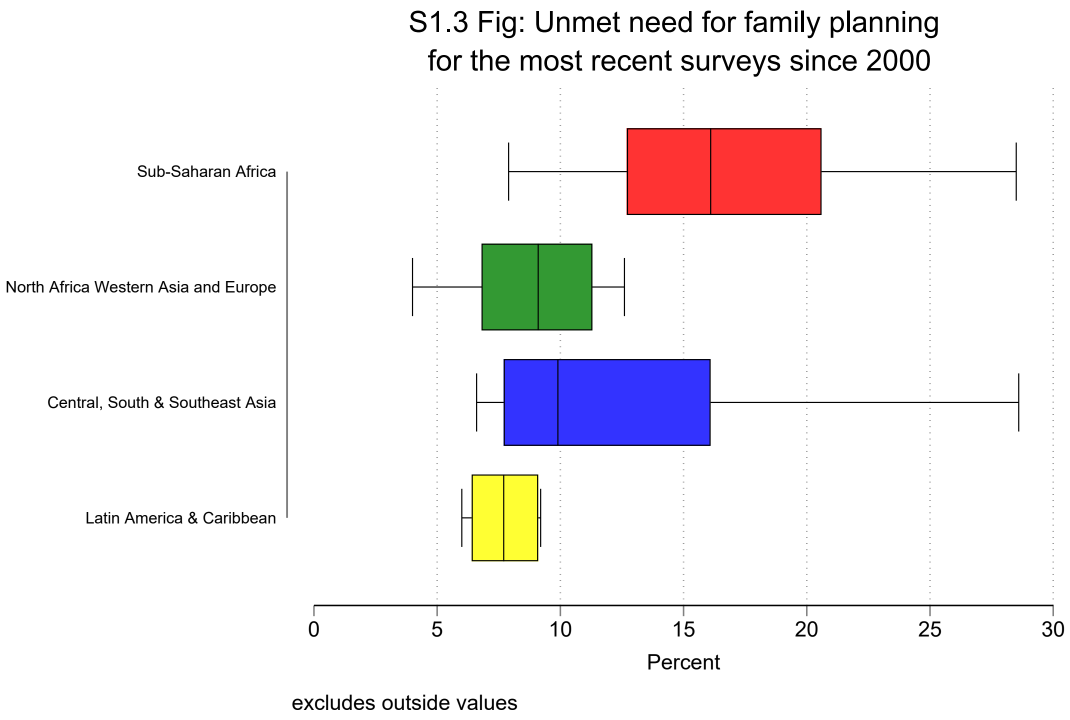
S1.3 Fig: Unmet need for family planning
for the most recent surveys since 2000
Sub-Saharan Africa
North Africa Western Asia and Europe
Central, South & Southeast Asia
Latin America & Caribbean
0
5
10
15
20
25
30
Percent
excludes outside values

S1.4 Fig: Method skewness for the most recent surveys since 2000

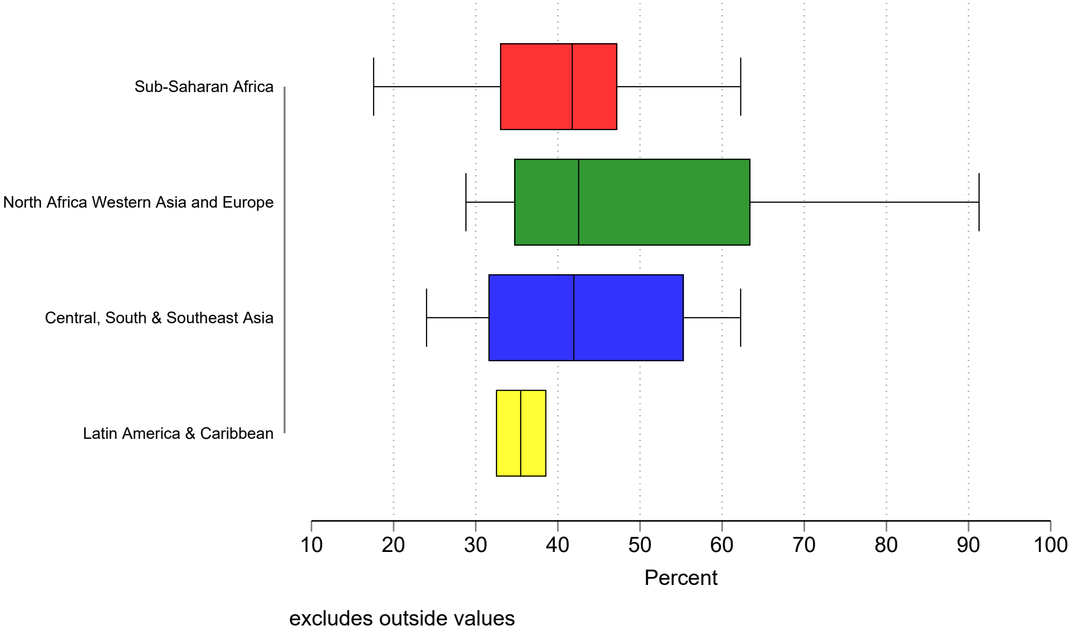

Supplement: S1 Fig — (PDF) [file pgph.0005174.s001.pdf]
