# Supplementary material for: Contraceptive discontinuation, switching, abandonment and their reproductive consequences: An analysis of 1,539,071 episodes of reversible method use contributed from 61 countries that participated in DHS: Population base-analysis
Source: PLOS Glob Public Health. 2025 Oct 31;5(10):e0005174. doi: 10.1371/journal.pgph.0005174 (PMC12578211; doi:10.1371/journal.pgph.0005174)
Supplement: S2 Fig — (PDF) [file pgph.0005174.s002.pdf]

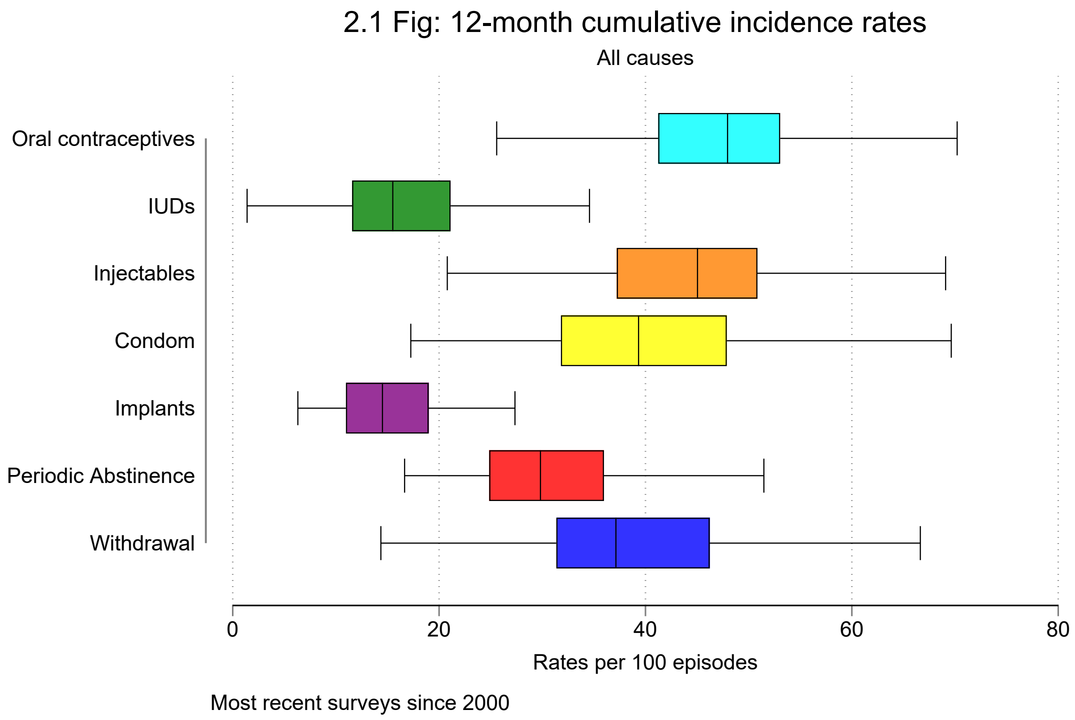
2.1 Fig: 12-month cumulative incidence rates
All causes
Oral contraceptives
IUDs
Injectables
Condom
Implants
Periodic Abstinence
Withdrawal
0
20
40
60
80
Rates per 100 episodes
Most recent surveys since 2000

2.2 Fig: 12-month cumulative incidence rates

Became pregnant while using

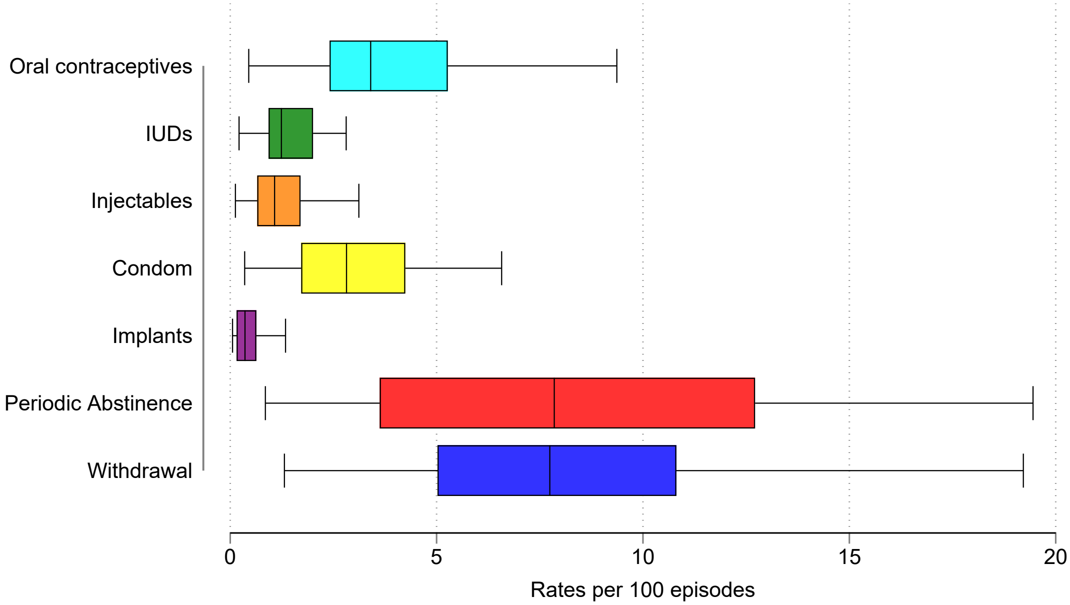

Most recent surveys since 2000

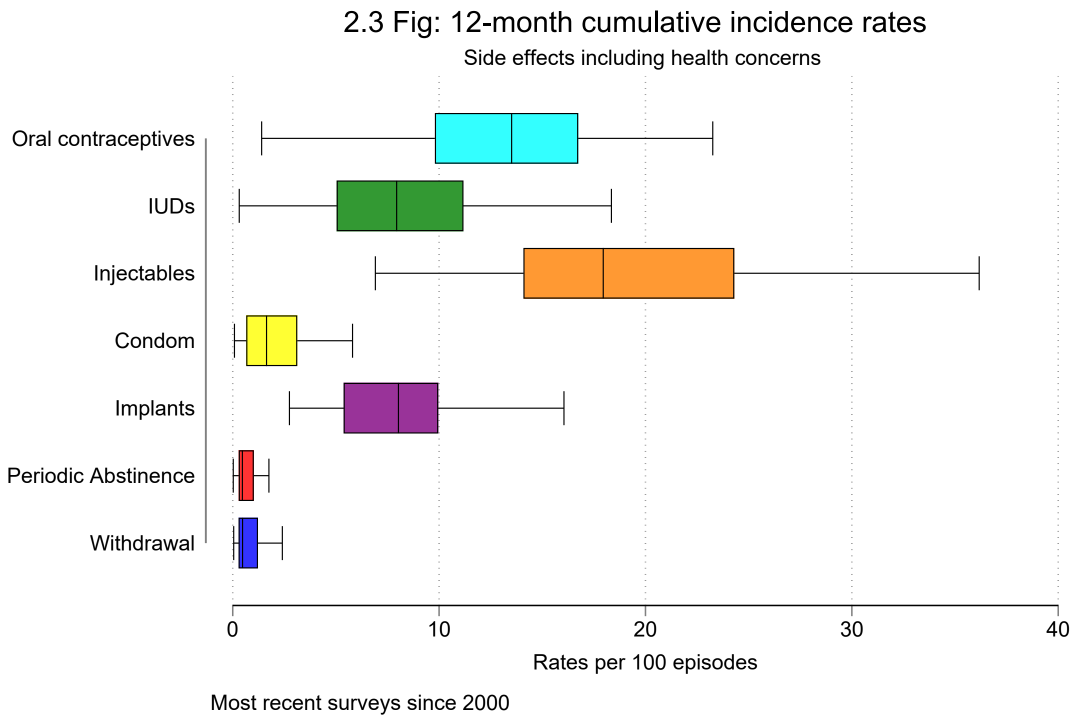

2.3 Fig: 12-month cumulative incidence rates
Side effects including health concerns
Oral contraceptives
IUDs
Injectables
Condom
Implants
Periodic Abstinence
Withdrawal
0
10
20
30
40
Rates per 100 episodes
Most recent surveys since 2000

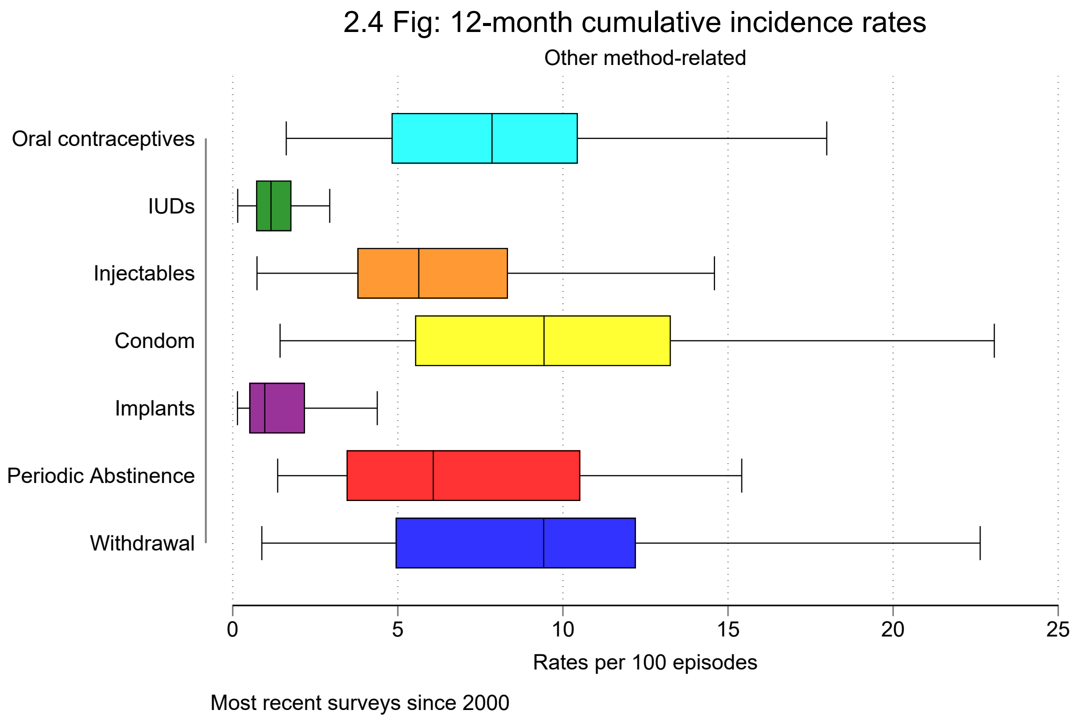
2.4 Fig: 12-month cumulative incidence rates
Other method-related
Oral contraceptives
IUDs
Injectables
Condom
Implants
Periodic Abstinence
Withdrawal
0
5
10
15
20
25
Rates per 100 episodes
Most recent surveys since 2000

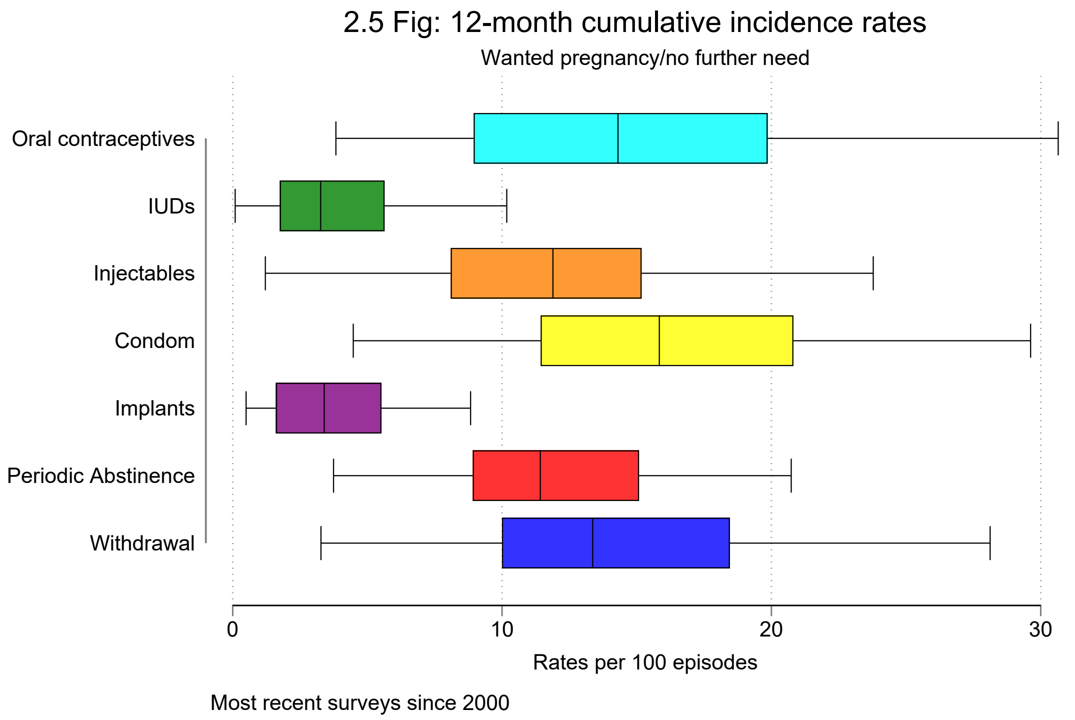
2.5 Fig: 12-month cumulative incidence rates
Wanted pregnancy/no further need
Oral contraceptives
IUDs
Injectables
Condom
Implants
Periodic Abstinence
Withdrawal
0
10
20
30
Rates per 100 episodes
Most recent surveys since 2000

2.6 Fig: 12-month cumulative incidence rates

Other or not stated reasons

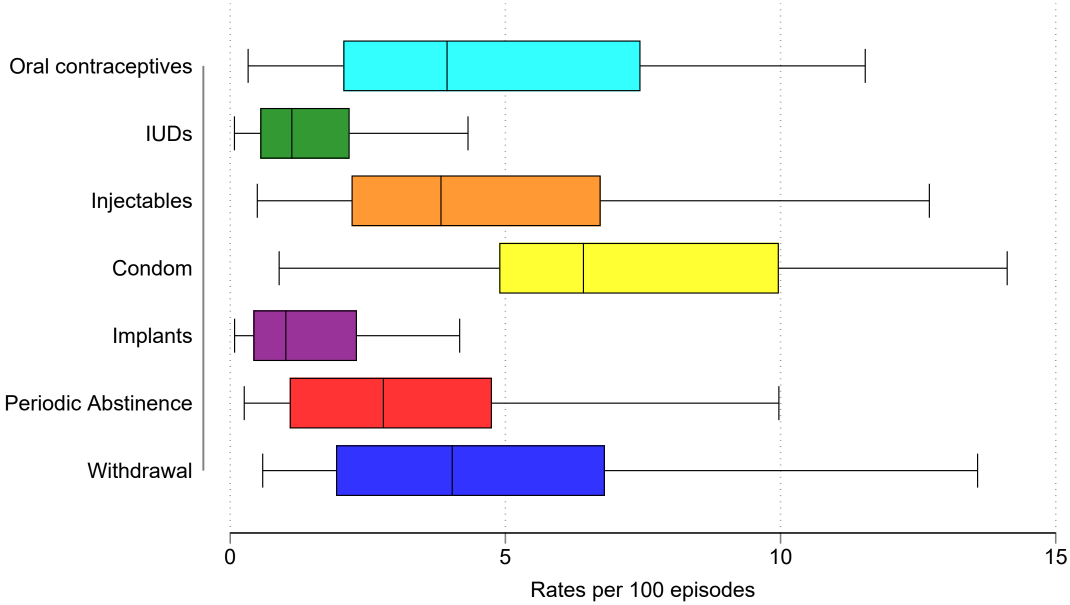

Most recent surveys since 2000
