# Supplementary material for: Contraceptive discontinuation, switching, abandonment and their reproductive consequences: An analysis of 1,539,071 episodes of reversible method use contributed from 61 countries that participated in DHS: Population base-analysis
Source: PLOS Glob Public Health. 2025 Oct 31;5(10):e0005174. doi: 10.1371/journal.pgph.0005174 (PMC12578211; doi:10.1371/journal.pgph.0005174)

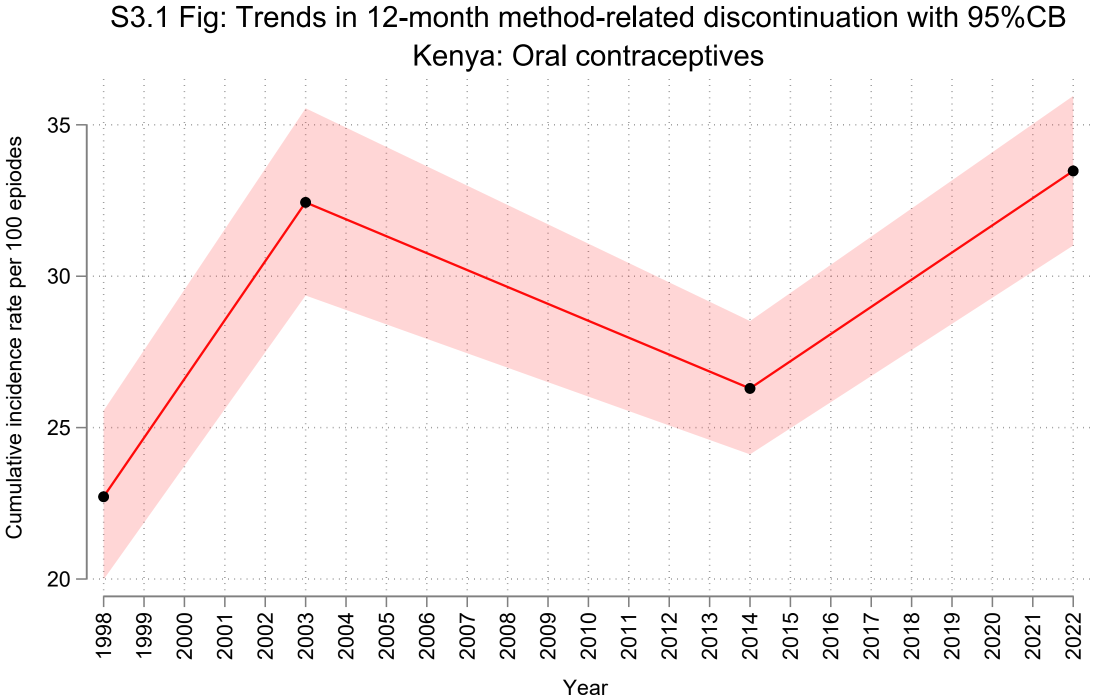
S3.1 Fig: Trends in 12-month method-related discontinuation with 95%CB
Kenya: Oral contraceptives
Cumulative incidence rate per 100 epiodes
35
30
25
20
1998
1999
2000
2001
2002
2003
2004
2005
2006
2007
2008
2009
2010
2011
2012
2013
2014
2015
2016
2017
2018
2019
2020
2021
2022
Year

S3.2 Fig: Trends in 12-month method-related discontinuation with 95%CB
Kenya: IUD

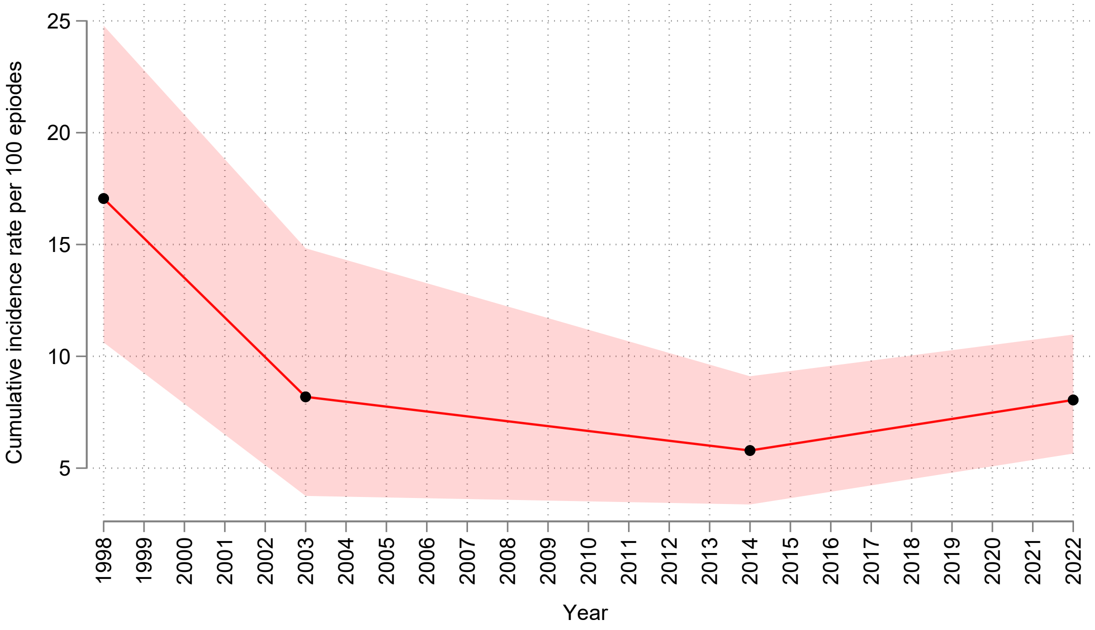

S3.3 Fig: Trends in 12-month method-related discontinuation with 95%CB
Kenya: Injectables

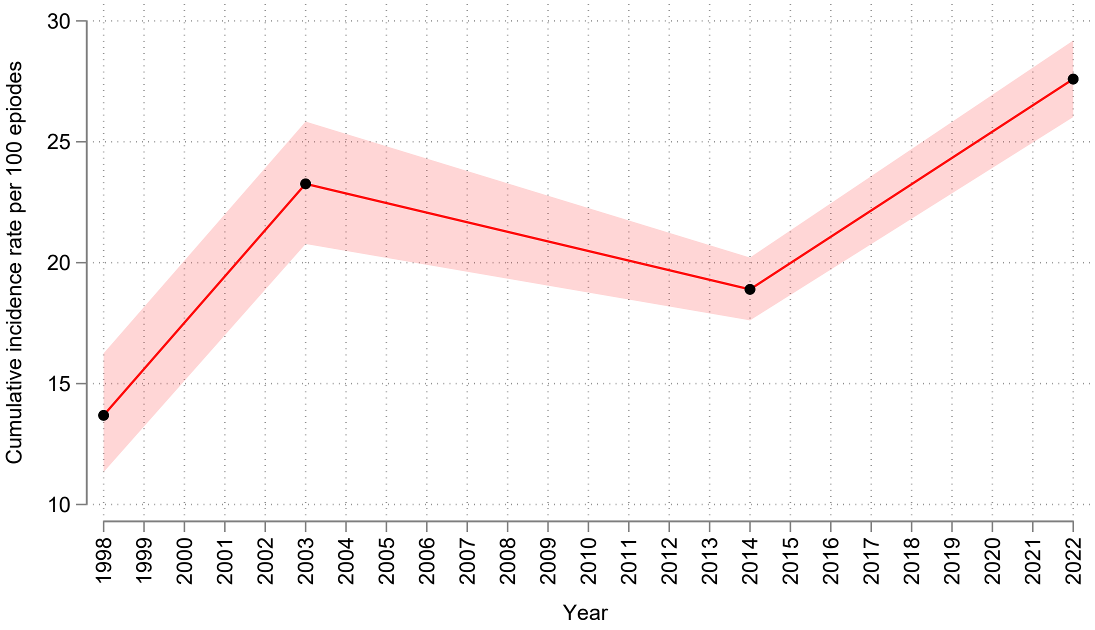

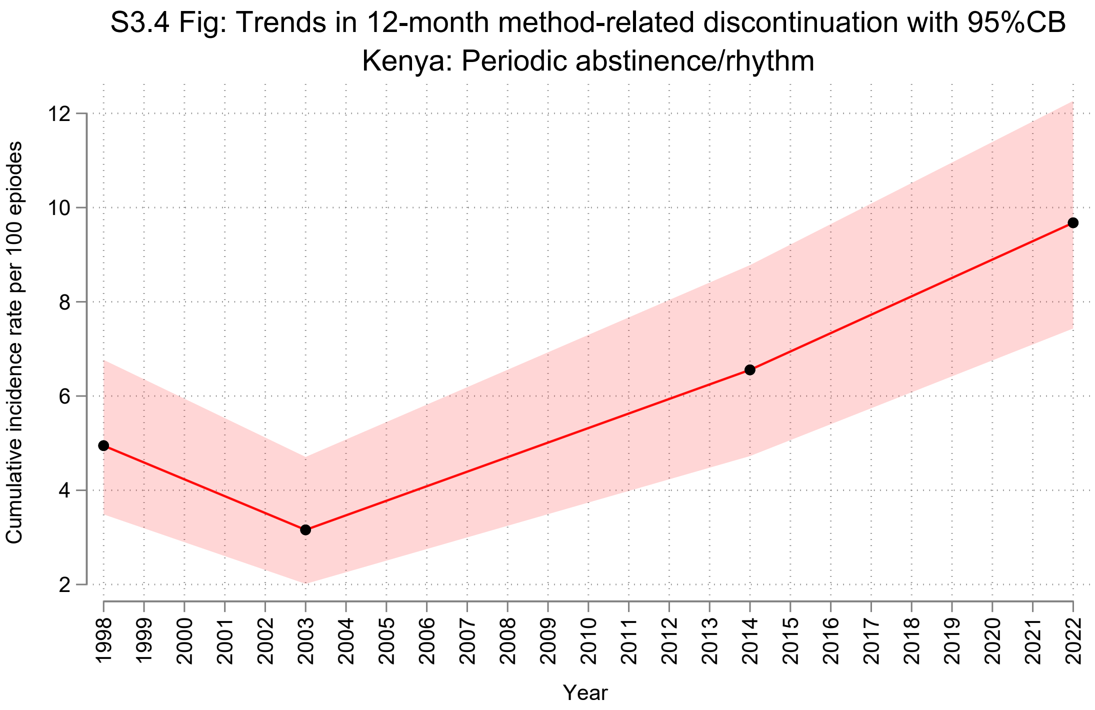
S3.4 Fig: Trends in 12-month method-related discontinuation with 95%CB
Kenya: Periodic abstinence/rhythm
Cumulative incidence rate per 100 epiodes
2
4
6
8
10
12
1998
1999
2000
2001
2002
2003
2004
2005
2006
2007
2008
2009
2010
2011
2012
2013
2014
2015
2016
2017
2018
2019
2020
2021
2022
Year

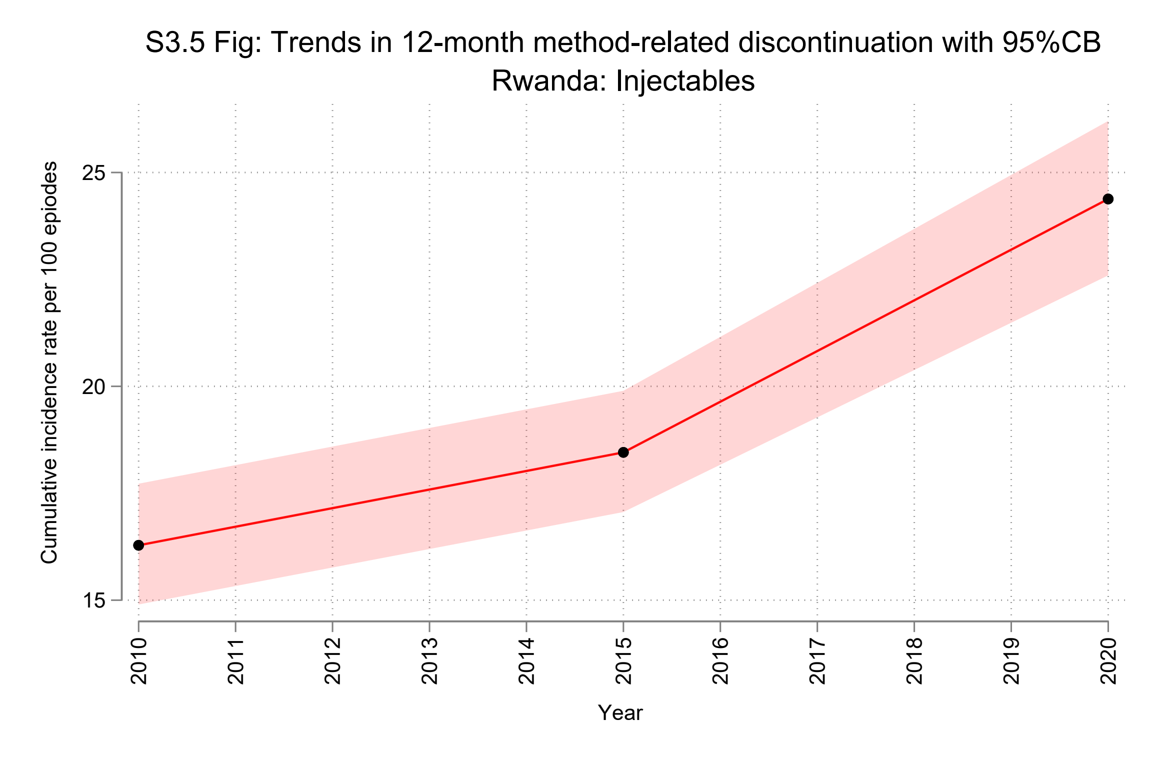
S3.5 Fig: Trends in 12-month method-related discontinuation with 95%CB
Rwanda: Injectables
Cumulative incidence rate per 100 epiodes
25
20
15
2010
2011
2012
2013
2014
2015
2016
2017
2018
2019
2020
Year

S3.6 Fig: Trends in 12-month method-related discontinuation with 95%CB
Rwanda: Condom

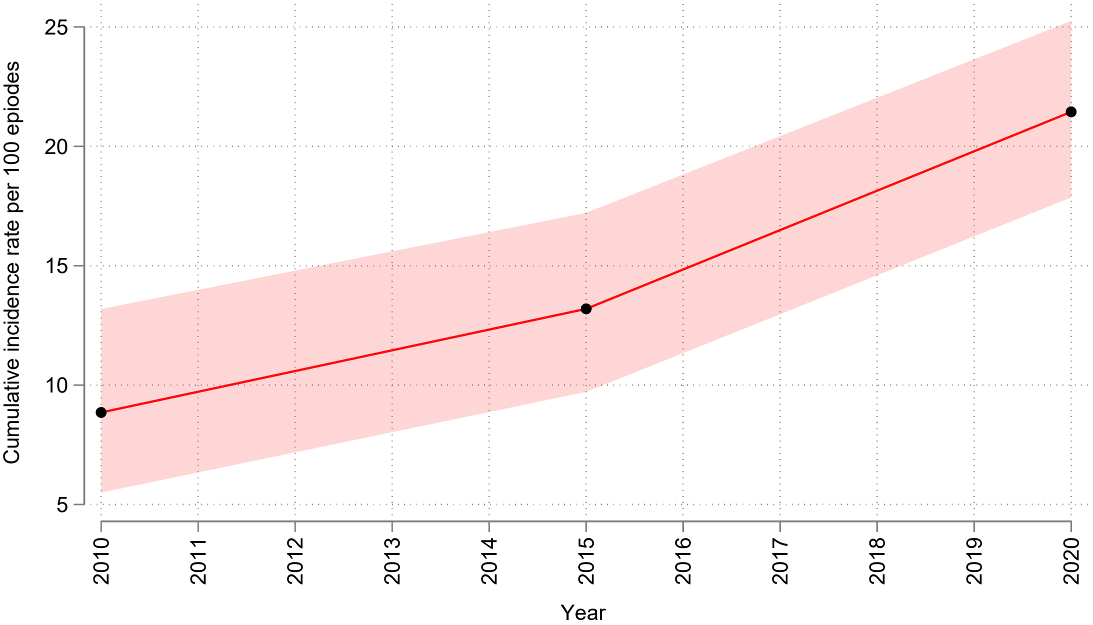

S3.7 Fig: Trends in 12-month method-related discontinuation with 95%CB
Rwanda: Withdrawal

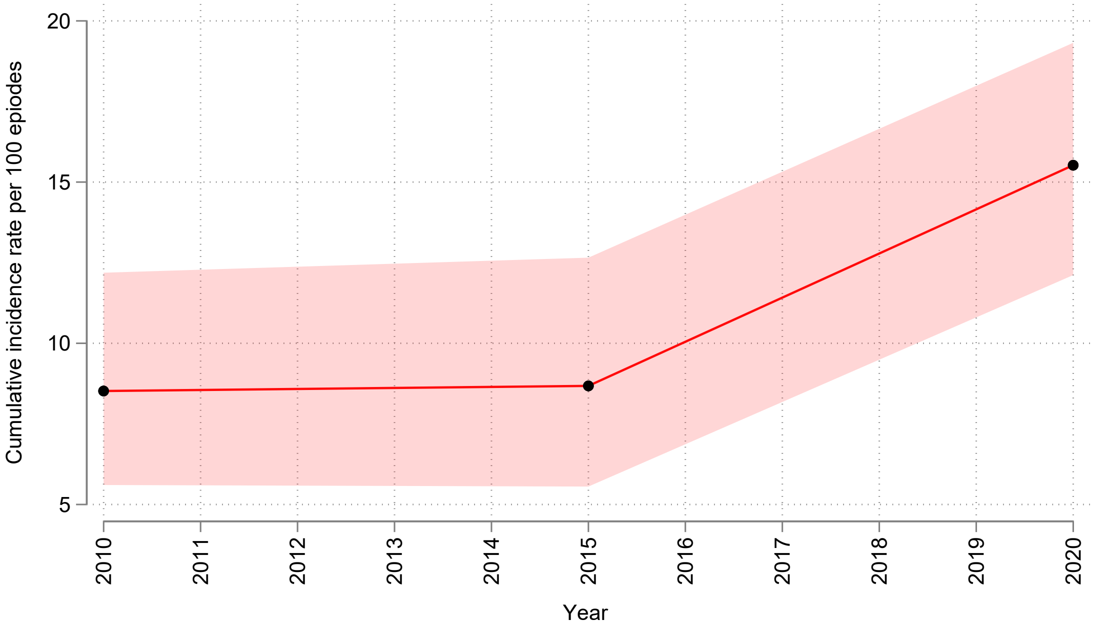

S3.8 Fig: Trends in 12-month method-related discontinuation with 95%CB
Senegal: Oral contraceptives

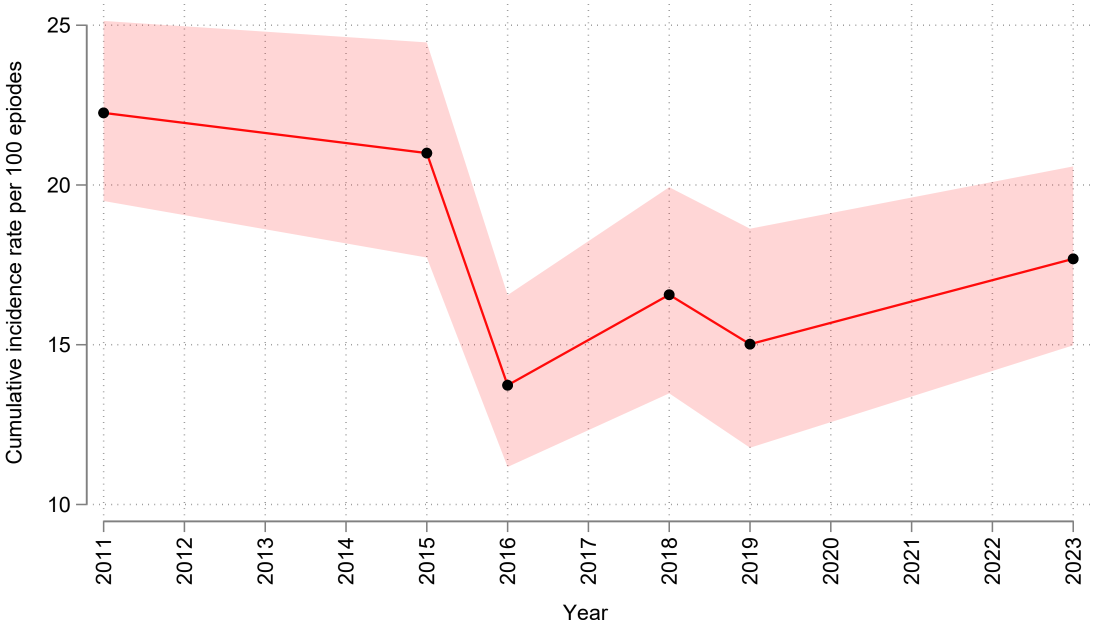

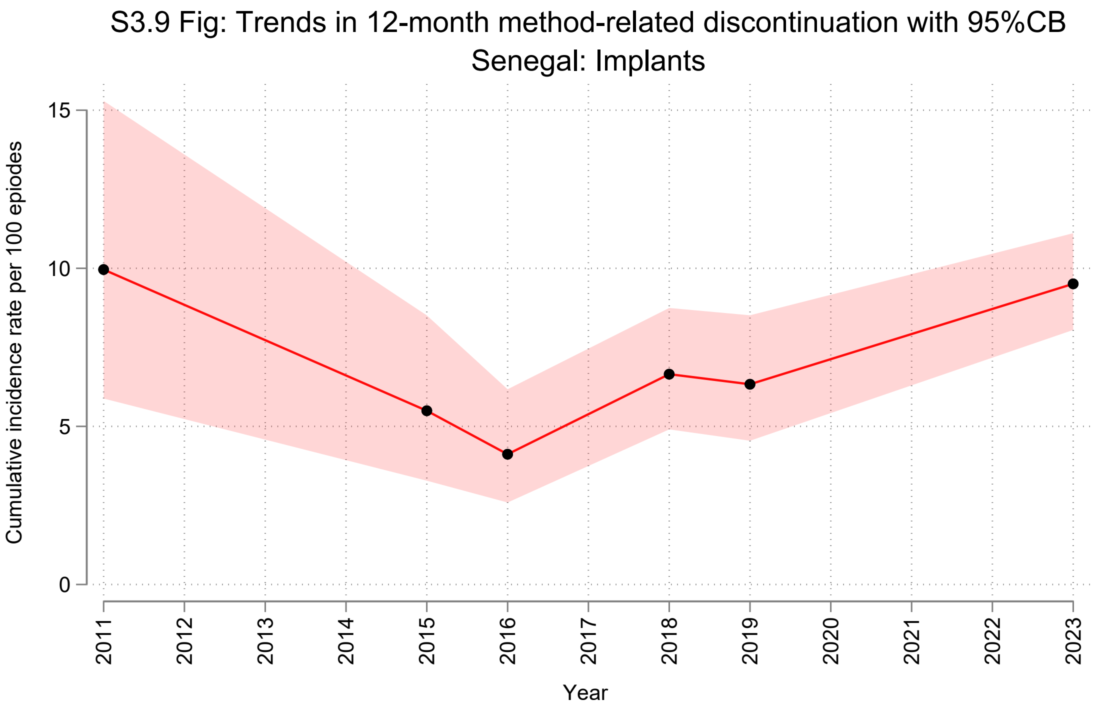
S3.9 Fig: Trends in 12-month method-related discontinuation with 95%CB
Senegal: Implants
Cumulative incidence rate per 100 epiodes
15
10
5
0
2011
2012
2013
2014
2015
2016
2017
2018
2019
2020
2021
2022
2023
Year

S3.10 Fig: Trends in 12-month method-related discontinuation with 95%CB
Tanzania: Condom

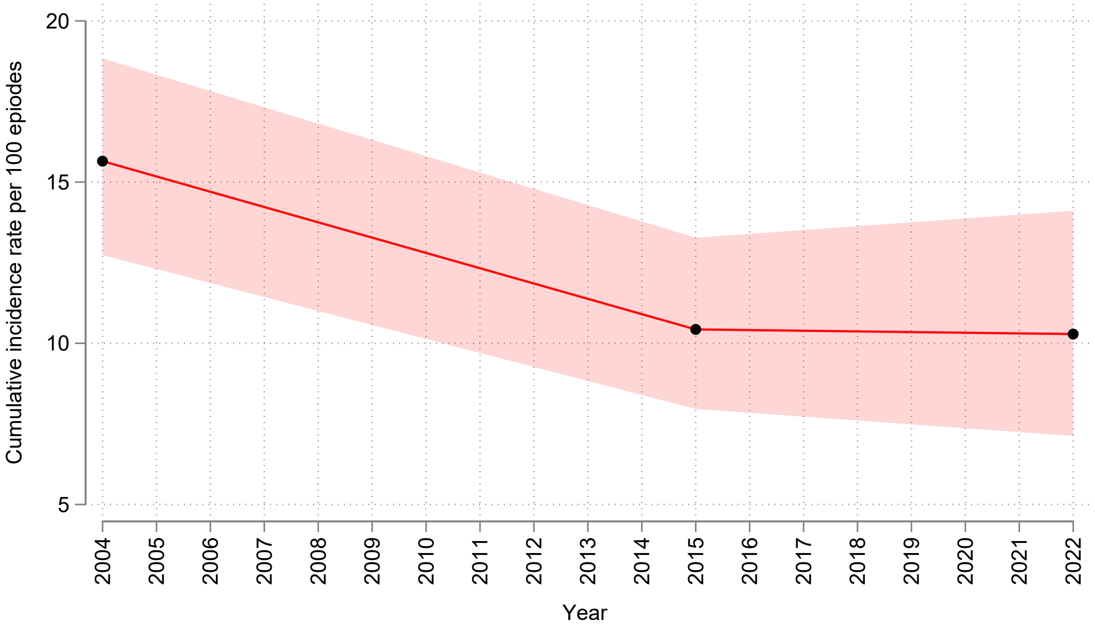

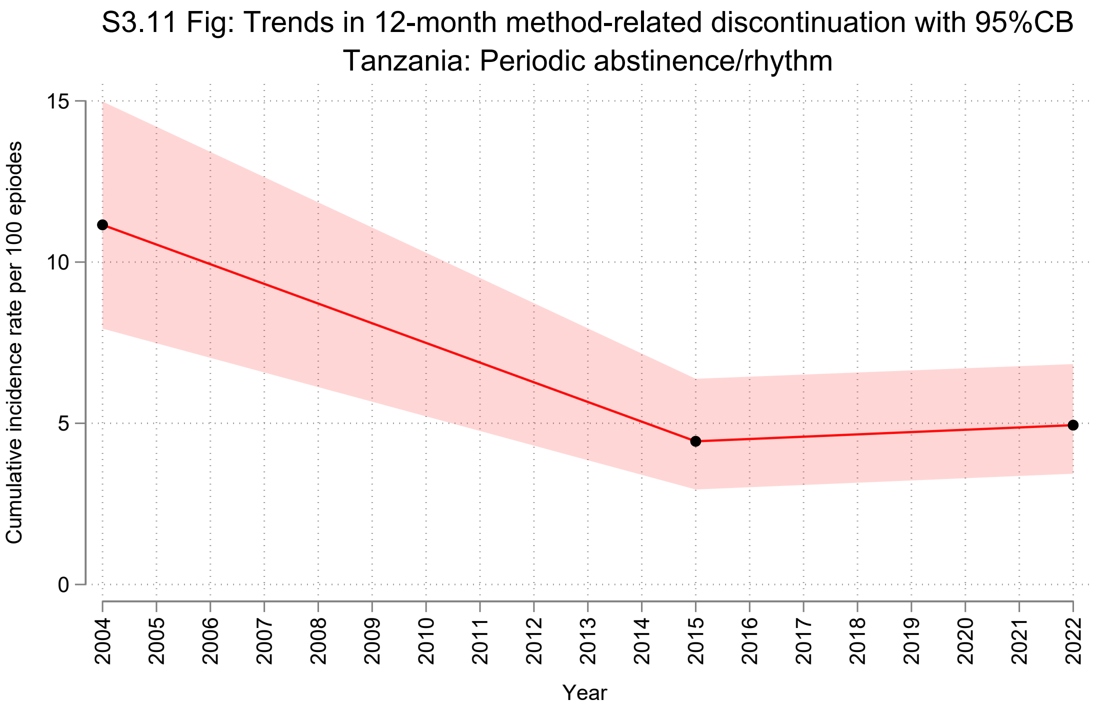
S3.11 Fig: Trends in 12-month method-related discontinuation with 95%CB
Tanzania: Periodic abstinence/rhythm
Cumulative incidence rate per 100 epiodes
15
10
5
0
2004
2005
2006
2007
2008
2009
2010
2011
2012
2013
2014
2015
2016
2017
2018
2019
2020
2021
2022
Year

S3.12 Fig: Trends in 12-month method-related discontinuation with 95%CB
Zimbabwe: Oral contraceptives

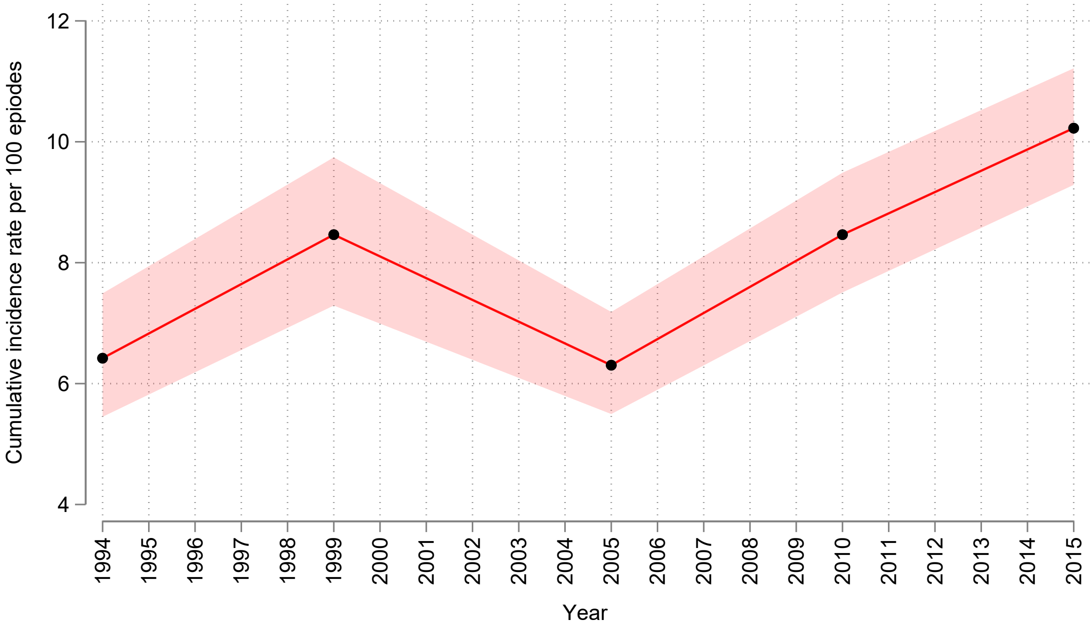

S3.13 Fig: Trends in 12-month method-related discontinuation with 95%CB
Zimbabwe: Injectables

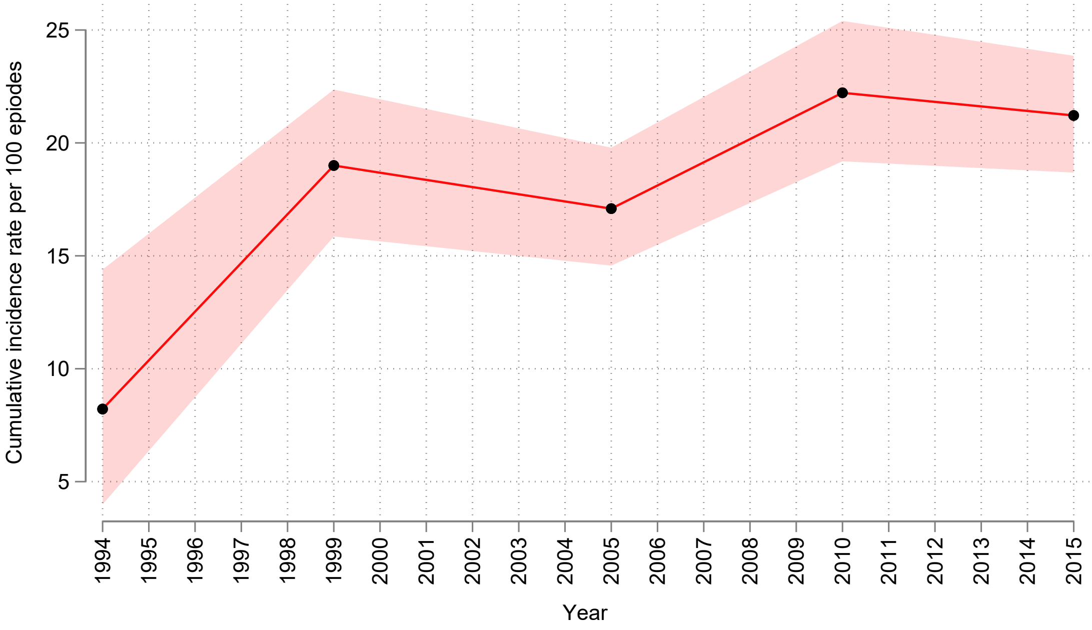

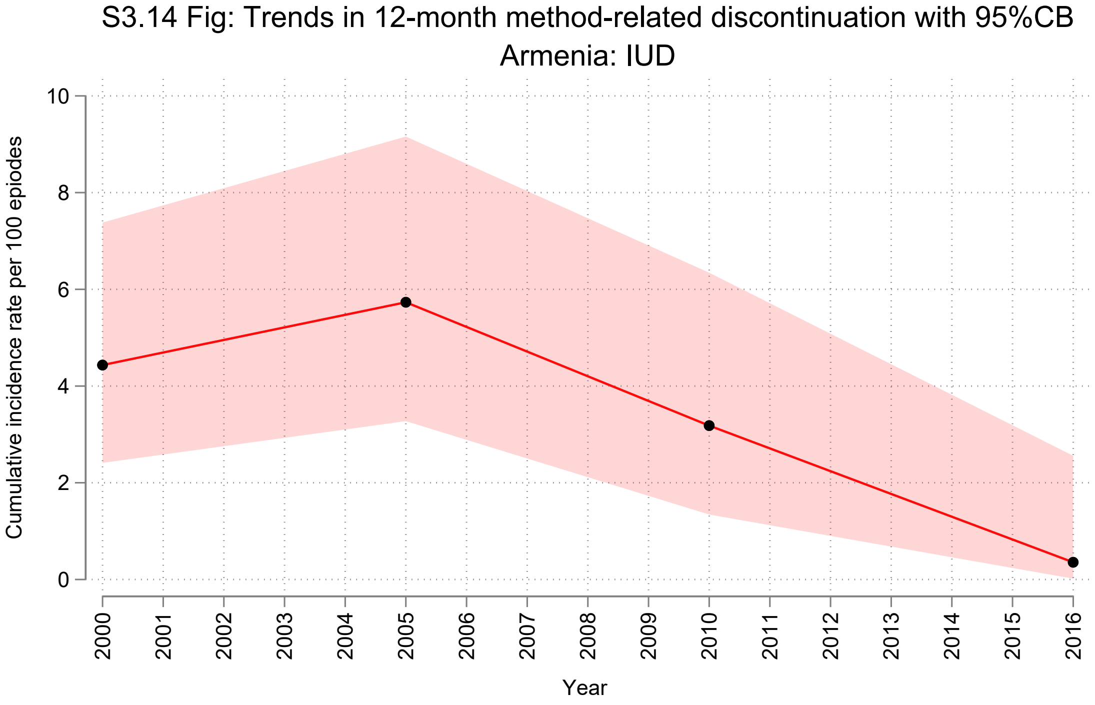
S3.14 Fig: Trends in 12-month method-related discontinuation with 95%CB
Armenia: IUD
Cumulative incidence rate per 100 epiodes
0
2
4
6
8
10
2000
2001
2002
2003
2004
2005
2006
2007
2008
2009
2010
2011
2012
2013
2014
2015
2016
Year

S3.15 Fig: Trends in 12-month method-related discontinuation with 95%CB
Armenia: Condom

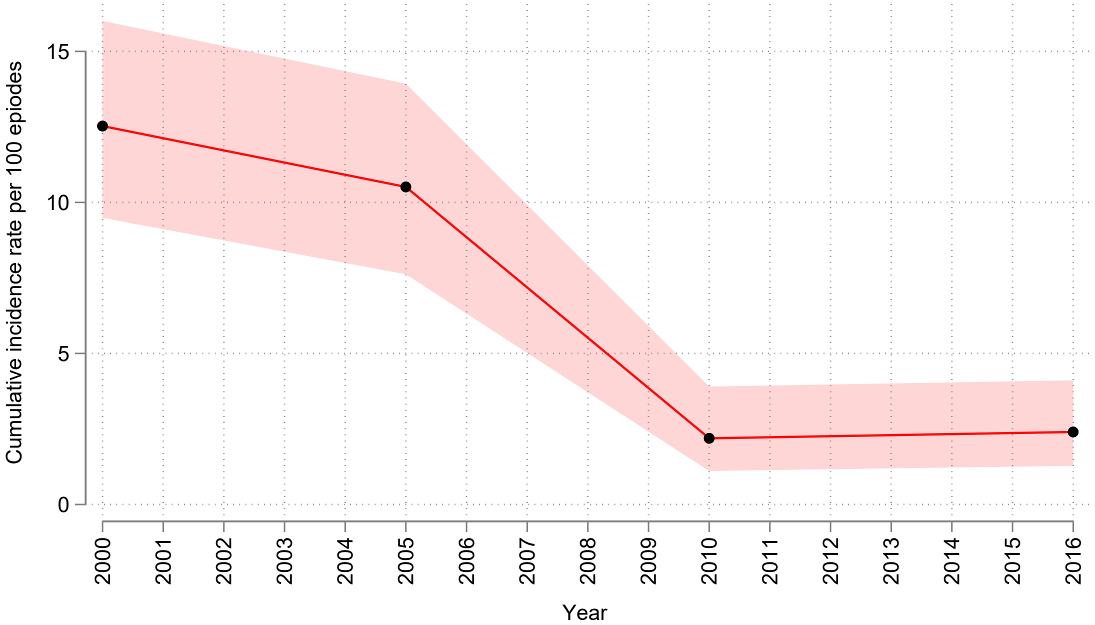

S3.16 Fig: Trends in 12-month method-related discontinuation with 95%CB
Egypt: Oral contraceptives

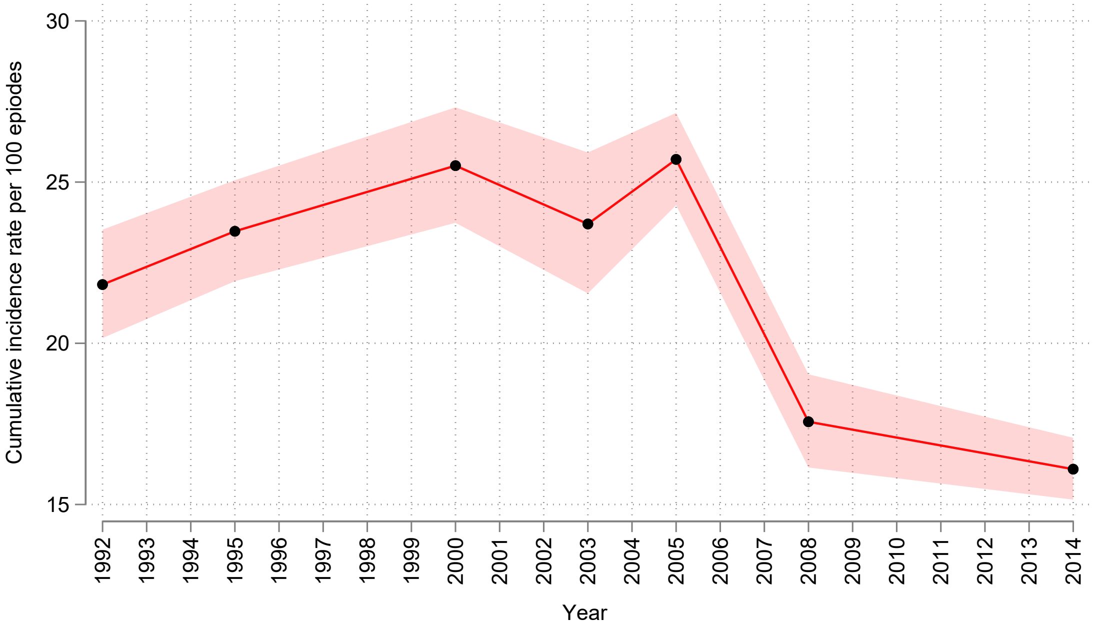

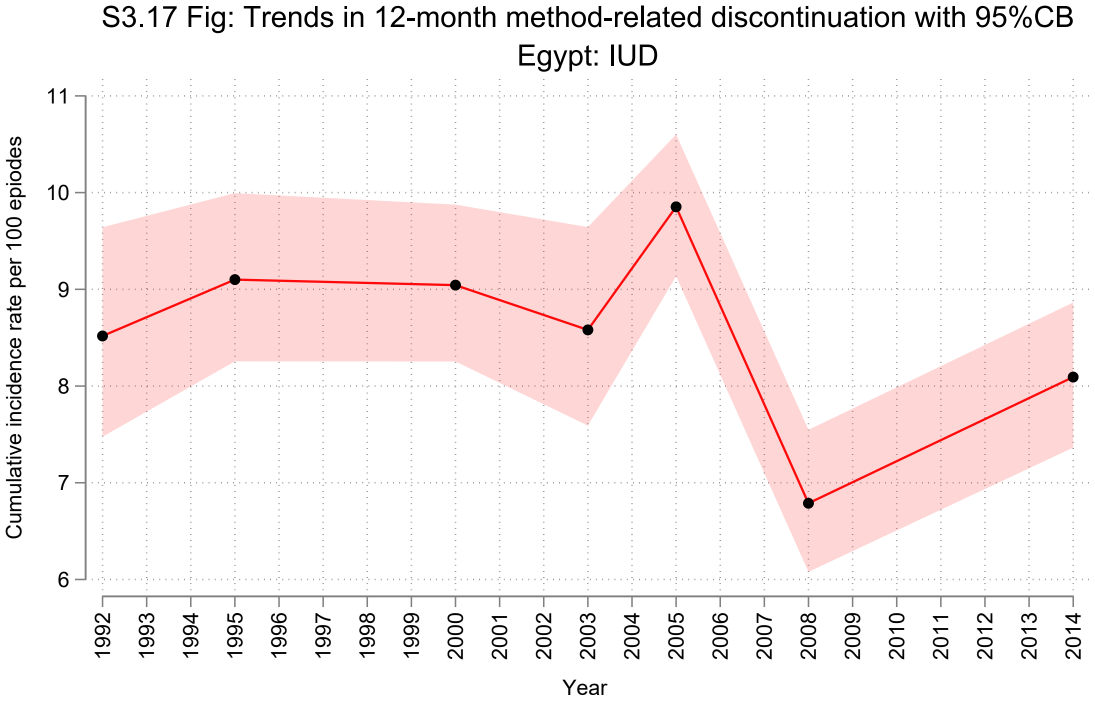
S3.17 Fig: Trends in 12-month method-related discontinuation with 95%CB
Egypt: IUD
Cumulative incidence rate per 100 episodes
11
10
9
8
7
6
1992
1993
1994
1995
1996
1997
1998
1999
2000
2001
2002
2003
2004
2005
2006
2007
2008
2009
2010
2011
2012
2013
2014
Year

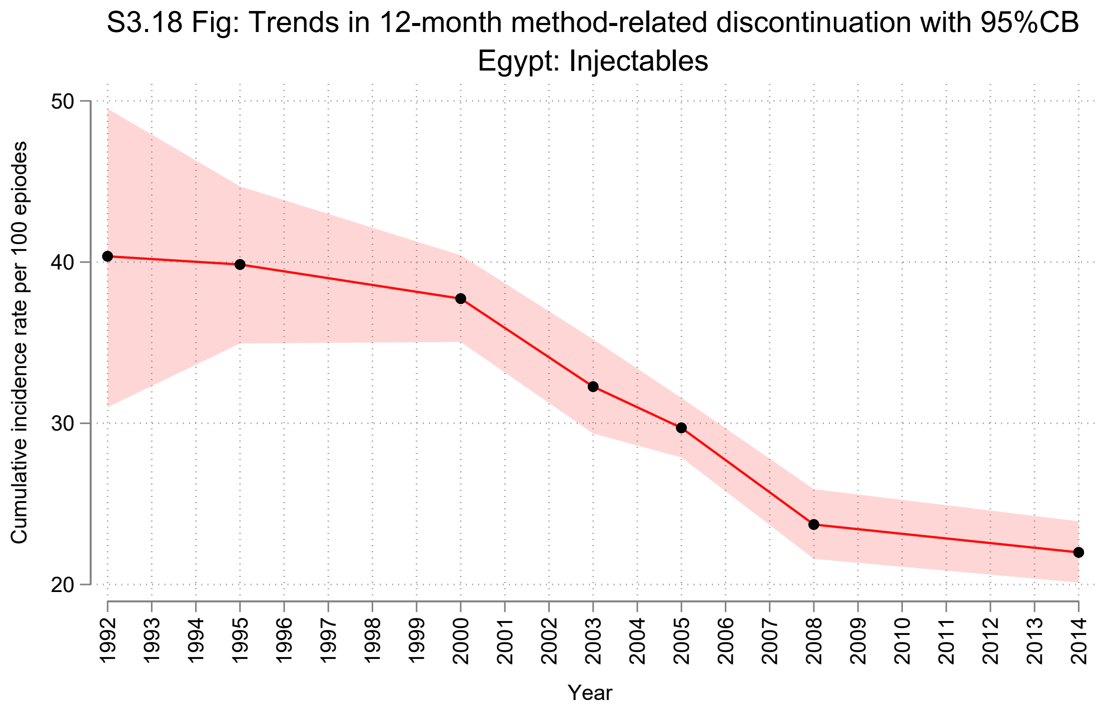
S3.18 Fig: Trends in 12-month method-related discontinuation with 95%CB
Egypt: Injectables
Cumulative incidence rate per 100 epiodes
50
40
30
20
1992
1993
1994
1995
1996
1997
1998
1999
2000
2001
2002
2003
2004
2005
2006
2007
2008
2009
2010
2011
2012
2013
2014
Year

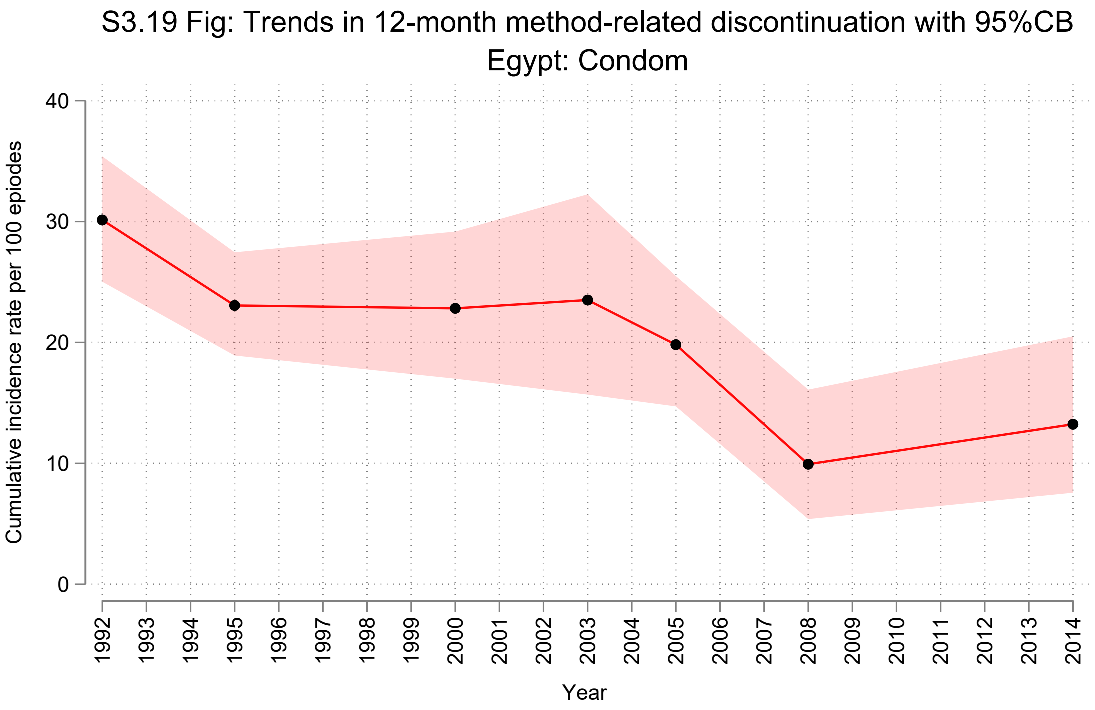
S3.19 Fig: Trends in 12-month method-related discontinuation with 95%CB
Egypt: Condom
Cumulative incidence rate per 100 epiodes
0
10
20
30
40
1992
1993
1994
1995
1996
1997
1998
1999
2000
2001
2002
2003
2004
2005
2006
2007
2008
2009
2010
2011
2012
2013
2014
Year

S3.20 Fig: Trends in 12-month method-related discontinuation with 95%CB
Jordan: Oral contraceptives

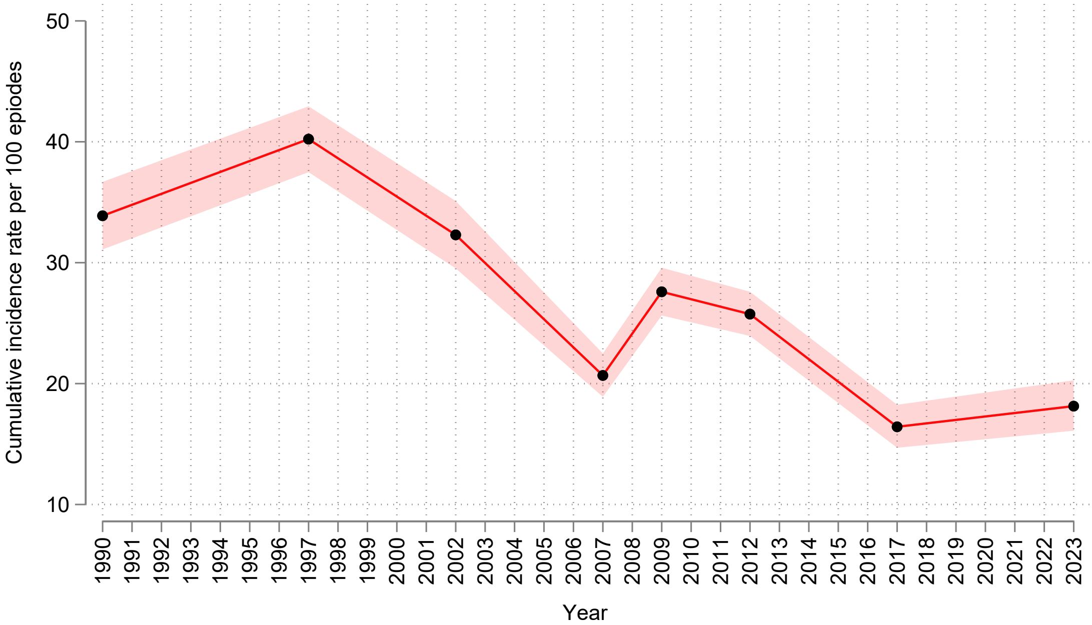

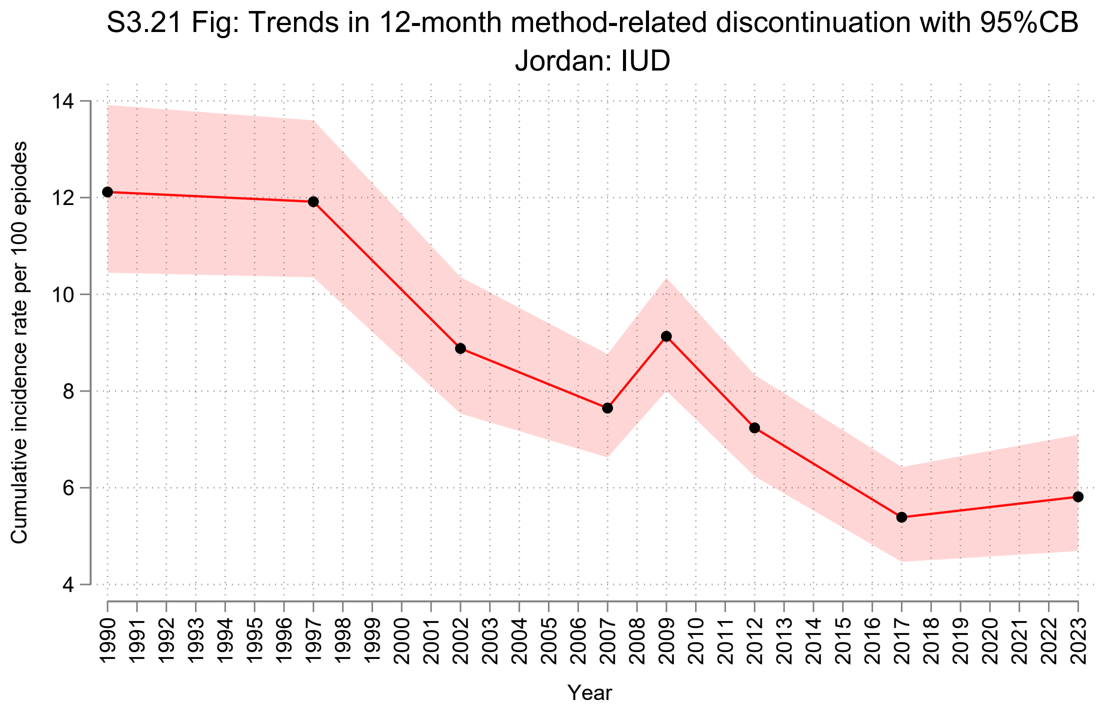
S3.21 Fig: Trends in 12-month method-related discontinuation with 95%CB
Jordan: IUD
Cumulative incidence rate per 100 episodes
4
6
8
10
12
14
1990
1991
1992
1993
1994
1995
1996
1997
1998
1999
2000
2001
2002
2003
2004
2005
2006
2007
2008
2009
2010
2011
2012
2013
2014
2015
2016
2017
2018
2019
2020
2021
2022
2023
Year

S3.22 Fig: Trends in 12-month method-related discontinuation with 95%CB
Jordan: Injectables

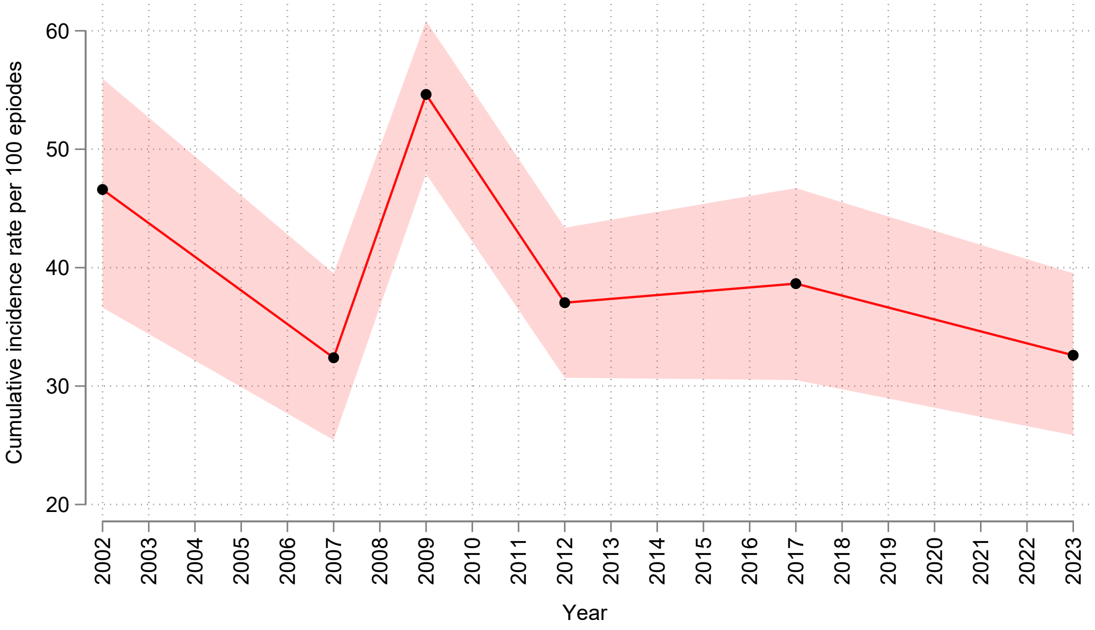

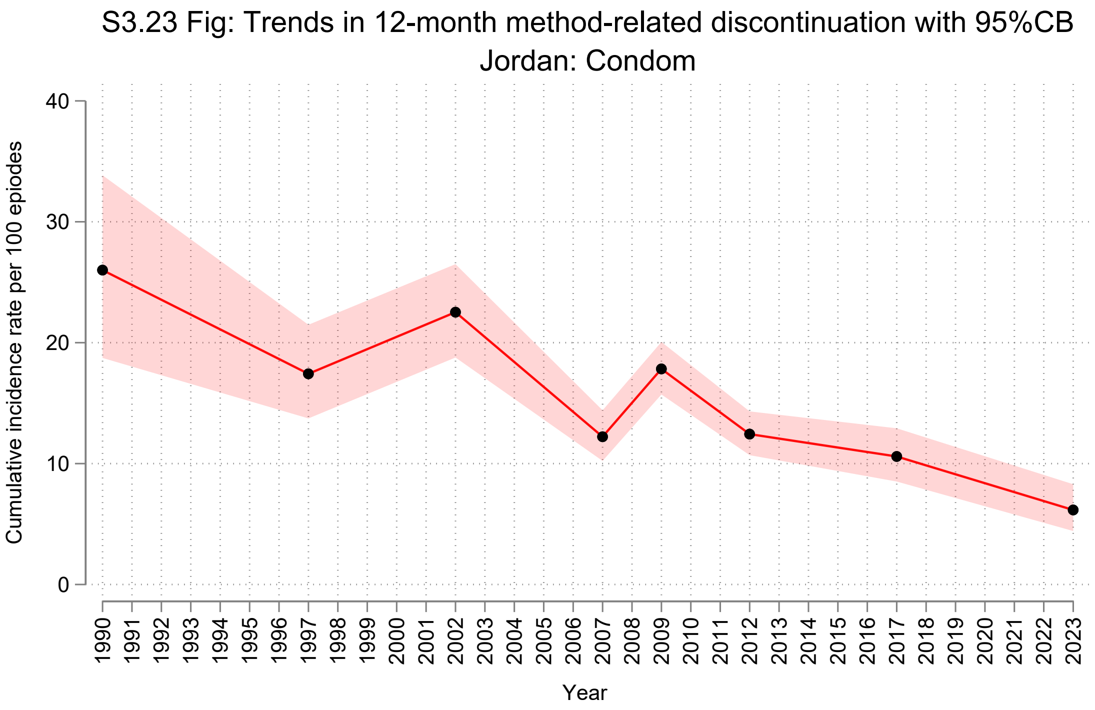
S3.23 Fig: Trends in 12-month method-related discontinuation with 95%CB
Jordan: Condom
Cumulative incidence rate per 100 epiodes
0
10
20
30
40
Year
1990
1991
1992
1993
1994
1995
1996
1997
1998
1999
2000
2001
2002
2003
2004
2005
2006
2007
2008
2009
2010
2011
2012
2013
2014
2015
2016
2017
2018
2019
2020
2021
2022
2023

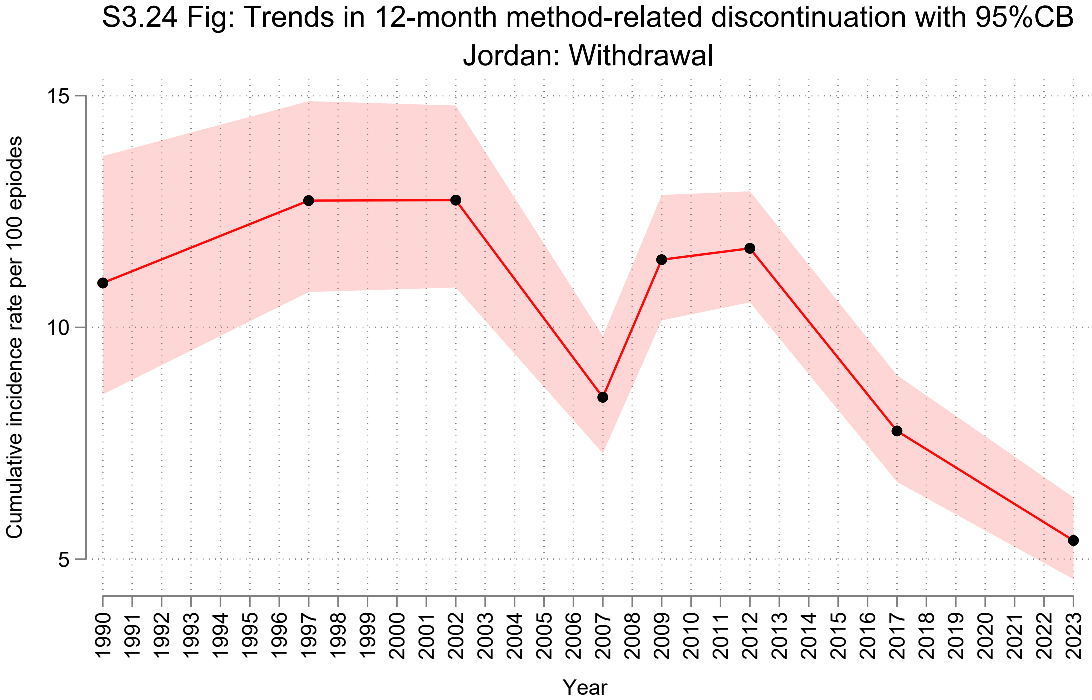
S3.24 Fig: Trends in 12-month method-related discontinuation with 95%CB
Jordan: Withdrawal
Cumulative incidence rate per 100 epiodes
15
10
5
1990
1991
1992
1993
1994
1995
1996
1997
1998
1999
2000
2001
2002
2003
2004
2005
2006
2007
2008
2009
2010
2011
2012
2013
2014
2015
2016
2017
2018
2019
2020
2021
2022
2023
Year

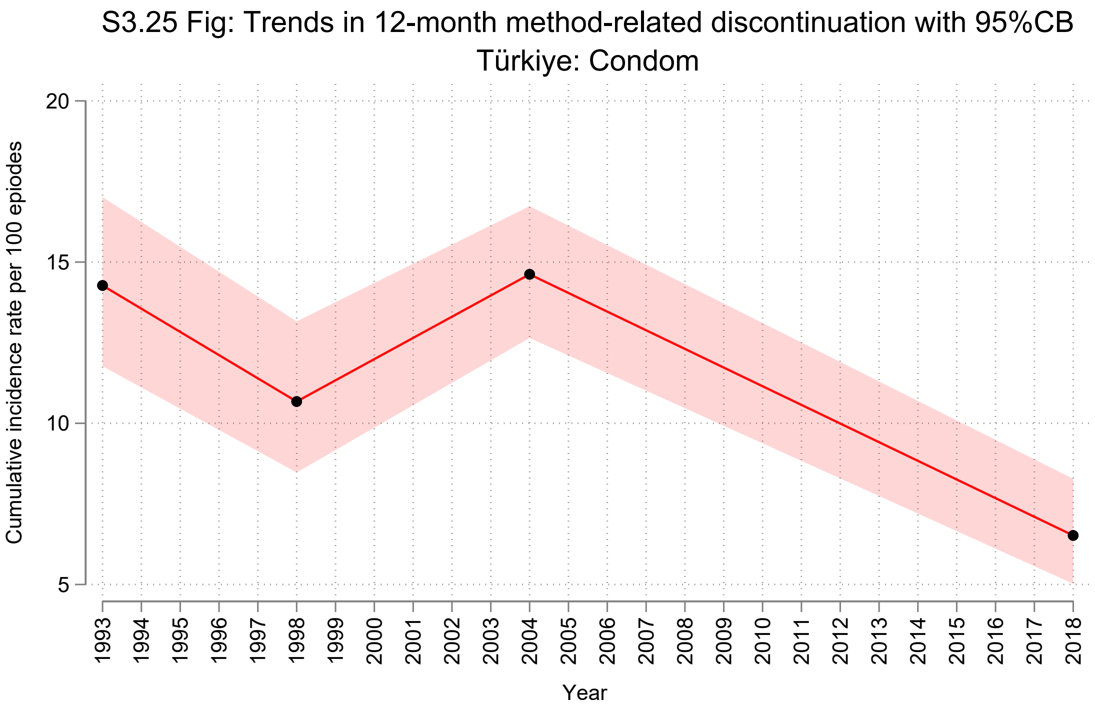
S3.25 Fig: Trends in 12-month method-related discontinuation with 95%CB
Türkiye: Condom
Cumulative incidence rate per 100 epiodes
20
15
10
5
1993
1994
1995
1996
1997
1998
1999
2000
2001
2002
2003
2004
2005
2006
2007
2008
2009
2010
2011
2012
2013
2014
2015
2016
2017
2018
Year

S3.26 Fig: Trends in 12-month method-related discontinuation with 95%CB
Türkiye: Withdrawal

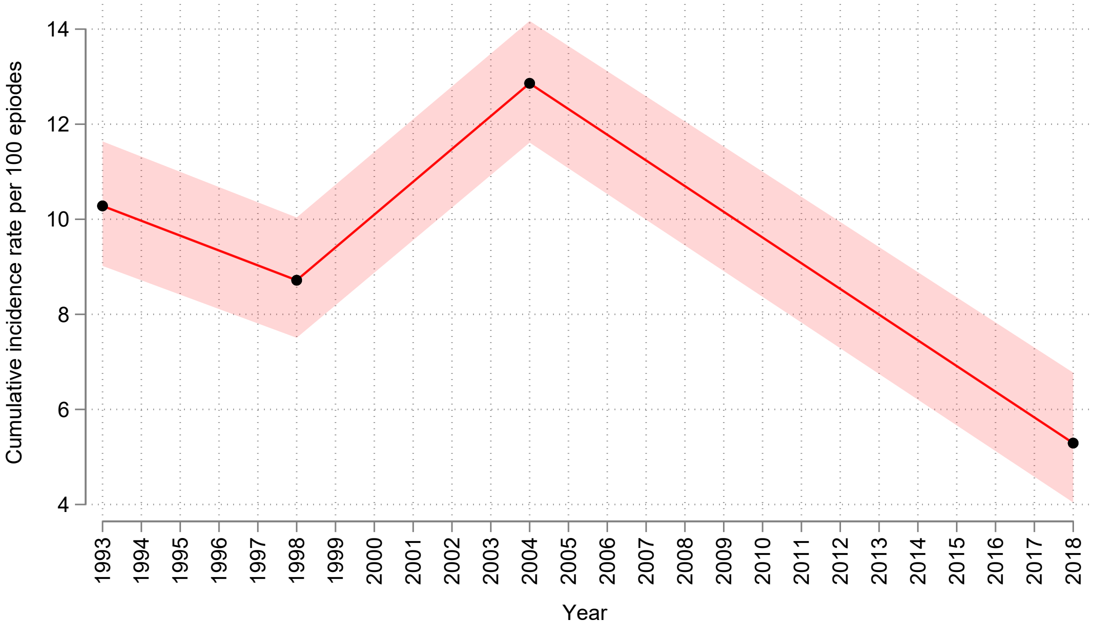

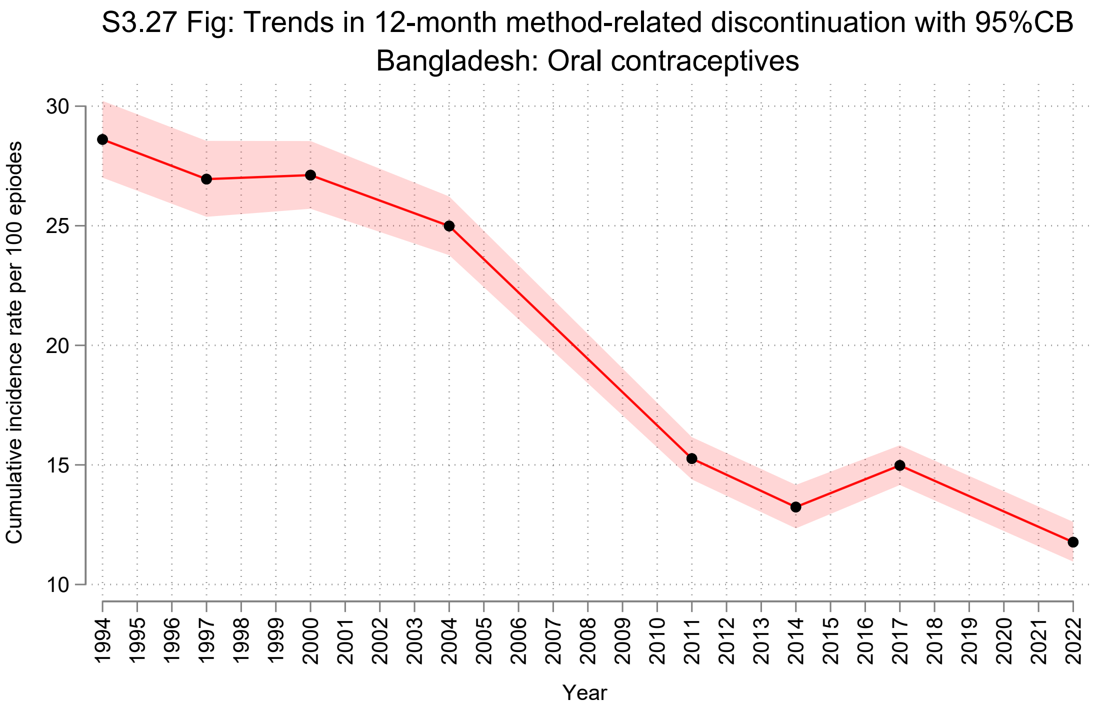
S3.27 Fig: Trends in 12-month method-related discontinuation with 95%CB
Bangladesh: Oral contraceptives
Cumulative incidence rate per 100 episodes
30
25
20
15
10
1994
1995
1996
1997
1998
1999
2000
2001
2002
2003
2004
2005
2006
2007
2008
2009
2010
2011
2012
2013
2014
2015
2016
2017
2018
2019
2020
2021
2022
Year

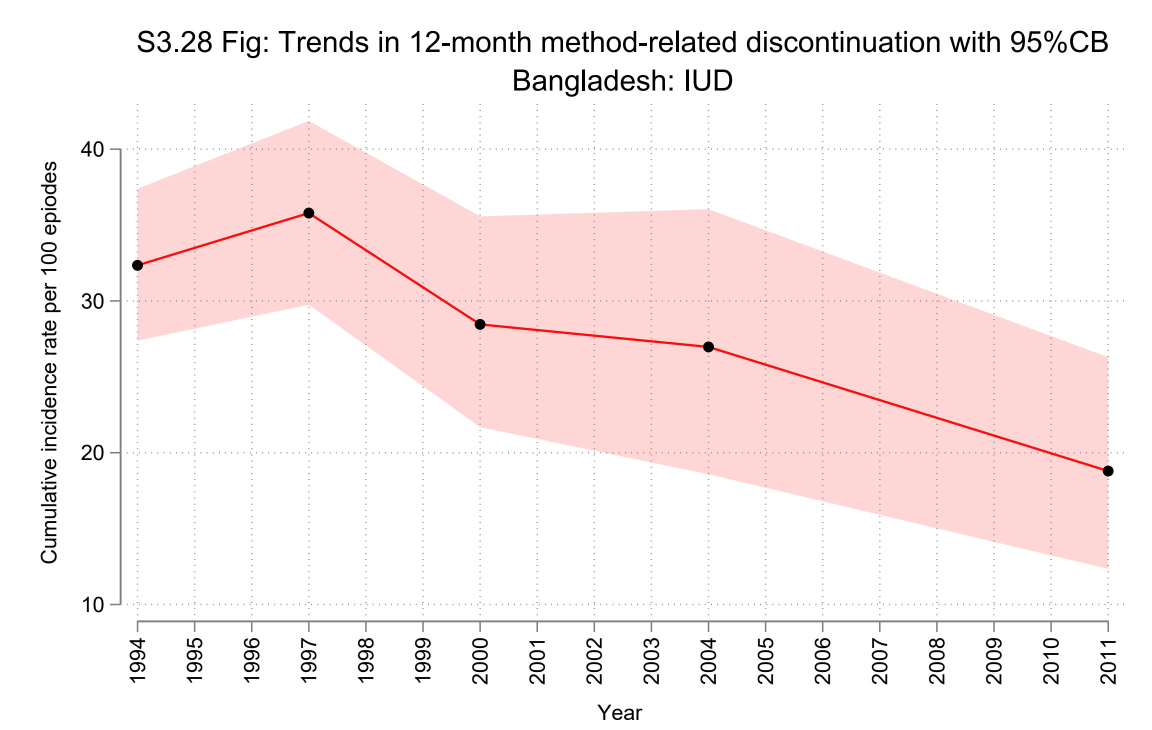
S3.28 Fig: Trends in 12-month method-related discontinuation with 95%CB
Bangladesh: IUD
Cumulative incidence rate per 100 epiodes
10
20
30
40
1994
1995
1996
1997
1998
1999
2000
2001
2002
2003
2004
2005
2006
2007
2008
2009
2010
2011
Year

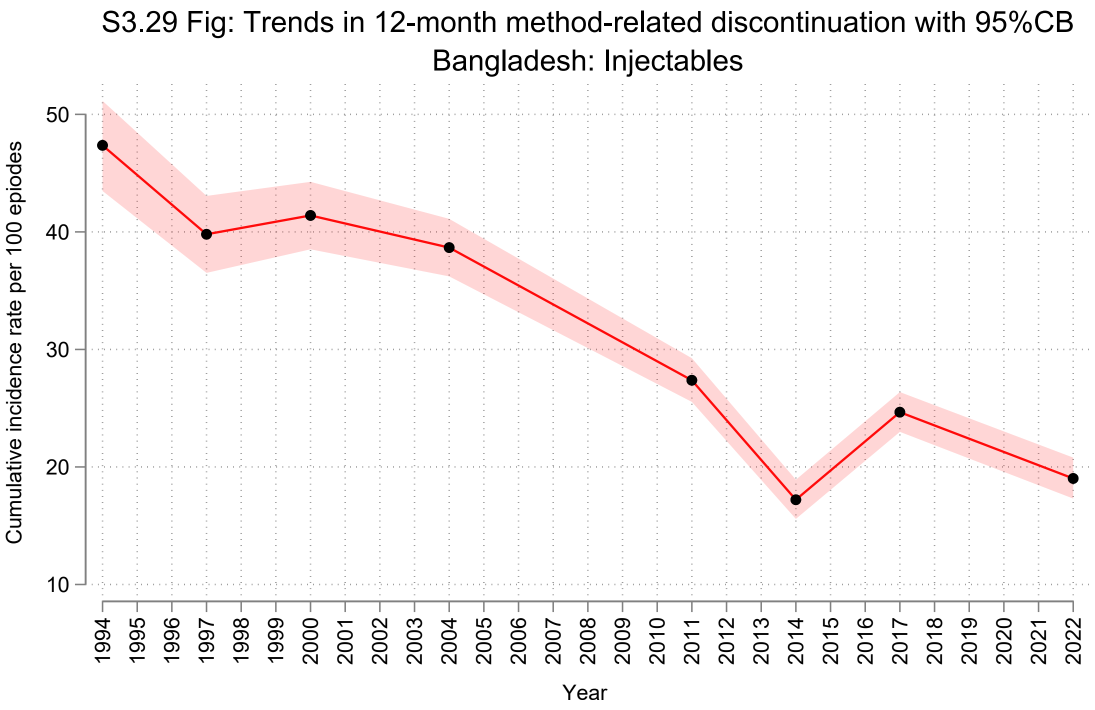
S3.29 Fig: Trends in 12-month method-related discontinuation with 95%CB
Bangladesh: Injectables
Cumulative incidence rate per 100 episodes
10
20
30
40
50
1994
1995
1996
1997
1998
1999
2000
2001
2002
2003
2004
2005
2006
2007
2008
2009
2010
2011
2012
2013
2014
2015
2016
2017
2018
2019
2020
2021
2022
Year

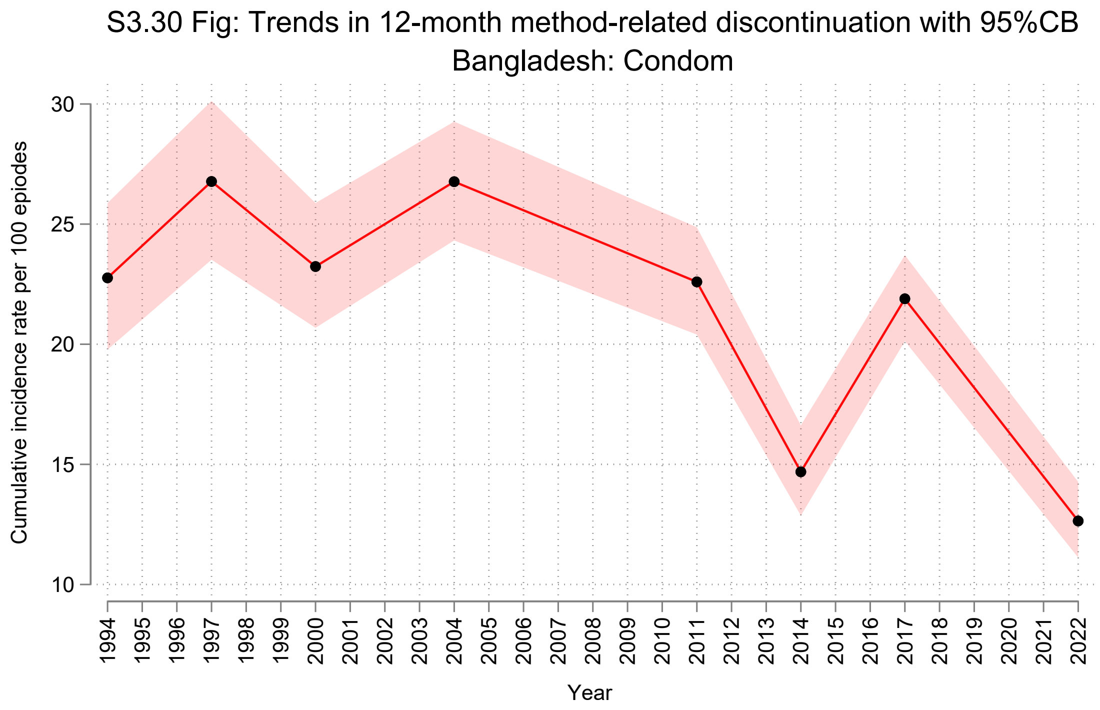
S3.30 Fig: Trends in 12-month method-related discontinuation with 95%CB
Bangladesh: Condom
Cumulative incidence rate per 100 episodes
30
25
20
15
10
1994
1995
1996
1997
1998
1999
2000
2001
2002
2003
2004
2005
2006
2007
2008
2009
2010
2011
2012
2013
2014
2015
2016
2017
2018
2019
2020
2021
2022
Year

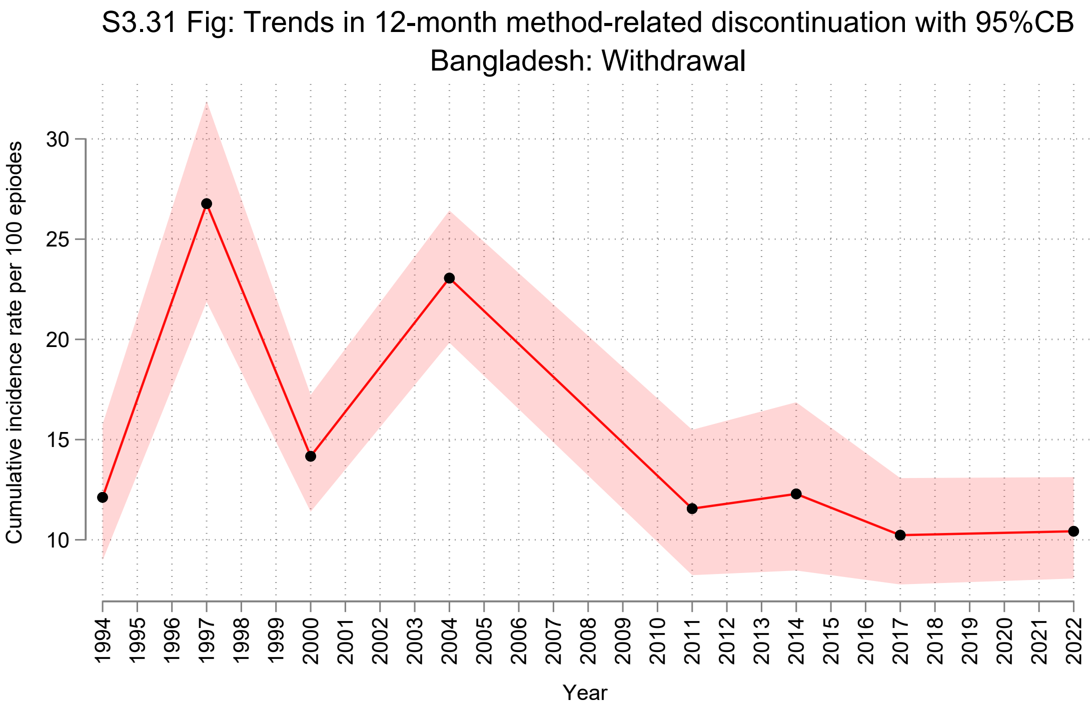
S3.31 Fig: Trends in 12-month method-related discontinuation with 95%CB
Bangladesh: Withdrawal
Cumulative incidence rate per 100 epiodes
30
25
20
15
10
1994
1995
1996
1997
1998
1999
2000
2001
2002
2003
2004
2005
2006
2007
2008
2009
2010
2011
2012
2013
2014
2015
2016
2017
2018
2019
2020
2021
2022
Year

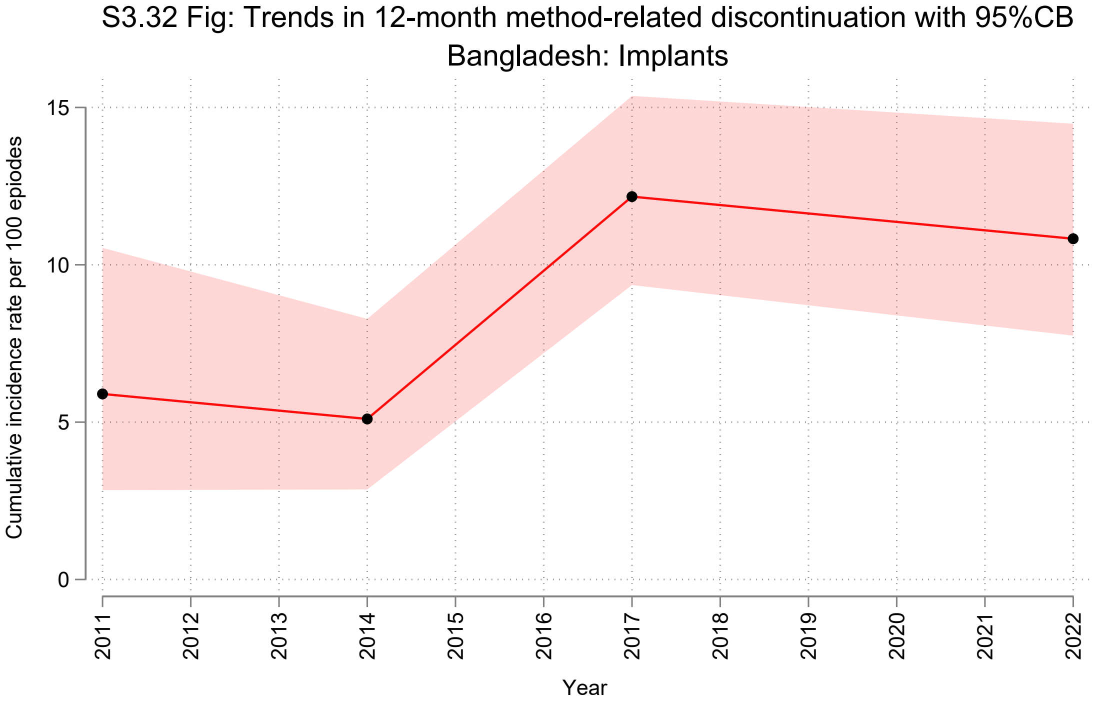
S3.32 Fig: Trends in 12-month method-related discontinuation with 95%CB
Bangladesh: Implants
Cumulative incidence rate per 100 epiodes
15
10
5
0
2011
2012
2013
2014
2015
2016
2017
2018
2019
2020
2021
2022
Year

S3.33 Fig: Trends in 12-month method-related discontinuation with 95%CB
Cambodia: Oral contraceptives

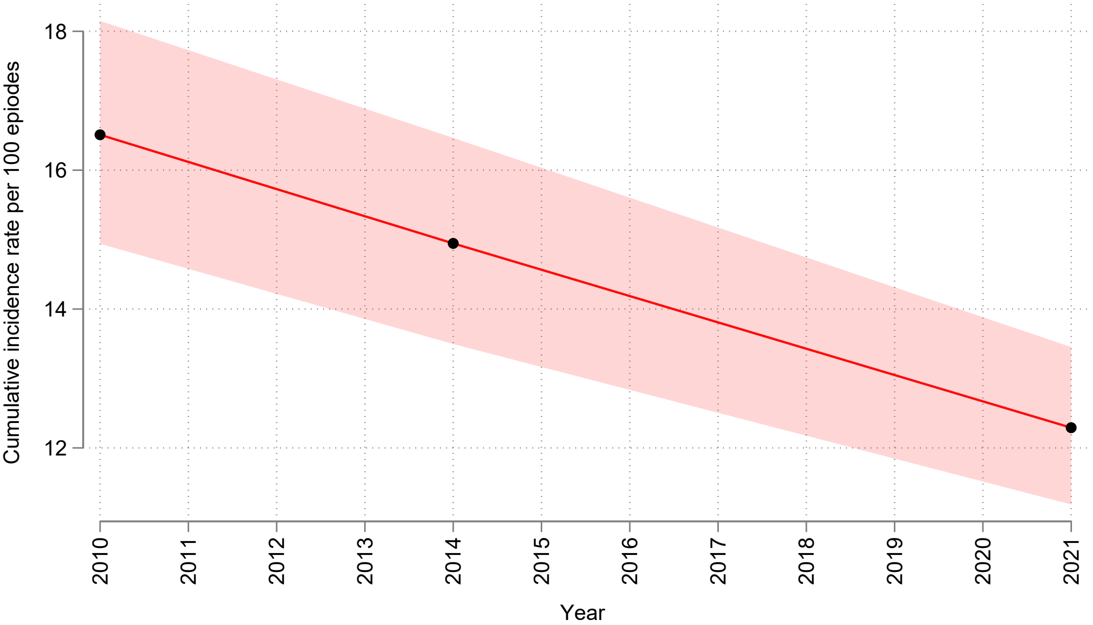

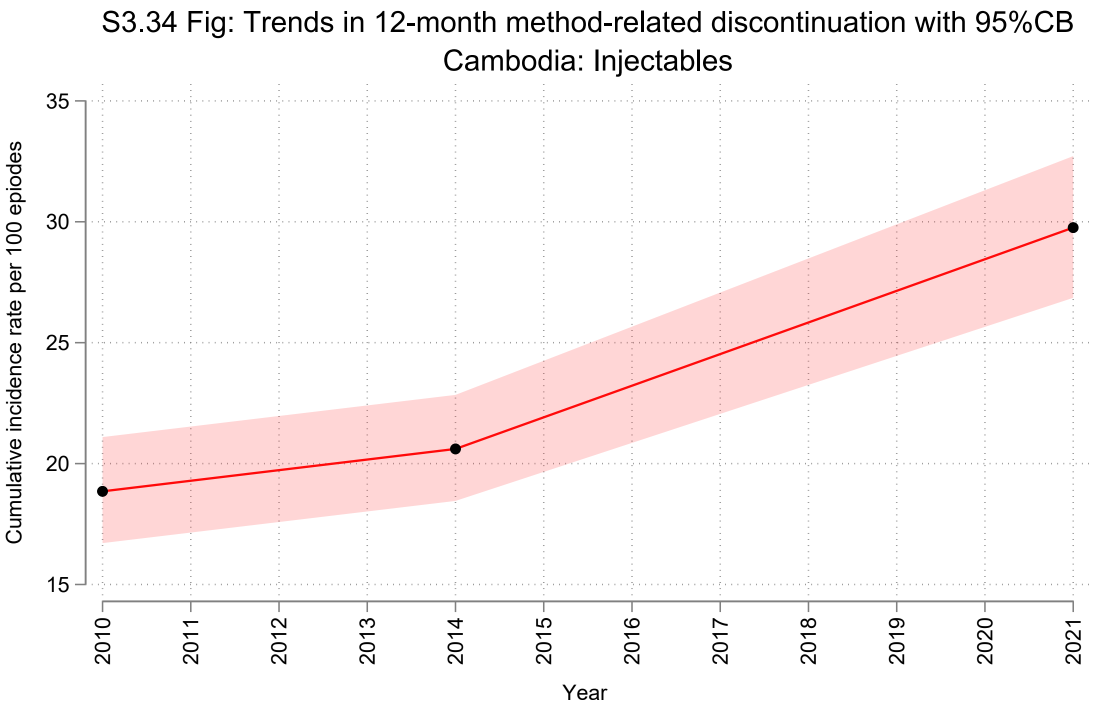
S3.34 Fig: Trends in 12-month method-related discontinuation with 95%CB
Cambodia: Injectables
Cumulative incidence rate per 100 epiodes
35
30
25
20
15
2010
2011
2012
2013
2014
2015
2016
2017
2018
2019
2020
2021
Year

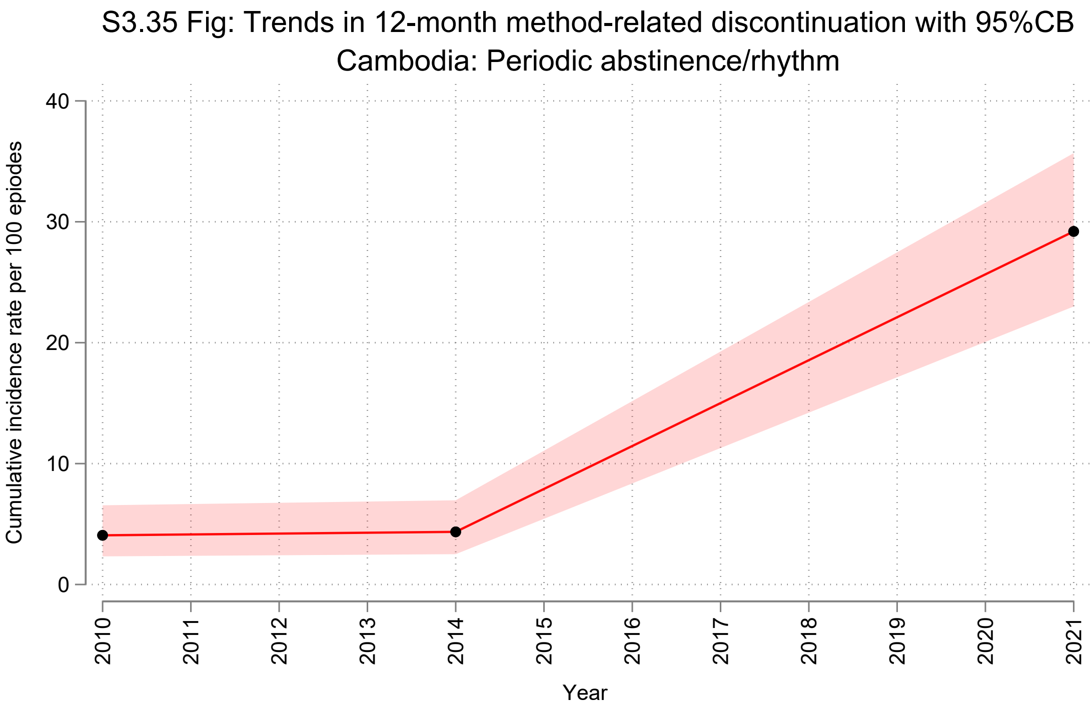

S3.35 Fig: Trends in 12-month method-related discontinuation with 95%CB
Cambodia: Periodic abstinence/rhythm
Cumulative incidence rate per 100 epiodes
0
10
20
30
40
2010
2011
2012
2013
2014
2015
2016
2017
2018
2019
2020
2021
Year

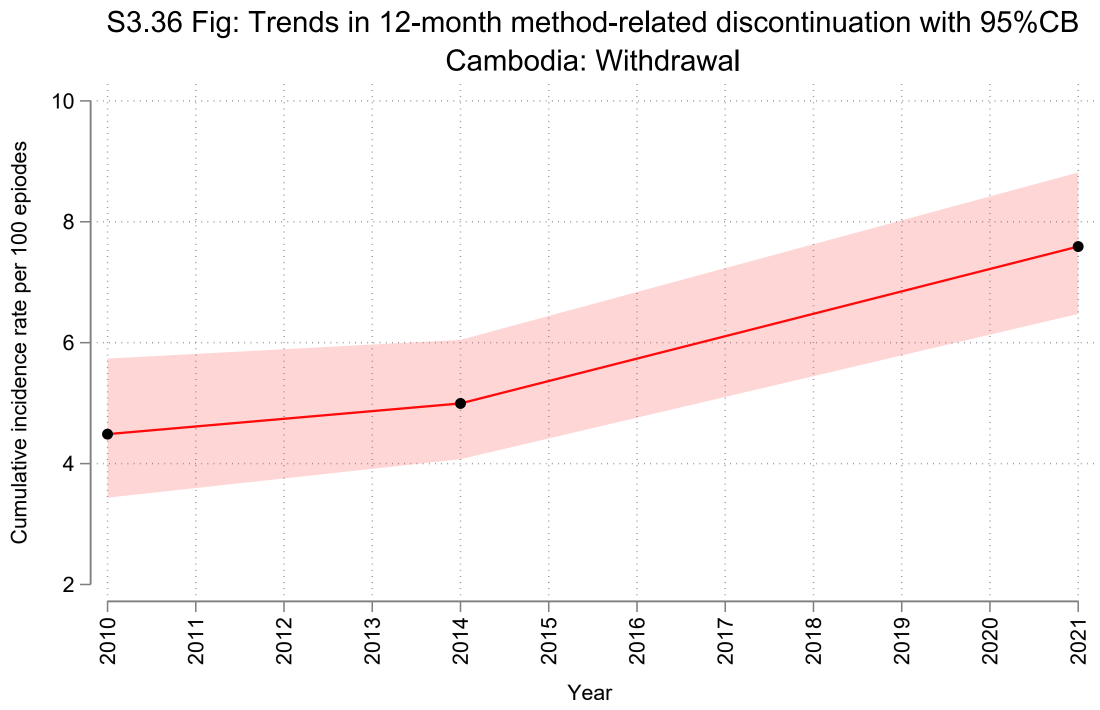

S3.36 Fig: Trends in 12-month method-related discontinuation with 95%CB
Cambodia: Withdrawal
Cumulative incidence rate per 100 epiodes
10
8
6
4
2
2010
2011
2012
2013
2014
2015
2016
2017
2018
2019
2020
2021
Year

S3.37 Fig: Trends in 12-month method-related discontinuation with 95%CB
India: IUD

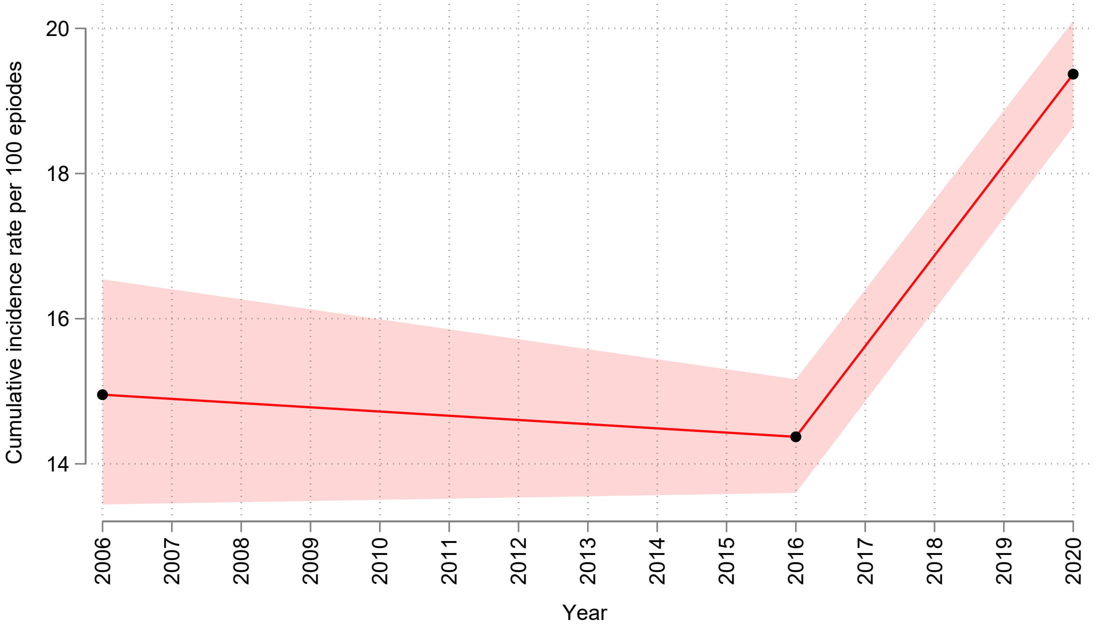

S3.38 Fig: Trends in 12-month method-related discontinuation with 95%CB
India: Condom

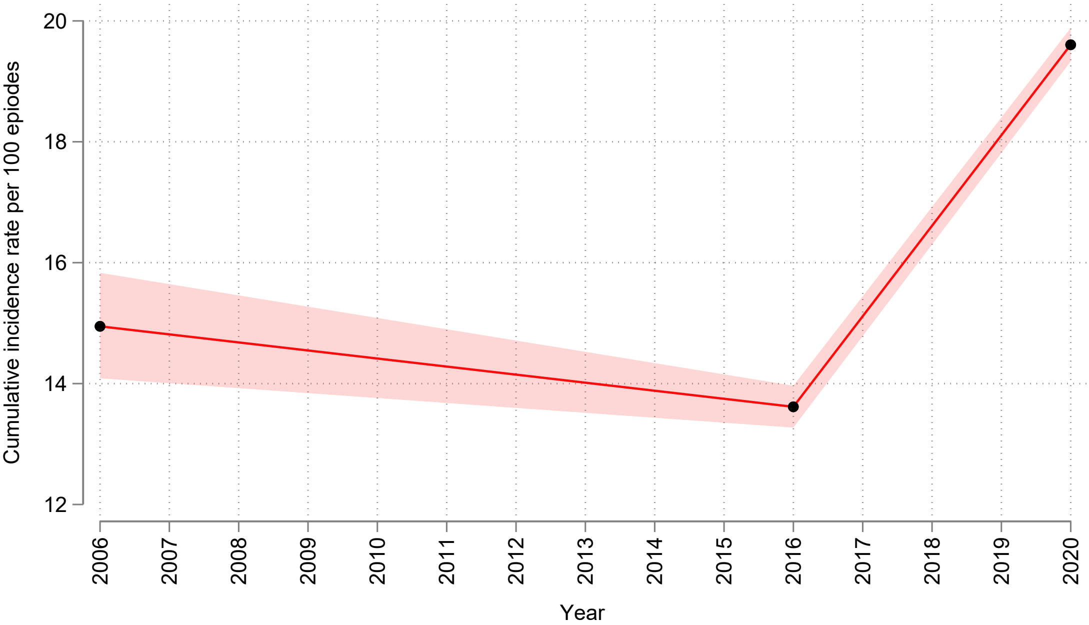

S3.39 Fig: Trends in 12-month method-related discontinuation with 95%CB
India: Periodic abstinence/rhythm

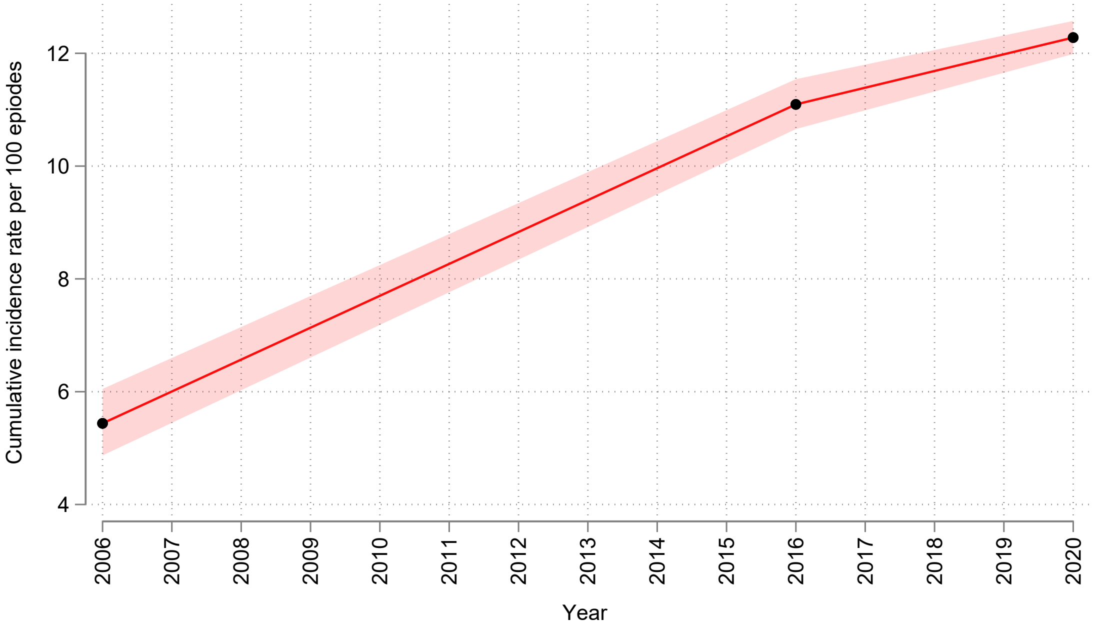

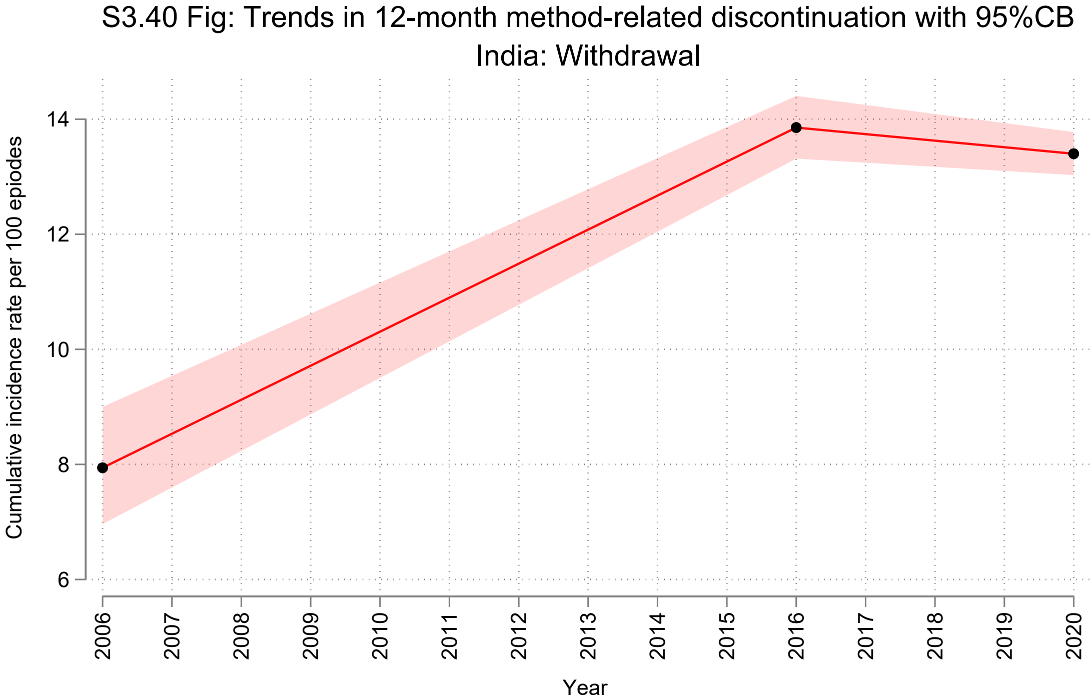
S3.40 Fig: Trends in 12-month method-related discontinuation with 95%CB
India: Withdrawal
Cumulative incidence rate per 100 epiodes
14
12
10
8
6
2006
2007
2008
2009
2010
2011
2012
2013
2014
2015
2016
2017
2018
2019
2020
Year

S3.41 Fig: Trends in 12-month method-related discontinuation with 95%CB
Indonesia: Oral contraceptives

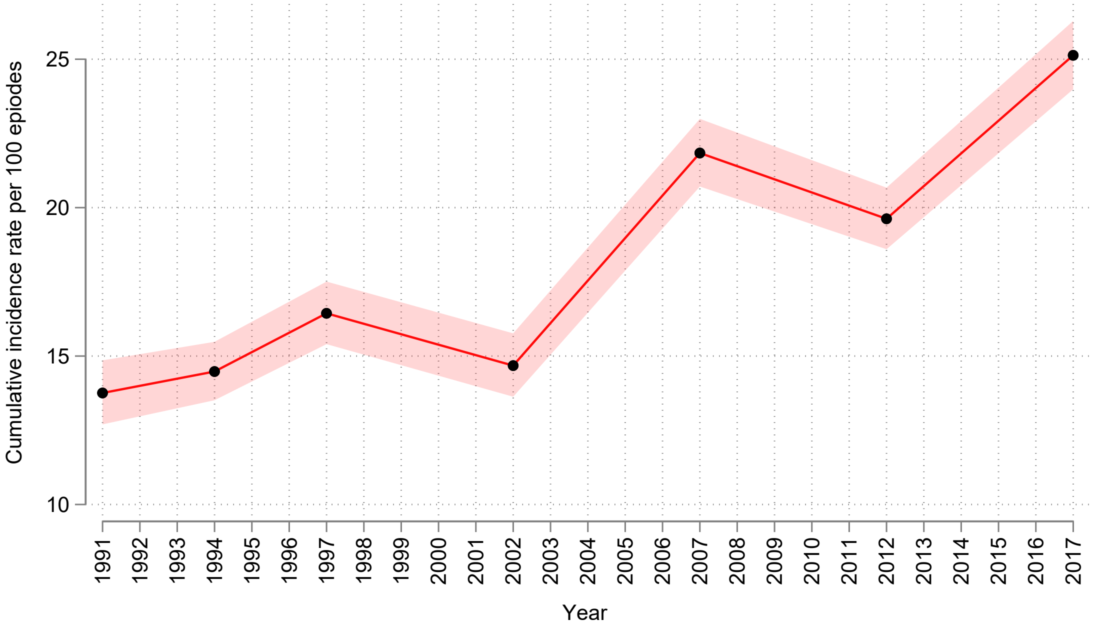

S3.42 Fig: Trends in 12-month method-related discontinuation with 95%CB
Indonesia: IUD

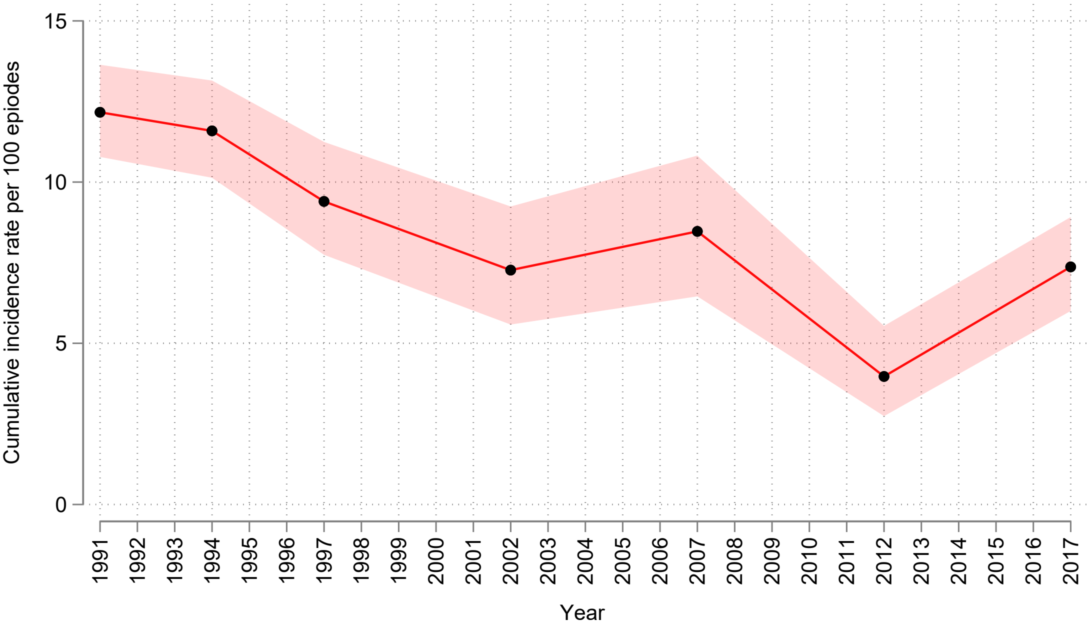

S3.43 Fig: Trends in 12-month method-related discontinuation with 95%CB
Indonesia: Condom

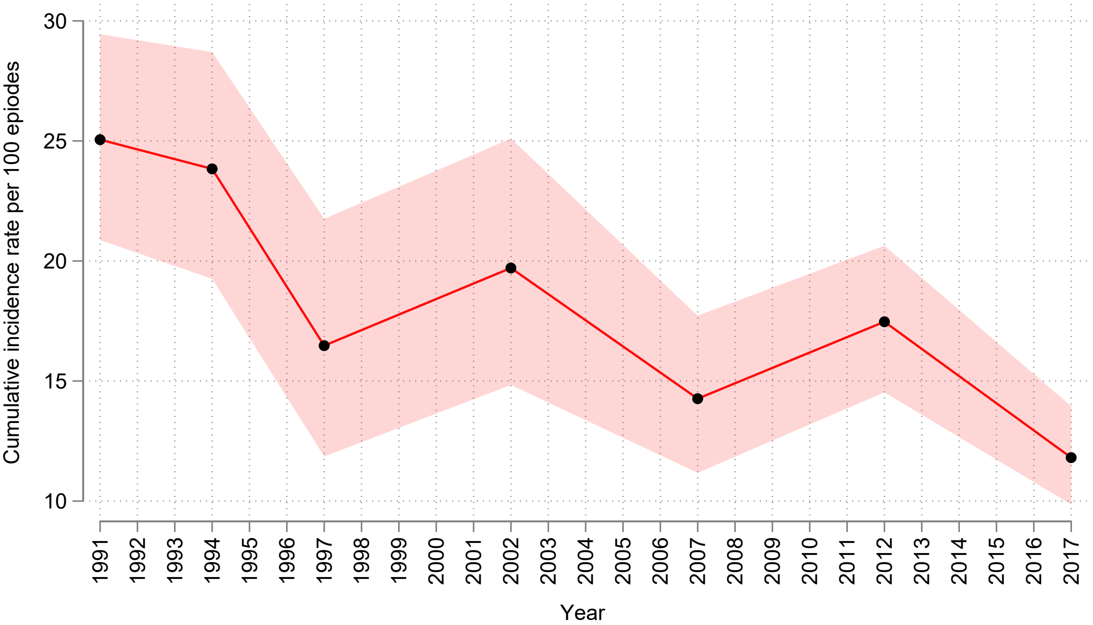

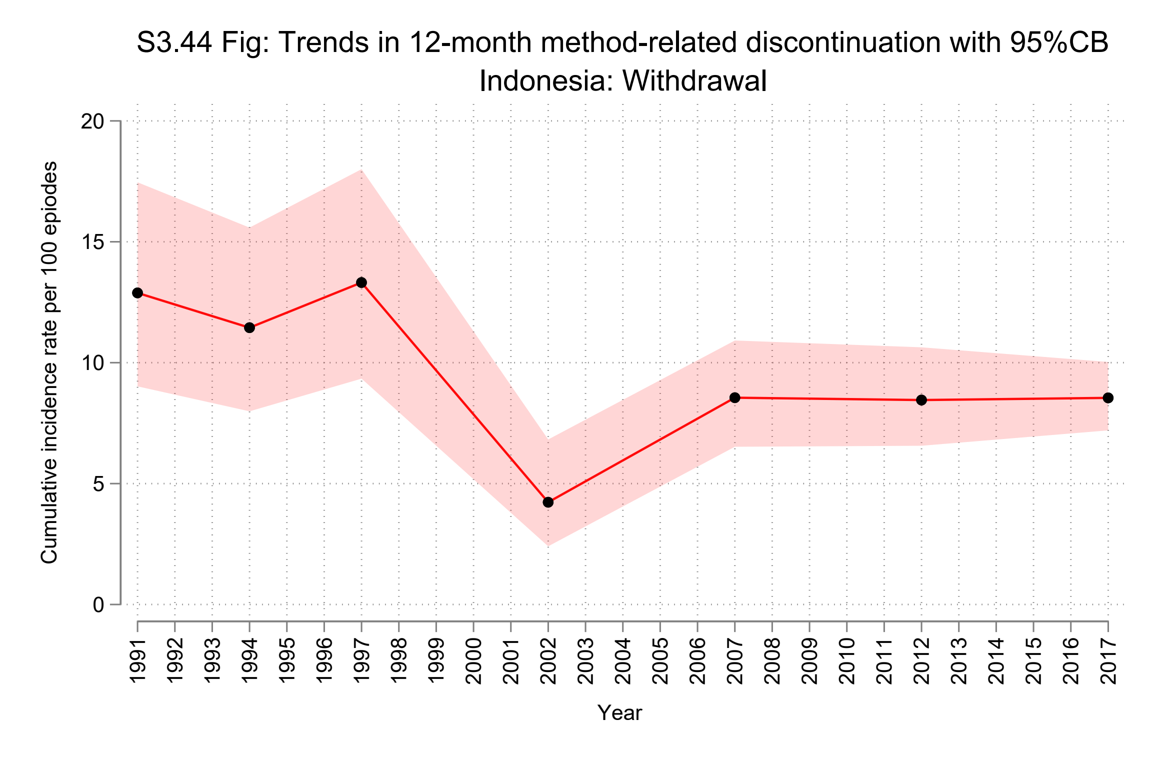
S3.44 Fig: Trends in 12-month method-related discontinuation with 95%CB
Indonesia: Withdrawal
Cumulative incidence rate per 100 epiodes
0
5
10
15
20
1991
1992
1993
1994
1995
1996
1997
1998
1999
2000
2001
2002
2003
2004
2005
2006
2007
2008
2009
2010
2011
2012
2013
2014
2015
2016
2017
Year

S3.45 Fig: Trends in 12-month method-related discontinuation with 95%CB
Indonesia: Implants

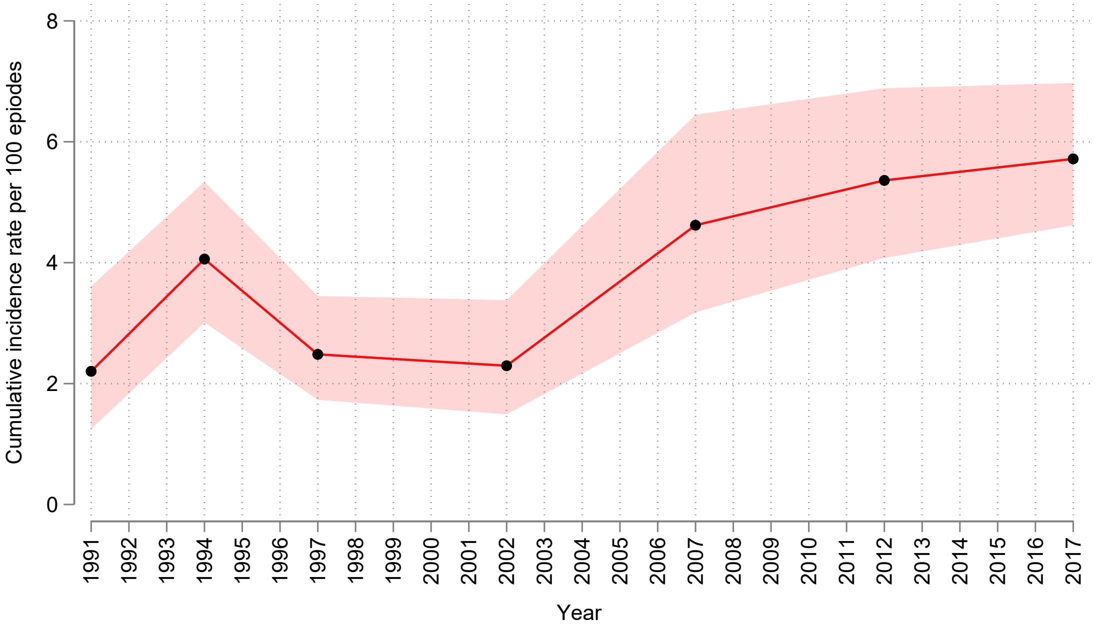

S3.46 Fig: Trends in 12-month method-related discontinuation with 95%CB
Nepal: Injectables

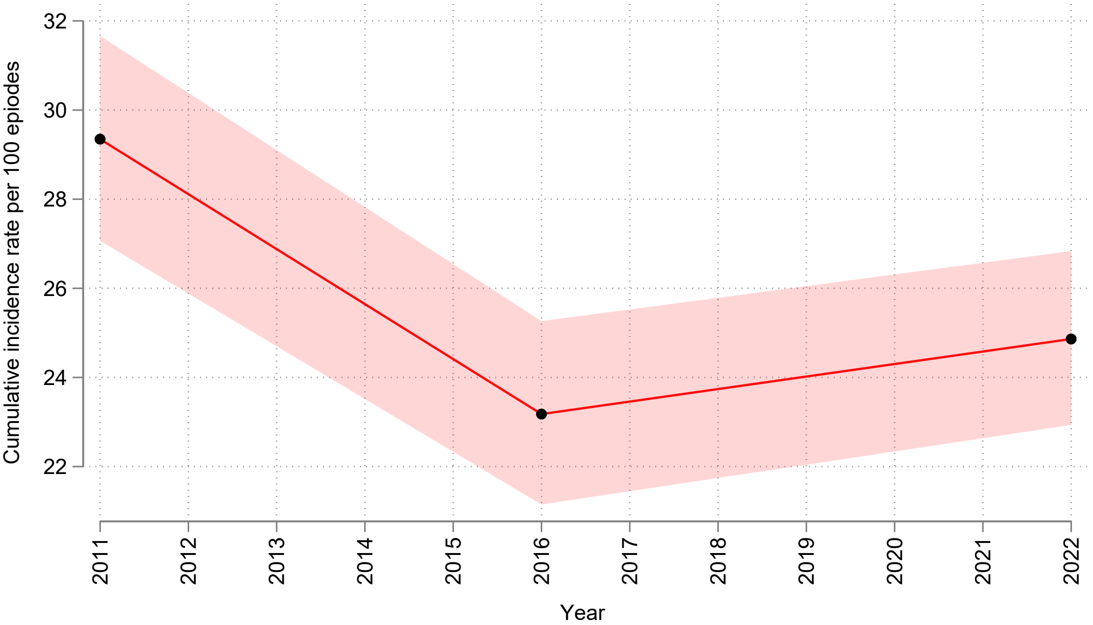

S3.47 Fig: Trends in 12-month method-related discontinuation with 95%CB
Philippines: Condom

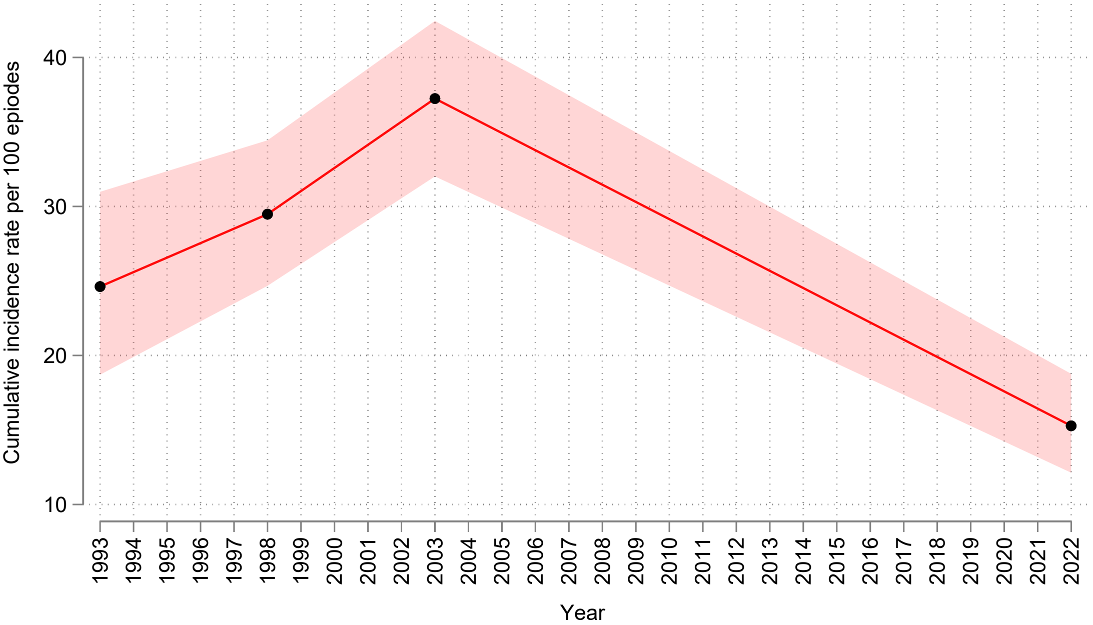

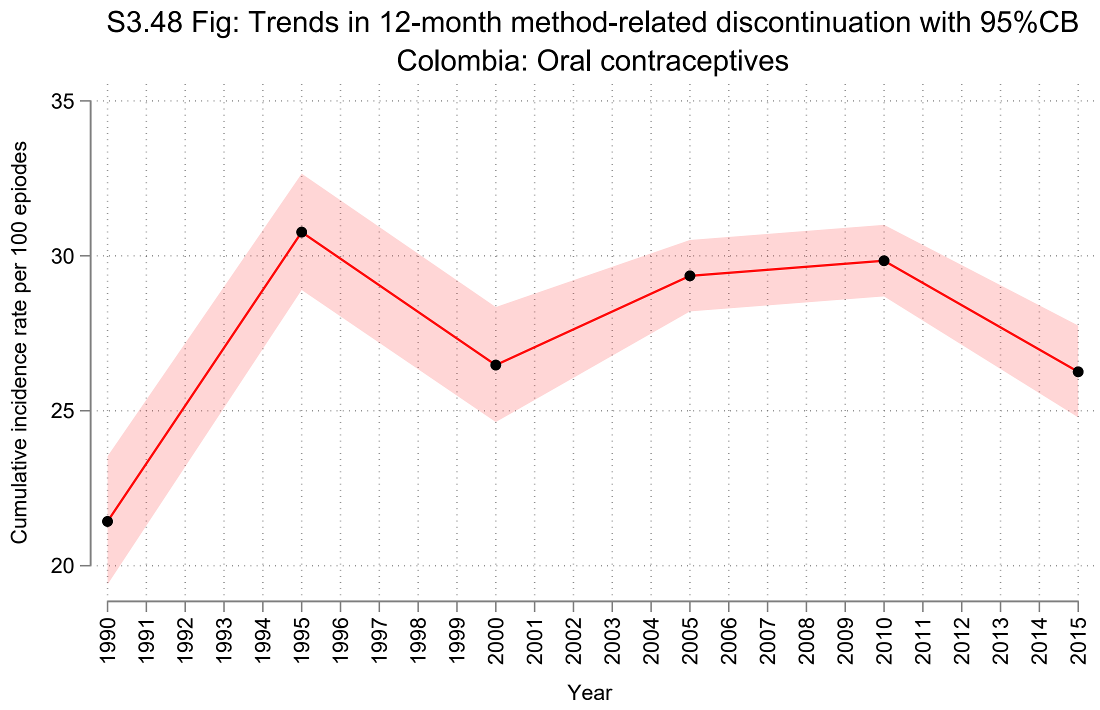
S3.48 Fig: Trends in 12-month method-related discontinuation with 95%CB
Colombia: Oral contraceptives
Cumulative incidence rate per 100 epiodes
35
30
25
20
1990
1991
1992
1993
1994
1995
1996
1997
1998
1999
2000
2001
2002
2003
2004
2005
2006
2007
2008
2009
2010
2011
2012
2013
2014
2015
Year

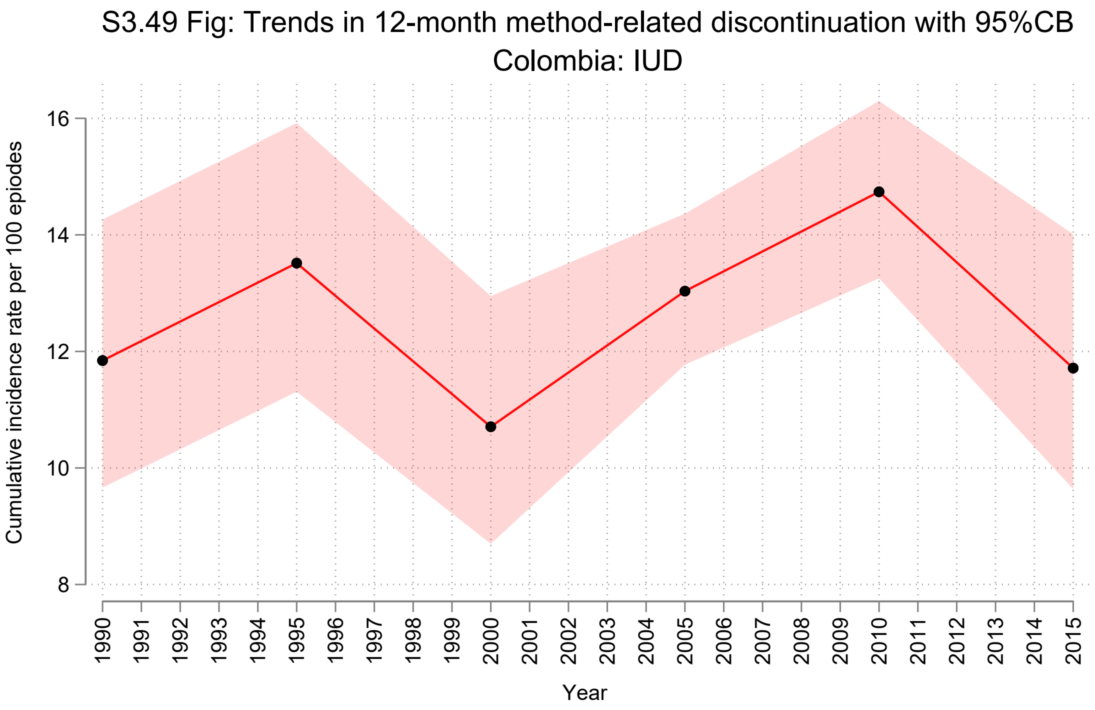
S3.49 Fig: Trends in 12-month method-related discontinuation with 95%CB
Colombia: IUD
Cumulative incidence rate per 100 episodes
16
14
12
10
8
1990
1991
1992
1993
1994
1995
1996
1997
1998
1999
2000
2001
2002
2003
2004
2005
2006
2007
2008
2009
2010
2011
2012
2013
2014
2015
Year

S3.50 Fig: Trends in 12-month method-related discontinuation with 95%CB
Colombia: Injectables

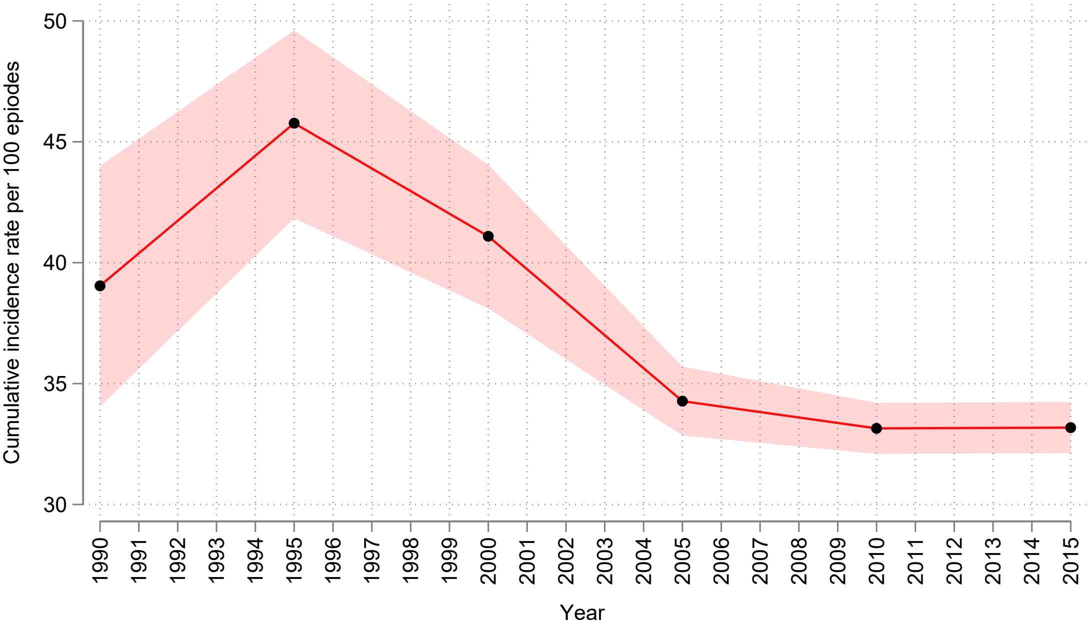

S3.51 Fig: Trends in 12-month method-related discontinuation with 95%CB
Colombia: Condom

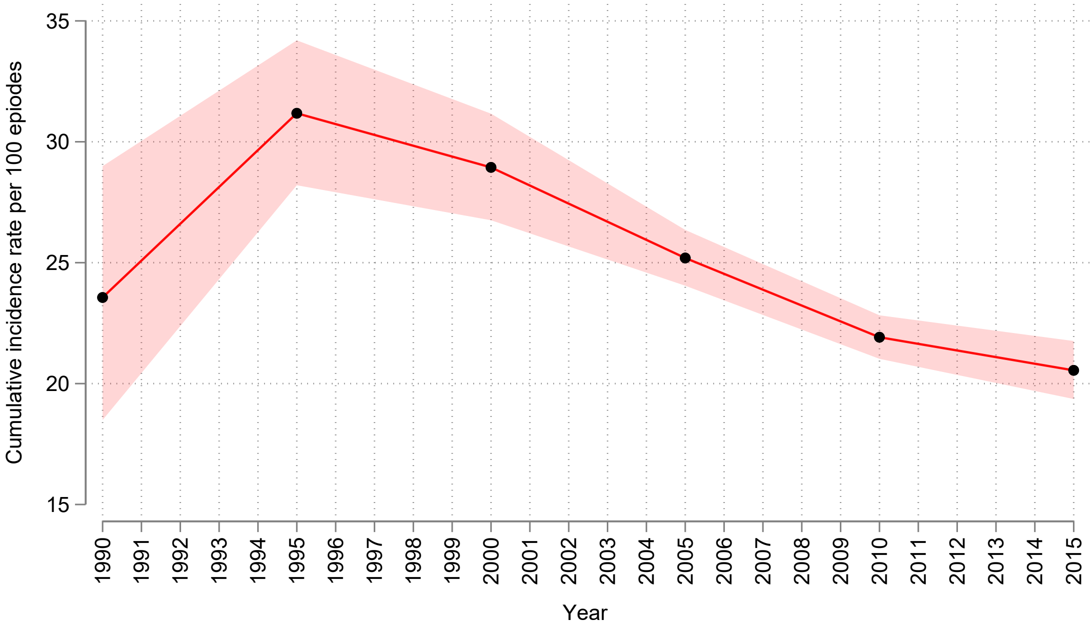

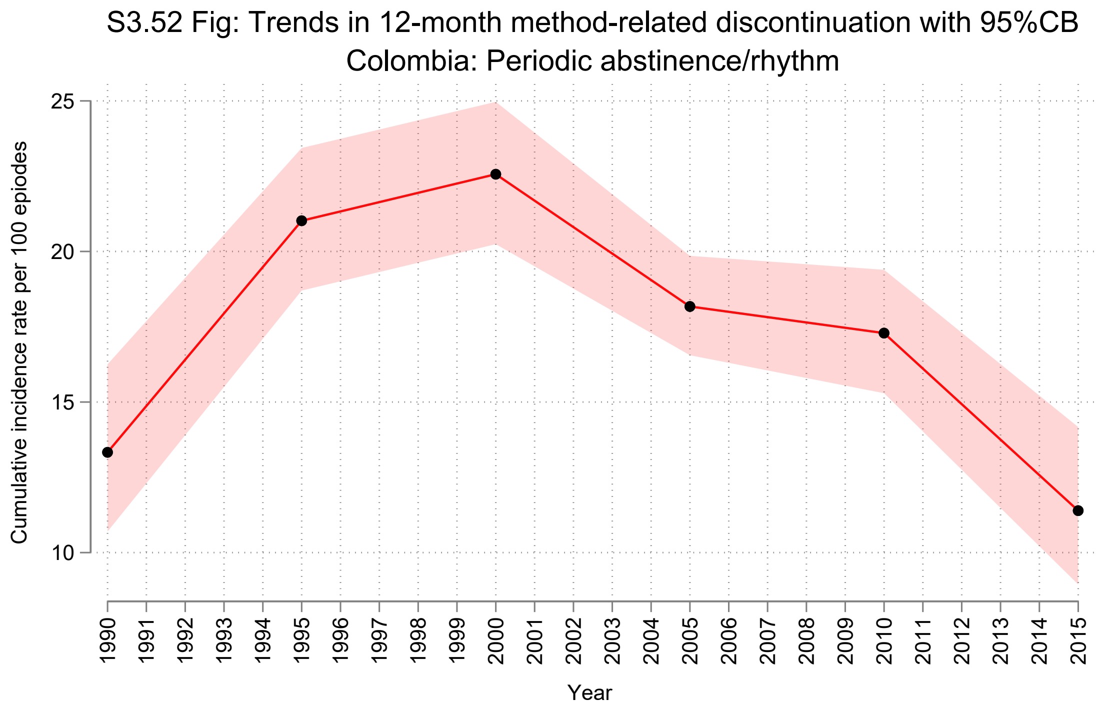
S3.52 Fig: Trends in 12-month method-related discontinuation with 95%CB
Colombia: Periodic abstinence/rhythm
Cumulative incidence rate per 100 epiodes
25
20
15
10
1990
1991
1992
1993
1994
1995
1996
1997
1998
1999
2000
2001
2002
2003
2004
2005
2006
2007
2008
2009
2010
2011
2012
2013
2014
2015
Year

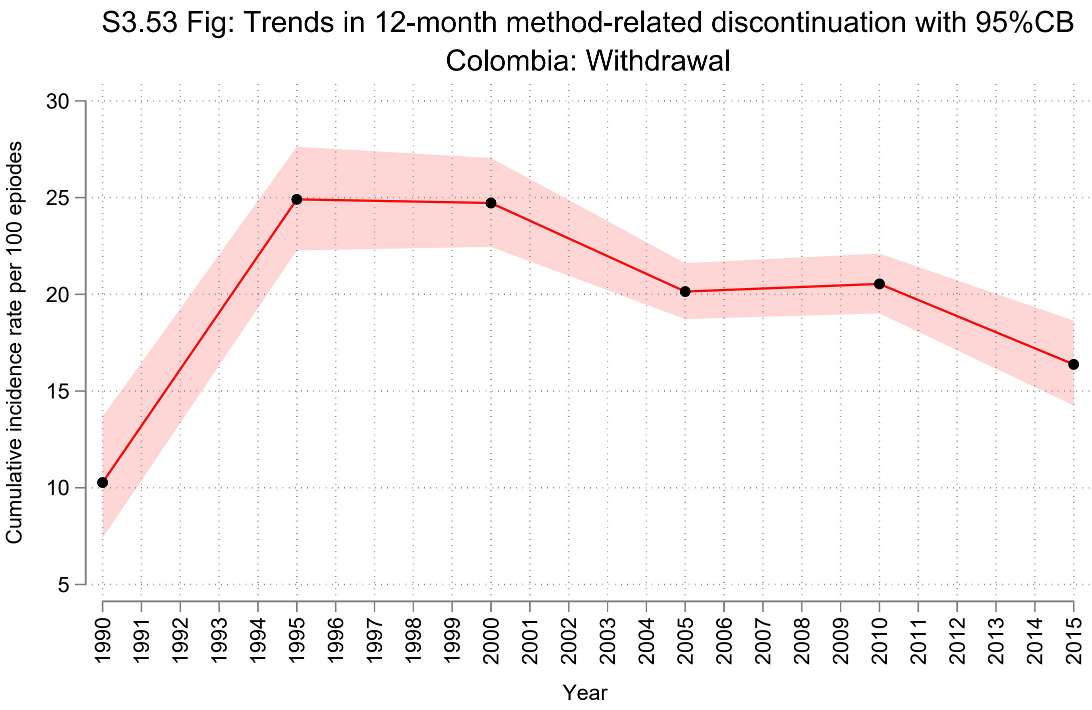

S3.53 Fig: Trends in 12-month method-related discontinuation with 95%CB
Colombia: Withdrawal
Cumulative incidence rate per 100 epiodes
30
25
20
15
10
5
1990
1991
1992
1993
1994
1995
1996
1997
1998
1999
2000
2001
2002
2003
2004
2005
2006
2007
2008
2009
2010
2011
2012
2013
2014
2015
Year

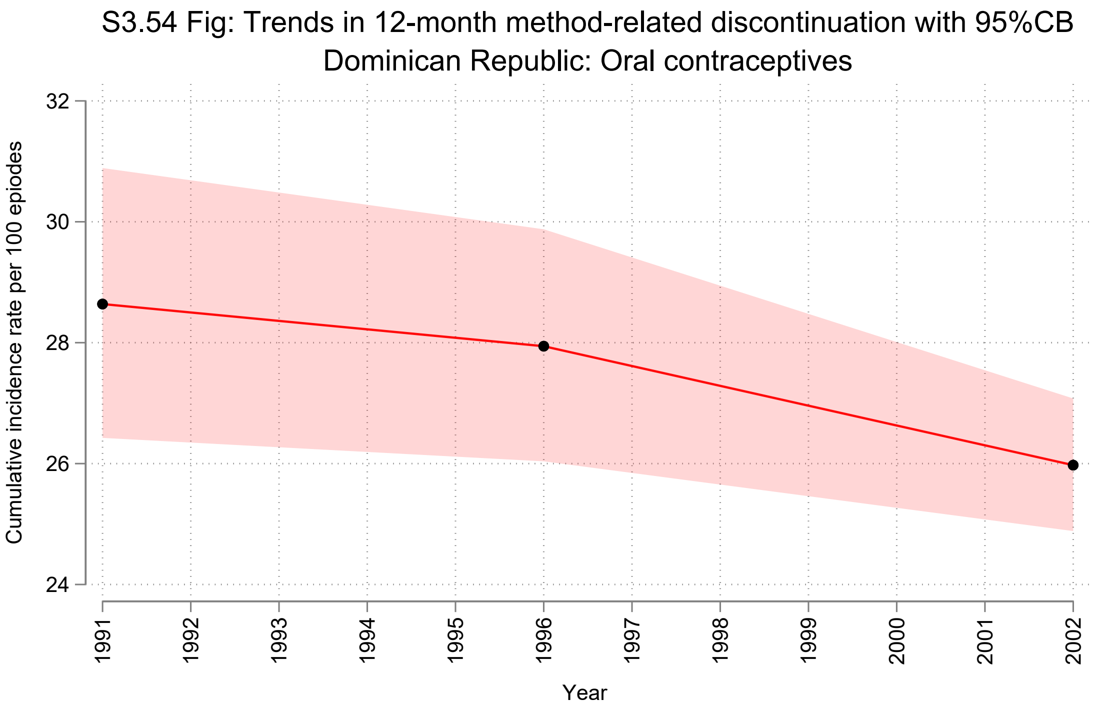
S3.54 Fig: Trends in 12-month method-related discontinuation with 95%CB
Dominican Republic: Oral contraceptives
Cumulative incidence rate per 100 epiodes
32
30
28
26
24
1991
1992
1993
1994
1995
1996
1997
1998
1999
2000
2001
2002
Year

S3.55 Fig: Trends in 12-month method-related discontinuation with 95%CB
Dominican Republic: IUD

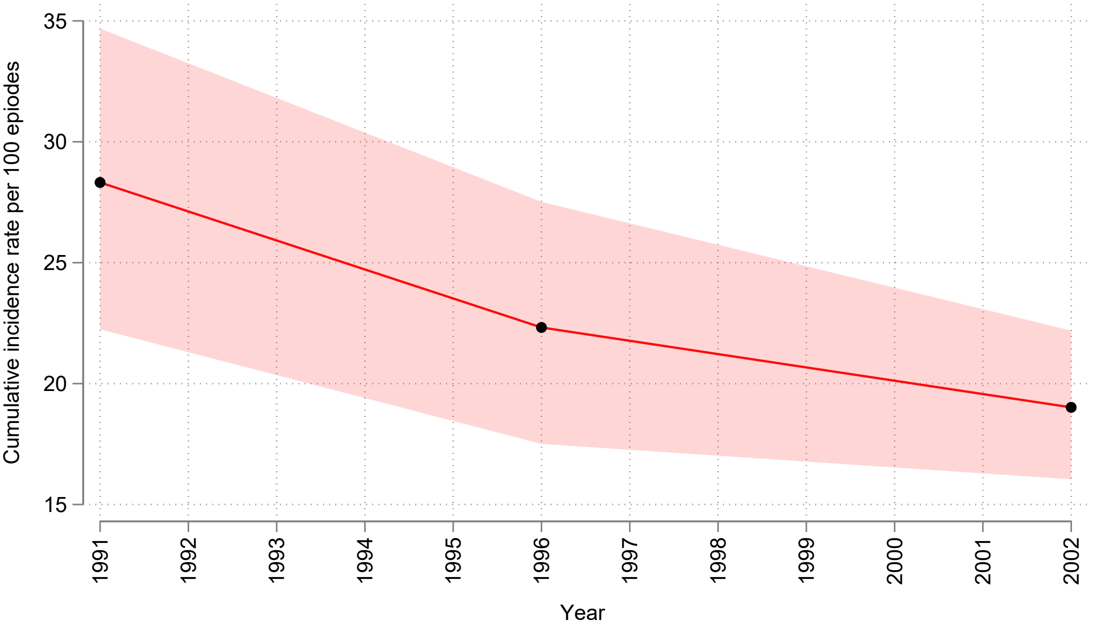

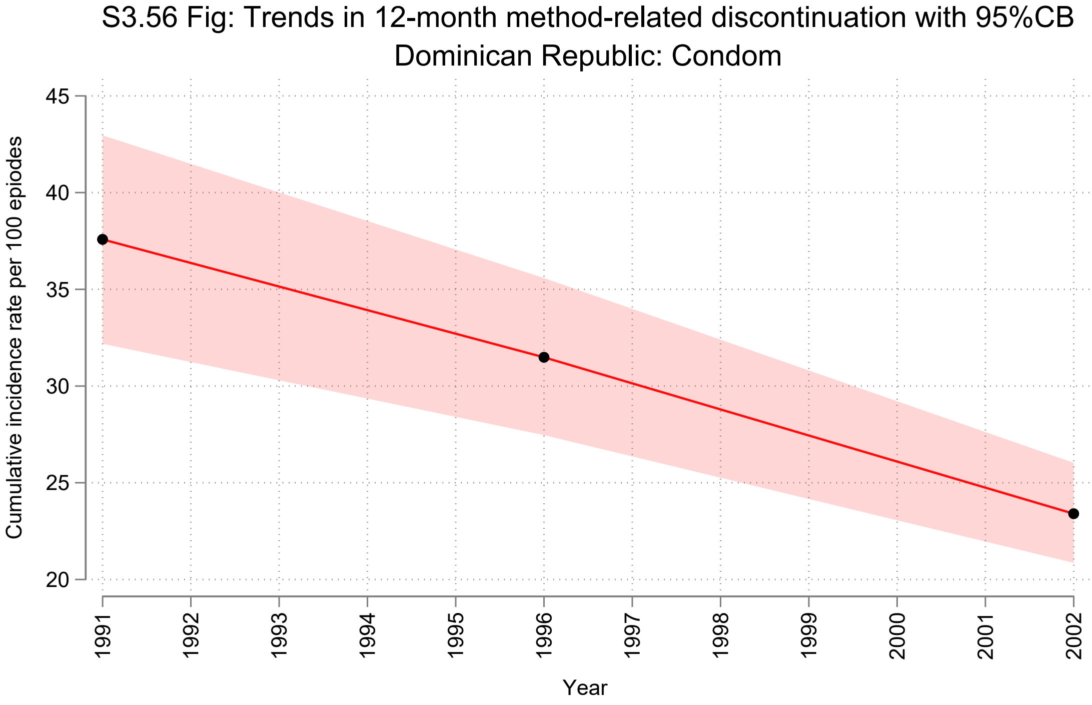
S3.56 Fig: Trends in 12-month method-related discontinuation with 95%CB
Dominican Republic: Condom
Cumulative incidence rate per 100 epiodes
45
40
35
30
25
20
1991
1992
1993
1994
1995
1996
1997
1998
1999
2000
2001
2002
Year

S3.57 Fig: Trends in 12-month method-related discontinuation with 95%CB
Dominican Republic: Withdrawal

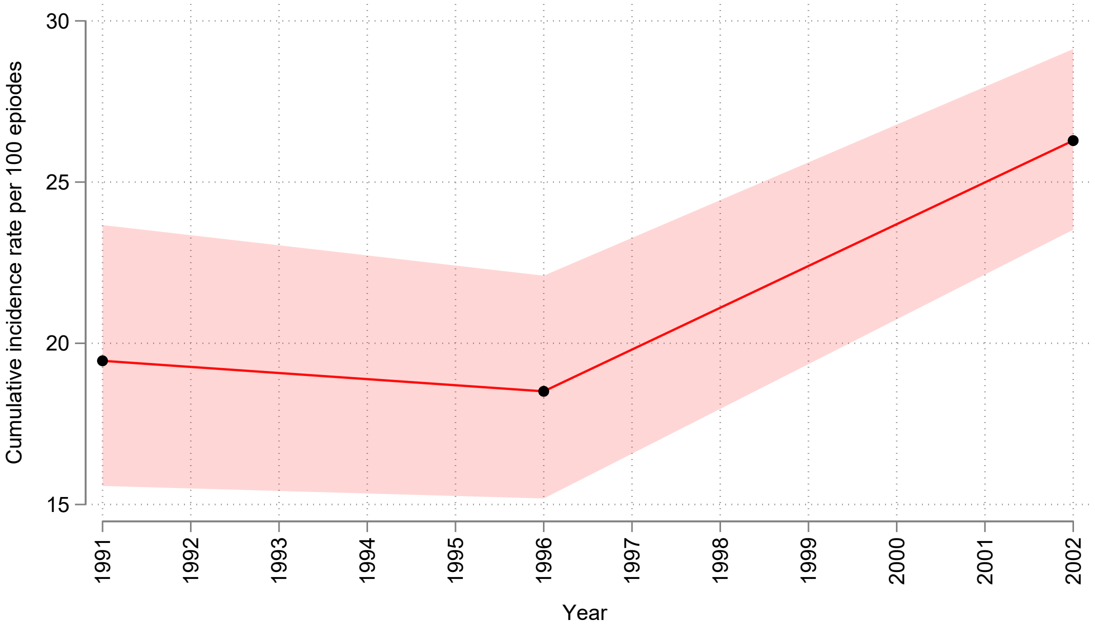

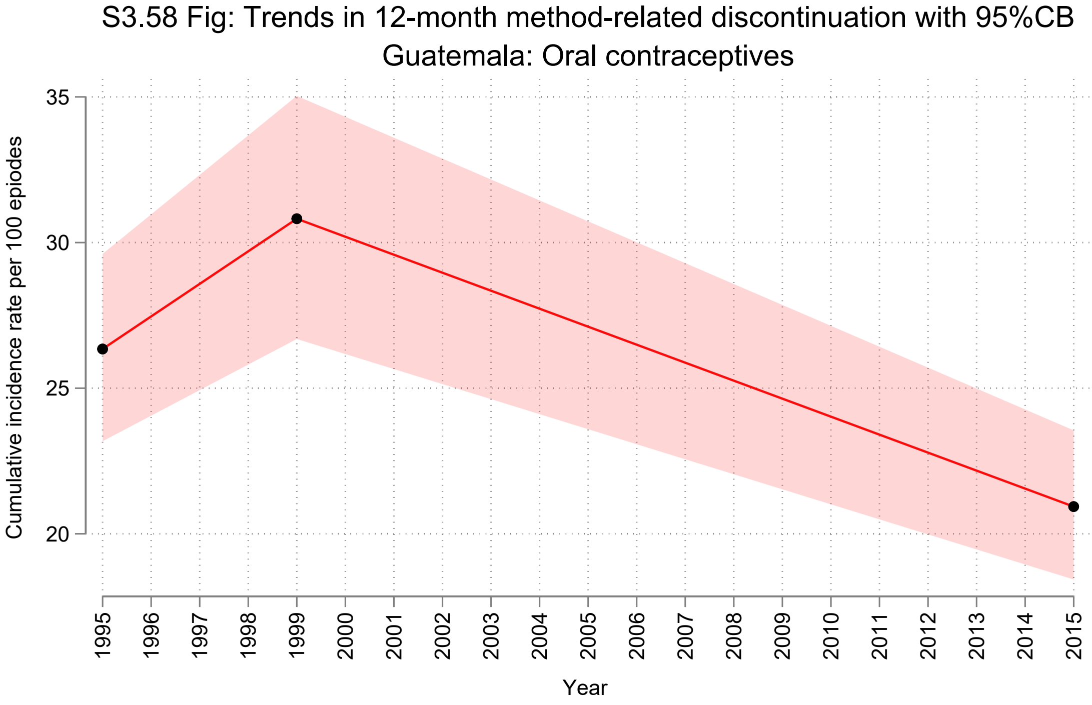
S3.58 Fig: Trends in 12-month method-related discontinuation with 95%CB
Guatemala: Oral contraceptives
Cumulative incidence rate per 100 episodes
35
30
25
20
1995
1996
1997
1998
1999
2000
2001
2002
2003
2004
2005
2006
2007
2008
2009
2010
2011
2012
2013
2014
2015
Year

S3.59 Fig: Trends in 12-month method-related discontinuation with 95%CB
Guatemala: Injectables

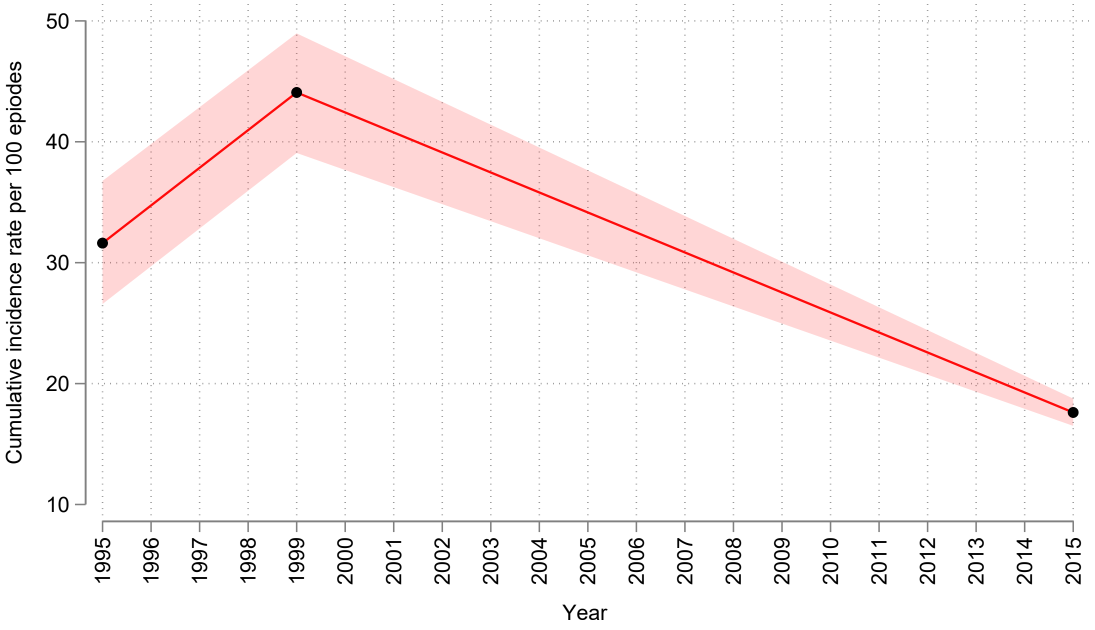

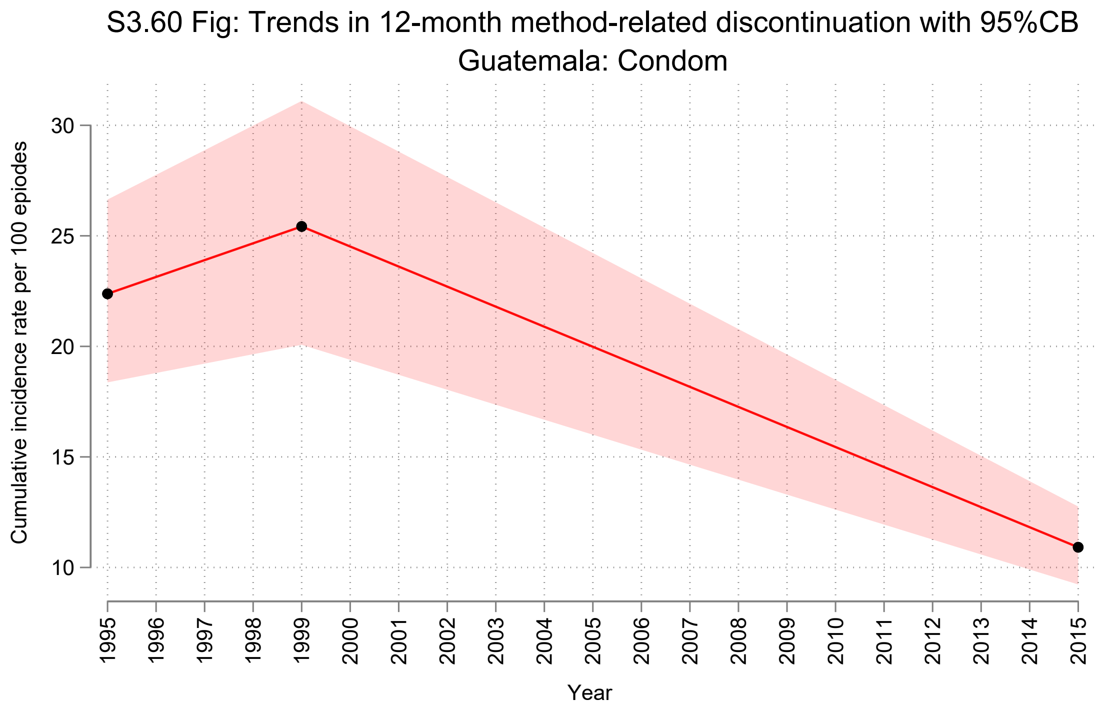
S3.60 Fig: Trends in 12-month method-related discontinuation with 95%CB
Guatemala: Condom
Cumulative incidence rate per 100 episodes
30
25
20
15
10
1995
1996
1997
1998
1999
2000
2001
2002
2003
2004
2005
2006
2007
2008
2009
2010
2011
2012
2013
2014
2015
Year

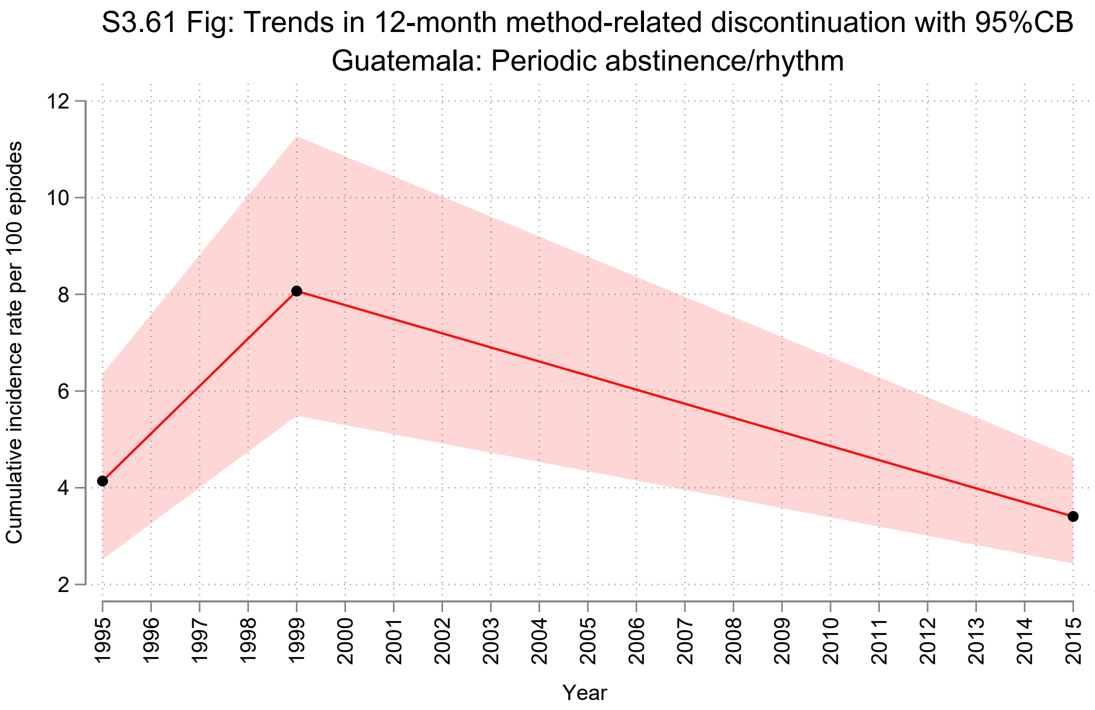

S3.61 Fig: Trends in 12-month method-related discontinuation with 95%CB
Guatemala: Periodic abstinence/rhythm
Cumulative incidence rate per 100 episodes
12
10
8
6
4
2
1995
1996
1997
1998
1999
2000
2001
2002
2003
2004
2005
2006
2007
2008
2009
2010
2011
2012
2013
2014
2015
Year

S3.62 Fig: Trends in 12-month method-related discontinuation with 95%CB
Peru: Oral contraceptives

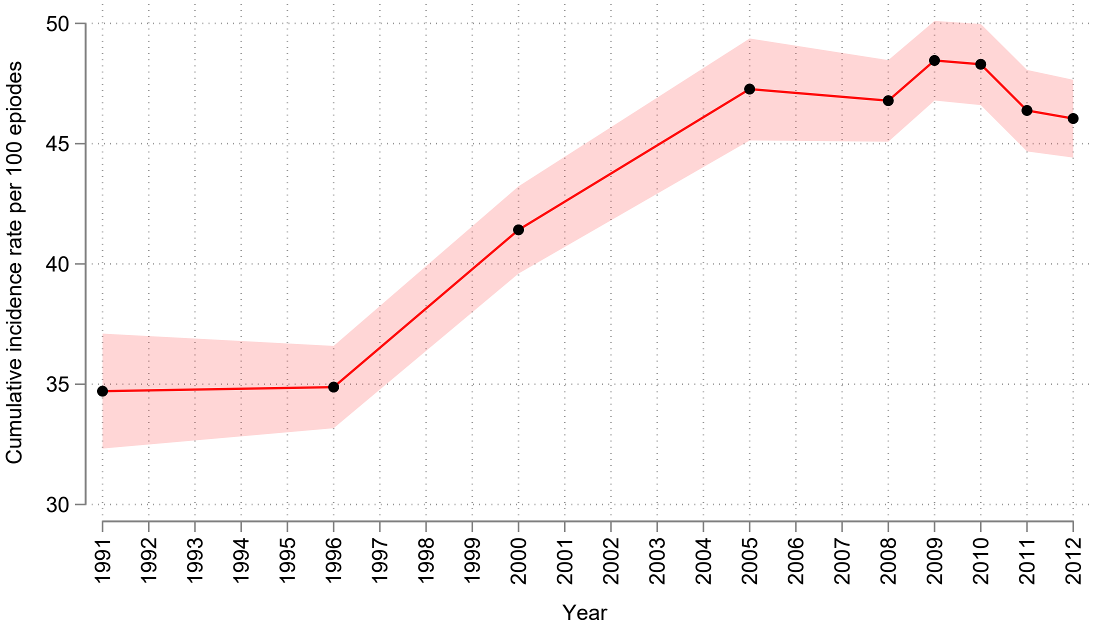

S3.63 Fig: Trends in 12-month method-related discontinuation with 95%CB
Peru: IUD

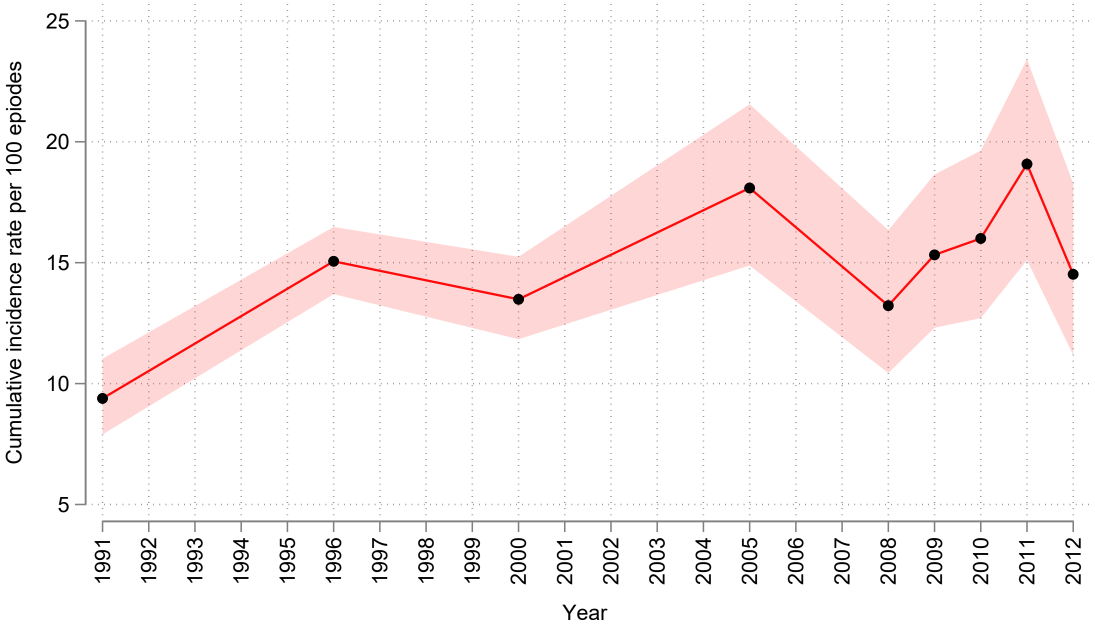

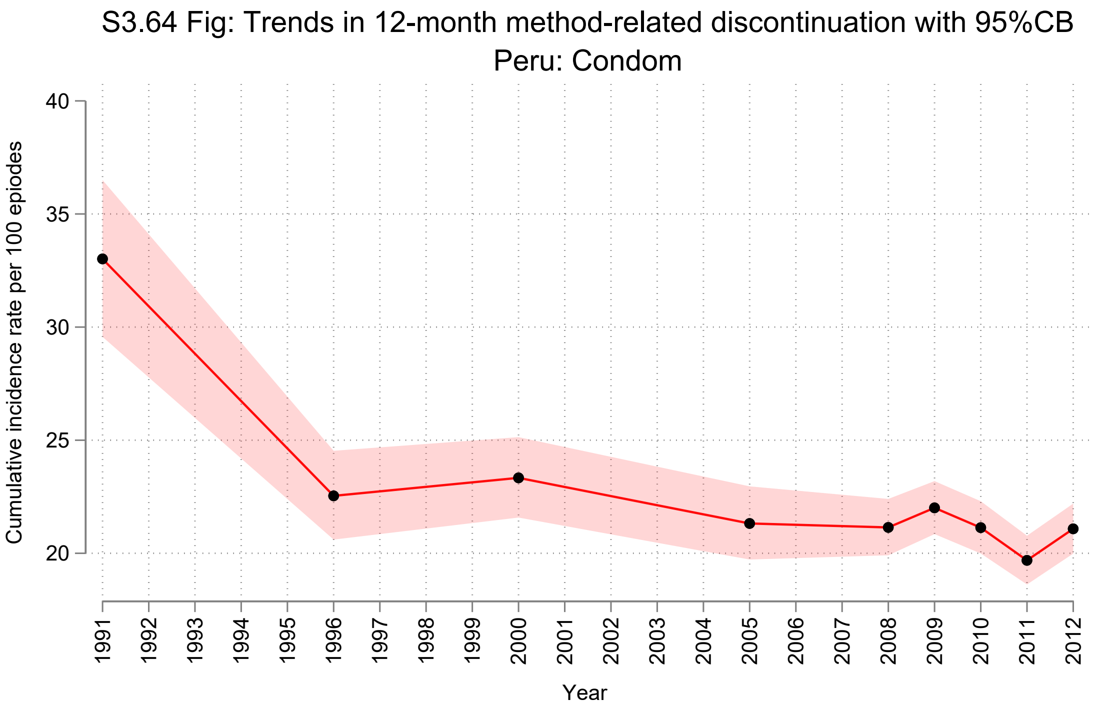
S3.64 Fig: Trends in 12-month method-related discontinuation with 95%CB
Peru: Condom
Cumulative incidence rate per 100 epiodes
40
35
30
25
20
1991
1992
1993
1994
1995
1996
1997
1998
1999
2000
2001
2002
2003
2004
2005
2006
2007
2008
2009
2010
2011
2012
Year

S3.65 Fig: Trends in 12-month method-related discontinuation with 95%CB
Peru: Periodic abstinence/rhythm

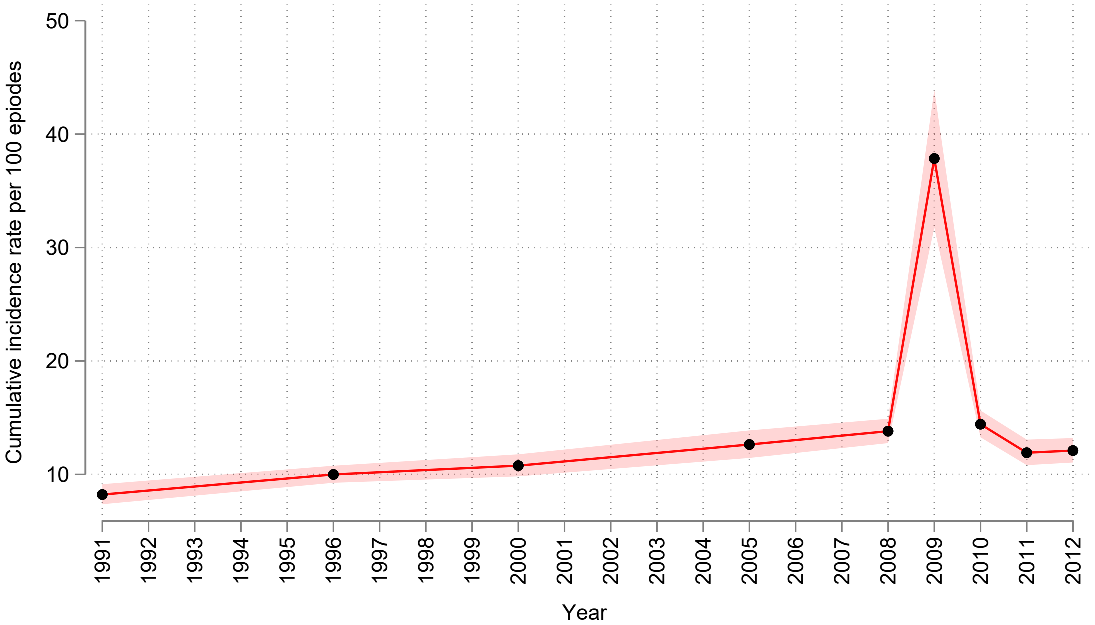

Supplement: S3 Fig — (PDF) [file pgph.0005174.s003.pdf]
