# Supplementary material for: Contraceptive discontinuation, switching, abandonment and their reproductive consequences: An analysis of 1,539,071 episodes of reversible method use contributed from 61 countries that participated in DHS: Population base-analysis
Source: PLOS Glob Public Health. 2025 Oct 31;5(10):e0005174. doi: 10.1371/journal.pgph.0005174 (PMC12578211; doi:10.1371/journal.pgph.0005174)
Supplement: S4 Fig — (PDF) [file pgph.0005174.s004.pdf]

S4.1 Fig: Cumulative incidence of method-related discontinuation at 12 months per 100 episodes with 95%CIs: Oral contraceptives

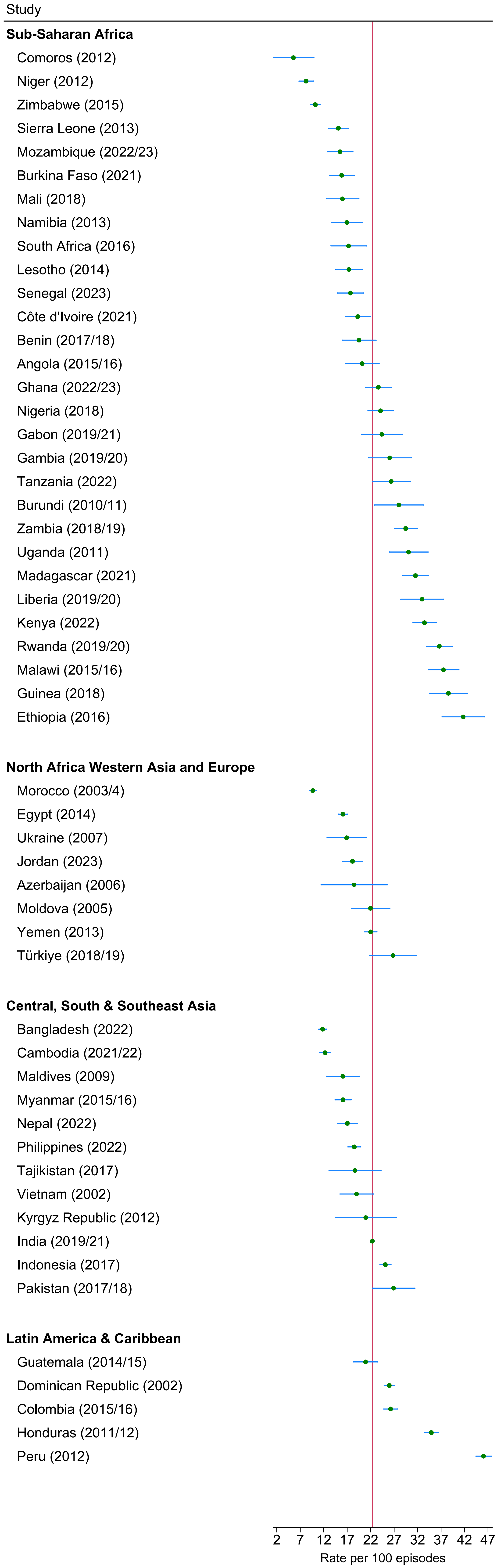

Most recent surveys since 2000

S4.2 Fig: Cumulative incidence of method-related discontinuation at 12 months per 100 episodes with 95%CIs: IUDs

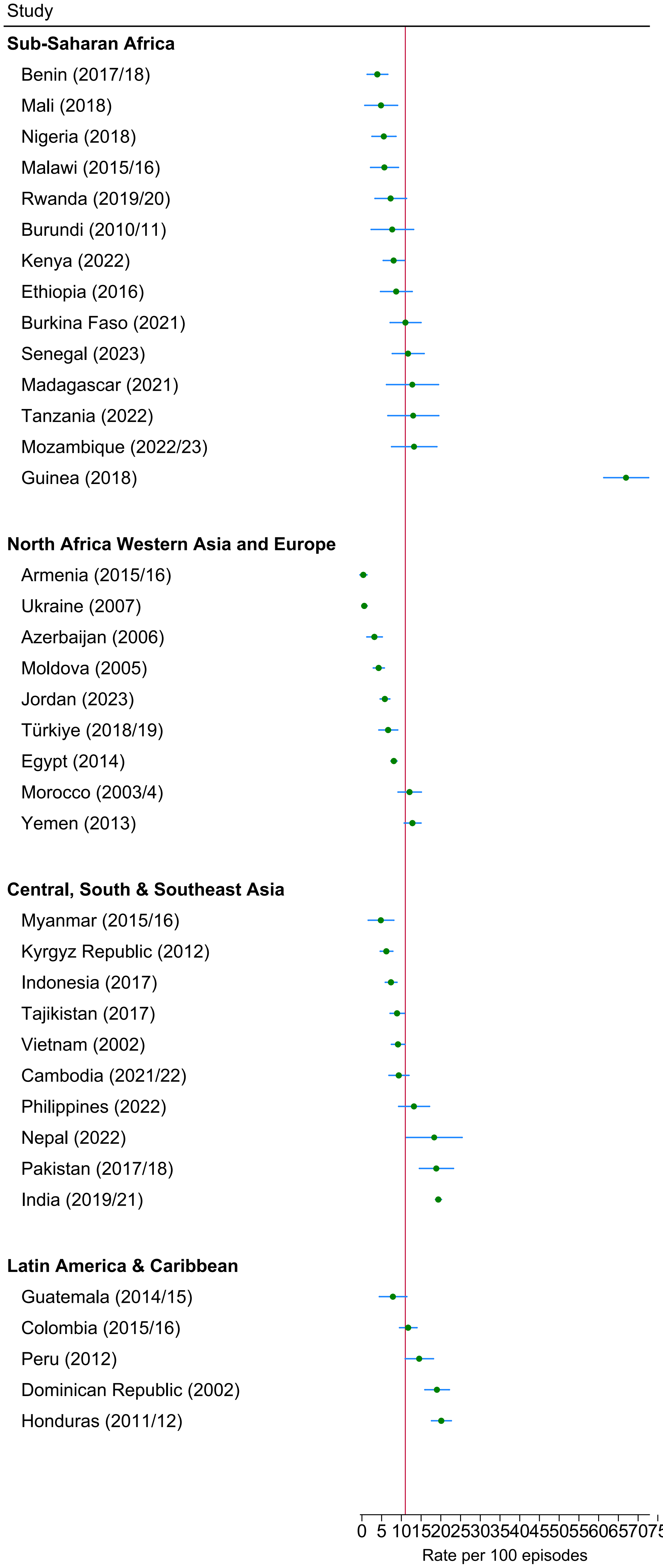

Most recent surveys since 2000

S4.3 Fig: Cumulative incidence of method-related discontinuation at 12 months per 100 episodes with 95%CIs: Injectables

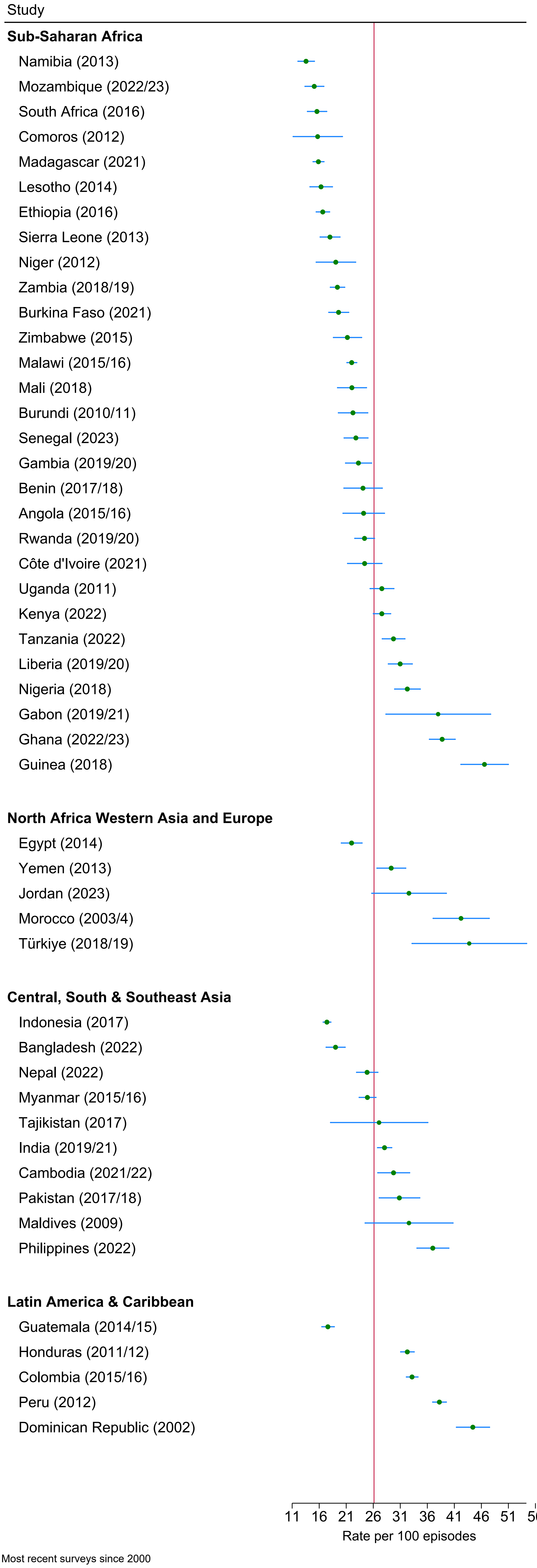

S4.4 Fig: Cumulative incidence of method-related discontinuation at 12 months per 100 episodes with 95%CIs: Condom

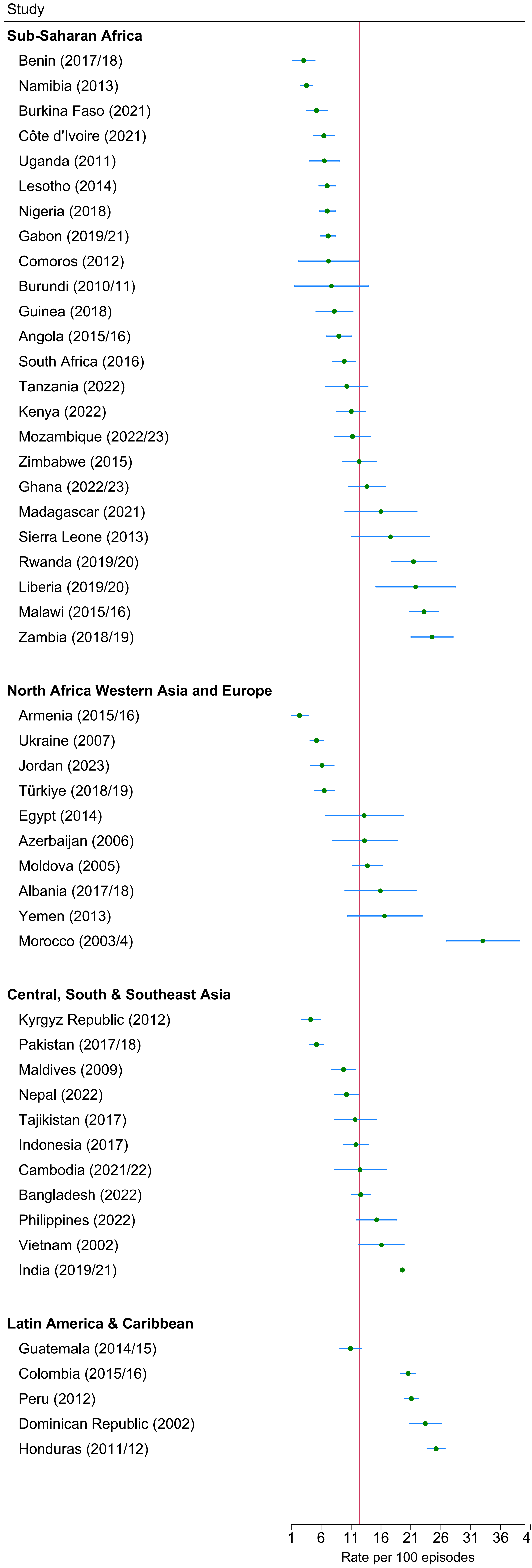

Most recent surveys since 2000

S4.5 Fig: Cumulative incidence of method-related discontinuation at 12 months per 100 episodes with 95%CIs: Implants

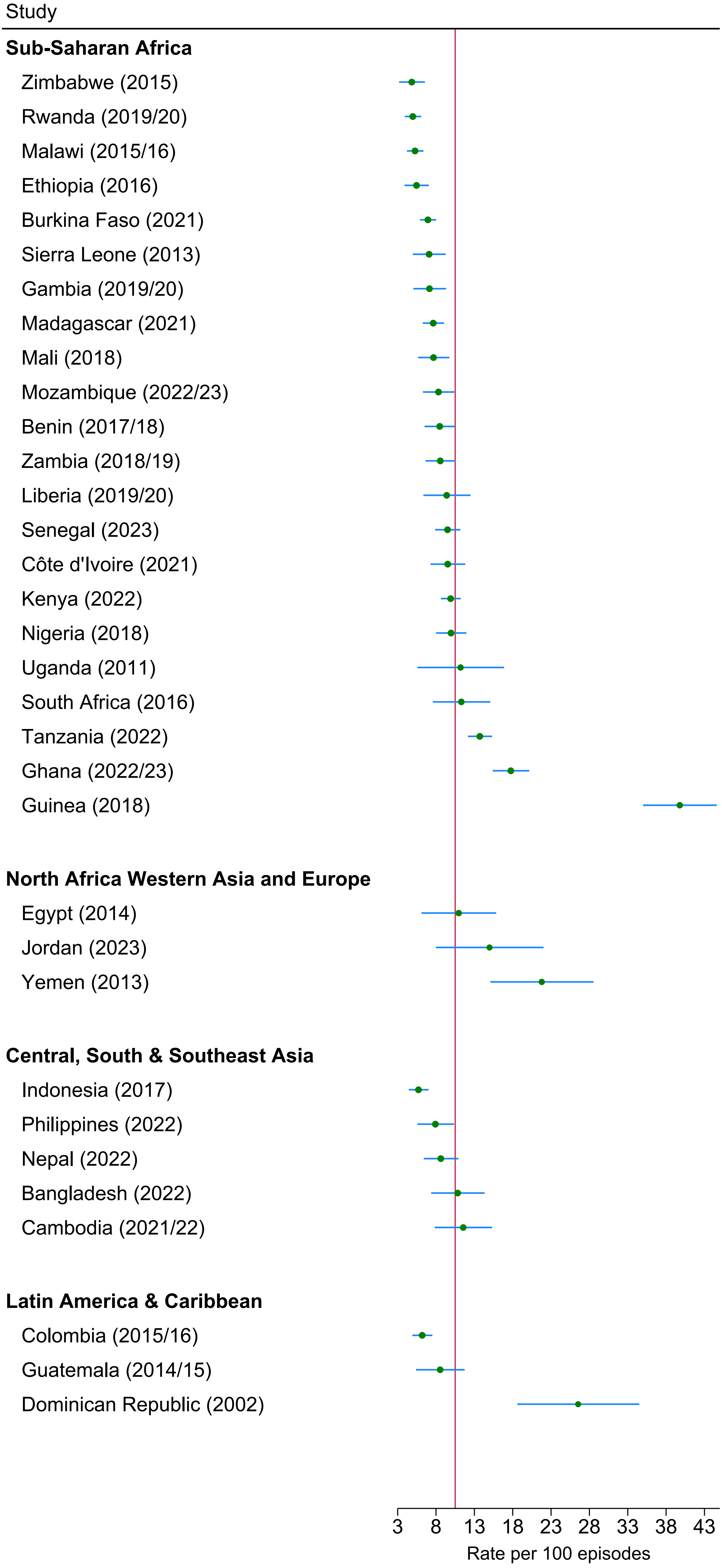

Most recent surveys since 2000

S4.6 Fig: Cumulative incidence of method-related discontinuation at 12 months per 100 episodes with 95%CIs: Periodic abstinence

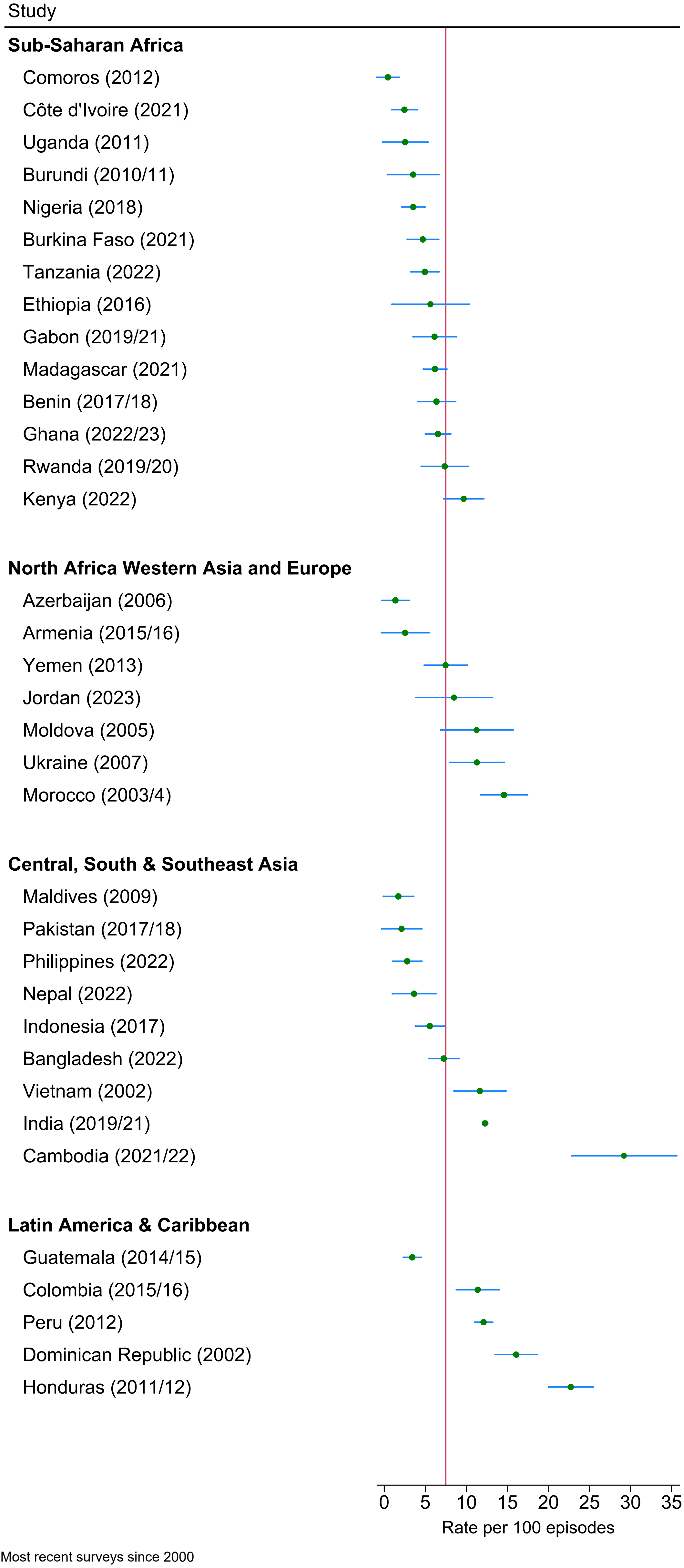

S4.7 Fig: Cumulative incidence of method-related discontinuation at 12 months per 100 episodes with 95%CIs: Withdrawal

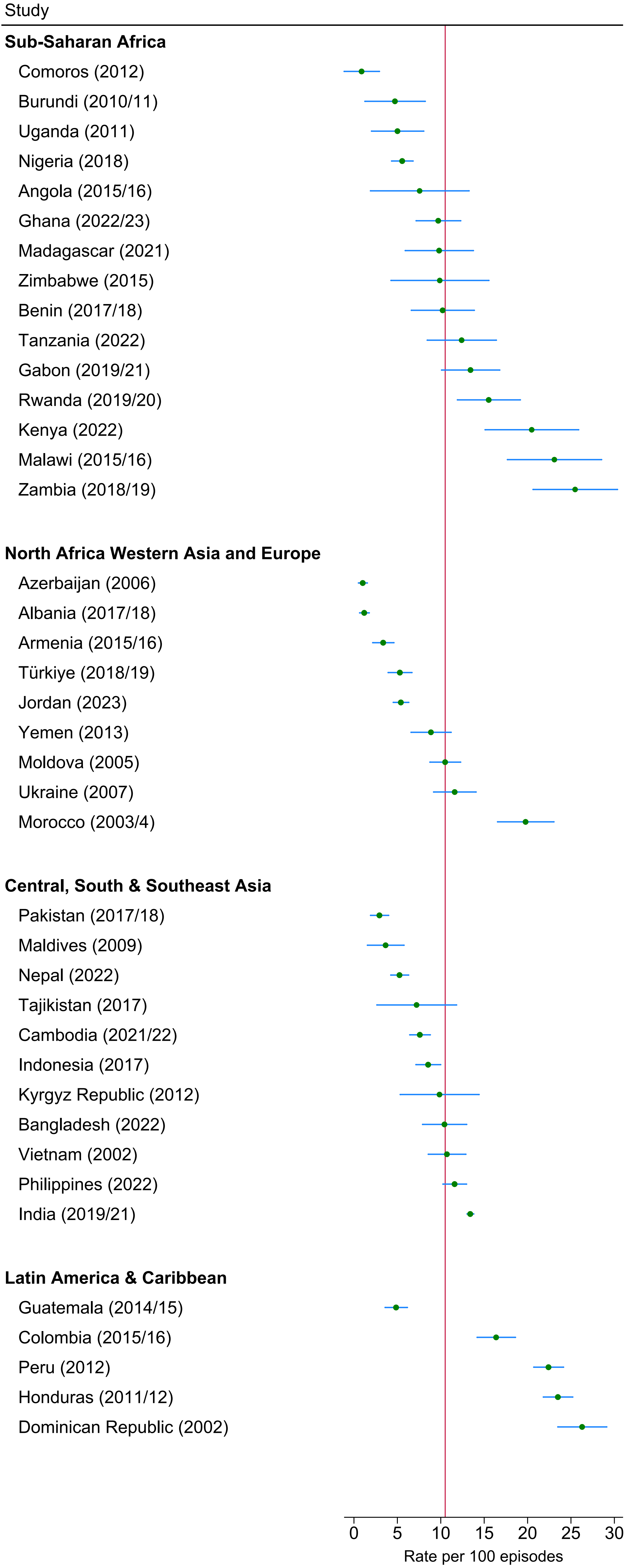

Most recent surveys since 2000
