# Supplementary material for: Contraceptive discontinuation, switching, abandonment and their reproductive consequences: An analysis of 1,539,071 episodes of reversible method use contributed from 61 countries that participated in DHS: Population base-analysis
Source: PLOS Glob Public Health. 2025 Oct 31;5(10):e0005174. doi: 10.1371/journal.pgph.0005174 (PMC12578211; doi:10.1371/journal.pgph.0005174)

S5.1 Fig: Trends in switching to any method at 3 months with 95%CB following method related discontinuation: Kenya: Oral contraceptives

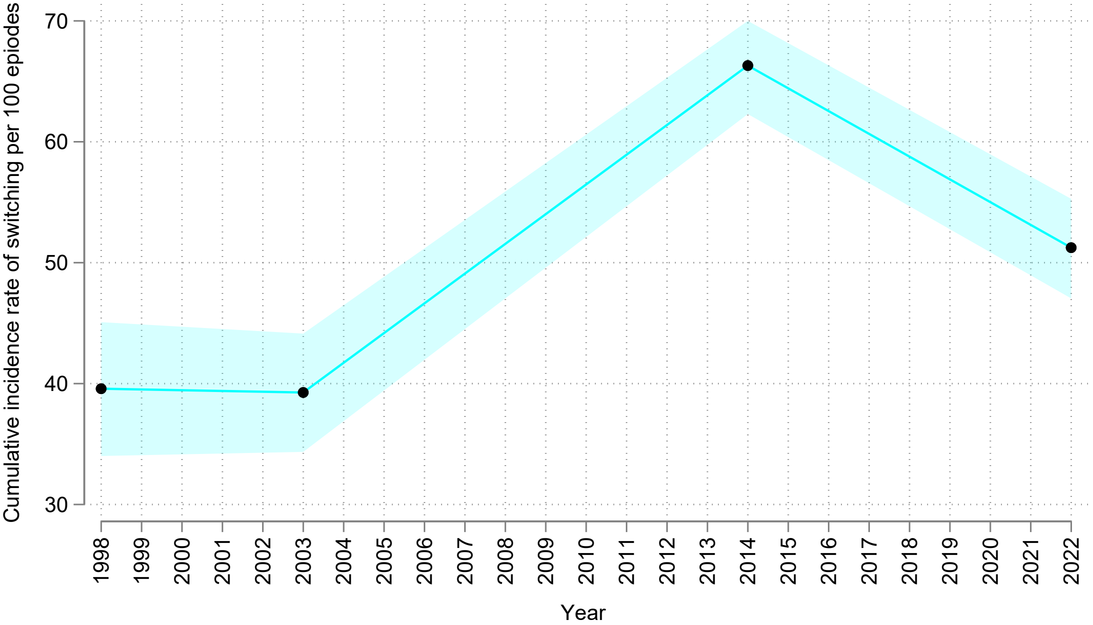

S5.2 Fig: Trends in switching to any method at 3 months with 95%CB following method related discontinuation: Rwanda: Injectables

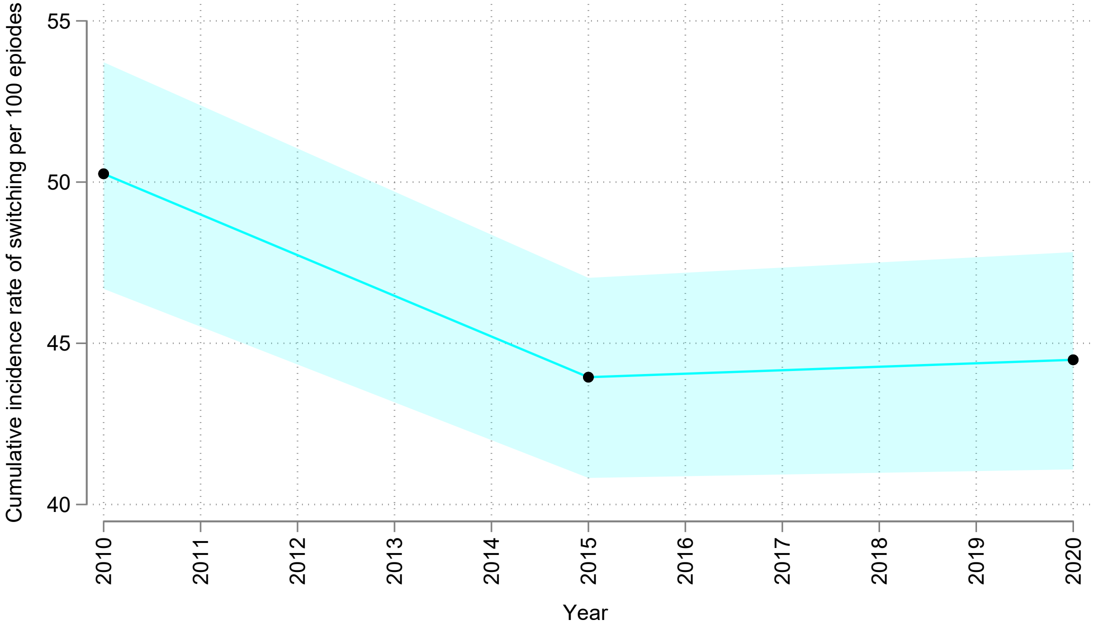

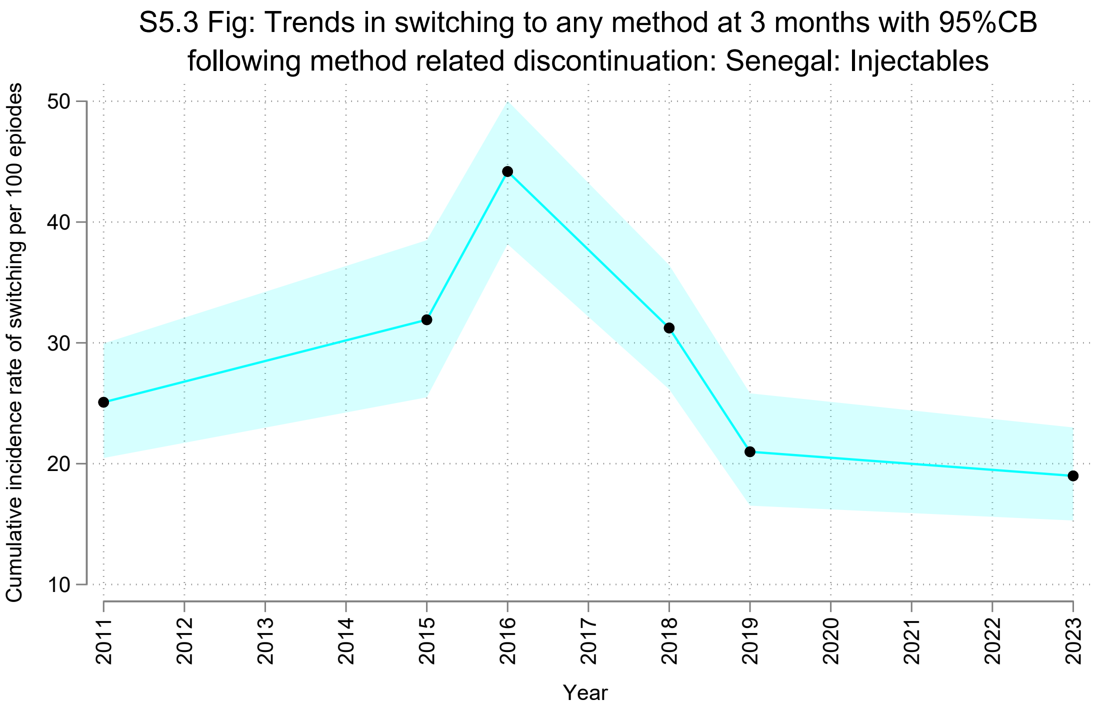

S5.3 Fig: Trends in switching to any method at 3 months with 95%CB
following method related discontinuation: Senegal: Injectables
Cumulative incidence rate of switching per 100 epiodes
10
20
30
40
50
2011
2012
2013
2014
2015
2016
2017
2018
2019
2020
2021
2022
2023
Year

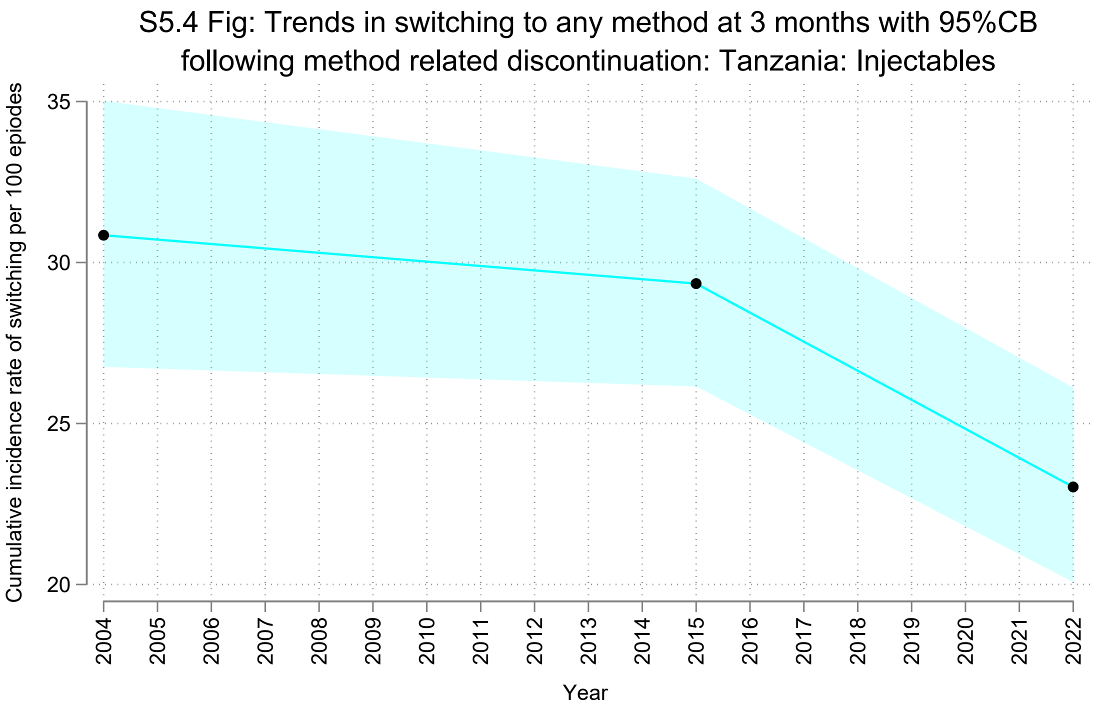

S5.4 Fig: Trends in switching to any method at 3 months with 95%CB
following method related discontinuation: Tanzania: Injectables
Cumulative incidence rate of switching per 100 epiodes
35
30
25
20
2004
2005
2006
2007
2008
2009
2010
2011
2012
2013
2014
2015
2016
2017
2018
2019
2020
2021
2022
Year

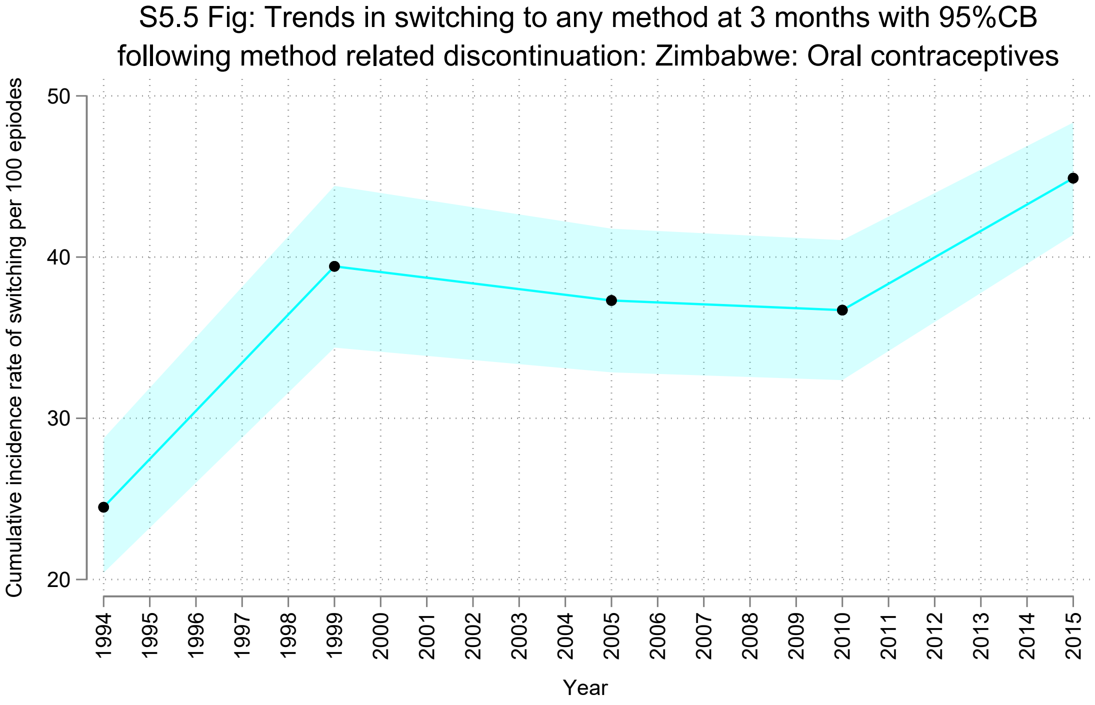
S5.5 Fig: Trends in switching to any method at 3 months with 95%CB
following method related discontinuation: Zimbabwe: Oral contraceptives
Cumulative incidence rate of switching per 100 epiodes
50
40
30
20
1994
1995
1996
1997
1998
1999
2000
2001
2002
2003
2004
2005
2006
2007
2008
2009
2010
2011
2012
2013
2014
2015
Year

S5.6 Fig: Trends in switching to any method at 3 months with 95%CB following method related discontinuation: Zimbabwe: Injectables

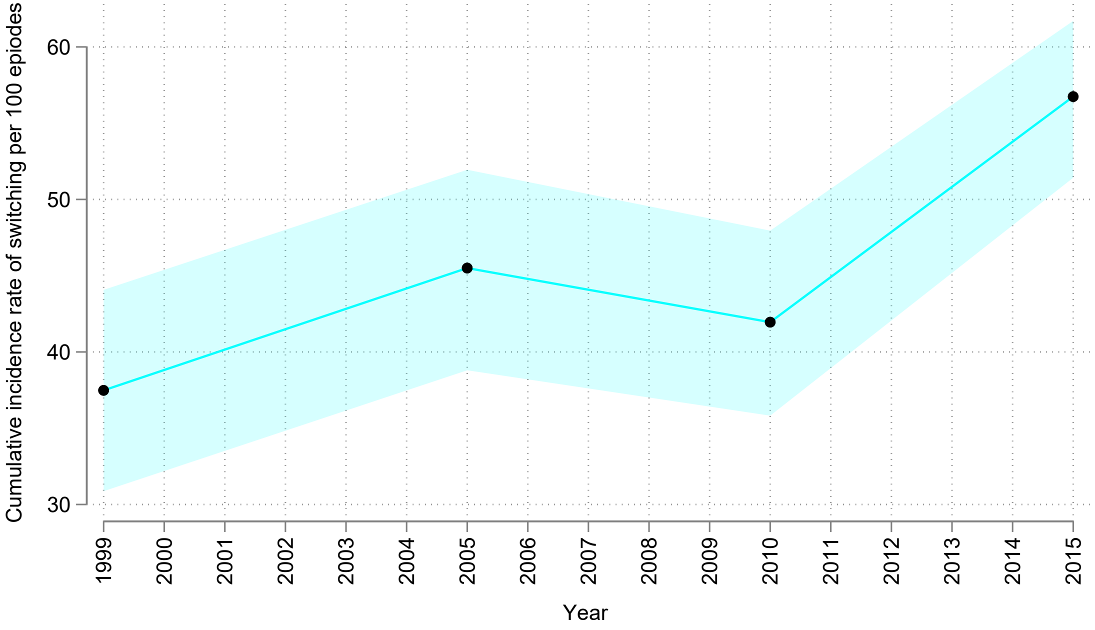

S5.7 Fig: Trends in switching to any method at 3 months with 95%CB following method related discontinuation: Egypt: Oral contraceptives

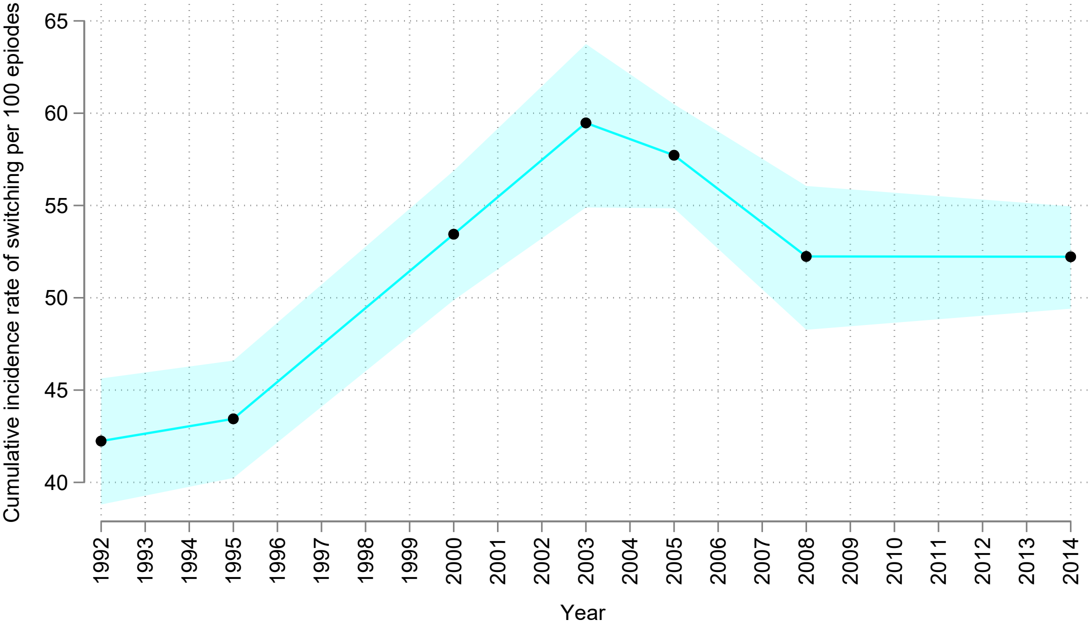

S5.8 Fig: Trends in switching to any method at 3 months with 95%CB following method related discontinuation: Egypt: IUD

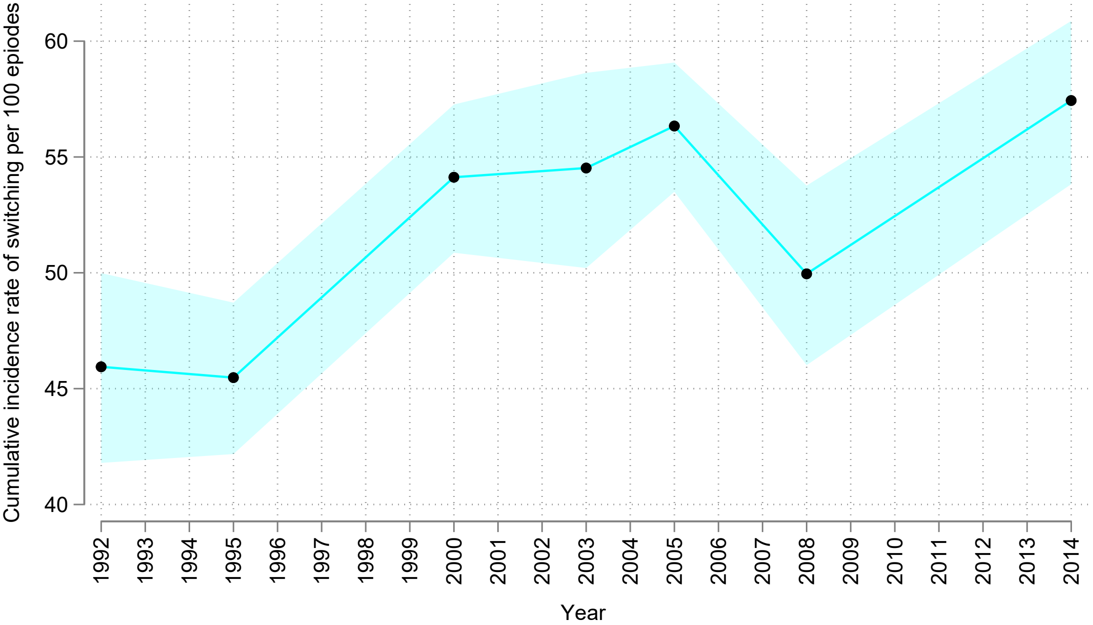

S5.9 Fig: Trends in switching to any method at 3 months with 95%CB following method related discontinuation: Jordan: Injectables

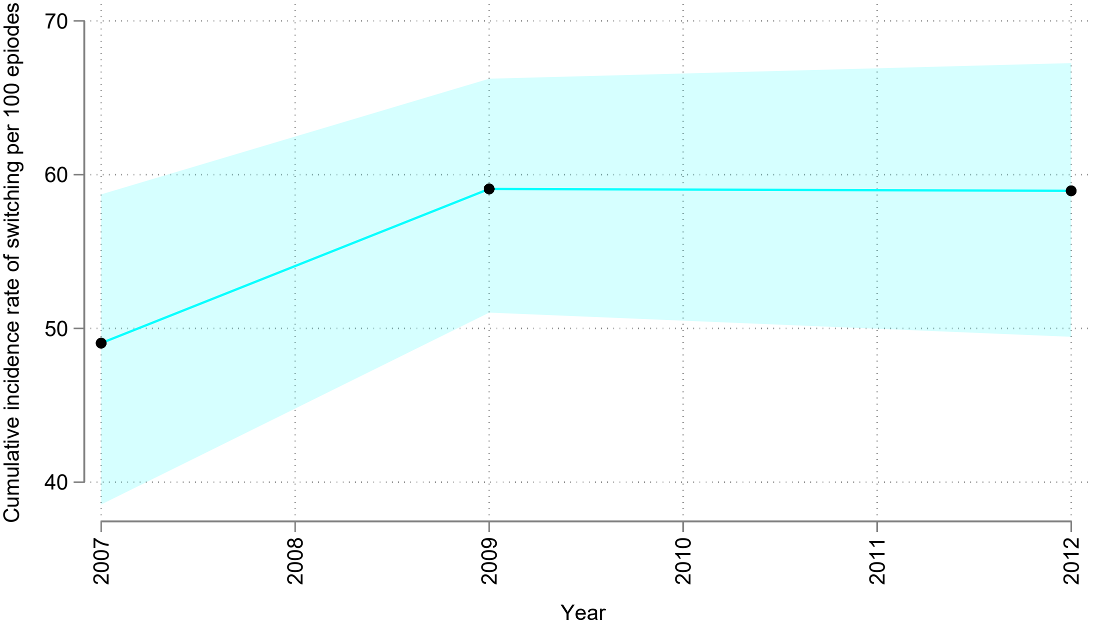

S5.10 Fig: Trends in switching to any method at 3 months with 95%CB following method related discontinuation: Jordan: Withdrawal

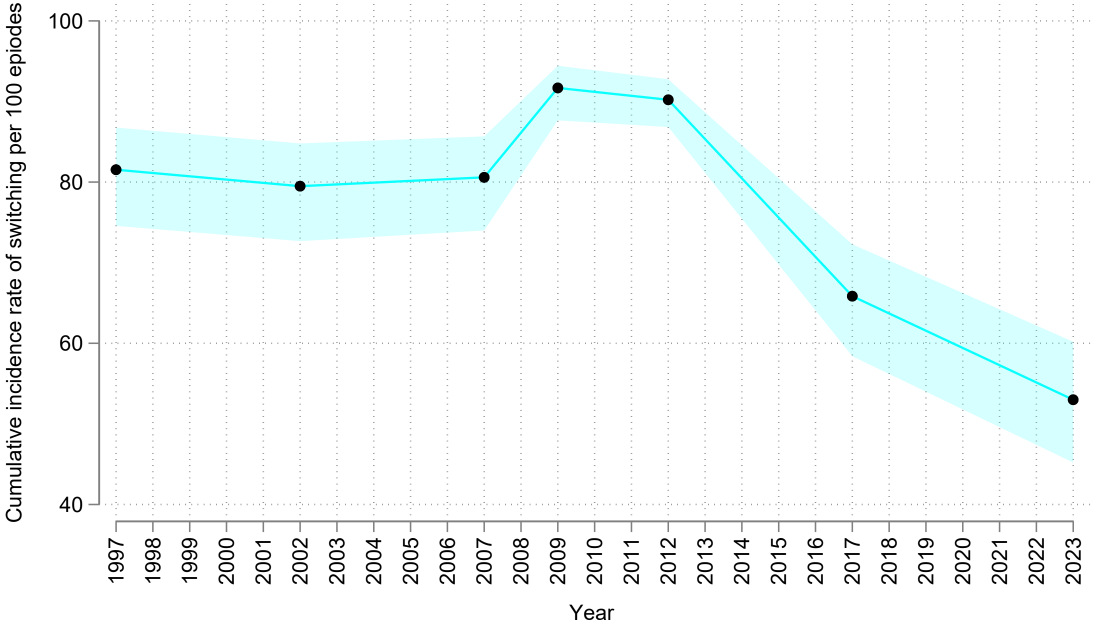

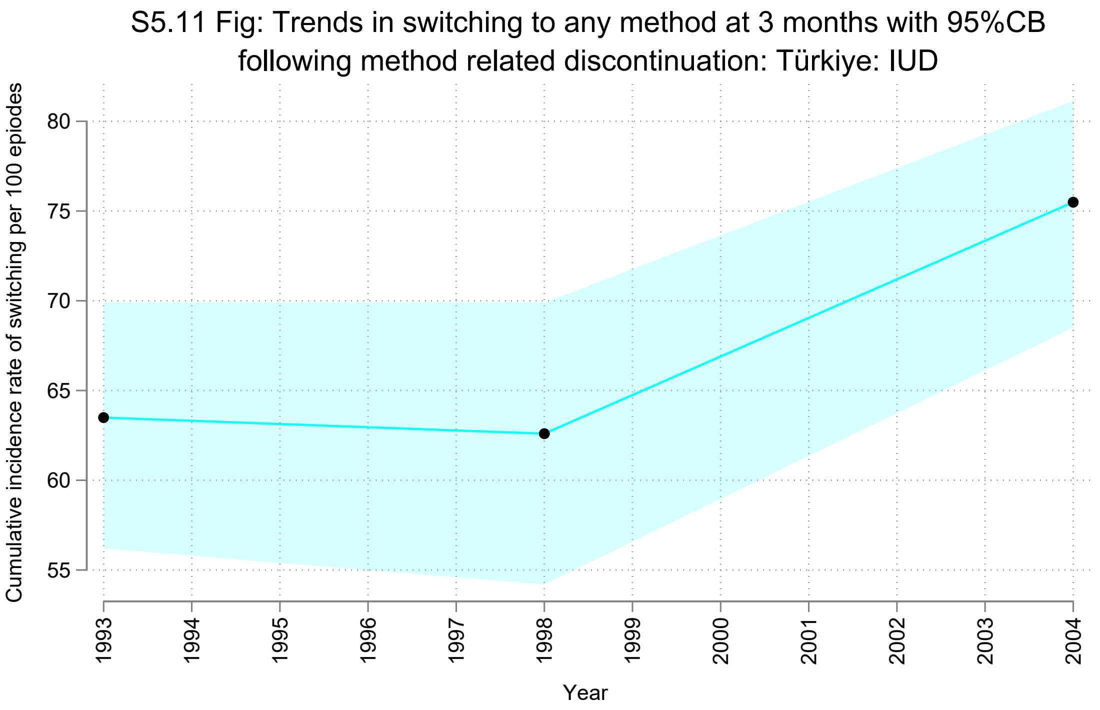
S5.11 Fig: Trends in switching to any method at 3 months with 95%CB
following method related discontinuation: Türkiye: IUD
Cumulative incidence rate of switching per 100 epiodes
80
75
70
65
60
55
1993
1994
1995
1996
1997
1998
1999
2000
2001
2002
2003
2004
Year

S5.12 Fig: Trends in switching to any method at 3 months with 95%CB following method related discontinuation: Bangladesh: Oral contraceptives

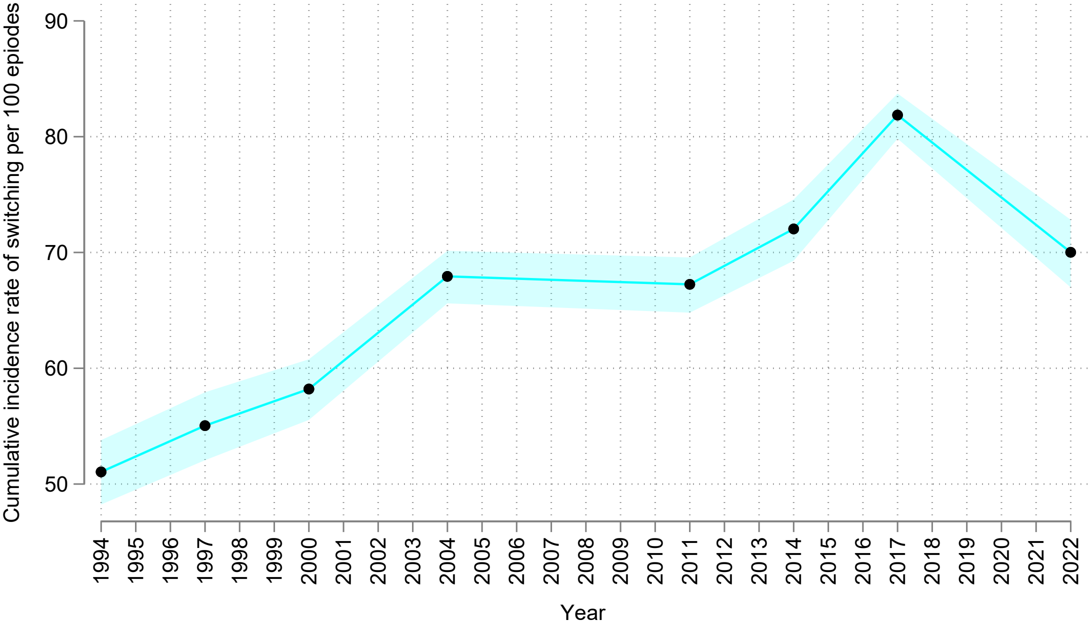

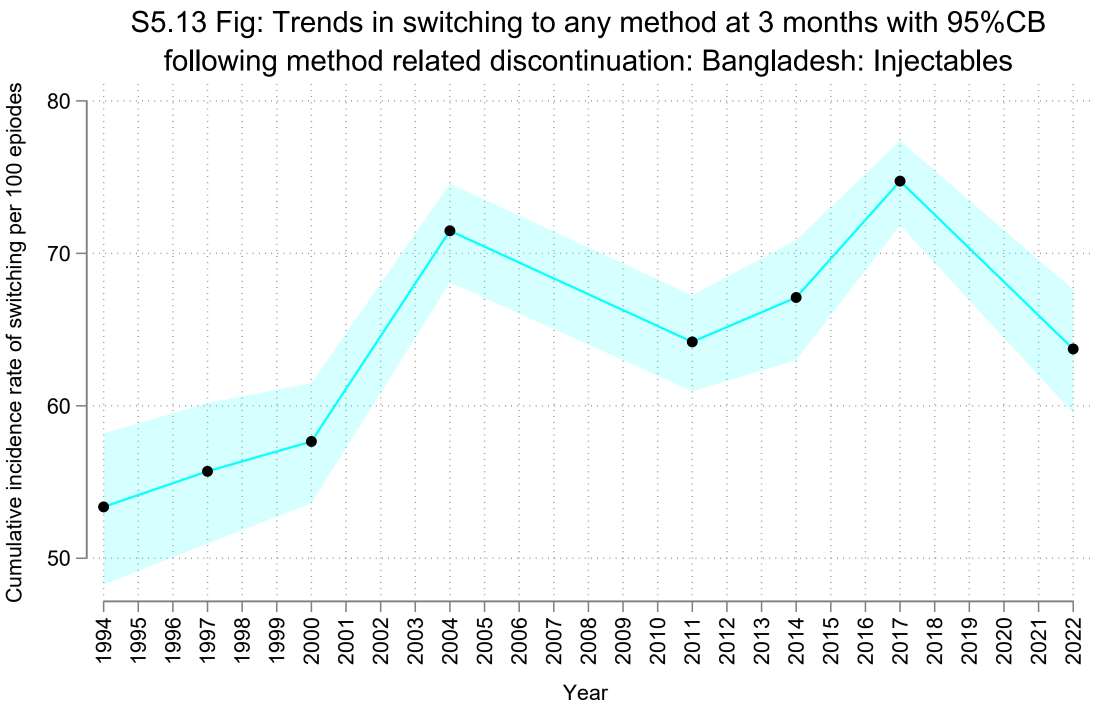
S5.13 Fig: Trends in switching to any method at 3 months with 95%CB
following method related discontinuation: Bangladesh: Injectables
Cumulative incidence rate of switching per 100 epiodes
80
70
60
50
1994
1995
1996
1997
1998
1999
2000
2001
2002
2003
2004
2005
2006
2007
2008
2009
2010
2011
2012
2013
2014
2015
2016
2017
2018
2019
2020
2021
2022
Year

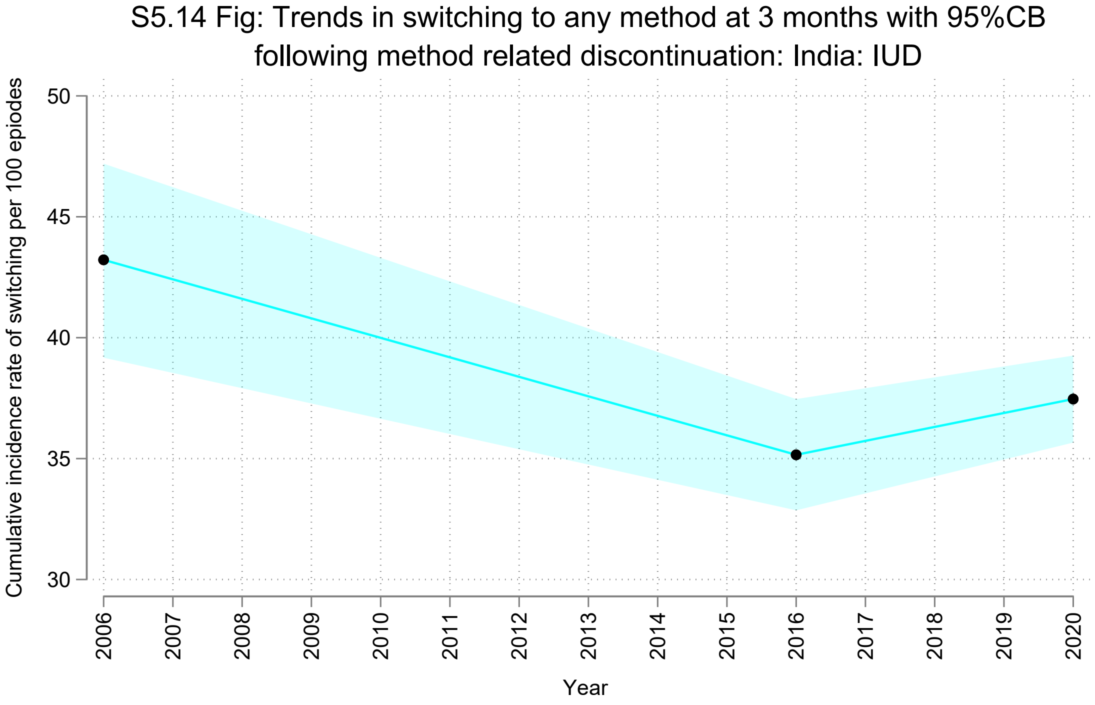
S5.14 Fig: Trends in switching to any method at 3 months with 95%CB following method related discontinuation: India: IUD
Cumulative incidence rate of switching per 100 epiodes
50
45
40
35
30
2006
2007
2008
2009
2010
2011
2012
2013
2014
2015
2016
2017
2018
2019
2020
Year

S5.15 Fig: Trends in switching to any method at 3 months with 95%CB following method related discontinuation: India: Condom

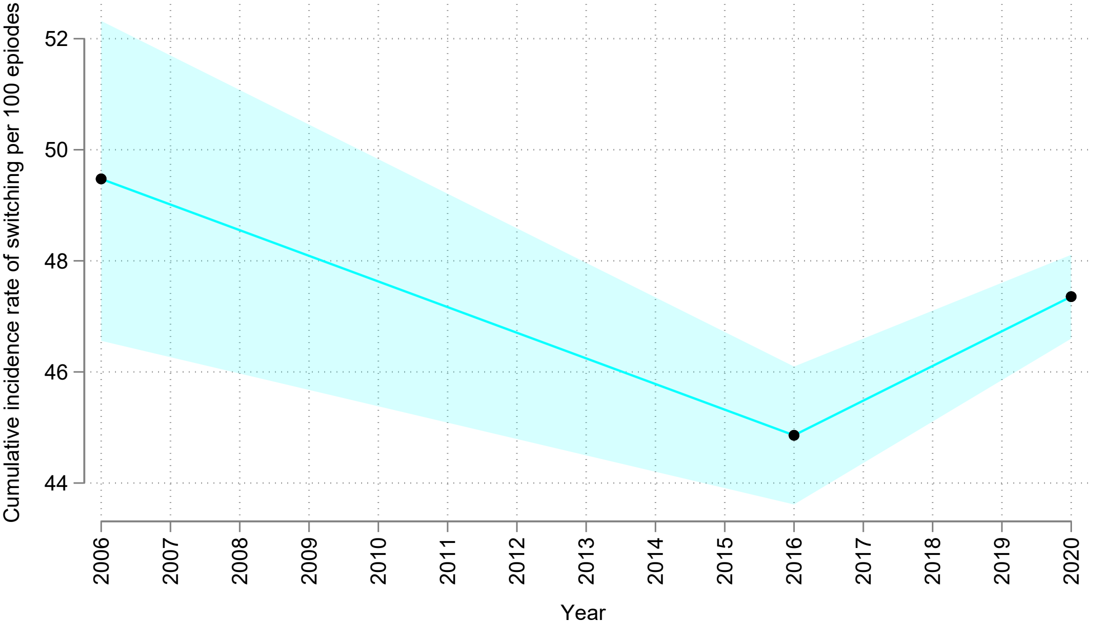

S5.16 Fig: Trends in switching to any method at 3 months with 95%CB following method related discontinuation: India: Periodic abstinence/rhythm

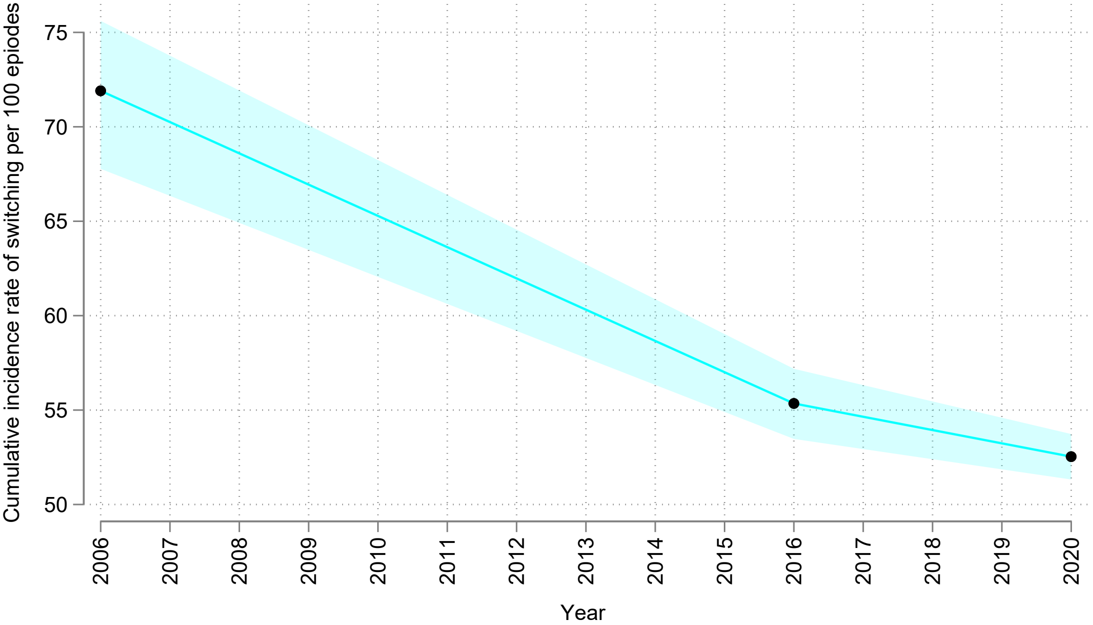

S5.17 Fig: Trends in switching to any method at 3 months with 95%CB following method related discontinuation: India: Withdrawal

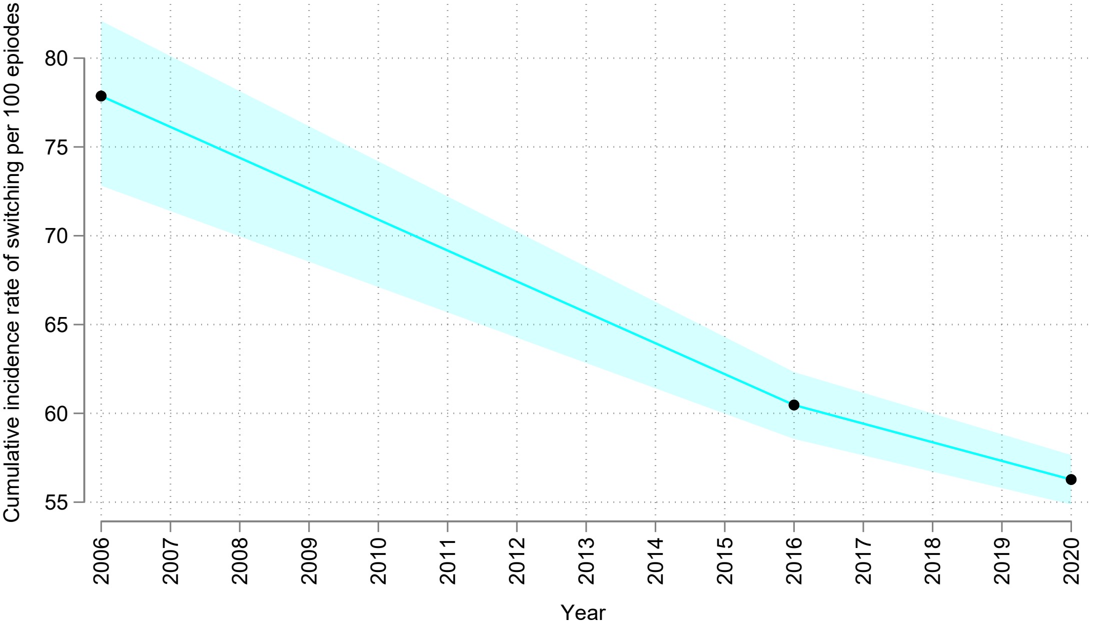

S5.18 Fig: Trends in switching to any method at 3 months with 95%CB following method related discontinuation: Indonesia: Oral contraceptives

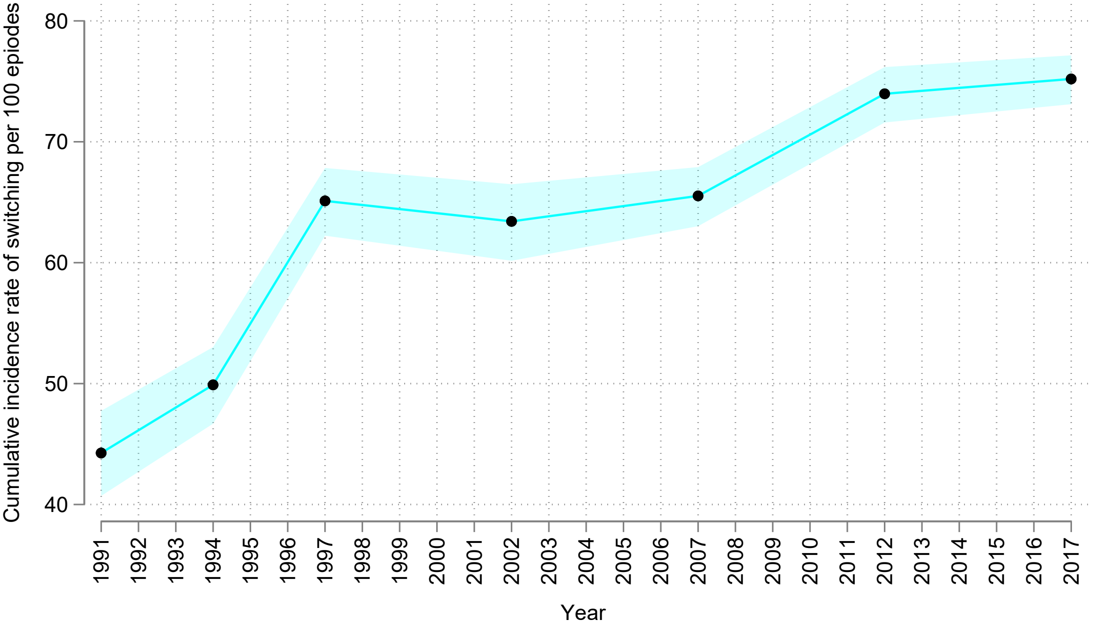

S5.19 Fig: Trends in switching to any method at 3 months with 95%CB following method related discontinuation: Nepal: Oral contraceptives

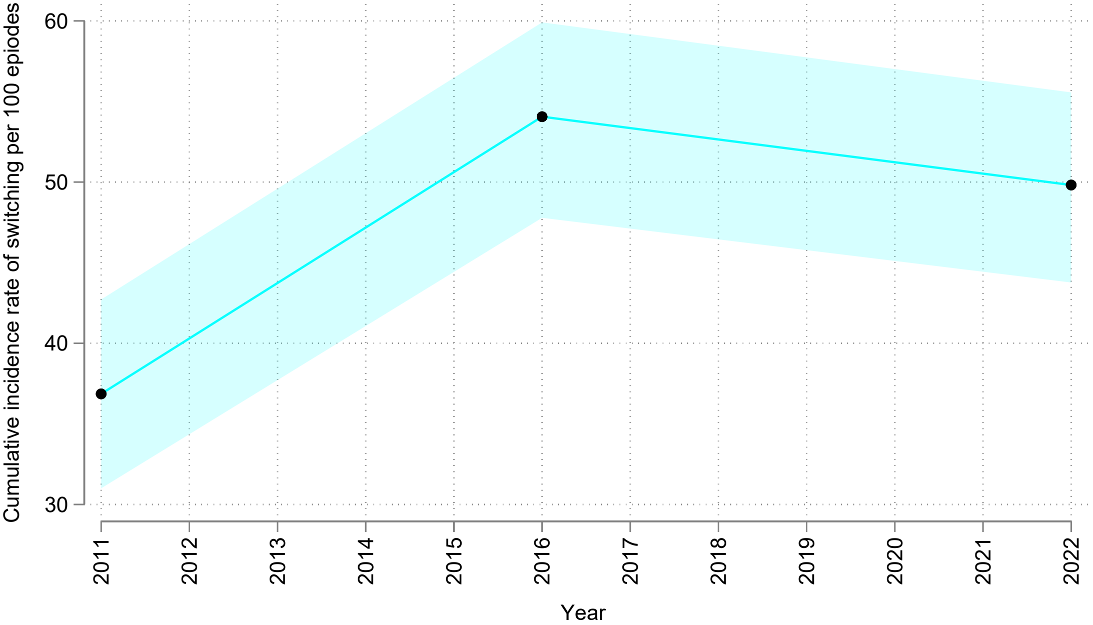

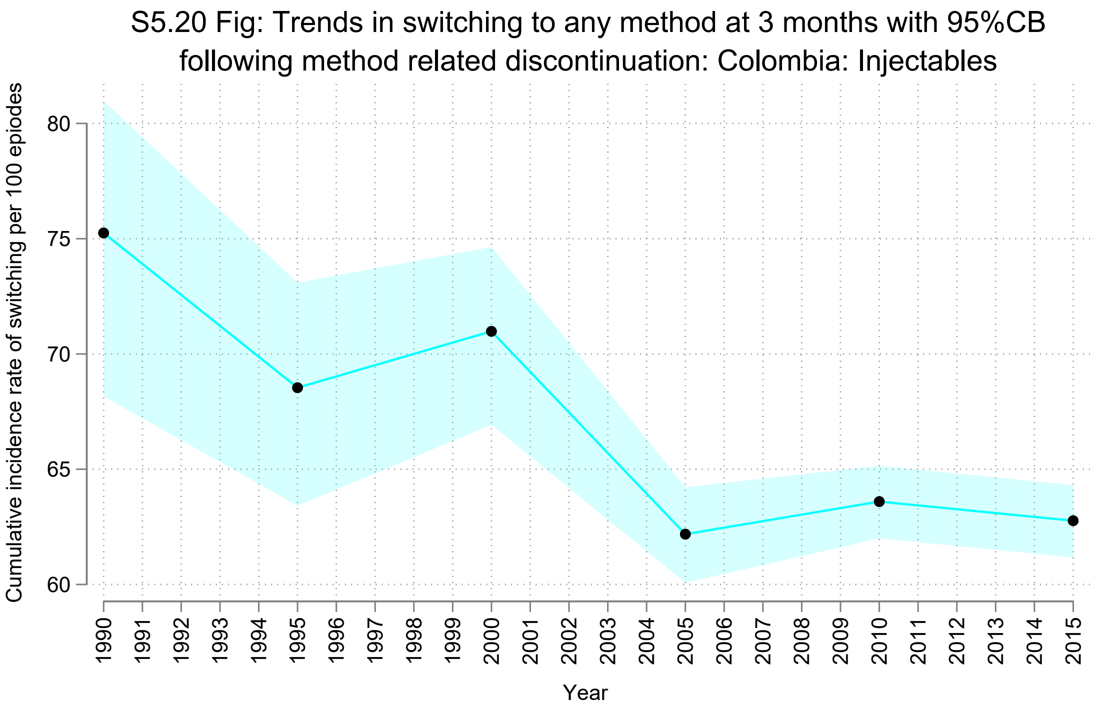

S5.20 Fig: Trends in switching to any method at 3 months with 95%CB
following method related discontinuation: Colombia: Injectables
Cumulative incidence rate of switching per 100 epiodes
80
75
70
65
60
1990
1991
1992
1993
1994
1995
1996
1997
1998
1999
2000
2001
2002
2003
2004
2005
2006
2007
2008
2009
2010
2011
2012
2013
2014
2015
Year

S5.21 Fig: Trends in switching to any method at 3 months with 95%CB following method related discontinuation: Colombia: Condom

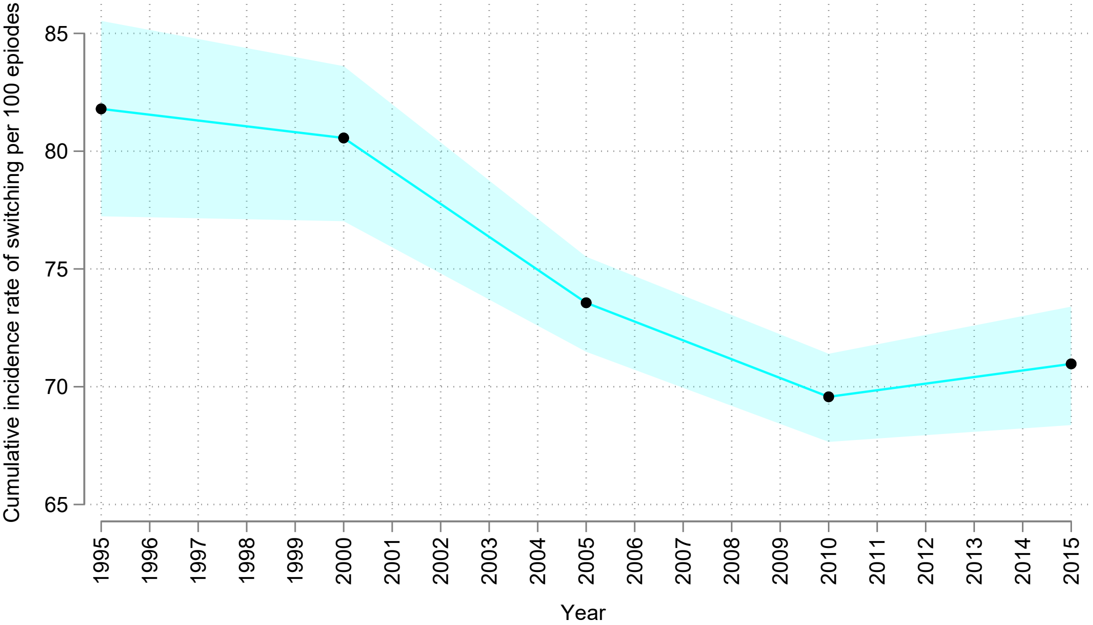

S5.22 Fig: Trends in switching to any method at 3 months with 95%CB following method related discontinuation: Colombia: Periodic abstinence/rhythm

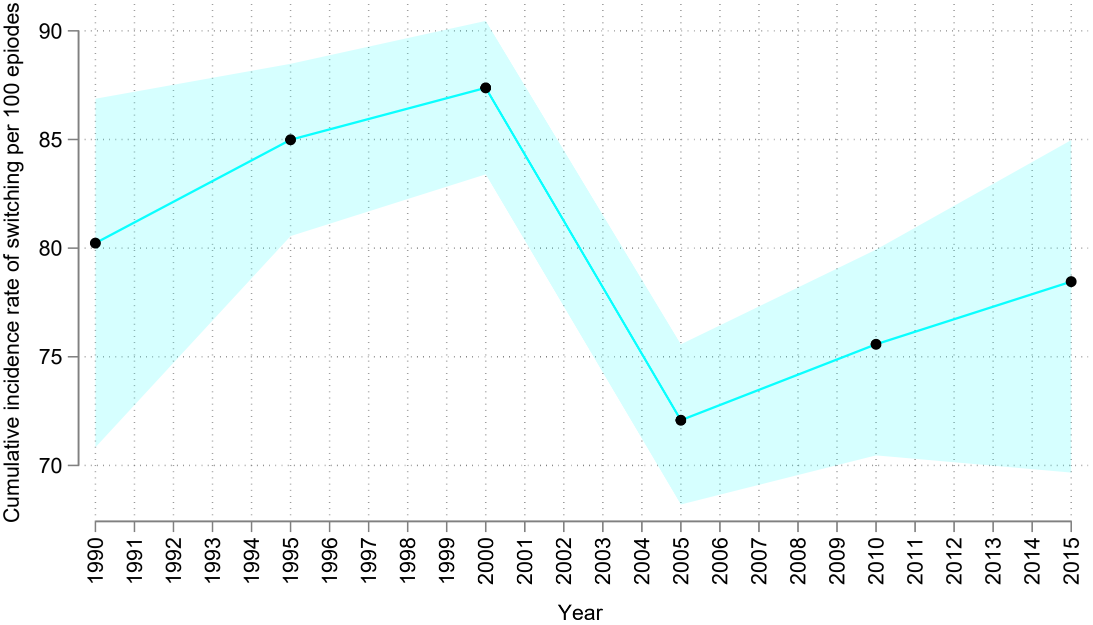

S5.23 Fig: Trends in switching to any method at 3 months with 95%CB following method related discontinuation: Colombia: Withdrawal

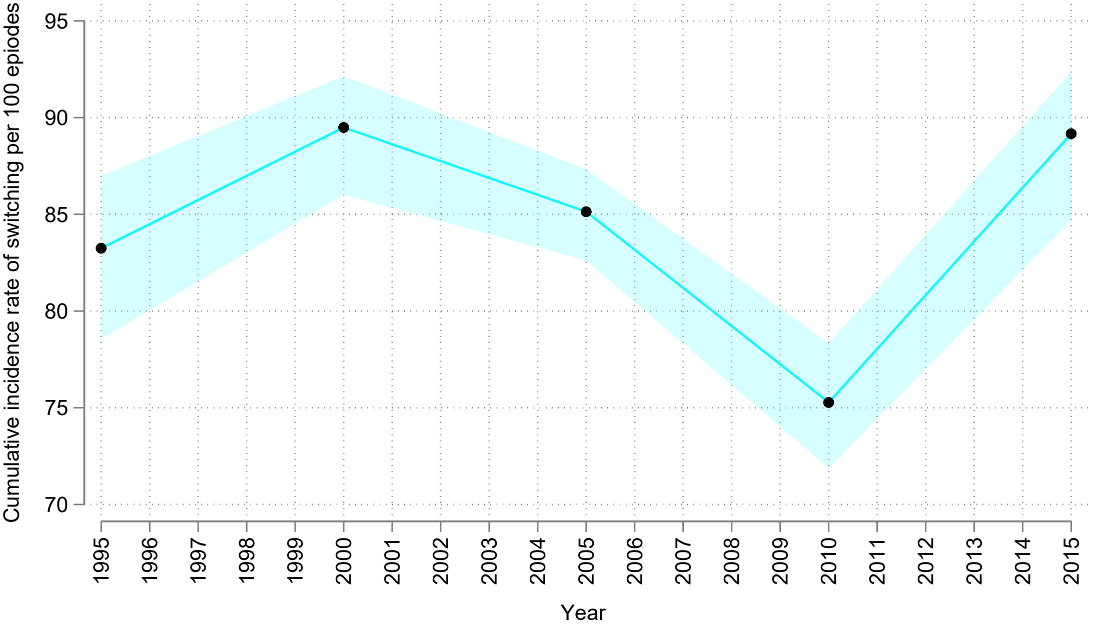

S5.24 Fig: Trends in switching to any method at 3 months with 95%CB following method related discontinuation: Guatemala: Oral contraceptives

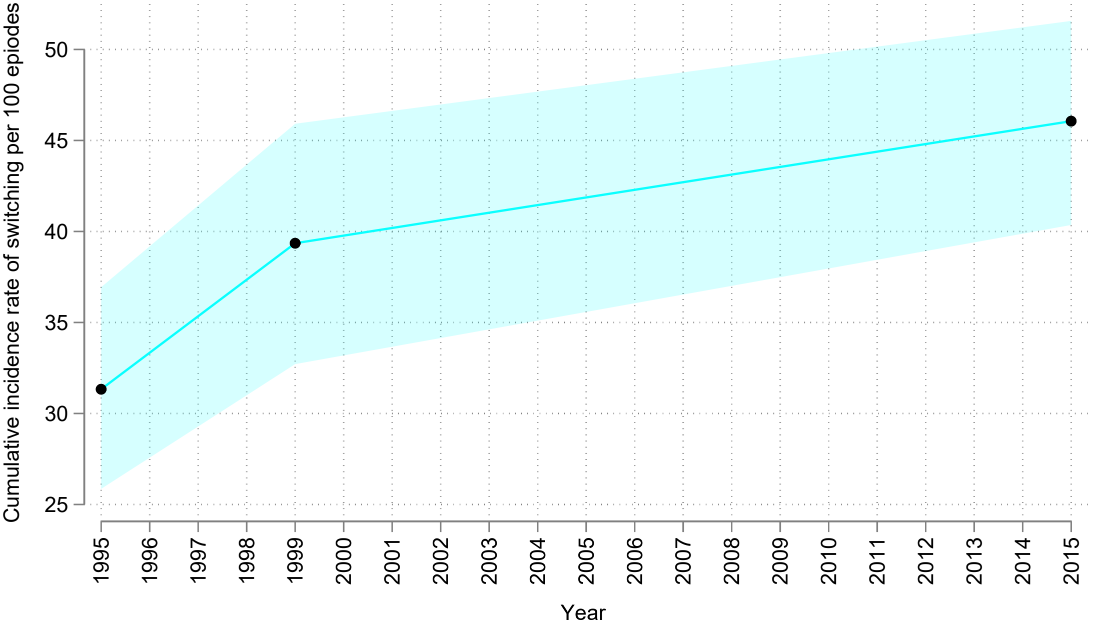

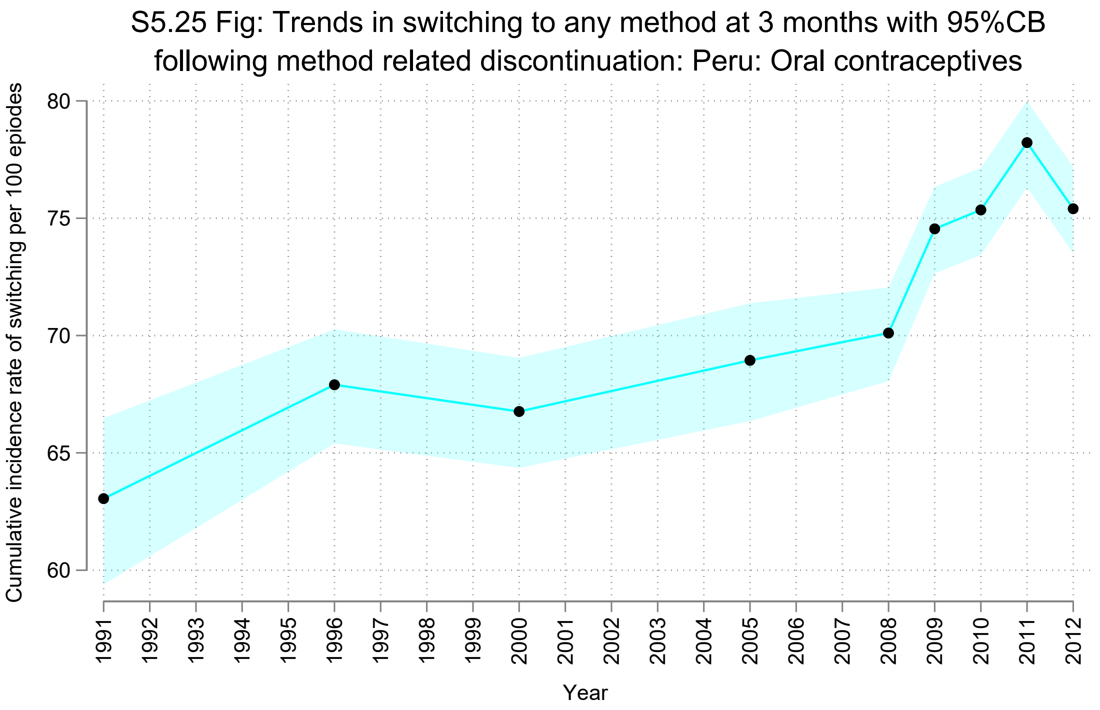
S5.25 Fig: Trends in switching to any method at 3 months with 95%CB
following method related discontinuation: Peru: Oral contraceptives
Cumulative incidence rate of switching per 100 epiodes
80
75
70
65
60
1991
1992
1993
1994
1995
1996
1997
1998
1999
2000
2001
2002
2003
2004
2005
2006
2007
2008
2009
2010
2011
2012
Year

S5.26 Fig: Trends in switching to any method at 3 months with 95%CB following method related discontinuation: Peru: IUD

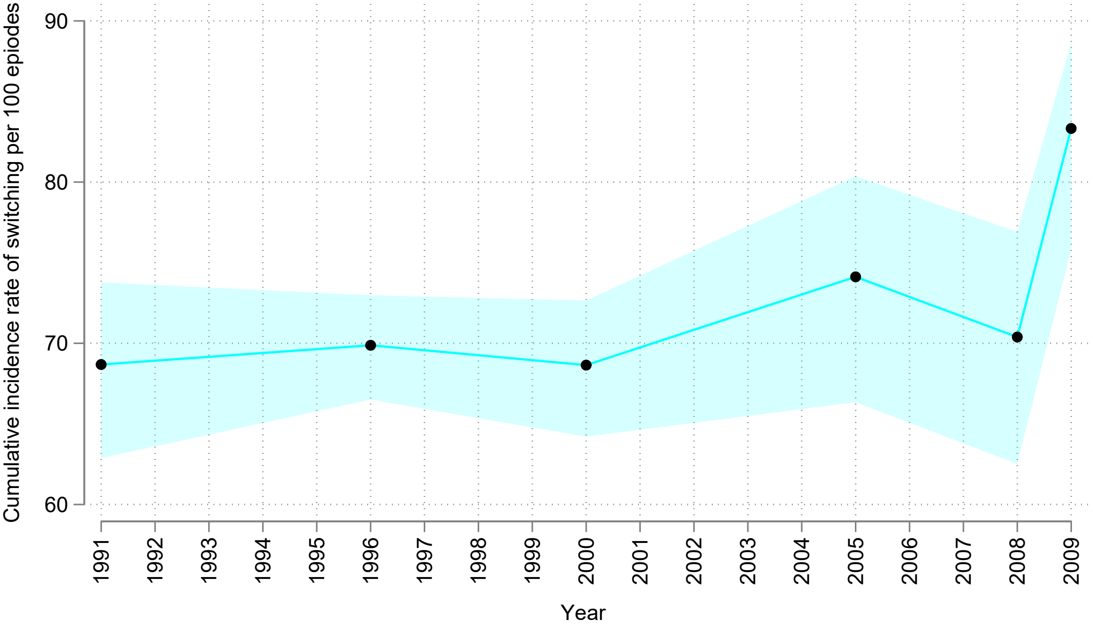

S5.27 Fig: Trends in switching to any method at 3 months with 95%CB following method related discontinuation: Peru: Injectables

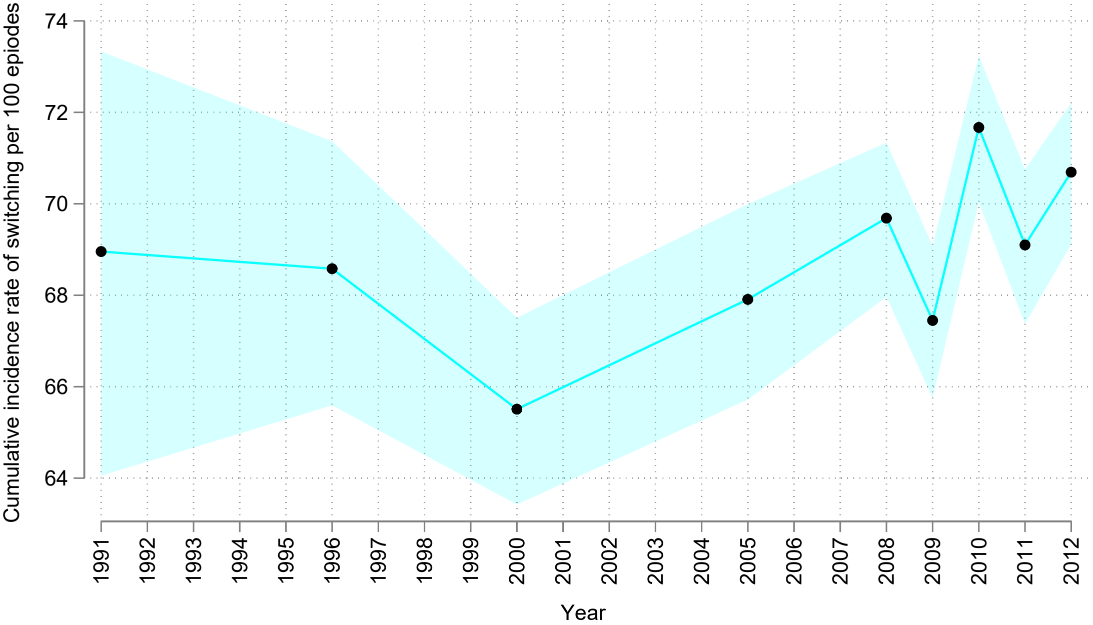

S5.28 Fig: Trends in switching to any method at 3 months with 95%CB following method related discontinuation: Peru: Periodic abstinence/rhythm

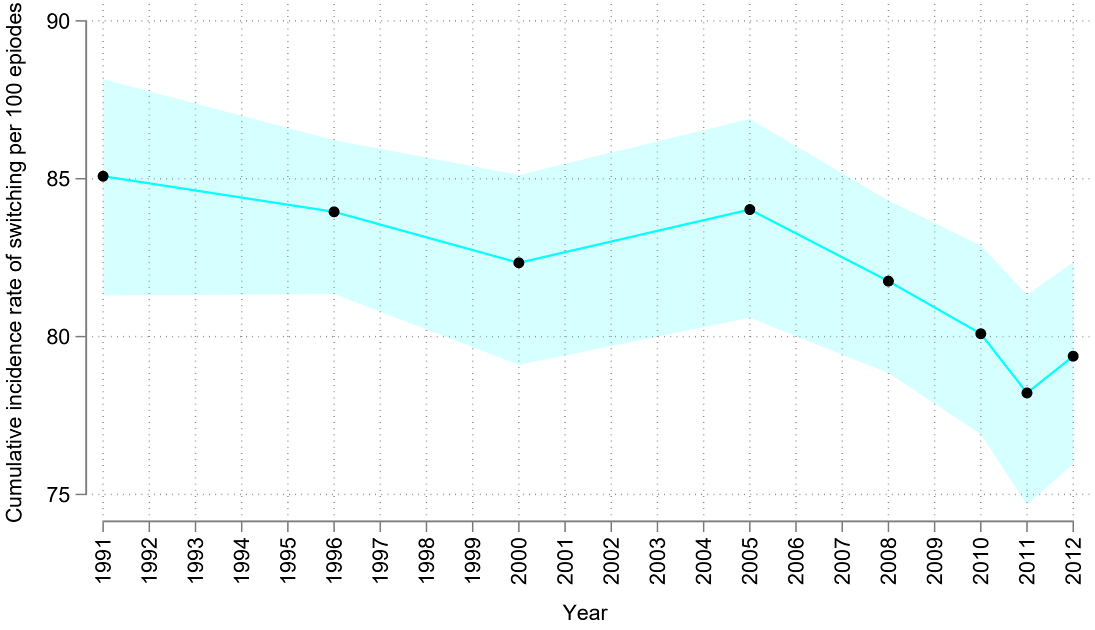

S5.29 Fig: Trends in switching to any method at 3 months with 95%CB following method related discontinuation: Peru: Withdrawal

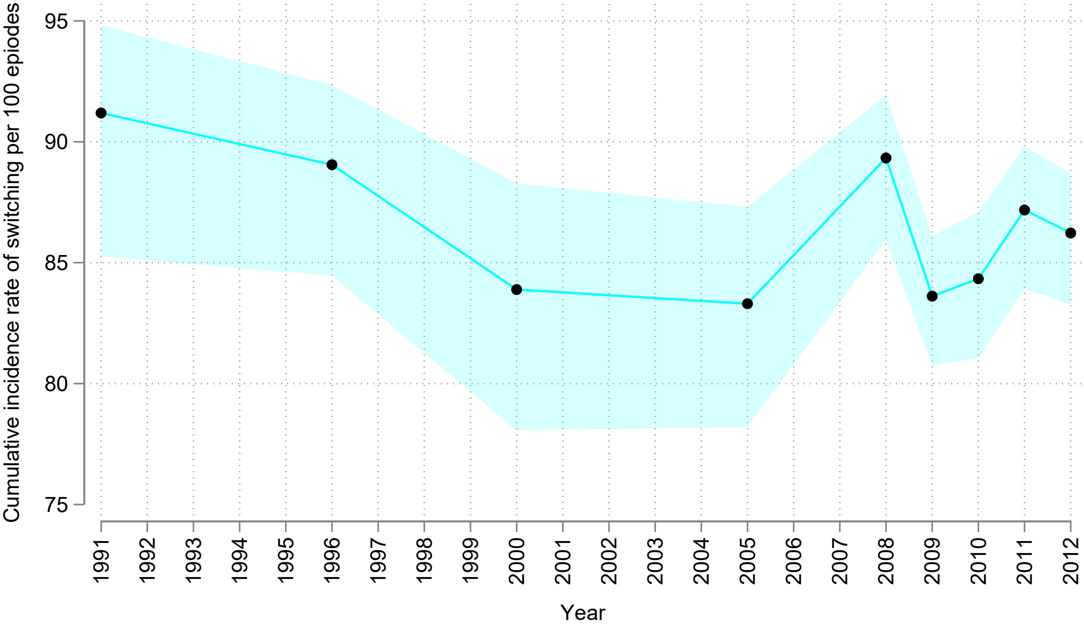

Supplement: S5 Fig — (PDF) [file pgph.0005174.s006.pdf]
