# Supplementary material for: Contraceptive discontinuation, switching, abandonment and their reproductive consequences: An analysis of 1,539,071 episodes of reversible method use contributed from 61 countries that participated in DHS: Population base-analysis
Source: PLOS Glob Public Health. 2025 Oct 31;5(10):e0005174. doi: 10.1371/journal.pgph.0005174 (PMC12578211; doi:10.1371/journal.pgph.0005174)
Supplement: S6 Fig — (PDF) [file pgph.0005174.s007.pdf]

S6.1 Fig: Cumulative incidence of switching to any method within 3 months following method-related discontinuation with 95%CIs

Oral contraceptives

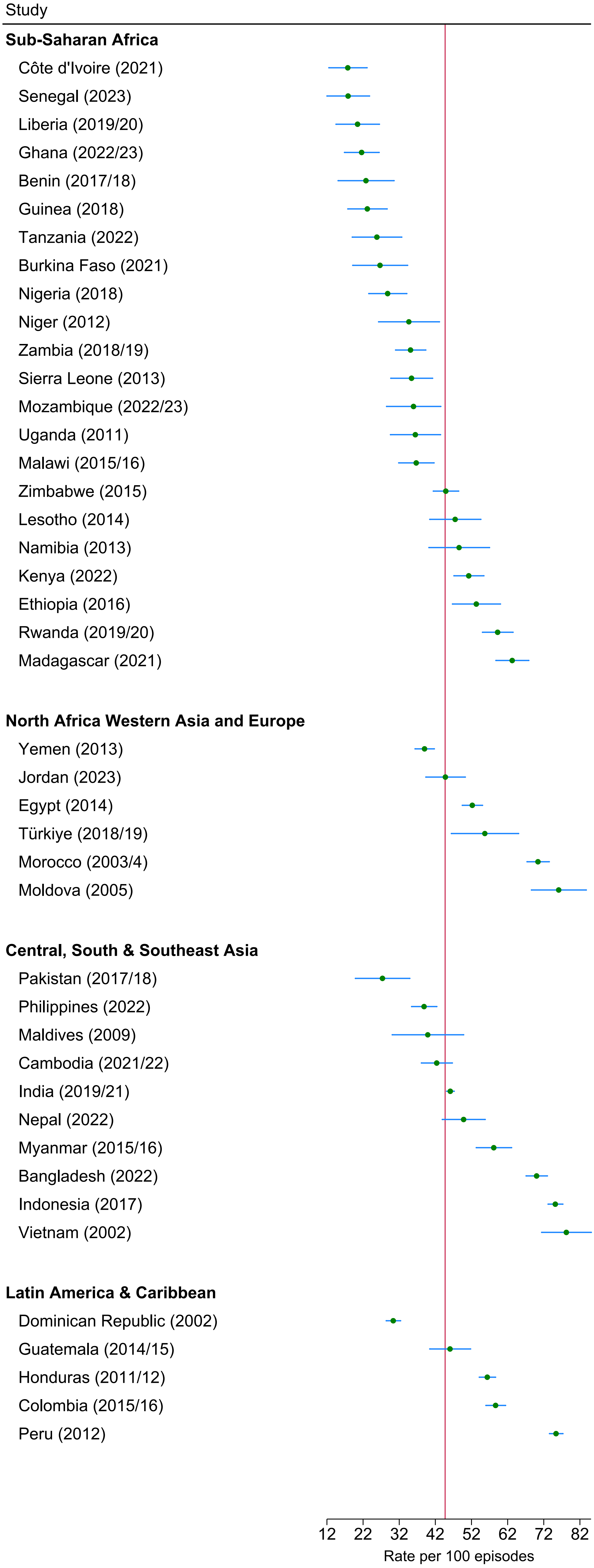

Most recent surveys since 2000

S6.2 Fig: Cumulative incidence of switching to any method within 3 months following method-related discontinuation with 95%CIs

IUDs

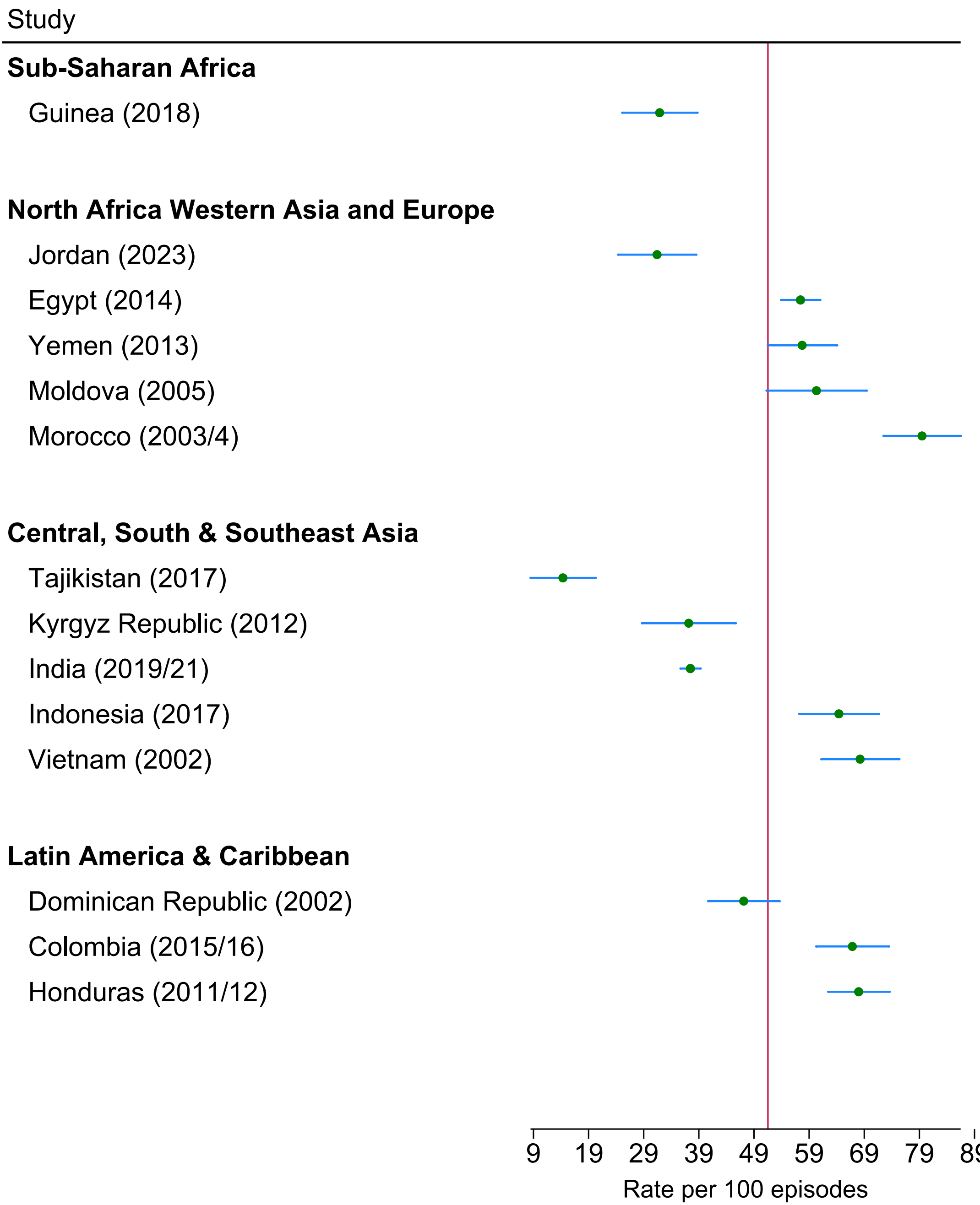

Most recent surveys since 2000

S6.3 Fig: Cumulative incidence of switching to any method within 3 months following method-related discontinuation with 95%CIs

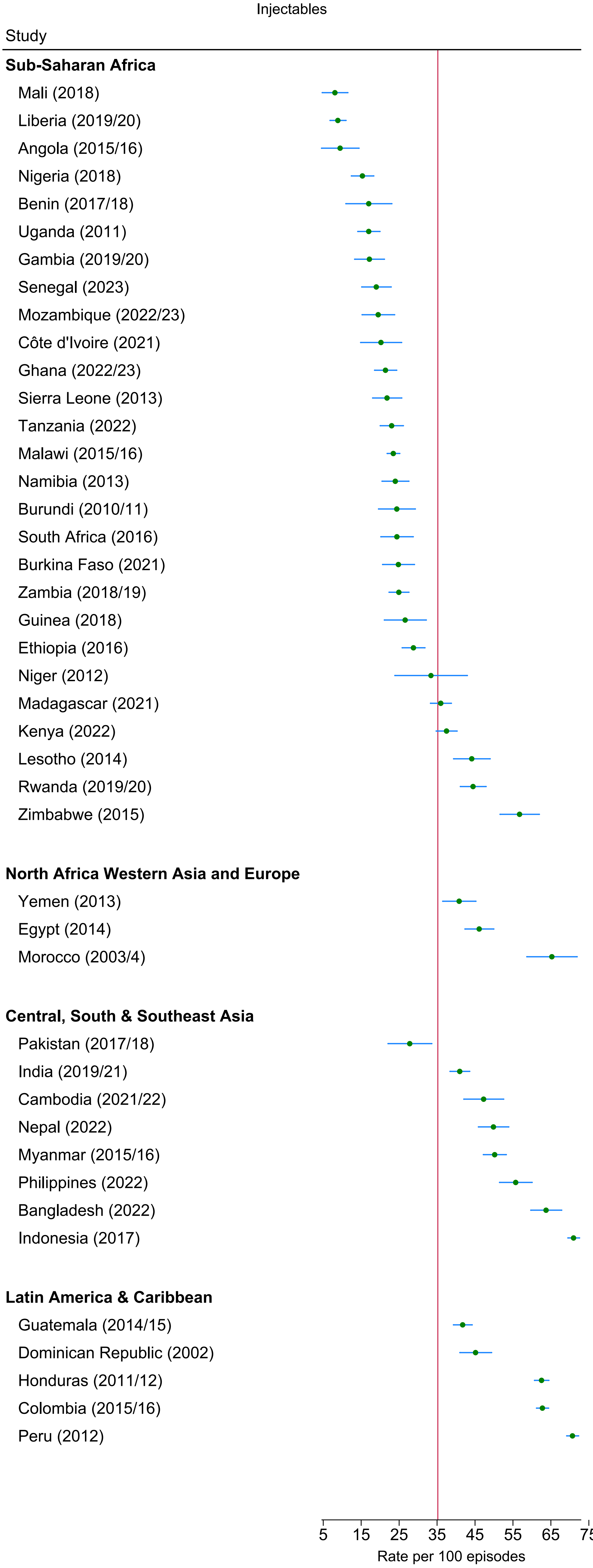

Most recent surveys since 2000

S6.4 Fig: Cumulative incidence of switching to any method within 3 months following method-related discontinuation with 95%CIs

Condom

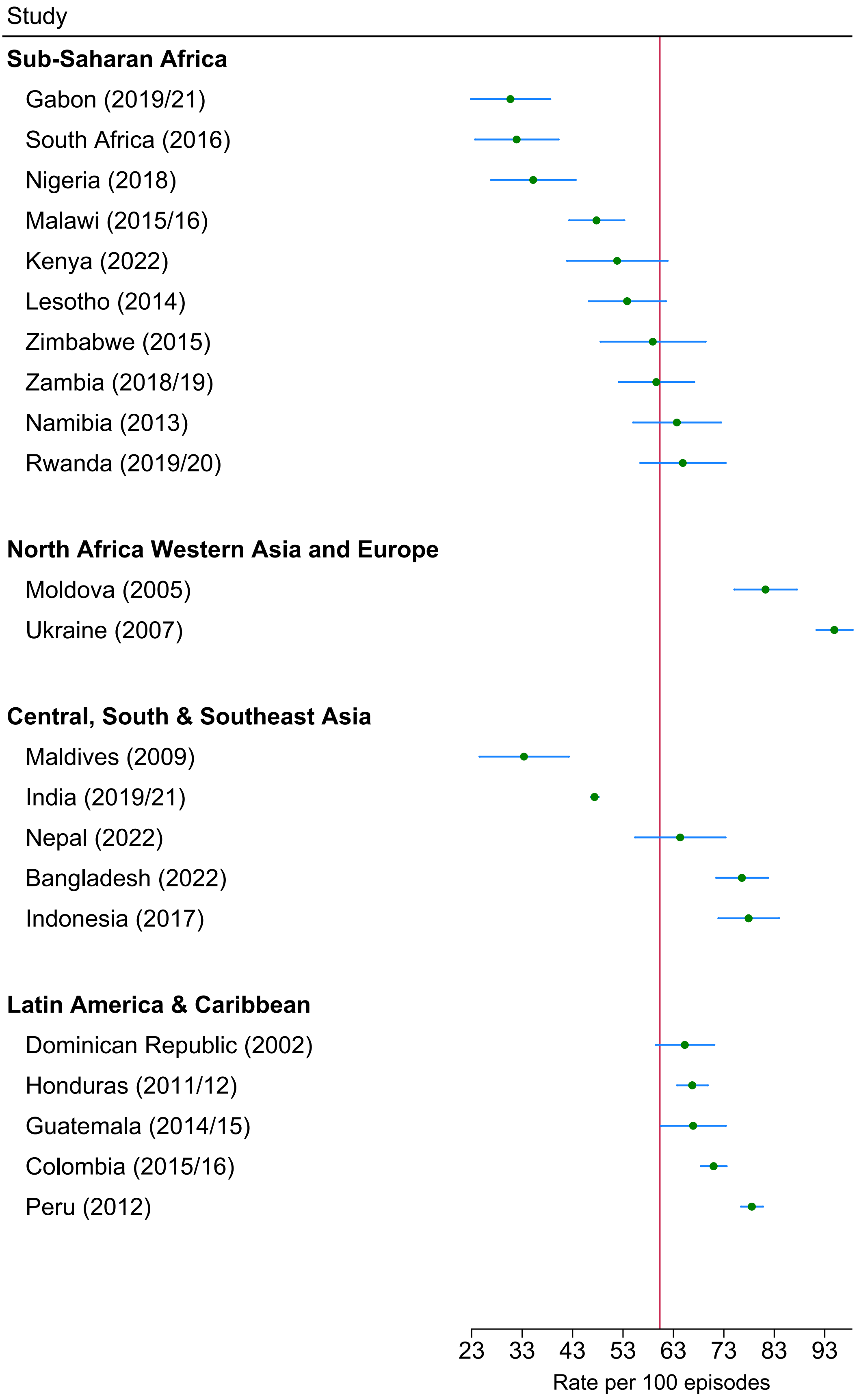

Most recent surveys since 2000

S6.5 Fig: Cumulative incidence of switching to any method within 3 months following method-related discontinuation with 95%CIs

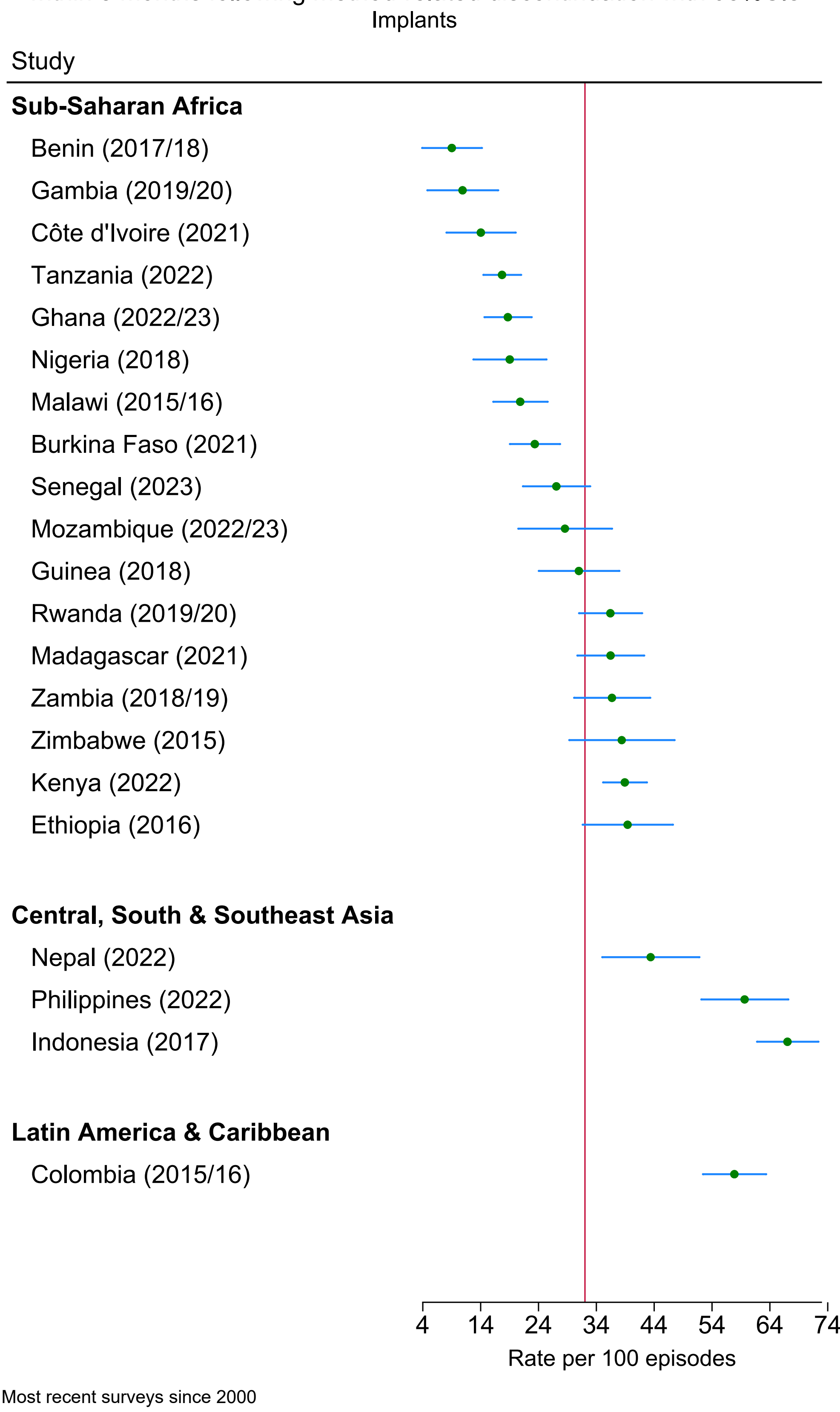

S6.6 Fig: Cumulative incidence of switching to any method within 3 months following method-related discontinuation with 95%CIs

Periodic Abstinence

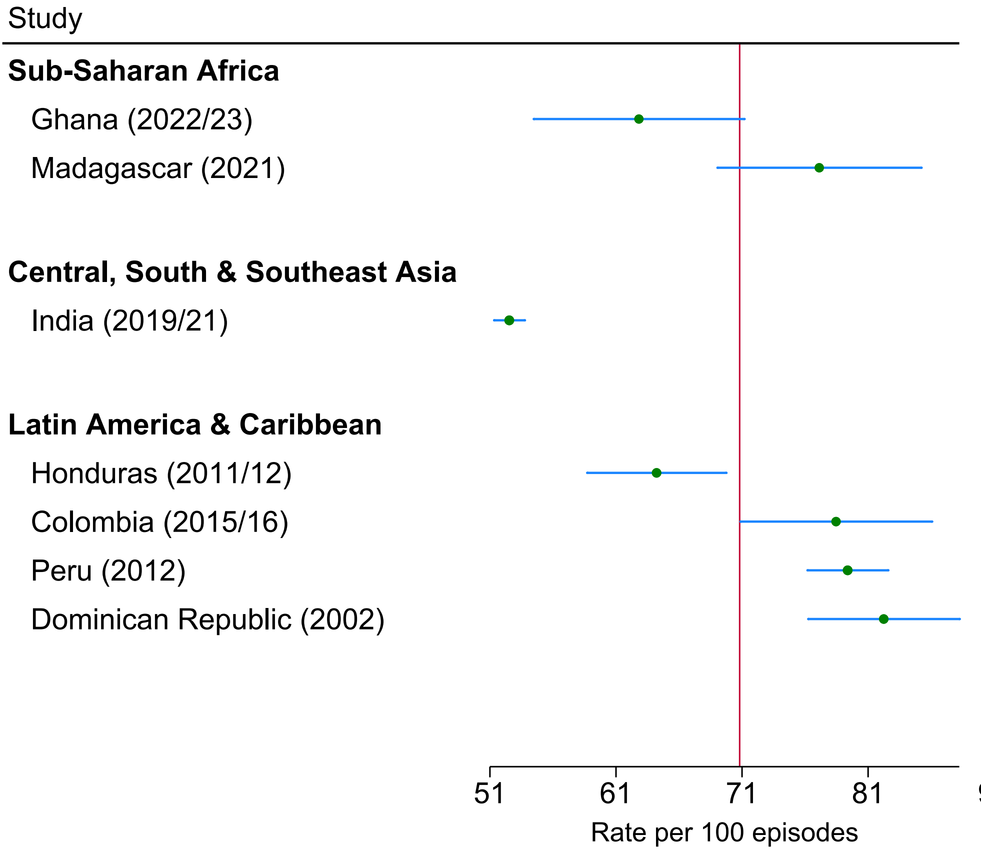

Most recent surveys since 2000

S6.7 Fig: Cumulative incidence of switching to any method within 3 months following method-related discontinuation with 95%CIs

Withdrawal

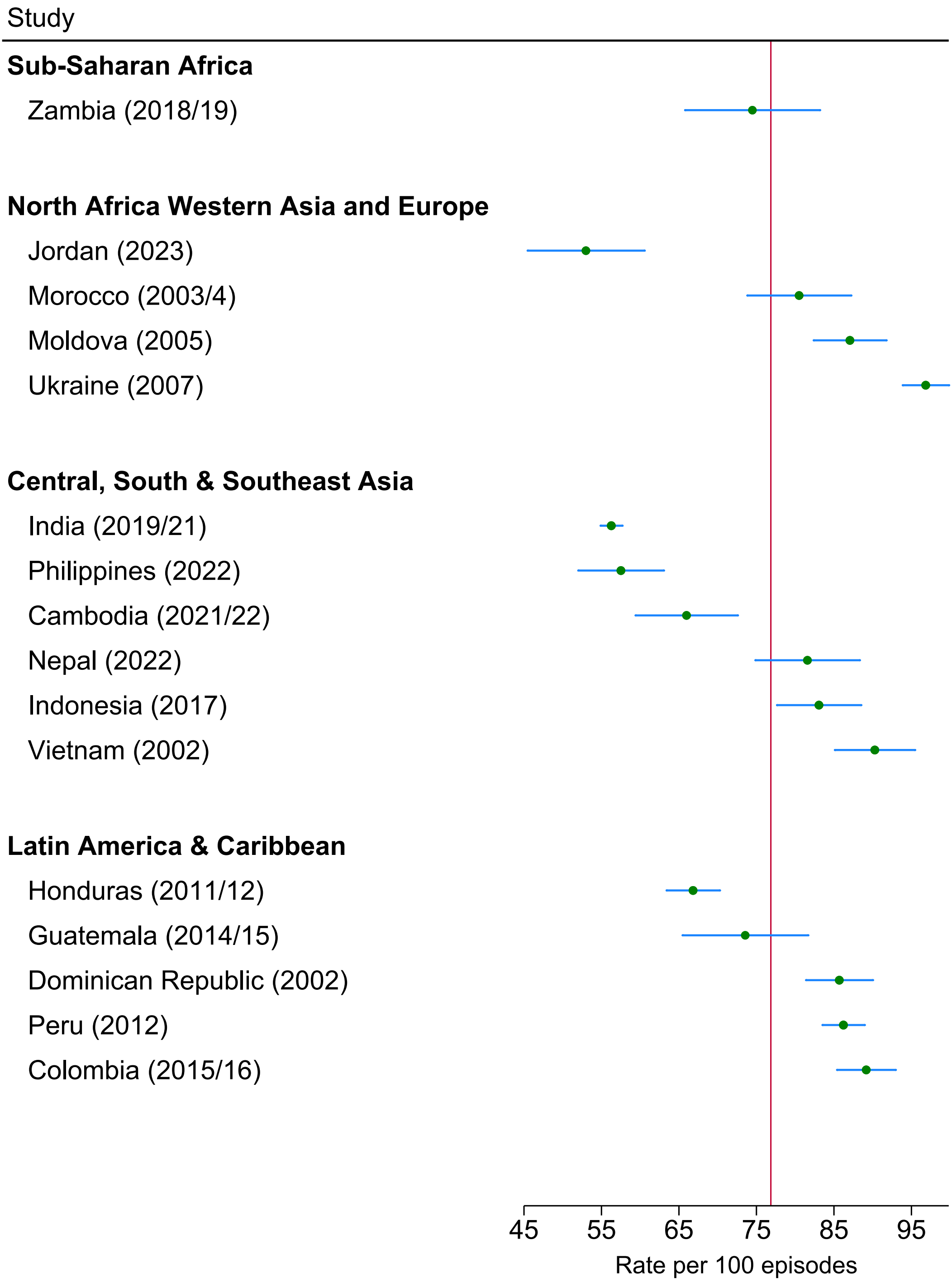

Most recent surveys since 2000
