# Supplementary material for: Contraceptive discontinuation, switching, abandonment and their reproductive consequences: An analysis of 1,539,071 episodes of reversible method use contributed from 61 countries that participated in DHS: Population base-analysis
Source: PLOS Glob Public Health. 2025 Oct 31;5(10):e0005174. doi: 10.1371/journal.pgph.0005174 (PMC12578211; doi:10.1371/journal.pgph.0005174)
Supplement: S8 Fig — (PDF) [file pgph.0005174.s009.pdf]

S8.1 Fig: Trends in 12 months method abandonment with 95%CB
Kenya: IUD

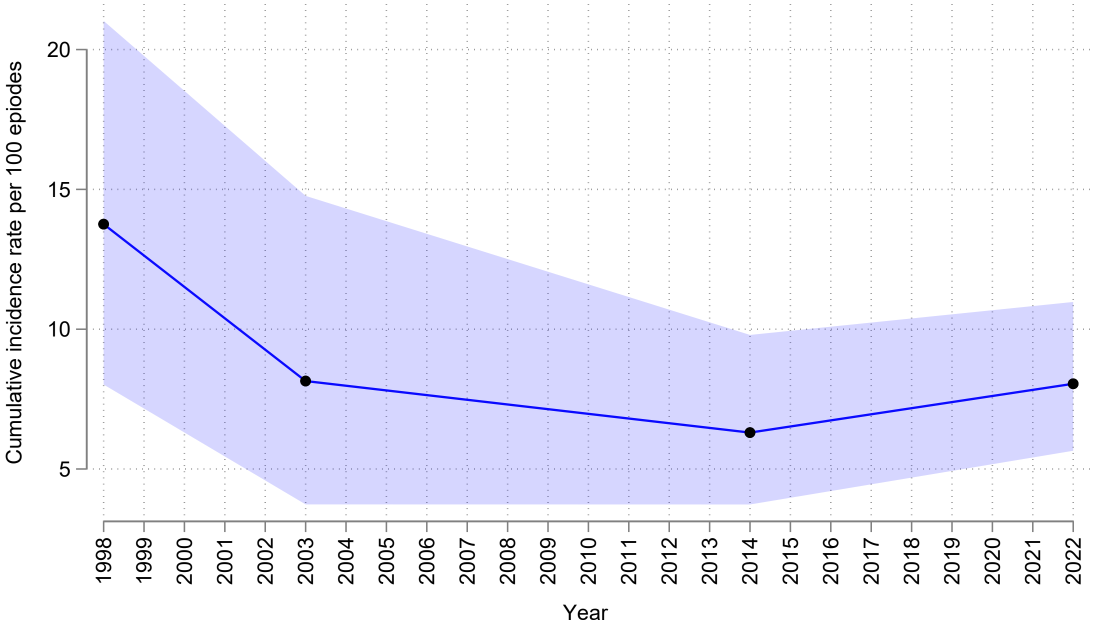

S8.2 Fig: Trends in 12 months method abandonment with 95%CB
Kenya: Injectables

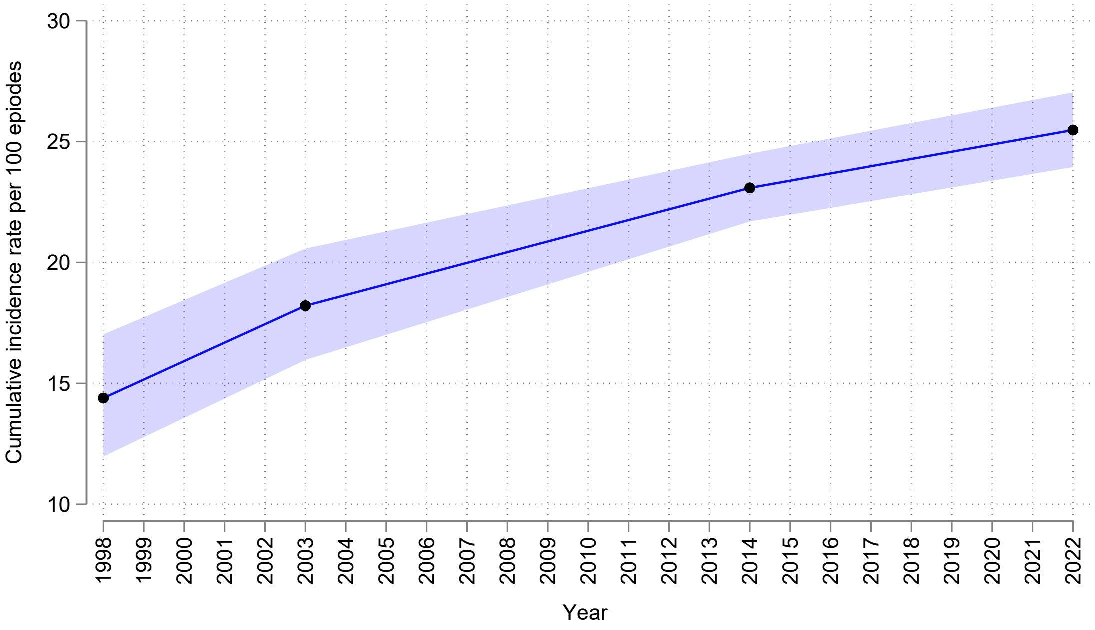

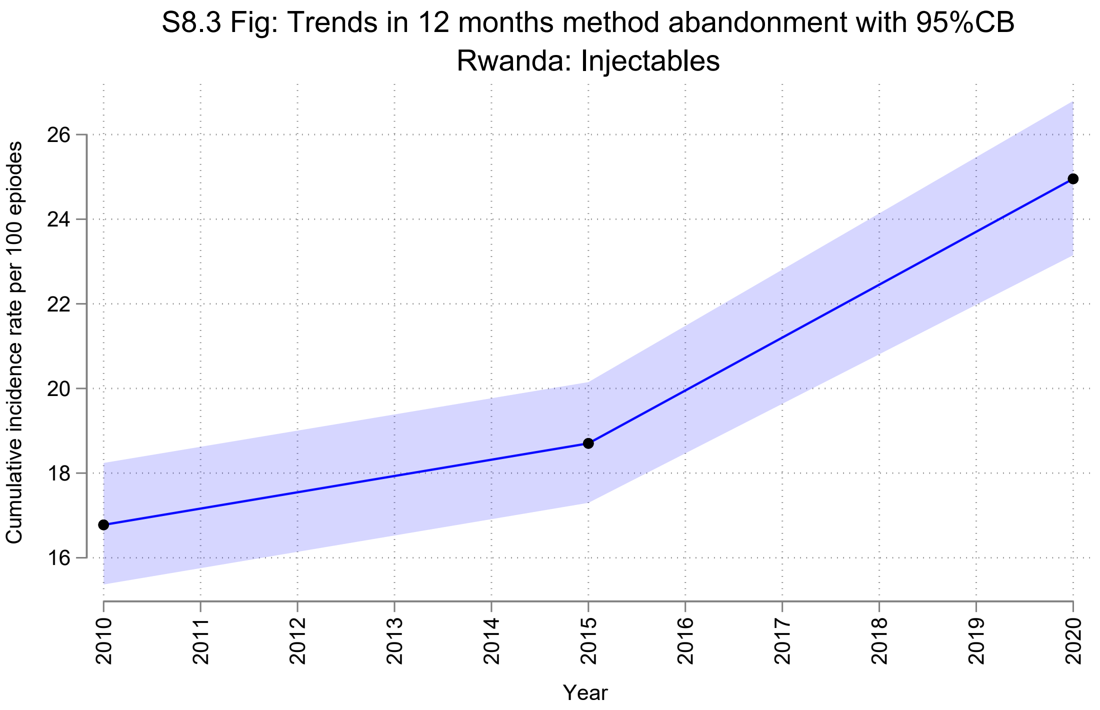
S8.3 Fig: Trends in 12 months method abandonment with 95%CB
Rwanda: Injectables
Cumulative incidence rate per 100 episodes
16
18
20
22
24
26
2010
2011
2012
2013
2014
2015
2016
2017
2018
2019
2020
Year

S8.4 Fig: Trends in 12 months method abandonment with 95%CB
Rwanda: Condom

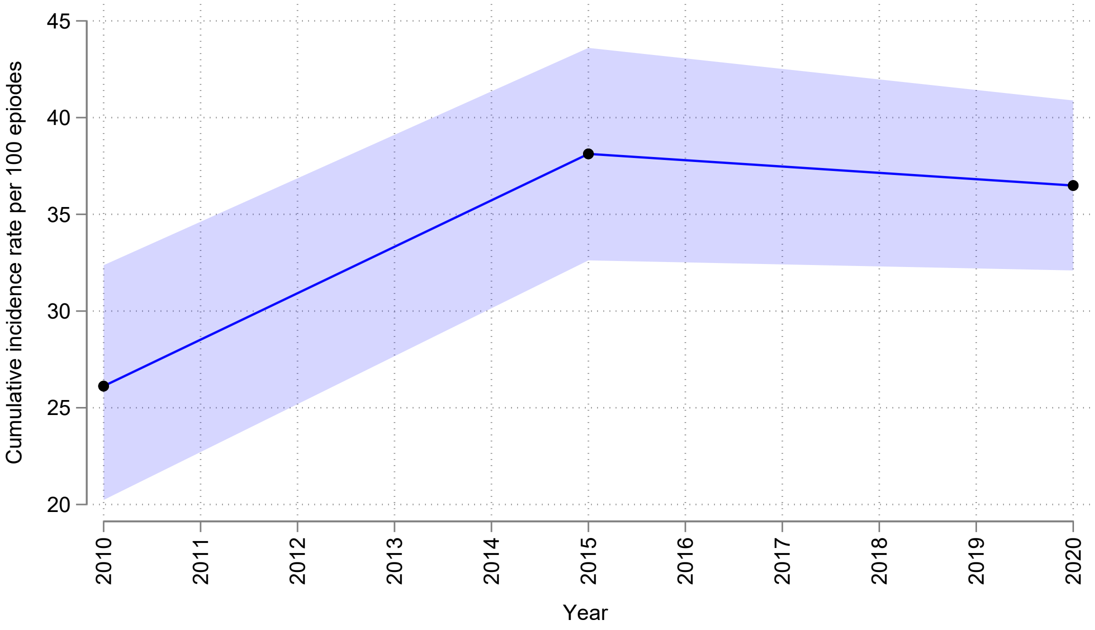

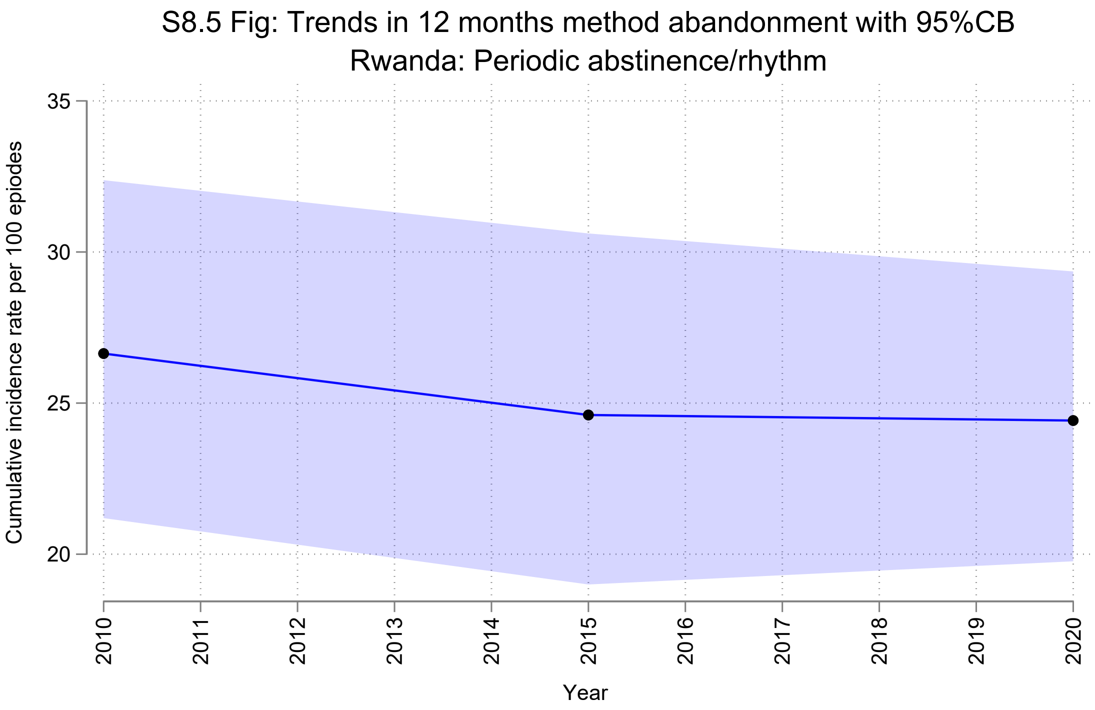

S8.5 Fig: Trends in 12 months method abandonment with 95%CB
Rwanda: Periodic abstinence/rhythm
Cumulative incidence rate per 100 epiodes
35
30
25
20
2010
2011
2012
2013
2014
2015
2016
2017
2018
2019
2020
Year

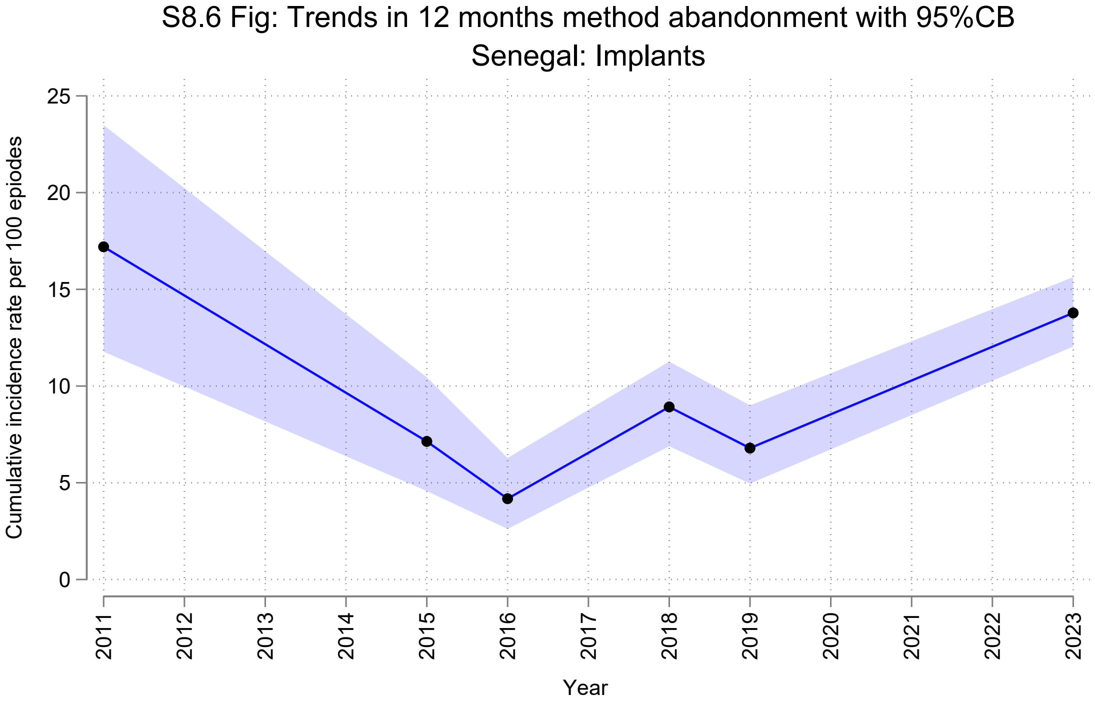

S8.6 Fig: Trends in 12 months method abandonment with 95%CB
Senegal: Implants
Cumulative incidence rate per 100 epiodes
0
5
10
15
20
25
2011
2012
2013
2014
2015
2016
2017
2018
2019
2020
2021
2022
2023
Year

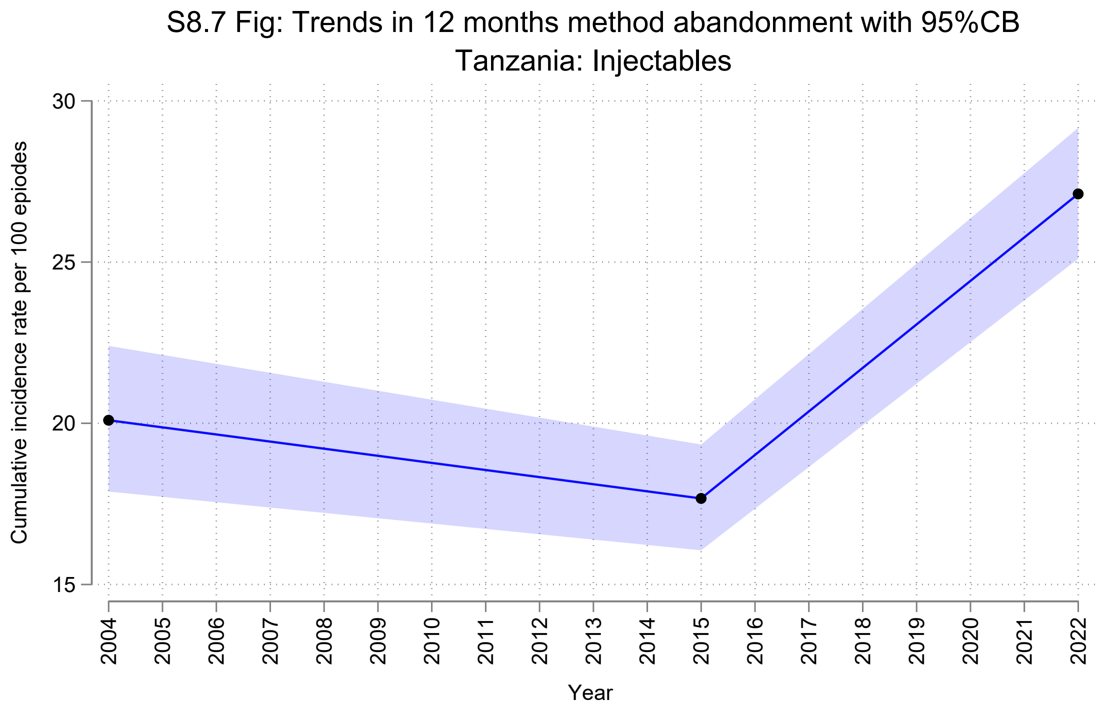

S8.7 Fig: Trends in 12 months method abandonment with 95%CB
Tanzania: Injectables
Cumulative incidence rate per 100 epiodes
30
25
20
15
2004
2005
2006
2007
2008
2009
2010
2011
2012
2013
2014
2015
2016
2017
2018
2019
2020
2021
2022
Year

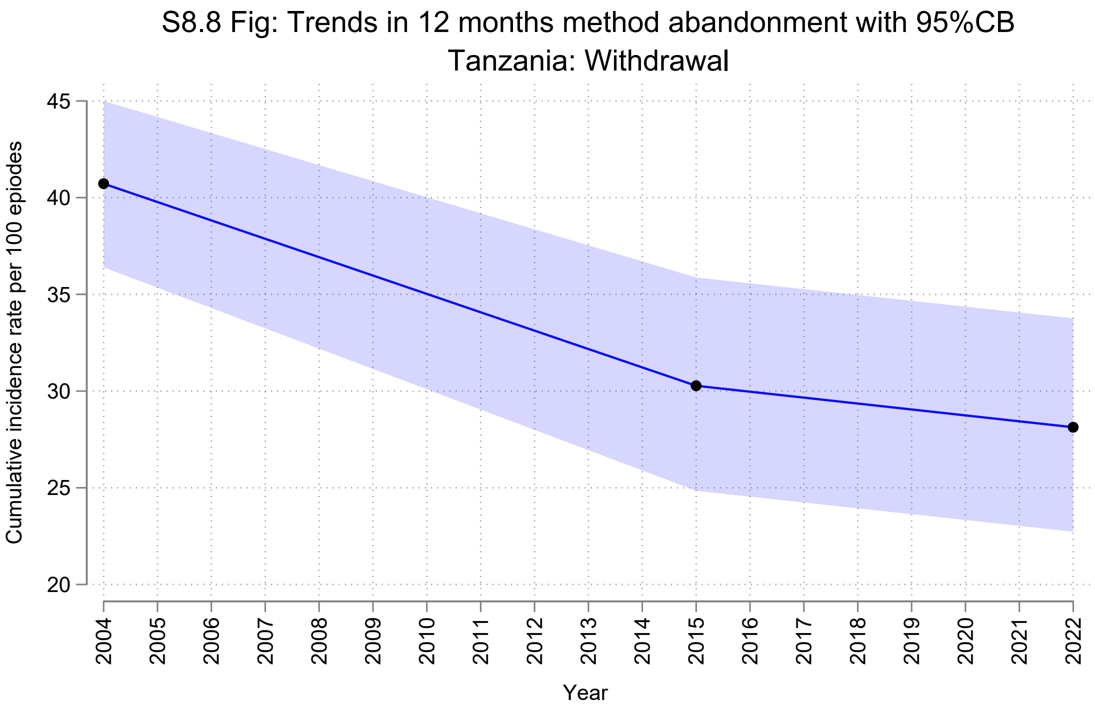
S8.8 Fig: Trends in 12 months method abandonment with 95%CB
Tanzania: Withdrawal
Cumulative incidence rate per 100 epiodes
45
40
35
30
25
20
2004
2005
2006
2007
2008
2009
2010
2011
2012
2013
2014
2015
2016
2017
2018
2019
2020
2021
2022
Year

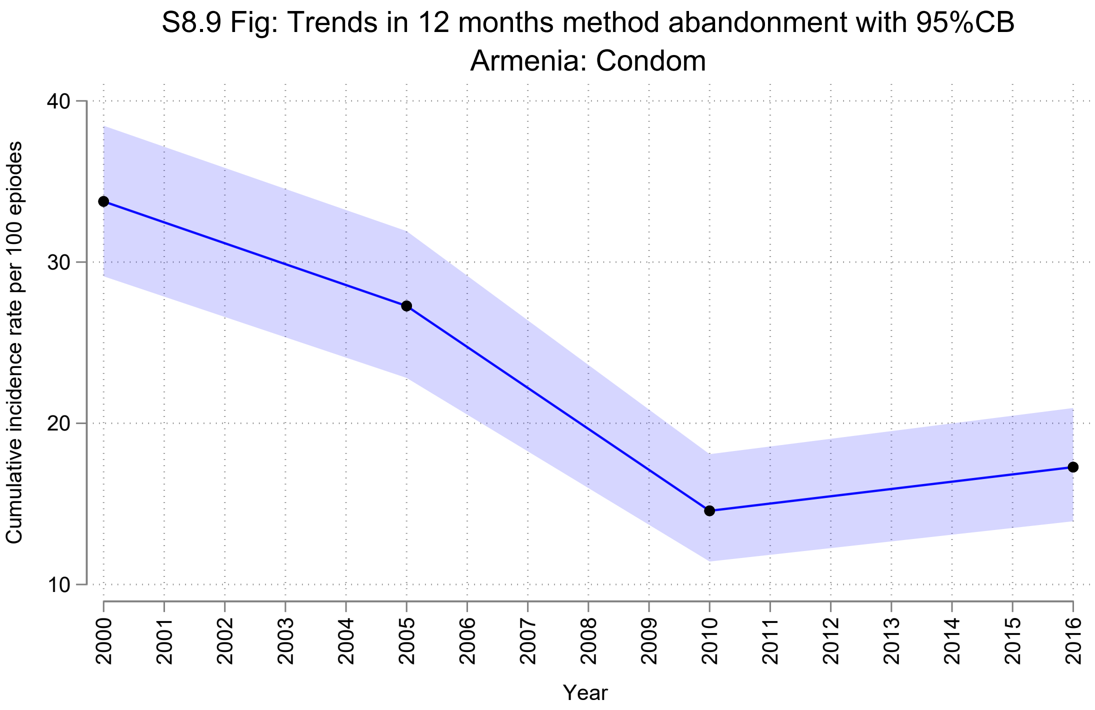
S8.9 Fig: Trends in 12 months method abandonment with 95%CB
Armenia: Condom
Cumulative incidence rate per 100 epiodes
40
30
20
10
2000
2001
2002
2003
2004
2005
2006
2007
2008
2009
2010
2011
2012
2013
2014
2015
2016
Year

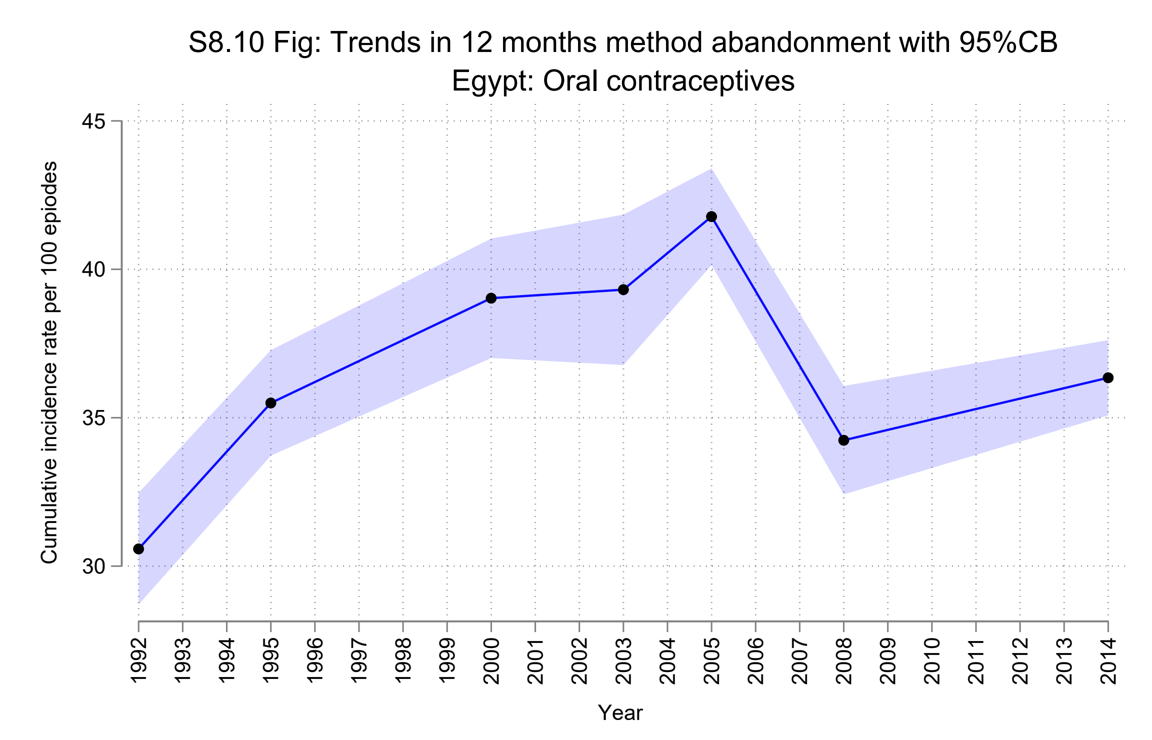
S8.10 Fig: Trends in 12 months method abandonment with 95%CB
Egypt: Oral contraceptives
Cumulative incidence rate per 100 epiodes
45
40
35
30
1992
1993
1994
1995
1996
1997
1998
1999
2000
2001
2002
2003
2004
2005
2006
2007
2008
2009
2010
2011
2012
2013
2014
Year

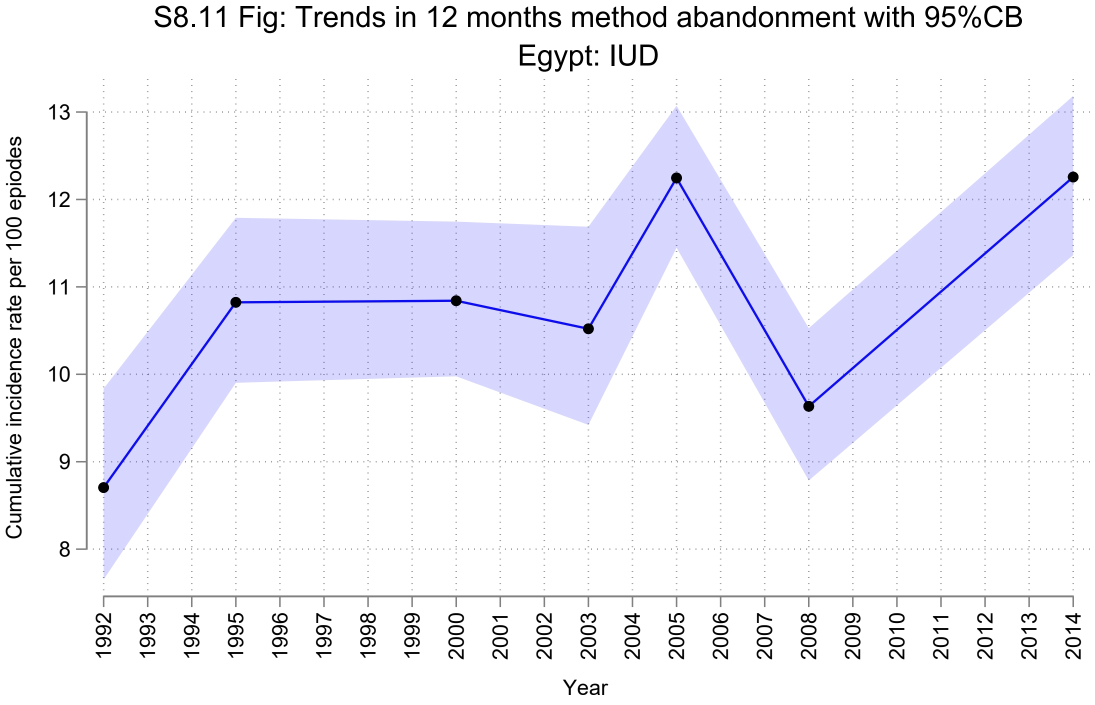
S8.11 Fig: Trends in 12 months method abandonment with 95%CB
Egypt: IUD
Cumulative incidence rate per 100 episodes
13
12
11
10
9
8
1992
1993
1994
1995
1996
1997
1998
1999
2000
2001
2002
2003
2004
2005
2006
2007
2008
2009
2010
2011
2012
2013
2014
Year

S8.12 Fig: Trends in 12 months method abandonment with 95%CB
Egypt: Injectables

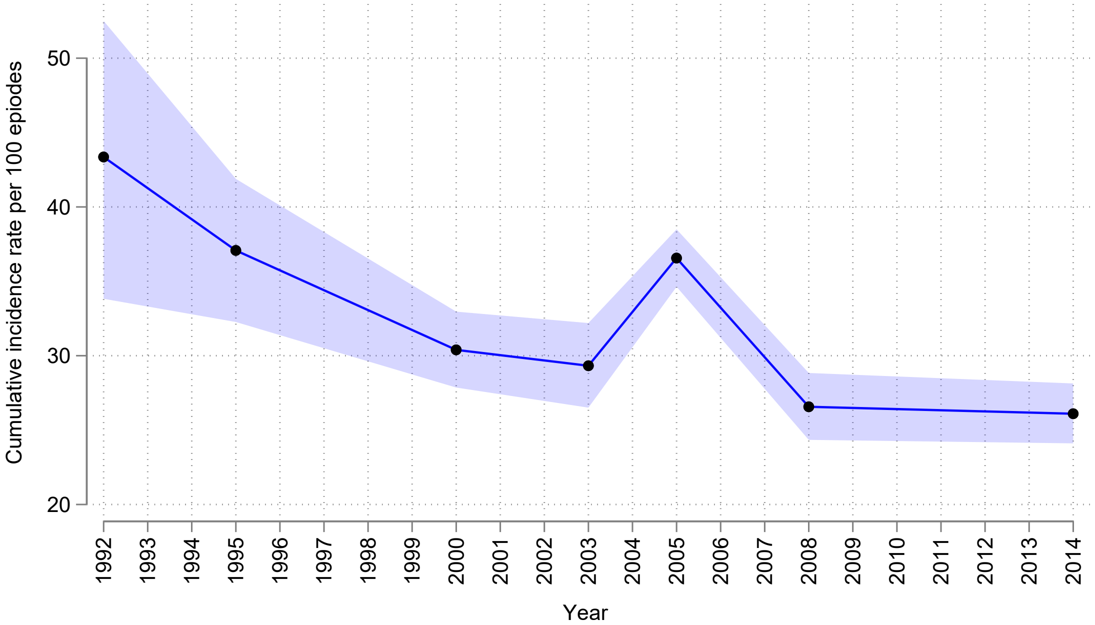

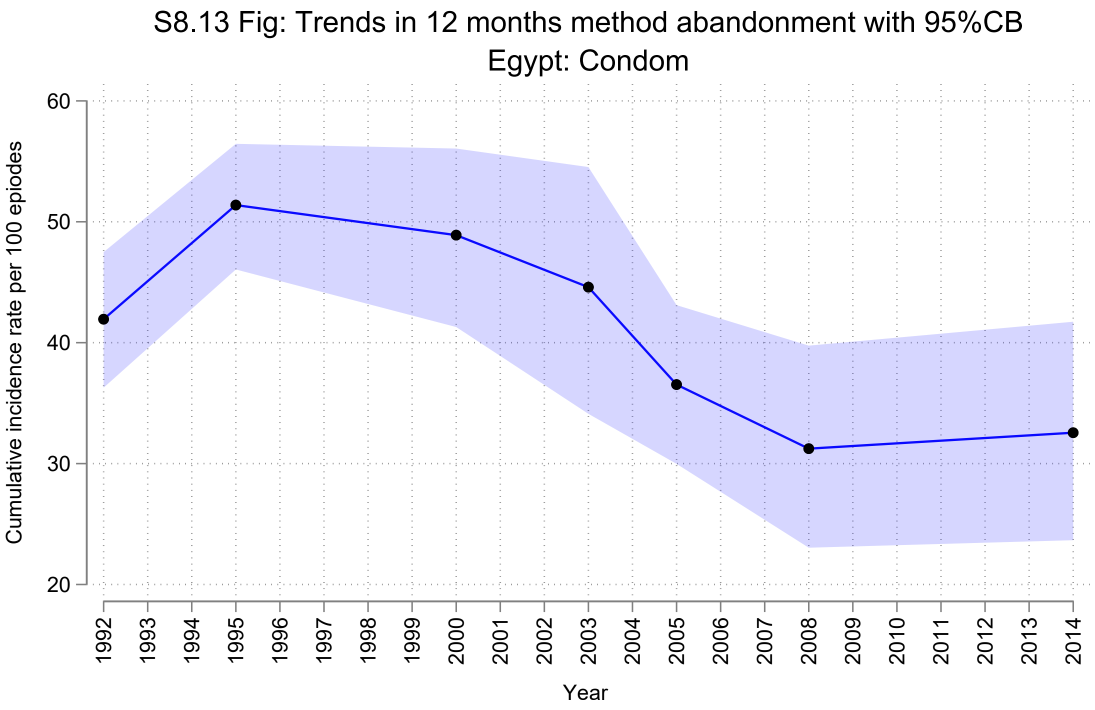
S8.13 Fig: Trends in 12 months method abandonment with 95%CB
Egypt: Condom
Cumulative incidence rate per 100 episodes
60
50
40
30
20
1992
1993
1994
1995
1996
1997
1998
1999
2000
2001
2002
2003
2004
2005
2006
2007
2008
2009
2010
2011
2012
2013
2014
Year

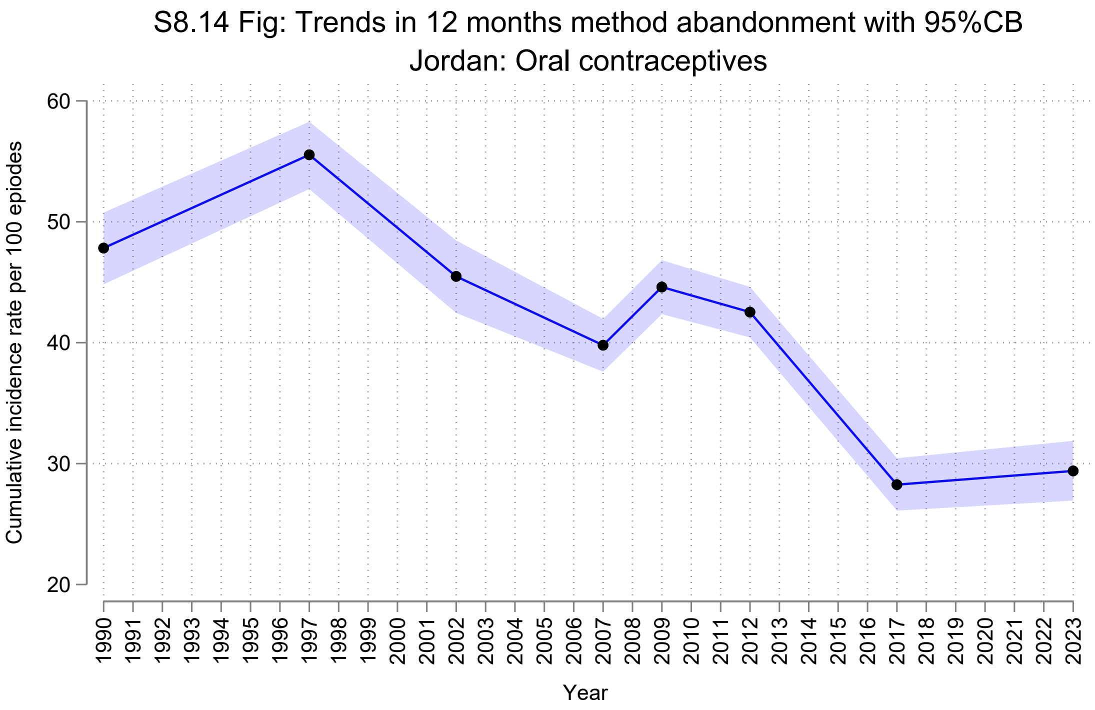
S8.14 Fig: Trends in 12 months method abandonment with 95%CB
Jordan: Oral contraceptives
Cumulative incidence rate per 100 epiodes
60
50
40
30
20
1990
1991
1992
1993
1994
1995
1996
1997
1998
1999
2000
2001
2002
2003
2004
2005
2006
2007
2008
2009
2010
2011
2012
2013
2014
2015
2016
2017
2018
2019
2020
2021
2022
2023
Year

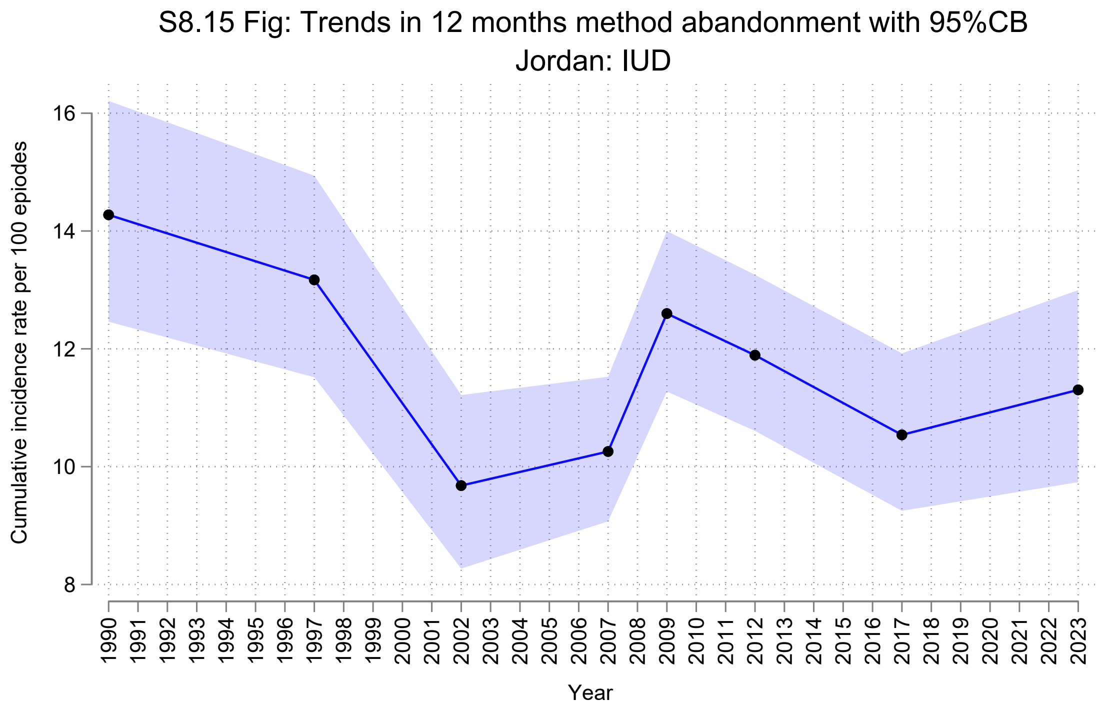
S8.15 Fig: Trends in 12 months method abandonment with 95%CB
Jordan: IUD
Cumulative incidence rate per 100 epiodes
16
14
12
10
8
1990 1991 1992 1993 1994 1995 1996 1997 1998 1999 2000 2001 2002 2003 2004 2005 2006 2007 2008 2009 2010 2011 2012 2013 2014 2015 2016 2017 2018 2019 2020 2021 2022 2023
Year

S8.16 Fig: Trends in 12 months method abandonment with 95%CB
Bangladesh: Oral contraceptives

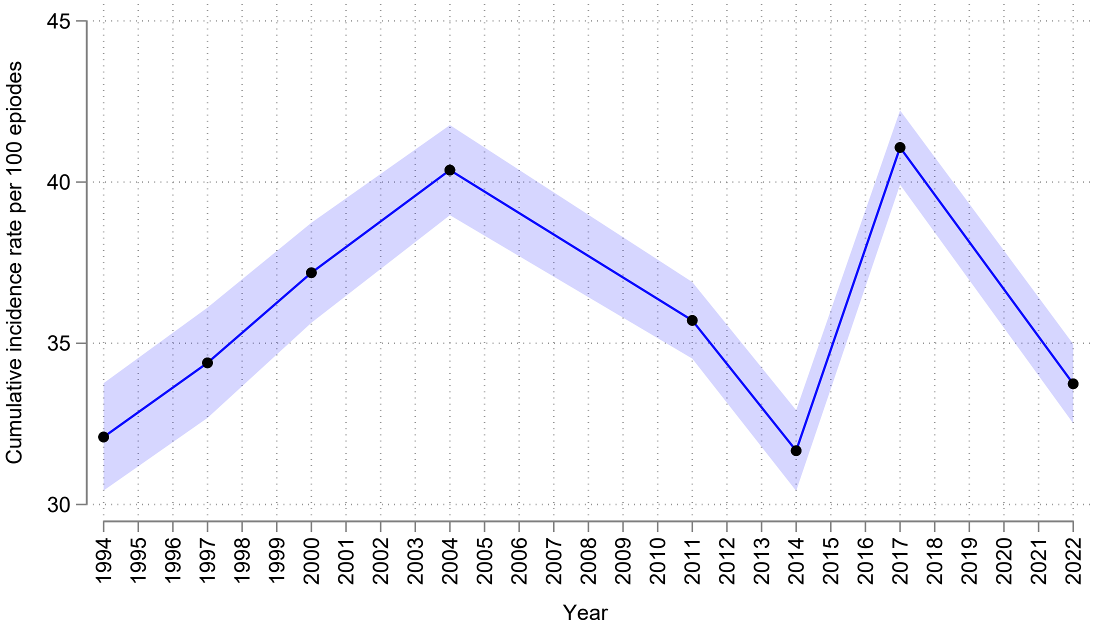

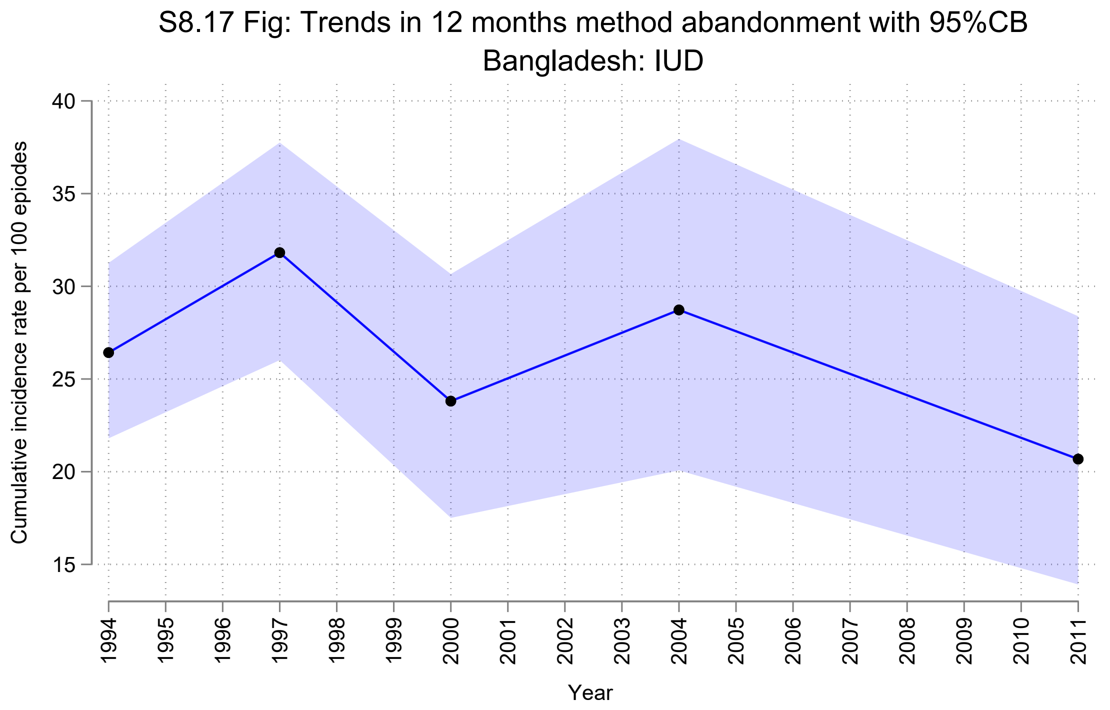

S8.17 Fig: Trends in 12 months method abandonment with 95%CB
Bangladesh: IUD
Cumulative incidence rate per 100 epiodes
40
35
30
25
20
15
1994
1995
1996
1997
1998
1999
2000
2001
2002
2003
2004
2005
2006
2007
2008
2009
2010
2011
Year

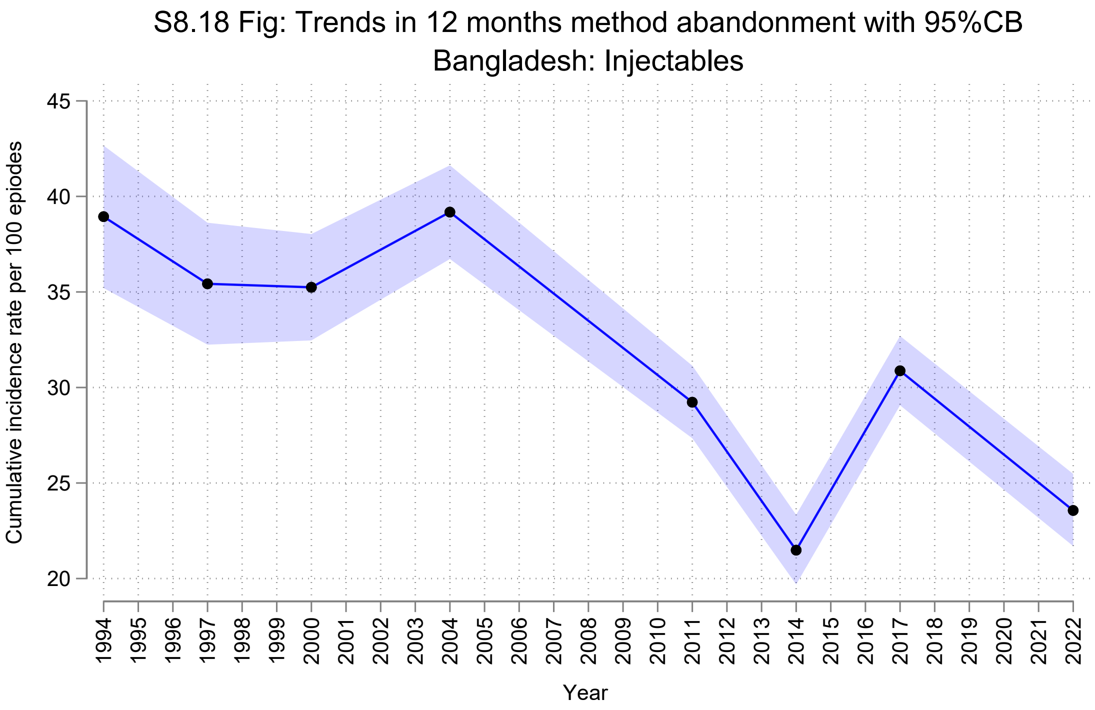

S8.18 Fig: Trends in 12 months method abandonment with 95%CB
Bangladesh: Injectables
Cumulative incidence rate per 100 epiodes
45
40
35
30
25
20
1994
1995
1996
1997
1998
1999
2000
2001
2002
2003
2004
2005
2006
2007
2008
2009
2010
2011
2012
2013
2014
2015
2016
2017
2018
2019
2020
2021
2022
Year

S8.19 Fig: Trends in 12 months method abandonment with 95%CB
Bangladesh: Condom

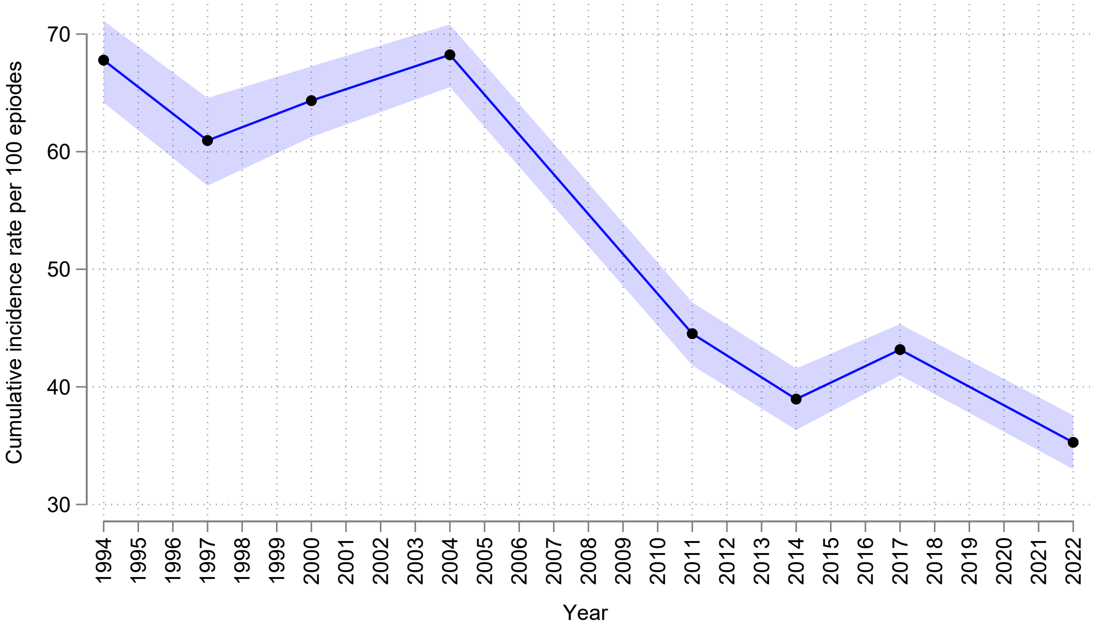

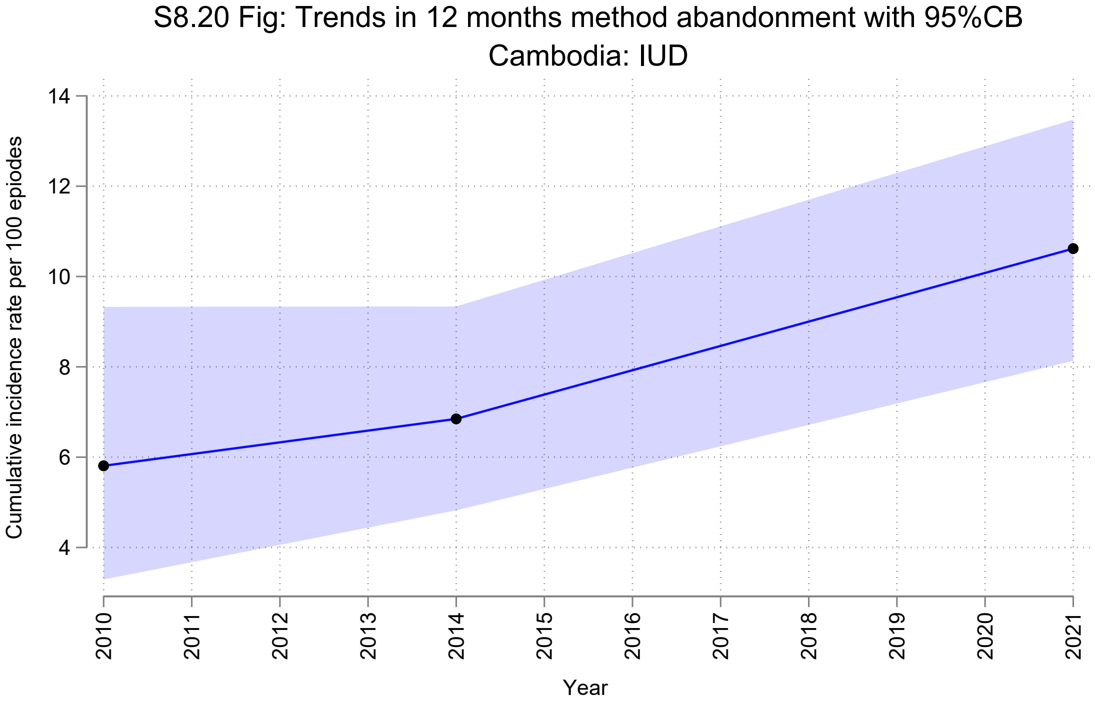
S8.20 Fig: Trends in 12 months method abandonment with 95%CB
Cambodia: IUD
Cumulative incidence rate per 100 epiodes
14
12
10
8
6
4
2010
2011
2012
2013
2014
2015
2016
2017
2018
2019
2020
2021
Year

S8.21 Fig: Trends in 12 months method abandonment with 95%CB
Cambodia: Injectables

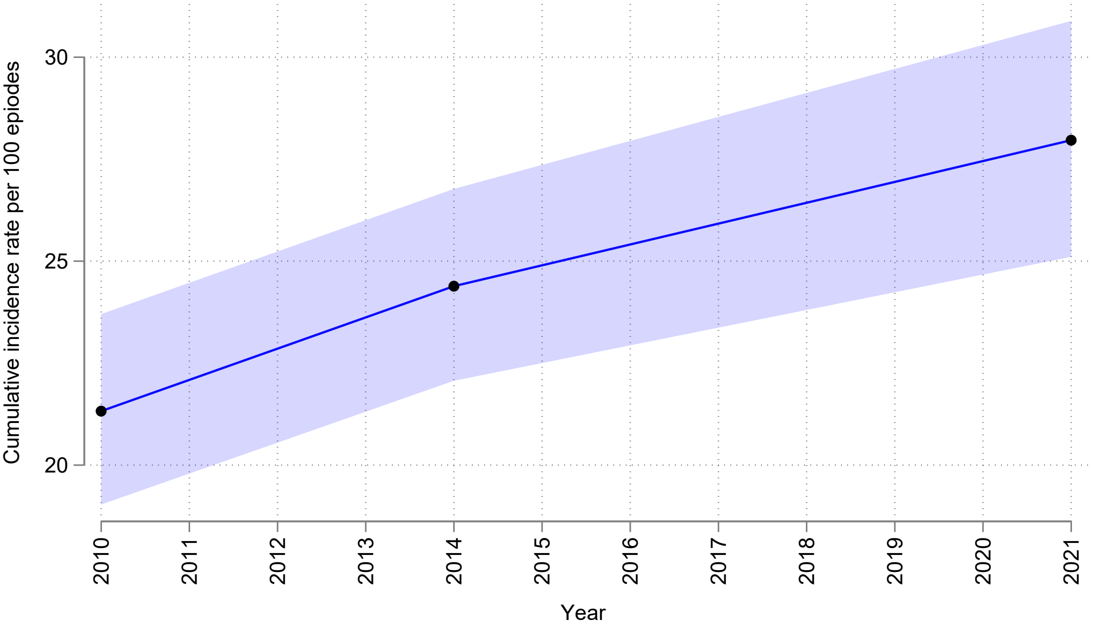

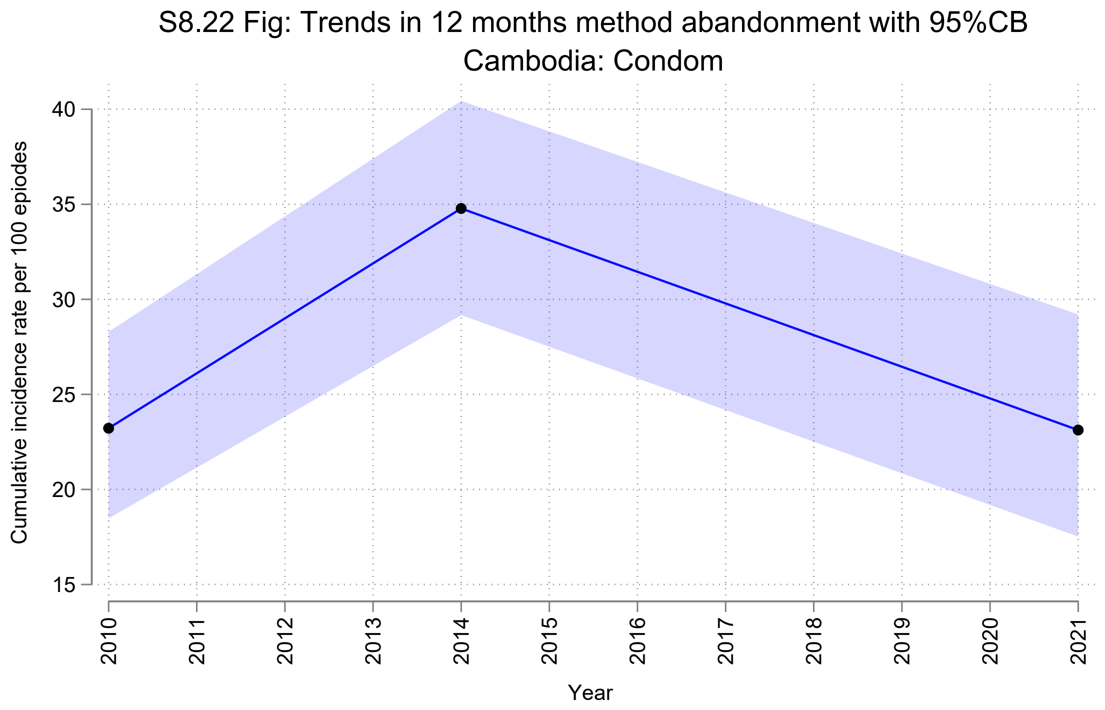
S8.22 Fig: Trends in 12 months method abandonment with 95%CB
Cambodia: Condom
Cumulative incidence rate per 100 episodes
15
20
25
30
35
40
2010
2011
2012
2013
2014
2015
2016
2017
2018
2019
2020
2021
Year

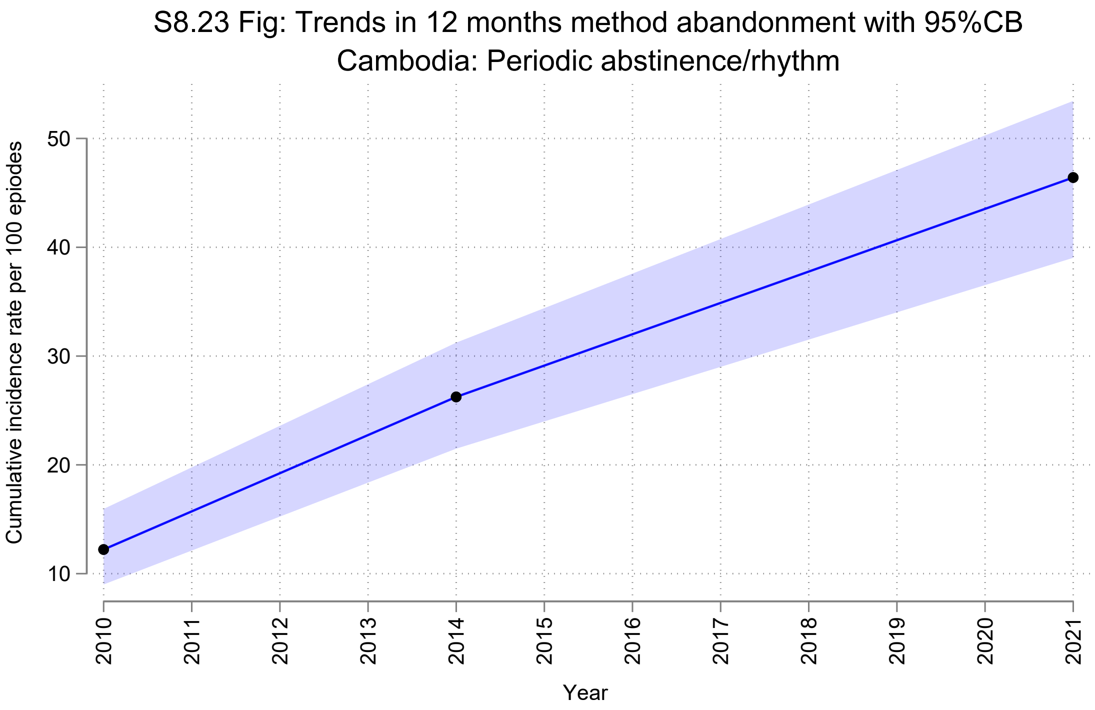
S8.23 Fig: Trends in 12 months method abandonment with 95%CB
Cambodia: Periodic abstinence/rhythm
Cumulative incidence rate per 100 epiodes
50
40
30
20
10
2010
2011
2012
2013
2014
2015
2016
2017
2018
2019
2020
2021
Year

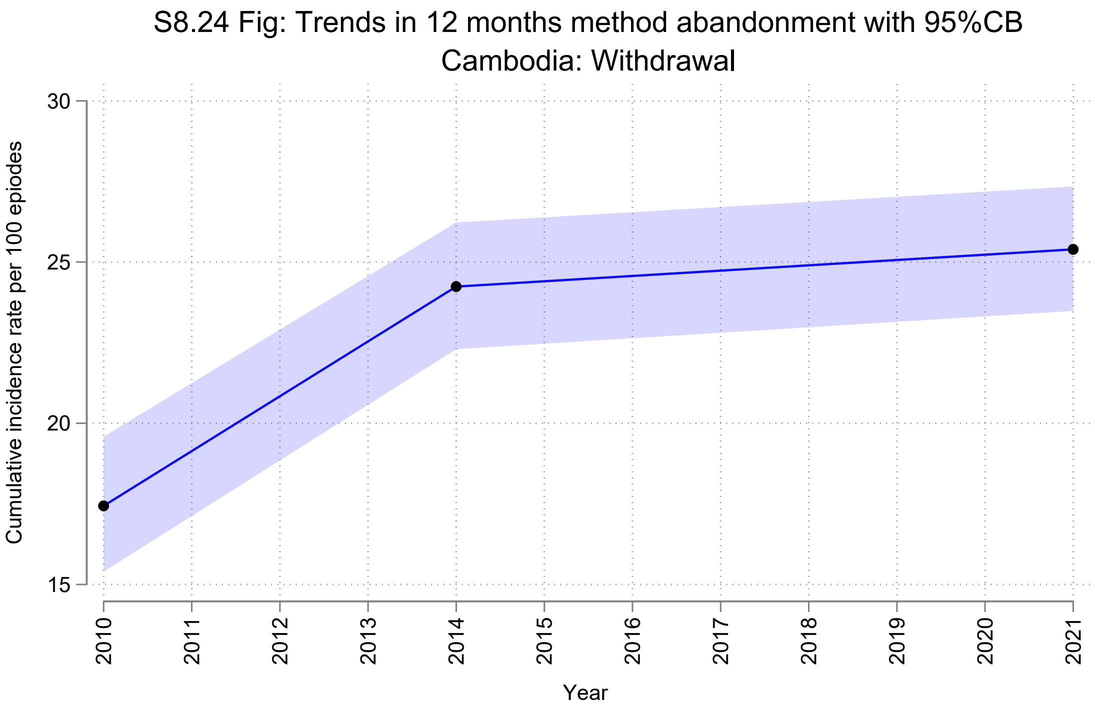
S8.24 Fig: Trends in 12 months method abandonment with 95%CB
Cambodia: Withdrawal
Cumulative incidence rate per 100 epiodes
30
25
20
15
2010
2011
2012
2013
2014
2015
2016
2017
2018
2019
2020
2021
Year

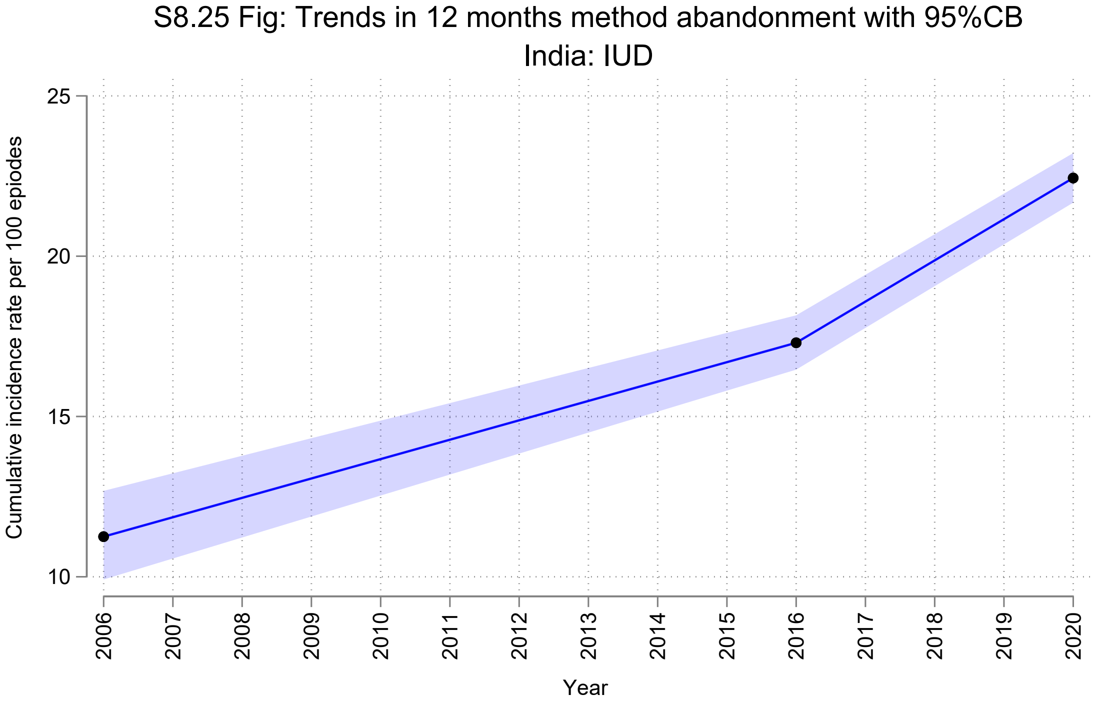
S8.25 Fig: Trends in 12 months method abandonment with 95%CB
India: IUD
Cumulative incidence rate per 100 epiodes
25
20
15
10
2006
2007
2008
2009
2010
2011
2012
2013
2014
2015
2016
2017
2018
2019
2020
Year

S8.26 Fig: Trends in 12 months method abandonment with 95%CB
India: Condom

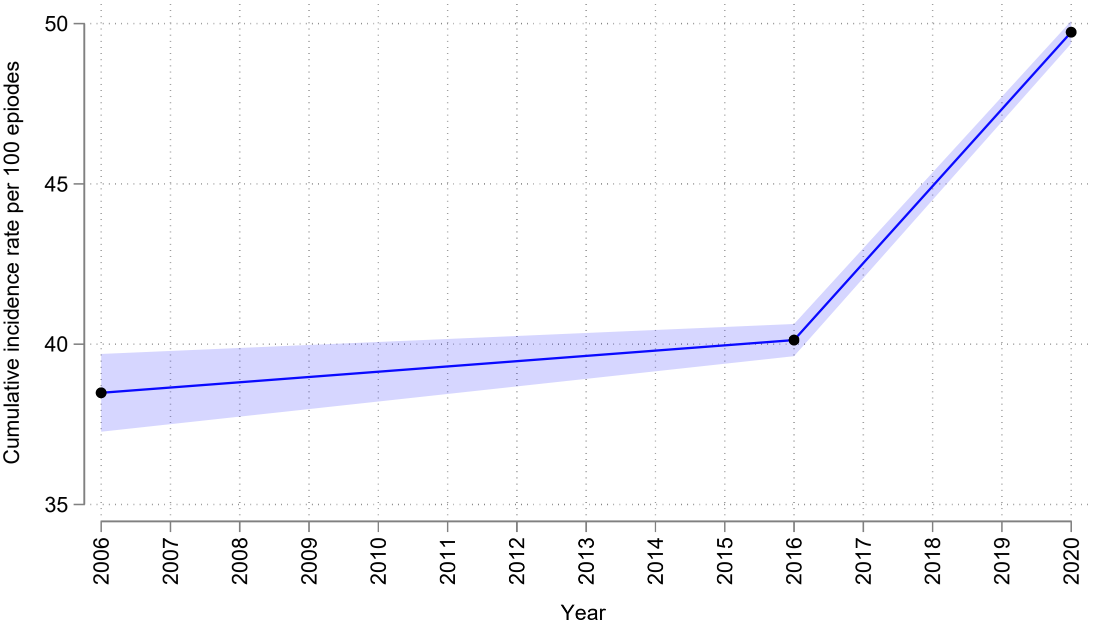

S8.27 Fig: Trends in 12 months method abandonment with 95%CB
India: Periodic abstinence/rhythm

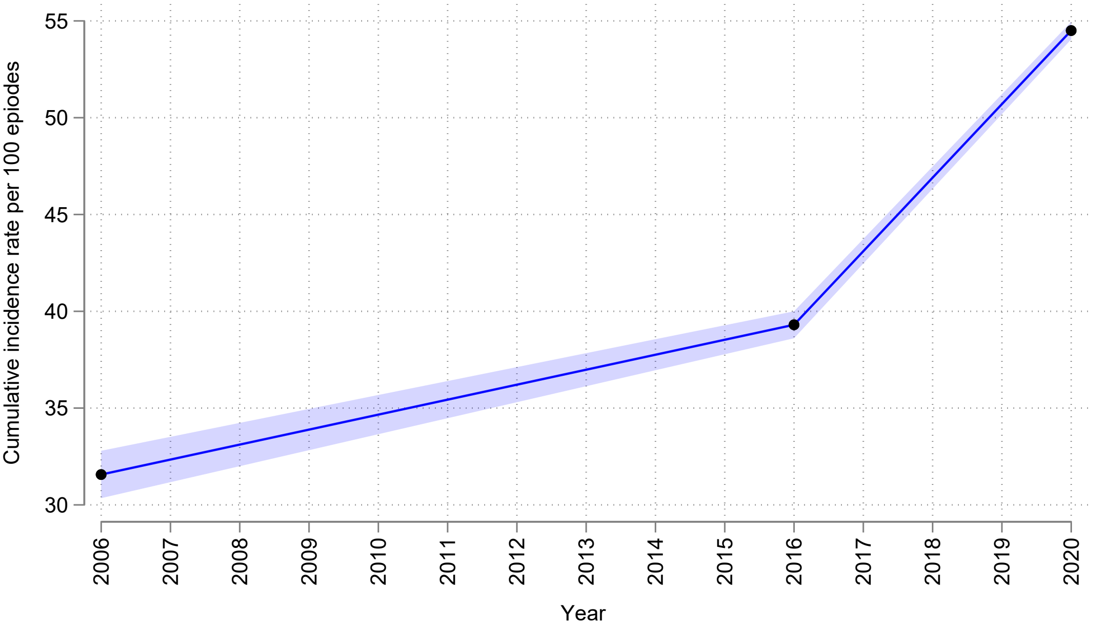

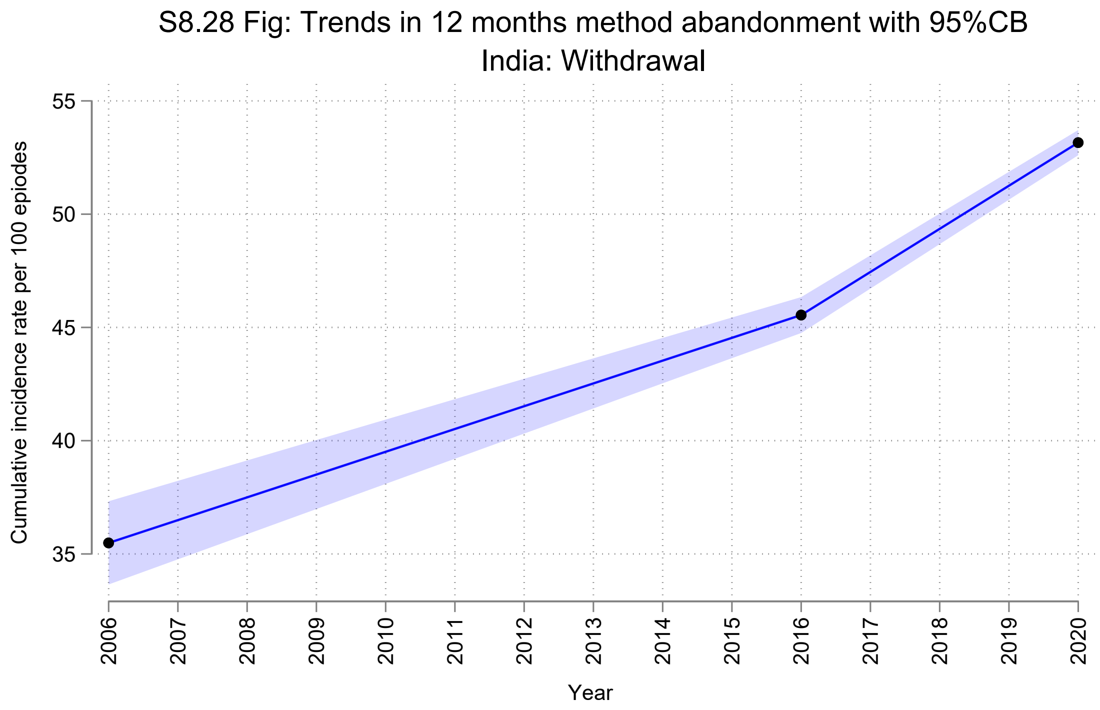
S8.28 Fig: Trends in 12 months method abandonment with 95%CB
India: Withdrawal
Cumulative incidence rate per 100 epiodes
55
50
45
40
35
2006
2007
2008
2009
2010
2011
2012
2013
2014
2015
2016
2017
2018
2019
2020
Year

S8.29 Fig: Trends in 12 months method abandonment with 95%CB
Indonesia: Oral contraceptives

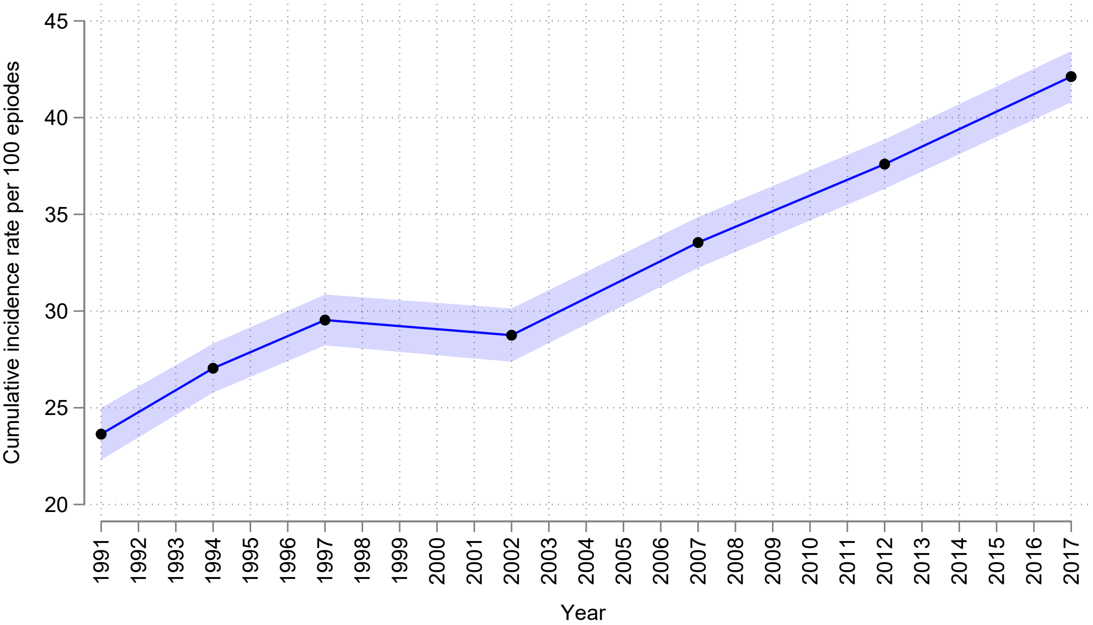

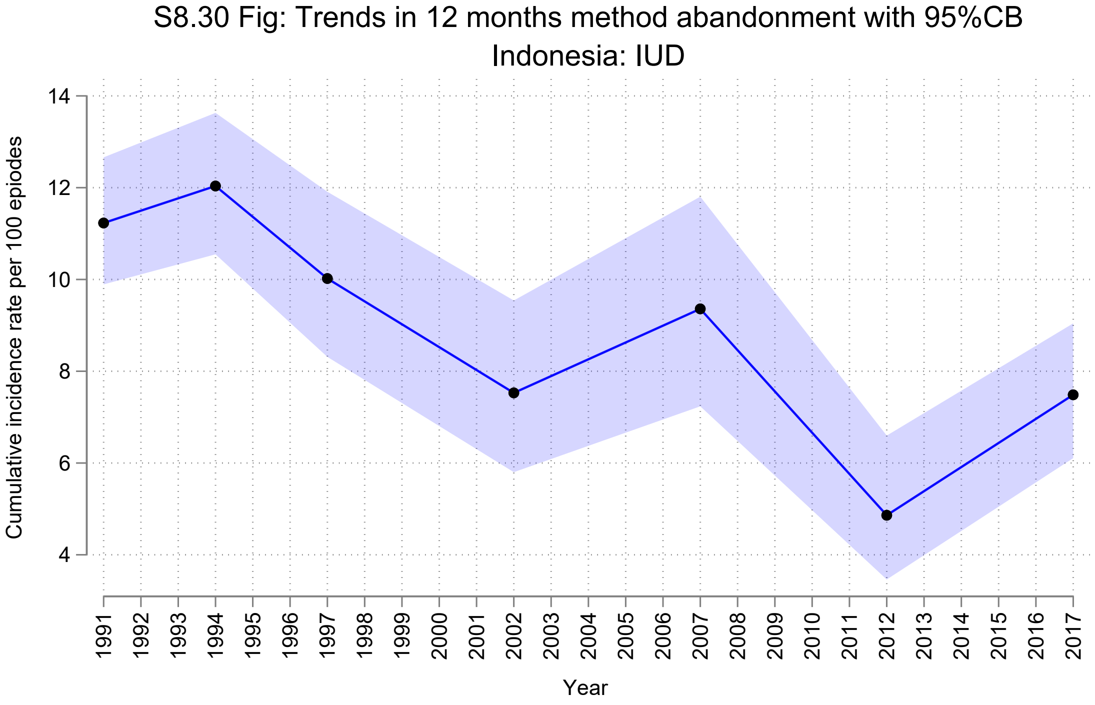
S8.30 Fig: Trends in 12 months method abandonment with 95%CB
Indonesia: IUD
Cumulative incidence rate per 100 epiodes
14
12
10
8
6
4
1991
1992
1993
1994
1995
1996
1997
1998
1999
2000
2001
2002
2003
2004
2005
2006
2007
2008
2009
2010
2011
2012
2013
2014
2015
2016
2017
Year

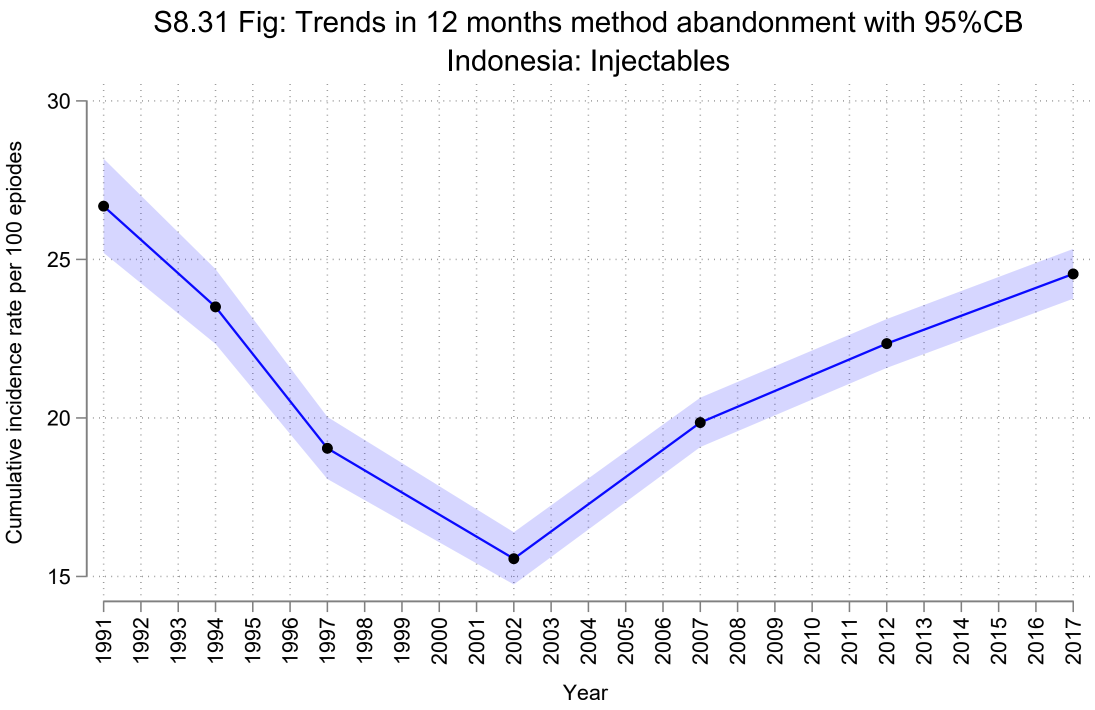
S8.31 Fig: Trends in 12 months method abandonment with 95%CB
Indonesia: Injectables
Cumulative incidence rate per 100 epiodes
30
25
20
15
1991
1992
1993
1994
1995
1996
1997
1998
1999
2000
2001
2002
2003
2004
2005
2006
2007
2008
2009
2010
2011
2012
2013
2014
2015
2016
2017
Year

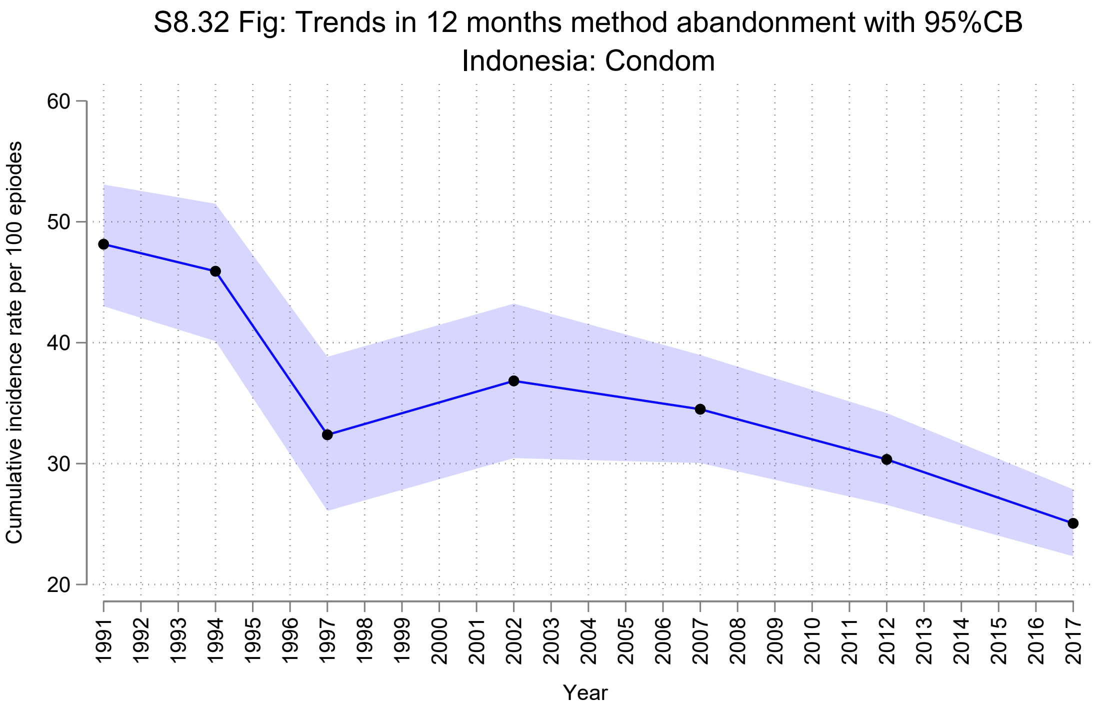

S8.32 Fig: Trends in 12 months method abandonment with 95%CB
Indonesia: Condom
Cumulative incidence rate per 100 epiodes
60
50
40
30
20
1991
1992
1993
1994
1995
1996
1997
1998
1999
2000
2001
2002
2003
2004
2005
2006
2007
2008
2009
2010
2011
2012
2013
2014
2015
2016
2017
Year

S8.33 Fig: Trends in 12 months method abandonment with 95%CB
Indonesia: Implants

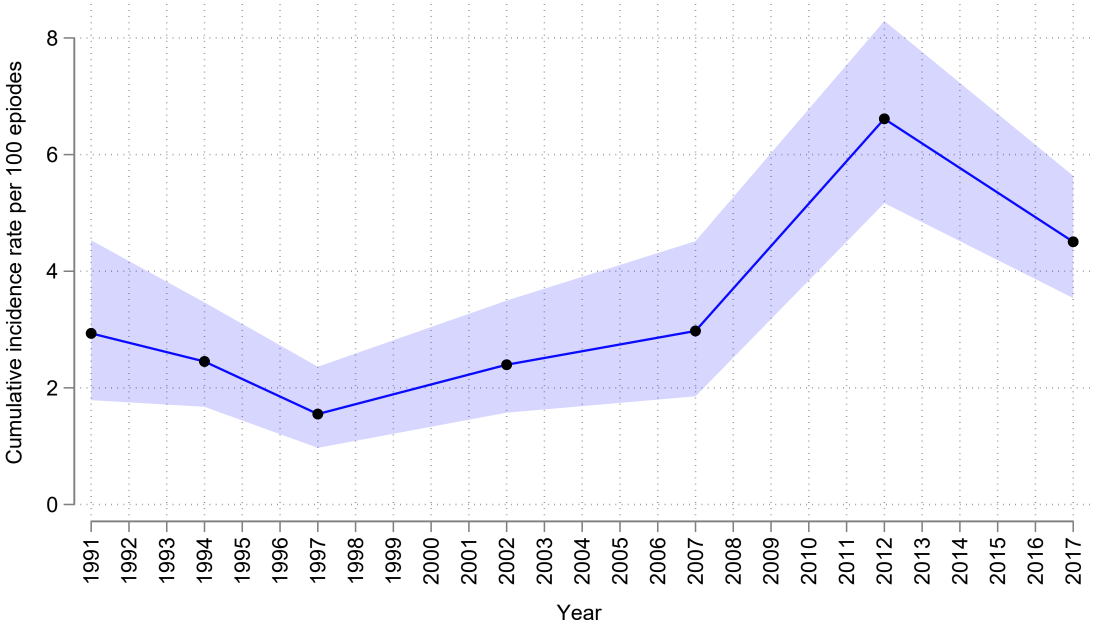

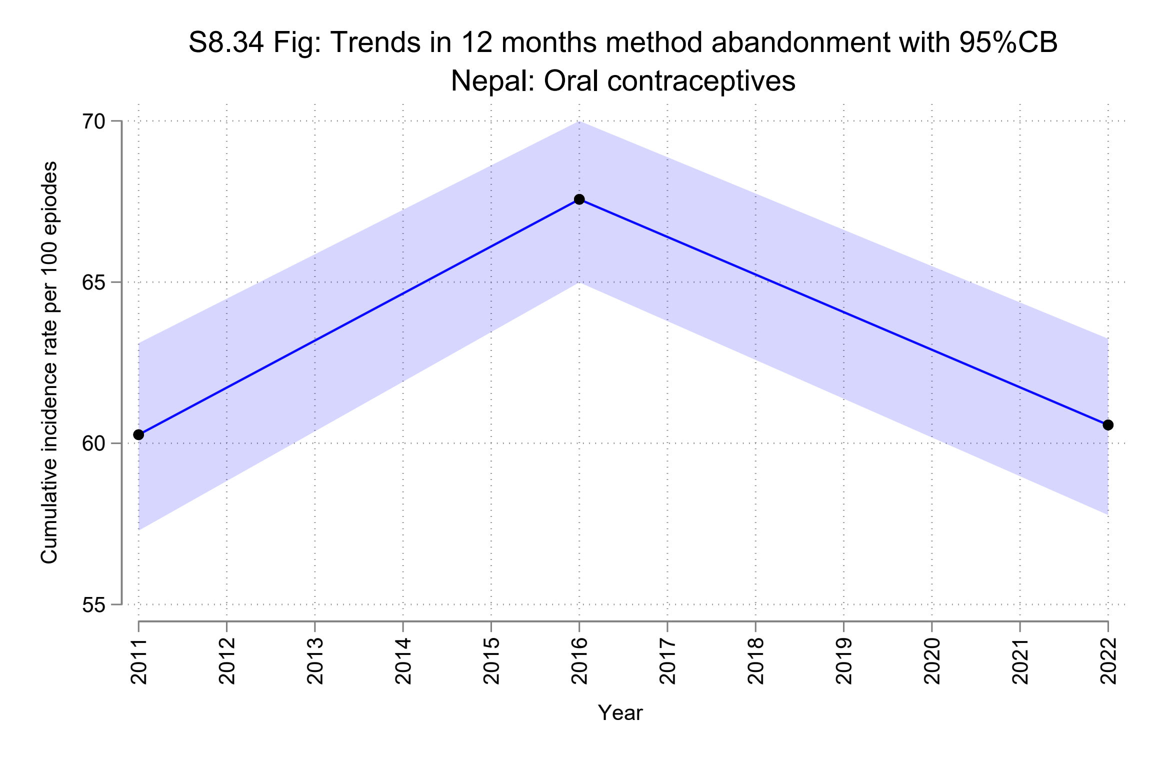
S8.34 Fig: Trends in 12 months method abandonment with 95%CB
Nepal: Oral contraceptives
Cumulative incidence rate per 100 epiodes
70
65
60
55
2011
2012
2013
2014
2015
2016
2017
2018
2019
2020
2021
2022
Year

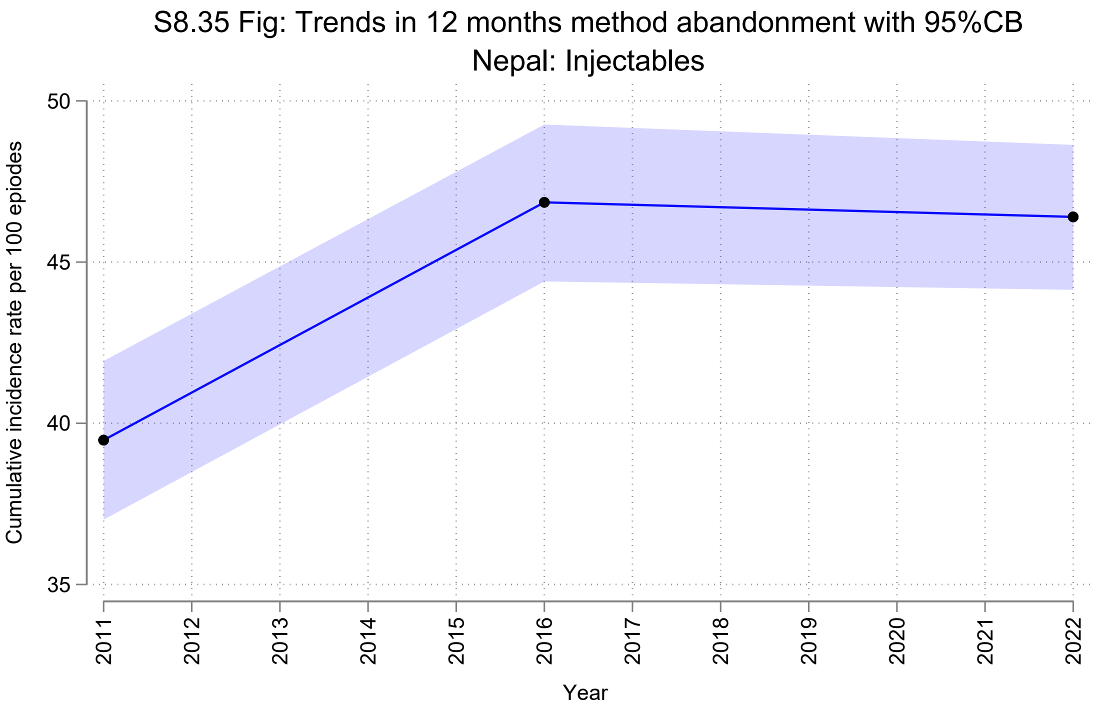
S8.35 Fig: Trends in 12 months method abandonment with 95%CB
Nepal: Injectables
Cumulative incidence rate per 100 epiodes
50
45
40
35
2011
2012
2013
2014
2015
2016
2017
2018
2019
2020
2021
2022
Year

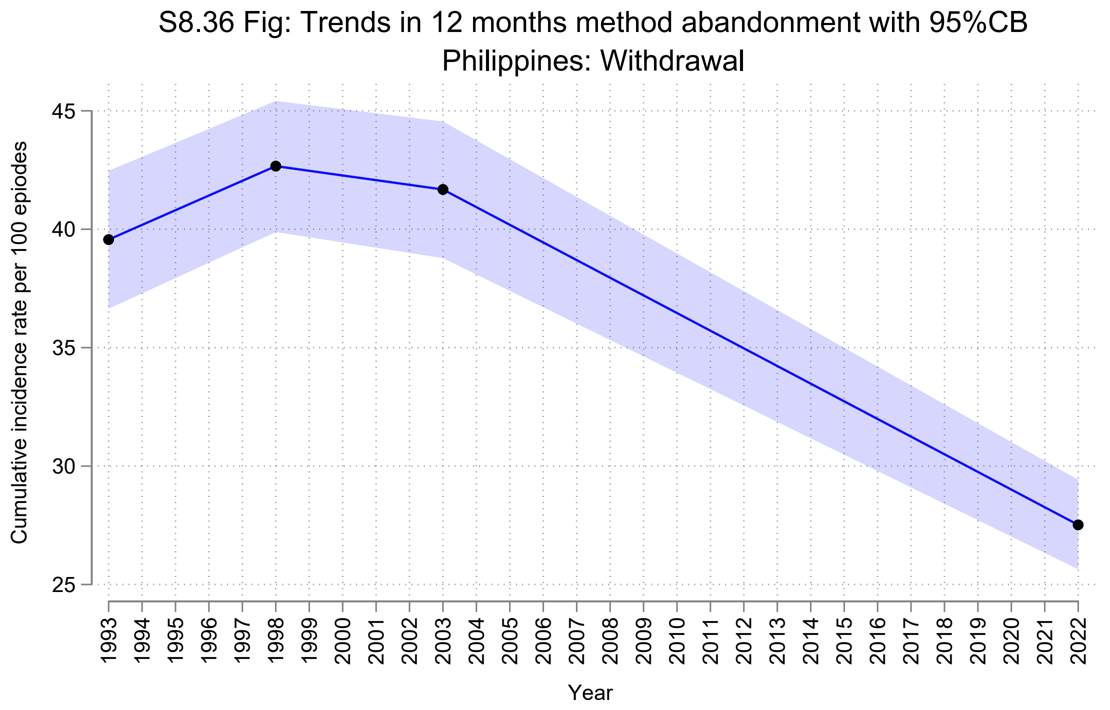
S8.36 Fig: Trends in 12 months method abandonment with 95%CB
Philippines: Withdrawal
Cumulative incidence rate per 100 epiodes
45
40
35
30
25
1993
1994
1995
1996
1997
1998
1999
2000
2001
2002
2003
2004
2005
2006
2007
2008
2009
2010
2011
2012
2013
2014
2015
2016
2017
2018
2019
2020
2021
2022
Year

S8.37 Fig: Trends in 12 months method abandonment with 95%CB
Colombia: Oral contraceptives

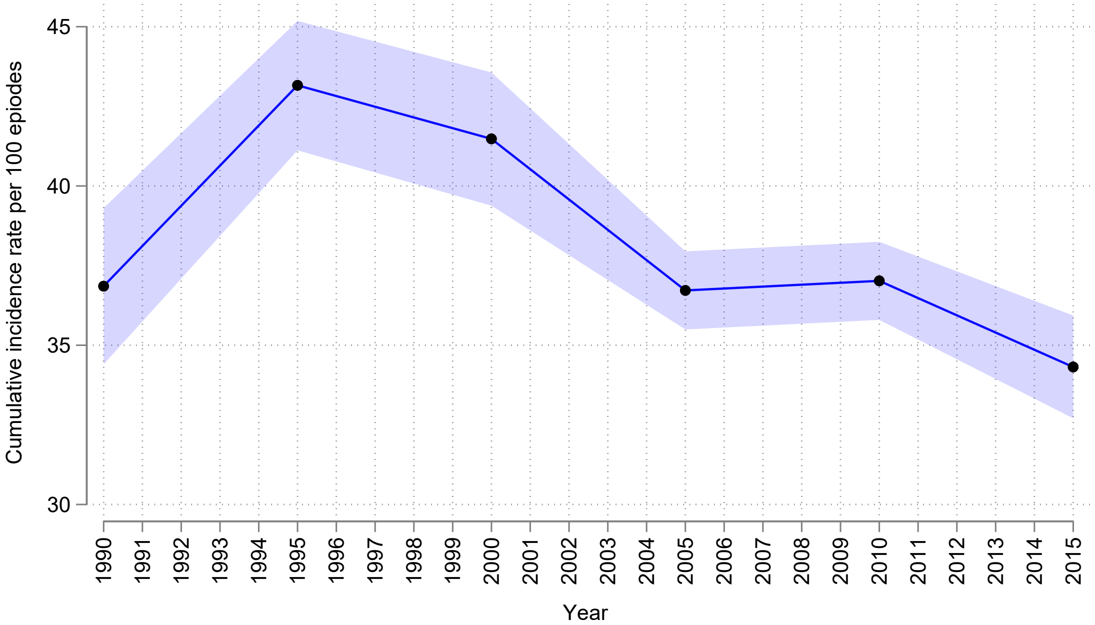

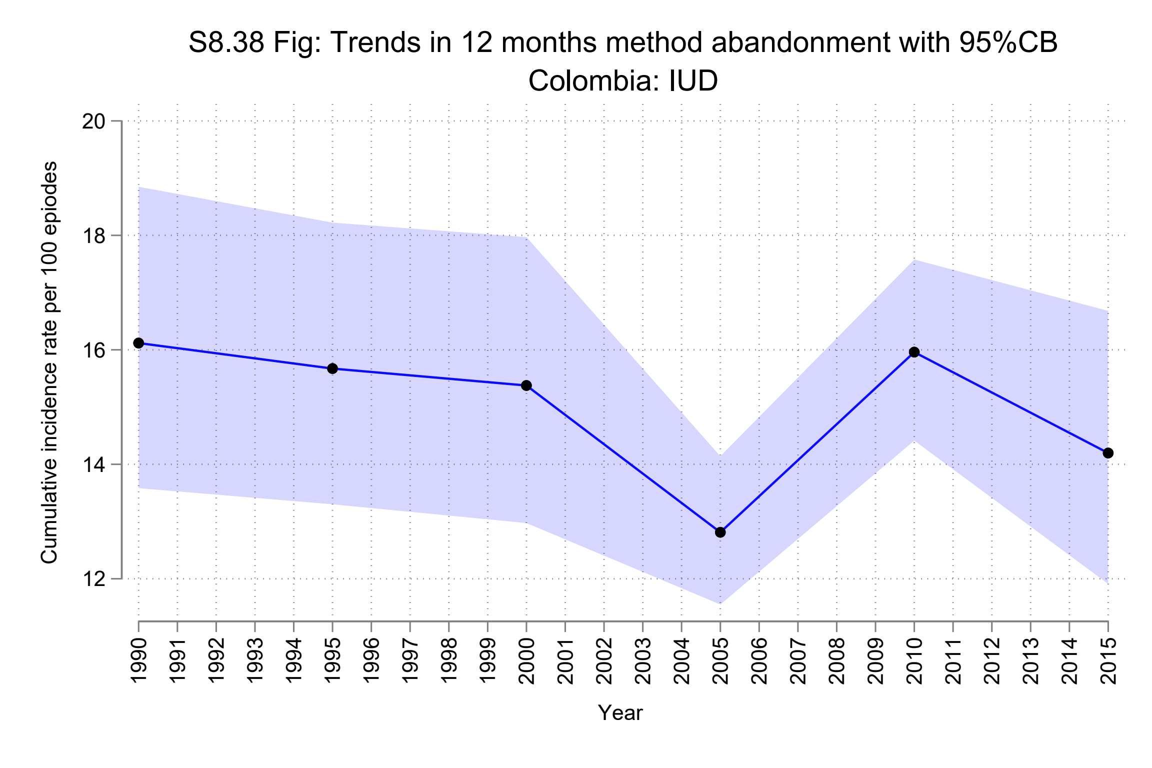
S8.38 Fig: Trends in 12 months method abandonment with 95%CB
Colombia: IUD
Cumulative incidence rate per 100 epiodes
20
18
16
14
12
1990
1991
1992
1993
1994
1995
1996
1997
1998
1999
2000
2001
2002
2003
2004
2005
2006
2007
2008
2009
2010
2011
2012
2013
2014
2015
Year

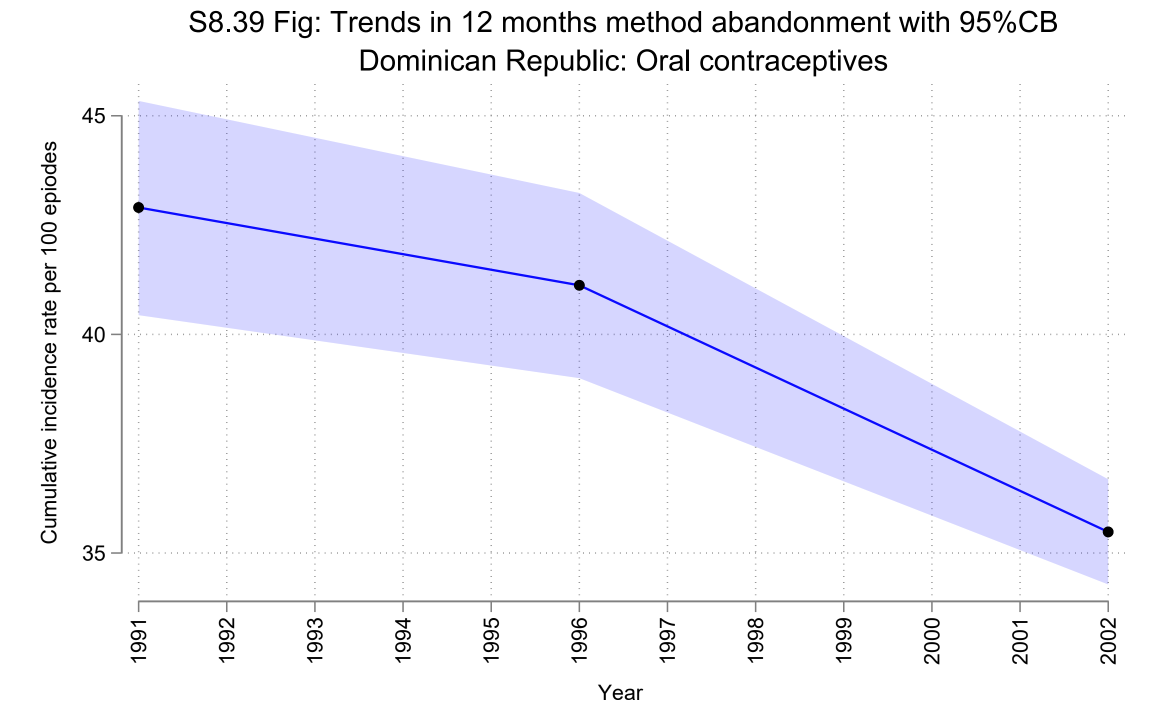

S8.39 Fig: Trends in 12 months method abandonment with 95%CB
Dominican Republic: Oral contraceptives
Cumulative incidence rate per 100 epiodes
45
40
35
1991
1992
1993
1994
1995
1996
1997
1998
1999
2000
2001
2002
Year

S8.40 Fig: Trends in 12 months method abandonment with 95%CB
Dominican Republic: Condom

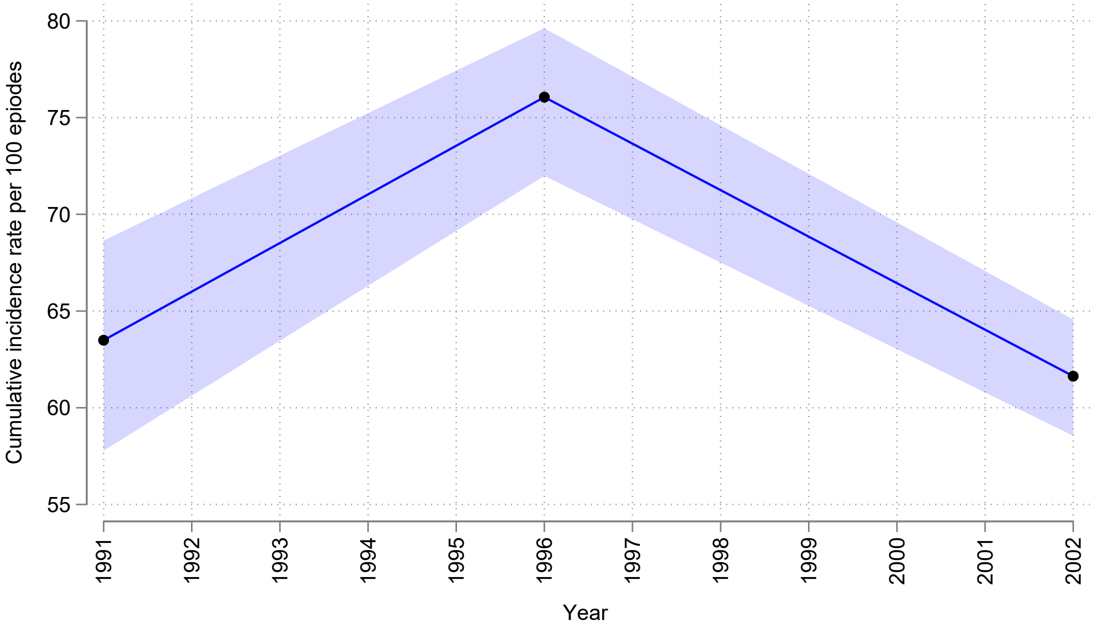

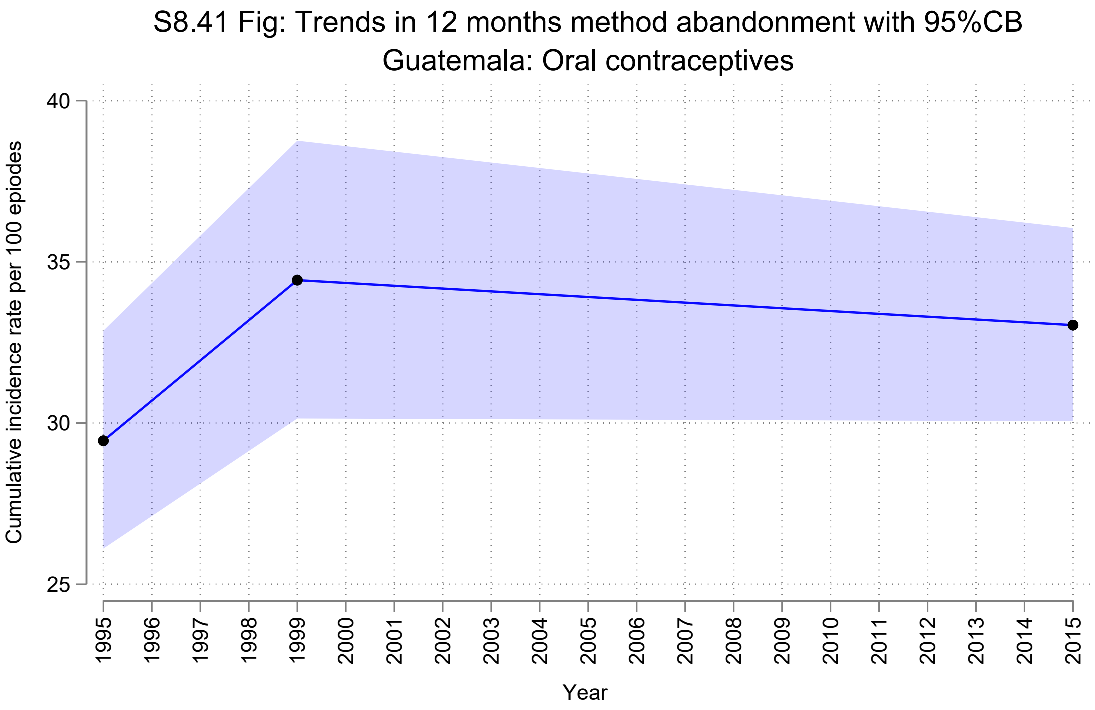
S8.41 Fig: Trends in 12 months method abandonment with 95%CB
Guatemala: Oral contraceptives
Cumulative incidence rate per 100 epiodes
40
35
30
25
1995
1996
1997
1998
1999
2000
2001
2002
2003
2004
2005
2006
2007
2008
2009
2010
2011
2012
2013
2014
2015
Year

S8.42 Fig: Trends in 12 months method abandonment with 95%CB
Guatemala: Injectables

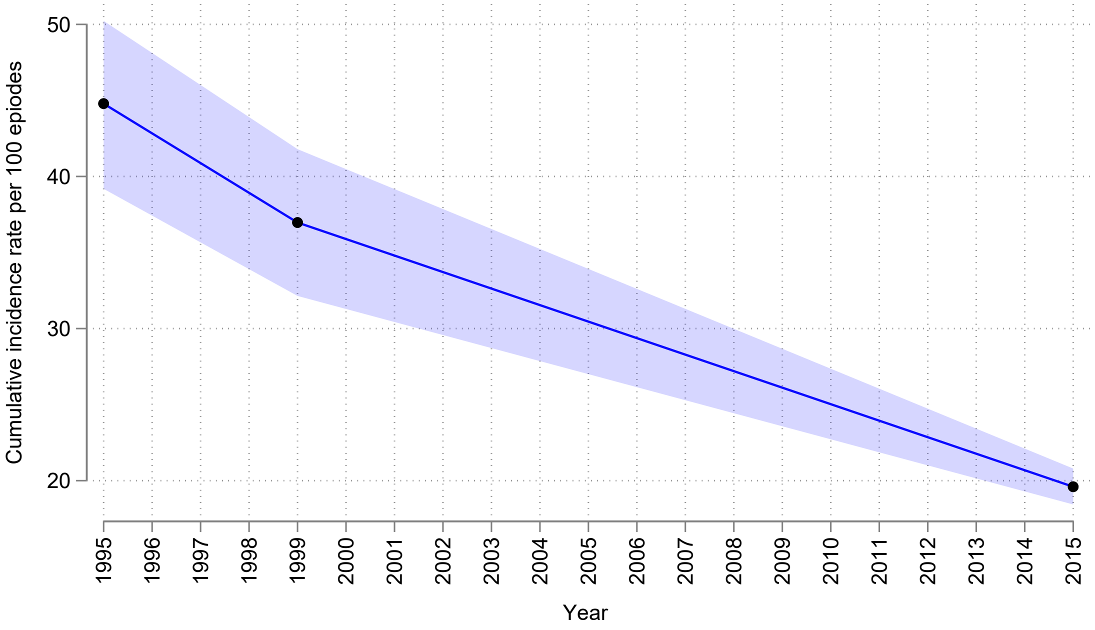

S8.43 Fig: Trends in 12 months method abandonment with 95%CB
Guatemala: Condom

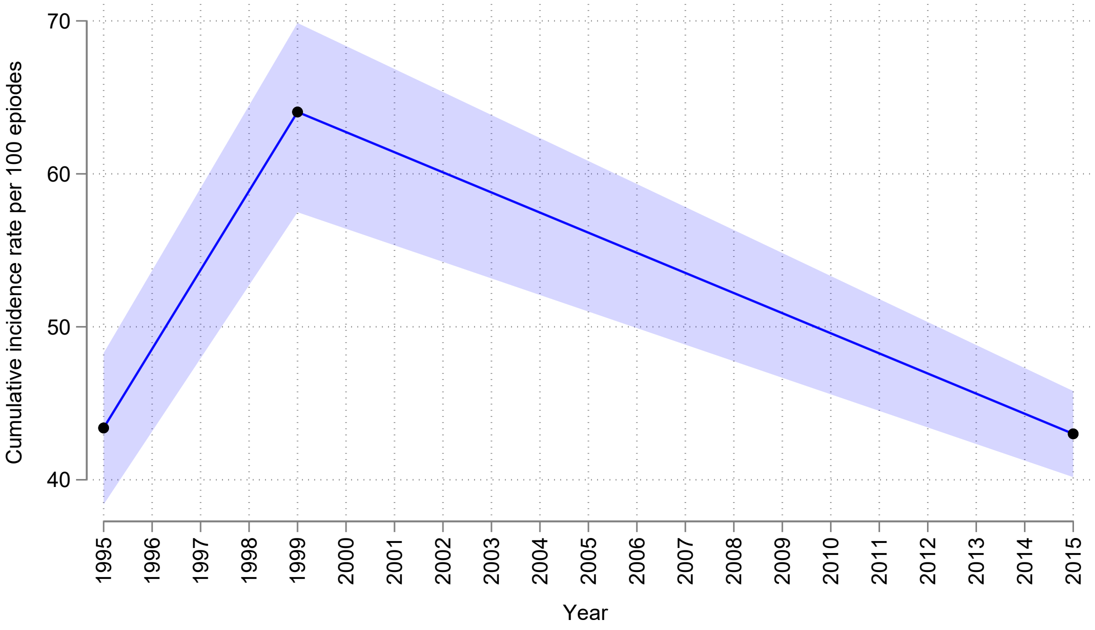

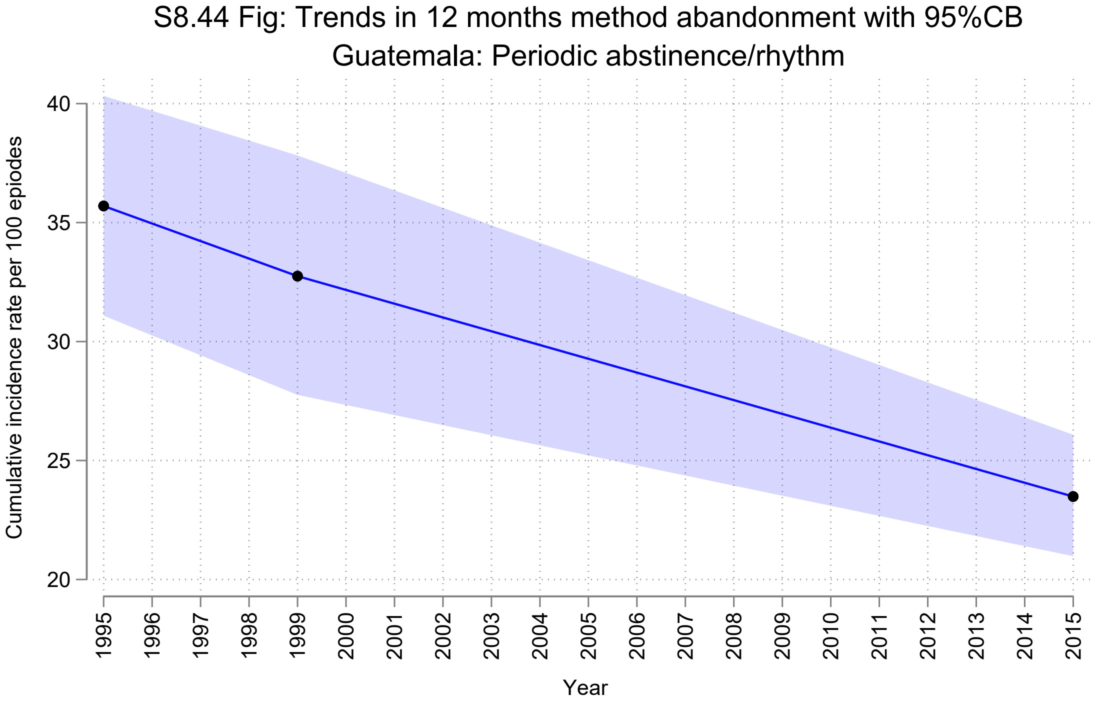
S8.44 Fig: Trends in 12 months method abandonment with 95%CB
Guatemala: Periodic abstinence/rhythm
Cumulative incidence rate per 100 episodes
40
35
30
25
20
1995
1996
1997
1998
1999
2000
2001
2002
2003
2004
2005
2006
2007
2008
2009
2010
2011
2012
2013
2014
2015
Year

S8.45 Fig: Trends in 12 months method abandonment with 95%CB
Peru: Oral contraceptives

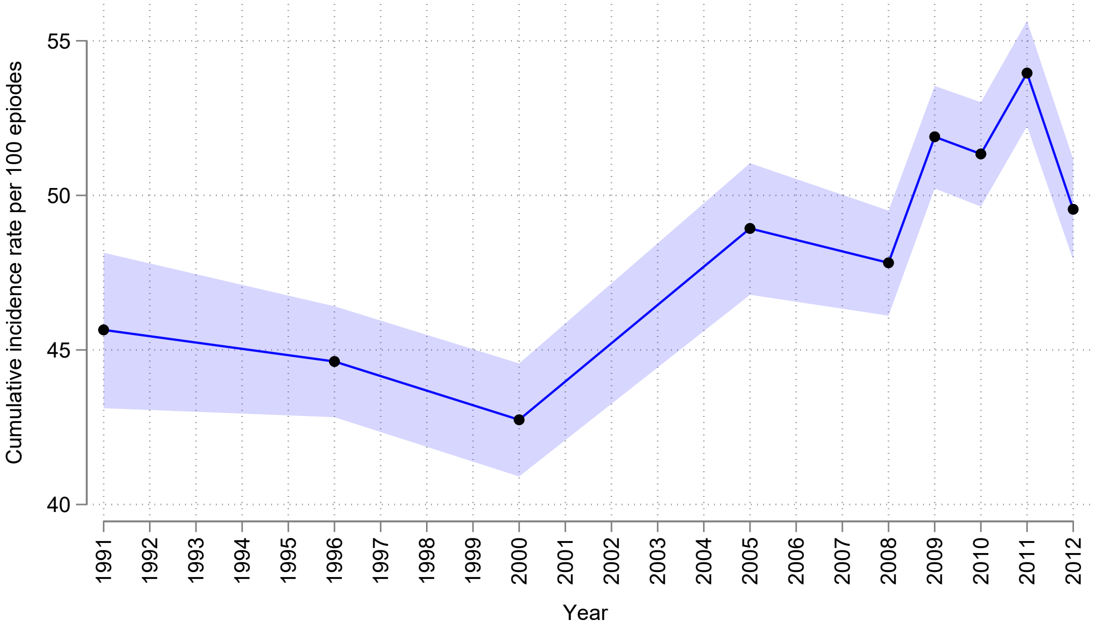

S8.46 Fig: Trends in 12 months method abandonment with 95%CB
Peru: Injectables

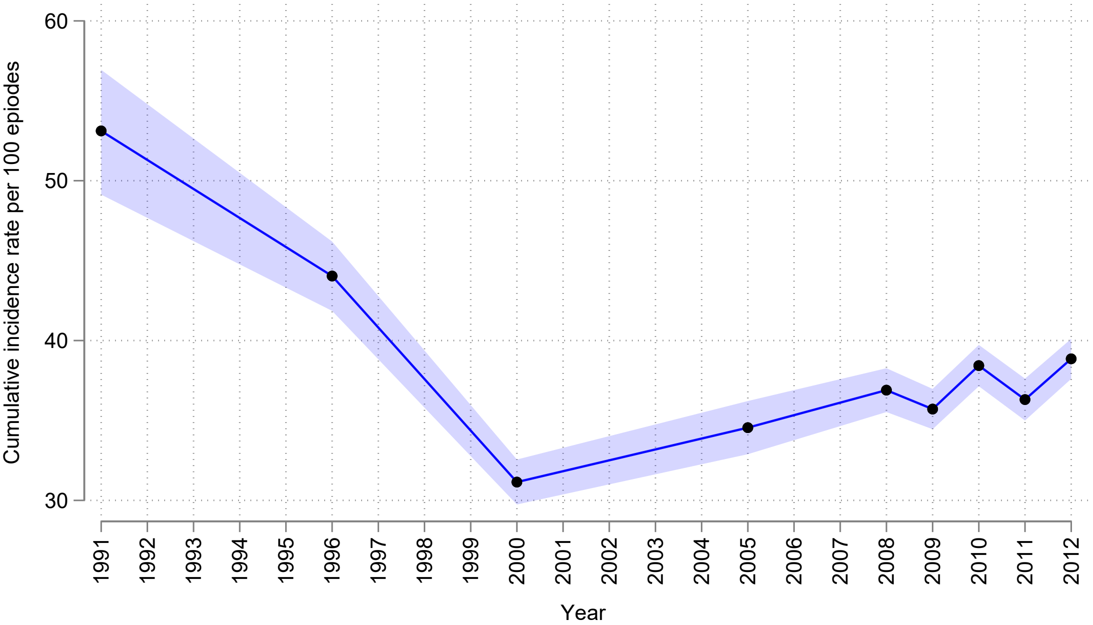

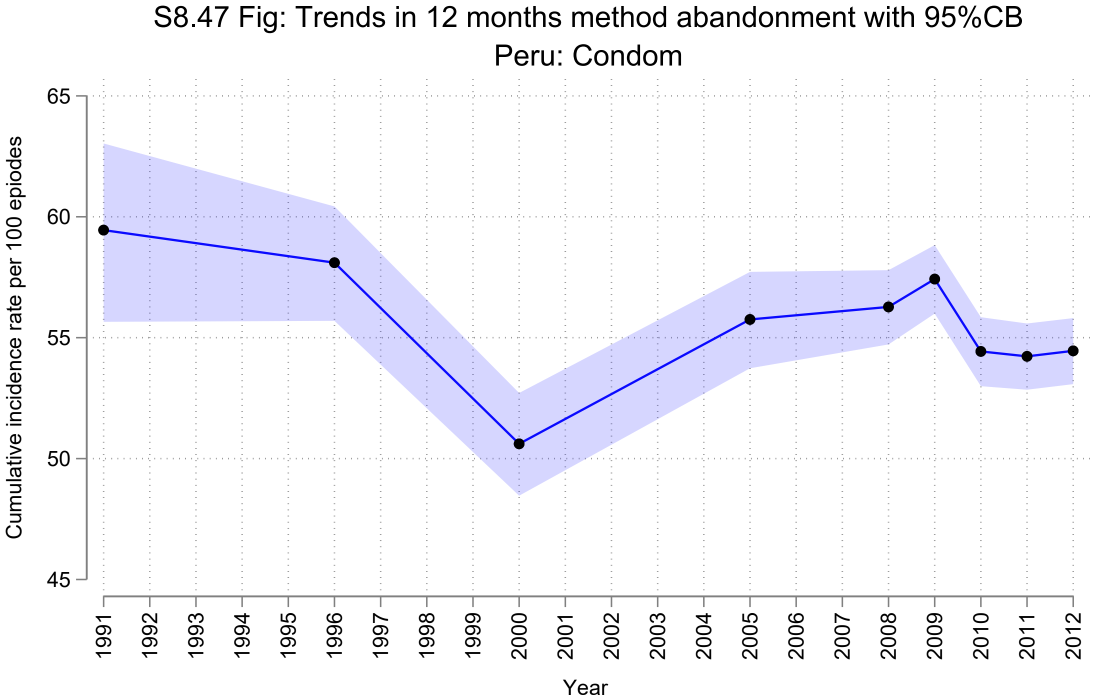
S8.47 Fig: Trends in 12 months method abandonment with 95%CB
Peru: Condom
Cumulative incidence rate per 100 epiodes
65
60
55
50
45
1991
1992
1993
1994
1995
1996
1997
1998
1999
2000
2001
2002
2003
2004
2005
2006
2007
2008
2009
2010
2011
2012
Year

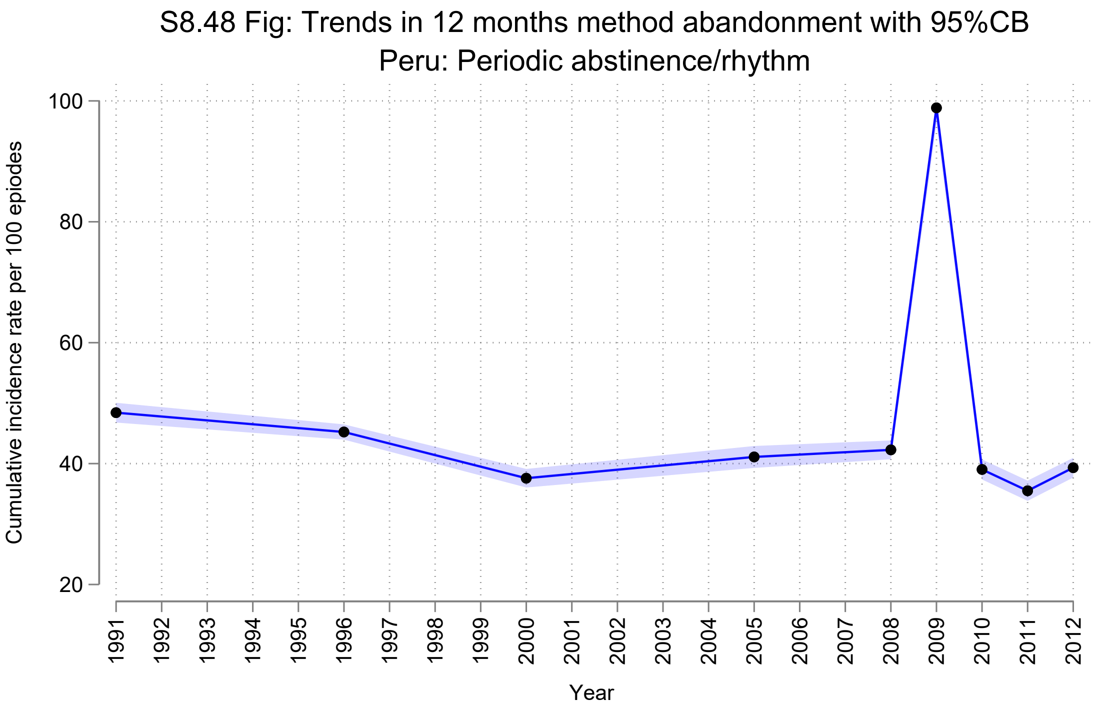
S8.48 Fig: Trends in 12 months method abandonment with 95%CB
Peru: Periodic abstinence/rhythm
Cumulative incidence rate per 100 epiodes
100
80
60
40
20
1991
1992
1993
1994
1995
1996
1997
1998
1999
2000
2001
2002
2003
2004
2005
2006
2007
2008
2009
2010
2011
2012
Year
