# Supplementary material for: Contraceptive discontinuation, switching, abandonment and their reproductive consequences: An analysis of 1,539,071 episodes of reversible method use contributed from 61 countries that participated in DHS: Population base-analysis
Source: PLOS Glob Public Health. 2025 Oct 31;5(10):e0005174. doi: 10.1371/journal.pgph.0005174 (PMC12578211; doi:10.1371/journal.pgph.0005174)
Supplement: S9 Fig — (PDF) [file pgph.0005174.s010.pdf]

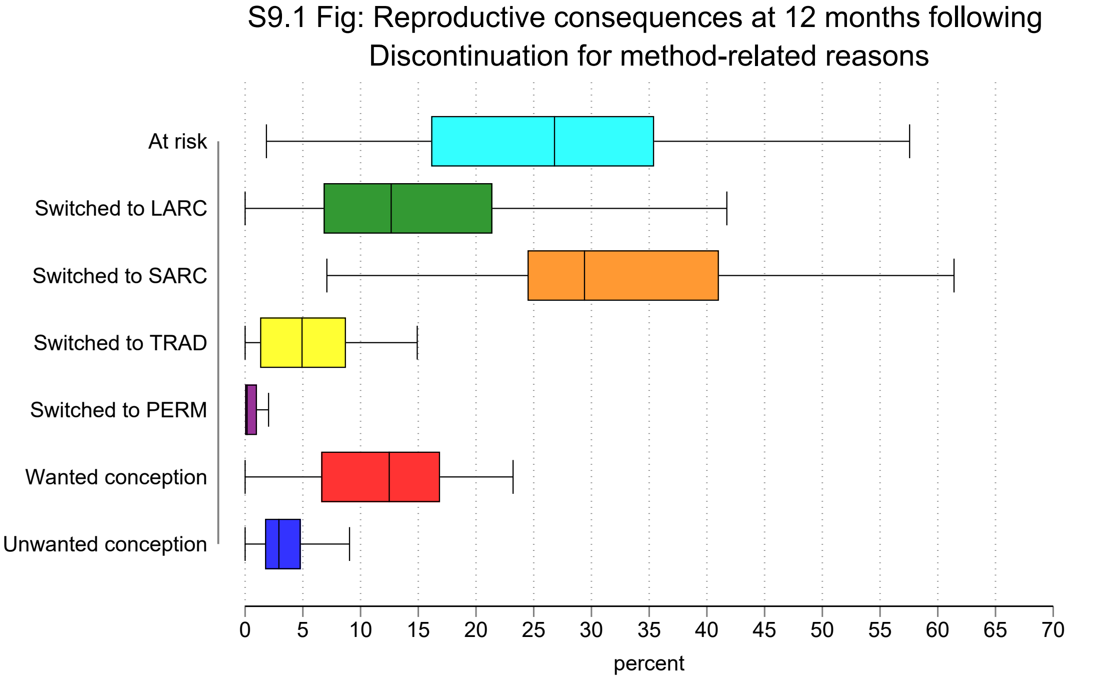

Based on 7 reversible methods with100+ episodes combined: OCs, IUDs, Injectables, Condom, Implant, PA and withdrawal. Most recent surveys since 2000

S9.2 Fig: Reproductive consequences at 12 months following Discontinuation for wanted pregnancy

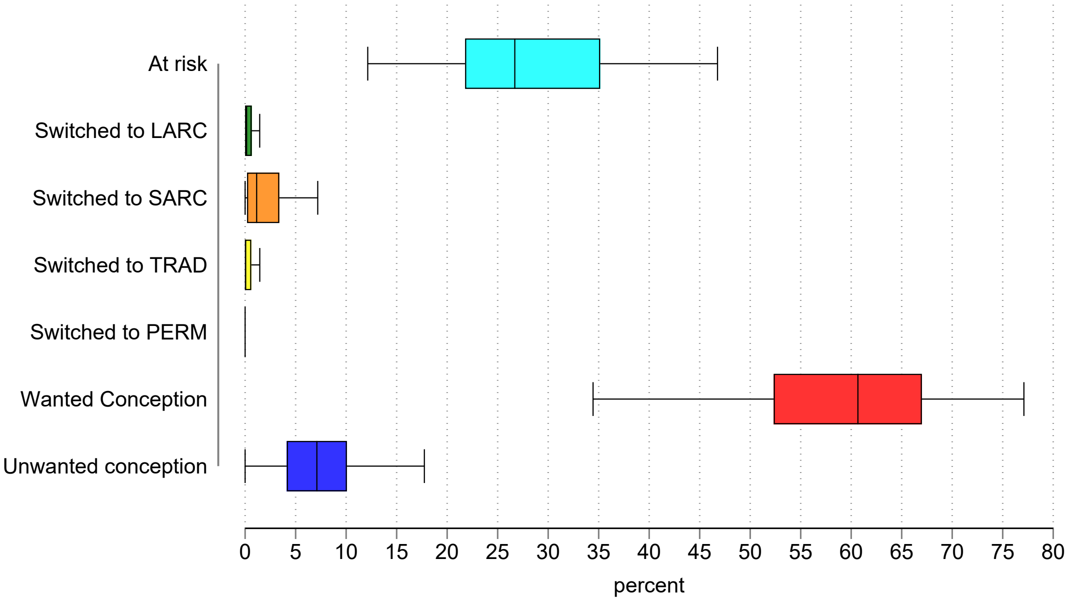

Based on 7 reversible methods with100+ episodes combined: OCs, IUDs, Injectables, Condom, Implant, PA and withdrawal. Most recent surveys since 2000

S9.3 Fig: Reproductive consequences at 12 months following Discontinuation for no further need reasons

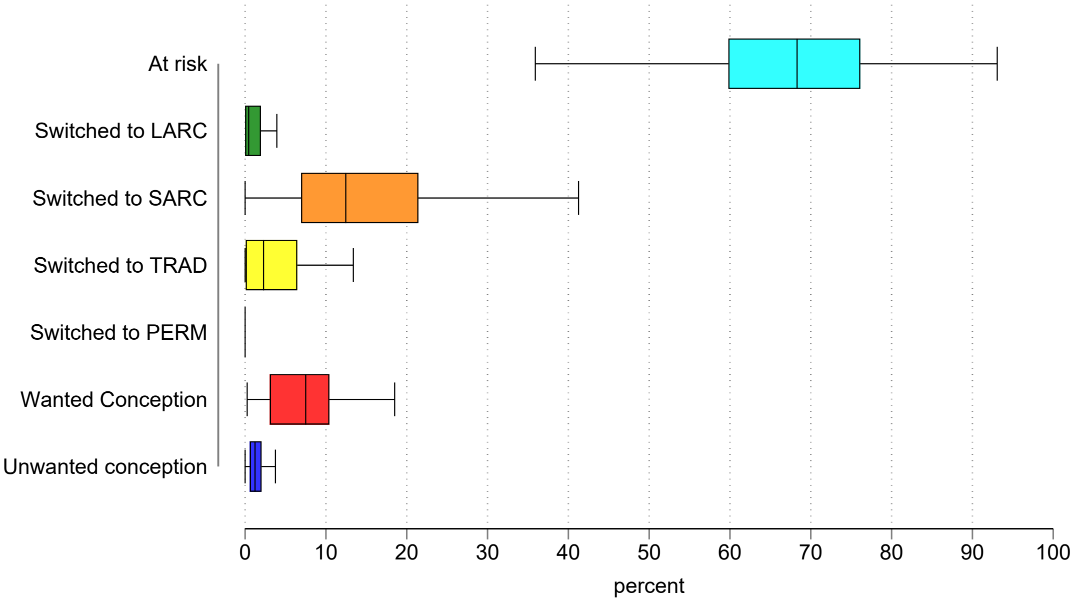

Based on 7 reversible methods with100+ episodes combined: OCs, IUDs, Injectables, Condom, Implant, PA and withdrawal. Most recent surveys since 2000

S9.4 Fig: Reproductive consequences at 12 months following Discontinuation for others or don't know reasons

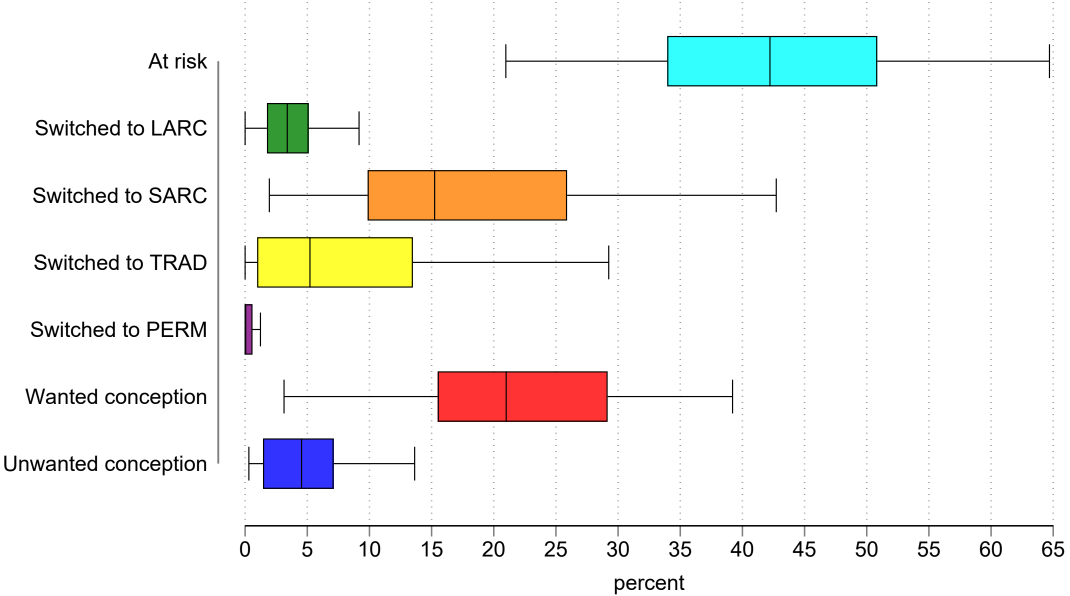

Based on 7 reversible methods with100+ episodes combined: OCs, IUDs, Injectables, Condom, Implant, PA and withdrawal. Most recent surveys since 2000
