# Supplementary material for: Contraceptive discontinuation, switching, abandonment and their reproductive consequences: An analysis of 1,539,071 episodes of reversible method use contributed from 61 countries that participated in DHS: Population base-analysis
Source: PLOS Glob Public Health. 2025 Oct 31;5(10):e0005174. doi: 10.1371/journal.pgph.0005174 (PMC12578211; doi:10.1371/journal.pgph.0005174)
Supplement: S2 Table — (PDF) [file pgph.0005174.s013.pdf]

S2 Table: Grouping of reported contraceptive methods

	Method grouped	Reported method	Group
**Modern methods**			
1	Oral contraceptives		SARC
2	IUD		LARC
3	Injectables		
		Injectables	SARC
		Injection 2 months	SARC
		Injections every 3 months	SARC
4	Condom		SARC
5	Sterilization		
		Female sterilization	Permanent
		Male sterilization	Permanent
6	Implants		LARC
7	Lactation amenorrhea method (LAM)		SARC
8	Other modern methods		
		Diaphragm	SARC
		Female condom	SARC
		Foam and Jelly	SARC
		Diaphragm/Foam/Jelly	SARC
		Patch	SARC
		Emergency contraception	SARC
		Suppository	SARC
		Vaginal ring	SARC
		Other modern methods	SARC
		Intra-vaginal method	SARC
		Chinese (monthly) pill	SARC
**Traditional methods**			
9	Periodic abstinence/rhythm		Traditional
10	Withdrawal		Traditional
11	Other traditional methods		
		Abstinence	Traditional
		Vaginal douche	Traditional
		Prolonged breastfeeding	Traditional
		Mucus	Traditional
		Basal body temperature/Symptothermal	Traditional
		Herbs	Traditional
		Massage	Traditional

LARC=Long Acting Reversible Method, SARC=Short Acting Reversible Method
