# Supplementary material for: Contraceptive discontinuation, switching, abandonment and their reproductive consequences: An analysis of 1,539,071 episodes of reversible method use contributed from 61 countries that participated in DHS: Population base-analysis
Source: PLOS Glob Public Health. 2025 Oct 31;5(10):e0005174. doi: 10.1371/journal.pgph.0005174 (PMC12578211; doi:10.1371/journal.pgph.0005174)
Supplement: S3 Table — (PDF) [file pgph.0005174.s014.pdf]

S3 Table: Grouping of reported reasons for discontinuations

Group reason		Reported reason
**1- Became pregnant while using**		
**2- Side effects including health concerns**		
	1	Side effects
	2	Health concerns
	3	Changes in menstrual bleeding
	4	Created menstrual problem
	5	Gained weight
**3-Other method-related**		
	1	Access, availability
	2	Wanted more effective method
	3	Inconvenient to use
	4	Cost
	5	To take a Rest/Break
	6	IUD expelled
	7	IUD expired
	8	Advice of doctor/ Medical advice
	9	Switch method/brand
	10	End of breastfeeding
	11	Period Returned
	12	Changed method
	13	Afraid of forgetting method
	14	Afraid of using method
	15	Doctor's opinion
	16	Method not available
	17	Lack of sexual satisfaction
	18	Did not like method
	19	Lack of privacy
	20	The absence of one condition of breastfeeding
	21	Expiration/lack of method
	22	Erectile dysfunction in husband
**4-Wanted to become pregnant/no need**		
	1	Wanted to become pregnant
	2	Infrequent sex, husband away
	3	Difficult to get pregnant/menopause
	4	Marital dissolution
	5	To opt for Hysterectomy
	6	Death of spouse
	7	Husband travelling/ill/absent/away
	8	Can't get pregnant
	9	Ramadan

5- Other/not stated

1	Husband disapproved
2	Fatalistic
3	Thinks she is pregnant
4	Method not available due to lockdown
5	Other
6	missing coded in calendar
7	Don't know
