# Supplementary material for: Contraceptive discontinuation, switching, abandonment and their reproductive consequences: An analysis of 1,539,071 episodes of reversible method use contributed from 61 countries that participated in DHS: Population base-analysis
Source: PLOS Glob Public Health. 2025 Oct 31;5(10):e0005174. doi: 10.1371/journal.pgph.0005174 (PMC12578211; doi:10.1371/journal.pgph.0005174)
Supplement: S4 Table — (PDF) [file pgph.0005174.s015.pdf]

**S4 Table: Comparison of all causes discontinuation at 12 months between 3 and 5 years analysis periods**

| | | Comparing Rate based on 3 years window to the rates based on 5 years window | | |
|---|---|---|---|---|
| Method | Number of country | Rate P3< LL rate P5 | Rate P3 within 95%CI of rate P5 | Rate P3> UL rate P5 |
| Pill | 52 | 11.5 | 69.2 | 19.2 |
| IUD | 33 | 15.2 | 84.9 | 0.0 |
| Injectables | 45 | 26.7 | 55.6 | 17.8 |
| Condom | 47 | 17.0 | 70.2 | 12.8 |
| Implants | 31 | 29.0 | 71.0 | 0.0 |
| Periodic abstinence | 30 | 10.0 | 76.7 | 13.3 |
| Withdrawal | 36 | 5.6 | 69.4 | 25.0 |
| | | | | |
| Median | | **15.2** | **70.2** | **13.3** |

LL=Lower Limit, UL=Upper Limit, CI=Confidence Intervals

P3= period 3 years, P5= Period 5 years
