# Supplementary material for: Contraceptive discontinuation, switching, abandonment and their reproductive consequences: An analysis of 1,539,071 episodes of reversible method use contributed from 61 countries that participated in DHS: Population base-analysis
Source: PLOS Glob Public Health. 2025 Oct 31;5(10):e0005174. doi: 10.1371/journal.pgph.0005174 (PMC12578211; doi:10.1371/journal.pgph.0005174)
Supplement: S5 Table — (PDF) [file pgph.0005174.s016.pdf]

**S5 Table : Number of method-specific episodes reported in the calendar during months 4-63 prior to survey date**

| Region | | All methods | Modern methods | | | | | | | | Traditional methods | | |
|---|---|---|---|---|---|---|---|---|---|---|---|---|---|
| Country | Survey year | | Oral contraceptive | IUD | Injectables | Condom | Sterilisation (Female & Male) | Implant | LAM | Other modem methods | Periodic abstinence | Withdrawal | Other traditional methods |
| **Sub-Saharan Africa** | | **(265,736)** | **(55,674)** | **(5,324)** | **(97,832)** | **(27,267)** | **(3,405)** | **(38,130)** | **(6,317)** | **(631)** | **(12,966)** | **(11,138)** | **(3,893)** |
| Angola | 2015/16 | 1,861 | 448 | 9 | 401 | 724 | 2 | 39 | 15 | 23 | 50 | 107 | 21 |
| Benin | 2017/18 | 3,849 | 512 | 231 | 631 | 452 | 12 | 999 | 26 | 7 | 463 | 332 | 54 |
| Burkina Faso | 2010 | 3,531 | 786 | 36 | 1,325 | 588 | 11 | 547 | 7 | 8 | 202 | 12 | 5 |
| Burkina Faso | 2021 | 8,045 | 864 | 325 | 2,115 | 722 | 12 | 3,220 | 113 | 3 | 540 | 25 | 9 |
| Burundi | 2010/11 | 2,179 | 328 | 139 | 1,112 | 140 | 21 | 42 | 17 | 2 | 173 | 195 | 1 |
| Comoros | 2012 | 877 | 131 | 4 | 249 | 123 | 11 | 64 | 36 | 0 | 141 | 112 | 4 |
| Côte d'Ivoire | 2021 | 4,264 | 994 | 22 | 887 | 685 | 4 | 863 | 27 | 9 | 488 | 59 | 85 |
| Ethiopia | 2005 | 2,719 | 748 | 30 | 1,486 | 148 | 7 | 26 | 29 | 0 | 191 | 46 | 5 |
| Ethiopia | 2016 | 5,318 | 560 | 202 | 3,216 | 81 | 13 | 1,000 | 36 | 1 | 110 | 30 | 0 |
| Gabon | 2019/21 | 3,197 | 326 | 5 | 135 | 1,618 | 7 | 64 | 23 | 13 | 411 | 437 | 19 |
| Gambia | 2013 | 883 | 258 | 21 | 402 | 85 | 23 | 17 | 1 | 4 | 11 | 16 | 44 |
| Gambia | 2019/20 | 2,900 | 388 | 54 | 1,359 | 77 | 25 | 744 | 21 | 1 | 19 | 61 | 144 |
| Ghana | 2014 | 2,828 | 546 | 43 | 953 | 279 | 61 | 372 | 9 | 76 | 301 | 156 | 24 |
| Ghana | 2022/23 | 7,879 | 1,008 | 69 | 2,060 | 494 | 138 | 1,402 | 187 | 17 | 923 | 457 | 202 |
| Guinea | 2018 | 2,767 | 540 | 251 | 510 | 317 | 5 | 416 | 529 | 1 | 27 | 64 | 21 |
| Kenya | 1998 | 3,109 | 927 | 108 | 843 | 279 | 120 | 39 | 0 | 0 | 670 | 64 | 58 |
| Kenya | 2003 | 3,595 | 953 | 113 | 1,193 | 355 | 77 | 86 | 0 | 0 | 656 | 81 | 79 |
| Kenya | 2014 | 7,997 | 1,419 | 272 | 3,660 | 650 | 139 | 983 | 52 | 28 | 613 | 140 | 35 |
| Kenya | 2022 | 9,951 | 1,263 | 365 | 3,359 | 675 | 74 | 2,703 | 393 | 6 | 586 | 211 | 34 |
| Lesotho | 2014 | 3,903 | 760 | 42 | 1,467 | 1,475 | 23 | 53 | 0 | 9 | 5 | 45 | 6 |
| Liberia | 2013 | 2,330 | 716 | 1 | 1,303 | 65 | 8 | 157 | 4 | 5 | 60 | 3 | 4 |
| Liberia | 2019/20 | 3,447 | 501 | 9 | 2,164 | 106 | 4 | 553 | 3 | 2 | 62 | 31 | 2 |
| Madagascar | 2021 | 10,830 | 1,139 | 118 | 5,739 | 165 | 55 | 1,940 | 139 | 0 | 1,247 | 234 | 53 |
| Malawi | 2004/5 | 5,250 | 468 | 10 | 2,985 | 511 | 302 | 43 | 0 | 0 | 93 | 514 | 320 |
| Malawi | 2015/16 | 15,783 | 963 | 205 | 9,231 | 1,332 | 802 | 2,634 | 92 | 4 | 73 | 282 | 110 |
| Mali | 2012/13 | 1,644 | 547 | 49 | 607 | 52 | 6 | 287 | 6 | 0 | 8 | 6 | 70 |
| Mali | 2018 | 2,464 | 447 | 109 | 922 | 22 | 16 | 799 | 14 | 3 | 24 | 28 | 64 |
| Mozambique | 2011 | 3,142 | 1,225 | 24 | 959 | 792 | 14 | 4 | 52 | 5 | 20 | 13 | 16 |
| Mozambique | 2022/23 | 5,358 | 961 | 131 | 2,260 | 621 | 29 | 1,169 | 9 | 2 | 42 | 77 | 27 |
| Namibia | 2013 | 5,151 | 587 | 38 | 2,526 | 1,737 | 101 | 12 | 9 | 10 | 15 | 18 | 21 |
| Niger | 2012 | 3,817 | 1,626 | 18 | 531 | 19 | 11 | 46 | 1,235 | 7 | 22 | 10 | 290 |
| Nigeria | 2013 | 8,247 | 1,087 | 276 | 1,281 | 2,178 | 39 | 78 | 643 | 238 | 868 | 1,216 | 293 |
| Nigeria | 2018 | 9,308 | 1,025 | 230 | 1,830 | 1,460 | 32 | 1,183 | 893 | 40 | 781 | 1,371 | 278 |
| Rwanda | 2010/11 | 5,777 | 1,038 | 37 | 3,068 | 270 | 37 | 539 | 73 | 0 | 298 | 334 | 5 |
| Rwanda | 2014/15 | 6,390 | 1,285 | 113 | 3,216 | 361 | 50 | 690 | 17 | 0 | 243 | 282 | 0 |
| Rwanda | 2019/20 | 8,023 | 1,222 | 199 | 2,382 | 536 | 88 | 2,561 | 31 | 1 | 354 | 421 | 25 |
| Senegal | 2010/11 | 2,489 | 688 | 54 | 982 | 182 | 15 | 137 | 211 | 13 | 67 | 24 | 109 |
| Senegal | 2015 | 2,167 | 491 | 62 | 917 | 89 | 12 | 361 | 16 | 6 | 66 | 19 | 125 |

| | | | | | | | | | | | | | |
|---|---|---|---|---|---|---|---|---|---|---|---|---|---|
| Senegal | 2016 | 2,283 | 508 | 80 | 912 | 61 | 14 | 504 | 35 | 5 | 50 | 18 | 93 |
| Senegal | 2018 | 2,760 | 494 | 125 | 1,087 | 68 | 13 | 786 | 21 | 1 | 40 | 11 | 96 |
| Senegal | 2019 | 2,451 | 402 | 120 | 1,004 | 51 | 12 | 734 | 6 | 0 | 30 | 9 | 75 |
| Senegal | 2023 | 4,220 | 582 | 231 | 1,481 | 51 | 14 | 1,698 | 49 | 0 | 19 | 28 | 44 |
| Sierra Leone | 2013 | 4,649 | 1,200 | 72 | 2,000 | 135 | 34 | 798 | 182 | 20 | 28 | 30 | 145 |
| South Africa | 2016 | 4,006 | 385 | 63 | 2,026 | 1,010 | 122 | 328 | 0 | 6 | 3 | 21 | 0 |
| Tanzania | 2004/5 | 4,325 | 1,226 | 16 | 1,362 | 514 | 84 | 31 | 95 | 0 | 328 | 482 | 182 |
| Tanzania | 2015/16 | 6,027 | 958 | 109 | 2,333 | 517 | 126 | 918 | 93 | 4 | 529 | 335 | 87 |
| Tanzania | 2022 | 6,737 | 535 | 116 | 2,048 | 312 | 117 | 2,336 | 139 | 0 | 615 | 411 | 82 |
| Uganda | 2011 | 3,461 | 498 | 42 | 1,747 | 439 | 83 | 179 | 25 | 39 | 144 | 225 | 35 |
| Zambia | 2013/14 | 9,448 | 2,514 | 136 | 3,760 | 849 | 87 | 724 | 305 | 9 | 120 | 820 | 90 |
| Zambia | 2018/19 | 7,860 | 1,344 | 47 | 4,105 | 611 | 70 | 1,039 | 146 | 0 | 27 | 383 | 33 |
| Zimbabwe | 1994 | 3,508 | 2,408 | 46 | 136 | 323 | 65 | 5 | 0 | 0 | 40 | 319 | 162 |
| Zimbabwe | 1999 | 3,400 | 2,108 | 24 | 659 | 233 | 55 | 15 | 86 | 0 | 32 | 145 | 39 |
| Zimbabwe | 2005/6 | 5,310 | 3,470 | 15 | 940 | 426 | 48 | 66 | 105 | 0 | 21 | 160 | 42 |
| Zimbabwe | 2010/11 | 5,163 | 3,369 | 13 | 852 | 536 | 21 | 196 | 22 | 3 | 6 | 103 | 24 |
| Zimbabwe | 2015 | 6,859 | 3,898 | 45 | 1,114 | 666 | 34 | 901 | 40 | 0 | 11 | 105 | 2 |
| **North Africa Western Asia and Europe** | | **(212,494)** | **(54,263)** | **(56,186)** | **(13,873)** | **(16,233)** | **(2,636)** | **(1,139)** | **(11,997)** | **(68)** | **(8,381)** | **(37,114)** | **(1,923)** |
| Albania | 2017/18 | 1,851 | 83 | 17 | 10 | 182 | 33 | 0 | 0 | 0 | 13 | 1,508 | 1 |
| Armenia | 2000 | 3,597 | 83 | 279 | 7 | 398 | 59 | 1 | 367 | 0 | 259 | 2,038 | 15 |
| Armenia | 2005 | 2,137 | 85 | 258 | 2 | 365 | 3 | 0 | 32 | 0 | 140 | 1,078 | 151 |
| Armenia | 2010 | 1,749 | 69 | 207 | 0 | 410 | 5 | 0 | 70 | 0 | 104 | 841 | 21 |
| Armenia | 2015/16 | 2,208 | 91 | 194 | 5 | 468 | 10 | 0 | 266 | 8 | 117 | 1,040 | 9 |
| Azerbaijan | 2006 | 2,805 | 104 | 352 | 3 | 187 | 6 | 0 | 149 | 0 | 178 | 1,795 | 9 |
| Egypt | 1992/93 | 6,406 | 2,515 | 2,796 | 121 | 338 | 55 | 2 | 0 | 0 | 131 | 96 | 16 |
| Egypt | 1995/96 | 8,929 | 3,011 | 4,154 | 504 | 427 | 61 | 4 | 0 | 0 | 161 | 120 | 18 |
| Egypt | 2000 | 10,564 | 2,615 | 5,280 | 1,472 | 218 | 93 | 28 | 0 | 0 | 90 | 63 | 15 |
| Egypt | 2003 | 6,952 | 1,673 | 2,909 | 1,279 | 115 | 38 | 75 | 0 | 0 | 70 | 46 | 6 |
| Egypt | 2005 | 15,684 | 4,207 | 6,575 | 2,950 | 246 | 88 | 239 | 0 | 0 | 117 | 68 | 7 |
| Egypt | 2008 | 11,178 | 3,161 | 4,726 | 1,753 | 130 | 62 | 144 | 0 | 0 | 67 | 41 | 4 |
| Egypt | 2014 | 15,533 | 6,431 | 5,364 | 2,006 | 131 | 102 | 207 | 0 | 0 | 70 | 57 | 6 |
| Jordan | 1990 | 5,485 | 1,128 | 1,478 | 11 | 131 | 177 | 0 | 0 | 0 | 676 | 557 | 32 |
| Jordan | 1997 | 6,302 | 1,311 | 1,701 | 67 | 383 | 121 | 5 | 0 | 0 | 841 | 1,091 | 23 |
| Jordan | 2002 | 6,253 | 1,155 | 1,590 | 154 | 465 | 88 | 3 | 824 | 0 | 620 | 1,286 | 6 |
| Jordan | 2007 | 10,180 | 1,965 | 2,519 | 255 | 930 | 150 | 7 | 1,427 | 0 | 771 | 2,110 | 2 |
| Jordan | 2009 | 10,929 | 2,108 | 2,301 | 315 | 1,210 | 121 | 4 | 1,347 | 0 | 712 | 2,660 | 149 |
| Jordan | 2012 | 13,378 | 2,402 | 2,438 | 328 | 1,243 | 118 | 56 | 1,837 | 26 | 555 | 3,210 | 1,163 |
| Jordan | 2017/18 | 8,301 | 1,873 | 2,139 | 188 | 816 | 88 | 53 | 473 | 0 | 153 | 2,512 | 0 |
| Jordan | 2023 | 7,682 | 1,550 | 1,737 | 252 | 760 | 104 | 128 | 204 | 3 | 129 | 2,798 | 3 |
| Moldova | 2005 | 4,631 | 452 | 905 | 9 | 925 | 103 | 2 | 521 | 0 | 244 | 1,258 | 49 |
| Morocco | 1992 | 4,046 | 2,869 | 252 | 33 | 108 | 71 | 0 | 0 | 0 | 368 | 272 | 30 |
| Morocco | 2003/4 | 10,149 | 6,144 | 493 | 428 | 244 | 64 | 0 | 1,470 | 0 | 597 | 670 | 8 |
| Türkiye | 1993 | 5,495 | 758 | 1,221 | 25 | 738 | 98 | 0 | 0 | 0 | 123 | 2,321 | 15 |
| Türkiye | 1998 | 4,848 | 671 | 1,023 | 43 | 737 | 137 | 0 | 0 | 0 | 84 | 2,020 | 33 |
| Türkiye | 2003/4 | 7,298 | 888 | 1,200 | 173 | 1,240 | 203 | 0 | 418 | 0 | 139 | 2,931 | 38 |
| Türkiye | 2018/19 | 3,349 | 331 | 474 | 106 | 970 | 217 | 0 | 0 | 0 | 18 | 1,207 | 16 |

| | | | | | | | | | | | | | |
|---|---|---|---|---|---|---|---|---|---|---|---|---|---|
| Ukraine | 2007 | 3,637 | 348 | 553 | 5 | 1,559 | 14 | 0 | 0 | 0 | 405 | 646 | 61 |
| Yemen | 2013 | 10,938 | 4,182 | 1,051 | 1,369 | 159 | 147 | 181 | 2,592 | 31 | 429 | 774 | 17 |
| **Central, South & Southeast Asia** | | **(814,380)** | **(185,793)** | **(48,018)** | **(100,552)** | **(163,073)** | **(91,754)** | **(12,372)** | **(12,650)** | **(504)** | **(95,719)** | **(96,206)** | **(2,513)** |
| Bangladesh | 1993/94 | 7,040 | 3,406 | 387 | 811 | 852 | 196 | 0 | 0 | 0 | 802 | 407 | 160 |
| Bangladesh | 1996/97 | 6,373 | 3,194 | 265 | 1,023 | 781 | 110 | 9 | 0 | 0 | 595 | 318 | 66 |
| Bangladesh | 1999/0 | 8,484 | 4,108 | 187 | 1,272 | 1,203 | 95 | 48 | 0 | 0 | 816 | 646 | 98 |
| Bangladesh | 2004 | 10,411 | 5,117 | 110 | 1,757 | 1,454 | 85 | 87 | 0 | 0 | 967 | 693 | 76 |
| Bangladesh | 2011 | 13,188 | 6,755 | 138 | 2,588 | 1,578 | 324 | 189 | 0 | 0 | 1,101 | 350 | 55 |
| Bangladesh | 2014 | 11,782 | 5,973 | 87 | 2,327 | 1,588 | 348 | 296 | 9 | 0 | 735 | 302 | 15 |
| Bangladesh | 2017/18 | 15,481 | 7,576 | 85 | 2,714 | 2,345 | 278 | 510 | 5 | 5 | 1,269 | 584 | 26 |
| Bangladesh | 2022 | 13,085 | 6,419 | 87 | 2,271 | 1,994 | 257 | 387 | 17 | 2 | 910 | 684 | 5 |
| Cambodia | 2010/11 | 6,332 | 2,264 | 289 | 1,440 | 346 | 155 | 68 | 3 | 0 | 407 | 1,350 | 6 |
| Cambodia | 2014 | 7,563 | 2,384 | 533 | 1,434 | 342 | 182 | 312 | 17 | 2 | 360 | 1,944 | 8 |
| Cambodia | 2021/22 | 8,792 | 3,878 | 482 | 1,351 | 187 | 175 | 353 | 13 | 4 | 233 | 1,991 | 0 |
| India | 2005/6 | 35,028 | 6,142 | 2,716 | 227 | 7,872 | 8,650 | 2 | 0 | 0 | 5,228 | 3,734 | 242 |
| India | 2015/16 | 165,429 | 29,568 | 9,930 | 1,602 | 42,893 | 37,682 | 0 | 977 | 86 | 22,081 | 19,755 | 95 |
| India | 2019/21 | 291,156 | 43,567 | 14,154 | 5,775 | 84,274 | 37,247 | 0 | 9,964 | 346 | 51,365 | 42,766 | 294 |
| Indonesia | 1991 | 12,117 | 3,923 | 2,114 | 3,651 | 458 | 232 | 624 | 0 | 0 | 396 | 362 | 96 |
| Indonesia | 1994 | 15,210 | 5,073 | 1,887 | 5,413 | 349 | 274 | 1,048 | 0 | 0 | 466 | 310 | 119 |
| Indonesia | 1997 | 16,211 | 5,431 | 1,170 | 6,847 | 260 | 258 | 1,204 | 0 | 0 | 394 | 307 | 63 |
| Indonesia | 2002/3 | 17,263 | 5,005 | 938 | 8,462 | 299 | 249 | 1,211 | 73 | 0 | 474 | 388 | 144 |
| Indonesia | 2007 | 21,049 | 5,967 | 658 | 11,268 | 486 | 259 | 852 | 56 | 0 | 503 | 742 | 148 |
| Indonesia | 2012 | 22,744 | 6,145 | 791 | 11,975 | 608 | 393 | 1,256 | 62 | 0 | 514 | 852 | 129 |
| Indonesia | 2017 | 24,880 | 5,654 | 1,147 | 12,076 | 894 | 618 | 1,791 | 116 | 0 | 759 | 1,686 | 114 |
| Kazakhstan | 1999 | 2,888 | 380 | 1,075 | 57 | 581 | 36 | 0 | 0 | 0 | 294 | 231 | 197 |
| Kyrgyz Republic | 2012 | 2,305 | 191 | 1,144 | 53 | 614 | 41 | 0 | 66 | 5 | 23 | 162 | 4 |
| Maldives | 2009 | 2,722 | 511 | 40 | 157 | 1,051 | 302 | 23 | 0 | 0 | 211 | 406 | 8 |
| Myanmar | 2015/16 | 5,874 | 1,848 | 187 | 3,357 | 93 | 184 | 88 | 13 | 11 | 30 | 56 | 7 |
| Nepal | 2011 | 5,874 | 1,165 | 122 | 1,800 | 1,131 | 321 | 108 | 0 | 1 | 119 | 903 | 8 |
| Nepal | 2016 | 7,654 | 1,587 | 163 | 1,963 | 1,196 | 265 | 344 | 2 | 0 | 164 | 1,793 | 5 |
| Nepal | 2022 | 8,404 | 1,403 | 121 | 2,404 | 989 | 287 | 827 | 28 | 1 | 188 | 2,075 | 3 |
| Pakistan | 2012/13 | 6,406 | 627 | 467 | 968 | 1,973 | 351 | 16 | 461 | 30 | 123 | 1,370 | 17 |
| Pakistan | 2017/18 | 4,579 | 438 | 253 | 632 | 1,570 | 324 | 65 | 39 | 0 | 138 | 1,109 | 8 |
| Philippines | 1993 | 4,721 | 1,512 | 312 | 21 | 200 | 342 | 0 | 0 | 0 | 1,011 | 1,230 | 74 |
| Philippines | 1998 | 6,087 | 1,588 | 404 | 482 | 331 | 205 | 0 | 6 | 0 | 1,060 | 1,292 | 103 |
| Philippines | 2003 | 5,920 | 1,998 | 315 | 698 | 366 | 250 | 1 | 91 | 5 | 819 | 1,258 | 88 |
| Philippines | 2022 | 10,006 | 3,711 | 317 | 1,354 | 458 | 418 | 632 | 347 | 0 | 387 | 2,344 | 19 |
| Tajikistan | 2012 | 1,950 | 237 | 1,037 | 165 | 257 | 22 | 4 | 71 | 5 | 5 | 144 | 2 |
| Tajikistan | 2017 | 2,265 | 236 | 1,207 | 120 | 386 | 33 | 17 | 134 | 1 | 3 | 124 | 0 |
| Vietnam | 1997 | 3,565 | 310 | 1,488 | 15 | 405 | 202 | 0 | 80 | 0 | 358 | 670 | 9 |
| Vietnam | 2002 | 3,542 | 502 | 1,211 | 22 | 409 | 104 | 0 | 0 | 0 | 411 | 868 | 2 |
| **Latin America & Caribbean** | | **(389,048)** | **(79,934)** | **(19,964)** | **(87,713)** | **(60,695)** | **(25,730)** | **(4,173)** | **(4,716)** | **(377)** | **(51,400)** | **(34,012)** | **(10,695)** |
| Bolivia | 1994 | 4,479 | 544 | 451 | 192 | 209 | 119 | 2 | 0 | 0 | 2,181 | 258 | 463 |
| Brazil | 1996 | 7,972 | 3,713 | 118 | 393 | 1,196 | 1,175 | 0 | 0 | 0 | 575 | 637 | 65 |
| Colombia | 1990 | 5,283 | 1,761 | 802 | 466 | 286 | 507 | 0 | 0 | 0 | 692 | 362 | 41 |
| Colombia | 1995 | 9,093 | 2,495 | 912 | 698 | 970 | 695 | 53 | 0 | 0 | 1,163 | 1,081 | 553 |

| | | | | | | | | | | | | | |
|---|---|---|---|---|---|---|---|---|---|---|---|---|---|
| Colombia | 2000 | 10,501 | 2,370 | 879 | 1,172 | 1,706 | 643 | 27 | 336 | 0 | 1,290 | 1,425 | 358 |
| Colombia | 2005 | 30,352 | 6,538 | 2,572 | 5,373 | 5,717 | 2,820 | 103 | 995 | 0 | 2,113 | 3,171 | 481 |
| Colombia | 2010 | 37,498 | 6,600 | 2,171 | 9,528 | 8,476 | 4,024 | 1,141 | 0 | 0 | 1,415 | 2,710 | 433 |
| Colombia | 2015/16 | 25,678 | 3,489 | 763 | 9,289 | 4,812 | 2,727 | 2,158 | 166 | 3 | 590 | 1,315 | 105 |
| Dominican Republic | 1991 | 3,736 | 1,691 | 188 | 12 | 251 | 779 | 9 | 0 | 0 | 324 | 361 | 72 |
| Dominican Republic | 1996 | 5,011 | 2,271 | 218 | 99 | 407 | 858 | 64 | 113 | 0 | 383 | 446 | 114 |
| Dominican Republic | 2002 | 14,473 | 6,622 | 491 | 1,050 | 1,033 | 2,392 | 107 | 483 | 0 | 656 | 931 | 659 |
| Guatemala | 1995 | 2,245 | 606 | 159 | 274 | 252 | 386 | 0 | 0 | 0 | 402 | 96 | 12 |
| Guatemala | 1998/99 | 1,616 | 422 | 77 | 312 | 136 | 245 | 0 | 0 | 0 | 298 | 90 | 9 |
| Guatemala | 2014/15 | 12,121 | 1,125 | 236 | 4,753 | 1,392 | 1,338 | 358 | 169 | 4 | 1,275 | 1,372 | 17 |
| Honduras | 2011/12 | 20,898 | 4,590 | 1,048 | 6,497 | 3,343 | 1,248 | 4 | 68 | 34 | 990 | 2,683 | 366 |
| Nicaragua | 1998 | 7,588 | 3,041 | 1,108 | 993 | 573 | 1,275 | 3 | 0 | 0 | 265 | 160 | 138 |
| Paraguay | 1990 | 4,206 | 1,392 | 224 | 740 | 271 | 151 | 0 | 0 | 0 | 499 | 179 | 681 |
| Peru | 1991/92 | 10,497 | 1,761 | 1,332 | 722 | 666 | 319 | 0 | 0 | 0 | 4,036 | 779 | 501 |
| Peru | 1996 | 21,524 | 3,424 | 2,641 | 2,925 | 1,640 | 863 | 31 | 0 | 0 | 6,832 | 1,217 | 1,248 |
| Peru | 2000 | 20,589 | 3,363 | 1,420 | 5,474 | 2,156 | 1,200 | 54 | 618 | 0 | 4,655 | 907 | 280 |
| Peru | 2004/6 | 17,140 | 2,811 | 482 | 4,402 | 2,727 | 247 | 7 | 441 | 2 | 3,919 | 1,095 | 743 |
| Peru | 2007/8 | 22,021 | 3,868 | 413 | 5,831 | 3,798 | 279 | 13 | 538 | 8 | 4,451 | 1,628 | 795 |
| Peru | 2009 | 23,861 | 3,904 | 387 | 6,873 | 4,295 | 335 | 9 | 151 | 326 | 285 | 4,517 | 674 |
| Peru | 2010 | 23,009 | 3,691 | 307 | 6,522 | 4,484 | 367 | 11 | 263 | 0 | 4,055 | 2,072 | 669 |
| Peru | 2011 | 22,715 | 3,751 | 278 | 6,288 | 4,751 | 381 | 8 | 176 | 0 | 3,804 | 2,116 | 601 |
| Peru | 2012 | 24,942 | 4,091 | 287 | 6,835 | 5,148 | 357 | 11 | 199 | 0 | 4,252 | 2,404 | 617 |
| **Total** | | **(1,681,658)** | **(375,664)** | **(129,492)** | **(299,970)** | **(267,268)** | **(123,525)** | **(55,814)** | **(35,680)** | **(1,580)** | **(168,466)** | **(178,470)** | **(19,024)** |

* Final analysis excluded methods with less than 100 episodes.
