# Supplementary material for: Contraceptive discontinuation, switching, abandonment and their reproductive consequences: An analysis of 1,539,071 episodes of reversible method use contributed from 61 countries that participated in DHS: Population base-analysis
Source: PLOS Glob Public Health. 2025 Oct 31;5(10):e0005174. doi: 10.1371/journal.pgph.0005174 (PMC12578211; doi:10.1371/journal.pgph.0005174)
Supplement: S6 Table — (PDF) [file pgph.0005174.s017.pdf]

S6 Table : Education level, Human Development Index (HDI), unmet need for family planning, and method skewness

Region<br>Country	Survey year	Sample	Percent of women with at least secondar	Human Development Index (HDI)	Contraceptive Prevalence	Unmet need for family planning	Method skewness
			Country specific factors				
**Sub-Saharan Africa**							
Angola	2015/16	AW	36.6	0.58	13.3	28.5	43.5
Benin	2017/18	AW	25.4	0.53	14.4	26.1	31.5
Burkina Faso	2010	AW	12.9	0.37	15.3	20.4	33.2
Burkina Faso	2021	AW	27.7	0.45	30.1	12.9	42.8
Burundi	2010/11	AW	17.1	0.41	13.4	20.2	47.2
Comoros	2012	AW	51.8	0.53	13.7	20.6	27.4
Côte d'Ivoire	2021	AW	27.8	0.55	22.0	18.4	22.5
Ethiopia	2005	AW	18.8	0.35	10.3	24.0	65.6
Ethiopia	2016	AW	21.9	0.47	25.3	15.2	62.3
Gabon	2019/21	AW	72.9	0.71	22.7	24.9	53.5
Gambia	2013	AW	36.3	0.47	7.1	17.3	42.8
Gambia	2019/20	AW	41.6	0.50	13.4	16.2	41.6
Ghana	2014	AW	57.1	0.60	22.8	21.3	26.4
Ghana	2022/23	AW	62.7	0.63	31.5	15.9	18.8
Guinea	2018	AW	19.0	0.46	11.8	17.7	23.5
Kenya	1998	AW	27.3	0.48	29.9	20.9	29.5
Kenya	2003	AW	31.2	0.50	28.4	19.5	36.9
Kenya	2014	AW	36.3	0.56	42.6	12.8	43.9
Kenya	2022	AW	51.4	0.57	46.6	9.9	29.2
Lesotho	2014	AW	58.5	0.50	48.9	12.6	38.9
Liberia	2013	AW	25.6	0.47	21.7	27.7	57.3
Liberia	2019/20	AW	33.4	0.48	26.2	26.7	57.9
Madagascar	2021	AW	40.1	0.50	40.1	12.0	53.2
Malawi	2004/5	AW	14.4	0.41	25.7	24.4	54.1
Malawi	2015/16	AW	27.5	0.49	46.0	15.1	49.0
Mali	2012/13	AW	16.2	0.41	10.0	23.3	38.2
Mali	2018	AW	20.7	0.43	16.2	21.1	41.7

Mozambique	2011	AW	23.3	0.41	12.3	20.8	34.9
Mozambique	2022/23	AW	35.8	0.44	25.8	22.3	40.3
Namibia	2013	AW	73.1	0.61	50.2	11.7	42.2
Niger	2012	AW	12.3	0.35	12.5	14.3	39.9
Nigeria	2013	AW	46.5	0.50	16.0	12.7	28.4
Nigeria	2018	AW	50.3	0.53	14.3	15.2	17.6
Rwanda	2010/11	AW	17.1	0.49	28.6	12.7	50.9
Rwanda	2014/15	AW	25.1	0.51	30.9	12.6	45.7
Rwanda	2019/20	AW	32.7	0.53	38.6	9.1	42.8
Senegal	2010/11	AW	17.9	0.47	9.6	20.9	38.5
Senegal	2015	AW	24.0	0.51	16.9	17.2	34.6
Senegal	2016	AW	25.9	0.51	18.0	16.1	32.0
Senegal	2018	AW	27.8	0.51	19.4	13.8	33.2
Senegal	2019	AW	28.8	0.51	18.8	14.7	37.4
Senegal	2023	AW	34.1	0.51	18.0	12.7	41.0
Sierra Leone	2013	AW	31.5	0.44	22.1	20.5	44.4
South Africa	2016	AW	87.6	0.72	48.2	11.9	33.0
Tanzania	2004/5	AW	13.7	0.44	22.5	19.5	30.9
Tanzania	2015/16	AW	27.3	0.52	32.4	16.8	30.6
Tanzania	2022	AW	35.8	0.55	30.9	16.2	35.8
Uganda	2011	AW	29.1	0.51	23.6	24.5	45.3
Zambia	2013/14	AW	45.0	0.55	35.1	16.7	39.2
Zambia	2018/19	AW	46.2	0.57	35.4	16.1	50.9
Zimbabwe	1994	AW	40.1	0.49	35.1	14.3	67.3
Zimbabwe	1999	AW	50.0	0.46	37.7	12.1	63.1
Zimbabwe	2005/6	AW	62.4	0.48	40.1	10.3	67.1
Zimbabwe	2010/11	AW	68.7	0.51	41.3	11.0	66.1
Zimbabwe	2015	AW	75.0	0.58	48.6	7.9	55.6
**North Africa Western Asia and Europe**							
Albania	2017/18	AW	54.4	0.80	33.2	11.3	91.3
Armenia	2000	AW	99.5	0.66	39.0	11.6	52.5
Armenia	2005	AW	99.5	0.70	33.1	12.1	51.8
Armenia	2010	AW	93.9	0.75	33.9	8.4	44.2
Armenia	2015/16	AW	93.3	0.77	36.7	8.0	43.3

Azerbaijan	2006	AW	97.1	0.69	32.0	9.6	63.4
Egypt	1992/93	EMW	27.0	0.58	47.1	22.9	59.3
Egypt	1995/96	EMW	30.1	0.60	47.9	20.2	62.6
Egypt	2000	EMW	39.4	0.63	56.1	13.7	63.3
Egypt	2003	EMW	43.0	0.65	60.0	11.8	61.0
Egypt	2005	EMW	48.7	0.65	59.2	12.3	61.5
Egypt	2008	EMW	54.1	0.67	60.3	11.6	59.8
Egypt	2014	EMW	67.7	0.70	58.5	12.6	51.4
Jordan	1990	EMW	52.9	0.62	40.0	26.5	38.1
Jordan	1997	EMW	73.7	0.66	52.6	20.1	43.9
Jordan	2002	EMW	78.2	0.69	55.8	14.9	42.3
Jordan	2007	EMW	83.7	0.71	56.8	13.8	39.0
Jordan	2009	EMW	85.8	0.72	59.3	13.4	37.9
Jordan	2012	EMW	87.8	0.72	61.2	11.7	34.8
Jordan	2017/18	EMW	88.2	0.72	51.8	14.2	40.1
Jordan	2023	EMW	88.2	0.71	60.1	10.8	33.9
Moldova	2005	AW	99.3	0.69	49.8	8.6	35.6
Morocco	1992	AW	19.8	0.46	22.9	13.0	67.7
Morocco	2003/4	AW	27.6	0.55	33.3	6.2	63.6
Türkiye	1993	EMW	17.3	0.62	62.6	14.6	41.8
Türkiye	1998	AW	29.5	0.66	44.2	9.7	38.2
Türkiye	2003/4	EMW	26.4	0.69	71.0	6.5	37.1
Türkiye	2018/19	AW	60.1	0.84	70.0	4.0	28.8
Ukraine	2007	AW	99.8	0.74	50.9	6.8	41.7
Yemen	2013	AW	19.6	0.49	33.5	27.0	34.7
**Central, South & Southeast Asia**							
Bangladesh	1993/94	EMW	99.5	0.42	44.9	21.6	38.6
Bangladesh	1996/97	EMW	99.6	0.45	49.8	19.7	41.8
Bangladesh	1999/0	EMW	95.0	0.48	54.3	18.2	42.4
Bangladesh	2004	EMW	94.4	0.51	58.5	15.0	44.8
Bangladesh	2011	EMW	15.7	0.56	61.2	13.5	44.5
Bangladesh	2014	EMW	18.6	0.59	62.4	12.0	43.2
Bangladesh	2017/18	EMW	28.2	0.62	61.9	12.0	41.0
Bangladesh	2022	EMW	31.8	0.67	64.0	10.0	42.9

Cambodia	2010/11	AW	44.1	0.54	31.4	10.6	30.3
Cambodia	2014	AW	47.2	0.57	38.5	8.5	31.0
Cambodia	2021/22	AW	52.6	0.59	43.1	8.2	41.0
India	2005/6	AW	60.0	0.53	43.8	10.4	67.4
India	2015/16	AW	36.0	0.63	40.8	9.4	68.3
India	2019/21	AW	42.8	0.65	50.1	6.6	58.0
Indonesia	1991	EMW	46.0	0.53	49.7	17.0	29.7
Indonesia	1994	EMW	53.7	0.55	54.7	15.3	31.2
Indonesia	1997	EMW	59.3	0.57	57.4	13.6	36.8
Indonesia	2002/3	EMW	65.2	0.61	60.3	13.2	46.0
Indonesia	2007	EMW	23.8	0.64	61.4	13.1	51.7
Indonesia	2012	AW	28.9	0.68	45.7	8.4	51.3
Indonesia	2017	AW	33.5	0.71	46.0	7.7	38.8
Kazakhstan	1999	AW	42.0	0.68	48.0	8.6	62.0
Kyrgyz Republic	2012	AW	49.6	0.67	24.4	12.1	59.9
Maldives	2009	EMW	66.3	0.68	34.7	28.6	30.3
Myanmar	2015/16	AW	73.5	0.56	31.6	9.8	52.7
Nepal	2011	AW	36.5	0.55	38.2	20.9	31.1
Nepal	2016	AW	47.8	0.59	40.8	18.2	28.5
Nepal	2022	AW	44.6	0.60	43.7	15.7	24.0
Pakistan	2012/13	EMW	50.0	0.52	35.4	20.1	24.9
Pakistan	2017/18	EMW	41.0	0.54	34.2	17.3	26.7
Philippines	1993	AW	30.3	0.61	24.2	18.1	30.4
Philippines	1998	AW	35.4	0.63	28.9	14.9	22.2
Philippines	2003	AW	65.1	0.65	31.6	14.7	26.7
Philippines	2022	AW	68.7	0.69	34.1	7.7	32.8
Tajikistan	2012	AW	74.5	0.64	18.9	15.4	66.4
Tajikistan	2017	AW	88.2	0.67	21.3	16.5	62.2
Vietnam	1997	EMW	67.2	0.56	75.3	8.4	51.1
Vietnam	2002	EMW	66.6	0.60	78.5	6.6	48.1
**Latin America & Caribbean**							
Bolivia	1994	AW	50.0	0.58	30.1	19.2	48.8
Brazil	1996	AW	60.1	0.65	55.4	8.5	49.4
Colombia	1990	AW	60.1	0.61	39.9	8.0	31.9

Colombia	1995	AW	58.9	0.64	48.1	7.8	35.1
Colombia	2000	AW	63.7	0.67	52.8	7.0	33.2
Colombia	2005	AW	69.2	0.70	56.4	6.8	36.4
Colombia	2010	AW	72.5	0.73	61.2	6.5	39.5
Colombia	2015/16	AW	78.8	0.75	64.9	6.0	38.6
Dominican Republic	1991	AW	37.9	0.58	36.8	11.6	68.9
Dominican Republic	1996	AW	39.1	0.62	44.6	9.8	64.1
Dominican Republic	2002	AW	45.5	0.66	51.2	9.1	64.9
Guatemala	1995	AW	16.7	0.52	21.4	18.9	46.7
Guatemala	1998/99	AW	18.2	0.54	26.6	18.5	45.0
Guatemala	2014/15	AW	39.5	0.63	39.4	9.2	35.5
Honduras	2011/12	AW	40.6	0.60	48.8	7.7	32.5
Nicaragua	1998	AW	39.5	0.55	40.8	11.9	45.6
Paraguay	1990	AW	32.7	0.60	32.7	11.2	26.9
Peru	1991/92	AW	61.9	0.63	35.7	12.7	35.0
Peru	1996	AW	59.4	0.65	40.9	11.4	28.1
Peru	2000	AW	60.6	0.68	44.0	9.3	21.1
Peru	2004/6	AW	66.5	0.70	45.8	7.9	24.6
Peru	2007/8	AW	68.1	0.71	48.0	8.4	22.6
Peru	2009	AW	68.6	0.72	49.2	7.5	23.5
Peru	2010	AW	69.0	0.73	50.1	7.0	22.7
Peru	2011	AW	69.4	0.73	50.9	6.6	22.9
Peru	2012	AW	71.4	0.74	51.5	6.4	23.2

AW=All women. EMW=Ever-married women

Contraceptive prevalence and Unmet need for family planning are based on married and sexually active unmarried women in AW samples

ICF, 2015. The DHS Program STATcompiler. Funded by USAID. http://www.statcompiler.com. September 23 2024
