# Supplementary material for: Contraceptive discontinuation, switching, abandonment and their reproductive consequences: An analysis of 1,539,071 episodes of reversible method use contributed from 61 countries that participated in DHS: Population base-analysis
Source: PLOS Glob Public Health. 2025 Oct 31;5(10):e0005174. doi: 10.1371/journal.pgph.0005174 (PMC12578211; doi:10.1371/journal.pgph.0005174)
Supplement: S7 Table — (PDF) [file pgph.0005174.s018.pdf]

S7 Table : Medians of discontinuation rates at 12 months, by method and reason

	All reasons	Became pregnant while using	Side effects including health concerns	Other method-related	Wanted pregnancy/ no need	Other/not stated
Oral contraceptives	48.0	3.4	13.5	7.9	14.3	3.9
IUDs	15.5	1.2	7.9	1.2	3.3	1.1
Injectables	45.0	1.1	18.0	5.6	11.9	3.8
Condom	39.3	2.8	1.6	9.4	15.8	6.4
Implants	14.5	0.4	8.0	1.0	3.4	1.0
Periodic Abstinence	29.8	7.9	0.5	6.1	11.4	2.8
Withdrawal	37.1	7.7	0.5	9.4	13.4	4.0
