# Supplementary material for: Contraceptive discontinuation, switching, abandonment and their reproductive consequences: An analysis of 1,539,071 episodes of reversible method use contributed from 61 countries that participated in DHS: Population base-analysis
Source: PLOS Glob Public Health. 2025 Oct 31;5(10):e0005174. doi: 10.1371/journal.pgph.0005174 (PMC12578211; doi:10.1371/journal.pgph.0005174)
Supplement: S8 Table — (PDF) [file pgph.0005174.s019.pdf]

S8.1 Table: 12-month overall estimated probabilities of discontinuation and reason-specific estimated cumulative incidence of discontinuation per 100 episodes of use

Oral contraceptives (OCs)

	No. of episodes	All reasons		Became pregnant while using		Side effect/health concerns		Other method-related		Wanted pregnancy/no need		Other/not stated	
		Rate	95%CI	Rate	95%CI	Rate	95%CI	Rate	95%CI	Rate	95%CI	Rate	95%CI
**Sub-Saharan Africa**													
Angola (2015/16)	448	50.8	(46.4 - 55.5)	3.5	(2.2 - 5.4)	12.4	(9.7 - 15.6)	7.8	(5.7 - 10.3)	8.8	(6.5 - 11.5)	18.3	(15.0 - 21.9)
Benin (2017/18)	512	51.3	(46.8 - 56.0)	2.5	(1.4 - 4.3)	10.8	(8.2 - 13.8)	8.8	(6.4 - 11.5)	20.9	(17.3 - 24.8)	8.4	(6.1 - 11.1)
Burkina Faso (2010)	786	24.9	(21.7 - 28.4)	1.9	(1.1 - 3.3)	7.3	(5.5 - 9.5)	2.7	(1.7 - 4.2)	9.9	(7.7 - 12.4)	2.9	(1.8 - 4.5)
Burkina Faso (2021)	864	45.1	(41.6 - 48.9)	1.3	(0.6 - 2.3)	7.0	(5.4 - 9.0)	8.8	(6.9 - 11.0)	17.7	(15.0 - 20.5)	10.4	(8.3 - 12.7)
Burundi (2010/11)	328	58.5	(52.7 - 64.4)	7.6	(4.9 - 11.2)	13.3	(9.7 - 17.6)	14.7	(10.9 - 19.1)	12.1	(8.6 - 16.2)	10.7	(7.4 - 14.6)
Comoros (2012)	131	18.4	(12.3 - 27.1)	2.0	(0.4 - 6.3)	1.4	(0.2 - 5.2)	4.2	(1.5 - 9.1)	5.0	(2.0 - 10.2)	5.8	(2.4 - 11.4)
Côte d'Ivoire (2021)	994	42.8	(39.5 - 46.2)	0.4	(0.1 - 1.1)	9.8	(7.9 - 11.9)	9.4	(7.6 - 11.5)	16.7	(14.2 - 19.2)	6.4	(4.9 - 8.2)
Ethiopia (2005)	748	62.8	(59.0 - 66.6)	2.8	(1.7 - 4.3)	25.8	(22.5 - 29.2)	9.4	(7.3 - 11.8)	16.2	(13.5 - 19.2)	8.6	(6.6 - 10.8)
Ethiopia (2016)	560	70.2	(65.8 - 74.5)	4.6	(3.0 - 6.8)	13.3	(10.4 - 16.7)	28.4	(24.4 - 32.5)	19.9	(16.3 - 23.6)	4.0	(2.5 - 6.1)
Gabon (2019/21)	326	50.1	(45.1 - 55.2)	1.5	(0.6 - 3.0)	14.9	(11.6 - 18.7)	9.4	(6.8 - 12.6)	13.5	(10.3 - 17.1)	10.6	(7.9 - 13.8)
Gambia (2013)	258	37.1	(31.4 - 43.6)	2.1	(0.8 - 4.6)	6.8	(4.1 - 10.4)	8.5	(5.4 - 12.4)	10.1	(6.7 - 14.4)	9.5	(6.4 - 13.5)
Gambia (2019/20)	388	55.3	(50.1 - 60.7)	2.0	(0.9 - 4.0)	15.1	(11.6 - 19.1)	10.9	(8.0 - 14.4)	20.3	(16.2 - 24.7)	6.9	(4.6 - 9.9)
Ghana (2014)	546	31.5	(27.6 - 36.0)	6.2	(4.3 - 8.6)	8.4	(6.2 - 11.1)	3.9	(2.5 - 6.0)	11.4	(8.7 - 14.5)	1.6	(0.7 - 3.0)
Ghana (2022/23)	1,008	50.4	(47.1 - 53.8)	3.4	(2.3 - 4.7)	17.7	(15.2 - 20.3)	6.0	(4.5 - 7.6)	19.2	(16.7 - 21.9)	4.2	(3.0 - 5.7)
Guinea (2018)	540	74.7	(70.9 - 78.3)	1.3	(0.6 - 2.6)	33.4	(29.5 - 37.3)	5.2	(3.5 - 7.3)	28.8	(25.1 - 32.6)	6.0	(4.2 - 8.1)
Kenya (1998)	927	37.0	(33.9 - 40.4)	2.2	(1.4 - 3.4)	17.3	(14.9 - 19.9)	5.4	(4.0 - 7.0)	6.3	(4.8 - 8.0)	5.8	(4.4 - 7.5)
Kenya (2003)	953	48.8	(45.5 - 52.2)	4.0	(2.8 - 5.4)	24.5	(21.8 - 27.4)	7.9	(6.2 - 9.8)	7.4	(5.8 - 9.2)	5.0	(3.7 - 6.6)
Kenya (2014)	1,419	46.7	(44.2 - 49.3)	5.2	(4.2 - 6.4)	16.2	(14.4 - 18.1)	10.1	(8.7 - 11.7)	10.4	(8.9 - 12.0)	4.8	(3.8 - 6.0)
Kenya (2022)	1,263	56.9	(54.3 - 59.6)	2.5	(1.8 - 3.5)	15.5	(13.7 - 17.4)	18.0	(16.0 - 20.0)	18.8	(16.8 - 20.9)	2.1	(1.4 - 3.0)
Lesotho (2014)	760	30.6	(27.3 - 34.1)	2.7	(1.6 - 4.1)	9.0	(7.0 - 11.2)	8.4	(6.5 - 10.6)	6.7	(5.0 - 8.7)	3.9	(2.6 - 5.5)
Liberia (2013)	716	36.5	(32.6 - 40.8)	5.0	(3.4 - 7.0)	13.2	(10.6 - 16.2)	7.5	(5.5 - 9.9)	3.6	(2.2 - 5.5)	7.2	(5.2 - 9.5)
Liberia (2019/20)	501	48.2	(43.4 - 53.2)	2.1	(1.0 - 3.8)	18.9	(15.3 - 22.9)	14.1	(10.9 - 17.6)	7.8	(5.5 - 10.7)	5.3	(3.4 - 7.8)
Madagascar (2021)	1,139	60.1	(57.2 - 63.0)	3.0	(2.1 - 4.2)	15.2	(13.2 - 17.3)	16.4	(14.3 - 18.6)	14.5	(12.5 - 16.7)	11.0	(9.3 - 12.9)
Malawi (2004/5)	468	52.9	(48.1 - 57.7)	7.0	(4.8 - 9.7)	21.5	(17.7 - 25.5)	6.8	(4.7 - 9.5)	12.4	(9.4 - 15.8)	5.2	(3.3 - 7.6)
Malawi (2015/16)	963	64.0	(60.7 - 67.3)	2.4	(1.5 - 3.6)	21.3	(18.6 - 24.0)	16.2	(13.9 - 18.8)	16.6	(14.2 - 19.2)	7.5	(5.8 - 9.4)
Mali (2012/13)	547	47.0	(42.5 - 51.7)	5.5	(3.7 - 7.9)	10.2	(7.7 - 13.2)	5.2	(3.5 - 7.5)	18.6	(15.2 - 22.4)	7.4	(5.2 - 9.9)
Mali (2018)	447	49.2	(44.5 - 54.2)	1.0	(0.3 - 2.4)	8.5	(6.1 - 11.4)	7.5	(5.3 - 10.2)	22.6	(18.7 - 26.8)	9.6	(7.1 - 12.6)
Mozambique (2011)	1,225	49.1	(45.7 - 52.7)	3.2	(2.2 - 4.6)	15.3	(12.9 - 17.9)	3.6	(2.5 - 5.0)	16.4	(13.9 - 19.1)	10.5	(8.5 - 12.8)
Mozambique (2022/23)	961	41.2	(37.6 - 45.1)	2.2	(1.3 - 3.5)	6.0	(4.4 - 7.9)	9.5	(7.5 - 11.8)	15.1	(12.5 - 17.9)	8.5	(6.6 - 10.7)
Namibia (2013)	587	29.2	(25.4 - 33.5)	3.1	(1.8 - 5.0)	9.9	(7.5 - 12.8)	7.0	(5.0 - 9.5)	5.5	(3.7 - 7.8)	3.7	(2.3 - 5.6)
Niger (2012)	1,626	47.7	(44.9 - 50.7)	0.9	(0.5 - 1.6)	4.0	(3.0 - 5.2)	4.3	(3.2 - 5.5)	27.0	(24.5 - 29.6)	11.5	(9.8 - 13.4)
Nigeria (2013)	1,087	27.4	(24.6 - 30.4)	5.4	(4.1 - 7.0)	3.7	(2.6 - 5.1)	2.3	(1.5 - 3.5)	12.7	(10.6 - 15.0)	3.2	(2.2 - 4.5)
Nigeria (2018)	1,025	52.0	(48.9 - 55.2)	7.0	(5.5 - 8.7)	13.7	(11.6 - 15.9)	10.5	(8.6 - 12.5)	18.3	(15.9 - 20.8)	2.6	(1.8 - 3.7)

Rwanda (2010/11)	1,038	48.4	(45.1 - 51.7)	4.3	(3.1 - 5.7)	19.8	(17.3 - 22.5)	13.9	(11.8 - 16.2)	4.8	(3.6 - 6.4)	5.5	(4.2 - 7.1)
Rwanda (2014/15)	1,285	42.8	(40.1 - 45.7)	4.1	(3.1 - 5.3)	17.4	(15.3 - 19.6)	11.4	(9.7 - 13.2)	7.5	(6.1 - 9.1)	2.4	(1.7 - 3.4)
Rwanda (2019/20)	1,222	52.0	(49.1 - 54.9)	5.3	(4.1 - 6.7)	16.2	(14.2 - 18.4)	20.4	(18.1 - 22.8)	7.3	(5.9 - 9.0)	2.7	(1.9 - 3.8)
Senegal (2010/11)	688	53.7	(50.3 - 57.2)	5.7	(4.2 - 7.4)	14.7	(12.4 - 17.2)	7.6	(5.9 - 9.5)	7.4	(5.8 - 9.4)	18.4	(15.8 - 21.0)
Senegal (2015)	491	48.4	(44.3 - 52.7)	3.9	(2.5 - 5.8)	13.1	(10.5 - 16.0)	7.9	(5.8 - 10.3)	17.8	(14.8 - 21.0)	5.7	(4.0 - 7.9)
Senegal (2016)	508	47.6	(43.7 - 51.6)	2.4	(1.4 - 3.9)	6.0	(4.3 - 8.0)	7.7	(5.8 - 10.0)	22.9	(19.7 - 26.3)	8.5	(6.5 - 10.9)
Senegal (2018)	494	46.9	(42.6 - 51.3)	2.4	(1.3 - 4.0)	12.9	(10.2 - 16.0)	3.7	(2.3 - 5.5)	24.2	(20.5 - 28.0)	3.7	(2.3 - 5.6)
Senegal (2019)	402	41.3	(36.7 - 46.2)	3.1	(1.7 - 5.1)	9.4	(6.8 - 12.4)	5.6	(3.7 - 8.1)	18.2	(14.7 - 22.0)	5.0	(3.1 - 7.4)
Senegal (2023)	582	53.1	(49.4 - 56.8)	3.5	(2.3 - 5.0)	14.5	(12.1 - 17.2)	3.2	(2.1 - 4.6)	21.3	(18.4 - 24.3)	10.6	(8.5 - 13.0)
Sierra Leone (2013)	1,200	27.3	(24.7 - 30.1)	3.5	(2.5 - 4.7)	10.3	(8.6 - 12.2)	4.8	(3.6 - 6.2)	5.8	(4.5 - 7.4)	2.9	(2.0 - 4.0)
South Africa (2016)	385	31.4	(26.9 - 36.4)	3.8	(2.2 - 6.1)	10.4	(7.6 - 13.8)	6.9	(4.7 - 9.6)	5.7	(3.6 - 8.4)	4.6	(2.7 - 7.1)
Tanzania (2004/5)	1,226	42.4	(39.6 - 45.4)	4.0	(3.0 - 5.3)	18.5	(16.3 - 20.8)	6.4	(5.0 - 7.9)	10.5	(8.8 - 12.4)	3.1	(2.2 - 4.2)
Tanzania (2015/16)	958	34.8	(31.8 - 37.9)	2.7	(1.8 - 3.9)	15.4	(13.1 - 17.8)	5.0	(3.7 - 6.5)	10.1	(8.3 - 12.2)	1.6	(0.9 - 2.6)
Tanzania (2022)	535	45.3	(40.9 - 50.0)	2.0	(1.0 - 3.6)	15.8	(12.7 - 19.3)	10.6	(8.0 - 13.6)	13.8	(10.9 - 17.1)	3.1	(1.8 - 5.0)
Uganda (2011)	498	55.4	(50.9 - 60.0)	9.6	(7.2 - 12.5)	21.5	(17.9 - 25.2)	8.6	(6.3 - 11.4)	11.2	(8.5 - 14.2)	4.5	(2.9 - 6.6)
Zambia (2013/14)	2,514	34.1	(32.2 - 36.1)	4.0	(3.3 - 4.9)	11.0	(9.8 - 12.2)	5.7	(4.8 - 6.6)	9.0	(7.8 - 10.1)	4.5	(3.7 - 5.4)
Zambia (2018/19)	1,344	52.4	(49.8 - 55.2)	3.5	(2.6 - 4.6)	19.8	(17.8 - 22.0)	9.6	(8.1 - 11.3)	14.1	(12.3 - 16.0)	5.3	(4.2 - 6.7)
Zimbabwe (1994)	2,408	16.1	(14.7 - 17.8)	2.2	(1.6 - 2.9)	4.6	(3.8 - 5.5)	1.8	(1.3 - 2.4)	5.4	(4.5 - 6.4)	2.2	(1.6 - 2.8)
Zimbabwe (1999)	2,108	17.7	(16.1 - 19.5)	1.8	(1.3 - 2.5)	4.5	(3.6 - 5.5)	4.0	(3.2 - 4.9)	4.9	(4.0 - 5.9)	2.5	(1.9 - 3.3)
Zimbabwe (2005/6)	3,470	17.3	(16.0 - 18.7)	2.2	(1.7 - 2.7)	3.0	(2.5 - 3.6)	3.3	(2.7 - 4.0)	7.1	(6.3 - 8.1)	1.7	(1.3 - 2.2)
Zimbabwe (2010/11)	3,369	21.8	(20.3 - 23.3)	2.6	(2.1 - 3.3)	4.8	(4.1 - 5.6)	3.6	(3.0 - 4.4)	8.2	(7.2 - 9.2)	2.5	(2.0 - 3.1)
Zimbabwe (2015)	3,898	21.5	(20.2 - 22.8)	2.7	(2.2 - 3.3)	5.1	(4.4 - 5.8)	5.1	(4.5 - 5.9)	7.7	(6.8 - 8.5)	0.9	(0.6 - 1.2)
**North Africa Western Asia and Europe**													
Azerbaijan (2006)	104	50.7	(41.9 - 60.1)	18.8	(12.3 - 26.4)	16.7	(10.6 - 24.0)	1.7	(0.3 - 5.6)	7.5	(3.7 - 13.2)	5.9	(2.6 - 11.1)
Egypt (1992/93)	2,515	43.1	(41.1 - 45.1)	8.8	(7.7 - 10.0)	20.1	(18.5 - 21.8)	1.7	(1.2 - 2.3)	11.1	(9.9 - 12.5)	1.3	(0.9 - 1.9)
Egypt (1995/96)	3,011	48.2	(46.4 - 50.1)	6.6	(5.7 - 7.6)	21.0	(19.5 - 22.5)	2.5	(2.0 - 3.1)	15.2	(13.9 - 16.5)	3.0	(2.4 - 3.6)
Egypt (2000)	2,615	50.3	(48.3 - 52.4)	6.2	(5.2 - 7.2)	21.8	(20.1 - 23.5)	3.7	(3.0 - 4.5)	16.5	(15.0 - 18.1)	2.2	(1.6 - 2.8)
Egypt (2003)	1,673	47.7	(45.1 - 50.3)	5.8	(4.7 - 7.1)	18.4	(16.5 - 20.5)	5.3	(4.2 - 6.5)	16.8	(15.0 - 18.8)	1.3	(0.8 - 2.0)
Egypt (2005)	4,207	51.9	(50.2 - 53.5)	6.8	(6.0 - 7.6)	19.0	(17.7 - 20.3)	6.7	(5.9 - 7.6)	17.1	(15.9 - 18.4)	2.3	(1.8 - 2.8)
Egypt (2008)	3,161	42.0	(40.2 - 44.0)	6.2	(5.3 - 7.2)	13.1	(11.9 - 14.5)	4.4	(3.7 - 5.2)	16.5	(15.1 - 17.9)	1.8	(1.4 - 2.4)
Egypt (2014)	6,431	43.4	(42.1 - 44.7)	7.7	(7.0 - 8.4)	11.8	(11.0 - 12.7)	4.3	(3.7 - 4.8)	18.9	(17.9 - 19.9)	0.7	(0.5 - 0.9)
Jordan (1990)	1,128	65.0	(62.1 - 67.8)	8.5	(6.9 - 10.2)	30.5	(27.8 - 33.3)	3.3	(2.4 - 4.5)	14.6	(12.6 - 16.7)	8.0	(6.5 - 9.7)
Jordan (1997)	1,311	68.6	(66.0 - 71.2)	9.8	(8.3 - 11.6)	31.8	(29.2 - 34.4)	8.4	(7.0 - 10.1)	15.5	(13.6 - 17.6)	3.1	(2.2 - 4.1)
Jordan (2002)	1,155	57.6	(54.6 - 60.6)	9.7	(8.0 - 11.5)	24.2	(21.7 - 26.8)	8.1	(6.6 - 9.8)	13.7	(11.7 - 15.8)	1.9	(1.3 - 2.9)
Jordan (2007)	1,965	47.7	(45.5 - 49.9)	5.8	(4.8 - 6.9)	16.9	(15.3 - 18.6)	3.7	(3.0 - 4.6)	20.5	(18.7 - 22.3)	0.8	(0.4 - 1.2)
Jordan (2009)	2,108	52.8	(50.6 - 55.1)	8.2	(7.0 - 9.5)	22.0	(20.2 - 23.8)	5.6	(4.7 - 6.7)	16.0	(14.3 - 17.6)	1.1	(0.7 - 1.6)
Jordan (2012)	2,402	49.3	(47.2 - 51.4)	6.6	(5.6 - 7.7)	18.0	(16.5 - 19.7)	7.7	(6.7 - 8.9)	14.9	(13.4 - 16.4)	2.1	(1.5 - 2.7)
Jordan (2017/18)	1,873	37.4	(35.1 - 39.8)	2.1	(1.5 - 2.9)	4.4	(3.5 - 5.5)	12.0	(10.5 - 13.6)	17.0	(15.2 - 18.8)	1.9	(1.3 - 2.6)
Jordan (2023)	1,550	38.8	(36.2 - 41.5)	3.6	(2.7 - 4.7)	12.4	(10.7 - 14.3)	5.7	(4.6 - 7.1)	16.3	(14.3 - 18.4)	0.8	(0.4 - 1.3)
Moldova (2005)	452	51.4	(46.5 - 56.5)	5.1	(3.2 - 7.6)	11.9	(8.9 - 15.3)	10.1	(7.4 - 13.3)	16.1	(12.7 - 20.0)	8.1	(5.7 - 11.1)
Morocco (1992)	2,869	38.4	(36.5 - 40.3)	5.9	(5.0 - 6.9)	11.4	(10.2 - 12.6)	0.6	(0.4 - 1.0)	16.6	(15.2 - 18.1)	3.8	(3.1 - 4.6)

Morocco (2003/4)	6,144	41.8	(40.6 - 43.1)	3.9	(3.4 - 4.4)	8.1	(7.4 - 8.8)	1.6	(1.3 - 2.0)	25.0	(23.9 - 26.1)	3.3	(2.9 - 3.8)
Türkiye (1993)	758	56.3	(52.7 - 60.0)	6.5	(4.8 - 8.4)	22.7	(19.7 - 25.8)	4.4	(3.1 - 6.1)	11.6	(9.4 - 14.1)	11.2	(9.0 - 13.6)
Türkiye (1998)	671	58.0	(54.2 - 61.9)	7.0	(5.2 - 9.2)	22.5	(19.4 - 25.8)	3.1	(1.9 - 4.7)	10.5	(8.3 - 13.1)	14.9	(12.3 - 17.7)
Türkiye (2003/4)	888	56.4	(53.0 - 59.8)	5.7	(4.2 - 7.4)	27.5	(24.5 - 30.6)	5.6	(4.2 - 7.3)	10.0	(8.1 - 12.2)	7.6	(5.9 - 9.5)
Türkiye (2018/19)	331	41.6	(36.3 - 47.4)	4.8	(2.8 - 7.6)	23.3	(18.7 - 28.1)	3.5	(1.8 - 6.0)	8.0	(5.4 - 11.4)	2.0	(0.8 - 4.1)
Ukraine (2007)	348	25.6	(21.1 - 30.9)	2.5	(1.2 - 4.7)	14.1	(10.5 - 18.2)	2.8	(1.4 - 5.1)	3.8	(2.1 - 6.5)	2.4	(1.1 - 4.6)
Yemen (2013)	4,182	44.1	(42.5 - 45.7)	5.4	(4.7 - 6.2)	17.3	(16.1 - 18.4)	4.8	(4.1 - 5.5)	13.2	(12.2 - 14.3)	3.4	(2.9 - 4.0)
**Central, South & Southeast Asia**													
Bangladesh (1993/94)	3,406	46.3	(44.6 - 48.1)	1.7	(1.3 - 2.2)	26.0	(24.5 - 27.6)	2.6	(2.0 - 3.2)	10.6	(9.5 - 11.7)	5.5	(4.7 - 6.3)
Bangladesh (1996/97)	3,194	45.9	(44.1 - 47.7)	2.9	(2.3 - 3.5)	24.7	(23.1 - 26.2)	2.3	(1.8 - 2.9)	10.7	(9.7 - 11.9)	5.3	(4.6 - 6.2)
Bangladesh (1999/0)	4,108	47.7	(46.1 - 49.4)	2.9	(2.4 - 3.5)	22.6	(21.3 - 23.9)	4.5	(3.9 - 5.2)	11.7	(10.7 - 12.8)	5.9	(5.2 - 6.7)
Bangladesh (2004)	5,117	47.8	(46.4 - 49.3)	3.8	(3.3 - 4.4)	21.2	(20.1 - 22.4)	3.8	(3.2 - 4.3)	16.5	(15.4 - 17.5)	2.5	(2.1 - 3.0)
Bangladesh (2011)	6,755	40.4	(39.2 - 41.7)	4.3	(3.8 - 4.8)	12.3	(11.5 - 13.1)	3.0	(2.6 - 3.4)	18.8	(17.9 - 19.8)	2.1	(1.7 - 2.4)
Bangladesh (2014)	5,973	35.3	(34.0 - 36.6)	4.9	(4.3 - 5.5)	10.5	(9.7 - 11.3)	2.8	(2.3 - 3.2)	15.2	(14.3 - 16.2)	1.9	(1.6 - 2.3)
Bangladesh (2017/18)	7,576	43.4	(42.2 - 44.5)	4.6	(4.2 - 5.2)	11.4	(10.7 - 12.2)	3.6	(3.2 - 4.0)	23.0	(22.0 - 24.0)	0.7	(0.6 - 1.0)
Bangladesh (2022)	6,419	37.1	(35.9 - 38.4)	4.3	(3.8 - 4.9)	8.5	(7.8 - 9.2)	3.3	(2.8 - 3.8)	20.0	(19.0 - 21.1)	1.0	(0.8 - 1.3)
Cambodia (2010/11)	2,264	31.0	(29.0 - 33.1)	2.2	(1.6 - 2.9)	10.1	(8.9 - 11.5)	6.4	(5.4 - 7.5)	9.1	(7.8 - 10.4)	3.2	(2.5 - 4.1)
Cambodia (2014)	2,384	28.0	(26.1 - 30.0)	1.5	(1.1 - 2.1)	9.7	(8.5 - 11.0)	5.2	(4.3 - 6.2)	9.6	(8.4 - 10.9)	1.9	(1.4 - 2.6)
Cambodia (2021/22)	3,878	26.3	(24.8 - 27.9)	1.3	(1.0 - 1.8)	7.0	(6.1 - 7.9)	5.3	(4.6 - 6.1)	11.3	(10.2 - 12.4)	1.4	(1.1 - 1.9)
India (2005/6)	6,142	50.2	(48.8 - 51.5)	2.6	(2.2 - 3.1)	21.6	(20.5 - 22.7)	5.1	(4.5 - 5.7)	16.1	(15.1 - 17.1)	4.7	(4.2 - 5.3)
India (2015/16)	29,568	42.2	(41.6 - 42.8)	1.9	(1.7 - 2.0)	10.9	(10.5 - 11.3)	7.7	(7.4 - 8.1)	16.3	(15.8 - 16.7)	5.4	(5.1 - 5.6)
India (2019/21)	43,567	54.2	(53.7 - 54.8)	2.4	(2.3 - 2.6)	9.2	(8.9 - 9.5)	13.1	(12.8 - 13.5)	19.9	(19.5 - 20.4)	9.5	(9.2 - 9.8)
Indonesia (1991)	3,923	31.4	(30.0 - 32.9)	2.7	(2.2 - 3.2)	11.3	(10.3 - 12.3)	2.5	(2.0 - 3.0)	10.6	(9.7 - 11.6)	4.4	(3.8 - 5.0)
Indonesia (1994)	5,073	34.2	(32.9 - 35.6)	4.2	(3.6 - 4.8)	11.0	(10.1 - 11.9)	3.5	(3.0 - 4.0)	12.2	(11.3 - 13.1)	3.4	(2.9 - 3.9)
Indonesia (1997)	5,431	34.8	(33.4 - 36.2)	4.1	(3.5 - 4.7)	11.6	(10.8 - 12.6)	4.8	(4.2 - 5.4)	12.7	(11.8 - 13.7)	1.5	(1.2 - 1.9)
Indonesia (2002/3)	5,005	33.6	(32.2 - 35.1)	4.1	(3.5 - 4.7)	9.2	(8.3 - 10.0)	5.5	(4.9 - 6.2)	9.8	(8.9 - 10.7)	5.1	(4.5 - 5.8)
Indonesia (2007)	5,967	40.4	(39.1 - 41.8)	3.1	(2.7 - 3.6)	12.6	(11.7 - 13.6)	9.2	(8.4 - 10.0)	10.5	(9.7 - 11.4)	4.9	(4.4 - 5.5)
Indonesia (2012)	6,145	42.3	(41.0 - 43.7)	3.3	(2.9 - 3.9)	13.8	(12.9 - 14.7)	5.8	(5.2 - 6.5)	11.7	(10.9 - 12.5)	7.7	(7.0 - 8.4)
Indonesia (2017)	5,654	47.6	(46.3 - 49.0)	3.2	(2.7 - 3.7)	17.1	(16.1 - 18.1)	8.0	(7.3 - 8.8)	16.0	(15.1 - 17.0)	3.3	(2.9 - 3.8)
Kazakhstan (1999)	380	66.6	(61.6 - 71.7)	9.9	(7.0 - 13.4)	21.0	(16.9 - 25.5)	19.9	(15.9 - 24.2)	13.0	(9.7 - 16.9)	2.7	(1.4 - 4.9)
Kyrgyz Republic (2012)	191	44.1	(36.4 - 52.7)	9.4	(5.3 - 14.9)	9.1	(5.2 - 14.2)	11.9	(7.4 - 17.6)	13.1	(8.2 - 19.1)	0.7	(0.1 - 3.7)
Maldives (2009)	511	38.9	(34.3 - 43.9)	2.9	(1.6 - 4.9)	13.4	(10.4 - 16.9)	2.7	(1.4 - 4.6)	8.7	(6.2 - 11.7)	11.3	(8.4 - 14.6)
Myanmar (2015/16)	1,848	44.4	(42.1 - 46.8)	5.5	(4.5 - 6.7)	8.2	(7.0 - 9.5)	7.9	(6.7 - 9.2)	22.4	(20.5 - 24.4)	0.3	(0.1 - 0.7)
Nepal (2011)	1,165	72.2	(69.4 - 74.8)	3.1	(2.2 - 4.3)	15.6	(13.6 - 17.9)	3.1	(2.1 - 4.2)	49.2	(46.2 - 52.1)	1.2	(0.7 - 2.0)
Nepal (2016)	1,587	75.1	(72.7 - 77.4)	2.3	(1.6 - 3.2)	11.6	(10.0 - 13.4)	3.7	(2.8 - 4.8)	56.9	(54.2 - 59.4)	0.6	(0.3 - 1.2)
Nepal (2022)	1,403	69.1	(66.5 - 71.7)	3.4	(2.5 - 4.6)	12.6	(10.8 - 14.5)	4.5	(3.4 - 5.7)	47.5	(44.7 - 50.3)	1.1	(0.6 - 1.8)
Pakistan (2012/13)	627	57.4	(53.1 - 61.7)	5.7	(3.9 - 8.0)	31.4	(27.4 - 35.4)	3.9	(2.4 - 5.8)	13.5	(10.7 - 16.6)	2.9	(1.7 - 4.6)
Pakistan (2017/18)	438	48.6	(43.6 - 53.9)	7.0	(4.7 - 9.9)	17.0	(13.4 - 21.0)	9.9	(7.1 - 13.2)	13.6	(10.3 - 17.4)	1.1	(0.4 - 2.7)
Philippines (1993)	1,512	41.6	(39.0 - 44.3)	5.2	(4.1 - 6.5)	14.2	(12.4 - 16.1)	2.9	(2.1 - 3.9)	11.0	(9.4 - 12.7)	8.2	(6.9 - 9.8)
Philippines (1998)	1,588	44.8	(42.3 - 47.5)	5.3	(4.2 - 6.6)	18.4	(16.5 - 20.5)	2.9	(2.1 - 3.8)	11.6	(10.1 - 13.3)	6.6	(5.4 - 7.9)
Philippines (2003)	1,998	40.4	(38.1 - 42.8)	3.9	(3.0 - 4.8)	18.2	(16.5 - 20.1)	4.5	(3.6 - 5.6)	10.0	(8.6 - 11.4)	3.9	(3.0 - 4.8)

Philippines (2022)	3,711	36.6	(34.9 - 38.3)	3.5	(2.9 - 4.2)	13.6	(12.4 - 14.8)	4.9	(4.2 - 5.7)	12.4	(11.3 - 13.6)	2.1	(1.7 - 2.7)
Tajikistan (2012)	237	36.4	(30.2 - 43.4)	5.6	(3.0 - 9.3)	2.9	(1.2 - 6.0)	6.9	(4.0 - 10.9)	10.6	(6.9 - 15.3)	10.3	(6.7 - 14.9)
Tajikistan (2017)	236	52.0	(45.1 - 59.3)	1.9	(0.6 - 4.5)	13.1	(8.8 - 18.3)	5.5	(2.9 - 9.4)	30.6	(24.4 - 37.1)	0.8	(0.2 - 2.9)
Vietnam (1997)	310	35.2	(29.8 - 41.3)	8.9	(5.8 - 12.7)	10.9	(7.6 - 14.8)	5.6	(3.3 - 8.8)	4.0	(2.1 - 6.9)	5.9	(3.6 - 9.0)
Vietnam (2002)	502	36.8	(32.5 - 41.3)	5.9	(4.0 - 8.3)	13.9	(10.9 - 17.2)	5.1	(3.4 - 7.4)	10.0	(7.5 - 12.9)	1.9	(0.9 - 3.4)
**Latin America & Caribbean**													
Bolivia (1994)	544	61.1	(56.9 - 65.4)	5.0	(3.4 - 7.1)	37.6	(33.5 - 41.8)	6.6	(4.7 - 9.0)	5.8	(4.0 - 8.0)	6.1	(4.3 - 8.3)
Brazil (1996)	3,713	47.0	(45.4 - 48.7)	4.7	(4.0 - 5.4)	12.9	(11.8 - 14.0)	3.9	(3.3 - 4.6)	9.2	(8.3 - 10.2)	16.3	(15.1 - 17.5)
Colombia (1990)	1,761	47.0	(44.5 - 49.6)	5.9	(4.8 - 7.2)	18.1	(16.2 - 20.1)	3.3	(2.5 - 4.3)	13.0	(11.4 - 14.8)	6.6	(5.5 - 8.0)
Colombia (1995)	2,495	54.6	(52.5 - 56.6)	6.4	(5.4 - 7.5)	24.7	(23.0 - 26.5)	6.1	(5.1 - 7.1)	13.3	(12.0 - 14.7)	4.1	(3.4 - 5.0)
Colombia (2000)	2,370	51.3	(49.2 - 53.4)	6.2	(5.2 - 7.3)	17.5	(15.9 - 19.1)	9.0	(7.8 - 10.2)	14.9	(13.4 - 16.4)	3.8	(3.0 - 4.7)
Colombia (2005)	6,538	49.4	(48.1 - 50.7)	6.6	(6.0 - 7.2)	18.2	(17.3 - 19.2)	11.1	(10.3 - 11.9)	12.1	(11.3 - 12.9)	1.4	(1.1 - 1.7)
Colombia (2010)	6,600	49.7	(48.4 - 51.0)	5.1	(4.5 - 5.6)	18.9	(17.9 - 19.9)	11.0	(10.2 - 11.8)	12.0	(11.2 - 12.8)	2.8	(2.4 - 3.3)
Colombia (2015/16)	3,489	45.5	(43.8 - 47.2)	5.4	(4.7 - 6.2)	16.7	(15.5 - 18.0)	9.5	(8.6 - 10.5)	11.9	(10.8 - 13.0)	2.0	(1.5 - 2.5)
Dominican Republic (19	1,691	65.6	(63.2 - 67.9)	6.1	(5.0 - 7.3)	25.3	(23.2 - 27.5)	3.3	(2.5 - 4.3)	23.0	(20.9 - 25.1)	7.8	(6.6 - 9.2)
Dominican Republic (19	2,271	60.6	(58.5 - 62.8)	7.1	(6.0 - 8.2)	25.4	(23.6 - 27.3)	2.5	(1.9 - 3.2)	19.4	(17.8 - 21.2)	6.2	(5.2 - 7.3)
Dominican Republic (20	6,622	53.1	(51.9 - 54.4)	6.8	(6.2 - 7.4)	21.7	(20.7 - 22.8)	4.2	(3.8 - 4.8)	15.3	(14.4 - 16.2)	5.1	(4.6 - 5.7)
Guatemala (1995)	606	48.5	(44.8 - 52.2)	5.2	(3.8 - 7.1)	22.4	(19.4 - 25.5)	4.0	(2.7 - 5.6)	7.8	(6.0 - 9.9)	9.1	(7.1 - 11.3)
Guatemala (1998/99)	422	53.1	(48.6 - 57.7)	2.5	(1.4 - 4.2)	27.6	(23.7 - 31.7)	3.2	(1.9 - 5.1)	10.9	(8.3 - 14.0)	8.8	(6.5 - 11.5)
Guatemala (2014/15)	1,125	44.1	(41.0 - 47.4)	10.2	(8.3 - 12.2)	15.8	(13.6 - 18.1)	5.2	(3.9 - 6.7)	10.7	(8.9 - 12.8)	2.3	(1.5 - 3.4)
Honduras (2011/12)	4,590	62.9	(61.5 - 64.4)	2.8	(2.3 - 3.3)	21.5	(20.3 - 22.7)	13.5	(12.5 - 14.5)	20.5	(19.3 - 21.7)	4.7	(4.1 - 5.4)
Nicaragua (1998)	3,041	50.9	(49.0 - 52.8)	6.5	(5.6 - 7.4)	19.2	(17.7 - 20.7)	5.1	(4.3 - 5.9)	13.1	(11.9 - 14.4)	7.0	(6.1 - 8.0)
Paraguay (1990)	1,392	61.0	(58.4 - 63.6)	2.7	(1.9 - 3.7)	32.2	(29.7 - 34.7)	4.0	(3.0 - 5.1)	8.2	(6.8 - 9.8)	13.9	(12.1 - 15.8)
Peru (1991/92)	1,761	57.5	(55.0 - 60.0)	4.0	(3.1 - 5.0)	29.4	(27.1 - 31.7)	5.3	(4.3 - 6.5)	10.9	(9.4 - 12.5)	7.9	(6.6 - 9.3)
Peru (1996)	3,424	55.0	(53.2 - 56.8)	5.5	(4.7 - 6.3)	28.5	(26.9 - 30.2)	6.3	(5.5 - 7.3)	7.1	(6.2 - 8.0)	7.5	(6.6 - 8.5)
Peru (2000)	3,363	56.2	(54.3 - 58.0)	4.3	(3.6 - 5.1)	31.6	(29.9 - 33.3)	9.8	(8.8 - 11.0)	6.8	(5.9 - 7.7)	3.7	(3.1 - 4.5)
Peru (2004/6)	2,811	62.8	(60.7 - 64.8)	2.5	(1.9 - 3.2)	33.8	(31.8 - 35.8)	13.5	(12.1 - 15.0)	8.6	(7.5 - 9.9)	4.3	(3.5 - 5.3)
Peru (2007/8)	3,868	61.5	(59.8 - 63.2)	2.5	(2.0 - 3.1)	31.9	(30.3 - 33.5)	14.9	(13.7 - 16.1)	6.8	(5.9 - 7.6)	5.4	(4.7 - 6.2)
Peru (2009)	3,904	63.5	(61.9 - 65.1)	3.4	(2.8 - 4.0)	30.0	(28.5 - 31.5)	18.5	(17.2 - 19.8)	8.9	(8.0 - 9.9)	2.7	(2.2 - 3.3)
Peru (2010)	3,691	62.6	(60.9 - 64.2)	2.3	(1.8 - 2.8)	32.1	(30.6 - 33.7)	16.2	(15.0 - 17.4)	8.9	(7.9 - 9.9)	3.1	(2.6 - 3.7)
Peru (2011)	3,751	63.3	(61.6 - 65.0)	2.9	(2.4 - 3.5)	32.6	(31.0 - 34.2)	13.8	(12.7 - 15.0)	8.6	(7.7 - 9.6)	5.4	(4.7 - 6.2)
Peru (2012)	4,091	59.9	(58.3 - 61.5)	2.8	(2.3 - 3.4)	32.0	(30.5 - 33.5)	14.1	(13.0 - 15.2)	8.9	(8.0 - 9.9)	2.1	(1.6 - 2.6)

CI= Confidence Interval

S8.2 Table: 12-month overall estimated probabilities of discontinuation and reason-specific estimated cumulative incidence of discontinuation per 100 episodes of use

IUD

	No. of episodes	All reasons		Became pregnant while using		Side effect/health concerns		Other method-related		Wanted pregnancy/no need		Other/not stated	
		Rate	95%CI	Rate	95%CI	Rate	95%CI	Rate	95%CI	Rate	95%CI	Rate	95%CI
**Sub-Saharan Africa**													
Benin (2017/18)	231	9.9107	6.51 - 14.94	1.0	(0.2 - 3.5)	3.1	(1.4 - 6.1)	0.8	(0.1 - 2.9)	2.0	(0.7 - 4.9)	2.9	(1.2 - 6.0)
Burkina Faso (2021)	325	20.859	16.3 - 26.43	0.5	(0.1 - 2.0)	8.2	(5.2 - 12.1)	2.8	(1.3 - 5.3)	5.8	(3.4 - 9.1)	3.6	(1.8 - 6.3)
Burundi (2010/11)	139	16.824	10.5 - 26.28	3.6	(0.8 - 10.0)	5.0	(1.8 - 10.6)	2.7	(0.6 - 7.5)	4.6	(1.7 - 9.7)	1.0	(0.1 - 3.8)
Ethiopia (2016)	202	11.558	7.81 - 16.93	0.0	(0.0 - 0.0)	7.6	(4.4 - 12.0)	1.1	(0.2 - 3.2)	1.1	(0.3 - 3.3)	1.7	(0.5 - 4.3)
Guinea (2018)	251	84.5	(79.8 - 88.6)	0.0	(0.0 0.0)	66.2	(60.2 - 71.6)	0.7	(0.1 - 2.4)	16.7	(12.4 - 21.5)	0.9	(0.2 - 2.7)
Kenya (1998)	108	22.3	(15.5 - 31.5)	2.1	(0.5 - 6.4)	14.7	(8.7 - 22.2)	2.3	(0.6 - 6.3)	3.1	(0.9 - 7.9)	0.0	(0.0 - 0.0)
Kenya (2003)	113	13.1	(7.7 - 21.9)	0.6	(0.0 - 4.6)	8.2	(3.8 - 14.8)	0.0	(0.0 - 0.0)	4.3	(1.4 - 9.9)	0.0	(0.0 - 0.0)
Kenya (2014)	272	8.3	(5.5 - 12.4)	0.8	(0.2 - 2.7)	5.6	(3.2 - 8.9)	0.2	(0.0 - 1.6)	1.1	(0.3 - 3.2)	0.6	(0.1 - 2.3)
Kenya (2022)	365	11.6	(8.8 - 15.2)	0.0	(0.0 0.0)	7.6	(5.3 - 10.4)	0.5	(0.1 - 1.7)	3.4	(1.9 - 5.6)	0.1	(0.0 - 1.4)
Madagascar (2021)	118	22.3	(15.4 - 31.8)	4.6	(1.7 - 9.7)	8.2	(3.8 - 14.6)	4.6	(1.6 - 9.9)	2.8	(0.7 - 7.5)	2.2	(0.5 - 6.8)
Malawi (2015/16)	205	12.1	(8.0 - 18.1)	0.0	(0.0 - 0.0)	5.2	(2.5 - 9.3)	0.5	(0.0 - 2.8)	4.9	(2.3 - 8.8)	1.6	(0.4 - 4.6)
Mali (2018)	109	16.6	(10.7 - 25.2)	0.0	(0.0 0.0)	0.0	(0.0 - 0.0)	4.8	(1.8 - 10.2)	10.2	(5.3 - 16.8)	1.6	(0.3 - 5.4)
Mozambique (2022/2	131	36.7	(29.2 - 45.5)	0.0	(0.0 0.0)	8.0	(4.2 - 13.3)	5.3	(2.3 - 10.0)	17.3	(11.5 - 24.1)	6.2	(3.0 - 11.1)
Nigeria (2013)	276	9.3	(6.3 - 13.6)	1.0	(0.3 - 2.8)	2.4	(1.0 - 4.9)	0.0	(0.0 0.0)	5.2	(2.9 - 8.5)	0.7	(0.1 - 2.4)
Nigeria (2018)	230	15.5	(11.3 - 21.1)	2.4	(0.9 - 5.2)	4.1	(2.0 - 7.4)	1.4	(0.4 - 3.7)	6.2	(3.5 - 10.0)	1.4	(0.4 - 3.7)
Rwanda (2014/15)	113	17.0	(10.8 - 26.2)	3.3	(0.9 - 8.2)	7.0	(3.1 - 13.3)	0.0	(0.0 0.0)	2.7	(0.6 - 7.8)	4.0	(1.3 - 9.2)
Rwanda (2019/20)	199	15.3	(10.6 - 21.7)	1.0	(0.2 - 3.6)	5.5	(2.7 - 9.7)	1.7	(0.4 - 4.8)	4.9	(2.3 - 9.0)	2.0	(0.6 - 4.9)
Senegal (2018)	125	14.3	(9.5 - 21.2)	3.7	(1.4 - 7.8)	3.6	(1.4 - 7.6)	2.2	(0.7 - 5.4)	3.5	(1.3 - 7.4)	1.3	(0.2 - 4.7)
Senegal (2019)	120	14.0	(8.9 - 21.6)	0.0	(0.0 0.0)	5.2	(2.3 - 10.0)	3.1	(0.9 - 7.5)	1.9	(0.4 - 5.7)	3.8	(1.3 - 8.4)
Senegal (2023)	231	21.1	(16.5 - 26.8)	1.1	(0.2 - 3.2)	10.9	(7.4 - 15.1)	0.8	(0.2 - 2.7)	5.9	(3.4 - 9.3)	2.5	(1.0 - 5.2)
Tanzania (2015/16)	109	13.2	(7.9 - 21.7)	1.1	(0.1 - 5.2)	11.0	(5.8 - 18.1)	0.0	(0.0 - 0.0)	1.1	(0.1 - 5.3)	0.0	(0.0 0.0)
Tanzania (2022)	116	27.6	(20.2 - 37.1)	0.0	(0.0 0.0)	11.5	(6.3 - 18.5)	1.4	(0.2 - 5.1)	14.0	(8.4 - 21.0)	0.6	(0.0 - 3.8)
Zambia (2013/14)	136	12.0	(7.6 - 18.6)	3.5	(1.3 - 7.5)	6.6	(3.2 - 11.4)	1.2	(0.2 - 4.5)	0.0	(0.0 - 0.0)	0.8	(0.1 - 3.6)
**North Africa Western Asia and Europe**													
Armenia (2000)	279	7.5	(4.9 - 11.4)	1.9	(0.8 - 4.1)	4.4	(2.4 - 7.4)	0.0	(0.0 - 0.0)	1.1	(0.3 - 3.1)	0.0	(0.0 - 0.0)
Armenia (2005)	258	7.5	(4.8 - 11.6)	0.4	(0.0 - 1.9)	5.7	(3.3 - 9.2)	0.0	(0.0 0.0)	1.4	(0.4 - 3.6)	0.0	(0.0 - 0.0)
Armenia (2010)	207	4.5	(2.3 - 8.6)	0.0	(0.0 - 0.0)	3.2	(1.3 - 6.3)	0.0	(0.0 - 0.0)	0.3	(0.0 - 2.4)	1.0	(0.2 - 3.5)
Armenia (2015/16)	194	2.3	(0.9 - 5.9)	0.9	(0.2 - 3.5)	0.4	(0.0 - 2.6)	0.0	(0.0 0.0)	0.5	(0.0 - 2.6)	0.5	(0.0 - 2.8)
Azerbaijan (2006)	352	5.6	(3.6 - 8.8)	0.9	(0.3 - 2.5)	2.8	(1.3 - 5.0)	0.4	(0.1 - 1.7)	0.6	(0.1 - 2.1)	0.9	(0.3 - 2.4)
Egypt (1992/93)	2,796	13.0	(11.7 - 14.3)	1.6	(1.2 - 2.1)	8.1	(7.1 - 9.2)	0.4	(0.2 - 0.7)	2.8	(2.2 - 3.5)	0.1	(0.0 - 0.3)
Egypt (1995/96)	4,154	15.1	(14.1 - 16.3)	1.4	(1.1 - 1.8)	8.9	(8.1 - 9.8)	0.2	(0.1 - 0.3)	4.1	(3.5 - 4.7)	0.6	(0.4 - 0.8)

Egypt (2000)	5,280	14.7	(13.7 - 15.7)	1.0	(0.7 - 1.3)	8.9	(8.1 - 9.7)	0.2	(0.1 - 0.3)	4.1	(3.6 - 4.7)	0.6	(0.4 - 0.8)
Egypt (2003)	2,909	14.1	(12.9 - 15.5)	1.5	(1.1 - 2.0)	8.3	(7.3 - 9.3)	0.3	(0.2 - 0.6)	4.0	(3.3 - 4.8)	0.1	(0.0 - 0.3)
Egypt (2005)	6,575	16.0	(15.1 - 16.9)	1.3	(1.1 - 1.6)	9.1	(8.4 - 9.8)	0.7	(0.6 - 1.0)	4.4	(4.0 - 5.0)	0.3	(0.2 - 0.5)
Egypt (2008)	4,726	12.6	(11.6 - 13.6)	0.9	(0.7 - 1.3)	6.5	(5.8 - 7.2)	0.3	(0.2 - 0.5)	4.4	(3.9 - 5.1)	0.4	(0.2 - 0.6)
Egypt (2014)	5,364	15.1	(14.1 - 16.1)	1.2	(1.0 - 1.6)	7.9	(7.2 - 8.7)	0.2	(0.1 - 0.3)	5.6	(5.0 - 6.3)	0.1	(0.0 - 0.2)
Jordan (1990)	1,478	21.1	(19.0 - 23.4)	2.4	(1.7 - 3.3)	11.0	(9.4 - 12.7)	1.1	(0.7 - 1.8)	4.7	(3.6 - 5.9)	1.9	(1.3 - 2.7)
Jordan (1997)	1,701	18.5	(16.7 - 20.6)	2.3	(1.6 - 3.1)	9.8	(8.3 - 11.3)	2.2	(1.5 - 3.0)	3.9	(3.0 - 5.0)	0.5	(0.2 - 1.0)
Jordan (2002)	1,590	13.5	(11.8 - 15.3)	1.4	(0.9 - 2.1)	7.7	(6.4 - 9.1)	1.2	(0.8 - 1.9)	3.1	(2.3 - 4.1)	0.1	(0.0 - 0.4)
Jordan (2007)	2,519	12.7	(11.4 - 14.1)	0.8	(0.5 - 1.2)	6.6	(5.6 - 7.6)	1.1	(0.7 - 1.5)	4.0	(3.2 - 4.8)	0.3	(0.1 - 0.6)
Jordan (2009)	2,301	15.5	(14.0 - 17.0)	0.9	(0.6 - 1.3)	8.0	(7.0 - 9.2)	1.1	(0.7 - 1.6)	5.0	(4.2 - 6.0)	0.4	(0.2 - 0.7)
Jordan (2012)	2,438	13.6	(12.3 - 15.1)	1.6	(1.2 - 2.2)	5.7	(4.8 - 6.7)	1.6	(1.1 - 2.1)	4.6	(3.8 - 5.5)	0.1	(0.0 - 0.4)
Jordan (2017/18)	2,139	13.1	(11.7 - 14.7)	0.2	(0.1 - 0.6)	0.7	(0.4 - 1.2)	4.7	(3.8 - 5.6)	7.0	(6.0 - 8.2)	0.5	(0.2 - 0.8)
Jordan (2023)	1,737	15.1	(13.4 - 17.1)	1.1	(0.6 - 1.8)	4.0	(3.1 - 5.1)	1.8	(1.2 - 2.6)	8.0	(6.7 - 9.5)	0.2	(0.1 - 0.6)
Moldova (2005)	905	6.9	(5.4 - 8.9)	1.3	(0.7 - 2.3)	3.6	(2.5 - 5.0)	0.6	(0.3 - 1.4)	0.8	(0.3 - 1.6)	0.6	(0.2 - 1.3)
Morocco (1992)	252	19.7	(15.1 - 25.4)	2.2	(0.8 - 4.7)	10.6	(7.1 - 15.0)	2.6	(1.1 - 5.2)	2.2	(0.8 - 4.9)	2.1	(0.8 - 4.5)
Morocco (2003/4)	493	16.6	(13.6 - 20.3)	1.4	(0.6 - 2.8)	11.2	(8.5 - 14.2)	0.9	(0.3 - 2.1)	1.7	(0.8 - 3.3)	1.4	(0.6 - 2.8)
Türkiye (1993)	1,221	10.8	(9.1 - 12.8)	1.0	(0.5 - 1.7)	6.8	(5.4 - 8.4)	0.0	(0.0 - 0.0)	1.5	(0.9 - 2.3)	1.5	(0.9 - 2.4)
Türkiye (1998)	1,023	10.4	(8.6 - 12.5)	1.0	(0.5 - 1.8)	6.4	(4.9 - 8.0)	0.2	(0.0 - 0.7)	1.5	(0.9 - 2.5)	1.3	(0.7 - 2.1)
Türkiye (2003/4)	1,200	11.3	(9.6 - 13.2)	1.6	(1.0 - 2.5)	6.7	(5.3 - 8.2)	0.1	(0.0 - 0.5)	1.2	(0.7 - 2.0)	1.7	(1.1 - 2.6)
Türkiye (2018/19)	474	12.7	(9.8 - 16.2)	1.9	(0.9 - 3.5)	5.4	(3.5 - 7.8)	1.3	(0.5 - 2.7)	2.3	(1.2 - 4.1)	1.9	(0.9 - 3.6)
Ukraine (2007)	553	1.4	(0.7 - 2.9)	0.6	(0.2 - 1.6)	0.3	(0.1 - 1.2)	0.3	(0.0 - 1.2)	0.1	(0.0 - 0.9)	0.1	(0.0 - 1.0)
Yemen (2013)	1,051	18.9	(16.5 - 21.5)	1.4	(0.8 - 2.3)	11.1	(9.2 - 13.2)	1.7	(1.0 - 2.7)	3.6	(2.6 - 4.9)	1.0	(0.5 - 1.8)
**Central, South & Southeast Asia**													
Bangladesh (1993/94)	387	38.4	(33.4 - 43.8)	0.3	(0.0 - 1.6)	30.9	(26.0 - 35.9)	1.5	(0.6 - 3.2)	2.3	(1.1 - 4.4)	3.4	(1.8 - 5.7)
Bangladesh (1996/97)	265	41.4	(35.4 - 47.9)	0.0	(0.0 0.0)	35.4	(29.4 - 41.4)	0.4	(0.0 - 2.0)	2.6	(1.1 - 5.3)	3.0	(1.4 - 5.7)
Bangladesh (1999/0)	187	33.8	(27.0 - 41.8)	0.0	(0.0 0.0)	27.6	(20.9 - 34.7)	0.8	(0.1 - 3.3)	3.2	(1.1 - 6.9)	2.2	(0.6 - 5.5)
Bangladesh (2004)	110	33.6	(25.1 - 44.0)	1.0	(0.1 - 5.3)	22.8	(15.1 - 31.5)	4.2	(1.4 - 9.7)	5.1	(1.8 - 10.9)	0.6	(0.0 - 4.2)
Bangladesh (2011)	138	23.2	(16.6 - 31.9)	1.7	(0.3 - 5.4)	17.0	(10.9 - 24.4)	1.8	(0.4 - 5.3)	0.9	(0.1 - 4.5)	1.9	(0.4 - 5.7)
Cambodia (2010/11)	289	7.3	(4.6 - 11.4)	0.3	(0.0 - 2.1)	2.6	(1.2 - 5.0)	2.8	(1.2 - 5.6)	1.5	(0.5 - 3.9)	0.0	(0.0 - 0.0)
Cambodia (2014)	533	9.8	(7.5 - 12.8)	0.0	(0.0 - 0.0)	5.9	(4.0 - 8.2)	2.6	(1.4 - 4.3)	0.5	(0.1 - 1.5)	0.8	(0.3 - 1.9)
Cambodia (2021/22)	482	15.5	(12.6 - 18.9)	0.6	(0.2 - 1.6)	6.4	(4.5 - 8.8)	2.9	(1.7 - 4.7)	5.1	(3.4 - 7.2)	0.5	(0.1 - 1.4)
India (2005/6)	2,716	20.1	(18.4 - 21.9)	0.9	(0.5 - 1.4)	13.3	(11.8 - 14.8)	1.7	(1.2 - 2.3)	2.9	(2.2 - 3.8)	1.3	(0.9 - 1.9)
India (2015/16)	9,930	27.2	(26.2 - 28.2)	1.3	(1.0 - 1.5)	8.2	(7.6 - 8.8)	6.2	(5.7 - 6.8)	8.1	(7.5 - 8.7)	3.5	(3.1 - 3.9)
India (2019/21)	14,154	34.6	(33.7 - 35.5)	2.0	(1.8 - 2.3)	11.4	(10.8 - 12.0)	8.0	(7.5 - 8.5)	8.9	(8.4 - 9.4)	4.3	(4.0 - 4.7)
Indonesia (1991)	2,114	16.5	(14.9 - 18.2)	1.3	(0.9 - 1.9)	7.9	(6.8 - 9.2)	4.2	(3.4 - 5.2)	1.8	(1.3 - 2.5)	1.2	(0.8 - 1.8)
Indonesia (1994)	1,887	15.5	(13.9 - 17.3)	1.9	(1.3 - 2.6)	8.5	(7.3 - 9.9)	3.1	(2.3 - 4.0)	1.1	(0.7 - 1.7)	1.0	(0.6 - 1.6)
Indonesia (1997)	1,170	12.6	(10.8 - 14.8)	1.4	(0.8 - 2.3)	7.9	(6.3 - 9.6)	1.6	(0.9 - 2.4)	1.6	(0.9 - 2.4)	0.2	(0.1 - 0.7)
Indonesia (2002/3)	938	9.1	(7.3 - 11.4)	0.7	(0.3 - 1.5)	6.2	(4.7 - 8.1)	1.1	(0.5 - 2.0)	0.7	(0.3 - 1.5)	0.5	(0.2 - 1.2)

Indonesia (2007)	658	11.7	(9.4 - 14.6)	0.6	(0.2 - 1.5)	6.9	(5.1 - 9.0)	1.6	(0.8 - 2.8)	1.9	(1.0 - 3.3)	0.7	(0.3 - 1.7)
Indonesia (2012)	791	6.2	(4.7 - 8.3)	0.2	(0.0 - 0.8)	3.2	(2.1 - 4.7)	0.7	(0.3 - 1.6)	1.3	(0.6 - 2.4)	0.8	(0.3 - 1.7)
Indonesia (2017)	1,147	9.3	(7.8 - 11.0)	0.6	(0.3 - 1.2)	5.0	(3.9 - 6.4)	2.3	(1.6 - 3.3)	1.0	(0.6 - 1.7)	0.3	(0.1 - 0.7)
Kazakhstan (1999)	1,075	12.4	(10.6 - 14.6)	3.2	(2.3 - 4.4)	5.8	(4.5 - 7.3)	0.8	(0.4 - 1.5)	1.7	(1.0 - 2.6)	1.0	(0.5 - 1.7)
Kyrgyz Republic (201	1,144	9.8	(8.0 - 11.9)	0.4	(0.1 - 1.0)	5.0	(3.7 - 6.6)	1.2	(0.6 - 2.0)	3.1	(2.1 - 4.3)	0.2	(0.0 - 0.7)
Myanmar (2015/16)	187	6.9	(3.9 - 12.0)	1.1	(0.2 - 4.0)	3.6	(1.5 - 7.3)	1.2	(0.2 - 3.8)	0.1	(0.0 - 3.0)	0.9	(0.1 - 3.4)
Nepal (2011)	122	23.3	(15.7 - 33.9)	0.0	(0.0 - 0.0)	19.7	(12.0 - 28.9)	1.2	(0.2 - 5.1)	2.4	(0.5 - 7.0)	0.0	(0.0 0.0)
Nepal (2016)	163	31.3	(24.3 - 39.7)	0.0	(0.0 - 0.0)	18.6	(12.7 - 25.5)	2.9	(1.0 - 6.5)	7.9	(4.2 - 13.2)	1.8	(0.4 - 5.0)
Nepal (2022)	121	25.6	(18.6 - 34.4)	1.9	(0.4 - 5.5)	17.4	(11.1 - 24.8)	0.9	(0.1 - 4.1)	5.3	(2.3 - 10.3)	0.0	(0.0 0.0)
Pakistan (2012/13)	467	27.4	(23.6 - 31.6)	1.5	(0.7 - 2.9)	22.1	(18.5 - 25.9)	0.8	(0.3 - 2.0)	1.9	(1.0 - 3.5)	1.1	(0.4 - 2.3)
Pakistan (2017/18)	253	22.4	(18.2 - 27.4)	2.4	(1.1 - 4.4)	17.7	(13.7 - 22.1)	1.2	(0.4 - 2.9)	1.2	(0.4 - 3.0)	0.0	(0.0 0.0)
Philippines (1993)	312	22.4	(17.7 - 28.3)	2.7	(1.2 - 5.3)	8.4	(5.3 - 12.3)	3.8	(1.9 - 6.6)	2.6	(1.1 - 5.4)	5.0	(2.7 - 8.3)
Philippines (1998)	404	14.5	(10.9 - 19.0)	1.1	(0.3 - 2.9)	8.2	(5.4 - 11.7)	3.6	(1.9 - 6.2)	1.1	(0.3 - 2.8)	0.5	(0.1 - 2.0)
Philippines (2003)	315	13.8	(10.2 - 18.4)	0.6	(0.1 - 2.2)	7.3	(4.6 - 10.8)	1.3	(0.4 - 3.3)	0.8	(0.2 - 2.5)	3.8	(2.0 - 6.6)
Philippines (2022)	317	18.0	(14.1 - 23.0)	1.6	(0.6 - 3.6)	12.1	(8.7 - 16.2)	1.0	(0.3 - 2.8)	2.2	(0.9 - 4.4)	1.1	(0.3 - 2.9)
Tajikistan (2012)	1,037	10.1	(8.3 - 12.3)	0.4	(0.1 - 1.0)	3.0	(2.0 - 4.2)	2.3	(1.4 - 3.4)	3.3	(2.2 - 4.6)	1.2	(0.6 - 2.1)
Tajikistan (2017)	1,207	15.1	(13.0 - 17.5)	0.7	(0.3 - 1.3)	8.5	(6.8 - 10.3)	0.4	(0.2 - 1.1)	4.8	(3.6 - 6.3)	0.7	(0.3 - 1.4)
Vietnam (1997)	1,488	10.8	(9.3 - 12.5)	1.5	(0.9 - 2.2)	7.5	(6.2 - 9.0)	0.1	(0.0 - 0.5)	0.8	(0.4 - 1.4)	0.9	(0.5 - 1.4)
Vietnam (2002)	1,211	12.9	(11.1 - 15.0)	1.7	(1.0 - 2.6)	8.9	(7.3 - 10.6)	0.3	(0.1 - 0.8)	1.8	(1.1 - 2.7)	0.3	(0.1 - 0.8)
**Latin America & Caribbean**													
Bolivia (1994)	451	12.9	(10.0 - 16.5)	1.6	(0.7 - 3.2)	8.3	(5.9 - 11.1)	0.4	(0.1 - 1.4)	1.0	(0.3 - 2.4)	1.6	(0.7 - 3.2)
Brazil (1996)	118	13.7	(8.6 - 21.6)	1.1	(0.1 - 4.4)	9.8	(5.2 - 16.1)	0.8	(0.1 - 4.5)	1.2	(0.1 - 4.9)	0.8	(0.1 - 4.0)
Colombia (1990)	802	20.3	(17.6 - 23.4)	4.6	(3.2 - 6.2)	11.0	(8.9 - 13.4)	0.8	(0.4 - 1.7)	1.9	(1.1 - 3.1)	2.0	(1.2 - 3.2)
Colombia (1995)	912	19.7	(17.2 - 22.6)	3.2	(2.2 - 4.5)	12.1	(10.0 - 14.4)	1.4	(0.8 - 2.3)	1.9	(1.1 - 3.1)	1.1	(0.6 - 2.0)
Colombia (2000)	879	17.2	(14.8 - 20.0)	3.7	(2.6 - 5.2)	9.7	(7.8 - 11.9)	1.0	(0.5 - 1.9)	1.5	(0.8 - 2.5)	1.3	(0.7 - 2.3)
Colombia (2005)	2,572	17.2	(15.8 - 18.7)	2.4	(1.9 - 3.1)	11.5	(10.3 - 12.8)	1.5	(1.1 - 2.0)	1.0	(0.7 - 1.5)	0.7	(0.4 - 1.1)
Colombia (2010)	2,171	20.2	(18.5 - 22.0)	2.7	(2.0 - 3.4)	13.9	(12.4 - 15.4)	0.9	(0.5 - 1.3)	1.7	(1.2 - 2.4)	1.1	(0.7 - 1.6)
Colombia (2015/16)	763	18.8	(16.3 - 21.6)	2.8	(1.8 - 4.1)	10.7	(8.7 - 12.9)	1.0	(0.5 - 1.9)	1.9	(1.1 - 3.0)	2.4	(1.5 - 3.6)
Dominican Republic (	188	38.5	(32.1 - 45.7)	1.0	(0.2 - 3.2)	27.6	(21.6 - 33.9)	0.7	(0.1 - 2.8)	4.3	(2.1 - 7.8)	4.8	(2.4 - 8.5)
Dominican Republic (	218	31.5	(26.2 - 37.6)	2.1	(0.8 - 4.5)	22.1	(17.3 - 27.3)	0.2	(0.0 - 1.8)	4.3	(2.3 - 7.3)	2.8	(1.2 - 5.4)
Dominican Republic (	491	28.4	(25.1 - 32.2)	2.1	(1.2 - 3.5)	14.7	(12.1 - 17.6)	4.3	(2.9 - 6.1)	3.1	(1.9 - 4.7)	4.2	(2.9 - 6.0)
Guatemala (1995)	159	19.2	(14.5 - 25.2)	0.4	(0.0 - 2.7)	8.6	(5.3 - 12.8)	0.8	(0.2 - 2.8)	5.2	(2.8 - 8.7)	4.2	(2.0 - 7.5)
Guatemala (2014/15)	236	15.2	(11.1 - 20.6)	0.9	(0.2 - 2.9)	6.7	(3.9 - 10.3)	1.2	(0.3 - 3.4)	4.2	(2.0 - 7.6)	2.2	(0.8 - 4.7)
Honduras (2011/12)	1,048	26.9	(24.2 - 29.7)	2.7	(1.9 - 3.9)	18.3	(16.0 - 20.8)	1.8	(1.1 - 2.7)	1.8	(1.1 - 2.9)	2.2	(1.4 - 3.2)
Nicaragua (1998)	1,108	24.9	(22.5 - 27.5)	1.6	(1.0 - 2.4)	16.2	(14.2 - 18.4)	1.8	(1.2 - 2.7)	3.1	(2.2 - 4.2)	2.2	(1.5 - 3.2)
Paraguay (1990)	224	17.0	(12.7 - 22.5)	1.8	(0.6 - 4.3)	9.5	(6.2 - 13.7)	3.0	(1.4 - 5.8)	0.8	(0.1 - 2.8)	1.9	(0.7 - 4.3)
Peru (1991/92)	1,332	12.7	(11.0 - 14.6)	1.2	(0.7 - 1.8)	9.1	(7.6 - 10.7)	0.3	(0.1 - 0.7)	0.9	(0.5 - 1.6)	1.2	(0.7 - 1.9)
Peru (1996)	2,641	18.4	(17.0 - 20.0)	0.6	(0.3 - 0.9)	14.1	(12.8 - 15.5)	0.9	(0.6 - 1.4)	1.6	(1.2 - 2.1)	1.2	(0.9 - 1.7)

Peru (2000)	1,420	16.1	(14.4 - 18.1)	0.5	(0.3 - 1.0)	12.9	(11.3 - 14.7)	0.5	(0.3 - 1.0)	1.2	(0.8 - 1.9)	0.9	(0.5 - 1.4)
Peru (2004/6)	482	22.1	(18.7 - 26.0)	0.5	(0.1 - 1.4)	17.9	(14.7 - 21.3)	0.2	(0.0 - 1.1)	2.8	(1.6 - 4.6)	0.7	(0.2 - 1.8)
Peru (2007/8)	413	14.7	(11.8 - 18.1)	0.6	(0.1 - 1.7)	13.2	(10.4 - 16.3)	0.0	(0.0 - 1.6)	0.1	(0.0 - 1.0)	0.8	(0.3 - 1.9)
Peru (2009)	387	20.9	(17.6 - 24.8)	1.7	(0.8 - 3.1)	14.8	(11.9 - 18.1)	0.5	(0.1 - 1.5)	1.5	(0.7 - 2.9)	2.4	(1.4 - 4.0)
Peru (2010)	307	22.2	(18.5 - 26.4)	2.0	(1.0 - 3.7)	15.6	(12.4 - 19.3)	0.4	(0.1 - 1.4)	2.0	(1.0 - 3.8)	2.1	(1.1 - 3.8)
Peru (2011)	278	23.1	(19.0 - 28.0)	0.8	(0.2 - 2.3)	18.2	(14.3 - 22.5)	0.9	(0.2 - 2.3)	1.7	(0.7 - 3.5)	1.6	(0.6 - 3.3)
Peru (2012)	287	16.5	(13.1 - 20.7)	0.2	(0.0 - 1.3)	14.2	(10.9 - 18.0)	0.3	(0.0 - 1.4)	0.7	(0.1 - 2.1)	1.1	(0.4 - 2.6)

CI= Confidence Interval

S8.3 Table: 12-month overall estimated probabilities of discontinuation and reason-specific estimated cumulative incidence of discontinuation per 100 episodes of use

Injectables

	No. of episodes	All reasons		Became pregnant while using		Side effect/health concerns		Other method-related		Wanted pregnancy/no need		Other/not stated	
		Rate	95%CI	Rate	95%CI	Rate	95%CI	Rate	95%CI	Rate	95%CI	Rate	95%CI
**Sub-Saharan Africa**													
Angola (2015/16)	401	42.3	(38.0 - 47.0)	0.0	(0.0 0.0)	19.5	(16.1 - 23.2)	4.7	(3.1 - 6.7)	9.4	(6.9 - 12.2)	8.8	(6.5 - 11.5)
Benin (2017/18)	631	57.5	(53.3 - 61.7)	0.1	(0.0 - 0.8)	15.8	(12.9 - 18.9)	8.3	(6.3 - 10.8)	20.6	(17.3 - 24.1)	12.6	(10.0 - 15.5)
Burkina Faso (2010)	1,325	29.7	(27.1 - 32.6)	0.3	(0.1 - 0.8)	9.7	(8.0 - 11.5)	4.2	(3.2 - 5.5)	12.8	(10.9 - 14.9)	2.8	(1.9 - 3.9)
Burkina Faso (2021)	2,115	45.4	(43.1 - 47.8)	0.9	(0.5 - 1.4)	9.0	(7.8 - 10.4)	10.6	(9.2 - 12.0)	17.7	(15.9 - 19.5)	7.2	(6.1 - 8.5)
Burundi (2010/11)	1,112	41.5	(38.3 - 44.9)	1.8	(1.0 - 2.8)	16.0	(13.7 - 18.5)	6.2	(4.8 - 7.9)	11.7	(9.7 - 14.0)	5.8	(4.4 - 7.4)
Comoros (2012)	249	27.4	(22.2 - 33.6)	1.4	(0.4 - 3.6)	13.1	(9.3 - 17.6)	2.6	(1.1 - 5.2)	7.7	(4.7 - 11.6)	2.6	(1.1 - 5.3)
Côte d'Ivoire (2021)	887	43.4	(39.7 - 47.1)	0.1	(0.0 - 0.9)	10.8	(8.7 - 13.1)	13.6	(11.2 - 16.3)	14.4	(11.9 - 17.1)	4.4	(3.1 - 6.1)
Ethiopia (2005)	1,486	35.5	(32.9 - 38.4)	0.3	(0.1 - 0.8)	12.0	(10.2 - 13.9)	5.9	(4.7 - 7.3)	11.8	(10.1 - 13.8)	5.5	(4.3 - 6.9)
Ethiopia (2016)	3,216	36.2	(34.6 - 37.8)	0.4	(0.3 - 0.7)	8.1	(7.2 - 9.0)	8.5	(7.7 - 9.5)	16.6	(15.4 - 17.8)	2.5	(2.0 - 3.0)
Gabon (2019/21)	135	49.4	(39.9 - 59.9)	0.0	(0.0 0.0)	28.0	(19.7 - 36.9)	10.0	(4.9 - 17.2)	1.2	(0.2 - 4.9)	10.2	(5.3 - 17.1)
Gambia (2013)	402	27.6	(23.0 - 33.0)	1.6	(0.6 - 3.5)	10.7	(7.7 - 14.3)	2.9	(1.4 - 5.1)	8.0	(5.3 - 11.4)	4.5	(2.6 - 7.2)
Gambia (2019/20)	1,359	52.5	(49.7 - 55.4)	0.9	(0.5 - 1.6)	18.0	(15.8 - 20.2)	5.3	(4.1 - 6.7)	18.2	(16.0 - 20.5)	10.1	(8.5 - 11.9)
Ghana (2014)	953	31.4	(28.1 - 35.0)	1.5	(0.8 - 2.6)	14.3	(11.8 - 17.0)	3.2	(2.1 - 4.7)	9.0	(7.1 - 11.3)	3.4	(2.2 - 4.9)
Ghana (2022/23)	2,060	59.0	(56.6 - 61.5)	0.7	(0.4 - 1.1)	32.4	(30.1 - 34.7)	6.4	(5.3 - 7.6)	15.2	(13.5 - 17.0)	4.4	(3.5 - 5.5)
Guinea (2018)	510	67.5	(63.3 - 71.6)	0.0	(0.0 0.0)	43.6	(39.3 - 47.9)	2.9	(1.7 - 4.7)	18.2	(15.0 - 21.7)	2.7	(1.5 - 4.4)
Kenya (1998)	843	23.7	(20.8 - 27.0)	0.9	(0.4 - 1.8)	12.3	(10.0 - 14.7)	1.4	(0.7 - 2.5)	4.4	(3.1 - 6.1)	4.7	(3.4 - 6.4)
Kenya (2003)	1,193	33.9	(31.2 - 36.9)	1.0	(0.6 - 1.8)	20.2	(17.9 - 22.7)	3.0	(2.1 - 4.2)	6.2	(4.9 - 7.8)	3.4	(2.4 - 4.6)
Kenya (2014)	3,660	32.6	(31.1 - 34.2)	1.8	(1.4 - 2.2)	15.4	(14.2 - 16.6)	3.5	(3.0 - 4.2)	8.3	(7.4 - 9.2)	3.7	(3.1 - 4.3)
Kenya (2022)	3,359	43.0	(41.3 - 44.8)	1.4	(1.1 - 1.9)	20.1	(18.7 - 21.5)	7.5	(6.6 - 8.5)	12.4	(11.2 - 13.6)	1.6	(1.2 - 2.1)
Lesotho (2014)	1,467	24.0	(21.8 - 26.5)	0.7	(0.3 - 1.3)	12.3	(10.6 - 14.2)	4.0	(3.0 - 5.2)	3.7	(2.7 - 4.8)	3.4	(2.5 - 4.5)
Liberia (2013)	1,303	28.2	(25.6 - 31.0)	1.0	(0.5 - 1.7)	18.9	(16.6 - 21.2)	3.5	(2.5 - 4.7)	3.2	(2.3 - 4.4)	1.6	(1.0 - 2.5)
Liberia (2019/20)	2,164	44.8	(42.4 - 47.2)	0.9	(0.5 - 1.5)	25.3	(23.3 - 27.4)	5.6	(4.6 - 6.8)	8.1	(6.9 - 9.5)	4.8	(3.9 - 5.9)
Madagascar (2021)	5,739	35.0	(33.7 - 36.4)	0.6	(0.4 - 0.8)	9.9	(9.1 - 10.7)	5.9	(5.3 - 6.6)	12.4	(11.5 - 13.3)	6.2	(5.6 - 6.9)
Malawi (2004/5)	2,985	33.7	(31.9 - 35.6)	1.4	(1.0 - 1.9)	14.5	(13.1 - 15.8)	2.1	(1.6 - 2.7)	10.5	(9.4 - 11.8)	5.2	(4.4 - 6.1)
Malawi (2015/16)	9,231	40.3	(39.2 - 41.4)	0.7	(0.5 - 0.8)	13.9	(13.1 - 14.6)	8.1	(7.5 - 8.7)	13.2	(12.5 - 14.0)	4.4	(3.9 - 4.8)
Mali (2012/13)	607	49.9	(45.4 - 54.5)	6.2	(4.3 - 8.6)	14.6	(11.6 - 17.9)	4.2	(2.7 - 6.2)	15.9	(12.8 - 19.4)	8.9	(6.6 - 11.6)
Mali (2018)	922	52.7	(49.5 - 56.0)	0.8	(0.4 - 1.6)	17.4	(15.1 - 19.9)	4.6	(3.4 - 6.1)	20.6	(18.0 - 23.3)	9.2	(7.5 - 11.2)
Mozambique (2011)	959	50.0	(46.4 - 53.7)	1.4	(0.8 - 2.4)	14.3	(11.9 - 16.8)	6.8	(5.1 - 8.7)	15.9	(13.3 - 18.7)	11.6	(9.5 - 14.0)
Mozambique (2022/2	2,260	43.3	(40.9 - 45.7)	0.2	(0.1 - 0.6)	6.9	(5.8 - 8.2)	8.2	(6.9 - 9.6)	16.8	(15.0 - 18.7)	11.2	(9.8 - 12.7)
Namibia (2013)	2,526	20.8	(19.1 - 22.7)	0.3	(0.1 - 0.6)	10.7	(9.4 - 12.1)	2.9	(2.2 - 3.7)	3.1	(2.4 - 4.0)	3.8	(3.1 - 4.7)
Niger (2012)	531	58.5	(53.9 - 63.2)	0.2	(0.0 - 1.1)	9.5	(7.0 - 12.5)	9.5	(7.1 - 12.5)	26.6	(22.6 - 30.8)	12.7	(9.8 - 16.0)
Nigeria (2013)	1,281	24.3	(21.8 - 27.1)	1.7	(1.1 - 2.7)	10.1	(8.4 - 12.0)	2.2	(1.4 - 3.2)	7.0	(5.5 - 8.7)	3.3	(2.3 - 4.5)
Nigeria (2018)	1,830	55.0	(52.5 - 57.6)	2.3	(1.7 - 3.2)	22.9	(20.8 - 25.0)	9.4	(8.0 - 10.9)	16.0	(14.2 - 17.9)	4.4	(3.4 - 5.5)

Rwanda (2010/11)	3,068	24.8	(23.2 - 26.6)	1.3	(0.9 - 1.8)	13.5	(12.2 - 14.8)	2.8	(2.2 - 3.5)	4.5	(3.8 - 5.4)	2.7	(2.2 - 3.4)
Rwanda (2014/15)	3,216	28.7	(27.1 - 30.4)	1.7	(1.3 - 2.3)	14.6	(13.3 - 15.9)	3.9	(3.2 - 4.6)	6.8	(5.9 - 7.8)	1.7	(1.3 - 2.2)
Rwanda (2019/20)	2,382	37.9	(35.9 - 40.0)	1.5	(1.0 - 2.1)	17.7	(16.1 - 19.4)	6.7	(5.7 - 7.8)	9.5	(8.4 - 10.8)	2.5	(1.9 - 3.2)
Senegal (2010/11)	982	54.0	(50.6 - 57.4)	0.9	(0.4 - 1.7)	24.4	(21.5 - 27.3)	6.3	(4.8 - 8.0)	9.2	(7.4 - 11.2)	13.3	(11.1 - 15.6)
Senegal (2015)	917	43.4	(40.0 - 46.8)	1.1	(0.6 - 2.0)	13.3	(11.1 - 15.8)	4.1	(2.9 - 5.6)	17.5	(15.0 - 20.1)	7.3	(5.7 - 9.2)
Senegal (2016)	912	43.0	(39.7 - 46.4)	1.2	(0.6 - 2.1)	18.6	(16.1 - 21.3)	4.7	(3.5 - 6.3)	12.5	(10.4 - 14.8)	5.9	(4.5 - 7.6)
Senegal (2018)	1,087	41.9	(39.0 - 44.9)	1.6	(1.0 - 2.5)	18.4	(16.2 - 20.8)	2.2	(1.4 - 3.2)	14.9	(12.9 - 17.1)	4.8	(3.6 - 6.2)
Senegal (2019)	1,004	40.4	(37.3 - 43.7)	0.4	(0.1 - 0.9)	17.4	(15.0 - 19.9)	4.8	(3.6 - 6.3)	13.3	(11.2 - 15.5)	4.6	(3.4 - 6.1)
Senegal (2023)	1,481	50.9	(48.2 - 53.5)	2.6	(1.8 - 3.5)	20.1	(18.0 - 22.2)	2.7	(2.0 - 3.7)	15.8	(14.0 - 17.8)	9.6	(8.2 - 11.2)
Sierra Leone (2013)	2,000	26.4	(24.4 - 28.6)	1.1	(0.7 - 1.7)	15.1	(13.4 - 16.8)	2.9	(2.2 - 3.8)	5.1	(4.1 - 6.2)	2.2	(1.6 - 3.0)
South Africa (2016)	2,026	25.3	(23.3 - 27.6)	1.3	(0.8 - 1.9)	11.4	(9.9 - 13.0)	4.2	(3.3 - 5.2)	5.4	(4.4 - 6.6)	3.1	(2.3 - 4.1)
Tanzania (2004/5)	1,362	37.9	(35.2 - 40.7)	1.0	(0.5 - 1.7)	21.7	(19.4 - 24.0)	4.9	(3.8 - 6.2)	7.6	(6.2 - 9.2)	2.7	(1.9 - 3.8)
Tanzania (2015/16)	2,333	33.2	(31.2 - 35.3)	0.7	(0.4 - 1.1)	16.9	(15.3 - 18.5)	4.4	(3.6 - 5.3)	8.8	(7.6 - 10.1)	2.5	(1.9 - 3.2)
Tanzania (2022)	2,048	49.9	(47.6 - 52.2)	1.1	(0.7 - 1.6)	22.1	(20.3 - 24.0)	7.6	(6.5 - 8.9)	15.0	(13.4 - 16.6)	4.1	(3.3 - 5.1)
Uganda (2011)	1,747	48.2	(45.8 - 50.7)	3.6	(2.8 - 4.6)	24.3	(22.2 - 26.4)	3.3	(2.5 - 4.2)	11.9	(10.4 - 13.5)	5.1	(4.1 - 6.3)
Zambia (2013/14)	3,760	28.4	(26.8 - 30.1)	1.8	(1.4 - 2.4)	11.7	(10.6 - 12.9)	3.1	(2.5 - 3.7)	7.5	(6.6 - 8.5)	4.3	(3.6 - 5.0)
Zambia (2018/19)	4,105	34.6	(33.0 - 36.2)	1.4	(1.1 - 1.9)	15.5	(14.4 - 16.8)	3.8	(3.2 - 4.5)	10.2	(9.2 - 11.2)	3.6	(3.1 - 4.3)
Zimbabwe (1994)	136	17.2	(11.0 - 26.4)	1.3	(0.2 - 4.9)	6.2	(2.7 - 11.8)	2.0	(0.4 - 6.4)	4.8	(1.5 - 11.2)	2.9	(0.8 - 7.4)
Zimbabwe (1999)	659	29.0	(25.4 - 32.9)	1.1	(0.5 - 2.3)	13.8	(11.1 - 16.9)	5.2	(3.5 - 7.2)	5.6	(3.9 - 7.7)	3.2	(2.0 - 5.0)
Zimbabwe (2005/6)	940	26.9	(23.9 - 30.2)	1.6	(0.9 - 2.7)	11.4	(9.3 - 13.7)	5.7	(4.3 - 7.5)	6.0	(4.5 - 7.9)	2.2	(1.4 - 3.5)
Zimbabwe (2010/11)	852	34.5	(31.0 - 38.2)	1.1	(0.5 - 2.1)	15.9	(13.3 - 18.8)	6.3	(4.7 - 8.3)	9.1	(7.1 - 11.4)	2.1	(1.2 - 3.4)
Zimbabwe (2015)	1,114	30.3	(27.4 - 33.3)	1.3	(0.7 - 2.2)	15.8	(13.6 - 18.2)	5.4	(4.1 - 6.9)	5.9	(4.5 - 7.5)	1.9	(1.1 - 2.9)
**North Africa Western Asia and Europe**													
Egypt (1992/93)	121	56.8	(47.6 - 66.4)	4.2	(1.5 - 9.2)	30.0	(21.7 - 38.8)	10.3	(5.5 - 17.0)	11.5	(6.4 - 18.2)	0.7	(0.0 - 4.3)
Egypt (1995/96)	504	56.1	(51.1 - 61.1)	0.6	(0.2 - 1.8)	31.3	(26.7 - 35.9)	8.6	(6.1 - 11.5)	8.9	(6.4 - 12.0)	6.7	(4.5 - 9.4)
Egypt (2000)	1,472	50.1	(47.3 - 53.0)	0.9	(0.5 - 1.5)	34.9	(32.3 - 37.6)	2.8	(2.0 - 3.8)	8.1	(6.6 - 9.6)	3.5	(2.5 - 4.6)
Egypt (2003)	1,279	43.3	(40.3 - 46.5)	0.3	(0.1 - 0.9)	29.0	(26.2 - 31.9)	3.2	(2.3 - 4.5)	10.0	(8.3 - 12.0)	0.7	(0.3 - 1.4)
Egypt (2005)	2,950	51.1	(49.1 - 53.1)	1.1	(0.7 - 1.6)	28.1	(26.3 - 29.9)	1.6	(1.2 - 2.2)	10.9	(9.7 - 12.2)	9.4	(8.3 - 10.6)
Egypt (2008)	1,753	38.8	(36.4 - 41.4)	0.9	(0.5 - 1.5)	22.2	(20.2 - 24.4)	1.5	(1.0 - 2.2)	11.4	(9.9 - 13.1)	2.7	(2.0 - 3.6)
Egypt (2014)	2,006	37.2	(35.0 - 39.5)	1.1	(0.7 - 1.6)	21.3	(19.4 - 23.2)	0.7	(0.4 - 1.2)	13.7	(12.1 - 15.3)	0.5	(0.3 - 0.9)
Jordan (2002)	154	58.5	(49.0 - 68.3)	0.7	(0.0 - 4.2)	43.3	(33.5 - 52.7)	3.3	(1.0 - 8.0)	8.7	(4.2 - 15.3)	2.5	(0.6 - 6.9)
Jordan (2007)	255	44.1	(36.9 - 52.0)	1.4	(0.3 - 4.2)	29.4	(22.6 - 36.4)	3.0	(1.2 - 6.4)	10.1	(6.1 - 15.2)	0.3	(0.0 - 2.7)
Jordan (2009)	315	65.6	(59.4 - 71.8)	1.8	(0.6 - 4.3)	49.1	(42.5 - 55.4)	5.5	(3.0 - 9.0)	8.1	(5.0 - 12.1)	1.1	(0.3 - 3.1)
Jordan (2012)	328	52.5	(46.1 - 59.3)	2.0	(0.7 - 4.5)	31.0	(25.0 - 37.2)	6.0	(3.4 - 9.6)	12.9	(8.9 - 17.8)	0.6	(0.1 - 2.4)
Jordan (2017/18)	188	58.6	(50.5 - 67.0)	0.4	(0.0 - 3.2)	8.9	(4.9 - 14.4)	29.7	(22.3 - 37.5)	19.2	(13.2 - 26.1)	0.4	(0.0 - 3.3)
Jordan (2023)	252	49.9	(42.8 - 57.4)	0.8	(0.1 - 3.2)	24.0	(18.0 - 30.4)	8.6	(5.1 - 13.2)	15.0	(10.3 - 20.5)	1.5	(0.4 - 4.0)
Morocco (2003/4)	428	52.7	(47.5 - 58.1)	1.2	(0.4 - 2.8)	40.0	(34.8 - 45.0)	2.3	(1.1 - 4.2)	4.7	(2.8 - 7.3)	4.6	(2.7 - 7.1)
Türkiye (2003/4)	173	81.1	(74.4 - 86.9)	3.3	(1.3 - 7.1)	56.4	(48.2 - 63.8)	9.9	(5.8 - 15.2)	6.6	(3.3 - 11.3)	4.9	(2.2 - 9.1)
Türkiye (2018/19)	106	54.9	(44.6 - 65.8)	2.4	(0.5 - 7.1)	36.2	(26.1 - 46.3)	7.6	(3.3 - 14.3)	6.6	(2.6 - 13.2)	2.2	(0.4 - 6.9)
Yemen (2013)	1,369	45.1	(42.2 - 48.0)	1.9	(1.3 - 2.8)	23.8	(21.4 - 26.3)	5.5	(4.3 - 7.0)	8.3	(6.8 - 10.0)	5.5	(4.3 - 7.0)
**Central, South & Southeast Asia**													

Bangladesh (1993/94)	811	59.8	(56.0 - 63.6)	1.1	(0.5 - 2.1)	41.5	(37.7 - 45.2)	5.9	(4.3 - 7.8)	6.3	(4.6 - 8.3)	5.0	(3.6 - 6.8)
Bangladesh (1996/97)	1,023	52.7	(49.4 - 56.1)	1.2	(0.6 - 2.1)	37.0	(33.8 - 40.3)	2.7	(1.8 - 4.0)	6.3	(4.8 - 8.1)	5.4	(4.0 - 7.0)
Bangladesh (1999/0)	1,272	51.8	(48.9 - 54.8)	1.3	(0.8 - 2.1)	37.2	(34.4 - 40.0)	4.2	(3.1 - 5.5)	4.9	(3.8 - 6.3)	4.1	(3.1 - 5.4)
Bangladesh (2004)	1,757	50.0	(47.5 - 52.5)	0.4	(0.2 - 0.8)	34.8	(32.4 - 37.2)	3.9	(3.0 - 4.9)	8.3	(7.0 - 9.8)	2.6	(1.9 - 3.4)
Bangladesh (2011)	2,588	37.8	(35.8 - 39.9)	1.2	(0.9 - 1.8)	23.9	(22.1 - 25.7)	3.5	(2.8 - 4.3)	7.4	(6.4 - 8.5)	1.8	(1.3 - 2.4)
Bangladesh (2014)	2,327	26.7	(24.8 - 28.8)	1.0	(0.6 - 1.5)	14.5	(13.0 - 16.1)	2.7	(2.1 - 3.5)	7.1	(6.0 - 8.3)	1.4	(1.0 - 2.0)
Bangladesh (2017/18)	2,714	36.3	(34.5 - 38.3)	1.1	(0.7 - 1.6)	21.7	(20.1 - 23.3)	3.0	(2.4 - 3.7)	9.8	(8.7 - 11.0)	0.8	(0.5 - 1.2)
Bangladesh (2022)	2,271	29.8	(27.8 - 31.9)	0.6	(0.4 - 1.1)	17.1	(15.5 - 18.8)	1.9	(1.4 - 2.6)	9.4	(8.2 - 10.8)	0.7	(0.4 - 1.1)
Cambodia (2010/11)	1,440	32.2	(29.6 - 34.9)	1.5	(0.9 - 2.4)	12.9	(11.1 - 14.8)	6.0	(4.8 - 7.4)	8.5	(7.0 - 10.2)	3.3	(2.4 - 4.5)
Cambodia (2014)	1,434	34.0	(31.4 - 36.6)	1.3	(0.8 - 2.1)	15.1	(13.2 - 17.1)	5.5	(4.4 - 6.9)	9.5	(7.9 - 11.1)	2.6	(1.8 - 3.5)
Cambodia (2021/22)	1,351	43.9	(40.8 - 47.2)	0.5	(0.2 - 1.1)	14.4	(12.3 - 16.7)	15.4	(13.1 - 17.7)	12.0	(10.0 - 14.2)	1.7	(1.0 - 2.7)
India (2005/6)	227	55.4	(47.6 - 63.5)	5.2	(2.5 - 9.5)	21.7	(15.6 - 28.4)	7.8	(4.2 - 12.8)	12.5	(7.8 - 18.3)	8.2	(4.5 - 13.4)
India (2015/16)	1,602	51.1	(48.3 - 54.1)	2.2	(1.4 - 3.1)	14.7	(12.7 - 16.7)	16.2	(14.2 - 18.4)	11.2	(9.5 - 13.1)	6.9	(5.5 - 8.4)
India (2019/21)	5,775	65.7	(64.3 - 67.1)	2.8	(2.3 - 3.3)	10.7	(9.8 - 11.6)	17.4	(16.3 - 18.5)	23.8	(22.6 - 25.0)	11.1	(10.2 - 12.0)
Indonesia (1991)	3,651	33.7	(32.2 - 35.3)	1.6	(1.2 - 2.0)	17.2	(16.0 - 18.5)	4.4	(3.8 - 5.1)	5.9	(5.1 - 6.7)	4.6	(4.0 - 5.3)
Indonesia (1994)	5,413	30.1	(28.8 - 31.4)	1.6	(1.3 - 2.0)	15.5	(14.5 - 16.5)	4.0	(3.4 - 4.5)	6.0	(5.3 - 6.6)	3.1	(2.6 - 3.6)
Indonesia (1997)	6,847	24.2	(23.2 - 25.3)	1.5	(1.2 - 1.8)	12.6	(11.8 - 13.4)	3.6	(3.1 - 4.1)	5.5	(4.9 - 6.1)	1.0	(0.8 - 1.3)
Indonesia (2002/3)	8,462	19.1	(18.3 - 20.1)	1.1	(0.9 - 1.4)	8.6	(8.0 - 9.2)	2.6	(2.3 - 3.0)	4.7	(4.3 - 5.2)	2.1	(1.8 - 2.5)
Indonesia (2007)	11,268	24.0	(23.1 - 24.8)	0.7	(0.6 - 0.9)	10.8	(10.2 - 11.4)	2.9	(2.6 - 3.3)	6.6	(6.1 - 7.1)	2.9	(2.6 - 3.3)
Indonesia (2012)	11,975	26.1	(25.3 - 26.9)	0.4	(0.3 - 0.6)	10.4	(9.9 - 11.0)	2.5	(2.2 - 2.8)	8.2	(7.7 - 8.7)	4.6	(4.2 - 5.0)
Indonesia (2017)	12,076	29.1	(28.3 - 29.9)	0.5	(0.4 - 0.6)	14.5	(13.9 - 15.2)	2.9	(2.6 - 3.2)	9.6	(9.1 - 10.1)	1.6	(1.4 - 1.8)
Maldives (2009)	157	45.0	(36.8 - 54.2)	0.3	(0.0 - 3.2)	30.0	(22.4 - 38.1)	2.6	(0.8 - 6.3)	4.3	(1.6 - 9.0)	7.8	(4.0 - 13.4)
Myanmar (2015/16)	3,357	42.9	(41.2 - 44.7)	0.9	(0.6 - 1.3)	16.9	(15.6 - 18.3)	8.0	(7.1 - 9.0)	13.9	(12.7 - 15.1)	3.2	(2.6 - 3.8)
Nepal (2011)	1,800	57.0	(54.5 - 59.5)	0.6	(0.3 - 1.1)	27.0	(24.7 - 29.2)	2.4	(1.7 - 3.2)	25.6	(23.5 - 27.8)	1.4	(0.9 - 2.1)
Nepal (2016)	1,963	59.6	(57.2 - 62.0)	0.3	(0.1 - 0.7)	20.3	(18.4 - 22.3)	2.8	(2.1 - 3.7)	35.0	(32.7 - 37.3)	1.1	(0.7 - 1.8)
Nepal (2022)	2,404	59.2	(57.0 - 61.5)	0.7	(0.4 - 1.1)	21.1	(19.3 - 23.0)	3.8	(3.0 - 4.7)	32.5	(30.4 - 34.6)	1.2	(0.8 - 1.7)
Pakistan (2012/13)	968	61.3	(58.0 - 64.5)	1.8	(1.1 - 2.8)	35.7	(32.6 - 38.9)	6.1	(4.6 - 7.8)	11.0	(9.0 - 13.2)	6.7	(5.2 - 8.5)
Pakistan (2017/18)	632	46.9	(43.0 - 51.1)	2.6	(1.5 - 4.2)	26.8	(23.3 - 30.5)	4.0	(2.6 - 5.8)	12.4	(9.8 - 15.2)	1.1	(0.5 - 2.2)
Philippines (1998)	482	53.6	(48.5 - 59.0)	1.9	(0.8 - 3.8)	33.1	(28.2 - 38.0)	4.8	(2.9 - 7.3)	4.2	(2.5 - 6.6)	9.7	(7.0 - 13.0)
Philippines (2003)	698	53.6	(49.6 - 57.8)	1.0	(0.4 - 2.1)	29.5	(25.9 - 33.3)	9.7	(7.5 - 12.3)	5.6	(3.9 - 7.7)	7.8	(5.8 - 10.2)
Philippines (2022)	1,354	47.3	(44.3 - 50.4)	1.7	(1.0 - 2.6)	27.8	(25.1 - 30.6)	9.2	(7.6 - 11.0)	7.1	(5.6 - 8.7)	1.5	(0.9 - 2.4)
Tajikistan (2012)	165	44.0	(36.6 - 52.1)	0.0	(0.0 - 0.0)	13.5	(8.7 - 19.2)	15.2	(10.2 - 21.2)	12.0	(7.5 - 17.6)	3.3	(1.3 - 6.8)
Tajikistan (2017)	120	42.2	(32.9 - 52.9)	1.3	(0.2 - 5.2)	20.4	(12.9 - 29.1)	6.7	(2.9 - 12.5)	13.9	(7.9 - 21.5)	0.0	(0.0 0.0)
**Latin America & Caribbean**													
Bolivia (1994)	192	79.3	(72.5 - 85.4)	5.3	(2.6 - 9.5)	38.9	(31.4 - 46.4)	13.6	(8.9 - 19.4)	11.4	(7.1 - 16.8)	10.1	(6.2 - 15.3)
Brazil (1996)	393	64.9	(59.8 - 69.9)	4.5	(2.6 - 7.1)	28.3	(23.6 - 33.1)	5.5	(3.4 - 8.3)	6.8	(4.5 - 9.7)	19.8	(15.8 - 24.1)
Colombia (1990)	466	65.2	(60.3 - 70.1)	9.3	(6.6 - 12.5)	33.1	(28.3 - 37.9)	5.9	(3.9 - 8.6)	8.5	(5.9 - 11.6)	8.4	(5.8 - 11.5)
Colombia (1995)	698	69.3	(65.6 - 72.9)	5.3	(3.8 - 7.3)	35.4	(31.7 - 39.1)	10.4	(8.2 - 12.9)	12.6	(10.1 - 15.3)	5.6	(4.1 - 7.6)
Colombia (2000)	1,172	64.2	(61.3 - 67.2)	5.0	(3.8 - 6.4)	28.9	(26.2 - 31.7)	12.2	(10.3 - 14.2)	14.4	(12.4 - 16.7)	3.7	(2.7 - 5.0)
Colombia (2005)	5,373	53.3	(51.8 - 54.8)	5.1	(4.5 - 5.8)	23.6	(22.4 - 24.9)	10.6	(9.7 - 11.6)	11.1	(10.2 - 12.1)	2.8	(2.3 - 3.3)
Colombia (2010)	9,528	50.2	(49.1 - 51.4)	3.9	(3.5 - 4.4)	21.9	(21.0 - 22.9)	11.2	(10.5 - 11.9)	10.4	(9.7 - 11.1)	2.8	(2.5 - 3.2)

Colombia (2015/16)	9,289	48.0	(46.9 - 49.1)	3.1	(2.7 - 3.5)	18.6	(17.7 - 19.5)	14.6	(13.8 - 15.4)	8.3	(7.7 - 9.0)	3.4	(3.0 - 3.8)
Dominican Republic (	1,050	69.1	(66.2 - 72.0)	4.6	(3.4 - 6.0)	35.7	(32.8 - 38.6)	8.7	(7.1 - 10.6)	11.6	(9.7 - 13.7)	8.5	(6.9 - 10.3)
Guatemala (1995)	274	59.3	(53.8 - 64.9)	2.5	(1.2 - 4.6)	21.1	(16.8 - 25.7)	10.5	(7.5 - 14.1)	6.4	(4.0 - 9.5)	18.8	(14.7 - 23.3)
Guatemala (1998/99)	312	58.5	(53.5 - 63.5)	4.0	(2.4 - 6.2)	34.2	(29.6 - 38.9)	9.8	(7.1 - 13.1)	6.4	(4.2 - 9.3)	4.0	(2.4 - 6.2)
Guatemala (2014/15)	4,753	29.7	(28.4 - 31.1)	2.6	(2.1 - 3.1)	14.1	(13.1 - 15.1)	3.5	(3.0 - 4.1)	8.2	(7.4 - 9.0)	1.4	(1.1 - 1.7)
Honduras (2011/12)	6,497	49.6	(48.3 - 51.0)	1.7	(1.4 - 2.0)	26.8	(25.6 - 27.9)	5.5	(5.0 - 6.2)	11.8	(11.0 - 12.7)	3.8	(3.3 - 4.3)
Nicaragua (1998)	993	62.2	(59.0 - 65.5)	4.5	(3.2 - 6.1)	27.6	(24.7 - 30.6)	8.1	(6.5 - 10.0)	9.4	(7.6 - 11.4)	12.6	(10.5 - 14.9)
Paraguay (1990)	740	72.2	(68.9 - 75.5)	8.0	(6.1 - 10.1)	34.5	(31.1 - 37.9)	6.0	(4.4 - 7.8)	12.4	(10.2 - 15.0)	11.3	(9.2 - 13.7)
Peru (1991/92)	722	68.5	(64.8 - 72.1)	4.0	(2.7 - 5.8)	37.1	(33.3 - 40.9)	12.8	(10.3 - 15.5)	7.0	(5.2 - 9.2)	7.6	(5.7 - 9.8)
Peru (1996)	2,925	55.7	(53.6 - 58.0)	2.3	(1.7 - 3.0)	31.0	(29.0 - 33.0)	7.5	(6.4 - 8.7)	5.5	(4.6 - 6.6)	9.3	(8.1 - 10.6)
Peru (2000)	5,474	42.1	(40.7 - 43.7)	1.1	(0.8 - 1.4)	26.3	(25.0 - 27.7)	5.8	(5.1 - 6.5)	6.2	(5.5 - 7.0)	2.7	(2.3 - 3.3)
Peru (2004/6)	4,402	45.7	(44.0 - 47.5)	0.7	(0.4 - 1.0)	30.7	(29.1 - 32.3)	6.6	(5.8 - 7.5)	4.9	(4.2 - 5.7)	2.8	(2.3 - 3.4)
Peru (2007/8)	5,831	48.1	(46.7 - 49.6)	0.8	(0.6 - 1.1)	31.4	(30.1 - 32.7)	7.6	(6.9 - 8.4)	4.8	(4.2 - 5.4)	3.5	(3.0 - 4.0)
Peru (2009)	6,873	46.9	(45.6 - 48.2)	1.1	(0.9 - 1.4)	28.6	(27.4 - 29.8)	6.7	(6.1 - 7.4)	5.8	(5.2 - 6.5)	4.7	(4.1 - 5.2)
Peru (2010)	6,522	48.0	(46.6 - 49.3)	1.0	(0.8 - 1.3)	30.7	(29.4 - 31.9)	6.4	(5.7 - 7.0)	6.1	(5.5 - 6.8)	3.8	(3.3 - 4.3)
Peru (2011)	6,288	47.4	(46.0 - 48.8)	1.2	(1.0 - 1.6)	31.0	(29.7 - 32.2)	5.7	(5.1 - 6.4)	5.6	(5.0 - 6.3)	3.8	(3.4 - 4.4)
Peru (2012)	6,835	49.3	(48.1 - 50.6)	1.0	(0.8 - 1.3)	33.1	(31.9 - 34.3)	5.2	(4.6 - 5.7)	6.7	(6.1 - 7.4)	3.4	(2.9 - 3.8)

CI= Confidence Interval

S8.4 Table: 12-month overall estimated probabilities of discontinuation and reason-specific estimated cumulative incidence of discontinuation per 100 episodes of use

Condom

	No. of episodes	All reasons		Became pregnant while using		Side effect/health concerns		Other method-related		Wanted pregnancy/no need		Other/not stated	
		Rate	95%CI	Rate	95%CI	Rate	95%CI	Rate	95%CI	Rate	95%CI	Rate	95%CI
**Sub-Saharan Africa**													
Angola (2015/16)	724	32.9	(29.5 - 36.6)	1.4	(0.7 - 2.6)	3.4	(2.3 - 5.0)	5.5	(4.1 - 7.3)	13.4	(11.0 - 16.1)	9.1	(7.1 - 11.4)
Benin (2017/18)	452	30.9	(26.3 - 36.1)	2.1	(1.0 - 4.1)	0.0	(0.0 0.0)	3.1	(1.6 - 5.3)	20.8	(16.7 - 25.3)	4.9	(3.0 - 7.4)
Burkina Faso (2010)	588	16.5	(13.6 - 20.0)	1.7	(0.8 - 3.1)	0.3	(0.1 - 1.3)	1.9	(1.0 - 3.4)	8.0	(5.9 - 10.5)	4.6	(3.0 - 6.6)
Burkina Faso (2021)	722	21.4	(18.4 - 24.9)	1.4	(0.7 - 2.6)	0.1	(0.0 - 0.7)	5.2	(3.6 - 7.1)	8.6	(6.5 - 11.0)	6.2	(4.5 - 8.3)
Burundi (2010/11)	140	38.4	(28.4 - 50.4)	2.5	(0.6 - 7.2)	0.7	(0.0 - 4.6)	7.0	(2.5 - 14.6)	15.2	(8.4 - 23.9)	13.0	(6.6 - 21.5)
Comoros (2012)	123	23.4	(16.2 - 33.2)	0.0	(0.0 0.0)	5.8	(2.3 - 11.5)	1.4	(0.2 - 5.5)	6.2	(2.5 - 12.3)	10.0	(5.1 - 16.8)
Côte d'Ivoire (2021)	685	33.2	(29.9 - 36.8)	0.8	(0.3 - 1.6)	0.7	(0.3 - 1.5)	5.8	(4.3 - 7.6)	17.2	(14.6 - 20.0)	8.8	(6.9 - 11.0)
Ethiopia (2005)	148	44.1	(33.1 - 56.9)	1.3	(0.1 - 7.1)	3.6	(0.8 - 10.1)	9.2	(3.9 - 17.4)	21.0	(12.2 - 31.3)	9.0	(3.6 - 17.5)
Gabon (2019/21)	1,618	46.9	(44.4 - 49.4)	1.6	(1.1 - 2.3)	0.3	(0.1 - 0.7)	6.9	(5.7 - 8.1)	24.3	(22.2 - 26.4)	13.8	(12.2 - 15.5)
Ghana (2014)	279	37.3	(31.4 - 43.9)	1.8	(0.6 - 4.5)	0.7	(0.1 - 2.5)	5.3	(2.9 - 8.6)	24.7	(19.4 - 30.3)	4.8	(2.6 - 8.0)
Ghana (2022/23)	494	49.8	(45.4 - 54.5)	2.6	(1.4 - 4.3)	1.6	(0.7 - 3.0)	12.1	(9.4 - 15.2)	28.1	(24.2 - 32.1)	5.5	(3.7 - 7.8)
Guinea (2018)	317	62.4	(57.0 - 67.9)	2.9	(1.5 - 5.2)	0.3	(0.0 - 1.6)	7.9	(5.3 - 11.2)	27.2	(22.3 - 32.2)	24.1	(19.6 - 28.9)
Kenya (1998)	279	62.9	(57.3 - 68.6)	3.5	(1.8 - 6.1)	0.4	(0.0 - 1.8)	16.2	(12.2 - 20.7)	23.5	(18.8 - 28.5)	19.3	(15.0 - 24.0)
Kenya (2003)	355	62.2	(56.8 - 67.6)	3.7	(1.9 - 6.2)	0.3	(0.0 - 1.7)	14.4	(10.8 - 18.5)	25.6	(20.9 - 30.5)	18.3	(14.2 - 22.7)
Kenya (2014)	650	46.0	(42.6 - 49.7)	1.9	(1.0 - 3.1)	0.8	(0.3 - 1.7)	3.9	(2.7 - 5.5)	27.2	(24.2 - 30.4)	12.2	(10.0 - 14.6)
Kenya (2022)	675	33.1	(29.6 - 36.9)	1.3	(0.6 - 2.5)	3.0	(1.9 - 4.5)	8.1	(6.2 - 10.3)	17.6	(14.7 - 20.6)	3.2	(2.1 - 4.8)
Lesotho (2014)	1,475	24.2	(22.0 - 26.5)	3.5	(2.6 - 4.5)	0.6	(0.3 - 1.1)	6.4	(5.2 - 7.8)	8.4	(7.0 - 9.9)	5.3	(4.2 - 6.6)
Liberia (2019/20)	106	40.5	(33.0 - 48.9)	0.0	(0.0 0.0)	0.1	(0.0 - 3.0)	21.7	(15.4 - 28.6)	13.1	(8.3 - 19.2)	5.5	(2.8 - 9.6)
Madagascar (2021)	165	49.0	(40.9 - 57.6)	2.4	(0.7 - 6.2)	4.0	(1.7 - 8.1)	11.9	(7.3 - 17.9)	21.0	(14.7 - 28.0)	9.6	(5.5 - 15.1)
Malawi (2004/5)	511	64.2	(59.5 - 68.9)	2.6	(1.3 - 4.7)	1.4	(0.6 - 2.9)	16.7	(13.4 - 20.3)	27.4	(23.2 - 31.8)	16.0	(12.8 - 19.6)
Malawi (2015/16)	1,332	62.1	(59.3 - 65.0)	1.5	(0.9 - 2.3)	2.5	(1.7 - 3.6)	20.7	(18.4 - 23.0)	25.7	(23.2 - 28.2)	11.8	(10.0 - 13.7)
Mozambique (2011)	792	40.2	(36.0 - 44.8)	2.1	(1.0 - 3.6)	2.2	(1.1 - 3.9)	6.4	(4.5 - 8.8)	14.4	(11.5 - 17.7)	15.2	(12.2 - 18.5)
Mozambique (2022/2	621	31.8	(27.6 - 36.5)	0.4	(0.1 - 1.3)	0.4	(0.1 - 1.6)	10.8	(8.1 - 13.9)	11.2	(8.4 - 14.5)	9.0	(6.6 - 11.8)
Namibia (2013)	1,737	17.3	(15.4 - 19.3)	3.5	(2.7 - 4.6)	0.6	(0.3 - 1.1)	3.0	(2.2 - 3.9)	4.5	(3.5 - 5.6)	5.7	(4.6 - 6.9)
Nigeria (2013)	2,178	21.1	(19.2 - 23.1)	1.9	(1.4 - 2.7)	0.5	(0.2 - 0.9)	2.0	(1.4 - 2.7)	11.5	(10.0 - 13.0)	5.2	(4.3 - 6.3)
Nigeria (2018)	1,460	35.4	(32.8 - 38.0)	4.7	(3.6 - 5.9)	0.9	(0.5 - 1.5)	6.2	(5.0 - 7.5)	19.5	(17.4 - 21.7)	4.1	(3.2 - 5.3)
Rwanda (2010/11)	270	28.2	(22.5 - 35.0)	5.4	(2.9 - 9.2)	0.4	(0.0 - 2.0)	8.5	(5.2 - 12.8)	4.4	(2.2 - 7.9)	9.5	(5.9 - 14.0)
Rwanda (2014/15)	361	40.7	(35.3 - 46.5)	6.3	(3.9 - 9.3)	0.4	(0.0 - 2.0)	12.8	(9.4 - 16.8)	9.2	(6.2 - 12.9)	12.0	(8.7 - 15.8)
Rwanda (2019/20)	536	44.5	(40.0 - 49.2)	2.5	(1.3 - 4.2)	0.7	(0.2 - 1.8)	20.7	(17.2 - 24.5)	13.0	(10.1 - 16.2)	7.6	(5.4 - 10.2)
Senegal (2010/11)	182	49.3	(42.3 - 56.8)	3.3	(1.4 - 6.5)	0.0	(0.0 0.0)	13.7	(9.2 - 19.1)	16.8	(11.9 - 22.3)	15.6	(10.7 - 21.3)
Sierra Leone (2013)	135	42.4	(34.5 - 51.3)	3.0	(1.0 - 6.9)	2.6	(0.8 - 6.4)	15.0	(9.6 - 21.6)	12.7	(7.8 - 18.9)	9.0	(5.0 - 14.6)
South Africa (2016)	1,010	30.7	(27.8 - 33.8)	3.6	(2.6 - 5.0)	1.7	(1.0 - 2.7)	8.2	(6.5 - 10.1)	9.5	(7.7 - 11.4)	7.8	(6.1 - 9.6)
Tanzania (2004/5)	514	47.1	(43.0 - 51.4)	1.6	(0.8 - 3.0)	2.5	(1.4 - 4.1)	13.1	(10.5 - 16.1)	16.8	(13.8 - 20.0)	13.0	(10.4 - 15.9)

Tanzania (2015/16)	517	29.4	(25.6 - 33.6)	1.7	(0.8 - 3.3)	1.6	(0.8 - 2.9)	8.9	(6.6 - 11.6)	12.0	(9.4 - 15.0)	5.2	(3.4 - 7.4)
Tanzania (2022)	312	39.4	(33.9 - 45.4)	1.3	(0.4 - 3.3)	2.3	(1.0 - 4.5)	8.0	(5.3 - 11.5)	18.3	(14.1 - 22.9)	9.5	(6.5 - 13.2)
Uganda (2011)	439	42.1	(37.2 - 47.3)	3.9	(2.2 - 6.3)	0.7	(0.2 - 1.9)	5.9	(3.8 - 8.6)	23.3	(19.2 - 27.6)	8.3	(5.8 - 11.3)
Zambia (2013/14)	849	31.7	(28.4 - 35.3)	1.8	(1.0 - 3.0)	0.2	(0.0 - 0.8)	11.4	(9.2 - 13.9)	7.6	(5.8 - 9.8)	10.7	(8.6 - 13.0)
Zambia (2018/19)	611	46.5	(42.5 - 50.7)	0.8	(0.3 - 1.8)	1.5	(0.7 - 2.7)	23.1	(19.7 - 26.6)	13.9	(11.2 - 16.8)	7.3	(5.4 - 9.5)
Zimbabwe (1994)	323	47.2	(41.6 - 53.2)	4.2	(2.3 - 7.0)	2.1	(0.9 - 4.2)	9.5	(6.5 - 13.2)	22.9	(18.2 - 27.9)	8.5	(5.6 - 12.1)
Zimbabwe (1999)	233	45.3	(38.8 - 52.4)	3.5	(1.5 - 6.8)	1.2	(0.3 - 3.7)	11.0	(7.2 - 15.6)	15.7	(11.2 - 20.9)	13.9	(9.7 - 18.9)
Zimbabwe (2005/6)	426	57.7	(52.7 - 62.8)	3.2	(1.7 - 5.3)	1.9	(0.8 - 3.8)	9.4	(6.8 - 12.6)	30.6	(26.0 - 35.3)	12.6	(9.6 - 16.2)
Zimbabwe (2010/11)	536	40.4	(36.0 - 45.1)	1.8	(0.8 - 3.4)	1.6	(0.7 - 3.0)	12.2	(9.4 - 15.3)	17.6	(14.2 - 21.2)	7.2	(5.1 - 9.8)
Zimbabwe (2015)	666	39.7	(35.6 - 44.1)	2.4	(1.3 - 4.0)	0.7	(0.2 - 1.7)	11.7	(9.1 - 14.6)	18.9	(15.6 - 22.4)	6.1	(4.3 - 8.4)
**North Africa Western Asia and Europe**													
Albania (2017/18)	182	46.6	(38.9 - 55.2)	0.0	(0.0 0.0)	1.8	(0.5 - 4.9)	14.1	(9.1 - 20.3)	10.8	(6.5 - 16.4)	19.9	(13.9 - 26.7)
Armenia (2000)	398	39.3	(34.7 - 44.4)	12.4	(9.4 - 15.9)	2.6	(1.3 - 4.5)	10.0	(7.3 - 13.2)	8.0	(5.6 - 11.0)	6.4	(4.3 - 9.0)
Armenia (2005)	365	31.4	(26.9 - 36.5)	7.1	(4.8 - 10.1)	0.4	(0.0 - 1.7)	10.1	(7.3 - 13.5)	10.6	(7.8 - 13.9)	3.2	(1.7 - 5.3)
Armenia (2010)	410	15.1	(12.0 - 18.8)	2.7	(1.5 - 4.6)	0.0	(0.0 - 0.0)	2.2	(1.1 - 3.9)	8.9	(6.5 - 11.8)	1.2	(0.5 - 2.6)
Armenia (2015/16)	468	17.7	(14.5 - 21.6)	3.0	(1.7 - 4.9)	0.4	(0.1 - 1.4)	2.0	(1.0 - 3.6)	11.4	(8.7 - 14.6)	0.9	(0.3 - 2.1)
Azerbaijan (2006)	187	50.2	(42.3 - 58.8)	9.9	(5.7 - 15.5)	0.0	(0.0 - 0.0)	13.3	(8.5 - 19.2)	14.2	(9.0 - 20.5)	12.9	(8.1 - 18.9)
Egypt (1992/93)	338	49.3	(43.8 - 55.2)	3.8	(2.1 - 6.5)	10.6	(7.5 - 14.5)	19.5	(15.2 - 24.1)	12.4	(9.0 - 16.5)	2.9	(1.4 - 5.3)
Egypt (1995/96)	427	58.8	(53.7 - 64.0)	10.5	(7.6 - 14.0)	3.7	(2.1 - 6.0)	19.3	(15.5 - 23.5)	9.1	(6.4 - 12.4)	16.1	(12.6 - 20.0)
Egypt (2000)	218	53.5	(46.3 - 61.1)	12.9	(8.4 - 18.3)	4.7	(2.2 - 8.6)	18.1	(12.9 - 24.0)	13.1	(8.6 - 18.5)	4.8	(2.3 - 8.5)
Egypt (2003)	115	48.1	(38.3 - 58.9)	8.9	(4.2 - 15.9)	1.7	(0.3 - 6.0)	21.8	(14.3 - 30.4)	5.8	(2.3 - 11.8)	9.8	(4.7 - 17.2)
Egypt (2005)	246	39.2	(32.8 - 46.2)	7.1	(4.1 - 11.2)	2.0	(0.7 - 4.7)	17.8	(13.0 - 23.3)	8.0	(4.8 - 12.2)	4.3	(2.1 - 7.5)
Egypt (2008)	130	32.3	(24.6 - 41.7)	8.1	(4.0 - 14.0)	0.0	(0.0 - 0.0)	9.9	(5.4 - 16.1)	4.1	(1.5 - 9.0)	10.2	(5.6 - 16.3)
Egypt (2014)	131	33.0	(24.7 - 43.1)	6.4	(2.7 - 12.2)	2.7	(0.7 - 7.3)	10.5	(5.5 - 17.3)	8.4	(3.9 - 14.9)	5.0	(1.9 - 10.2)
Jordan (1990)	131	66.8	(58.3 - 75.1)	12.0	(7.0 - 18.3)	12.6	(7.5 - 19.2)	13.4	(8.3 - 19.7)	11.7	(6.8 - 18.1)	17.1	(11.1 - 24.2)
Jordan (1997)	383	68.4	(63.4 - 73.3)	20.8	(16.7 - 25.2)	5.3	(3.3 - 8.0)	12.1	(9.1 - 15.6)	10.1	(7.3 - 13.5)	20.1	(16.1 - 24.4)
Jordan (2002)	465	58.0	(53.4 - 62.6)	13.2	(10.3 - 16.6)	7.7	(5.5 - 10.5)	14.8	(11.7 - 18.2)	12.9	(9.9 - 16.2)	9.4	(6.9 - 12.2)
Jordan (2007)	930	44.6	(41.4 - 47.9)	10.1	(8.2 - 12.2)	3.3	(2.3 - 4.6)	8.9	(7.2 - 10.8)	15.4	(13.1 - 17.8)	6.9	(5.4 - 8.6)
Jordan (2009)	1,210	53.6	(50.7 - 56.5)	10.5	(8.8 - 12.3)	6.5	(5.2 - 8.0)	11.4	(9.6 - 13.2)	17.8	(15.7 - 20.0)	7.5	(6.1 - 9.0)
Jordan (2012)	1,243	39.1	(36.5 - 41.9)	11.6	(10.0 - 13.5)	2.6	(1.9 - 3.6)	9.8	(8.2 - 11.5)	10.2	(8.6 - 11.9)	4.9	(3.8 - 6.1)
Jordan (2017/18)	816	33.7	(30.4 - 37.2)	3.4	(2.3 - 4.9)	1.0	(0.5 - 2.0)	9.6	(7.6 - 11.8)	16.7	(14.1 - 19.5)	3.0	(1.9 - 4.4)
Jordan (2023)	760	29.9	(26.3 - 33.8)	3.9	(2.5 - 5.6)	1.4	(0.7 - 2.6)	4.8	(3.3 - 6.7)	18.2	(15.2 - 21.5)	1.6	(0.8 - 2.9)
Moldova (2005)	925	41.4	(37.9 - 45.1)	5.0	(3.6 - 6.8)	0.5	(0.2 - 1.2)	13.3	(11.0 - 15.8)	15.6	(13.1 - 18.3)	7.0	(5.3 - 9.0)
Morocco (1992)	108	61.4	(52.1 - 70.8)	10.6	(5.6 - 17.4)	0.0	(0.0 - 0.0)	10.7	(5.7 - 17.6)	7.9	(3.7 - 14.1)	32.3	(23.6 - 41.3)
Morocco (2003/4)	244	52.2	(45.9 - 58.9)	2.5	(1.0 - 5.2)	0.9	(0.2 - 2.9)	32.1	(26.2 - 38.2)	12.4	(8.6 - 16.9)	4.3	(2.2 - 7.5)
Türkiye (1993)	738	50.1	(46.4 - 54.0)	8.4	(6.4 - 10.6)	0.5	(0.1 - 1.2)	13.8	(11.3 - 16.5)	6.8	(5.1 - 8.9)	20.6	(17.7 - 23.7)
Türkiye (1998)	737	44.3	(40.6 - 48.2)	5.8	(4.2 - 7.8)	0.6	(0.2 - 1.4)	10.1	(8.0 - 12.5)	9.8	(7.7 - 12.2)	18.1	(15.3 - 21.1)
Türkiye (2003/4)	1,240	46.8	(43.9 - 49.8)	5.7	(4.5 - 7.2)	0.2	(0.1 - 0.7)	14.4	(12.4 - 16.5)	10.9	(9.2 - 12.8)	15.6	(13.5 - 17.7)
Türkiye (2018/19)	970	29.9	(26.9 - 33.0)	5.5	(4.1 - 7.1)	1.2	(0.6 - 2.1)	5.3	(4.0 - 6.9)	11.8	(9.8 - 14.0)	6.1	(4.7 - 7.8)
Ukraine (2007)	1,559	23.2	(21.2 - 25.4)	2.8	(2.1 - 3.7)	0.2	(0.1 - 0.6)	5.1	(4.0 - 6.3)	9.4	(8.0 - 10.9)	5.7	(4.6 - 7.0)
Yemen (2013)	159	47.9	(39.7 - 56.8)	8.3	(4.4 - 13.8)	4.9	(2.1 - 9.3)	11.7	(7.0 - 17.8)	11.4	(6.7 - 17.4)	11.6	(6.9 - 17.8)

**Central, South & Southeast Asia**												
Bangladesh (1993/94)	852	73.1	(69.7 - 76.4)	5.7	(4.2 - 7.6)	13.9	(11.4 - 16.5)	8.9	(7.0 - 11.1)	17.3	(14.6 - 20.2)	27.3	(24.1 - 30.5)
Bangladesh (1996/97)	781	66.2	(62.5 - 69.8)	6.4	(4.7 - 8.4)	11.4	(9.2 - 13.9)	15.3	(12.8 - 18.1)	14.4	(11.8 - 17.1)	18.7	(15.9 - 21.6)
Bangladesh (1999/0)	1,203	68.6	(65.6 - 71.5)	6.6	(5.2 - 8.3)	9.7	(8.0 - 11.6)	13.5	(11.5 - 15.7)	15.6	(13.4 - 17.9)	23.1	(20.6 - 25.7)
Bangladesh (2004)	1,454	71.9	(69.3 - 74.5)	6.3	(5.0 - 7.7)	7.0	(5.7 - 8.5)	19.8	(17.6 - 22.0)	15.1	(13.2 - 17.2)	23.8	(21.5 - 26.2)
Bangladesh (2011)	1,578	48.1	(45.5 - 50.9)	7.7	(6.4 - 9.2)	5.6	(4.5 - 7.0)	17.0	(15.0 - 19.0)	12.7	(11.0 - 14.6)	5.1	(4.0 - 6.4)
Bangladesh (2014)	1,588	41.6	(39.1 - 44.4)	3.7	(2.8 - 4.8)	5.8	(4.7 - 7.1)	8.9	(7.4 - 10.5)	16.2	(14.3 - 18.2)	7.0	(5.8 - 8.4)
Bangladesh (2017/18)	2,345	46.3	(44.1 - 48.5)	6.0	(5.0 - 7.1)	6.1	(5.1 - 7.2)	15.8	(14.3 - 17.4)	16.3	(14.7 - 17.9)	2.2	(1.6 - 2.9)
Bangladesh (2022)	1,994	38.3	(36.0 - 40.7)	3.7	(2.9 - 4.7)	3.3	(2.5 - 4.2)	9.4	(8.0 - 10.8)	17.6	(15.8 - 19.4)	4.4	(3.5 - 5.4)
Cambodia (2010/11)	346	27.4	(22.6 - 33.1)	3.7	(1.9 - 6.5)	3.3	(1.7 - 5.8)	8.9	(6.0 - 12.6)	6.2	(3.8 - 9.3)	5.3	(3.1 - 8.4)
Cambodia (2014)	342	39.1	(33.6 - 45.1)	3.0	(1.4 - 5.5)	5.4	(3.2 - 8.4)	9.5	(6.5 - 13.2)	17.1	(13.0 - 21.8)	4.1	(2.2 - 6.9)
Cambodia (2021/22)	187	31.8	(25.9 - 38.7)	1.0	(0.2 - 3.5)	7.3	(4.4 - 11.2)	5.2	(2.8 - 8.7)	16.1	(11.3 - 21.6)	2.2	(0.8 - 4.8)
India (2005/6)	7,872	46.1	(44.9 - 47.4)	3.6	(3.2 - 4.1)	2.9	(2.5 - 3.3)	12.1	(11.3 - 12.9)	20.5	(19.5 - 21.5)	7.1	(6.5 - 7.8)
India (2015/16)	42,893	47.9	(47.4 - 48.4)	3.0	(2.8 - 3.2)	3.4	(3.2 - 3.5)	10.3	(10.0 - 10.6)	22.1	(21.7 - 22.6)	9.2	(8.9 - 9.5)
India (2019/21)	84,274	60.3	(59.9 - 60.6)	2.8	(2.7 - 2.9)	4.0	(3.8 - 4.1)	15.6	(15.4 - 15.9)	25.5	(25.2 - 25.8)	12.4	(12.2 - 12.6)
Indonesia (1991)	458	52.8	(47.9 - 57.9)	9.6	(6.9 - 12.8)	1.5	(0.6 - 3.1)	23.6	(19.5 - 27.9)	9.7	(7.0 - 13.0)	8.5	(6.0 - 11.5)
Indonesia (1994)	349	52.6	(47.0 - 58.4)	5.6	(3.4 - 8.6)	2.0	(0.9 - 4.1)	21.8	(17.4 - 26.5)	11.7	(8.4 - 15.6)	11.4	(8.1 - 15.4)
Indonesia (1997)	260	38.2	(31.9 - 45.2)	6.4	(3.6 - 10.3)	0.6	(0.1 - 2.7)	15.8	(11.3 - 21.0)	10.5	(6.8 - 15.3)	4.7	(2.4 - 8.1)
Indonesia (2002/3)	299	38.9	(32.7 - 45.7)	3.1	(1.4 - 6.1)	2.5	(1.0 - 5.3)	17.2	(12.7 - 22.3)	7.6	(4.6 - 11.6)	8.5	(5.2 - 12.7)
Indonesia (2007)	486	39.3	(34.9 - 44.1)	5.0	(3.2 - 7.3)	3.8	(2.3 - 6.0)	10.5	(7.8 - 13.5)	13.4	(10.4 - 16.8)	6.7	(4.7 - 9.2)
Indonesia (2012)	608	32.4	(28.7 - 36.4)	2.1	(1.2 - 3.5)	3.4	(2.1 - 5.1)	14.1	(11.4 - 17.0)	9.3	(7.0 - 11.9)	3.6	(2.2 - 5.3)
Indonesia (2017)	894	27.2	(24.5 - 30.2)	2.2	(1.4 - 3.3)	2.3	(1.5 - 3.4)	9.5	(7.7 - 11.5)	11.2	(9.3 - 13.2)	2.1	(1.3 - 3.1)
Kazakhstan (1999)	581	60.1	(55.8 - 64.4)	10.4	(7.9 - 13.3)	0.5	(0.1 - 1.5)	26.8	(23.0 - 30.7)	14.8	(11.9 - 18.0)	7.6	(5.5 - 10.1)
Kyrgyz Republic (201	614	30.4	(26.9 - 34.3)	11.4	(9.0 - 14.2)	0.4	(0.1 - 1.2)	3.9	(2.5 - 5.6)	9.8	(7.6 - 12.4)	4.9	(3.4 - 6.9)
Maldives (2009)	1,051	45.1	(41.9 - 48.4)	4.2	(3.1 - 5.7)	4.8	(3.5 - 6.3)	5.0	(3.7 - 6.5)	17.0	(14.7 - 19.5)	14.1	(12.0 - 16.4)
Nepal (2011)	1,131	65.2	(62.0 - 68.5)	3.8	(2.6 - 5.3)	0.7	(0.3 - 1.4)	10.8	(8.8 - 13.0)	45.8	(42.4 - 49.1)	4.2	(3.0 - 5.7)
Nepal (2016)	1,196	70.2	(67.3 - 73.1)	2.6	(1.7 - 3.8)	1.1	(0.6 - 1.9)	10.4	(8.6 - 12.5)	53.0	(49.9 - 56.1)	3.1	(2.1 - 4.3)
Nepal (2022)	989	62.3	(59.1 - 65.6)	2.4	(1.5 - 3.6)	0.6	(0.3 - 1.4)	9.6	(7.7 - 11.7)	47.3	(44.0 - 50.6)	2.4	(1.6 - 3.6)
Pakistan (2012/13)	1,973	39.2	(36.9 - 41.5)	7.8	(6.6 - 9.2)	3.4	(2.6 - 4.3)	6.8	(5.7 - 8.0)	14.7	(13.0 - 16.4)	6.5	(5.4 - 7.7)
Pakistan (2017/18)	1,570	35.3	(32.8 - 37.8)	6.6	(5.4 - 8.0)	2.4	(1.7 - 3.2)	2.9	(2.1 - 3.8)	20.3	(18.3 - 22.4)	3.1	(2.3 - 4.1)
Philippines (1993)	200	61.3	(54.0 - 68.6)	15.4	(10.4 - 21.3)	4.0	(1.8 - 7.4)	20.7	(15.2 - 26.7)	8.7	(5.1 - 13.6)	12.5	(8.2 - 17.7)
Philippines (1998)	331	61.0	(55.7 - 66.3)	8.5	(5.8 - 11.8)	5.7	(3.5 - 8.5)	23.8	(19.4 - 28.5)	7.7	(5.1 - 10.9)	15.4	(11.7 - 19.5)
Philippines (2003)	366	58.4	(53.0 - 63.8)	8.1	(5.4 - 11.3)	11.2	(8.1 - 14.9)	26.0	(21.4 - 30.9)	7.3	(4.8 - 10.4)	5.8	(3.6 - 8.7)
Philippines (2022)	458	37.4	(33.0 - 42.1)	3.2	(1.8 - 5.2)	2.6	(1.4 - 4.3)	12.7	(9.8 - 16.0)	14.3	(11.3 - 17.7)	4.6	(3.0 - 6.8)
Tajikistan (2012)	257	33.4	(27.3 - 40.5)	5.3	(2.8 - 9.1)	0.4	(0.0 - 2.3)	9.0	(5.5 - 13.4)	9.8	(6.1 - 14.4)	8.9	(5.5 - 13.3)
Tajikistan (2017)	386	35.2	(30.3 - 40.7)	1.7	(0.7 - 3.7)	1.4	(0.5 - 3.1)	10.3	(7.3 - 13.9)	16.5	(12.7 - 20.8)	5.3	(3.2 - 8.1)
Vietnam (1997)	405	33.3	(28.6 - 38.6)	11.0	(8.0 - 14.6)	1.3	(0.4 - 3.0)	11.3	(8.2 - 14.9)	6.7	(4.3 - 9.7)	3.1	(1.6 - 5.3)
Vietnam (2002)	409	39.3	(34.5 - 44.5)	8.2	(5.7 - 11.3)	3.5	(1.9 - 5.8)	12.6	(9.5 - 16.2)	12.0	(8.9 - 15.5)	3.1	(1.6 - 5.2)
**Latin America & Caribbean**													
Bolivia (1994)	209	70.2	(63.5 - 76.7)	5.3	(2.8 - 9.2)	6.0	(3.2 - 9.9)	23.5	(17.7 - 29.7)	16.4	(11.6 - 22.0)	19.1	(13.9 - 24.8)
Brazil (1996)	1,196	61.8	(58.9 - 64.6)	5.1	(3.9 - 6.5)	3.7	(2.7 - 4.8)	22.4	(20.0 - 24.8)	13.5	(11.6 - 15.5)	17.1	(15.0 - 19.3)

Colombia (1990)	286	62.6	(56.5 - 68.8)	6.1	(3.6 - 9.5)	0.7	(0.1 - 2.5)	22.8	(17.8 - 28.2)	12.5	(8.7 - 17.2)	20.4	(15.7 - 25.6)
Colombia (1995)	970	66.6	(63.5 - 69.7)	4.9	(3.7 - 6.5)	0.4	(0.1 - 1.0)	30.8	(27.8 - 33.8)	19.3	(16.8 - 21.9)	11.1	(9.2 - 13.3)
Colombia (2000)	1,706	63.1	(60.7 - 65.5)	3.5	(2.6 - 4.4)	2.1	(1.5 - 2.9)	26.8	(24.7 - 29.0)	21.8	(19.8 - 23.8)	8.9	(7.6 - 10.4)
Colombia (2005)	5,717	55.3	(54.0 - 56.6)	5.0	(4.4 - 5.6)	1.6	(1.3 - 2.0)	23.5	(22.4 - 24.7)	20.4	(19.3 - 21.5)	4.7	(4.2 - 5.3)
Colombia (2010)	8,476	58.4	(57.3 - 59.5)	3.6	(3.2 - 4.0)	1.5	(1.3 - 1.8)	20.4	(19.5 - 21.3)	26.5	(25.5 - 27.5)	6.4	(5.9 - 7.0)
Colombia (2015/16)	4,812	53.7	(52.2 - 55.2)	3.6	(3.1 - 4.2)	1.8	(1.4 - 2.2)	18.8	(17.6 - 19.9)	24.3	(23.0 - 25.6)	5.2	(4.6 - 5.9)
Dominican Republic (	251	79.8	(75.0 - 84.1)	7.0	(4.5 - 10.2)	4.8	(2.8 - 7.6)	32.8	(27.6 - 38.1)	16.7	(12.8 - 21.1)	18.5	(14.4 - 23.1)
Dominican Republic (	407	83.5	(80.0 - 86.7)	6.7	(4.7 - 9.2)	2.4	(1.3 - 4.0)	29.1	(25.2 - 33.1)	20.9	(17.4 - 24.5)	24.4	(20.8 - 28.3)
Dominican Republic (	1,033	69.6	(66.7 - 72.5)	2.8	(1.9 - 4.0)	2.4	(1.6 - 3.4)	21.0	(18.6 - 23.6)	23.2	(20.6 - 25.8)	20.2	(17.9 - 22.7)
Guatemala (1995)	252	52.7	(47.8 - 57.8)	11.1	(8.2 - 14.4)	6.2	(4.1 - 8.9)	16.2	(12.7 - 20.0)	9.8	(7.1 - 13.0)	9.5	(6.8 - 12.6)
Guatemala (1998/99)	136	67.6	(61.5 - 73.6)	2.4	(1.0 - 5.0)	6.0	(3.4 - 9.6)	19.4	(14.7 - 24.6)	22.9	(17.8 - 28.5)	16.8	(12.4 - 21.8)
Guatemala (2014/15)	1,392	46.9	(44.1 - 49.8)	4.3	(3.2 - 5.6)	3.5	(2.6 - 4.7)	7.4	(6.0 - 9.0)	25.0	(22.7 - 27.5)	6.6	(5.3 - 8.1)
Honduras (2011/12)	3,343	79.3	(77.8 - 80.7)	1.3	(1.0 - 1.8)	8.8	(7.8 - 9.8)	16.4	(15.2 - 17.7)	29.6	(28.1 - 31.2)	23.1	(21.7 - 24.5)
Nicaragua (1998)	573	64.6	(60.6 - 68.6)	5.9	(4.1 - 8.0)	4.5	(3.0 - 6.5)	21.5	(18.2 - 25.0)	11.8	(9.3 - 14.6)	21.0	(17.7 - 24.4)
Paraguay (1990)	271	73.2	(67.7 - 78.5)	4.3	(2.2 - 7.2)	5.1	(2.8 - 8.3)	19.8	(15.3 - 24.8)	7.2	(4.5 - 10.7)	36.9	(31.2 - 42.6)
Peru (1991/92)	666	67.3	(63.8 - 70.9)	8.2	(6.3 - 10.4)	6.9	(5.2 - 8.9)	26.2	(23.0 - 29.4)	12.7	(10.3 - 15.3)	13.4	(11.0 - 16.1)
Peru (1996)	1,640	61.3	(59.0 - 63.7)	6.8	(5.6 - 8.0)	3.9	(3.0 - 4.8)	18.7	(16.9 - 20.5)	14.4	(12.8 - 16.1)	17.6	(15.9 - 19.4)
Peru (2000)	2,156	55.6	(53.5 - 57.8)	5.9	(5.0 - 7.0)	2.8	(2.1 - 3.5)	20.6	(18.9 - 22.3)	16.0	(14.5 - 17.5)	10.4	(9.2 - 11.7)
Peru (2004/6)	2,727	59.4	(57.5 - 61.4)	6.0	(5.1 - 7.0)	3.9	(3.2 - 4.8)	17.4	(15.9 - 18.9)	17.0	(15.5 - 18.5)	15.2	(13.8 - 16.7)
Peru (2007/8)	3,798	60.3	(58.8 - 61.8)	6.2	(5.4 - 6.9)	3.9	(3.4 - 4.5)	17.2	(16.1 - 18.4)	17.2	(16.1 - 18.4)	15.8	(14.7 - 16.9)
Peru (2009)	4,295	61.5	(60.1 - 62.9)	6.0	(5.4 - 6.7)	3.7	(3.2 - 4.2)	18.3	(17.3 - 19.4)	17.0	(15.9 - 18.0)	16.5	(15.5 - 17.6)
Peru (2010)	4,484	58.4	(57.0 - 59.8)	5.6	(4.9 - 6.2)	4.7	(4.1 - 5.3)	16.4	(15.4 - 17.5)	16.2	(15.2 - 17.3)	15.5	(14.5 - 16.5)
Peru (2011)	4,751	58.0	(56.6 - 59.3)	5.5	(4.9 - 6.1)	4.3	(3.8 - 4.9)	15.3	(14.4 - 16.3)	14.9	(14.0 - 15.9)	17.9	(16.9 - 18.9)
Peru (2012)	5,148	58.9	(57.5 - 60.2)	6.0	(5.4 - 6.7)	3.3	(2.9 - 3.8)	17.8	(16.7 - 18.8)	19.8	(18.8 - 20.9)	12.0	(11.1 - 12.9)

CI= Confidence Interval

S8.5 Table: 12-month overall estimated probabilities of discontinuation and reason-specific estimated cumulative incidence of discontinuation per 100 episodes of use

Implant

	No. of episodes	All reasons		Became pregnant while using		Side effect/health concerns		Other method-related		Wanted pregnancy/no need		Other/not stated	
		Rate	95%CI	Rate	95%CI	Rate	95%CI	Rate	95%CI	Rate	95%CI	Rate	95%CI
**Sub-Saharan Africa**													
Benin (2017/18)	999	21.4	(18.8 - 24.4)	0.1	(0.0 - 0.6)	6.2	(4.7 - 7.9)	2.3	(1.5 - 3.5)	8.8	(7.0 - 10.9)	4.0	(2.8 - 5.4)
Burkina Faso (2010)	547	3.6	(2.2 - 5.9)	0.0	(0.0 0.0)	1.0	(0.4 - 2.3)	0.4	(0.1 - 1.5)	1.8	(0.8 - 3.4)	0.5	(0.1 - 1.6)
Burkina Faso (2021)	3220	14.5	(13.2 - 15.9)	0.4	(0.2 - 0.7)	4.6	(3.8 - 5.4)	2.4	(1.9 - 3.0)	5.5	(4.7 - 6.4)	1.6	(1.2 - 2.2)
Côte d'Ivoire (2021)	863	16.0	(13.6 - 18.9)	0.1	(0.0 - 0.7)	4.3	(3.0 - 6.0)	5.2	(3.8 - 7.0)	3.9	(2.7 - 5.5)	2.5	(1.6 - 3.8)
Ethiopia (2016)	1,000	11.0	(9.1 - 13.2)	0.3	(0.1 - 0.9)	2.7	(1.8 - 4.0)	2.7	(1.8 - 3.9)	4.5	(3.2 - 6.0)	0.7	(0.3 - 1.4)
Gambia (2019/20)	744	17.9	(15.1 - 21.3)	0.1	(0.0 - 0.7)	5.9	(4.2 - 8.0)	1.2	(0.6 - 2.3)	7.0	(5.1 - 9.2)	3.8	(2.4 - 5.5)
Ghana (2014)	372	7.0	(4.6 - 10.5)	0.5	(0.1 - 1.9)	5.0	(2.9 - 7.9)	0.7	(0.2 - 2.2)	0.7	(0.1 - 2.5)	0.0	(0.0 - 0.0)
Ghana (2022/23)	1,402	24.0	(21.6 - 26.7)	0.2	(0.0 - 0.6)	16.1	(13.9 - 18.3)	1.7	(1.1 - 2.6)	4.0	(2.9 - 5.2)	2.1	(1.4 - 3.1)
Guinea (2018)	416	54.3	(49.5 - 59.2)	0.0	(0.0 0.0)	39.1	(34.4 - 43.7)	0.7	(0.2 - 2.0)	13.2	(10.0 - 16.7)	1.4	(0.5 - 2.9)
Kenya (2014)	983	8.7	(7.1 - 10.7)	0.3	(0.1 - 0.9)	6.9	(5.4 - 8.6)	0.2	(0.0 - 0.6)	1.0	(0.5 - 1.8)	0.3	(0.1 - 0.9)
Kenya (2022)	2,703	13.6	(12.3 - 15.0)	0.4	(0.2 - 0.7)	8.7	(7.6 - 9.8)	1.2	(0.9 - 1.7)	2.8	(2.2 - 3.5)	0.5	(0.3 - 0.8)
Liberia (2013)	157	8.3	(4.3 - 15.7)	0.0	(0.0 0.0)	2.5	(0.7 - 6.4)	0.0	(0.0 0.0)	5.8	(2.2 - 11.8)	0.0	(0.0 0.0)
Liberia (2019/20)	553	13.0	(10.0 - 16.8)	0.3	(0.0 - 1.5)	8.9	(6.3 - 12.0)	0.5	(0.1 - 1.7)	2.2	(1.0 - 4.1)	1.1	(0.4 - 2.7)
Madagascar (2021)	1,940	15.3	(13.7 - 17.2)	0.1	(0.0 - 0.4)	5.4	(4.4 - 6.5)	2.3	(1.6 - 3.1)	3.4	(2.6 - 4.4)	4.2	(3.3 - 5.2)
Malawi (2015/16)	2,634	7.8	(6.8 - 9.1)	0.1	(0.0 - 0.4)	4.9	(4.0 - 5.8)	0.4	(0.2 - 0.8)	1.6	(1.1 - 2.2)	0.8	(0.5 - 1.3)
Mali (2012/13)	287	19.1	(14.3 - 25.2)	1.6	(0.5 - 3.8)	5.7	(3.1 - 9.3)	1.5	(0.4 - 3.9)	5.1	(2.6 - 9.0)	5.2	(2.7 - 8.8)
Mali (2018)	799	17.8	(15.2 - 20.8)	0.0	(0.0 0.0)	7.0	(5.3 - 9.0)	0.7	(0.3 - 1.4)	7.3	(5.6 - 9.3)	2.8	(1.8 - 4.2)
Mozambique (2022/2	1,169	15.9	(13.5 - 18.6)	0.0	(0.0 0.0)	3.9	(2.7 - 5.5)	4.4	(3.1 - 6.0)	3.8	(2.6 - 5.3)	3.8	(2.6 - 5.2)
Nigeria (2018)	1,183	16.0	(13.9 - 18.5)	1.2	(0.6 - 2.0)	9.0	(7.4 - 10.9)	0.9	(0.5 - 1.7)	3.9	(2.8 - 5.3)	1.0	(0.5 - 1.8)
Rwanda (2010/11)	539	4.7	(3.1 - 7.1)	0.3	(0.0 - 1.4)	3.6	(2.1 - 5.6)	0.3	(0.0 - 1.3)	0.3	(0.0 - 1.3)	0.2	(0.0 - 1.0)
Rwanda (2014/15)	690	3.4	(2.2 - 5.2)	0.3	(0.1 - 1.1)	2.1	(1.2 - 3.5)	0.0	(0.0 - 0.0)	0.8	(0.3 - 1.9)	0.1	(0.0 - 0.9)
Rwanda (2019/20)	2,561	7.0	(6.0 - 8.2)	0.5	(0.2 - 0.8)	4.8	(4.0 - 5.8)	0.1	(0.0 - 0.4)	1.2	(0.8 - 1.8)	0.4	(0.2 - 0.7)
Senegal (2010/11)	137	22.8	(17.0 - 30.1)	1.0	(0.2 - 3.7)	7.1	(3.7 - 12.0)	2.9	(1.1 - 6.2)	1.6	(0.4 - 4.6)	10.2	(6.2 - 15.3)
Senegal (2015)	361	10.0	(7.0 - 14.0)	0.2	(0.0 - 1.5)	4.5	(2.5 - 7.3)	1.0	(0.3 - 2.9)	3.8	(2.0 - 6.5)	0.4	(0.1 - 1.8)
Senegal (2016)	504	7.3	(5.3 - 10.1)	0.0	(0.0 0.0)	3.2	(1.9 - 5.1)	0.9	(0.3 - 2.2)	2.5	(1.4 - 4.3)	0.7	(0.2 - 1.8)
Senegal (2018)	786	12.8	(10.4 - 15.6)	0.4	(0.1 - 1.3)	6.3	(4.6 - 8.3)	0.4	(0.1 - 1.1)	2.1	(1.2 - 3.4)	3.7	(2.4 - 5.3)
Senegal (2019)	734	11.2	(8.9 - 14.1)	0.4	(0.1 - 1.3)	5.1	(3.5 - 7.1)	1.2	(0.5 - 2.4)	2.7	(1.6 - 4.3)	1.7	(0.9 - 3.0)
Senegal (2023)	1,698	20.8	(18.7 - 23.0)	1.3	(0.8 - 2.0)	9.1	(7.7 - 10.7)	0.4	(0.1 - 0.8)	7.1	(5.9 - 8.6)	2.8	(2.0 - 3.7)
Sierra Leone (2013)	798	9.3	(7.2 - 11.9)	0.2	(0.0 - 0.8)	6.5	(4.8 - 8.7)	0.6	(0.2 - 1.5)	1.2	(0.5 - 2.3)	0.8	(0.3 - 1.8)
South Africa (2016)	328	12.1	(8.8 - 16.4)	0.0	(0.0 - 0.0)	10.3	(7.1 - 14.1)	1.0	(0.3 - 2.7)	0.8	(0.2 - 2.4)	0.0	(0.0 0.0)
Tanzania (2015/16)	918	10.1	(8.2 - 12.5)	0.6	(0.2 - 1.3)	6.9	(5.3 - 8.9)	0.1	(0.0 - 0.6)	2.2	(1.4 - 3.5)	0.2	(0.1 - 0.8)
Tanzania (2022)	2,336	22.1	(20.4 - 23.9)	0.3	(0.1 - 0.5)	12.4	(11.0 - 13.9)	1.3	(0.9 - 1.8)	6.7	(5.7 - 7.8)	1.4	(1.0 - 1.9)
Uganda (2011)	179	13.9	(9.0 - 21.3)	0.8	(0.1 - 3.8)	10.0	(5.6 - 15.8)	1.2	(0.2 - 5.2)	2.0	(0.5 - 5.4)	0.0	(0.0 - 0.0)

Zambia (2013/14)	724	4.7	(3.3 - 6.7)	0.1	(0.0 - 0.8)	3.8	(2.5 - 5.5)	0.0	(0.0 - 0.0)	0.4	(0.1 - 1.1)	0.5	(0.1 - 1.3)
Zambia (2018/19)	1,039	12.5	(10.5 - 14.8)	0.3	(0.1 - 1.0)	7.7	(6.0 - 9.6)	0.9	(0.4 - 1.6)	2.5	(1.7 - 3.7)	1.0	(0.5 - 1.8)
Zimbabwe (2010/11)	196	7.9	(4.5 - 13.9)	0.3	(0.0 - 2.7)	5.5	(2.5 - 10.4)	0.0	(0.0 0.0)	1.3	(0.2 - 4.3)	0.8	(0.1 - 3.3)
Zimbabwe (2015)	901	6.3	(4.8 - 8.3)	0.4	(0.1 - 1.1)	4.5	(3.1 - 6.1)	0.4	(0.1 - 1.1)	0.5	(0.1 - 1.3)	0.6	(0.2 - 1.3)
**North Africa Western Asia and Europe**													
Egypt (2005)	239	20.0	(14.6 - 27.0)	0.0	(0.0 - 0.0)	17.8	(12.3 - 24.1)	0.1	(0.0 - 2.7)	2.1	(0.6 - 5.2)	0.0	(0.0 0.0)
Egypt (2008)	144	26.0	(18.8 - 35.2)	1.6	(0.3 - 5.7)	19.1	(12.5 - 26.7)	0.8	(0.1 - 4.0)	4.5	(1.6 - 9.7)	0.0	(0.0 0.0)
Egypt (2014)	207	14.7	(10.1 - 21.1)	0.9	(0.1 - 3.4)	11.0	(6.8 - 16.3)	0.0	(0.0 - 0.0)	2.8	(1.0 - 6.3)	0.0	(0.0 0.0)
Jordan (2023)	128	22.1	(15.2 - 31.4)	0.1	(0.0 - 4.9)	14.1	(8.2 - 21.6)	0.9	(0.1 - 4.4)	7.0	(3.2 - 12.9)	0.0	(0.0 0.0)
Yemen (2013)	181	27.4	(20.9 - 35.3)	1.4	(0.3 - 4.5)	15.3	(10.1 - 21.5)	6.5	(3.3 - 11.1)	3.9	(1.6 - 7.9)	0.2	(0.0 - 2.6)
**Central, South & Southeast Asia**													
Bangladesh (2011)	189	7.6	(4.3 - 13.2)	1.1	(0.2 - 4.3)	5.9	(2.8 - 10.5)	0.0	(0.0 0.0)	0.5	(0.0 - 2.7)	0.0	(0.0 0.0)
Bangladesh (2014)	296	7.9	(5.1 - 12.0)	0.0	(0.0 0.0)	5.1	(2.9 - 8.3)	0.0	(0.0 - 0.0)	2.8	(1.2 - 5.4)	0.0	(0.0 - 0.0)
Bangladesh (2017/18)	510	13.0	(10.2 - 16.5)	0.0	(0.0 0.0)	12.2	(9.4 - 15.4)	0.0	(0.0 - 0.0)	0.9	(0.3 - 2.1)	0.0	(0.0 - 0.0)
Bangladesh (2022)	387	14.5	(11.1 - 18.9)	0.4	(0.1 - 1.6)	9.8	(6.9 - 13.3)	1.0	(0.3 - 2.5)	3.0	(1.5 - 5.4)	0.4	(0.0 - 1.8)
Cambodia (2014)	312	7.9	(5.1 - 12.2)	0.0	(0.0 0.0)	4.2	(2.2 - 7.3)	1.3	(0.3 - 3.7)	1.7	(0.5 - 4.3)	0.6	(0.1 - 2.2)
Cambodia (2021/22)	353	19.0	(15.0 - 23.9)	0.0	(0.0 0.0)	8.0	(5.3 - 11.5)	3.5	(1.9 - 6.0)	6.9	(4.4 - 10.1)	0.6	(0.1 - 2.2)
Indonesia (1991)	624	4.2	(2.9 - 6.2)	0.6	(0.2 - 1.5)	2.2	(1.2 - 3.6)	0.0	(0.0 - 0.0)	1.2	(0.5 - 2.4)	0.2	(0.0 - 0.9)
Indonesia (1994)	1,048	4.6	(3.5 - 6.0)	0.3	(0.1 - 0.8)	3.8	(2.8 - 5.0)	0.3	(0.1 - 0.7)	0.1	(0.0 - 0.5)	0.1	(0.0 - 0.5)
Indonesia (1997)	1,204	2.8	(2.0 - 3.9)	0.1	(0.0 - 0.5)	2.0	(1.3 - 2.9)	0.5	(0.2 - 1.0)	0.2	(0.1 - 0.7)	0.0	(0.0 - 0.0)
Indonesia (2002/3)	1,211	3.0	(2.1 - 4.2)	0.1	(0.0 - 0.5)	2.2	(1.4 - 3.2)	0.1	(0.0 - 0.6)	0.5	(0.2 - 1.1)	0.2	(0.0 - 0.7)
Indonesia (2007)	852	5.6	(4.1 - 7.7)	0.1	(0.0 - 0.7)	3.2	(2.0 - 4.8)	1.4	(0.7 - 2.6)	0.4	(0.1 - 1.2)	0.5	(0.2 - 1.4)
Indonesia (2012)	1,256	8.3	(6.7 - 10.2)	0.2	(0.1 - 0.7)	4.8	(3.6 - 6.3)	0.5	(0.2 - 1.1)	1.4	(0.7 - 2.3)	1.4	(0.8 - 2.2)
Indonesia (2017)	1,791	6.9	(5.7 - 8.3)	0.2	(0.0 - 0.5)	5.4	(4.3 - 6.6)	0.3	(0.1 - 0.8)	0.9	(0.5 - 1.5)	0.1	(0.0 - 0.4)
Nepal (2011)	108	15.1	(8.6 - 25.7)	0.0	(0.0 0.0)	6.7	(2.6 - 13.6)	1.0	(0.1 - 5.6)	7.3	(2.6 - 15.2)	0.0	(0.0 0.0)
Nepal (2016)	344	8.1	(5.4 - 12.1)	0.4	(0.1 - 1.7)	6.7	(4.1 - 10.1)	0.5	(0.0 - 2.4)	0.6	(0.1 - 2.0)	0.0	(0.0 - 0.0)
Nepal (2022)	827	10.6	(8.5 - 13.2)	0.0	(0.0 0.0)	8.4	(6.5 - 10.7)	0.2	(0.0 - 0.9)	1.8	(1.0 - 3.0)	0.2	(0.0 - 0.9)
Philippines (2022)	632	10.2	(7.9 - 13.1)	0.7	(0.2 - 1.7)	7.1	(5.1 - 9.4)	0.9	(0.3 - 2.0)	0.6	(0.2 - 1.6)	1.0	(0.4 - 2.2)
**Latin America & Caribbean**													
Colombia (2005)	103	16.8	(9.9 - 27.8)	6.9	(2.5 - 14.3)	5.6	(1.7 - 13.0)	2.2	(0.4 - 7.5)	2.1	(0.3 - 7.6)	0.0	(0.0 0.0)
Colombia (2010)	1,141	6.8	(5.5 - 8.6)	0.2	(0.0 - 0.7)	5.3	(4.0 - 6.7)	0.3	(0.1 - 0.8)	0.7	(0.3 - 1.4)	0.4	(0.2 - 0.9)
Colombia (2015/16)	2,158	8.0	(6.8 - 9.4)	0.5	(0.3 - 1.0)	5.9	(4.8 - 7.1)	0.3	(0.1 - 0.7)	0.9	(0.5 - 1.5)	0.4	(0.1 - 0.8)
Dominican Republic (	107	27.2	(20.2 - 36.1)	0.0	(0.0 0.0)	24.4	(17.2 - 32.3)	2.1	(0.5 - 5.9)	0.5	(0.0 - 3.5)	0.2	(0.0 - 3.8)
Guatemala (2014/15)	358	12.6	(9.5 - 16.7)	0.6	(0.2 - 1.9)	8.1	(5.4 - 11.4)	0.5	(0.1 - 1.7)	3.4	(1.9 - 5.8)	0.0	(0.0 - 0.0)

CI= Confidence Interval

S8.6 Table: 12-month overall estimated probabilities of discontinuation and reason-specific estimated cumulative incidence of discontinuation per 100 episodes of use

Periodic abstinence

	No. of episodes	All reasons		Became pregnant while using		Side effect/health concerns		Other method-related		Wanted pregnancy/no need		Other/not stated	
		Rate	95%CI	Rate	95%CI	Rate	95%CI	Rate	95%CI	Rate	95%CI	Rate	95%CI
**Sub-Saharan Africa**													
Benin (2017/18)	463	30.3	(26.1 - 34.9)	3.6	(2.1 - 5.7)	0.0	(0.0 0.0)	6.4	(4.3 - 8.9)	17.6	(14.1 - 21.4)	2.7	(1.5 - 4.5)
Burkina Faso (2010)	202	19.0	(13.9 - 25.6)	10.9	(6.8 - 16.0)	0.0	(0.0 0.0)	2.7	(1.0 - 5.8)	4.0	(1.8 - 7.6)	1.5	(0.3 - 4.3)
Burkina Faso (2021)	540	23.3	(19.7 - 27.5)	2.2	(1.1 - 4.0)	0.0	(0.0 - 1.3)	4.7	(3.0 - 6.8)	12.7	(9.9 - 16.0)	3.7	(2.2 - 5.6)
Burundi (2010/11)	173	38.5	(30.8 - 47.5)	17.3	(11.3 - 24.4)	0.0	(0.0 0.0)	3.5	(1.3 - 7.7)	13.9	(8.7 - 20.2)	3.8	(1.5 - 8.0)
Comoros (2012)	141	16.8	(10.7 - 25.8)	7.9	(3.6 - 14.2)	0.4	(0.0 - 4.6)	0.0	(0.0 0.0)	3.7	(1.1 - 8.9)	4.7	(1.7 - 10.2)
Côte d'Ivoire (2021)	488	26.1	(22.0 - 30.7)	2.5	(1.3 - 4.4)	0.3	(0.0 - 1.5)	2.2	(1.0 - 4.0)	16.4	(12.9 - 20.2)	4.8	(3.0 - 7.1)
Ethiopia (2005)	191	44.0	(35.0 - 54.1)	5.7	(2.3 - 11.2)	1.1	(0.1 - 4.8)	11.1	(6.1 - 17.9)	23.4	(15.7 - 31.9)	2.7	(0.7 - 7.2)
Ethiopia (2016)	110	21.1	(13.8 - 31.6)	3.6	(1.0 - 9.2)	0.0	(0.0 0.0)	5.6	(2.1 - 11.6)	11.9	(6.0 - 20.0)	0.0	(0.0 0.0)
Gabon (2019/21)	411	32.6	(27.7 - 38.1)	1.7	(0.6 - 3.6)	0.0	(0.0 0.0)	6.1	(3.9 - 9.1)	19.9	(15.7 - 24.5)	4.9	(2.9 - 7.6)
Ghana (2014)	301	21.0	(17.0 - 25.9)	11.8	(8.6 - 15.6)	0.0	(0.0 0.0)	0.8	(0.2 - 2.4)	8.0	(5.4 - 11.2)	0.5	(0.1 - 1.9)
Ghana (2022/23)	923	30.7	(27.9 - 33.7)	7.0	(5.5 - 8.7)	0.5	(0.2 - 1.1)	6.0	(4.7 - 7.6)	14.3	(12.2 - 16.6)	2.8	(2.0 - 4.0)
Kenya (1998)	670	33.9	(30.4 - 37.6)	14.0	(11.5 - 16.7)	0.0	(0.0 0.0)	4.9	(3.5 - 6.8)	10.4	(8.2 - 12.8)	4.6	(3.2 - 6.4)
Kenya (2003)	656	34.2	(30.6 - 38.0)	15.6	(12.9 - 18.5)	0.0	(0.0 0.0)	3.2	(2.0 - 4.7)	9.6	(7.5 - 12.0)	5.8	(4.2 - 7.8)
Kenya (2014)	613	25.2	(21.9 - 29.0)	9.2	(7.0 - 11.8)	0.0	(0.0 0.0)	6.6	(4.7 - 8.8)	8.0	(6.0 - 10.4)	1.5	(0.7 - 2.7)
Kenya (2022)	586	33.1	(29.4 - 37.2)	5.6	(3.9 - 7.7)	0.9	(0.4 - 2.0)	8.7	(6.6 - 11.2)	15.1	(12.3 - 18.2)	2.8	(1.6 - 4.4)
Madagascar (2021)	1,247	26.3	(23.8 - 29.0)	8.9	(7.3 - 10.7)	0.0	(0.0 0.0)	6.2	(4.9 - 7.7)	10.2	(8.5 - 12.1)	1.0	(0.5 - 1.7)
Nigeria (2013)	868	22.4	(19.7 - 25.5)	6.4	(4.9 - 8.3)	0.0	(0.0 0.0)	1.3	(0.7 - 2.3)	11.2	(9.1 - 13.5)	3.5	(2.4 - 4.9)
Nigeria (2018)	781	34.7	(31.3 - 38.5)	9.4	(7.4 - 11.7)	0.1	(0.0 - 0.8)	3.4	(2.3 - 5.0)	20.7	(17.8 - 23.9)	1.1	(0.5 - 2.1)
Rwanda (2010/11)	298	26.9	(21.7 - 33.0)	13.9	(9.9 - 18.6)	0.0	(0.0 0.0)	5.8	(3.3 - 9.2)	4.3	(2.3 - 7.5)	2.9	(1.3 - 5.5)
Rwanda (2014/15)	243	25.7	(20.3 - 32.2)	13.0	(8.9 - 17.9)	0.0	(0.0 0.0)	6.1	(3.4 - 9.9)	4.7	(2.4 - 8.2)	1.9	(0.6 - 4.6)
Rwanda (2019/20)	354	28.0	(23.3 - 33.4)	10.9	(7.7 - 14.7)	0.4	(0.1 - 1.9)	6.9	(4.5 - 10.1)	7.6	(5.0 - 10.9)	2.2	(1.0 - 4.3)
Tanzania (2004/5)	328	31.6	(26.7 - 37.2)	6.0	(3.7 - 9.0)	0.0	(0.0 - 0.0)	11.2	(7.9 - 15.0)	12.4	(9.0 - 16.4)	2.1	(0.9 - 4.2)
Tanzania (2015/16)	529	20.6	(17.5 - 24.2)	8.2	(6.2 - 10.7)	0.2	(0.0 - 1.1)	4.2	(2.8 - 6.1)	6.3	(4.5 - 8.5)	1.6	(0.8 - 2.9)
Tanzania (2022)	615	27.9	(24.6 - 31.6)	3.9	(2.6 - 5.7)	1.1	(0.5 - 2.2)	3.8	(2.5 - 5.5)	13.8	(11.3 - 16.7)	5.2	(3.7 - 7.2)
Uganda (2011)	144	25.0	(18.2 - 33.9)	10.7	(6.0 - 17.0)	0.4	(0.0 - 3.6)	2.2	(0.6 - 6.0)	9.4	(5.0 - 15.5)	2.4	(0.6 - 6.4)
Zambia (2013/14)	120	30.4	(23.2 - 39.2)	15.0	(9.4 - 21.8)	0.0	(0.0 - 0.0)	9.2	(5.1 - 14.8)	1.5	(0.3 - 4.9)	4.8	(2.0 - 9.4)
**North Africa Western Asia and Europe**													
Armenia (2000)	259	40.9	(35.1 - 47.3)	23.2	(18.2 - 28.6)	0.1	(0.0 - 2.1)	9.5	(6.3 - 13.5)	6.5	(3.9 - 9.9)	1.6	(0.6 - 3.8)
Armenia (2005)	140	42.4	(35.0 - 50.7)	30.0	(23.0 - 37.3)	0.0	(0.0 0.0)	3.7	(1.5 - 7.5)	7.4	(3.9 - 12.3)	1.4	(0.3 - 4.4)
Armenia (2010)	104	28.0	(19.8 - 38.5)	13.0	(7.0 - 20.9)	0.0	(0.0 0.0)	3.0	(0.7 - 8.1)	10.2	(5.1 - 17.3)	1.8	(0.3 - 6.4)
Armenia (2015/16)	117	27.6	(20.2 - 37.0)	12.7	(7.4 - 19.6)	0.0	(0.0 0.0)	2.5	(0.7 - 6.7)	11.4	(6.3 - 18.3)	0.9	(0.1 - 4.3)
Azerbaijan (2006)	178	24.9	(19.3 - 31.7)	14.4	(9.9 - 19.8)	0.0	(0.0 0.0)	1.4	(0.3 - 3.9)	6.2	(3.3 - 10.3)	2.9	(1.1 - 6.0)
Egypt (1992/93)	131	40.9	(32.7 - 50.2)	20.3	(13.7 - 27.9)	2.1	(0.5 - 5.9)	9.2	(4.9 - 15.2)	7.8	(3.9 - 13.5)	1.4	(0.2 - 5.1)

Egypt (1995/96)	161	47.1	(38.7 - 56.3)	15.9	(10.1 - 22.9)	0.0	(0.0 0.0)	11.9	(6.9 - 18.2)	11.1	(6.3 - 17.5)	8.1	(4.2 - 13.7)
Egypt (2005)	117	31.0	(23.8 - 39.7)	17.6	(11.7 - 24.6)	0.0	(0.0 0.0)	6.8	(3.4 - 12.0)	4.4	(1.8 - 8.9)	2.1	(0.6 - 5.8)
Jordan (1990)	676	56.5	(52.6 - 60.4)	29.2	(25.8 - 32.8)	2.2	(1.3 - 3.6)	5.0	(3.5 - 6.8)	12.3	(9.9 - 15.0)	7.8	(5.9 - 10.0)
Jordan (1997)	841	63.9	(60.6 - 67.3)	30.6	(27.4 - 33.8)	0.9	(0.4 - 1.7)	13.9	(11.6 - 16.3)	16.3	(13.9 - 19.0)	2.3	(1.4 - 3.5)
Jordan (2002)	620	53.4	(49.4 - 57.6)	28.3	(24.7 - 32.1)	0.2	(0.0 - 1.0)	6.6	(4.7 - 8.8)	17.7	(14.7 - 20.9)	0.6	(0.2 - 1.6)
Jordan (2007)	771	39.8	(36.2 - 43.6)	21.1	(18.1 - 24.3)	0.1	(0.0 - 0.8)	6.3	(4.7 - 8.3)	11.7	(9.4 - 14.3)	0.5	(0.2 - 1.3)
Jordan (2009)	712	46.6	(43.0 - 50.3)	20.3	(17.4 - 23.3)	1.3	(0.7 - 2.4)	10.9	(8.8 - 13.3)	13.7	(11.3 - 16.3)	0.4	(0.1 - 1.1)
Jordan (2012)	555	45.6	(41.6 - 49.7)	20.4	(17.3 - 23.8)	1.5	(0.7 - 2.9)	7.3	(5.4 - 9.6)	16.1	(13.3 - 19.2)	0.1	(0.0 - 0.9)
Jordan (2017/18)	153	33.6	(26.9 - 41.5)	6.3	(3.2 - 10.8)	5.3	(2.6 - 9.5)	8.1	(4.6 - 12.8)	12.4	(7.8 - 18.0)	1.5	(0.4 - 4.4)
Jordan (2023)	129	17.8	(12.3 - 25.3)	0.9	(0.1 - 3.8)	2.6	(0.8 - 6.3)	5.9	(2.7 - 10.7)	8.1	(4.3 - 13.6)	0.3	(0.0 - 3.1)
Moldova (2005)	244	36.0	(29.6 - 43.3)	11.9	(7.7 - 16.9)	0.5	(0.0 - 2.6)	10.7	(6.9 - 15.5)	9.3	(5.7 - 14.0)	3.5	(1.6 - 6.9)
Morocco (1992)	368	52.2	(46.9 - 57.7)	25.1	(20.6 - 29.9)	0.6	(0.1 - 2.0)	8.6	(5.9 - 11.9)	11.6	(8.4 - 15.3)	6.3	(4.0 - 9.2)
Morocco (2003/4)	597	51.5	(47.5 - 55.6)	18.8	(15.8 - 22.1)	1.2	(0.5 - 2.3)	13.4	(10.8 - 16.3)	10.7	(8.4 - 13.4)	7.3	(5.4 - 9.6)
Türkiye (1993)	123	63.4	(54.7 - 72.1)	24.4	(17.2 - 32.4)	0.8	(0.1 - 4.2)	10.4	(5.8 - 16.6)	17.0	(10.8 - 24.3)	10.8	(6.1 - 17.1)
Türkiye (2003/4)	139	53.4	(44.8 - 62.6)	17.2	(11.2 - 24.3)	1.8	(0.3 - 6.1)	13.0	(7.8 - 19.6)	15.3	(9.6 - 22.2)	6.2	(2.9 - 11.2)
Ukraine (2007)	405	29.8	(25.3 - 34.9)	7.9	(5.4 - 11.0)	0.4	(0.0 - 1.6)	10.9	(7.9 - 14.4)	8.1	(5.5 - 11.2)	2.6	(1.3 - 4.6)
Yemen (2013)	429	33.3	(28.8 - 38.2)	16.1	(12.6 - 19.9)	0.5	(0.1 - 1.7)	7.0	(4.8 - 9.8)	6.4	(4.3 - 9.2)	3.3	(1.8 - 5.4)
**Central, South & Southeast Asia**													
Bangladesh (1993/94)	802	45.8	(42.1 - 49.6)	8.3	(6.4 - 10.5)	2.3	(1.4 - 3.6)	8.0	(6.2 - 10.2)	15.4	(12.8 - 18.2)	11.7	(9.5 - 14.2)
Bangladesh (1996/97)	595	43.5	(39.4 - 47.8)	10.6	(8.1 - 13.3)	0.1	(0.0 - 0.8)	9.2	(7.0 - 11.9)	12.9	(10.3 - 15.9)	10.7	(8.3 - 13.4)
Bangladesh (1999/0)	816	43.3	(39.7 - 47.2)	8.4	(6.4 - 10.6)	2.1	(1.2 - 3.3)	12.4	(10.1 - 14.9)	10.1	(7.9 - 12.4)	10.5	(8.4 - 12.9)
Bangladesh (2004)	967	43.2	(39.9 - 46.7)	10.4	(8.4 - 12.6)	0.1	(0.0 - 0.6)	13.0	(10.8 - 15.3)	10.2	(8.3 - 12.3)	9.6	(7.8 - 11.6)
Bangladesh (2011)	1,101	24.5	(21.8 - 27.4)	5.3	(4.0 - 6.9)	0.8	(0.4 - 1.6)	9.3	(7.5 - 11.2)	7.4	(5.9 - 9.3)	1.7	(1.0 - 2.7)
Bangladesh (2014)	735	18.4	(15.6 - 21.6)	3.9	(2.6 - 5.5)	0.2	(0.0 - 0.9)	7.9	(6.0 - 10.2)	4.7	(3.2 - 6.5)	1.8	(1.0 - 3.0)
Bangladesh (2017/18)	1,269	29.3	(26.6 - 32.1)	4.8	(3.6 - 6.2)	1.0	(0.5 - 1.8)	13.1	(11.2 - 15.2)	8.6	(7.0 - 10.3)	1.8	(1.1 - 2.7)
Bangladesh (2022)	910	23.2	(20.4 - 26.3)	3.6	(2.5 - 5.1)	0.8	(0.4 - 1.7)	6.4	(4.8 - 8.2)	11.5	(9.4 - 13.8)	0.8	(0.4 - 1.6)
Cambodia (2010/11)	407	12.7	(9.6 - 16.8)	4.0	(2.3 - 6.4)	0.1	(0.0 - 1.8)	4.0	(2.3 - 6.5)	2.8	(1.4 - 4.9)	1.9	(0.8 - 3.8)
Cambodia (2014)	360	27.3	(22.7 - 32.6)	10.9	(7.8 - 14.7)	1.0	(0.3 - 2.5)	3.4	(1.8 - 5.8)	6.8	(4.4 - 10.0)	5.2	(3.1 - 8.0)
Cambodia (2021/22)	233	52.7	(45.7 - 60.2)	6.5	(3.4 - 10.9)	0.1	(0.0 - 2.4)	29.1	(22.9 - 35.5)	7.1	(3.9 - 11.5)	10.0	(6.2 - 14.7)
India (2005/6)	5,228	33.1	(31.9 - 34.4)	7.9	(7.2 - 8.6)	0.4	(0.3 - 0.6)	5.0	(4.5 - 5.6)	17.0	(16.0 - 18.0)	2.8	(2.4 - 3.3)
India (2015/16)	22,081	44.4	(43.7 - 45.1)	5.0	(4.7 - 5.3)	1.4	(1.2 - 1.5)	9.7	(9.3 - 10.2)	20.6	(20.0 - 21.2)	7.7	(7.4 - 8.1)
India (2019/21)	51,365	60.4	(60.0 - 60.9)	4.8	(4.6 - 5.0)	1.8	(1.6 - 1.9)	10.5	(10.3 - 10.8)	27.2	(26.8 - 27.6)	16.1	(15.7 - 16.4)
Indonesia (1991)	396	40.3	(35.0 - 46.1)	15.1	(11.3 - 19.4)	0.3	(0.0 - 1.6)	9.8	(6.8 - 13.4)	6.4	(4.0 - 9.5)	8.8	(5.9 - 12.3)
Indonesia (1994)	466	34.0	(29.3 - 39.3)	12.1	(9.0 - 15.8)	0.9	(0.3 - 2.4)	7.5	(5.1 - 10.5)	7.2	(4.8 - 10.2)	6.2	(4.0 - 9.1)
Indonesia (1997)	394	28.2	(23.3 - 33.8)	11.3	(8.0 - 15.3)	0.1	(0.0 - 1.7)	6.7	(4.2 - 10.0)	9.5	(6.4 - 13.2)	0.6	(0.1 - 2.1)
Indonesia (2002/3)	474	16.8	(13.3 - 21.3)	3.8	(2.2 - 6.2)	0.4	(0.0 - 1.6)	5.7	(3.6 - 8.6)	5.3	(3.3 - 8.1)	1.6	(0.6 - 3.4)
Indonesia (2007)	503	24.9	(20.9 - 29.5)	5.4	(3.5 - 8.0)	2.0	(0.9 - 3.7)	5.4	(3.5 - 8.0)	10.0	(7.3 - 13.2)	2.1	(1.0 - 3.8)
Indonesia (2012)	514	19.8	(16.2 - 24.2)	4.4	(2.7 - 6.8)	1.3	(0.5 - 2.9)	3.0	(1.7 - 5.1)	4.7	(2.9 - 7.1)	6.4	(4.2 - 9.1)
Indonesia (2017)	759	27.4	(24.2 - 31.0)	5.9	(4.3 - 7.9)	1.0	(0.5 - 2.0)	4.5	(3.1 - 6.3)	14.0	(11.5 - 16.7)	1.9	(1.1 - 3.2)
Kazakhstan (1999)	294	51.7	(45.8 - 57.9)	22.7	(17.9 - 27.8)	0.0	(0.0 - 0.0)	21.2	(16.5 - 26.3)	6.0	(3.6 - 9.2)	1.8	(0.7 - 4.0)
Maldives (2009)	211	23.7	(18.3 - 30.4)	4.3	(2.1 - 7.7)	0.0	(0.0 0.0)	1.7	(0.5 - 4.4)	11.0	(7.0 - 15.9)	6.7	(3.7 - 10.8)

Nepal (2011)	119	34.4	(25.7 - 45.0)	1.0	(0.1 - 4.6)	0.0	(0.0 0.0)	7.1	(2.9 - 13.7)	24.0	(16.0 - 32.9)	2.3	(0.5 - 7.1)
Nepal (2016)	164	57.8	(50.8 - 65.0)	13.3	(8.9 - 18.5)	0.0	(0.0 - 0.0)	5.3	(2.8 - 9.1)	39.0	(32.0 - 45.9)	0.3	(0.0 - 2.3)
Nepal (2022)	188	28.2	(22.3 - 35.3)	2.8	(1.1 - 6.0)	0.0	(0.0 0.0)	3.6	(1.6 - 7.0)	20.7	(15.2 - 26.8)	1.0	(0.2 - 3.3)
Pakistan (2012/13)	123	35.9	(28.5 - 44.7)	4.5	(1.9 - 9.0)	0.0	(0.0 0.0)	1.1	(0.2 - 4.1)	18.4	(12.4 - 25.4)	11.9	(7.1 - 18.0)
Pakistan (2017/18)	138	34.9	(27.3 - 44.0)	3.0	(1.0 - 6.9)	0.0	(0.0 0.0)	2.1	(0.6 - 5.7)	29.8	(22.0 - 37.9)	0.0	(0.0 0.0)
Philippines (1993)	1,011	32.2	(29.1 - 35.5)	15.7	(13.3 - 18.3)	1.2	(0.6 - 2.1)	3.1	(2.1 - 4.4)	6.4	(4.9 - 8.2)	5.7	(4.3 - 7.5)
Philippines (1998)	1,060	36.7	(33.8 - 39.9)	18.6	(16.2 - 21.2)	1.2	(0.7 - 2.1)	5.6	(4.2 - 7.1)	7.0	(5.5 - 8.8)	4.2	(3.1 - 5.6)
Philippines (2003)	819	32.8	(29.4 - 36.4)	12.6	(10.3 - 15.2)	1.7	(1.0 - 2.9)	6.1	(4.5 - 8.1)	6.0	(4.4 - 7.9)	6.3	(4.7 - 8.2)
Philippines (2022)	387	16.7	(13.1 - 21.1)	3.9	(2.2 - 6.3)	1.2	(0.4 - 2.9)	1.6	(0.6 - 3.4)	8.9	(6.2 - 12.3)	1.1	(0.4 - 2.6)
Vietnam (1997)	358	29.6	(24.9 - 35.0)	12.8	(9.4 - 16.7)	0.2	(0.0 - 1.4)	7.9	(5.3 - 11.2)	6.8	(4.4 - 9.9)	1.9	(0.8 - 3.9)
Vietnam (2002)	411	33.9	(29.4 - 38.8)	15.4	(12.1 - 19.1)	0.3	(0.0 - 1.3)	11.4	(8.5 - 14.7)	6.1	(4.0 - 8.7)	0.7	(0.2 - 2.1)
**Latin America & Caribbean**													
Bolivia (1994)	2,181	40.7	(38.6 - 43.0)	20.0	(18.2 - 21.8)	1.2	(0.8 - 1.8)	6.0	(5.0 - 7.1)	9.8	(8.6 - 11.2)	3.7	(2.9 - 4.6)
Brazil (1996)	575	58.0	(53.9 - 62.1)	17.2	(14.2 - 20.5)	1.5	(0.7 - 2.7)	18.2	(15.2 - 21.5)	5.6	(3.9 - 7.7)	15.5	(12.7 - 18.5)
Colombia (1990)	692	47.2	(43.1 - 51.4)	18.0	(14.9 - 21.3)	0.4	(0.1 - 1.3)	12.9	(10.3 - 15.8)	12.0	(9.5 - 14.8)	3.9	(2.5 - 5.6)
Colombia (1995)	1,163	55.6	(52.7 - 58.5)	18.2	(16.0 - 20.5)	0.6	(0.3 - 1.3)	20.4	(18.1 - 22.8)	12.6	(10.7 - 14.6)	3.8	(2.8 - 5.0)
Colombia (2000)	1,290	59.2	(56.4 - 62.0)	19.5	(17.3 - 21.8)	0.6	(0.3 - 1.2)	22.0	(19.7 - 24.3)	14.5	(12.6 - 16.5)	2.6	(1.8 - 3.6)
Colombia (2005)	2,113	48.1	(46.0 - 50.3)	17.9	(16.3 - 19.6)	0.2	(0.1 - 0.4)	18.0	(16.4 - 19.7)	11.5	(10.1 - 12.9)	0.5	(0.3 - 0.9)
Colombia (2010)	1,415	51.4	(48.7 - 54.2)	16.4	(14.5 - 18.5)	1.0	(0.6 - 1.7)	16.3	(14.3 - 18.3)	16.0	(14.1 - 18.1)	1.7	(1.1 - 2.5)
Colombia (2015/16)	590	38.2	(34.3 - 42.3)	14.6	(11.9 - 17.7)	2.2	(1.2 - 3.7)	9.2	(7.0 - 11.7)	11.1	(8.7 - 13.8)	1.1	(0.5 - 2.2)
Dominican Republic (	324	64.2	(59.3 - 69.2)	21.9	(17.9 - 26.3)	0.1	(0.0 - 1.2)	13.7	(10.5 - 17.4)	18.3	(14.5 - 22.4)	10.2	(7.3 - 13.5)
Dominican Republic (	383	70.2	(65.8 - 74.4)	25.0	(21.1 - 29.1)	0.0	(0.0 0.0)	14.8	(11.7 - 18.3)	20.1	(16.5 - 23.9)	10.3	(7.7 - 13.3)
Dominican Republic (	656	56.9	(53.4 - 60.4)	19.4	(16.8 - 22.3)	0.7	(0.3 - 1.4)	15.4	(13.0 - 18.0)	16.4	(13.9 - 19.1)	4.9	(3.6 - 6.6)
Guatemala (1995)	402	37.6	(33.1 - 42.4)	14.4	(11.2 - 18.0)	0.6	(0.1 - 1.8)	3.6	(2.1 - 5.7)	13.4	(10.4 - 16.9)	5.6	(3.7 - 8.0)
Guatemala (1998/99)	298	37.5	(32.5 - 43.0)	19.1	(15.1 - 23.5)	1.3	(0.5 - 3.0)	6.7	(4.4 - 9.7)	8.9	(6.1 - 12.2)	1.5	(0.5 - 3.3)
Guatemala (2014/15)	1,275	23.9	(21.4 - 26.6)	9.0	(7.3 - 10.8)	0.2	(0.1 - 0.8)	3.2	(2.2 - 4.3)	10.1	(8.4 - 12.0)	1.4	(0.8 - 2.2)
Honduras (2011/12)	990	54.3	(51.1 - 57.6)	8.5	(6.8 - 10.4)	0.3	(0.1 - 0.9)	22.4	(19.8 - 25.2)	14.5	(12.3 - 16.9)	8.6	(6.9 - 10.5)
Nicaragua (1998)	265	47.3	(41.5 - 53.4)	10.3	(7.1 - 14.3)	0.8	(0.2 - 2.4)	11.3	(8.0 - 15.4)	13.8	(10.0 - 18.1)	11.0	(7.7 - 15.1)
Paraguay (1990)	499	52.2	(47.9 - 56.7)	18.4	(15.1 - 21.9)	0.3	(0.0 - 1.2)	14.7	(11.7 - 17.9)	9.0	(6.7 - 11.7)	9.9	(7.5 - 12.7)
Peru (1991/92)	4,036	49.5	(47.8 - 51.1)	26.5	(25.1 - 27.9)	0.2	(0.1 - 0.3)	8.1	(7.2 - 9.0)	10.9	(9.9 - 11.9)	3.9	(3.3 - 4.5)
Peru (1996)	6,832	46.8	(45.5 - 48.1)	23.6	(22.6 - 24.7)	0.5	(0.3 - 0.7)	9.5	(8.8 - 10.3)	9.4	(8.7 - 10.2)	3.7	(3.3 - 4.2)
Peru (2000)	4,655	39.5	(38.0 - 41.1)	17.6	(16.4 - 18.8)	0.3	(0.2 - 0.5)	10.5	(9.5 - 11.5)	9.3	(8.4 - 10.2)	1.9	(1.5 - 2.3)
Peru (2004/6)	3,919	42.9	(41.1 - 44.8)	16.1	(14.8 - 17.5)	0.2	(0.1 - 0.5)	12.4	(11.2 - 13.6)	11.5	(10.3 - 12.7)	2.7	(2.1 - 3.3)
Peru (2007/8)	4,451	44.8	(43.3 - 46.4)	15.6	(14.4 - 16.7)	0.2	(0.1 - 0.4)	13.6	(12.6 - 14.7)	11.4	(10.4 - 12.4)	4.0	(3.4 - 4.7)
Peru (2009)	285	99.6	(97.9 - #####	2.3	(0.9 - 4.8)	0.8	(0.1 - 2.6)	37.1	(31.0 - 43.1)	4.1	(2.1 - 7.1)	55.4	(48.9 - 61.4)
Peru (2010)	4,055	41.8	(40.2 - 43.5)	14.6	(13.5 - 15.9)	0.2	(0.1 - 0.4)	14.3	(13.1 - 15.5)	9.2	(8.3 - 10.2)	3.5	(2.9 - 4.2)
Peru (2011)	3,804	37.9	(36.3 - 39.7)	15.0	(13.8 - 16.3)	0.2	(0.1 - 0.4)	11.7	(10.6 - 12.9)	7.8	(6.9 - 8.8)	3.2	(2.6 - 3.9)
Peru (2012)	4,252	41.4	(39.8 - 43.1)	16.6	(15.4 - 17.9)	0.2	(0.1 - 0.4)	11.9	(10.8 - 13.0)	10.2	(9.2 - 11.2)	2.5	(2.0 - 3.0)

CI= Confidence Interval

S8.7 Table: 12-month overall estimated probabilities of discontinuation and reason-specific estimated cumulative incidence of discontinuation per 100 episodes of use

Withdrawal

	No. of episodes	All reasons		Became pregnant while using		Side effect/health concerns		Other method-related		Wanted pregnancy/no need		Other/not stated	
		Rate	95%CI	Rate	95%CI	Rate	95%CI	Rate	95%CI	Rate	95%CI	Rate	95%CI
**Sub-Saharan Africa**													
Angola (2015/16)	107	47.7	(37.2 - 59.4)	2.2	(0.4 - 7.4)	1.9	(0.3 - 6.6)	5.7	(2.0 - 12.1)	20.8	(12.8 - 30.2)	17.1	(9.6 - 26.4)
Benin (2017/18)	332	39.0	(33.5 - 45.1)	2.8	(1.3 - 5.1)	0.0	(0.0 0.0)	10.2	(7.0 - 14.2)	18.6	(14.2 - 23.4)	7.5	(4.8 - 10.9)
Burundi (2010/11)	195	42.4	(34.6 - 51.1)	19.2	(13.1 - 26.2)	0.0	(0.0 0.0)	4.7	(2.1 - 9.1)	9.8	(5.6 - 15.3)	8.7	(4.7 - 14.0)
Comoros (2012)	112	7.1	(3.2 - 15.3)	2.1	(0.4 - 7.2)	0.0	(0.0 - 0.0)	0.9	(0.0 - 5.4)	3.3	(0.8 - 8.8)	0.9	(0.0 - 5.4)
Gabon (2019/21)	437	54.9	(50.1 - 59.9)	3.6	(2.2 - 5.6)	0.0	(0.0 0.0)	13.4	(10.3 - 17.0)	28.1	(23.9 - 32.5)	9.8	(7.2 - 12.8)
Ghana (2014)	156	23.7	(17.7 - 31.4)	10.0	(5.8 - 15.4)	0.0	(0.0 0.0)	4.9	(2.4 - 9.0)	8.1	(4.4 - 13.3)	0.7	(0.1 - 3.6)
Ghana (2022/23)	457	43.6	(39.4 - 48.0)	6.9	(4.9 - 9.3)	0.0	(0.0 0.0)	9.7	(7.3 - 12.5)	22.4	(19.0 - 26.1)	4.6	(3.0 - 6.6)
Kenya (2014)	140	31.8	(23.3 - 42.4)	8.7	(4.1 - 15.5)	1.3	(0.2 - 5.4)	6.9	(3.0 - 13.0)	12.5	(6.7 - 20.4)	2.3	(0.5 - 7.0)
Kenya (2022)	211	39.7	(33.5 - 46.6)	2.8	(1.2 - 5.7)	0.4	(0.0 - 2.6)	20.1	(15.1 - 25.7)	12.7	(8.8 - 17.4)	3.6	(1.7 - 6.7)
Madagascar (2021)	234	43.4	(37.0 - 50.4)	9.4	(5.9 - 13.8)	0.0	(0.0 0.0)	9.8	(6.4 - 14.2)	21.4	(16.1 - 27.2)	2.8	(1.2 - 5.6)
Malawi (2004/5)	514	41.2	(36.6 - 46.0)	10.3	(7.6 - 13.4)	0.4	(0.1 - 1.4)	9.9	(7.3 - 12.9)	15.1	(11.9 - 18.6)	5.6	(3.7 - 8.0)
Malawi (2015/16)	282	57.8	(51.5 - 64.3)	4.9	(2.6 - 8.2)	0.4	(0.1 - 2.1)	22.6	(17.5 - 28.2)	19.6	(14.8 - 25.0)	10.2	(6.8 - 14.5)
Nigeria (2013)	1,216	29.0	(26.2 - 31.9)	7.0	(5.5 - 8.7)	0.0	(0.0 0.0)	3.1	(2.1 - 4.3)	14.5	(12.4 - 16.7)	4.4	(3.2 - 5.8)
Nigeria (2018)	1,371	40.2	(37.6 - 42.9)	10.5	(9.0 - 12.2)	0.1	(0.0 - 0.5)	5.4	(4.3 - 6.7)	21.2	(19.1 - 23.5)	2.9	(2.1 - 3.9)
Rwanda (2010/11)	334	30.5	(25.4 - 36.4)	14.6	(10.7 - 19.1)	0.0	(0.0 0.0)	8.5	(5.6 - 12.2)	4.9	(2.7 - 8.0)	2.5	(1.1 - 4.9)
Rwanda (2014/15)	282	32.0	(26.4 - 38.3)	13.9	(9.9 - 18.5)	0.0	(0.0 0.0)	8.7	(5.6 - 12.7)	6.5	(3.8 - 10.2)	2.9	(1.3 - 5.7)
Rwanda (2019/20)	421	43.0	(38.2 - 48.2)	14.2	(11.0 - 17.9)	0.2	(0.0 - 1.4)	15.3	(11.9 - 19.1)	10.4	(7.6 - 13.7)	2.8	(1.5 - 4.8)
Tanzania (2004/5)	482	42.0	(37.9 - 46.5)	10.8	(8.3 - 13.7)	0.2	(0.0 - 1.0)	11.1	(8.5 - 14.0)	15.7	(12.7 - 19.0)	4.3	(2.8 - 6.2)
Tanzania (2015/16)	335	32.4	(27.1 - 38.4)	10.0	(6.7 - 14.0)	0.2	(0.0 - 1.6)	18.4	(14.1 - 23.3)	3.7	(1.9 - 6.4)	0.1	(0.0 - 2.1)
Tanzania (2022)	411	33.7	(28.2 - 39.8)	4.1	(2.1 - 7.2)	1.4	(0.5 - 3.5)	11.0	(7.6 - 15.1)	12.0	(8.4 - 16.2)	5.2	(3.0 - 8.3)
Uganda (2011)	225	45.5	(38.8 - 52.7)	22.6	(17.1 - 28.7)	0.0	(0.0 0.0)	5.0	(2.6 - 8.6)	12.6	(8.5 - 17.6)	5.3	(2.7 - 9.0)
Zambia (2013/14)	820	30.8	(27.4 - 34.5)	7.7	(5.8 - 9.9)	0.0	(0.0 0.0)	10.7	(8.5 - 13.2)	6.4	(4.7 - 8.5)	6.0	(4.4 - 8.0)
Zambia (2018/19)	383	47.0	(41.5 - 52.8)	7.9	(5.2 - 11.2)	0.0	(0.0 0.0)	25.5	(20.8 - 30.4)	10.8	(7.6 - 14.6)	2.8	(1.4 - 5.1)
Zimbabwe (1994)	319	30.0	(25.1 - 35.6)	9.3	(6.3 - 13.0)	0.0	(0.0 0.0)	8.3	(5.5 - 11.8)	6.6	(4.2 - 9.9)	5.8	(3.5 - 8.7)
Zimbabwe (1999)	145	22.6	(16.4 - 30.5)	1.7	(0.4 - 4.9)	0.0	(0.0 0.0)	3.6	(1.4 - 7.6)	9.3	(5.1 - 14.9)	8.0	(4.2 - 13.2)
Zimbabwe (2005/6)	160	31.8	(24.1 - 41.1)	6.1	(2.7 - 11.7)	0.9	(0.1 - 4.4)	6.4	(2.9 - 12.0)	7.6	(3.7 - 13.3)	10.8	(6.1 - 16.8)
Zimbabwe (2010/11)	103	37.7	(28.9 - 48.3)	8.5	(4.0 - 15.3)	1.2	(0.2 - 4.9)	13.0	(7.4 - 20.3)	9.5	(4.6 - 16.4)	5.5	(2.1 - 11.3)
Zimbabwe (2015)	105	32.4	(24.2 - 42.6)	5.1	(1.9 - 10.8)	0.0	(0.0 - 0.0)	9.9	(5.2 - 16.4)	15.8	(9.4 - 23.7)	1.6	(0.3 - 5.6)
**North Africa Western Asia and Europe**													
Albania (2017/18)	1,508	14.4	(12.7 - 16.2)	1.3	(0.8 - 2.0)	0.2	(0.0 - 0.5)	1.0	(0.6 - 1.6)	10.1	(8.7 - 11.7)	1.8	(1.2 - 2.5)
Armenia (2000)	2,038	39.9	(37.6 - 42.2)	28.7	(26.6 - 30.8)	0.9	(0.5 - 1.4)	2.6	(2.0 - 3.5)	5.7	(4.7 - 6.8)	2.0	(1.4 - 2.7)

Armenia (2005)	1,078	35.1	(32.3 - 38.0)	21.7	(19.2 - 24.2)	0.2	(0.0 - 0.6)	1.7	(1.0 - 2.6)	9.7	(8.1 - 11.6)	1.8	(1.1 - 2.7)
Armenia (2010)	841	28.4	(25.3 - 31.8)	14.0	(11.6 - 16.6)	0.0	(0.0 0.0)	1.6	(0.9 - 2.7)	11.3	(9.2 - 13.7)	1.4	(0.8 - 2.5)
Armenia (2015/16)	1,040	31.4	(28.4 - 34.7)	9.6	(7.8 - 11.8)	0.0	(0.0 0.0)	3.4	(2.3 - 4.7)	17.3	(14.8 - 19.9)	1.2	(0.6 - 2.1)
Azerbaijan (2006)	1,795	30.9	(28.6 - 33.3)	19.8	(17.9 - 21.9)	0.0	(0.0 - 0.0)	1.0	(0.6 - 1.6)	5.6	(4.5 - 6.8)	4.4	(3.5 - 5.6)
Egypt (1995/96)	120	46.4	(36.7 - 57.3)	7.8	(3.4 - 14.6)	0.6	(0.0 - 4.3)	10.4	(5.3 - 17.3)	13.4	(7.3 - 21.3)	14.3	(8.1 - 22.2)
Jordan (1990)	557	55.3	(51.2 - 59.6)	19.9	(16.7 - 23.4)	2.6	(1.5 - 4.1)	8.4	(6.3 - 10.8)	9.5	(7.3 - 12.2)	14.9	(12.1 - 18.0)
Jordan (1997)	1,091	58.1	(54.9 - 61.2)	23.3	(20.7 - 26.0)	1.0	(0.5 - 1.8)	11.7	(9.8 - 13.8)	13.7	(11.6 - 15.9)	8.3	(6.7 - 10.2)
Jordan (2002)	1,286	50.3	(47.3 - 53.3)	16.5	(14.4 - 18.8)	0.7	(0.3 - 1.4)	12.0	(10.2 - 14.0)	18.0	(15.7 - 20.3)	3.1	(2.2 - 4.2)
Jordan (2007)	2,110	36.2	(34.0 - 38.5)	13.4	(11.8 - 15.0)	1.2	(0.8 - 1.8)	7.3	(6.2 - 8.5)	12.2	(10.8 - 13.8)	2.1	(1.5 - 2.9)
Jordan (2009)	2,660	41.4	(39.3 - 43.6)	13.0	(11.6 - 14.5)	1.5	(1.0 - 2.0)	10.0	(8.8 - 11.3)	14.7	(13.2 - 16.2)	2.3	(1.7 - 3.0)
Jordan (2012)	3,210	43.8	(42.0 - 45.7)	12.9	(11.6 - 14.2)	1.0	(0.6 - 1.4)	10.8	(9.6 - 11.9)	18.0	(16.5 - 19.4)	1.3	(0.9 - 1.8)
Jordan (2017/18)	2,512	34.8	(32.7 - 36.9)	5.2	(4.3 - 6.2)	1.0	(0.7 - 1.5)	6.7	(5.7 - 7.9)	19.9	(18.1 - 21.6)	2.0	(1.4 - 2.6)
Jordan (2023)	2,798	31.3	(29.5 - 33.2)	5.9	(5.0 - 6.9)	1.2	(0.9 - 1.7)	4.2	(3.4 - 5.0)	18.4	(17.0 - 20.0)	1.5	(1.1 - 2.1)
Moldova (2005)	1,258	38.2	(35.5 - 41.1)	13.0	(11.1 - 15.0)	0.6	(0.3 - 1.2)	9.9	(8.3 - 11.7)	9.2	(7.6 - 10.9)	5.6	(4.3 - 7.0)
Morocco (1992)	272	48.1	(42.0 - 54.6)	11.4	(7.8 - 15.7)	0.8	(0.2 - 2.7)	6.6	(3.9 - 10.2)	11.3	(7.7 - 15.6)	18.1	(13.6 - 23.0)
Morocco (2003/4)	670	49.4	(45.4 - 53.6)	8.0	(6.0 - 10.5)	1.2	(0.5 - 2.3)	18.6	(15.5 - 21.9)	16.6	(13.7 - 19.8)	5.0	(3.5 - 6.9)
Türkiye (1993)	2,321	41.1	(38.9 - 43.2)	15.4	(13.8 - 17.0)	0.2	(0.1 - 0.5)	10.1	(8.8 - 11.4)	9.2	(8.0 - 10.5)	6.3	(5.3 - 7.4)
Türkiye (1998)	2,020	40.1	(37.9 - 42.3)	12.2	(10.8 - 13.8)	0.4	(0.2 - 0.7)	8.4	(7.2 - 9.7)	9.2	(7.9 - 10.5)	9.9	(8.7 - 11.3)
Türkiye (2003/4)	2,931	42.7	(40.8 - 44.7)	13.5	(12.2 - 14.9)	0.5	(0.3 - 0.8)	12.4	(11.1 - 13.7)	11.5	(10.3 - 12.8)	4.9	(4.1 - 5.7)
Türkiye (2018/19)	1,207	32.1	(29.3 - 35.0)	9.9	(8.1 - 11.8)	0.4	(0.2 - 1.0)	4.9	(3.7 - 6.3)	14.0	(11.9 - 16.2)	2.9	(2.0 - 4.1)
Ukraine (2007)	646	36.0	(32.5 - 39.8)	7.9	(6.0 - 10.1)	1.6	(0.9 - 2.8)	10.0	(7.9 - 12.4)	6.0	(4.4 - 8.0)	10.5	(8.3 - 13.0)
Yemen (2013)	774	35.6	(31.8 - 39.7)	12.6	(10.0 - 15.4)	0.3	(0.0 - 1.1)	8.6	(6.5 - 11.0)	6.9	(5.0 - 9.2)	7.3	(5.3 - 9.6)
**Central, South & Southeast Asia**													
Bangladesh (1993/94)	407	56.6	(51.3 - 62.1)	9.1	(6.3 - 12.5)	5.1	(3.1 - 7.7)	7.0	(4.7 - 10.0)	17.3	(13.4 - 21.5)	18.2	(14.3 - 22.4)
Bangladesh (1996/97)	318	61.5	(55.8 - 67.2)	4.9	(2.8 - 7.9)	16.6	(12.7 - 21.0)	10.2	(7.0 - 14.0)	12.1	(8.6 - 16.3)	17.7	(13.5 - 22.2)
Bangladesh (1999/0)	646	53.8	(49.6 - 58.1)	10.0	(7.7 - 12.8)	5.2	(3.6 - 7.2)	9.0	(6.7 - 11.6)	13.3	(10.6 - 16.3)	16.3	(13.4 - 19.5)
Bangladesh (2004)	693	60.8	(56.9 - 64.8)	8.8	(6.7 - 11.2)	0.9	(0.3 - 1.8)	22.2	(19.0 - 25.5)	21.2	(18.1 - 24.6)	7.8	(5.9 - 10.1)
Bangladesh (2011)	350	27.0	(22.2 - 32.6)	8.9	(5.9 - 12.7)	1.1	(0.3 - 2.8)	10.4	(7.3 - 14.2)	4.5	(2.5 - 7.3)	2.1	(0.9 - 4.2)
Bangladesh (2014)	302	27.9	(22.5 - 34.3)	4.4	(2.2 - 7.8)	0.0	(0.0 0.0)	12.3	(8.5 - 16.9)	6.8	(4.0 - 10.6)	4.4	(2.3 - 7.5)
Bangladesh (2017/18)	584	32.2	(28.3 - 36.5)	6.8	(4.8 - 9.2)	1.7	(0.8 - 3.1)	8.6	(6.3 - 11.2)	11.1	(8.6 - 14.0)	4.1	(2.6 - 6.1)
Bangladesh (2022)	684	33.7	(29.9 - 37.8)	6.1	(4.3 - 8.3)	0.8	(0.3 - 1.8)	9.6	(7.4 - 12.3)	14.8	(12.0 - 17.8)	2.4	(1.4 - 3.9)
Cambodia (2010/11)	1,350	18.2	(16.1 - 20.4)	7.9	(6.5 - 9.4)	0.2	(0.1 - 0.7)	4.2	(3.2 - 5.5)	4.9	(3.8 - 6.1)	1.0	(0.5 - 1.6)
Cambodia (2014)	1,944	25.2	(23.3 - 27.2)	11.3	(9.9 - 12.8)	0.4	(0.2 - 0.8)	4.6	(3.7 - 5.6)	7.3	(6.2 - 8.6)	1.6	(1.1 - 2.2)
Cambodia (2021/22)	1,991	27.9	(26.0 - 30.0)	8.1	(7.0 - 9.4)	0.5	(0.3 - 0.8)	7.1	(6.0 - 8.3)	11.1	(9.7 - 12.5)	1.2	(0.8 - 1.7)
India (2005/6)	3,734	37.2	(35.4 - 39.1)	7.5	(6.5 - 8.6)	0.6	(0.4 - 1.0)	7.3	(6.4 - 8.3)	17.6	(16.1 - 19.1)	4.2	(3.5 - 5.0)
India (2015/16)	19,755	50.9	(50.1 - 51.7)	4.8	(4.5 - 5.1)	1.8	(1.6 - 2.1)	12.0	(11.5 - 12.5)	23.5	(22.8 - 24.2)	8.8	(8.3 - 9.2)
India (2019/21)	42,766	59.2	(58.6 - 59.7)	4.6	(4.4 - 4.8)	2.4	(2.2 - 2.6)	11.0	(10.7 - 11.3)	27.6	(27.1 - 28.1)	13.6	(13.2 - 14.0)
Indonesia (1991)	362	50.1	(44.0 - 56.7)	8.0	(5.0 - 11.9)	1.0	(0.3 - 2.9)	11.9	(8.2 - 16.3)	12.5	(8.7 - 17.0)	16.7	(12.4 - 21.6)

Indonesia (1994)	310	37.1	(31.6 - 43.2)	11.2	(7.8 - 15.4)	0.0	(0.0 0.0)	11.4	(8.0 - 15.6)	6.3	(3.8 - 9.7)	8.1	(5.3 - 11.7)
Indonesia (1997)	307	33.1	(27.5 - 39.6)	11.1	(7.4 - 15.5)	0.0	(0.0 - 0.0)	13.3	(9.3 - 18.0)	5.5	(3.1 - 8.8)	3.3	(1.6 - 6.0)
Indonesia (2002/3)	388	22.2	(18.0 - 27.2)	6.6	(4.2 - 9.8)	0.7	(0.2 - 2.1)	3.5	(1.9 - 6.0)	8.1	(5.4 - 11.4)	3.2	(1.7 - 5.6)
Indonesia (2007)	742	25.7	(22.5 - 29.4)	3.2	(2.0 - 4.8)	1.1	(0.5 - 2.2)	7.4	(5.5 - 9.7)	8.8	(6.7 - 11.2)	5.2	(3.7 - 7.1)
Indonesia (2012)	852	24.4	(21.4 - 27.7)	5.5	(4.0 - 7.3)	2.8	(1.8 - 4.2)	5.6	(4.1 - 7.5)	5.8	(4.2 - 7.7)	4.6	(3.3 - 6.4)
Indonesia (2017)	1,686	26.4	(24.2 - 28.8)	5.9	(4.8 - 7.2)	1.3	(0.8 - 2.0)	7.2	(6.0 - 8.6)	9.9	(8.4 - 11.5)	2.1	(1.5 - 2.9)
Kazakhstan (1999)	231	64.4	(58.6 - 70.1)	17.9	(13.6 - 22.7)	0.5	(0.1 - 2.2)	24.2	(19.3 - 29.4)	8.4	(5.5 - 12.1)	13.4	(9.7 - 17.7)
Kyrgyz Republic (201	162	31.8	(25.3 - 39.6)	6.9	(3.7 - 11.5)	0.7	(0.1 - 3.1)	9.2	(5.4 - 14.2)	9.8	(5.8 - 15.0)	5.3	(2.6 - 9.4)
Maldives (2009)	406	27.5	(22.9 - 32.9)	6.4	(4.0 - 9.5)	0.4	(0.0 - 1.6)	3.3	(1.7 - 5.7)	11.3	(8.1 - 15.1)	6.1	(3.8 - 9.1)
Nepal (2011)	903	55.1	(51.7 - 58.6)	7.0	(5.3 - 8.9)	0.1	(0.0 - 0.6)	3.9	(2.8 - 5.5)	43.0	(39.6 - 46.4)	1.0	(0.5 - 2.0)
Nepal (2016)	1,793	64.3	(61.9 - 66.8)	4.1	(3.1 - 5.2)	0.4	(0.1 - 0.8)	6.0	(4.9 - 7.3)	53.4	(50.9 - 55.9)	0.5	(0.2 - 1.0)
Nepal (2022)	2,075	50.6	(48.4 - 52.9)	5.2	(4.2 - 6.2)	0.1	(0.0 - 0.3)	5.2	(4.2 - 6.3)	39.6	(37.4 - 41.8)	0.6	(0.3 - 1.0)
Pakistan (2012/13)	1,370	33.3	(30.8 - 36.0)	8.8	(7.4 - 10.5)	1.0	(0.5 - 1.6)	3.3	(2.5 - 4.4)	16.3	(14.3 - 18.3)	3.9	(3.0 - 5.1)
Pakistan (2017/18)	1,109	25.0	(22.4 - 27.8)	6.1	(4.7 - 7.7)	0.4	(0.1 - 1.0)	2.5	(1.7 - 3.6)	14.5	(12.4 - 16.8)	1.5	(0.8 - 2.4)
Philippines (1993)	1,230	41.8	(38.9 - 44.8)	21.2	(18.8 - 23.7)	3.8	(2.8 - 5.0)	3.7	(2.7 - 4.9)	4.6	(3.5 - 6.0)	8.6	(7.0 - 10.3)
Philippines (1998)	1,292	46.0	(43.2 - 48.8)	22.4	(20.1 - 24.8)	4.8	(3.7 - 6.0)	6.2	(4.9 - 7.6)	6.6	(5.3 - 8.0)	6.0	(4.8 - 7.4)
Philippines (2003)	1,258	44.7	(41.8 - 47.6)	17.5	(15.4 - 19.8)	2.9	(2.1 - 4.0)	7.9	(6.4 - 9.6)	7.6	(6.1 - 9.2)	8.7	(7.2 - 10.4)
Philippines (2022)	2,344	33.1	(31.1 - 35.1)	8.3	(7.2 - 9.5)	3.5	(2.8 - 4.3)	8.1	(7.0 - 9.3)	11.0	(9.8 - 12.4)	2.1	(1.6 - 2.8)
Tajikistan (2012)	144	34.5	(27.4 - 42.9)	6.0	(2.9 - 10.7)	0.0	(0.0 0.0)	5.5	(2.6 - 10.1)	8.1	(4.4 - 13.1)	15.0	(9.7 - 21.3)
Tajikistan (2017)	124	27.4	(20.4 - 36.2)	4.3	(1.6 - 9.1)	0.0	(0.0 0.0)	7.2	(3.5 - 12.7)	9.6	(5.2 - 15.5)	6.4	(3.0 - 11.4)
Vietnam (1997)	670	18.8	(15.8 - 22.2)	7.5	(5.5 - 9.8)	0.1	(0.0 - 0.9)	8.3	(6.3 - 10.7)	1.7	(0.8 - 3.0)	1.2	(0.5 - 2.3)
Vietnam (2002)	868	31.5	(28.4 - 35.0)	14.6	(12.2 - 17.2)	0.0	(0.0 0.0)	10.7	(8.7 - 13.0)	5.4	(3.9 - 7.2)	0.9	(0.4 - 1.7)
**Latin America & Caribbean**													
Bolivia (1994)	258	58.2	(51.7 - 64.9)	15.2	(10.8 - 20.4)	1.8	(0.6 - 4.2)	13.9	(9.7 - 18.8)	10.2	(6.6 - 14.6)	17.2	(12.6 - 22.5)
Brazil (1996)	637	63.9	(60.1 - 67.6)	15.9	(13.2 - 18.8)	0.6	(0.2 - 1.5)	24.4	(21.2 - 27.7)	7.9	(6.0 - 10.2)	15.0	(12.4 - 17.8)
Colombia (1990)	362	43.4	(38.3 - 48.8)	16.8	(13.1 - 20.9)	0.0	(0.0 - 0.0)	10.3	(7.4 - 13.7)	9.9	(7.1 - 13.3)	6.4	(4.1 - 9.3)
Colombia (1995)	1,081	63.8	(60.8 - 66.9)	14.7	(12.6 - 17.0)	1.0	(0.5 - 1.8)	23.9	(21.3 - 26.6)	15.2	(13.1 - 17.5)	9.0	(7.3 - 10.8)
Colombia (2000)	1,425	65.0	(62.4 - 67.6)	17.1	(15.1 - 19.2)	0.2	(0.1 - 0.6)	24.5	(22.3 - 26.8)	18.5	(16.5 - 20.7)	4.7	(3.7 - 5.9)
Colombia (2005)	3,171	50.8	(48.9 - 52.6)	16.2	(14.9 - 17.6)	0.4	(0.2 - 0.7)	19.7	(18.3 - 21.2)	11.4	(10.3 - 12.5)	3.0	(2.4 - 3.6)
Colombia (2010)	2,710	54.1	(52.2 - 56.0)	11.5	(10.3 - 12.7)	0.4	(0.2 - 0.7)	20.2	(18.6 - 21.7)	18.5	(17.1 - 20.1)	3.5	(2.8 - 4.3)
Colombia (2015/16)	1,315	45.4	(42.5 - 48.5)	11.1	(9.3 - 13.0)	0.3	(0.1 - 0.8)	16.1	(14.0 - 18.3)	15.7	(13.6 - 17.9)	2.3	(1.5 - 3.3)
Dominican Republic (	361	76.8	(72.1 - 81.1)	22.2	(18.0 - 26.7)	1.4	(0.5 - 3.1)	18.1	(14.3 - 22.2)	16.7	(13.1 - 20.8)	18.3	(14.6 - 22.5)
Dominican Republic (	446	71.2	(67.0 - 75.3)	16.3	(13.1 - 19.8)	0.0	(0.0 0.0)	18.5	(15.2 - 22.1)	16.5	(13.3 - 20.0)	19.9	(16.4 - 23.6)
Dominican Republic (	931	66.6	(63.5 - 69.7)	12.9	(10.8 - 15.2)	0.5	(0.2 - 1.2)	25.8	(23.0 - 28.6)	16.1	(13.8 - 18.5)	11.3	(9.4 - 13.5)
Guatemala (2014/15)	1,372	25.8	(23.3 - 28.5)	9.1	(7.5 - 10.9)	0.1	(0.0 - 0.6)	4.7	(3.6 - 6.1)	10.2	(8.5 - 12.1)	1.7	(1.0 - 2.6)
Honduras (2011/12)	2,683	63.1	(61.1 - 65.0)	7.6	(6.6 - 8.7)	0.9	(0.6 - 1.3)	22.6	(21.0 - 24.3)	18.5	(17.0 - 20.0)	13.5	(12.2 - 14.9)
Nicaragua (1998)	160	63.9	(56.7 - 71.1)	7.0	(3.8 - 11.4)	0.6	(0.1 - 2.9)	12.7	(8.3 - 18.1)	9.6	(5.8 - 14.6)	34.1	(27.1 - 41.1)
Paraguay (1990)	179	45.9	(38.7 - 53.9)	10.6	(6.5 - 15.8)	4.3	(1.9 - 8.2)	12.6	(8.2 - 18.0)	6.9	(3.7 - 11.4)	11.5	(7.2 - 16.8)

Peru (1991/92)	779	58.6	(55.0 - 62.2)	19.7	(16.9 - 22.7)	1.7	(1.0 - 2.8)	15.1	(12.7 - 17.7)	10.5	(8.4 - 12.8)	11.6	(9.4 - 14.0)
Peru (1996)	1,217	56.9	(54.1 - 59.7)	17.7	(15.6 - 19.9)	0.9	(0.5 - 1.5)	18.9	(16.8 - 21.1)	9.2	(7.7 - 10.9)	10.2	(8.6 - 12.0)
Peru (2000)	907	46.6	(43.4 - 49.9)	12.6	(10.5 - 14.9)	0.5	(0.2 - 1.1)	15.4	(13.2 - 17.8)	11.4	(9.5 - 13.6)	6.7	(5.1 - 8.4)
Peru (2004/6)	1,095	52.7	(49.4 - 56.0)	12.2	(10.1 - 14.4)	0.3	(0.1 - 0.8)	20.9	(18.3 - 23.6)	10.9	(8.9 - 13.0)	8.5	(6.8 - 10.4)
Peru (2007/8)	1,628	59.5	(57.1 - 61.9)	13.6	(12.0 - 15.4)	0.1	(0.0 - 0.4)	22.2	(20.2 - 24.3)	15.3	(13.6 - 17.1)	8.2	(7.0 - 9.6)
Peru (2009)	4,517	44.2	(42.6 - 45.8)	16.9	(15.8 - 18.1)	0.1	(0.0 - 0.2)	12.7	(11.7 - 13.8)	10.5	(9.6 - 11.5)	4.0	(3.4 - 4.6)
Peru (2010)	2,072	57.4	(55.4 - 59.5)	13.0	(11.6 - 14.4)	0.3	(0.1 - 0.6)	19.8	(18.2 - 21.5)	15.6	(14.1 - 17.1)	8.8	(7.7 - 10.0)
Peru (2011)	2,116	53.1	(51.0 - 55.3)	14.2	(12.8 - 15.7)	0.0	(0.0 - 0.2)	18.0	(16.4 - 19.6)	11.6	(10.3 - 13.1)	9.3	(8.1 - 10.5)
Peru (2012)	2,404	58.2	(56.2 - 60.3)	13.9	(12.5 - 15.3)	0.0	(0.0 - 0.3)	22.4	(20.7 - 24.1)	15.6	(14.2 - 17.1)	6.3	(5.3 - 7.3)

CI= Confidence Interval
