# Supplementary material for: Contraceptive discontinuation, switching, abandonment and their reproductive consequences: An analysis of 1,539,071 episodes of reversible method use contributed from 61 countries that participated in DHS: Population base-analysis
Source: PLOS Glob Public Health. 2025 Oct 31;5(10):e0005174. doi: 10.1371/journal.pgph.0005174 (PMC12578211; doi:10.1371/journal.pgph.0005174)
Supplement: S9 Table — (PDF) [file pgph.0005174.s020.pdf]

S9 Table: Trends in 12-month method-related discontinuations

	Declining trends		Increasing trends		No of countries
	Significant	Not significant	Not significan	Significant	
Contraceptive method					
Oral contraceptive	7	2	4	5	18
IUD	7	2	3	3	15
Injectables	6	2	4	4	16
Condom	12	3	1	2	18
Implants		2	2	3	7
Periodic abstinence	3	8	1	4	16
Withdrawal	5	3	3	4	15
**Total**	40	22	18	25	105
