# Supplementary material for: Contraceptive discontinuation, switching, abandonment and their reproductive consequences: An analysis of 1,539,071 episodes of reversible method use contributed from 61 countries that participated in DHS: Population base-analysis
Source: PLOS Glob Public Health. 2025 Oct 31;5(10):e0005174. doi: 10.1371/journal.pgph.0005174 (PMC12578211; doi:10.1371/journal.pgph.0005174)
Supplement: S10 Table — (PDF) [file pgph.0005174.s021.pdf]

S10 Table : Medians of switching rates within 3 months following method related discontinuation, by method and status

Contraceptive method	At risk after discontinuation	Switched to LARC	SARC	Traditional	Permanent	Became pregnant
Oral contraceptives	44.1	7.1	22.9	6.0	0.0	9.5
IUDs	31.8	2.3	35.2	8.5	1.0	8.0
Injectables	61.5	5.5	17.1	2.6	0.0	8.2
Condoms	26.6	7.3	33.4	10.6	0.5	8.5
Implants	55.5	1.9	23.2	1.7	0.0	10.6
Periodic abstinence	17.3	5.3	48.1	12.4	2.1	4.7
Withdrawal	16.7	10.9	59.9	4.3	1.3	4.0
