# Supplementary material for: Contraceptive discontinuation, switching, abandonment and their reproductive consequences: An analysis of 1,539,071 episodes of reversible method use contributed from 61 countries that participated in DHS: Population base-analysis
Source: PLOS Glob Public Health. 2025 Oct 31;5(10):e0005174. doi: 10.1371/journal.pgph.0005174 (PMC12578211; doi:10.1371/journal.pgph.0005174)
Supplement: S11 Table — (PDF) [file pgph.0005174.s022.pdf]

S11.1 Table: Status at 3 months following method related discontinuation

Oral contraceptives (OCs)

	At risk		Switched to:								Became pregnant	
			LARC		SARC		Traditional		Permanent			
	Rate	95%CI	Rate	95%CI	Rate	95%CI	Rate	95%CI	Rate	95%CI	Rate	95%CI
**Sub-Saharan Africa**												
Benin (2017/18)	12.1	(7.0 - 18.8)	10.0	(5.4 - 16.3)	7.9	(3.9 - 13.7)	4.7	(1.8 - 9.6)	0.0	(0.0 - 0.0)	12.6	(7.3 - 19.3)
Burkina Faso (2010)	15.6	(9.8 - 22.6)	5.9	(2.6 - 11.0)	12.4	(7.3 - 18.9)	1.9	(0.4 - 5.6)	0.0	(0.0 - 0.0)	7.9	(4.0 - 13.5)
Burkina Faso (2021)	6.3	(3.0 - 11.3)	10.0	(5.6 - 15.8)	12.9	(7.9 - 19.2)	3.6	(1.3 - 7.8)	0.0	(0.0 - 0.0)	8.9	(4.8 - 14.5)
Côte d'Ivoire (2021)	3.7	(1.7 - 7.0)	1.2	(0.3 - 3.5)	11.9	(7.9 - 16.7)	4.4	(2.2 - 7.8)	0.0	(0.0 - 0.0)	7.1	(4.1 - 11.1)
Ethiopia (2005)	5.8	(3.5 - 8.8)	0.4	(0.0 - 1.8)	48.3	(42.6 - 53.9)	4.0	(2.2 - 6.6)	0.0	(0.0 - 0.0)	12.8	(9.3 - 16.9)
Ethiopia (2016)	5.3	(2.9 - 8.9)	8.5	(5.3 - 12.6)	40.4	(33.9 - 46.8)	4.4	(2.2 - 7.6)	0.0	(0.0 - 0.0)	9.6	(6.2 - 14.0)
Ghana (2022/23)	5.4	(3.2 - 8.4)	2.2	(0.9 - 4.4)	10.5	(7.3 - 14.3)	8.6	(5.8 - 12.2)	0.0	(0.0 - 0.0)	15.8	(11.9 - 20.2)
Guinea (2018)	0.4	(0.0 - 2.2)	9.0	(5.8 - 13.1)	12.5	(8.7 - 17.1)	1.6	(0.5 - 3.8)	0.0	(0.0 - 0.0)	5.6	(3.1 - 9.0)
Kenya (1998)	7.4	(4.8 - 10.7)	3.6	(1.9 - 6.2)	29.7	(24.7 - 34.9)	5.6	(3.4 - 8.6)	0.4	(0.0 - 1.8)	11.2	(7.9 - 15.0)
Kenya (2003)	10.3	(7.5 - 13.5)	4.6	(2.8 - 7.0)	28.9	(24.5 - 33.5)	5.0	(3.1 - 7.4)	0.4	(0.1 - 1.6)	12.5	(9.4 - 16.0)
Kenya (2014)	11.9	(9.5 - 14.7)	19.7	(16.6 - 23.0)	39.6	(35.6 - 43.5)	6.5	(4.7 - 8.7)	0.4	(0.1 - 1.2)	5.4	(3.8 - 7.4)
Kenya (2022)	8.6	(6.5 - 11.0)	27.2	(23.6 - 30.8)	19.1	(16.0 - 22.4)	4.2	(2.8 - 6.1)	0.1	(0.0 - 0.9)	3.7	(2.4 - 5.5)
Lesotho (2014)	20.6	(15.4 - 26.4)	4.4	(2.2 - 7.9)	41.4	(34.6 - 48.1)	0.8	(0.1 - 2.9)	0.0	(0.0 - 0.0)	9.5	(5.9 - 14.0)
Liberia (2013)	10.6	(6.6 - 15.7)	0.4	(0.0 - 2.7)	12.8	(8.4 - 18.2)	0.3	(0.0 - 2.6)	0.0	(0.0 - 0.0)	22.8	(16.9 - 29.3)
Liberia (2019/20)	5.6	(2.8 - 9.6)	2.8	(1.0 - 6.0)	15.2	(10.4 - 20.9)	2.2	(0.7 - 5.2)	0.0	(0.0 - 0.0)	8.1	(4.7 - 12.6)
Madagascar (2021)	4.6	(2.9 - 6.8)	10.2	(7.6 - 13.2)	44.9	(40.2 - 49.5)	7.5	(5.3 - 10.2)	0.0	(0.0 - 0.0)	3.6	(2.2 - 5.6)
Malawi (2004/5)	9.1	(5.3 - 14.0)	1.1	(0.2 - 3.8)	22.8	(16.8 - 29.5)	5.4	(2.7 - 9.6)	0.7	(0.1 - 3.2)	17.1	(11.8 - 23.3)
Malawi (2015/16)	6.1	(4.0 - 8.8)	6.8	(4.6 - 9.6)	26.0	(21.7 - 30.4)	0.8	(0.2 - 2.2)	2.5	(1.3 - 4.5)	10.0	(7.3 - 13.2)
Mozambique (2011)	5.6	(3.0 - 9.5)	0.0	(0.0 - 0.0)	12.2	(8.0 - 17.2)	0.3	(0.0 - 2.4)	0.0	(0.0 - 0.0)	13.5	(9.1 - 18.7)
Mozambique (2022/23)	5.5	(2.7 - 9.7)	10.1	(6.1 - 15.4)	23.7	(17.5 - 30.6)	1.9	(0.5 - 5.0)	0.0	(0.0 - 0.0)	8.6	(4.9 - 13.6)
Namibia (2013)	5.0	(2.2 - 9.6)	0.7	(0.1 - 3.6)	44.7	(36.3 - 52.8)	1.9	(0.5 - 5.4)	1.2	(0.2 - 4.3)	9.5	(5.3 - 15.1)
Niger (2012)	8.6	(4.5 - 14.3)	3.9	(1.5 - 8.5)	24.7	(17.5 - 32.5)	6.0	(2.7 - 11.2)	0.0	(0.0 - 0.0)	8.5	(4.4 - 14.2)
Nigeria (2013)	8.9	(4.5 - 15.1)	3.9	(1.3 - 8.8)	26.1	(18.3 - 34.6)	6.3	(2.8 - 12.0)	0.0	(0.0 - 0.0)	12.9	(7.4 - 19.9)
Nigeria (2018)	6.4	(4.0 - 9.6)	6.1	(3.7 - 9.3)	11.7	(8.3 - 15.7)	10.8	(7.5 - 14.7)	0.0	(0.0 - 0.0)	10.4	(7.2 - 14.3)
Rwanda (2010/11)	10.3	(7.6 - 13.6)	5.4	(3.5 - 7.9)	49.7	(44.7 - 54.5)	6.0	(4.0 - 8.7)	0.0	(0.0 - 0.0)	6.7	(4.5 - 9.5)
Rwanda (2014/15)	9.2	(6.9 - 12.0)	6.4	(4.4 - 8.8)	49.3	(44.8 - 53.7)	6.0	(4.1 - 8.3)	0.0	(0.0 - 0.0)	8.5	(6.2 - 11.2)
Rwanda (2019/20)	5.2	(3.5 - 7.3)	17.3	(14.2 - 20.7)	35.7	(31.6 - 39.8)	5.9	(4.1 - 8.2)	0.0	(0.0 - 0.0)	8.2	(6.1 - 10.8)
Senegal (2010/11)	12.2	(8.3 - 17.0)	2.1	(0.8 - 4.8)	20.4	(15.3 - 26.1)	4.5	(2.2 - 7.8)	0.0	(0.0 - 0.0)	12.9	(8.8 - 17.8)
Senegal (2015)	14.4	(9.3 - 20.5)	5.9	(2.9 - 10.4)	19.3	(13.4 - 26.0)	5.8	(2.8 - 10.3)	0.0	(0.0 - 0.0)	13.1	(8.3 - 19.0)
Senegal (2023)	11.9	(7.5 - 17.3)	5.4	(2.6 - 9.6)	10.9	(6.7 - 16.1)	1.5	(0.3 - 4.3)	0.0	(0.0 - 0.0)	15.1	(10.1 - 21.0)
Sierra Leone (2013)	3.8	(1.9 - 6.6)	7.0	(4.4 - 10.5)	27.2	(22.0 - 32.7)	1.0	(0.3 - 2.9)	0.0	(0.0 - 0.0)	12.3	(8.7 - 16.6)
Tanzania (2004/5)	13.0	(10.0 - 16.5)	0.5	(0.1 - 1.7)	31.6	(27.2 - 36.1)	5.3	(3.5 - 7.8)	0.0	(0.0 - 0.0)	11.3	(8.5 - 14.6)
Tanzania (2015/16)	8.9	(6.0 - 12.5)	11.0	(7.8 - 14.9)	24.6	(19.8 - 29.6)	6.9	(4.4 - 10.2)	0.0	(0.0 - 0.0)	11.5	(8.2 - 15.5)

Tanzania (2022)	5.2	(2.5 - 9.3)	11.2	(6.9 - 16.7)	10.8	(6.6 - 16.2)	3.4	(1.3 - 7.1)	0.0	(0.0 - 0.0)	8.2	(4.6 - 13.0)
Uganda (2011)	8.4	(5.0 - 12.8)	6.2	(3.4 - 10.2)	24.7	(18.8 - 31.0)	5.1	(2.6 - 8.9)	0.0	(0.0 - 0.0)	14.8	(10.2 - 20.2)
Zambia (2013/14)	10.4	(8.2 - 12.9)	6.8	(5.1 - 8.9)	33.1	(29.5 - 36.7)	2.7	(1.7 - 4.2)	0.0	(0.0 - 0.0)	11.9	(9.6 - 14.5)
Zambia (2018/19)	7.8	(5.7 - 10.3)	5.4	(3.6 - 7.5)	28.0	(24.2 - 32.0)	1.4	(0.7 - 2.8)	0.0	(0.0 - 0.0)	9.3	(7.0 - 12.0)
Zimbabwe (1994)	10.8	(8.1 - 14.1)	2.6	(1.4 - 4.5)	14.7	(11.5 - 18.4)	6.5	(4.4 - 9.2)	0.5	(0.1 - 1.6)	18.8	(15.2 - 22.8)
Zimbabwe (1999)	15.4	(11.9 - 19.2)	2.1	(1.0 - 4.0)	33.4	(28.6 - 38.2)	3.0	(1.6 - 5.1)	0.5	(0.1 - 1.7)	13.1	(9.9 - 16.7)
Zimbabwe (2005/6)	17.1	(13.8 - 20.7)	1.1	(0.4 - 2.5)	34.8	(30.5 - 39.2)	1.2	(0.5 - 2.5)	0.0	(0.0 - 0.0)	12.7	(9.8 - 15.9)
Zimbabwe (2010/11)	15.5	(12.5 - 18.9)	7.4	(5.3 - 9.9)	26.3	(22.5 - 30.3)	1.7	(0.8 - 3.2)	0.3	(0.1 - 1.2)	16.6	(13.5 - 20.0)
Zimbabwe (2015)	13.1	(10.9 - 15.6)	23.7	(20.9 - 26.7)	19.1	(16.5 - 21.9)	1.4	(0.7 - 2.4)	0.1	(0.0 - 0.6)	14.9	(12.5 - 17.5)
**North Africa Western Asia and Europe**												
Egypt (1992/93)	9.5	(7.6 - 11.6)	34.6	(31.3 - 37.9)	6.6	(5.0 - 8.4)	0.8	(0.3 - 1.6)	0.1	(0.0 - 0.7)	24.3	(21.4 - 27.3)
Egypt (1995/96)	7.1	(5.6 - 8.9)	32.3	(29.3 - 35.3)	8.7	(7.0 - 10.6)	2.2	(1.4 - 3.3)	0.0	(0.0 - 0.0)	21.3	(18.8 - 24.0)
Egypt (2000)	9.6	(7.6 - 11.7)	32.5	(29.3 - 35.8)	19.4	(16.8 - 22.3)	0.8	(0.4 - 1.7)	0.2	(0.0 - 0.7)	16.5	(14.0 - 19.2)
Egypt (2003)	12.7	(9.9 - 15.9)	35.0	(30.7 - 39.2)	22.6	(18.9 - 26.4)	1.5	(0.7 - 3.0)	0.1	(0.0 - 1.1)	11.1	(8.5 - 14.1)
Egypt (2005)	11.0	(9.3 - 12.8)	38.2	(35.4 - 40.9)	16.9	(14.9 - 19.1)	2.2	(1.5 - 3.1)	0.0	(0.0 - 0.4)	11.1	(9.4 - 13.0)
Egypt (2008)	12.8	(10.4 - 15.5)	33.8	(30.1 - 37.4)	15.4	(12.8 - 18.3)	2.3	(1.3 - 3.7)	0.2	(0.0 - 0.9)	12.3	(9.9 - 15.0)
Egypt (2014)	13.1	(11.4 - 15.1)	33.3	(30.8 - 35.9)	12.4	(10.6 - 14.2)	6.0	(4.8 - 7.4)	0.0	(0.0 - 0.0)	9.6	(8.1 - 11.3)
Jordan (1990)	5.5	(3.7 - 7.8)	18.9	(15.5 - 22.6)	7.4	(5.2 - 10.0)	20.3	(16.7 - 24.0)	0.2	(0.0 - 1.2)	25.3	(21.4 - 29.3)
Jordan (1997)	5.8	(4.2 - 7.9)	21.0	(17.9 - 24.4)	17.6	(14.7 - 20.7)	26.5	(23.1 - 30.1)	0.2	(0.0 - 0.9)	15.1	(12.4 - 18.0)
Jordan (2002)	7.0	(4.9 - 9.5)	26.0	(22.1 - 30.1)	11.7	(9.0 - 14.8)	23.1	(19.4 - 27.0)	0.3	(0.1 - 1.3)	14.2	(11.2 - 17.5)
Jordan (2007)	8.7	(6.6 - 11.1)	23.4	(20.1 - 26.9)	14.7	(12.0 - 17.7)	21.0	(17.8 - 24.3)	0.2	(0.0 - 1.0)	10.7	(8.4 - 13.4)
Jordan (2009)	9.5	(7.6 - 11.7)	19.5	(16.8 - 22.4)	13.9	(11.6 - 16.5)	31.8	(28.5 - 35.1)	0.4	(0.1 - 1.0)	10.8	(8.8 - 13.2)
Jordan (2012)	15.6	(13.2 - 18.2)	19.6	(16.9 - 22.4)	18.3	(15.7 - 21.0)	31.1	(27.9 - 34.3)	0.0	(0.0 - 0.8)	5.1	(3.8 - 6.8)
Jordan (2017/18)	9.3	(6.7 - 12.4)	17.8	(14.2 - 21.7)	11.2	(8.3 - 14.5)	17.1	(13.6 - 21.0)	0.0	(0.0 - 0.0)	12.5	(9.5 - 16.0)
Jordan (2023)	8.5	(5.8 - 11.8)	21.2	(16.9 - 25.7)	6.8	(4.4 - 9.9)	15.0	(11.4 - 19.1)	1.0	(0.3 - 2.6)	15.7	(12.0 - 19.8)
Moldova (2005)	6.7	(3.1 - 12.0)	22.0	(15.1 - 29.6)	17.6	(11.4 - 24.8)	34.6	(26.3 - 43.0)	2.0	(0.5 - 5.7)	2.7	(0.8 - 6.7)
Morocco (1992)	8.3	(6.1 - 10.8)	10.0	(7.6 - 12.7)	6.2	(4.4 - 8.5)	21.2	(17.9 - 24.8)	2.6	(1.5 - 4.3)	18.4	(15.3 - 21.8)
Morocco (2003/4)	7.9	(6.2 - 9.8)	17.4	(15.0 - 20.0)	22.7	(20.0 - 25.5)	28.7	(25.8 - 31.8)	1.3	(0.7 - 2.2)	7.9	(6.2 - 9.8)
Türkiye (1993)	9.2	(6.2 - 13.0)	16.7	(12.5 - 21.3)	17.3	(13.1 - 22.0)	30.2	(24.9 - 35.7)	0.8	(0.2 - 2.6)	8.8	(5.8 - 12.5)
Türkiye (1998)	8.9	(5.6 - 13.0)	13.8	(9.7 - 18.6)	14.5	(10.3 - 19.3)	39.9	(33.6 - 46.2)	1.1	(0.3 - 3.2)	10.7	(7.1 - 15.1)
Türkiye (2003/4)	8.0	(5.5 - 11.2)	15.0	(11.5 - 19.0)	28.5	(23.9 - 33.3)	28.8	(24.1 - 33.6)	0.6	(0.1 - 2.0)	8.5	(5.9 - 11.7)
Türkiye (2018/19)	6.4	(2.8 - 11.9)	8.6	(4.4 - 14.7)	27.5	(19.6 - 36.0)	19.0	(12.3 - 26.7)	0.1	(0.0 - 4.5)	8.1	(4.0 - 14.0)
Yemen (2013)	9.5	(7.9 - 11.1)	15.0	(13.1 - 16.9)	12.4	(10.7 - 14.2)	11.2	(9.5 - 12.9)	0.4	(0.1 - 0.9)	17.1	(15.1 - 19.2)
**Central, South & Southeast Asia**												
Bangladesh (1993/94)	9.2	(7.7 - 10.9)	4.9	(3.8 - 6.2)	32.1	(29.5 - 34.7)	13.1	(11.3 - 15.0)	0.6	(0.3 - 1.2)	11.9	(10.2 - 13.8)
Bangladesh (1996/97)	7.5	(6.0 - 9.1)	3.7	(2.7 - 4.9)	36.4	(33.6 - 39.2)	14.3	(12.4 - 16.5)	0.5	(0.2 - 1.0)	13.8	(11.8 - 15.9)
Bangladesh (1999/0)	8.6	(7.2 - 10.2)	2.7	(2.0 - 3.7)	40.7	(38.1 - 43.3)	14.1	(12.4 - 16.0)	0.3	(0.1 - 0.8)	9.5	(8.0 - 11.1)
Bangladesh (2004)	9.3	(7.9 - 10.7)	2.3	(1.7 - 3.1)	46.3	(43.9 - 48.7)	18.6	(16.8 - 20.6)	0.4	(0.2 - 0.9)	7.5	(6.3 - 8.8)
Bangladesh (2011)	12.3	(10.7 - 14.0)	3.7	(2.9 - 4.8)	52.4	(49.8 - 54.9)	8.4	(7.1 - 9.9)	2.3	(1.6 - 3.1)	5.4	(4.4 - 6.7)
Bangladesh (2014)	12.8	(10.9 - 14.8)	6.2	(4.9 - 7.7)	55.5	(52.6 - 58.4)	7.7	(6.3 - 9.4)	2.2	(1.4 - 3.2)	3.2	(2.3 - 4.3)
Bangladesh (2017/18)	11.4	(9.9 - 13.1)	8.2	(6.9 - 9.6)	61.5	(59.0 - 63.9)	11.0	(9.6 - 12.7)	0.6	(0.3 - 1.0)	2.1	(1.4 - 2.9)

Bangladesh (2022)	9.9	(8.2 - 11.9)	4.9	(3.7 - 6.4)	54.4	(51.2 - 57.5)	9.4	(7.7 - 11.3)	0.5	(0.2 - 1.2)	2.8	(1.9 - 4.0)
Cambodia (2010/11)	8.2	(5.9 - 10.8)	4.5	(2.9 - 6.6)	22.8	(19.2 - 26.6)	15.0	(12.0 - 18.3)	2.3	(1.2 - 3.9)	11.6	(8.9 - 14.6)
Cambodia (2014)	11.5	(8.9 - 14.5)	7.6	(5.5 - 10.2)	17.7	(14.5 - 21.2)	19.3	(16.0 - 22.9)	0.2	(0.0 - 1.1)	13.2	(10.4 - 16.4)
Cambodia (2021/22)	8.2	(6.0 - 10.7)	4.2	(2.7 - 6.2)	21.2	(17.8 - 24.7)	16.3	(13.3 - 19.6)	0.3	(0.1 - 1.2)	13.0	(10.3 - 16.0)
India (2005/6)	7.4	(6.3 - 8.7)	3.7	(2.9 - 4.6)	17.4	(15.7 - 19.2)	16.6	(14.9 - 18.3)	6.1	(5.0 - 7.2)	10.3	(9.0 - 11.8)
India (2015/16)	4.6	(4.1 - 5.2)	1.9	(1.6 - 2.3)	18.5	(17.6 - 19.5)	14.7	(13.9 - 15.6)	5.0	(4.5 - 5.5)	5.6	(5.1 - 6.2)
India (2019/21)	3.3	(3.0 - 3.7)	1.2	(0.9 - 1.4)	28.2	(27.3 - 29.1)	12.7	(12.0 - 13.4)	3.5	(3.1 - 3.9)	5.8	(5.3 - 6.3)
Indonesia (1991)	10.1	(8.1 - 12.3)	14.5	(12.1 - 17.1)	25.0	(22.0 - 28.1)	4.0	(2.8 - 5.6)	0.6	(0.2 - 1.3)	8.7	(6.9 - 10.9)
Indonesia (1994)	7.7	(6.1 - 9.5)	10.7	(8.8 - 12.7)	34.6	(31.6 - 37.6)	3.0	(2.1 - 4.3)	1.5	(0.9 - 2.5)	9.5	(7.8 - 11.5)
Indonesia (1997)	9.7	(8.1 - 11.5)	10.1	(8.4 - 11.9)	48.8	(45.8 - 51.6)	5.6	(4.4 - 7.1)	0.4	(0.2 - 1.0)	5.0	(3.8 - 6.4)
Indonesia (2002/3)	12.0	(10.0 - 14.2)	6.6	(5.1 - 8.4)	53.6	(50.3 - 56.8)	2.4	(1.6 - 3.6)	0.5	(0.2 - 1.1)	4.2	(3.0 - 5.6)
Indonesia (2007)	9.1	(7.7 - 10.7)	5.7	(4.6 - 7.0)	56.7	(54.2 - 59.2)	3.0	(2.2 - 4.0)	0.0	(0.0 - 0.6)	9.0	(7.6 - 10.5)
Indonesia (2012)	10.0	(8.5 - 11.6)	6.5	(5.3 - 7.9)	60.0	(57.4 - 62.5)	7.0	(5.7 - 8.3)	0.4	(0.2 - 0.9)	3.0	(2.2 - 3.9)
Indonesia (2017)	9.0	(7.7 - 10.4)	7.4	(6.2 - 8.6)	61.7	(59.4 - 63.9)	5.8	(4.8 - 6.9)	0.2	(0.1 - 0.6)	3.3	(2.6 - 4.3)
Kazakhstan (1999)	6.2	(3.3 - 10.4)	16.2	(11.2 - 22.0)	29.1	(22.6 - 35.9)	25.0	(18.9 - 31.6)	0.0	(0.0 - 0.0)	7.4	(4.1 - 11.8)
Maldives (2009)	1.9	(0.3 - 6.3)	0.0	(0.0 - 0.0)	29.5	(20.7 - 38.8)	10.3	(5.3 - 17.4)	0.0	(0.0 - 0.0)	4.2	(1.4 - 9.6)
Myanmar (2015/16)	10.3	(7.5 - 13.5)	3.5	(2.0 - 5.6)	52.6	(47.6 - 57.4)	1.9	(0.9 - 3.6)	0.0	(0.0 - 0.0)	5.1	(3.3 - 7.6)
Nepal (2011)	9.7	(6.5 - 13.7)	2.7	(1.2 - 5.2)	23.9	(18.9 - 29.2)	7.6	(4.8 - 11.3)	2.1	(0.9 - 4.4)	6.1	(3.6 - 9.4)
Nepal (2016)	6.5	(3.9 - 9.9)	8.1	(5.2 - 11.8)	23.1	(18.2 - 28.4)	18.7	(14.3 - 23.7)	3.7	(1.9 - 6.5)	6.9	(4.2 - 10.4)
Nepal (2022)	10.3	(7.1 - 14.2)	7.6	(4.9 - 11.1)	21.4	(16.8 - 26.4)	19.1	(14.7 - 23.9)	1.6	(0.6 - 3.6)	5.9	(3.5 - 9.1)
Pakistan (2012/13)	10.3	(6.7 - 14.7)	4.2	(2.1 - 7.4)	21.6	(16.5 - 27.2)	8.7	(5.5 - 12.9)	6.2	(3.5 - 9.9)	11.7	(7.9 - 16.3)
Pakistan (2017/18)	8.2	(4.3 - 13.5)	5.2	(2.3 - 9.9)	18.9	(12.9 - 25.9)	2.2	(0.6 - 5.8)	0.8	(0.1 - 3.8)	15.4	(9.9 - 21.9)
Philippines (1993)	8.9	(6.3 - 12.2)	2.6	(1.3 - 4.7)	7.3	(4.9 - 10.3)	22.5	(18.3 - 26.9)	0.4	(0.1 - 1.6)	19.6	(15.7 - 23.9)
Philippines (1998)	7.3	(5.1 - 10.0)	2.4	(1.3 - 4.2)	14.1	(11.0 - 17.6)	31.4	(27.1 - 35.8)	0.3	(0.0 - 1.3)	13.7	(10.7 - 17.1)
Philippines (2003)	7.1	(5.2 - 9.3)	3.3	(2.1 - 5.0)	20.9	(17.7 - 24.3)	18.6	(15.5 - 21.9)	0.6	(0.2 - 1.6)	16.3	(13.4 - 19.4)
Philippines (2022)	6.8	(5.2 - 8.7)	4.8	(3.5 - 6.5)	17.9	(15.3 - 20.7)	15.7	(13.3 - 18.4)	0.0	(0.0 - 0.7)	14.0	(11.6 - 16.5)
Vietnam (2002)	5.0	(2.2 - 9.5)	30.8	(23.4 - 38.6)	10.8	(6.4 - 16.6)	31.8	(24.3 - 39.6)	4.8	(2.1 - 9.2)	10.6	(6.2 - 16.4)
**Latin America & Caribbean**												
Bolivia (1994)	10.4	(7.2 - 14.2)	7.1	(4.5 - 10.4)	22.0	(17.4 - 26.9)	30.8	(25.6 - 36.2)	0.4	(0.1 - 1.9)	10.2	(7.1 - 14.0)
Brazil (1996)	11.6	(9.6 - 13.7)	3.4	(2.4 - 4.7)	21.3	(18.8 - 24.0)	17.9	(15.5 - 20.4)	5.2	(3.9 - 6.8)	13.9	(11.8 - 16.2)
Colombia (1990)	11.8	(9.1 - 14.8)	10.0	(7.6 - 12.8)	20.1	(16.7 - 23.7)	15.9	(12.8 - 19.2)	4.3	(2.8 - 6.3)	17.7	(14.5 - 21.2)
Colombia (1995)	10.0	(8.2 - 12.0)	7.9	(6.3 - 9.7)	24.3	(21.7 - 27.0)	23.7	(21.1 - 26.3)	3.9	(2.8 - 5.2)	13.0	(11.0 - 15.2)
Colombia (2000)	8.5	(6.8 - 10.5)	7.6	(5.9 - 9.5)	29.2	(26.1 - 32.3)	20.0	(17.4 - 22.8)	4.6	(3.3 - 6.2)	15.6	(13.3 - 18.1)
Colombia (2005)	9.1	(8.1 - 10.3)	8.9	(7.8 - 10.0)	28.6	(26.9 - 30.4)	11.9	(10.7 - 13.1)	6.4	(5.5 - 7.4)	16.6	(15.2 - 18.1)
Colombia (2010)	8.0	(7.0 - 9.0)	10.3	(9.2 - 11.5)	33.2	(31.4 - 35.0)	8.7	(7.7 - 9.8)	5.1	(4.3 - 6.0)	10.7	(9.5 - 11.9)
Colombia (2015/16)	8.1	(6.7 - 9.6)	9.7	(8.2 - 11.4)	36.5	(33.9 - 39.1)	8.0	(6.6 - 9.5)	4.0	(3.0 - 5.2)	7.4	(6.1 - 8.9)
Dominican Republic (1991)	10.9	(8.6 - 13.6)	6.0	(4.2 - 8.1)	4.4	(3.0 - 6.3)	13.4	(10.8 - 16.3)	1.7	(0.9 - 3.0)	22.3	(19.1 - 25.8)
Dominican Republic (1996)	6.7	(5.1 - 8.7)	5.5	(4.0 - 7.3)	9.7	(7.7 - 12.0)	13.0	(10.7 - 15.5)	1.4	(0.7 - 2.4)	29.6	(26.4 - 32.9)
Dominican Republic (2002)	10.2	(9.0 - 11.5)	4.5	(3.7 - 5.4)	13.6	(12.2 - 15.1)	9.5	(8.3 - 10.8)	2.3	(1.8 - 3.0)	24.3	(22.5 - 26.2)
Guatemala (1995)	10.0	(6.8 - 14.0)	6.3	(3.8 - 9.7)	14.9	(11.0 - 19.5)	3.3	(1.6 - 6.0)	6.7	(4.1 - 10.1)	16.9	(12.6 - 21.6)
Guatemala (1998/99)	4.5	(2.2 - 7.9)	0.0	(0.0 - 0.0)	26.9	(21.1 - 33.1)	6.7	(3.9 - 10.7)	5.7	(3.1 - 9.4)	16.3	(11.7 - 21.7)

Guatemala (2014/15)	13.3	(9.8 - 17.4)	7.9	(5.2 - 11.3)	28.4	(23.4 - 33.5)	7.3	(4.8 - 10.6)	2.3	(1.0 - 4.4)	11.6	(8.3 - 15.5)
Honduras (2011/12)	8.4	(7.2 - 9.7)	3.2	(2.5 - 4.1)	40.8	(38.6 - 43.0)	11.2	(9.9 - 12.7)	0.6	(0.3 - 1.0)	24.7	(22.8 - 26.7)
Nicaragua (1998)	7.9	(6.3 - 9.7)	8.3	(6.7 - 10.2)	26.8	(24.0 - 29.6)	7.1	(5.6 - 8.8)	5.7	(4.4 - 7.3)	16.3	(14.1 - 18.7)
Paraguay (1990)	6.2	(4.5 - 8.3)	4.3	(2.9 - 6.1)	40.0	(36.1 - 43.9)	27.5	(24.0 - 31.1)	0.4	(0.1 - 1.3)	7.9	(6.0 - 10.2)
Peru (1991/92)	8.0	(6.1 - 10.1)	8.8	(6.9 - 11.0)	25.5	(22.4 - 28.7)	28.5	(25.3 - 31.9)	0.0	(0.0 - 0.0)	13.3	(10.9 - 15.9)
Peru (1996)	9.6	(8.2 - 11.2)	10.1	(8.7 - 11.7)	36.7	(34.3 - 39.2)	18.4	(16.4 - 20.4)	1.9	(1.3 - 2.7)	9.0	(7.6 - 10.5)
Peru (2000)	7.5	(6.3 - 8.9)	6.4	(5.2 - 7.6)	43.4	(40.9 - 45.8)	14.8	(13.1 - 16.6)	1.8	(1.3 - 2.6)	9.6	(8.2 - 11.1)
Peru (2004/6)	9.3	(7.8 - 11.0)	3.3	(2.4 - 4.4)	52.2	(49.4 - 54.8)	13.1	(11.4 - 15.0)	0.0	(0.0 - 0.4)	8.7	(7.2 - 10.3)
Peru (2007/8)	10.0	(8.7 - 11.3)	2.3	(1.7 - 3.0)	49.4	(47.2 - 51.6)	18.2	(16.5 - 19.9)	0.0	(0.0 - 0.3)	8.7	(7.5 - 10.0)
Peru (2009)	8.1	(7.0 - 9.3)	1.5	(1.0 - 2.0)	60.0	(58.0 - 62.1)	12.4	(11.1 - 13.9)	0.2	(0.1 - 0.4)	7.6	(6.6 - 8.8)
Peru (2010)	7.6	(6.5 - 8.7)	1.5	(1.0 - 2.1)	55.3	(53.1 - 57.4)	18.4	(16.7 - 20.1)	0.0	(0.0 - 0.3)	8.7	(7.5 - 9.9)
Peru (2011)	6.9	(5.9 - 8.1)	2.1	(1.6 - 2.8)	56.6	(54.4 - 58.7)	18.9	(17.2 - 20.6)	0.2	(0.1 - 0.5)	7.1	(6.0 - 8.3)
Peru (2012)	7.9	(6.8 - 9.0)	0.9	(0.6 - 1.4)	56.8	(54.7 - 58.9)	17.1	(15.5 - 18.7)	0.2	(0.1 - 0.5)	8.6	(7.5 - 9.9)

CI= Confidence Interval

S11.2 Table: Status at 3 months following method related discontinuation

IUD

	At risk		**Switched to:**								Became pregnant	
			LARC		SARC		Traditional		Permanent			
	Rate	95%CI	Rate	95%CI	Rate	95%CI	Rate	95%CI	Rate	95%CI	Rate	95%CI
**Sub-Saharan Africa**												
Guinea (2018)	0.0	(0.0 - 0.0)	10.1	(6.2 - 15.0)	21.1	(15.5 - 27.3)	0.7	(0.1 - 3.1)	0.0	(0.0 - 0.0)	5.1	(2.5 - 8.9)
**North Africa Western Asia and Europe**												
Egypt (1992/93)	13.6	(11.0 - 16.5)	5.1	(3.5 - 7.1)	37.2	(33.3 - 41.1)	2.7	(1.6 - 4.2)	0.3	(0.0 - 1.1)	17.5	(14.6 - 20.7)
Egypt (1995/96)	12.1	(10.1 - 14.3)	3.7	(2.6 - 5.1)	38.7	(35.5 - 41.8)	2.2	(1.4 - 3.4)	0.0	(0.0 - 0.0)	20.5	(18.0 - 23.2)
Egypt (2000)	10.1	(8.3 - 12.1)	5.8	(4.5 - 7.4)	46.0	(42.8 - 49.1)	1.2	(0.6 - 2.0)	0.4	(0.2 - 1.1)	15.4	(13.2 - 17.7)
Egypt (2003)	10.8	(8.3 - 13.5)	3.4	(2.1 - 5.2)	48.5	(44.2 - 52.6)	2.2	(1.2 - 3.7)	0.0	(0.0 - 0.0)	14.9	(12.1 - 18.0)
Egypt (2005)	9.6	(8.0 - 11.3)	5.3	(4.1 - 6.6)	49.1	(46.3 - 51.9)	1.2	(0.7 - 2.0)	0.0	(0.0 - 0.0)	12.9	(11.1 - 14.8)
Egypt (2008)	12.9	(10.5 - 15.6)	4.7	(3.2 - 6.4)	43.1	(39.3 - 46.8)	0.9	(0.4 - 1.8)	0.0	(0.0 - 0.0)	15.6	(13.0 - 18.5)
Egypt (2014)	11.3	(9.2 - 13.6)	2.4	(1.5 - 3.6)	49.9	(46.3 - 53.3)	4.3	(3.1 - 5.9)	0.0	(0.0 - 0.0)	13.0	(10.8 - 15.5)
Jordan (1990)	9.7	(6.9 - 13.1)	1.0	(0.3 - 2.5)	24.8	(20.4 - 29.4)	11.4	(8.4 - 15.0)	1.8	(0.8 - 3.6)	27.6	(23.0 - 32.3)
Jordan (1997)	12.1	(9.1 - 15.5)	2.1	(1.0 - 3.9)	29.3	(24.8 - 33.8)	16.8	(13.3 - 20.7)	1.0	(0.3 - 2.4)	20.6	(16.8 - 24.8)
Jordan (2002)	9.2	(6.5 - 12.5)	0.8	(0.2 - 2.2)	28.1	(23.5 - 32.8)	22.2	(18.0 - 26.6)	0.0	(0.0 - 0.0)	18.6	(14.7 - 22.8)
Jordan (2007)	7.6	(5.3 - 10.4)	1.2	(0.5 - 2.7)	32.6	(28.1 - 37.1)	18.8	(15.2 - 22.7)	0.1	(0.0 - 1.2)	17.1	(13.7 - 20.9)
Jordan (2009)	13.0	(10.0 - 16.4)	1.6	(0.7 - 3.1)	39.3	(34.6 - 43.9)	20.5	(16.8 - 24.5)	0.1	(0.0 - 1.1)	14.3	(11.1 - 17.8)
Jordan (2012)	11.3	(8.7 - 14.4)	1.5	(0.7 - 3.0)	35.7	(31.3 - 40.0)	25.3	(21.5 - 29.4)	0.2	(0.0 - 1.1)	11.2	(8.6 - 14.3)
Jordan (2017/18)	7.8	(4.7 - 11.9)	3.0	(1.2 - 6.0)	24.0	(18.5 - 30.0)	12.6	(8.5 - 17.5)	0.8	(0.1 - 2.9)	11.7	(7.8 - 16.5)
Jordan (2023)	19.7	(14.3 - 25.8)	3.1	(1.2 - 6.4)	11.9	(7.7 - 17.0)	14.2	(9.6 - 19.7)	0.0	(0.0 - 0.0)	15.2	(10.5 - 20.8)
Moldova (2005)	15.7	(9.7 - 23.0)	2.4	(0.6 - 6.6)	31.1	(22.9 - 39.7)	23.6	(16.3 - 31.7)	2.9	(0.8 - 7.2)	6.1	(2.7 - 11.5)
Morocco (2003/4)	11.8	(6.9 - 18.0)	0.0	(0.0 - 0.0)	69.5	(60.7 - 76.7)	9.8	(5.4 - 15.7)	0.2	(0.0 - 3.7)	2.8	(0.9 - 6.8)
Türkiye (1993)	17.8	(12.9 - 23.4)	1.5	(0.4 - 4.0)	29.3	(23.2 - 35.7)	30.1	(24.0 - 36.5)	1.1	(0.2 - 3.4)	8.7	(5.3 - 13.1)
Türkiye (1998)	11.6	(7.1 - 17.3)	1.0	(0.1 - 3.8)	29.0	(22.0 - 36.4)	31.0	(23.7 - 38.4)	0.9	(0.1 - 3.7)	12.7	(8.0 - 18.5)
Türkiye (2003/4)	11.5	(7.4 - 16.6)	0.9	(0.2 - 3.4)	42.2	(35.0 - 49.3)	31.9	(25.3 - 38.7)	0.2	(0.0 - 2.5)	5.1	(2.5 - 9.0)
Yemen (2013)	12.0	(8.3 - 16.4)	1.8	(0.6 - 4.1)	46.7	(40.4 - 52.9)	8.6	(5.5 - 12.6)	0.0	(0.0 - 0.0)	12.2	(8.4 - 16.6)
**Central, South & Southeast Asia**												
Bangladesh (1993/94)	8.9	(5.2 - 13.9)	0.6	(0.1 - 3.0)	56.2	(48.3 - 63.4)	9.4	(5.6 - 14.5)	1.1	(0.2 - 3.7)	6.6	(3.5 - 11.0)
Bangladesh (1996/97)	5.7	(2.5 - 10.7)	0.0	(0.0 - 0.0)	62.7	(53.5 - 70.5)	6.9	(3.3 - 12.3)	3.8	(1.4 - 8.3)	4.1	(1.5 - 8.6)
India (2005/6)	7.4	(5.5 - 9.7)	1.1	(0.5 - 2.2)	26.5	(23.1 - 30.1)	8.2	(6.2 - 10.6)	6.6	(4.8 - 8.8)	10.0	(7.8 - 12.5)
India (2015/16)	8.2	(7.0 - 9.6)	4.1	(3.2 - 5.1)	18.8	(17.0 - 20.7)	6.2	(5.1 - 7.4)	5.2	(4.2 - 6.4)	4.8	(3.9 - 5.9)
India (2019/21)	5.8	(5.0 - 6.7)	3.3	(2.7 - 4.0)	16.9	(15.6 - 18.3)	11.8	(10.7 - 13.1)	4.8	(4.0 - 5.6)	6.8	(5.9 - 7.8)
Indonesia (1991)	8.5	(6.0 - 11.5)	3.9	(2.3 - 6.2)	47.6	(42.5 - 52.4)	3.7	(2.1 - 5.9)	3.4	(1.9 - 5.5)	5.1	(3.2 - 7.6)
Indonesia (1994)	6.3	(4.1 - 9.2)	4.4	(2.6 - 6.9)	58.0	(52.6 - 63.0)	3.5	(1.9 - 5.9)	1.3	(0.5 - 3.0)	8.5	(5.9 - 11.8)

Indonesia (1997)	2.2	(0.7 - 4.9)	2.6	(1.0 - 5.5)	65.5	(58.5 - 71.7)	4.2	(2.0 - 7.6)	0.0	(0.0 - 0.0)	2.1	(0.7 - 4.8)
Indonesia (2002/3)	4.4	(1.6 - 9.3)	0.2	(0.0 - 4.3)	72.4	(63.2 - 79.6)	7.7	(3.7 - 13.5)	1.1	(0.1 - 4.7)	4.2	(1.6 - 9.1)
Indonesia (2017)	12.1	(7.8 - 17.4)	3.3	(1.3 - 6.7)	51.8	(44.1 - 58.9)	8.9	(5.2 - 13.7)	0.0	(0.0 - 0.0)	5.3	(2.6 - 9.3)
Kazakhstan (1999)	15.8	(10.8 - 21.7)	2.8	(1.0 - 6.1)	21.6	(15.8 - 28.0)	16.0	(11.0 - 21.8)	0.0	(0.0 - 0.0)	14.8	(10.0 - 20.6)
Kyrgyz Republic (2012)	7.3	(3.6 - 12.7)	0.3	(0.0 - 3.5)	28.2	(20.6 - 36.2)	7.5	(3.7 - 12.9)	1.2	(0.2 - 4.5)	16.9	(10.9 - 23.9)
Pakistan (2012/13)	17.5	(12.6 - 23.1)	2.0	(0.6 - 4.7)	25.5	(19.6 - 31.7)	9.3	(5.8 - 13.9)	1.1	(0.2 - 3.4)	12.8	(8.6 - 17.8)
Tajikistan (2012)	10.3	(5.4 - 17.1)	0.2	(0.0 - 4.5)	21.6	(14.2 - 30.0)	5.5	(2.2 - 11.1)	0.7	(0.0 - 4.5)	16.0	(9.7 - 23.8)
Tajikistan (2017)	13.2	(8.3 - 19.2)	0.1	(0.0 - 3.4)	12.2	(7.5 - 18.1)	0.5	(0.0 - 3.3)	0.8	(0.1 - 3.6)	8.7	(4.8 - 14.0)
Vietnam (1997)	8.1	(4.8 - 12.6)	0.4	(0.0 - 2.5)	21.7	(16.1 - 27.9)	20.3	(14.9 - 26.4)	5.5	(2.9 - 9.4)	14.5	(9.9 - 19.9)
Vietnam (2002)	7.9	(4.5 - 12.5)	1.9	(0.6 - 4.9)	34.3	(27.3 - 41.4)	29.6	(23.0 - 36.5)	1.9	(0.5 - 4.8)	15.0	(10.2 - 20.8)
**Latin America & Caribbean**												
Colombia (1990)	12.6	(8.4 - 17.8)	1.6	(0.4 - 4.2)	46.9	(39.7 - 53.8)	13.3	(9.0 - 18.6)	8.9	(5.4 - 13.5)	5.7	(3.0 - 9.6)
Colombia (1995)	10.9	(7.3 - 15.3)	1.6	(0.5 - 4.0)	46.1	(39.6 - 52.3)	11.0	(7.4 - 15.4)	4.4	(2.3 - 7.6)	8.1	(5.0 - 12.0)
Colombia (2000)	11.8	(7.6 - 17.0)	0.5	(0.0 - 2.8)	54.4	(46.8 - 61.4)	15.8	(10.9 - 21.6)	4.1	(1.8 - 7.7)	4.7	(2.2 - 8.5)
Colombia (2005)	8.3	(6.3 - 10.5)	0.6	(0.2 - 1.5)	48.5	(44.7 - 52.3)	10.1	(7.9 - 12.5)	6.8	(5.1 - 8.9)	8.1	(6.2 - 10.3)
Colombia (2010)	6.7	(4.9 - 8.8)	6.6	(4.8 - 8.7)	50.6	(46.7 - 54.4)	5.4	(3.9 - 7.4)	6.4	(4.6 - 8.4)	3.9	(2.6 - 5.6)
Colombia (2015/16)	5.4	(2.8 - 9.1)	8.5	(5.2 - 12.9)	50.1	(42.9 - 56.7)	1.9	(0.6 - 4.5)	5.9	(3.2 - 9.8)	3.2	(1.3 - 6.3)
Dominican Republic (2002)	2.8	(1.2 - 5.6)	0.0	(0.0 - 0.0)	35.8	(29.7 - 42.0)	6.2	(3.6 - 9.8)	5.1	(2.8 - 8.5)	10.9	(7.3 - 15.3)
Honduras (2011/12)	8.6	(5.7 - 12.2)	0.1	(0.0 - 1.7)	55.7	(49.7 - 61.2)	8.2	(5.4 - 11.8)	2.9	(1.4 - 5.4)	3.9	(2.1 - 6.6)
Nicaragua (1998)	8.3	(6.0 - 11.2)	0.4	(0.1 - 1.5)	51.5	(46.6 - 56.1)	3.2	(1.8 - 5.2)	7.7	(5.4 - 10.5)	8.7	(6.3 - 11.6)
Peru (1991/92)	12.9	(9.4 - 17.0)	1.3	(0.4 - 3.1)	38.9	(33.4 - 44.5)	26.0	(21.2 - 31.2)	1.3	(0.4 - 3.1)	7.3	(4.7 - 10.6)
Peru (1996)	13.8	(11.5 - 16.2)	1.0	(0.5 - 1.9)	46.6	(43.2 - 49.9)	18.5	(15.9 - 21.2)	2.4	(1.5 - 3.6)	5.6	(4.2 - 7.3)
Peru (2000)	10.2	(7.7 - 13.1)	0.4	(0.1 - 1.4)	49.1	(44.5 - 53.4)	13.5	(10.6 - 16.7)	5.1	(3.3 - 7.3)	6.3	(4.3 - 8.7)
Peru (2004/6)	9.0	(5.2 - 14.0)	1.4	(0.3 - 4.3)	58.4	(50.3 - 65.6)	13.0	(8.3 - 18.7)	0.0	(0.0 - 0.0)	7.9	(4.4 - 12.8)
Peru (2007/8)	15.3	(10.3 - 21.2)	0.0	(0.0 - 0.0)	58.3	(50.4 - 65.4)	10.9	(6.8 - 16.2)	0.0	(0.0 - 0.0)	2.7	(0.9 - 6.0)
Peru (2009)	7.1	(3.6 - 12.2)	0.9	(0.1 - 3.9)	68.1	(59.7 - 75.2)	13.7	(8.6 - 20.0)	0.1	(0.0 - 5.3)	2.8	(0.9 - 6.7)

CI= Confidence Interval

S11.3 Table: Status at 3 months following method related discontinuation

Injectables

	At risk		Switched to: LARC		SARC		Traditional		Permanent		Became pregnant	
	Rate	95%CI	Rate	95%CI	Rate	95%CI	Rate	95%CI	Rate	95%CI	Rate	95%CI
**Sub-Saharan Africa**												
Angola (2015/16)	5.1	(2.2 - 9.6)	0.0	(0.0 - 0.0)	7.0	(3.6 - 12.1)	2.2	(0.6 - 5.7)	0.0	(0.0 - 0.0)	7.5	(3.9 - 12.7)
Benin (2017/18)	9.4	(5.5 - 14.6)	5.4	(2.6 - 9.7)	6.3	(3.2 - 10.9)	4.7	(2.1 - 8.9)	0.0	(0.0 - 0.0)	4.8	(2.2 - 9.0)
Burkina Faso (2010)	10.5	(7.0 - 14.9)	2.9	(1.3 - 5.7)	13.9	(9.8 - 18.7)	3.4	(1.6 - 6.3)	0.0	(0.0 - 0.0)	5.7	(3.2 - 9.2)
Burkina Faso (2021)	4.2	(2.6 - 6.4)	10.8	(8.1 - 14.0)	11.0	(8.2 - 14.2)	2.6	(1.4 - 4.4)	0.0	(0.0 - 0.0)	6.2	(4.1 - 8.7)
Burundi (2010/11)	11.5	(8.3 - 15.4)	9.3	(6.4 - 12.9)	14.1	(10.5 - 18.2)	0.9	(0.2 - 2.5)	0.0	(0.0 - 0.0)	7.8	(5.2 - 11.2)
Côte d'Ivoire (2021)	4.7	(2.4 - 8.1)	8.1	(4.9 - 12.2)	10.4	(6.8 - 14.8)	1.6	(0.5 - 4.0)	0.0	(0.0 - 0.0)	8.2	(5.0 - 12.3)
Ethiopia (2005)	8.0	(5.3 - 11.4)	1.7	(0.7 - 3.7)	27.4	(22.5 - 32.5)	1.5	(0.6 - 3.5)	0.0	(0.0 - 0.0)	12.6	(9.1 - 16.6)
Ethiopia (2016)	9.0	(7.3 - 11.0)	18.6	(16.1 - 21.2)	8.4	(6.7 - 10.4)	1.4	(0.8 - 2.3)	0.0	(0.0 - 0.6)	9.3	(7.5 - 11.3)
Gambia (2019/20)	7.7	(5.3 - 10.8)	3.8	(2.2 - 6.1)	12.3	(9.2 - 15.9)	0.8	(0.2 - 2.3)	0.0	(0.0 - 0.0)	7.9	(5.4 - 11.0)
Ghana (2014)	11.3	(7.4 - 16.1)	5.4	(2.8 - 9.1)	9.9	(6.3 - 14.5)	5.1	(2.7 - 8.8)	0.0	(0.0 - 0.0)	7.9	(4.7 - 12.2)
Ghana (2022/23)	5.8	(4.3 - 7.6)	3.3	(2.2 - 4.8)	14.9	(12.5 - 17.5)	3.0	(2.0 - 4.4)	0.0	(0.0 - 0.0)	9.8	(7.9 - 12.0)
Guinea (2018)	2.8	(1.2 - 5.3)	10.4	(7.1 - 14.5)	13.3	(9.5 - 17.8)	2.2	(0.9 - 4.7)	0.0	(0.0 - 0.0)	5.7	(3.3 - 9.0)
Kenya (1998)	7.8	(4.6 - 12.2)	1.2	(0.3 - 3.7)	22.4	(16.9 - 28.5)	2.8	(1.1 - 5.9)	1.8	(0.6 - 4.5)	6.8	(3.9 - 10.9)
Kenya (2003)	8.2	(5.6 - 11.3)	2.6	(1.3 - 4.6)	22.8	(18.6 - 27.2)	4.3	(2.5 - 6.7)	0.0	(0.0 - 0.0)	11.0	(8.1 - 14.5)
Kenya (2014)	10.0	(8.3 - 11.8)	20.0	(17.7 - 22.5)	24.1	(21.6 - 26.6)	4.8	(3.7 - 6.2)	0.5	(0.2 - 1.0)	7.4	(6.0 - 9.1)
Kenya (2022)	9.7	(8.1 - 11.4)	17.7	(15.6 - 19.8)	16.3	(14.3 - 18.4)	2.7	(1.9 - 3.7)	0.0	(0.0 - 0.0)	8.4	(6.9 - 10.0)
Lesotho (2014)	17.7	(14.3 - 21.5)	3.1	(1.7 - 5.1)	39.4	(34.8 - 44.0)	0.9	(0.3 - 2.2)	0.0	(0.0 - 0.0)	9.2	(6.7 - 12.2)
Liberia (2013)	15.6	(12.1 - 19.5)	0.9	(0.3 - 2.3)	6.5	(4.3 - 9.4)	0.3	(0.0 - 1.5)	0.0	(0.0 - 0.0)	8.2	(5.7 - 11.3)
Liberia (2019/20)	7.8	(6.0 - 9.8)	1.1	(0.5 - 2.0)	5.7	(4.2 - 7.5)	1.8	(1.0 - 3.0)	0.0	(0.0 - 0.0)	10.1	(8.1 - 12.4)
Madagascar (2021)	9.4	(7.8 - 11.1)	13.7	(11.8 - 15.7)	15.4	(13.4 - 17.5)	6.2	(5.0 - 7.7)	0.1	(0.0 - 0.5)	4.0	(3.0 - 5.3)
Malawi (2004/5)	7.5	(5.8 - 9.4)	0.1	(0.0 - 0.7)	5.8	(4.3 - 7.6)	2.3	(1.4 - 3.5)	0.8	(0.4 - 1.7)	11.1	(9.0 - 13.4)
Malawi (2015/16)	4.9	(4.1 - 5.7)	9.8	(8.7 - 11.0)	9.9	(8.8 - 11.1)	1.7	(1.2 - 2.2)	1.9	(1.4 - 2.5)	10.5	(9.4 - 11.8)
Mali (2012/13)	4.3	(1.6 - 9.3)	3.0	(0.9 - 7.5)	14.4	(8.7 - 21.6)	0.0	(0.0 - 0.0)	0.0	(0.0 - 0.0)	4.0	(1.4 - 8.9)
Mali (2018)	4.2	(2.2 - 7.1)	2.4	(1.0 - 4.8)	5.5	(3.2 - 8.8)	0.0	(0.0 - 0.0)	0.0	(0.0 - 0.0)	9.1	(6.0 - 13.0)
Mozambique (2011)	3.4	(1.5 - 6.6)	0.0	(0.0 - 0.0)	15.2	(10.7 - 20.4)	0.2	(0.0 - 2.2)	0.0	(0.0 - 0.0)	10.8	(7.1 - 15.5)
Mozambique (2022/23)	3.6	(2.0 - 6.1)	3.4	(1.8 - 5.7)	14.0	(10.6 - 18.0)	2.0	(0.9 - 4.0)	0.0	(0.0 - 0.0)	7.1	(4.7 - 10.2)
Namibia (2013)	10.0	(7.8 - 12.7)	0.1	(0.0 - 0.9)	23.6	(20.2 - 27.1)	0.2	(0.0 - 1.0)	0.0	(0.0 - 0.0)	5.7	(4.0 - 7.8)
Niger (2012)	7.4	(3.3 - 13.8)	0.0	(0.0 - 0.0)	33.2	(24.0 - 42.7)	0.0	(0.0 - 0.0)	0.0	(0.0 - 0.0)	3.9	(1.2 - 9.2)
Nigeria (2013)	10.4	(6.6 - 15.2)	3.3	(1.4 - 6.5)	11.4	(7.4 - 16.3)	8.5	(5.1 - 13.0)	0.0	(0.0 - 0.0)	3.2	(1.4 - 6.5)
Nigeria (2018)	6.1	(4.4 - 8.3)	5.4	(3.8 - 7.5)	7.4	(5.5 - 9.8)	2.3	(1.3 - 3.7)	0.1	(0.0 - 0.8)	8.7	(6.6 - 11.2)
Rwanda (2010/11)	9.8	(7.8 - 12.0)	19.1	(16.5 - 22.0)	24.5	(21.5 - 27.5)	6.2	(4.7 - 8.1)	0.2	(0.0 - 0.7)	7.4	(5.7 - 9.4)
Rwanda (2014/15)	9.3	(7.6 - 11.2)	14.0	(12.0 - 16.3)	23.9	(21.3 - 26.6)	5.6	(4.3 - 7.2)	0.1	(0.0 - 0.6)	11.7	(9.8 - 13.8)
Rwanda (2019/20)	5.2	(3.8 - 6.8)	22.2	(19.4 - 25.1)	16.1	(13.7 - 18.7)	5.8	(4.4 - 7.5)	0.1	(0.0 - 0.7)	11.8	(9.7 - 14.1)

Senegal (2010/11)	8.4	(5.7 - 11.7)	0.7	(0.2 - 2.2)	22.8	(18.4 - 27.4)	1.1	(0.3 - 2.7)	0.0	(0.0 - 0.0)	9.6	(6.7 - 13.1)
Senegal (2015)	7.2	(4.2 - 11.4)	8.9	(5.4 - 13.3)	19.7	(14.5 - 25.4)	3.0	(1.2 - 6.0)	0.0	(0.0 - 0.0)	10.5	(6.8 - 15.3)
Senegal (2016)	4.7	(2.6 - 7.7)	14.8	(10.8 - 19.3)	24.9	(19.9 - 30.2)	4.3	(2.3 - 7.2)	0.0	(0.0 - 0.0)	7.4	(4.7 - 10.9)
Senegal (2018)	6.6	(4.2 - 9.7)	11.7	(8.4 - 15.5)	14.9	(11.2 - 19.0)	4.2	(2.4 - 6.8)	0.0	(0.0 - 0.0)	12.6	(9.2 - 16.5)
Senegal (2019)	10.0	(6.9 - 13.7)	7.4	(4.8 - 10.7)	11.7	(8.3 - 15.6)	1.6	(0.6 - 3.5)	0.0	(0.0 - 0.0)	9.9	(6.8 - 13.6)
Senegal (2023)	5.4	(3.5 - 7.9)	6.5	(4.4 - 9.3)	11.0	(8.1 - 14.3)	1.4	(0.6 - 3.0)	0.0	(0.0 - 0.0)	6.5	(4.3 - 9.2)
Sierra Leone (2013)	10.1	(7.6 - 13.1)	3.0	(1.7 - 4.8)	17.7	(14.4 - 21.4)	0.9	(0.3 - 2.1)	0.0	(0.0 - 0.0)	8.6	(6.3 - 11.4)
South Africa (2016)	7.4	(5.1 - 10.2)	5.7	(3.7 - 8.2)	18.5	(14.9 - 22.5)	0.0	(0.0 - 0.0)	0.0	(0.0 - 0.0)	12.0	(9.0 - 15.4)
Tanzania (2004/5)	7.3	(5.2 - 9.8)	0.4	(0.1 - 1.4)	24.3	(20.6 - 28.2)	5.7	(3.9 - 8.0)	0.2	(0.0 - 1.1)	5.9	(4.0 - 8.2)
Tanzania (2015/16)	9.1	(7.2 - 11.3)	11.5	(9.3 - 13.9)	13.4	(11.1 - 15.9)	3.5	(2.3 - 4.9)	0.9	(0.4 - 1.7)	5.4	(4.0 - 7.2)
Tanzania (2022)	5.4	(4.0 - 7.2)	11.9	(9.7 - 14.3)	7.3	(5.6 - 9.3)	3.5	(2.4 - 5.0)	0.0	(0.0 - 0.0)	13.1	(10.8 - 15.6)
Uganda (2011)	12.0	(9.7 - 14.7)	2.2	(1.3 - 3.6)	12.0	(9.7 - 14.7)	2.3	(1.4 - 3.7)	0.3	(0.1 - 1.0)	13.8	(11.2 - 16.5)
Zambia (2013/14)	11.3	(9.2 - 13.6)	5.1	(3.7 - 6.8)	19.8	(17.1 - 22.6)	2.6	(1.7 - 3.9)	0.1	(0.0 - 0.7)	12.2	(10.1 - 14.6)
Zambia (2018/19)	7.9	(6.4 - 9.6)	8.8	(7.2 - 10.6)	14.3	(12.3 - 16.5)	1.5	(0.9 - 2.3)	0.0	(0.0 - 0.0)	12.0	(10.1 - 14.0)
Zimbabwe (1999)	16.3	(11.6 - 21.6)	0.9	(0.2 - 3.1)	36.1	(29.6 - 42.6)	0.0	(0.0 - 0.0)	0.2	(0.0 - 2.2)	5.3	(2.8 - 9.0)
Zimbabwe (2005/6)	14.5	(10.3 - 19.4)	2.0	(0.7 - 4.4)	41.3	(34.9 - 47.6)	0.9	(0.2 - 3.0)	0.3	(0.0 - 2.1)	7.3	(4.4 - 11.1)
Zimbabwe (2010/11)	14.2	(10.3 - 18.8)	6.4	(3.8 - 9.8)	33.5	(27.8 - 39.2)	1.4	(0.4 - 3.4)	0.0	(0.0 - 0.0)	9.4	(6.2 - 13.3)
Zimbabwe (2015)	10.8	(7.9 - 14.3)	18.3	(14.5 - 22.4)	37.4	(32.5 - 42.3)	0.0	(0.0 - 0.0)	0.3	(0.0 - 1.5)	7.9	(5.4 - 10.9)
**North Africa Western Asia and Europe**												
Egypt (1995/96)	13.3	(9.0 - 18.5)	21.1	(15.7 - 27.0)	27.7	(21.7 - 34.1)	1.3	(0.3 - 3.8)	0.0	(0.0 - 0.0)	3.4	(1.5 - 6.6)
Egypt (2000)	13.6	(11.1 - 16.5)	21.0	(17.8 - 24.3)	24.5	(21.2 - 27.9)	0.7	(0.2 - 1.6)	0.0	(0.0 - 0.0)	5.3	(3.7 - 7.3)
Egypt (2003)	13.9	(10.9 - 17.3)	24.2	(20.4 - 28.3)	24.6	(20.7 - 28.6)	1.4	(0.6 - 2.8)	0.0	(0.0 - 0.0)	6.1	(4.1 - 8.5)
Egypt (2005)	11.6	(9.7 - 13.7)	20.7	(18.3 - 23.2)	27.2	(24.5 - 29.9)	0.8	(0.4 - 1.5)	0.1	(0.0 - 0.5)	5.0	(3.8 - 6.5)
Egypt (2008)	12.6	(10.0 - 15.5)	15.7	(12.8 - 18.8)	30.1	(26.3 - 33.9)	0.4	(0.1 - 1.2)	0.0	(0.0 - 0.0)	3.4	(2.1 - 5.2)
Egypt (2014)	14.6	(12.0 - 17.3)	9.6	(7.5 - 11.9)	29.4	(26.0 - 32.9)	6.3	(4.7 - 8.3)	0.3	(0.1 - 1.0)	4.7	(3.3 - 6.4)
Jordan (2007)	5.7	(2.2 - 11.7)	9.7	(4.8 - 16.7)	16.5	(9.8 - 24.7)	20.5	(13.0 - 29.1)	2.1	(0.4 - 6.8)	8.0	(3.6 - 14.6)
Jordan (2009)	8.7	(5.0 - 13.7)	13.7	(9.0 - 19.5)	31.8	(24.8 - 39.0)	13.2	(8.5 - 18.9)	0.0	(0.0 - 0.0)	11.4	(7.1 - 16.9)
Jordan (2012)	13.7	(8.2 - 20.5)	10.4	(5.7 - 16.7)	35.9	(27.3 - 44.4)	12.3	(7.2 - 18.9)	0.0	(0.0 - 0.0)	8.4	(4.3 - 14.2)
Morocco (2003/4)	11.9	(7.9 - 16.8)	0.6	(0.1 - 2.7)	57.0	(49.9 - 63.5)	6.9	(4.0 - 11.0)	0.5	(0.1 - 2.6)	3.0	(1.3 - 6.1)
Türkiye (2003/4)	5.3	(2.2 - 10.4)	12.6	(7.4 - 19.3)	33.9	(25.5 - 42.5)	28.9	(21.0 - 37.3)	0.0	(0.0 - 0.0)	5.9	(2.6 - 11.2)
Yemen (2013)	4.9	(3.2 - 7.1)	6.9	(4.9 - 9.3)	28.9	(25.0 - 33.0)	4.6	(3.0 - 6.7)	0.2	(0.0 - 1.1)	12.1	(9.4 - 15.1)
**Central, South & Southeast Asia**												
Bangladesh (1993/94)	12.1	(9.1 - 15.5)	3.2	(1.8 - 5.2)	41.6	(36.8 - 46.4)	7.0	(4.8 - 9.8)	0.7	(0.2 - 2.0)	5.4	(3.5 - 7.9)
Bangladesh (1996/97)	11.4	(8.7 - 14.6)	3.3	(1.9 - 5.2)	46.1	(41.5 - 50.6)	5.9	(4.0 - 8.4)	0.0	(0.0 - 0.0)	6.1	(4.1 - 8.5)
Bangladesh (1999/0)	10.7	(8.4 - 13.3)	1.9	(1.0 - 3.2)	47.2	(43.2 - 51.0)	7.6	(5.7 - 9.9)	0.6	(0.2 - 1.4)	5.7	(4.1 - 7.7)
Bangladesh (2004)	9.2	(7.3 - 11.3)	1.7	(1.0 - 2.8)	60.2	(56.7 - 63.6)	8.6	(6.8 - 10.7)	0.2	(0.0 - 0.8)	5.3	(3.9 - 7.1)
Bangladesh (2011)	13.1	(11.0 - 15.3)	2.9	(1.9 - 4.1)	50.7	(47.4 - 53.9)	8.5	(6.8 - 10.4)	1.7	(1.0 - 2.7)	2.1	(1.3 - 3.1)
Bangladesh (2014)	14.7	(11.9 - 17.7)	5.9	(4.2 - 8.1)	51.3	(47.1 - 55.4)	7.3	(5.3 - 9.6)	1.7	(0.9 - 3.1)	3.7	(2.3 - 5.4)
Bangladesh (2017/18)	13.1	(11.0 - 15.2)	5.9	(4.5 - 7.4)	57.0	(53.9 - 60.0)	10.1	(8.4 - 12.1)	0.8	(0.4 - 1.5)	2.2	(1.4 - 3.2)
Bangladesh (2022)	12.1	(9.5 - 14.9)	3.7	(2.3 - 5.5)	50.7	(46.5 - 54.8)	8.2	(6.1 - 10.6)	0.1	(0.0 - 0.9)	2.7	(1.6 - 4.3)
Cambodia (2010/11)	12.4	(9.2 - 16.1)	3.4	(1.9 - 5.7)	25.7	(21.2 - 30.4)	7.1	(4.7 - 10.1)	1.4	(0.5 - 3.1)	6.2	(4.0 - 9.0)

Cambodia (2014)	8.0	(5.7 - 10.7)	7.0	(4.9 - 9.6)	32.3	(28.0 - 36.6)	11.8	(9.0 - 15.0)	0.3	(0.0 - 1.3)	3.0	(1.7 - 4.9)
Cambodia (2021/22)	7.5	(5.0 - 10.5)	3.9	(2.2 - 6.3)	33.5	(28.6 - 38.4)	9.2	(6.5 - 12.5)	0.2	(0.0 - 1.4)	6.2	(4.0 - 9.0)
India (2015/16)	9.0	(6.6 - 11.9)	0.8	(0.3 - 2.0)	20.4	(16.8 - 24.2)	7.9	(5.6 - 10.6)	5.0	(3.2 - 7.3)	8.4	(6.1 - 11.2)
India (2019/21)	3.7	(2.8 - 4.8)	1.6	(1.1 - 2.4)	27.3	(25.0 - 29.7)	8.2	(6.8 - 9.7)	3.4	(2.5 - 4.4)	5.5	(4.4 - 6.7)
Indonesia (1991)	6.5	(5.3 - 8.0)	11.2	(9.5 - 13.0)	53.2	(50.4 - 56.0)	2.0	(1.3 - 2.9)	0.5	(0.2 - 1.1)	3.3	(2.4 - 4.4)
Indonesia (1994)	8.6	(7.3 - 10.0)	8.3	(7.1 - 9.7)	56.4	(54.0 - 58.7)	2.0	(1.4 - 2.7)	0.7	(0.4 - 1.2)	2.7	(2.0 - 3.6)
Indonesia (1997)	12.4	(10.9 - 13.9)	10.0	(8.7 - 11.5)	54.5	(52.2 - 56.8)	2.6	(1.9 - 3.4)	0.3	(0.1 - 0.6)	2.2	(1.6 - 3.0)
Indonesia (2002/3)	12.7	(11.2 - 14.4)	7.9	(6.6 - 9.2)	51.4	(48.9 - 53.8)	4.6	(3.7 - 5.7)	0.3	(0.1 - 0.7)	2.3	(1.7 - 3.1)
Indonesia (2007)	10.6	(9.4 - 11.8)	7.0	(6.1 - 8.1)	57.0	(55.0 - 58.9)	4.7	(3.9 - 5.6)	0.3	(0.1 - 0.6)	3.2	(2.6 - 4.0)
Indonesia (2012)	11.6	(10.4 - 12.8)	10.2	(9.1 - 11.4)	54.0	(52.1 - 55.8)	4.7	(3.9 - 5.5)	0.2	(0.1 - 0.5)	1.9	(1.4 - 2.4)
Indonesia (2017)	10.0	(9.0 - 11.0)	11.5	(10.5 - 12.6)	51.6	(50.0 - 53.3)	7.1	(6.3 - 8.0)	0.3	(0.1 - 0.5)	1.3	(1.0 - 1.7)
Myanmar (2015/16)	9.7	(8.0 - 11.5)	3.8	(2.7 - 5.0)	45.1	(42.1 - 48.0)	0.6	(0.3 - 1.3)	0.3	(0.1 - 0.8)	1.9	(1.2 - 2.8)
Nepal (2011)	6.7	(4.9 - 9.0)	4.0	(2.6 - 5.8)	24.3	(20.9 - 27.8)	10.7	(8.3 - 13.3)	3.6	(2.3 - 5.4)	6.2	(4.4 - 8.3)
Nepal (2016)	8.1	(5.9 - 10.7)	10.3	(7.9 - 13.2)	18.3	(15.0 - 21.7)	14.6	(11.7 - 17.8)	2.2	(1.1 - 3.7)	6.1	(4.2 - 8.4)
Nepal (2022)	8.2	(6.2 - 10.5)	12.7	(10.2 - 15.5)	17.0	(14.1 - 20.0)	17.7	(14.8 - 20.8)	2.3	(1.3 - 3.7)	4.7	(3.2 - 6.6)
Pakistan (2012/13)	6.0	(4.1 - 8.4)	1.8	(0.9 - 3.3)	20.1	(16.6 - 23.9)	12.5	(9.7 - 15.7)	0.0	(0.0 - 2.3)	5.7	(3.8 - 8.1)
Pakistan (2017/18)	4.5	(2.4 - 7.7)	1.9	(0.7 - 4.3)	18.1	(13.5 - 23.3)	6.6	(3.9 - 10.3)	0.9	(0.2 - 2.9)	11.5	(7.8 - 15.9)
Philippines (1998)	9.8	(6.0 - 14.6)	1.9	(0.6 - 4.7)	31.1	(24.6 - 37.8)	16.7	(11.8 - 22.4)	0.5	(0.0 - 2.7)	2.9	(1.2 - 6.2)
Philippines (2003)	7.3	(4.8 - 10.5)	0.9	(0.2 - 2.5)	48.0	(42.4 - 53.4)	10.3	(7.3 - 14.0)	0.3	(0.0 - 1.7)	5.2	(3.1 - 8.1)
Philippines (2022)	9.7	(7.4 - 12.4)	5.3	(3.6 - 7.4)	40.7	(36.5 - 44.8)	9.0	(6.8 - 11.6)	0.0	(0.0 - 0.0)	3.8	(2.4 - 5.6)
**Latin America & Caribbean**												
Bolivia (1994)	5.8	(2.4 - 11.6)	8.3	(3.9 - 14.7)	30.9	(22.2 - 40.0)	27.1	(18.8 - 36.0)	0.0	(0.0 - 0.0)	8.0	(3.8 - 14.3)
Brazil (1996)	7.4	(3.8 - 12.6)	1.8	(0.4 - 5.2)	50.2	(41.5 - 58.2)	10.8	(6.3 - 16.7)	2.0	(0.5 - 5.4)	11.4	(6.8 - 17.4)
Colombia (1990)	7.4	(4.2 - 11.9)	8.2	(4.7 - 12.7)	52.1	(44.6 - 59.1)	13.3	(8.8 - 18.7)	1.3	(0.3 - 3.9)	9.5	(5.8 - 14.4)
Colombia (1995)	10.7	(7.8 - 14.1)	6.7	(4.4 - 9.6)	40.8	(35.7 - 45.8)	17.9	(14.1 - 22.0)	2.8	(1.4 - 4.9)	7.6	(5.1 - 10.6)
Colombia (2000)	9.7	(7.4 - 12.4)	7.5	(5.5 - 9.9)	44.0	(39.8 - 48.1)	16.5	(13.5 - 19.7)	2.4	(1.4 - 4.0)	6.3	(4.5 - 8.5)
Colombia (2005)	8.9	(7.7 - 10.1)	9.0	(7.9 - 10.3)	36.5	(34.4 - 38.5)	11.0	(9.7 - 12.3)	5.5	(4.6 - 6.6)	11.0	(9.8 - 12.4)
Colombia (2010)	8.3	(7.4 - 9.2)	14.3	(13.1 - 15.4)	34.0	(32.5 - 35.5)	8.3	(7.4 - 9.2)	6.6	(5.8 - 7.4)	6.7	(5.9 - 7.5)
Colombia (2015/16)	9.3	(8.4 - 10.3)	14.6	(13.5 - 15.8)	37.9	(36.3 - 39.5)	4.7	(4.1 - 5.5)	5.0	(4.3 - 5.7)	5.3	(4.6 - 6.0)
Dominican Republic (2002)	11.4	(9.0 - 14.2)	3.7	(2.4 - 5.5)	31.5	(27.7 - 35.3)	7.3	(5.3 - 9.6)	1.9	(1.0 - 3.3)	12.0	(9.5 - 14.8)
Guatemala (1998/99)	20.7	(15.7 - 26.2)	2.2	(0.8 - 4.8)	31.5	(25.5 - 37.6)	8.3	(5.2 - 12.4)	7.7	(4.7 - 11.6)	4.5	(2.3 - 7.8)
Guatemala (2014/15)	10.2	(8.7 - 11.7)	7.8	(6.6 - 9.2)	17.8	(15.9 - 19.7)	13.3	(11.7 - 15.0)	2.7	(2.0 - 3.6)	10.4	(8.9 - 11.9)
Honduras (2011/12)	10.1	(9.0 - 11.3)	4.4	(3.7 - 5.3)	43.9	(42.0 - 45.8)	12.9	(11.7 - 14.2)	0.6	(0.3 - 0.9)	8.3	(7.3 - 9.4)
Nicaragua (1998)	11.2	(8.3 - 14.5)	7.8	(5.4 - 10.7)	45.7	(40.6 - 50.5)	5.7	(3.7 - 8.3)	4.9	(3.0 - 7.4)	3.1	(1.7 - 5.2)
Paraguay (1990)	8.2	(5.6 - 11.4)	3.1	(1.7 - 5.3)	53.4	(48.0 - 58.5)	15.3	(11.8 - 19.3)	0.5	(0.1 - 1.9)	5.3	(3.3 - 8.0)
Peru (1991/92)	9.0	(6.4 - 12.1)	10.1	(7.4 - 13.4)	32.8	(28.2 - 37.5)	24.9	(20.8 - 29.3)	0.5	(0.1 - 1.7)	9.9	(7.2 - 13.1)
Peru (1996)	11.1	(9.2 - 13.1)	10.0	(8.3 - 11.9)	35.4	(32.5 - 38.3)	21.2	(18.7 - 23.7)	1.1	(0.6 - 1.8)	4.6	(3.5 - 6.0)
Peru (2000)	10.2	(9.0 - 11.5)	5.7	(4.8 - 6.8)	42.4	(40.3 - 44.4)	13.4	(12.0 - 14.9)	3.4	(2.7 - 4.2)	4.2	(3.4 - 5.1)
Peru (2004/6)	12.0	(10.6 - 13.5)	3.4	(2.6 - 4.3)	46.3	(44.0 - 48.5)	17.4	(15.8 - 19.2)	0.0	(0.0 - 0.3)	4.2	(3.3 - 5.1)
Peru (2007/8)	10.9	(9.8 - 12.1)	2.2	(1.7 - 2.8)	48.6	(46.8 - 50.4)	18.0	(16.6 - 19.4)	0.1	(0.0 - 0.2)	4.6	(3.9 - 5.4)
Peru (2009)	9.4	(8.4 - 10.5)	2.0	(1.5 - 2.5)	54.6	(52.9 - 56.4)	10.2	(9.2 - 11.3)	0.1	(0.0 - 0.3)	5.0	(4.2 - 5.8)

Peru (2010)	10.1	(9.1 - 11.2)	1.4	(1.0 - 1.9)	51.7	(50.0 - 53.5)	17.8	(16.5 - 19.1)	0.0	(0.0 - 0.2)	4.7	(4.0 - 5.5)
Peru (2011)	10.7	(9.6 - 11.9)	1.0	(0.7 - 1.4)	51.1	(49.3 - 52.9)	16.2	(14.9 - 17.6)	0.1	(0.0 - 0.3)	5.0	(4.3 - 5.8)
Peru (2012)	10.6	(9.6 - 11.7)	1.5	(1.1 - 2.0)	49.9	(48.3 - 51.6)	18.6	(17.3 - 19.9)	0.0	(0.0 - 0.0)	3.9	(3.3 - 4.6)

CI= Confidence Interval

S11.4 Table: Status at 3 months following method related discontinuation

Condom

	At risk		Switched to: LARC		SARC		Traditional		Permanent		Became pregnant	
	Rate	95%CI	Rate	95%CI	Rate	95%CI	Rate	95%CI	Rate	95%CI	Rate	95%CI
**Sub-Saharan Africa**												
Gabon (2019/21)	9.5	(5.4 - 14.9)	1.6	(0.4 - 4.8)	19.0	(13.0 - 25.8)	8.3	(4.5 - 13.5)	0.0	(0.0 - 0.0)	12.7	(7.9 - 18.7)
Kenya (2022)	9.6	(4.9 - 16.2)	15.4	(9.2 - 23.0)	27.9	(19.6 - 36.8)	6.4	(2.7 - 12.3)	0.7	(0.0 - 4.4)	7.2	(3.2 - 13.2)
Lesotho (2014)	33.1	(26.5 - 39.8)	1.2	(0.3 - 3.7)	50.1	(42.7 - 56.9)	0.1	(0.0 - 2.6)	0.0	(0.0 - 0.0)	10.3	(6.5 - 15.2)
Malawi (2015/16)	6.1	(3.9 - 9.1)	11.3	(8.2 - 15.0)	32.9	(27.9 - 38.0)	0.8	(0.2 - 2.3)	2.3	(1.1 - 4.4)	5.4	(3.3 - 8.2)
Namibia (2013)	13.2	(7.8 - 19.9)	0.7	(0.1 - 4.0)	62.2	(52.8 - 70.2)	0.0	(0.0 - 0.0)	0.6	(0.0 - 3.9)	8.8	(4.6 - 14.8)
Nigeria (2018)	11.0	(6.3 - 17.1)	6.4	(3.0 - 11.6)	17.3	(11.4 - 24.3)	10.9	(6.3 - 17.0)	0.0	(0.0 - 0.0)	8.4	(4.4 - 14.0)
Rwanda (2019/20)	8.4	(4.4 - 14.0)	23.3	(16.4 - 31.0)	33.6	(25.5 - 41.8)	7.2	(3.6 - 12.6)	0.0	(0.0 - 0.0)	11.1	(6.4 - 17.3)
South Africa (2016)	10.0	(5.6 - 16.0)	6.3	(3.0 - 11.5)	23.2	(16.3 - 30.8)	0.7	(0.1 - 3.8)	1.1	(0.1 - 4.4)	5.1	(2.2 - 10.0)
Zambia (2013/14)	12.5	(7.7 - 18.5)	12.1	(7.4 - 18.1)	60.3	(51.7 - 67.9)	2.5	(0.8 - 6.2)	0.0	(0.0 - 0.0)	8.6	(4.7 - 14.0)
Zambia (2018/19)	9.3	(5.5 - 14.3)	8.7	(5.0 - 13.5)	46.7	(39.0 - 54.0)	3.8	(1.6 - 7.5)	0.0	(0.0 - 0.0)	3.9	(1.7 - 7.6)
Zimbabwe (2015)	10.0	(4.9 - 17.2)	22.1	(14.2 - 31.2)	32.1	(22.7 - 41.8)	3.5	(1.0 - 8.9)	0.0	(0.0 - 0.0)	13.2	(7.2 - 21.0)
**North Africa Western Asia and Europe**												
Egypt (1992/93)	10.4	(5.5 - 17.1)	38.8	(29.6 - 47.9)	25.1	(17.3 - 33.6)	10.2	(5.4 - 16.8)	0.0	(0.0 - 0.0)	5.7	(2.4 - 11.3)
Egypt (1995/96)	18.5	(11.5 - 26.9)	38.4	(28.6 - 48.0)	24.4	(16.3 - 33.4)	4.5	(1.5 - 10.0)	0.0	(0.0 - 0.0)	7.8	(3.5 - 14.4)
Jordan (2002)	10.6	(5.8 - 17.0)	28.2	(20.4 - 36.6)	15.6	(9.7 - 22.8)	30.6	(22.4 - 39.1)	0.7	(0.0 - 4.1)	3.7	(1.2 - 8.3)
Jordan (2007)	6.8	(3.5 - 11.5)	15.6	(10.4 - 21.8)	16.0	(10.7 - 22.2)	40.6	(32.8 - 48.2)	0.0	(0.0 - 0.0)	13.2	(8.4 - 19.1)
Jordan (2009)	13.2	(9.3 - 17.8)	29.8	(24.1 - 35.6)	17.4	(12.9 - 22.4)	24.6	(19.4 - 30.2)	0.0	(0.0 - 0.0)	7.4	(4.6 - 11.2)
Jordan (2012)	5.2	(2.7 - 8.9)	34.8	(28.2 - 41.4)	24.7	(18.9 - 30.9)	24.0	(18.3 - 30.1)	0.0	(0.0 - 0.0)	2.9	(1.1 - 5.9)
Moldova (2005)	9.7	(5.7 - 15.0)	23.6	(17.3 - 30.6)	23.1	(16.8 - 30.0)	34.4	(27.0 - 41.9)	0.0	(0.0 - 0.0)	4.9	(2.2 - 9.1)
Türkiye (1993)	8.6	(4.6 - 14.2)	48.6	(39.8 - 56.8)	12.3	(7.3 - 18.5)	22.0	(15.3 - 29.4)	0.8	(0.1 - 3.9)	3.5	(1.2 - 7.7)
Türkiye (2003/4)	7.7	(4.6 - 11.8)	34.3	(28.0 - 40.7)	25.2	(19.6 - 31.2)	31.4	(25.3 - 37.7)	0.0	(0.0 - 0.0)	0.7	(0.1 - 2.7)
Ukraine (2007)	1.0	(0.1 - 3.8)	25.5	(18.7 - 32.8)	30.1	(22.9 - 37.6)	39.3	(31.4 - 47.1)	0.0	(0.0 - 0.0)	0.0	(0.0 - 0.0)
**Central, South & Southeast Asia**												
Bangladesh (1993/94)	6.7	(3.7 - 10.9)	5.5	(2.8 - 9.4)	51.4	(44.0 - 58.3)	19.9	(14.5 - 25.9)	0.7	(0.1 - 3.0)	3.2	(1.3 - 6.5)
Bangladesh (1996/97)	7.3	(4.3 - 11.3)	1.7	(0.6 - 4.2)	62.4	(55.5 - 68.5)	13.8	(9.6 - 18.8)	0.6	(0.1 - 2.6)	5.5	(3.0 - 9.2)
Bangladesh (1999/0)	8.2	(5.3 - 11.9)	1.2	(0.3 - 3.1)	65.8	(59.8 - 71.1)	13.0	(9.4 - 17.4)	0.0	(0.0 - 0.0)	3.8	(2.0 - 6.6)
Bangladesh (2004)	6.7	(4.4 - 9.5)	0.5	(0.1 - 1.8)	67.5	(62.5 - 72.1)	16.7	(13.1 - 20.7)	0.0	(0.0 - 0.0)	1.9	(0.8 - 3.7)
Bangladesh (2011)	12.7	(9.6 - 16.2)	3.8	(2.2 - 6.1)	68.4	(63.5 - 72.8)	8.0	(5.5 - 10.9)	0.8	(0.2 - 2.1)	1.6	(0.7 - 3.3)
Bangladesh (2014)	15.3	(11.3 - 19.9)	2.1	(0.9 - 4.4)	74.2	(68.5 - 79.0)	2.7	(1.2 - 5.1)	0.4	(0.1 - 2.0)	1.7	(0.6 - 3.8)
Bangladesh (2017/18)	12.5	(9.9 - 15.3)	2.4	(1.4 - 3.9)	69.9	(66.0 - 73.5)	10.5	(8.2 - 13.2)	0.0	(0.0 - 0.0)	1.2	(0.5 - 2.3)
Bangladesh (2022)	14.0	(10.4 - 18.3)	4.6	(2.6 - 7.5)	59.0	(53.1 - 64.4)	10.9	(7.6 - 14.7)	0.0	(0.0 - 2.6)	1.6	(0.6 - 3.6)
India (2005/6)	10.0	(8.4 - 11.8)	7.4	(6.0 - 9.0)	14.9	(13.0 - 17.1)	19.9	(17.7 - 22.2)	6.8	(5.5 - 8.4)	10.3	(8.6 - 12.1)
India (2015/16)	5.3	(4.8 - 5.9)	4.1	(3.6 - 4.6)	18.8	(17.8 - 19.8)	15.7	(14.8 - 16.6)	5.6	(5.0 - 6.1)	5.9	(5.3 - 6.5)

India (2019/21)	3.2	(3.0 - 3.5)	0.9	(0.8 - 1.1)	28.7	(28.1 - 29.4)	13.7	(13.2 - 14.3)	3.3	(3.1 - 3.6)	6.0	(5.6 - 6.3)
Indonesia (1991)	3.4	(1.1 - 7.9)	24.4	(17.1 - 32.5)	37.6	(29.0 - 46.3)	17.8	(11.5 - 25.1)	0.0	(0.0 - 0.0)	6.9	(3.2 - 12.4)
Indonesia (2012)	12.4	(7.4 - 18.6)	12.0	(7.2 - 18.3)	56.7	(47.7 - 64.6)	13.4	(8.2 - 19.8)	0.0	(0.0 - 0.0)	3.6	(1.3 - 7.9)
Indonesia (2017)	10.8	(6.8 - 15.7)	10.0	(6.2 - 14.8)	43.2	(36.0 - 50.2)	22.9	(17.2 - 29.2)	1.7	(0.5 - 4.5)	7.9	(4.6 - 12.3)
Kazakhstan (1999)	7.7	(4.4 - 12.2)	18.7	(13.4 - 24.6)	26.4	(20.3 - 33.0)	37.9	(30.9 - 44.9)	0.0	(0.0 - 0.0)	4.2	(1.9 - 7.8)
Maldives (2009)	9.3	(4.8 - 15.6)	5.4	(2.2 - 10.8)	12.8	(7.4 - 19.7)	15.1	(9.1 - 22.4)	0.0	(0.0 - 0.0)	11.5	(6.4 - 18.3)
Nepal (2011)	9.7	(5.3 - 15.7)	6.0	(2.7 - 11.2)	26.5	(19.1 - 34.6)	29.5	(21.7 - 37.7)	10.6	(5.9 - 16.7)	4.8	(1.9 - 9.6)
Nepal (2016)	13.5	(8.5 - 19.7)	11.6	(7.0 - 17.5)	31.6	(24.1 - 39.3)	16.1	(10.6 - 22.7)	4.6	(2.0 - 9.0)	3.8	(1.5 - 8.0)
Nepal (2022)	10.2	(5.5 - 16.6)	13.1	(7.7 - 20.0)	28.6	(20.6 - 37.1)	18.3	(11.8 - 25.9)	3.8	(1.3 - 8.5)	10.0	(5.4 - 16.4)
Pakistan (2012/13)	9.2	(5.9 - 13.4)	6.7	(3.9 - 10.5)	5.5	(3.0 - 9.1)	24.5	(19.1 - 30.3)	0.9	(0.2 - 3.0)	19.8	(14.9 - 25.3)
Philippines (1998)	7.8	(3.8 - 13.6)	9.0	(4.7 - 15.1)	15.8	(9.9 - 23.0)	39.7	(30.8 - 48.4)	0.0	(0.0 - 0.0)	11.4	(6.5 - 18.0)
Philippines (2003)	2.9	(1.0 - 6.8)	1.1	(0.2 - 4.2)	27.7	(20.5 - 35.3)	38.5	(30.3 - 46.5)	0.0	(0.0 - 0.0)	7.8	(4.1 - 13.0)
**Latin America & Caribbean**												
Brazil (1996)	9.6	(6.8 - 12.9)	2.2	(1.0 - 4.1)	58.5	(53.2 - 63.4)	15.9	(12.3 - 19.9)	2.7	(1.4 - 4.8)	3.4	(1.9 - 5.7)
Colombia (1995)	6.9	(4.5 - 9.9)	17.7	(13.8 - 21.9)	41.1	(35.9 - 46.3)	18.0	(14.1 - 22.3)	4.9	(3.0 - 7.6)	6.2	(3.9 - 9.1)
Colombia (2000)	8.6	(6.5 - 11.0)	10.5	(8.2 - 13.2)	45.2	(41.0 - 49.2)	20.3	(17.1 - 23.7)	4.3	(2.8 - 6.2)	7.8	(5.8 - 10.2)
Colombia (2005)	11.2	(9.8 - 12.6)	9.2	(7.9 - 10.5)	43.6	(41.3 - 45.8)	13.1	(11.6 - 14.7)	7.6	(6.4 - 8.8)	10.4	(9.0 - 11.8)
Colombia (2010)	10.1	(9.0 - 11.4)	10.9	(9.7 - 12.2)	42.1	(40.1 - 44.1)	8.9	(7.8 - 10.1)	7.3	(6.3 - 8.4)	12.1	(10.8 - 13.4)
Colombia (2015/16)	9.5	(8.0 - 11.2)	14.9	(13.0 - 16.9)	42.9	(40.2 - 45.6)	5.9	(4.7 - 7.3)	6.4	(5.2 - 7.9)	11.0	(9.4 - 12.8)
Dominican Republic (1996)	5.2	(2.6 - 9.3)	10.7	(6.6 - 15.8)	35.7	(28.6 - 42.8)	22.7	(16.7 - 29.1)	6.3	(3.3 - 10.6)	9.8	(6.0 - 14.8)
Dominican Republic (2002)	9.0	(5.9 - 12.8)	5.8	(3.4 - 9.1)	38.9	(33.0 - 44.7)	17.4	(13.1 - 22.2)	2.7	(1.2 - 5.2)	12.0	(8.5 - 16.3)
Guatemala (2014/15)	11.5	(7.6 - 16.2)	7.9	(4.7 - 12.0)	46.9	(40.0 - 53.5)	10.3	(6.6 - 14.9)	1.7	(0.5 - 4.2)	6.2	(3.5 - 10.0)
Honduras (2011/12)	10.0	(8.2 - 12.0)	3.6	(2.5 - 5.0)	42.0	(38.8 - 45.2)	19.3	(16.8 - 21.9)	1.5	(0.9 - 2.5)	12.7	(10.7 - 15.0)
Nicaragua (1998)	7.3	(4.0 - 11.9)	10.4	(6.4 - 15.6)	43.6	(36.0 - 51.0)	7.5	(4.1 - 12.1)	8.1	(4.6 - 12.9)	5.5	(2.7 - 9.7)
Peru (1991/92)	8.7	(5.8 - 12.5)	14.2	(10.4 - 18.7)	26.7	(21.6 - 32.1)	35.1	(29.4 - 40.8)	1.1	(0.3 - 3.0)	4.1	(2.2 - 6.9)
Peru (1996)	8.0	(5.8 - 10.6)	12.9	(10.1 - 16.1)	43.3	(38.8 - 47.7)	27.5	(23.5 - 31.5)	1.5	(0.7 - 2.9)	1.9	(0.9 - 3.4)
Peru (2000)	9.6	(7.4 - 12.1)	11.9	(9.5 - 14.6)	46.4	(42.5 - 50.3)	17.8	(14.9 - 20.9)	2.6	(1.5 - 4.0)	5.4	(3.8 - 7.4)
Peru (2004/6)	9.0	(7.0 - 11.4)	7.8	(5.9 - 10.1)	57.3	(53.4 - 61.1)	16.8	(14.1 - 19.8)	1.2	(0.6 - 2.3)	4.1	(2.8 - 5.9)
Peru (2007/8)	9.6	(7.9 - 11.5)	3.6	(2.6 - 4.9)	59.9	(56.9 - 62.7)	16.1	(14.0 - 18.4)	0.6	(0.3 - 1.2)	5.2	(3.9 - 6.6)
Peru (2009)	10.8	(9.2 - 12.5)	2.7	(1.9 - 3.6)	67.2	(64.6 - 69.7)	9.8	(8.2 - 11.4)	0.3	(0.1 - 0.8)	3.1	(2.2 - 4.1)
Peru (2010)	7.7	(6.4 - 9.3)	3.1	(2.3 - 4.2)	62.3	(59.6 - 64.9)	15.3	(13.4 - 17.3)	0.2	(0.0 - 0.6)	5.3	(4.2 - 6.7)
Peru (2011)	9.1	(7.6 - 10.7)	1.6	(1.0 - 2.4)	58.5	(55.8 - 61.1)	19.4	(17.3 - 21.6)	0.1	(0.0 - 0.4)	6.1	(4.9 - 7.5)
Peru (2012)	9.9	(8.4 - 11.5)	3.5	(2.7 - 4.6)	56.4	(53.8 - 59.0)	17.6	(15.7 - 19.6)	0.5	(0.2 - 1.0)	7.6	(6.3 - 9.1)

CI= Confidence Interval

S11.5 Table: Status at 3 months following method related discontinuation

Implant

	At risk		Switched to:								Became pregnant	
			LARC		SARC		Traditional		Permanent			
	Rate	95%CI	Rate	95%CI	Rate	95%CI	Rate	95%CI	Rate	95%CI	Rate	95%CI
**Sub-Saharan Africa**												
Benin (2017/18)	19.6	(13.2 - 26.8)	0.0	(0.0 - 0.0)	7.4	(3.7 - 12.8)	1.1	(0.1 - 4.3)	0.0	(0.0 - 0.0)	9.8	(5.4 - 15.7)
Burkina Faso (2021)	6.7	(4.4 - 9.5)	2.5	(1.2 - 4.4)	19.2	(15.4 - 23.4)	1.0	(0.3 - 2.5)	0.3	(0.0 - 1.5)	10.3	(7.5 - 13.6)
Côte d'Ivoire (2021)	7.0	(3.4 - 12.1)	0.9	(0.1 - 4.0)	12.2	(7.3 - 18.4)	0.8	(0.1 - 3.8)	0.0	(0.0 - 0.0)	5.2	(2.3 - 9.9)
Ethiopia (2016)	12.7	(8.0 - 18.4)	0.4	(0.0 - 3.0)	37.7	(30.1 - 45.2)	0.8	(0.1 - 3.5)	0.0	(0.0 - 0.0)	9.4	(5.5 - 14.6)
Gambia (2019/20)	9.1	(4.5 - 15.6)	0.6	(0.0 - 4.3)	8.0	(3.8 - 14.3)	2.0	(0.4 - 6.3)	0.0	(0.0 - 0.0)	11.8	(6.4 - 18.8)
Ghana (2022/23)	5.9	(3.8 - 8.7)	1.5	(0.6 - 3.1)	13.7	(10.4 - 17.5)	3.2	(1.8 - 5.4)	0.0	(0.0 - 0.0)	14.2	(10.8 - 18.0)
Guinea (2018)	0.9	(0.1 - 3.4)	1.8	(0.5 - 4.8)	28.5	(22.0 - 35.4)	0.5	(0.0 - 2.9)	0.0	(0.0 - 0.0)	8.5	(4.9 - 13.2)
Kenya (2014)	17.0	(11.7 - 23.1)	0.6	(0.1 - 3.1)	36.9	(29.6 - 44.2)	4.3	(1.9 - 8.2)	0.0	(0.0 - 0.0)	11.5	(7.2 - 16.9)
Kenya (2022)	7.7	(5.8 - 9.9)	6.8	(5.1 - 8.9)	28.6	(25.2 - 32.0)	2.7	(1.6 - 4.1)	0.3	(0.0 - 1.0)	11.8	(9.5 - 14.4)
Madagascar (2021)	10.6	(7.3 - 14.5)	1.5	(0.5 - 3.5)	29.7	(24.4 - 35.2)	4.9	(2.8 - 8.0)	0.0	(0.0 - 0.0)	7.7	(4.9 - 11.3)
Malawi (2015/16)	11.6	(8.2 - 15.5)	1.0	(0.3 - 2.7)	18.3	(14.2 - 22.9)	0.0	(0.0 - 0.0)	1.0	(0.3 - 2.7)	9.1	(6.2 - 12.7)
Mozambique (2022/23)	5.9	(2.6 - 11.0)	2.3	(0.6 - 6.2)	22.9	(15.9 - 30.7)	3.0	(0.9 - 7.2)	0.0	(0.0 - 0.0)	13.1	(7.9 - 19.7)
Nigeria (2018)	17.0	(11.6 - 23.3)	1.9	(0.5 - 5.1)	10.0	(6.0 - 15.3)	6.1	(3.1 - 10.6)	0.0	(0.0 - 0.0)	8.4	(4.8 - 13.4)
Rwanda (2019/20)	13.0	(9.6 - 17.1)	2.6	(1.2 - 4.8)	29.0	(24.1 - 34.1)	3.2	(1.7 - 5.6)	1.0	(0.3 - 2.7)	10.8	(7.7 - 14.6)
Senegal (2023)	6.7	(4.0 - 10.5)	3.2	(1.5 - 6.1)	22.1	(17.0 - 27.7)	1.6	(0.5 - 4.0)	0.0	(0.0 - 0.0)	17.8	(13.1 - 23.0)
Tanzania (2015/16)	13.7	(9.1 - 19.3)	0.7	(0.1 - 3.2)	23.3	(17.3 - 29.9)	1.9	(0.6 - 4.9)	0.7	(0.1 - 3.1)	10.4	(6.4 - 15.4)
Tanzania (2022)	10.4	(8.1 - 13.2)	4.8	(3.2 - 6.8)	10.9	(8.5 - 13.7)	1.5	(0.7 - 2.8)	0.0	(0.0 - 0.0)	13.0	(10.4 - 16.0)
Zambia (2013/14)	17.0	(10.8 - 24.3)	0.0	(0.0 - 0.0)	38.4	(29.6 - 47.1)	1.5	(0.3 - 5.2)	0.0	(0.0 - 0.0)	3.8	(1.3 - 8.4)
Zambia (2018/19)	10.9	(7.2 - 15.5)	0.4	(0.0 - 2.3)	34.1	(27.9 - 40.5)	0.5	(0.0 - 2.4)	0.7	(0.1 - 2.8)	11.5	(7.7 - 16.2)
Zimbabwe (2015)	19.0	(12.5 - 26.6)	1.9	(0.4 - 5.8)	35.0	(26.4 - 43.7)	0.0	(0.0 - 0.0)	0.6	(0.0 - 4.0)	13.1	(7.7 - 20.0)
**Central, South & Southeast Asia**												
Bangladesh (2017/18)	13.6	(8.8 - 19.5)	0.0	(0.0 - 0.0)	58.5	(50.3 - 65.8)	7.0	(3.7 - 11.7)	2.2	(0.6 - 5.4)	3.3	(1.3 - 7.1)
Indonesia (2002/3)	15.1	(9.4 - 22.0)	2.4	(0.6 - 6.4)	57.0	(47.7 - 65.3)	1.2	(0.2 - 4.6)	3.6	(1.3 - 8.1)	2.7	(0.8 - 6.8)
Indonesia (2007)	18.4	(11.9 - 26.1)	1.5	(0.3 - 5.3)	47.0	(37.5 - 55.9)	2.0	(0.4 - 6.1)	0.2	(0.0 - 4.0)	9.1	(4.7 - 15.3)
Indonesia (2012)	7.4	(4.1 - 12.0)	5.6	(2.8 - 9.8)	65.2	(57.5 - 71.8)	2.7	(1.0 - 6.0)	1.8	(0.5 - 4.8)	2.0	(0.6 - 5.0)
Indonesia (2017)	13.1	(9.7 - 17.1)	6.6	(4.3 - 9.7)	52.6	(47.0 - 57.9)	6.2	(3.9 - 9.2)	0.6	(0.1 - 2.1)	3.2	(1.7 - 5.6)
Nepal (2022)	15.4	(9.9 - 21.9)	1.0	(0.1 - 4.1)	21.8	(15.3 - 29.1)	17.3	(11.5 - 24.1)	3.1	(1.0 - 7.0)	9.2	(5.1 - 14.8)
Philippines (2022)	13.4	(8.9 - 18.8)	1.6	(0.4 - 4.4)	47.1	(39.7 - 54.2)	8.5	(5.0 - 13.2)	0.0	(0.0 - 0.0)	3.5	(1.5 - 6.9)
**Latin America & Caribbean**												
Colombia (2010)	20.4	(14.0 - 27.6)	3.1	(1.0 - 7.1)	39.9	(31.5 - 48.1)	3.2	(1.1 - 7.2)	6.9	(3.4 - 12.1)	6.8	(3.3 - 11.9)
Colombia (2015/16)	12.8	(9.5 - 16.6)	4.8	(2.9 - 7.5)	45.3	(39.9 - 50.5)	1.5	(0.6 - 3.3)	4.6	(2.7 - 7.2)	7.5	(5.0 - 10.7)

CI= Confidence Interval

S11.6 Table: Status at 3 months following method related discontinuation

Periodic abstinence

	At risk		**Switched to:**								Became pregnant	
			LARC		SARC		Traditional		Permanent			
	Rate	95%CI	Rate	95%CI	Rate	95%CI	Rate	95%CI	Rate	95%CI	Rate	95%CI
**Sub-Saharan Africa**												
Ghana (2022/23)	10.8	(6.2 - 16.8)	7.2	(3.6 - 12.5)	39.6	(31.2 - 47.9)	15.7	(10.1 - 22.5)	0.0	(0.0 - 0.0)	4.3	(1.7 - 8.8)
Madagascar (2021)	16.2	(10.3 - 23.3)	12.8	(7.6 - 19.4)	57.8	(48.5 - 66.0)	3.5	(1.2 - 7.9)	0.0	(0.0 - 0.0)	1.1	(0.1 - 4.5)
**North Africa Western Asia and Europe**												
Jordan (1997)	5.4	(2.4 - 10.1)	19.0	(12.9 - 26.0)	45.4	(36.9 - 53.6)	19.4	(13.2 - 26.4)	0.9	(0.1 - 4.0)	5.3	(2.3 - 9.9)
**Central, South & Southeast Asia**												
Bangladesh (1993/94)	11.5	(6.4 - 18.4)	7.5	(3.5 - 13.5)	62.4	(52.5 - 70.8)	11.6	(6.4 - 18.4)	0.0	(0.0 - 0.0)	0.0	(0.0 - 0.0)
Bangladesh (1999/0)	9.0	(4.9 - 14.7)	2.6	(0.8 - 6.5)	78.3	(70.1 - 84.5)	8.3	(4.4 - 13.9)	0.6	(0.0 - 3.7)	0.0	(0.0 - 0.0)
Bangladesh (2004)	14.6	(9.7 - 20.6)	4.4	(2.0 - 8.4)	67.7	(59.8 - 74.4)	9.3	(5.4 - 14.4)	2.2	(0.7 - 5.5)	1.5	(0.3 - 4.5)
Bangladesh (2011)	11.9	(7.2 - 17.8)	4.8	(2.1 - 9.2)	75.7	(67.7 - 82.0)	0.7	(0.1 - 3.5)	6.8	(3.4 - 11.8)	0.0	(0.0 - 0.0)
Bangladesh (2017/18)	14.6	(10.3 - 19.5)	6.0	(3.4 - 9.6)	74.8	(68.6 - 80.0)	1.4	(0.4 - 3.7)	1.5	(0.4 - 3.8)	0.0	(0.0 - 0.0)
India (2005/6)	12.5	(9.9 - 15.5)	5.0	(3.4 - 7.1)	32.3	(28.4 - 36.3)	13.1	(10.4 - 16.1)	20.3	(17.0 - 23.8)	7.5	(5.5 - 9.9)
India (2015/16)	6.1	(5.3 - 7.0)	2.3	(1.8 - 2.9)	28.2	(26.6 - 29.9)	16.2	(14.9 - 17.6)	7.7	(6.7 - 8.7)	4.5	(3.7 - 5.3)
India (2019/21)	4.1	(3.6 - 4.6)	0.8	(0.6 - 1.0)	20.7	(19.7 - 21.7)	24.9	(23.9 - 25.9)	5.4	(4.8 - 5.9)	6.1	(5.5 - 6.6)
**Latin America & Caribbean**												
Bolivia (1994)	9.3	(5.8 - 13.8)	21.2	(15.9 - 27.1)	51.0	(43.9 - 57.7)	6.4	(3.6 - 10.4)	0.3	(0.0 - 2.3)	1.9	(0.6 - 4.5)
Brazil (1996)	3.9	(1.5 - 8.2)	0.9	(0.1 - 4.0)	78.0	(70.0 - 84.1)	2.2	(0.6 - 5.9)	7.1	(3.6 - 12.3)	3.2	(1.1 - 7.2)
Colombia (1990)	14.4	(8.3 - 22.1)	15.8	(9.4 - 23.7)	59.7	(49.3 - 68.7)	1.2	(0.1 - 5.3)	3.5	(1.0 - 8.6)	4.0	(1.3 - 9.3)
Colombia (1995)	9.1	(6.3 - 12.6)	9.6	(6.6 - 13.1)	62.1	(56.5 - 67.2)	8.6	(5.8 - 12.0)	4.6	(2.7 - 7.4)	3.5	(1.9 - 6.0)
Colombia (2000)	7.9	(5.4 - 11.1)	10.1	(7.2 - 13.6)	64.0	(58.7 - 68.8)	9.7	(6.9 - 13.1)	3.5	(1.9 - 5.8)	1.9	(0.8 - 3.7)
Colombia (2005)	18.5	(15.5 - 21.8)	8.2	(6.1 - 10.6)	53.5	(49.3 - 57.5)	3.6	(2.3 - 5.3)	6.8	(5.0 - 9.1)	7.4	(5.5 - 9.8)
Colombia (2010)	10.4	(7.4 - 14.0)	8.3	(5.6 - 11.5)	51.6	(46.1 - 56.8)	9.0	(6.2 - 12.4)	6.2	(3.9 - 9.2)	6.4	(4.1 - 9.4)
Colombia (2015/16)	13.1	(7.7 - 19.9)	9.1	(4.7 - 15.1)	47.3	(38.0 - 56.0)	12.0	(6.9 - 18.6)	9.4	(4.9 - 15.5)	4.3	(1.6 - 9.1)
Dominican Republic (2002)	8.2	(4.6 - 13.1)	2.4	(0.8 - 5.7)	47.9	(40.0 - 55.4)	25.5	(19.0 - 32.5)	6.1	(3.1 - 10.5)	3.2	(1.2 - 6.8)
Honduras (2011/12)	7.2	(4.6 - 10.6)	5.3	(3.1 - 8.2)	48.0	(42.2 - 53.5)	8.6	(5.8 - 12.2)	2.1	(0.9 - 4.3)	24.9	(20.1 - 30.0)
Peru (1991/92)	12.7	(9.8 - 16.1)	21.6	(17.8 - 25.7)	57.3	(52.4 - 61.9)	6.0	(4.0 - 8.6)	0.1	(0.0 - 1.2)	0.8	(0.2 - 2.1)
Peru (1996)	11.6	(9.6 - 13.8)	15.8	(13.5 - 18.2)	59.5	(56.2 - 62.6)	5.4	(4.0 - 7.0)	3.0	(2.0 - 4.3)	1.4	(0.8 - 2.3)
Peru (2000)	12.1	(9.8 - 14.7)	9.4	(7.3 - 11.7)	65.6	(61.9 - 69.1)	3.0	(1.9 - 4.5)	3.4	(2.2 - 5.0)	2.7	(1.7 - 4.1)
Peru (2004/6)	13.1	(10.4 - 16.1)	5.3	(3.6 - 7.4)	76.7	(72.9 - 80.1)	1.8	(0.9 - 3.3)	0.0	(0.0 - 1.3)	0.7	(0.2 - 1.7)
Peru (2007/8)	11.9	(9.8 - 14.3)	2.1	(1.2 - 3.3)	77.2	(74.1 - 80.0)	1.6	(0.9 - 2.7)	0.6	(0.2 - 1.4)	5.8	(4.4 - 7.6)
Peru (2010)	13.5	(11.2 - 16.1)	2.5	(1.5 - 3.8)	74.6	(71.3 - 77.6)	2.3	(1.4 - 3.5)	0.1	(0.0 - 0.7)	5.8	(4.3 - 7.7)
Peru (2011)	13.7	(11.1 - 16.6)	1.1	(0.5 - 2.2)	74.8	(71.1 - 78.1)	2.1	(1.1 - 3.4)	0.1	(0.0 - 0.9)	7.5	(5.6 - 9.8)
Peru (2012)	12.3	(9.9 - 15.0)	1.0	(0.4 - 2.0)	75.5	(72.0 - 78.6)	2.4	(1.4 - 3.8)	0.0	(0.0 - 0.0)	6.2	(4.6 - 8.3)

CI= Confidence Interval

S11.7 Table: Status at 3 months following method related discontinuation

Withdrawal

	At risk		Switched to:								Became pregnant	
			LARC		SARC		Traditional		Permanent			
	Rate	95%CI	Rate	95%CI	Rate	95%CI	Rate	95%CI	Rate	95%CI	Rate	95%CI
**Sub-Saharan Africa**												
Zambia (2013/14)	13.5	(8.3 - 19.9)	10.1	(5.6 - 16.0)	71.9	(63.3 - 78.8)	1.4	(0.2 - 4.7)	0.0	(0.0 - 0.0)	0.0	(0.0 - 0.0)
Zambia (2018/19)	13.3	(7.6 - 20.6)	10.7	(5.7 - 17.6)	63.0	(52.9 - 71.6)	0.0	(0.0 - 0.0)	0.0	(0.0 - 0.0)	3.1	(0.9 - 7.9)
**North Africa Western Asia and Europe**												
Jordan (1997)	12.3	(7.7 - 17.9)	22.2	(16.1 - 28.9)	41.8	(34.1 - 49.4)	16.7	(11.4 - 22.9)	0.8	(0.1 - 3.4)	2.6	(0.9 - 6.0)
Jordan (2002)	7.6	(4.3 - 12.2)	36.0	(29.0 - 43.2)	35.3	(28.3 - 42.4)	6.8	(3.7 - 11.2)	1.3	(0.3 - 3.9)	3.1	(1.2 - 6.5)
Jordan (2007)	11.9	(7.9 - 16.7)	33.7	(27.3 - 40.3)	39.2	(32.4 - 45.8)	3.9	(1.9 - 7.3)	2.0	(0.6 - 4.7)	2.5	(1.0 - 5.5)
Jordan (2009)	6.2	(3.9 - 9.3)	35.3	(30.1 - 40.6)	45.8	(40.2 - 51.3)	9.5	(6.6 - 13.1)	0.0	(0.0 - 0.0)	1.0	(0.3 - 2.7)
Jordan (2012)	7.3	(5.0 - 10.2)	49.1	(44.1 - 53.9)	36.7	(31.9 - 41.4)	4.2	(2.5 - 6.5)	0.2	(0.0 - 1.3)	0.3	(0.0 - 1.4)
Jordan (2017/18)	12.7	(8.5 - 17.7)	42.1	(35.1 - 48.8)	18.3	(13.3 - 24.0)	3.0	(1.2 - 6.1)	0.0	(0.0 - 0.0)	9.6	(6.0 - 14.2)
Jordan (2023)	15.6	(10.7 - 21.2)	34.6	(27.7 - 41.6)	12.3	(8.0 - 17.6)	4.5	(2.1 - 8.2)	0.0	(0.0 - 0.0)	18.4	(13.2 - 24.4)
Moldova (2005)	7.2	(4.1 - 11.3)	44.1	(37.0 - 50.8)	36.5	(29.8 - 43.2)	5.0	(2.6 - 8.7)	1.5	(0.4 - 4.1)	3.6	(1.6 - 6.9)
Morocco (2003/4)	16.6	(10.9 - 23.3)	9.2	(5.1 - 14.8)	69.0	(60.5 - 76.1)	2.3	(0.6 - 6.0)	0.0	(0.0 - 0.0)	0.0	(0.0 - 0.0)
Türkiye (1993)	14.0	(10.2 - 18.4)	52.1	(46.0 - 57.7)	31.1	(25.8 - 36.6)	1.1	(0.3 - 3.0)	0.7	(0.1 - 2.3)	0.4	(0.1 - 2.0)
Türkiye (1998)	8.5	(5.3 - 12.6)	50.0	(43.2 - 56.4)	35.3	(29.0 - 41.6)	0.4	(0.0 - 2.2)	2.4	(0.9 - 5.1)	1.2	(0.3 - 3.4)
Türkiye (2003/4)	9.2	(6.7 - 12.2)	53.4	(48.5 - 58.0)	35.1	(30.5 - 39.6)	0.9	(0.3 - 2.3)	0.6	(0.2 - 1.8)	0.4	(0.1 - 1.5)
Ukraine (2007)	1.5	(0.3 - 4.9)	10.3	(5.9 - 16.2)	83.2	(75.7 - 88.6)	3.3	(1.2 - 7.4)	0.0	(0.0 - 0.0)	0.0	(0.0 - 0.0)
**Central, South & Southeast Asia**												
Bangladesh (2004)	5.9	(2.9 - 10.2)	0.0	(0.0 - 0.0)	78.6	(71.5 - 84.2)	15.0	(10.0 - 20.9)	0.0	(0.0 - 0.0)	0.0	(0.0 - 0.0)
Cambodia (2014)	14.3	(9.8 - 19.7)	16.0	(11.2 - 21.6)	56.6	(49.2 - 63.3)	1.6	(0.4 - 4.3)	1.4	(0.4 - 4.0)	7.2	(4.1 - 11.5)
Cambodia (2021/22)	10.2	(6.5 - 14.8)	12.8	(8.7 - 17.8)	48.1	(41.2 - 54.7)	4.2	(2.0 - 7.5)	0.0	(0.0 - 0.0)	5.4	(2.9 - 9.0)
India (2005/6)	11.0	(7.8 - 14.7)	9.3	(6.4 - 12.9)	35.6	(30.4 - 40.9)	21.7	(17.4 - 26.5)	10.9	(7.7 - 14.6)	2.6	(1.2 - 4.8)
India (2015/16)	6.3	(5.4 - 7.3)	2.3	(1.8 - 3.0)	30.3	(28.6 - 32.1)	20.0	(18.5 - 21.5)	6.7	(5.8 - 7.7)	3.9	(3.2 - 4.7)
India (2019/21)	5.6	(5.0 - 6.2)	1.2	(0.9 - 1.5)	30.1	(28.9 - 31.4)	18.6	(17.5 - 19.7)	5.3	(4.7 - 5.9)	5.7	(5.1 - 6.4)
Indonesia (2007)	15.6	(8.9 - 24.1)	3.3	(0.8 - 8.7)	52.1	(41.1 - 62.1)	3.5	(0.9 - 9.0)	0.0	(0.0 - 0.0)	22.5	(14.3 - 31.8)
Indonesia (2017)	11.7	(7.6 - 16.8)	14.3	(9.8 - 19.8)	67.5	(60.2 - 73.7)	0.0	(0.0 - 0.0)	1.1	(0.2 - 3.6)	3.7	(1.6 - 7.1)
Nepal (2016)	20.4	(13.8 - 27.9)	11.0	(6.2 - 17.2)	51.3	(42.2 - 59.7)	0.0	(0.0 - 0.0)	8.0	(4.1 - 13.6)	0.9	(0.1 - 4.2)
Nepal (2022)	12.8	(8.0 - 18.8)	21.8	(15.5 - 28.8)	54.4	(46.0 - 62.1)	1.2	(0.2 - 4.3)	2.9	(1.0 - 6.7)	0.8	(0.1 - 3.6)
Philippines (1993)	13.0	(7.4 - 20.2)	5.3	(2.0 - 10.7)	36.4	(27.2 - 45.5)	18.6	(11.8 - 26.6)	1.4	(0.2 - 5.4)	12.9	(7.3 - 20.1)
Philippines (1998)	7.6	(4.3 - 12.0)	5.4	(2.8 - 9.4)	45.2	(37.8 - 52.3)	16.7	(11.7 - 22.5)	0.9	(0.1 - 3.3)	6.3	(3.4 - 10.5)
Philippines (2003)	9.5	(5.7 - 14.5)	2.9	(1.1 - 6.3)	50.4	(42.6 - 57.6)	17.1	(11.8 - 23.1)	1.0	(0.2 - 3.7)	8.5	(4.9 - 13.4)
Philippines (2022)	12.1	(8.8 - 15.9)	2.6	(1.3 - 4.8)	45.2	(39.8 - 50.5)	7.9	(5.3 - 11.1)	0.2	(0.0 - 1.4)	10.0	(7.0 - 13.5)

Vietnam (2002)	8.0	(4.1 - 13.5)	49.7	(40.7 - 58.0)	31.1	(23.3 - 39.2)	6.9	(3.4 - 12.2)	2.6	(0.7 - 6.5)	1.7	(0.4 - 5.3)
**Latin America & Caribbean**												
Brazil (1996)	9.1	(5.6 - 13.6)	0.8	(0.1 - 3.0)	74.6	(68.0 - 80.1)	3.7	(1.7 - 7.0)	5.6	(2.9 - 9.3)	2.4	(0.9 - 5.3)
Colombia (1995)	10.1	(7.0 - 13.8)	10.8	(7.6 - 14.6)	54.6	(48.8 - 60.0)	13.1	(9.6 - 17.1)	4.8	(2.8 - 7.6)	3.7	(2.0 - 6.2)
Colombia (2000)	7.6	(5.3 - 10.5)	12.6	(9.6 - 16.1)	60.0	(55.1 - 64.6)	12.1	(9.1 - 15.4)	4.5	(2.8 - 6.8)	1.2	(0.4 - 2.6)
Colombia (2005)	9.2	(7.4 - 11.2)	11.4	(9.4 - 13.6)	59.4	(56.1 - 62.6)	5.0	(3.7 - 6.6)	9.3	(7.5 - 11.3)	3.0	(2.0 - 4.3)
Colombia (2010)	13.6	(11.2 - 16.2)	8.3	(6.4 - 10.5)	57.2	(53.5 - 60.7)	4.0	(2.8 - 5.7)	5.2	(3.7 - 7.0)	5.8	(4.2 - 7.7)
Colombia (2015/16)	7.9	(5.1 - 11.4)	12.5	(9.0 - 16.6)	67.4	(61.6 - 72.6)	2.7	(1.3 - 5.1)	5.7	(3.4 - 8.9)	1.6	(0.6 - 3.7)
Dominican Republic (1996)	9.4	(4.9 - 15.8)	9.1	(4.7 - 15.4)	57.5	(47.7 - 66.1)	14.6	(8.8 - 21.9)	2.0	(0.4 - 6.1)	1.3	(0.2 - 5.0)
Dominican Republic (2002)	5.5	(3.3 - 8.6)	4.1	(2.2 - 6.9)	60.6	(54.6 - 66.1)	15.1	(11.2 - 19.5)	4.4	(2.4 - 7.2)	4.0	(2.2 - 6.8)
Guatemala (2014/15)	23.4	(16.1 - 31.5)	5.0	(2.0 - 10.1)	61.7	(52.2 - 69.9)	2.3	(0.6 - 6.4)	4.4	(1.7 - 9.3)	0.0	(0.0 - 0.0)
Honduras (2011/12)	10.3	(8.2 - 12.6)	2.2	(1.4 - 3.5)	58.4	(54.8 - 61.9)	4.1	(2.8 - 5.7)	1.8	(1.0 - 2.9)	17.2	(14.5 - 20.0)
Peru (1991/92)	6.2	(3.1 - 10.8)	11.8	(7.3 - 17.5)	49.5	(41.3 - 57.1)	29.5	(22.5 - 36.8)	0.0	(0.0 - 0.0)	0.5	(0.0 - 3.1)
Peru (1996)	9.8	(6.8 - 13.5)	12.6	(9.1 - 16.6)	52.0	(46.2 - 57.4)	22.2	(17.7 - 27.0)	1.1	(0.3 - 2.8)	0.8	(0.2 - 2.4)
Peru (2000)	10.5	(6.8 - 15.1)	8.1	(4.9 - 12.2)	57.7	(50.7 - 64.0)	16.4	(11.8 - 21.7)	1.2	(0.3 - 3.5)	0.0	(0.0 - 0.0)
Peru (2004/6)	15.6	(11.5 - 20.3)	3.0	(1.4 - 5.6)	54.5	(48.3 - 60.3)	25.2	(20.1 - 30.6)	0.6	(0.1 - 2.2)	1.0	(0.2 - 2.8)
Peru (2007/8)	8.7	(6.3 - 11.7)	2.2	(1.1 - 4.0)	73.2	(68.7 - 77.1)	13.9	(10.8 - 17.4)	0.0	(0.0 - 0.0)	0.7	(0.2 - 2.0)
Peru (2009)	14.0	(11.6 - 16.6)	3.1	(2.0 - 4.5)	79.8	(76.7 - 82.5)	0.1	(0.0 - 0.7)	0.4	(0.1 - 1.1)	0.5	(0.1 - 1.2)
Peru (2010)	15.1	(12.3 - 18.1)	1.4	(0.7 - 2.6)	65.4	(61.4 - 69.1)	15.7	(12.9 - 18.7)	1.6	(0.8 - 2.8)	0.3	(0.1 - 1.1)
Peru (2011)	10.2	(7.8 - 13.1)	2.1	(1.1 - 3.7)	69.9	(65.6 - 73.7)	15.2	(12.2 - 18.4)	0.0	(0.0 - 1.7)	1.6	(0.7 - 3.0)
Peru (2012)	12.7	(10.3 - 15.4)	2.2	(1.2 - 3.5)	68.7	(64.9 - 72.1)	14.6	(12.0 - 17.5)	0.7	(0.2 - 1.6)	0.4	(0.1 - 1.2)

CI= Confidence Interval
