# Supplementary material for: Contraceptive discontinuation, switching, abandonment and their reproductive consequences: An analysis of 1,539,071 episodes of reversible method use contributed from 61 countries that participated in DHS: Population base-analysis
Source: PLOS Glob Public Health. 2025 Oct 31;5(10):e0005174. doi: 10.1371/journal.pgph.0005174 (PMC12578211; doi:10.1371/journal.pgph.0005174)
Supplement: S12 Table — (PDF) [file pgph.0005174.s023.pdf]

S12 Table: Trends in switching to any method at 3 months following method-related discontinuations

Contraceptive method	Declining trends		Increasing trends		No of countries
	Significant	Not significant	Not significant	Significant	
Oral contraceptive		8	2	8	18
IUD	1	2	1	3	7
Injectables	4		6	4	14
Condom	2	3	2		7
Implants			1		1
Periodic abstinence	3		1		4
Withdrawal	4	1	1		6
**Total**	14	14	14	15	57
