# Supplementary material for: Contraceptive discontinuation, switching, abandonment and their reproductive consequences: An analysis of 1,539,071 episodes of reversible method use contributed from 61 countries that participated in DHS: Population base-analysis
Source: PLOS Glob Public Health. 2025 Oct 31;5(10):e0005174. doi: 10.1371/journal.pgph.0005174 (PMC12578211; doi:10.1371/journal.pgph.0005174)
Supplement: S13 Table — (PDF) [file pgph.0005174.s024.pdf]

S13 Table: Medians of 12-month abandonment following method-related discontinuation, by method

Contraceptive method	Method-related discontinuation	Abandoned after method-related discontinuation		Percentage abandoned
		Yes	No	
Oral contraceptives	20.6	11.9	8.4	57.3
IUD	8.8	4.1	4.5	56.3
Injectables	24.4	16.0	8.0	71.1
Condom	11.5	4.6	6.1	45.9
Implants	8.6	5.7	2.5	69.4
Periodic abstinence	6.2	1.5	4.1	21.7
Withdrawal	9.8	2.3	6.4	21.7
