# Supplementary material for: Contraceptive discontinuation, switching, abandonment and their reproductive consequences: An analysis of 1,539,071 episodes of reversible method use contributed from 61 countries that participated in DHS: Population base-analysis
Source: PLOS Glob Public Health. 2025 Oct 31;5(10):e0005174. doi: 10.1371/journal.pgph.0005174 (PMC12578211; doi:10.1371/journal.pgph.0005174)
Supplement: S14 Table — (PDF) [file pgph.0005174.s025.pdf]

S14.1 Table: 12-month cumulative incidence of abandonment following method-related discontinuation per 100 episodes of use

Oral contraceptives (OCs)

	Method-related		Abandoned after method-related discontinuation				Percentage abandoned
			Yes		No		
	Rate	95%CI	Rate	95%CI	Rate	95%CI	
**Sub-Saharan Africa**							
Angola (2015/16)	20.2	(16.8 - 23.9)	17.1	(13.9 - 20.6)	3.1	(1.9 - 4.8)	84.5
Benin (2017/18)	19.5	(16.1 - 23.2)	15.6	(12.4 - 19.0)	4.0	(2.5 - 6.0)	79.7
Burkina Faso (2010)	10.1	(7.9 - 12.5)	8.2	(6.3 - 10.5)	1.9	(1.0 - 3.1)	81.6
Burkina Faso (2021)	15.8	(13.3 - 18.6)	11.8	(9.6 - 14.3)	4.0	(2.7 - 5.6)	74.8
Burundi (2010/11)	28.0	(22.9 - 33.4)	12.6	(9.0 - 16.8)	15.4	(11.5 - 19.8)	45.1
Comoros (2012)	5.6	(2.3 - 11.0)	4.1	(1.5 - 9.1)	1.4	(0.2 - 5.3)	74.7
Côte d'Ivoire (2021)	19.2	(16.7 - 21.9)	16.0	(13.7 - 18.6)	3.2	(2.2 - 4.5)	83.4
Ethiopia (2005)	35.2	(31.5 - 38.9)	16.7	(13.9 - 19.7)	18.5	(15.6 - 21.6)	47.4
Ethiopia (2016)	41.7	(37.2 - 46.2)	19.0	(15.6 - 22.8)	22.7	(19.0 - 26.6)	45.7
Gabon (2019/21)	24.4	(20.2 - 28.8)	18.5	(14.8 - 22.5)	5.9	(3.9 - 8.5)	75.8
Gambia (2013)	15.3	(11.1 - 20.1)	10.6	(7.2 - 14.8)	4.7	(2.5 - 7.9)	69.1
Gambia (2019/20)	26.1	(21.6 - 30.8)	20.6	(16.6 - 25.0)	5.4	(3.4 - 8.2)	79.1
Ghana (2014)	12.4	(9.6 - 15.5)	11.2	(8.6 - 14.2)	1.2	(0.5 - 2.4)	90.3
Ghana (2022/23)	23.6	(20.9 - 26.5)	17.8	(15.3 - 20.4)	5.9	(4.5 - 7.5)	75.2
Guinea (2018)	38.6	(34.5 - 42.6)	28.9	(25.2 - 32.7)	9.7	(7.4 - 12.4)	74.8
Kenya (1998)	22.7	(20.0 - 25.6)	13.8	(11.6 - 16.2)	8.9	(7.2 - 10.9)	60.7
Kenya (2003)	32.4	(29.4 - 35.5)	19.7	(17.2 - 22.4)	12.7	(10.6 - 15.0)	60.8
Kenya (2014)	26.3	(24.1 - 28.5)	9.2	(7.9 - 10.7)	17.1	(15.2 - 19.0)	35.1
Kenya (2022)	33.5	(31.0 - 36.0)	16.0	(14.1 - 18.0)	17.5	(15.5 - 19.5)	47.8
Lesotho (2014)	17.4	(14.7 - 20.2)	8.6	(6.7 - 10.8)	8.8	(6.9 - 11.0)	49.3
Liberia (2013)	20.8	(17.5 - 24.3)	18.9	(15.7 - 22.3)	1.9	(1.0 - 3.3)	91.0
Liberia (2019/20)	33.0	(28.4 - 37.5)	25.9	(21.8 - 30.2)	7.1	(4.9 - 9.8)	78.5
Madagascar (2021)	31.6	(28.9 - 34.3)	11.4	(9.6 - 13.3)	20.2	(17.9 - 22.6)	36.0
Malawi (2004/5)	28.3	(24.1 - 32.6)	18.7	(15.1 - 22.5)	9.6	(7.1 - 12.6)	66.0
Malawi (2015/16)	37.5	(34.3 - 40.7)	23.3	(20.6 - 26.2)	14.2	(12.0 - 16.6)	62.2
Mali (2012/13)	15.4	(12.3 - 18.9)	10.6	(8.0 - 13.6)	4.8	(3.1 - 7.0)	68.9
Mali (2018)	16.0	(12.7 - 19.6)	11.9	(9.1 - 15.2)	4.1	(2.5 - 6.2)	74.6
Mozambique (2011)	19.0	(16.3 - 21.7)	16.7	(14.2 - 19.4)	2.3	(1.4 - 3.4)	88.1
Mozambique (2022/23)	15.5	(12.9 - 18.3)	10.3	(8.2 - 12.7)	5.2	(3.7 - 7.0)	66.5
Namibia (2013)	16.9	(13.8 - 20.4)	8.9	(6.6 - 11.6)	8.0	(5.9 - 10.7)	52.5
Niger (2012)	8.2	(6.8 - 9.9)	5.3	(4.2 - 6.7)	2.9	(2.1 - 4.0)	64.8
Nigeria (2013)	6.1	(4.6 - 7.7)	4.6	(3.4 - 6.0)	1.5	(0.9 - 2.4)	75.2
Nigeria (2018)	24.1	(21.5 - 26.8)	17.0	(14.7 - 19.4)	7.1	(5.6 - 8.8)	70.6

Rwanda (2010/11)	33.8	(30.7 - 36.8)	12.4	(10.4 - 14.7)	21.3	(18.7 - 24.0)	36.8
Rwanda (2014/15)	28.8	(26.2 - 31.4)	9.0	(7.4 - 10.7)	19.8	(17.6 - 22.1)	31.1
Rwanda (2019/20)	36.6	(33.8 - 39.4)	13.5	(11.6 - 15.5)	23.1	(20.7 - 25.6)	36.9
Senegal (2010/11)	22.3	(19.5 - 25.1)	16.0	(13.6 - 18.6)	6.3	(4.8 - 8.0)	71.8
Senegal (2015)	21.0	(17.7 - 24.5)	13.3	(10.7 - 16.3)	7.7	(5.7 - 10.0)	63.6
Senegal (2016)	13.7	(11.2 - 16.6)	9.5	(7.3 - 11.9)	4.3	(2.9 - 6.0)	68.9
Senegal (2018)	16.6	(13.5 - 19.9)	11.0	(8.5 - 13.9)	5.5	(3.8 - 7.8)	66.6
Senegal (2019)	15.0	(11.8 - 18.6)	9.3	(6.7 - 12.3)	5.7	(3.8 - 8.2)	61.9
Senegal (2023)	17.7	(15.0 - 20.6)	14.7	(12.2 - 17.4)	3.0	(1.9 - 4.4)	83.2
Sierra Leone (2013)	15.1	(13.0 - 17.4)	9.7	(8.0 - 11.6)	5.4	(4.1 - 6.9)	64.2
South Africa (2016)	17.3	(13.7 - 21.3)	14.7	(11.4 - 18.5)	2.6	(1.4 - 4.4)	85.1
Tanzania (2004/5)	24.8	(22.3 - 27.4)	14.7	(12.7 - 16.9)	10.1	(8.4 - 11.9)	59.3
Tanzania (2015/16)	20.3	(17.8 - 23.0)	12.7	(10.6 - 14.9)	7.7	(6.1 - 9.5)	62.2
Tanzania (2022)	26.4	(22.5 - 30.5)	19.9	(16.4 - 23.7)	6.5	(4.5 - 8.9)	75.4
Uganda (2011)	30.1	(26.0 - 34.3)	17.5	(14.2 - 21.0)	12.6	(9.8 - 15.8)	58.1
Zambia (2013/14)	16.6	(15.2 - 18.2)	9.6	(8.5 - 10.8)	7.1	(6.1 - 8.1)	57.6
Zambia (2018/19)	29.5	(27.1 - 31.9)	19.5	(17.4 - 21.7)	10.0	(8.5 - 11.6)	66.2
Zimbabwe (1994)	6.4	(5.5 - 7.5)	4.6	(3.8 - 5.5)	1.8	(1.3 - 2.4)	71.9
Zimbabwe (1999)	8.5	(7.3 - 9.7)	4.7	(3.9 - 5.7)	3.7	(2.9 - 4.6)	56.1
Zimbabwe (2005/6)	6.3	(5.5 - 7.2)	3.8	(3.2 - 4.5)	2.5	(2.0 - 3.1)	60.3
Zimbabwe (2010/11)	8.5	(7.5 - 9.5)	5.0	(4.3 - 5.8)	3.5	(2.9 - 4.2)	58.9
Zimbabwe (2015)	10.2	(9.3 - 11.2)	5.1	(4.4 - 5.8)	5.1	(4.4 - 5.8)	50.0
**North Africa Western Asia and Europe**							
Azerbaijan (2006)	18.5	(12.0 - 26.0)	12.9	(7.6 - 19.6)	5.5	(2.4 - 10.7)	70.0
Egypt (1992/93)	21.8	(20.2 - 23.5)	12.5	(11.2 - 13.9)	9.3	(8.2 - 10.6)	57.2
Egypt (1995/96)	23.5	(21.9 - 25.1)	12.7	(11.5 - 14.0)	10.7	(9.6 - 11.9)	54.2
Egypt (2000)	25.5	(23.7 - 27.3)	11.3	(10.1 - 12.7)	14.2	(12.8 - 15.7)	44.3
Egypt (2003)	23.7	(21.5 - 25.9)	8.3	(7.0 - 9.8)	15.4	(13.6 - 17.3)	35.2
Egypt (2005)	25.7	(24.3 - 27.1)	10.1	(9.1 - 11.1)	15.6	(14.4 - 16.8)	39.3
Egypt (2008)	17.6	(16.1 - 19.0)	7.8	(6.8 - 8.9)	9.8	(8.7 - 10.9)	44.4
Egypt (2014)	16.1	(15.1 - 17.1)	7.0	(6.4 - 7.7)	9.1	(8.3 - 9.8)	43.7
Jordan (1990)	33.9	(31.1 - 36.7)	17.2	(15.0 - 19.4)	16.7	(14.6 - 19.0)	50.6
Jordan (1997)	40.2	(37.5 - 42.9)	13.1	(11.3 - 15.0)	27.1	(24.7 - 29.6)	32.6
Jordan (2002)	32.3	(29.5 - 35.1)	12.1	(10.2 - 14.1)	20.2	(17.9 - 22.6)	37.5
Jordan (2007)	20.7	(18.9 - 22.5)	7.9	(6.7 - 9.1)	12.8	(11.4 - 14.3)	38.1
Jordan (2009)	27.6	(25.6 - 29.6)	8.2	(7.1 - 9.5)	19.4	(17.6 - 21.1)	29.8
Jordan (2012)	25.7	(23.9 - 27.6)	6.8	(5.8 - 7.9)	19.0	(17.4 - 20.6)	26.3
Jordan (2017/18)	16.4	(14.7 - 18.2)	9.1	(7.8 - 10.6)	7.3	(6.1 - 8.6)	55.6
Jordan (2023)	18.1	(16.1 - 20.3)	9.4	(7.9 - 11.1)	8.7	(7.3 - 10.3)	51.8
Moldova (2005)	22.0	(18.0 - 26.2)	4.7	(2.9 - 7.1)	17.3	(13.7 - 21.2)	21.3
Morocco (1992)	12.0	(10.8 - 13.3)	6.9	(6.0 - 7.9)	5.1	(4.3 - 6.0)	57.3

Morocco (2003/4)	9.7	(8.9 - 10.5)	2.5	(2.1 - 2.9)	7.2	(6.5 - 7.9)	26.0
Türkiye (1993)	27.1	(23.9 - 30.4)	8.6	(6.7 - 10.8)	18.5	(15.7 - 21.4)	31.9
Türkiye (1998)	25.6	(22.3 - 29.0)	6.4	(4.7 - 8.5)	19.2	(16.2 - 22.3)	25.0
Türkiye (2003/4)	33.1	(29.9 - 36.3)	8.5	(6.7 - 10.5)	24.6	(21.7 - 27.6)	25.6
Türkiye (2018/19)	26.8	(21.9 - 31.8)	11.9	(8.6 - 15.8)	14.9	(11.1 - 19.1)	44.5
Ukraine (2007)	16.9	(12.9 - 21.3)	3.1	(1.6 - 5.4)	13.8	(10.2 - 17.9)	18.2
Yemen (2013)	22.0	(20.7 - 23.3)	13.2	(12.1 - 14.2)	8.8	(8.0 - 9.8)	59.8
**Central, South & Southeast Asia**							
Bangladesh (1993/94)	28.6	(27.0 - 30.2)	14.2	(13.0 - 15.5)	14.4	(13.1 - 15.6)	49.8
Bangladesh (1996/97)	26.9	(25.4 - 28.5)	11.5	(10.4 - 12.7)	15.4	(14.2 - 16.8)	42.7
Bangladesh (1999/0)	27.1	(25.7 - 28.5)	10.5	(9.6 - 11.5)	16.6	(15.4 - 17.8)	38.9
Bangladesh (2004)	25.0	(23.8 - 26.2)	7.4	(6.7 - 8.2)	17.5	(16.5 - 18.6)	29.8
Bangladesh (2011)	15.3	(14.4 - 16.2)	4.7	(4.2 - 5.3)	10.5	(9.8 - 11.3)	31.0
Bangladesh (2014)	13.2	(12.3 - 14.2)	3.6	(3.2 - 4.2)	9.6	(8.8 - 10.4)	27.6
Bangladesh (2017/18)	15.0	(14.2 - 15.8)	2.3	(2.0 - 2.7)	12.7	(11.9 - 13.5)	15.3
Bangladesh (2022)	11.8	(11.0 - 12.6)	3.4	(2.9 - 3.9)	8.4	(7.7 - 9.1)	28.7
Cambodia (2010/11)	16.5	(14.9 - 18.1)	8.9	(7.7 - 10.2)	7.6	(6.5 - 8.8)	53.9
Cambodia (2014)	14.9	(13.5 - 16.5)	7.7	(6.6 - 8.8)	7.3	(6.2 - 8.4)	51.4
Cambodia (2021/22)	12.3	(11.2 - 13.5)	6.7	(5.9 - 7.6)	5.6	(4.9 - 6.4)	54.4
India (2005/6)	26.7	(25.5 - 27.9)	15.1	(14.2 - 16.1)	11.6	(10.7 - 12.5)	56.7
India (2015/16)	18.6	(18.2 - 19.1)	11.3	(10.9 - 11.6)	7.4	(7.1 - 7.7)	60.4
India (2019/21)	22.4	(21.9 - 22.8)	12.2	(11.8 - 12.5)	10.2	(9.9 - 10.5)	54.5
Indonesia (1991)	13.8	(12.7 - 14.9)	7.8	(7.0 - 8.7)	6.0	(5.3 - 6.7)	56.6
Indonesia (1994)	14.5	(13.5 - 15.5)	7.2	(6.5 - 7.9)	7.3	(6.6 - 8.1)	49.5
Indonesia (1997)	16.4	(15.4 - 17.5)	5.3	(4.7 - 5.9)	11.2	(10.3 - 12.1)	32.0
Indonesia (2002/3)	14.7	(13.6 - 15.8)	4.9	(4.3 - 5.6)	9.8	(8.9 - 10.7)	33.2
Indonesia (2007)	21.8	(20.7 - 23.0)	6.9	(6.2 - 7.6)	15.0	(14.0 - 16.0)	31.5
Indonesia (2012)	19.6	(18.6 - 20.7)	4.7	(4.2 - 5.3)	14.9	(14.0 - 15.8)	24.2
Indonesia (2017)	25.1	(24.0 - 26.3)	5.5	(4.9 - 6.1)	19.6	(18.6 - 20.7)	21.9
Kazakhstan (1999)	40.9	(35.7 - 46.1)	10.7	(7.7 - 14.2)	30.2	(25.4 - 35.1)	26.2
Kyrgyz Republic (2012)	21.0	(14.9 - 27.8)	9.4	(5.4 - 14.7)	11.6	(7.1 - 17.2)	44.7
Maldives (2009)	16.1	(12.7 - 19.8)	9.1	(6.6 - 12.1)	7.0	(4.8 - 9.7)	56.5
Myanmar (2015/16)	16.1	(14.4 - 17.9)	6.0	(5.0 - 7.2)	10.1	(8.7 - 11.5)	37.5
Nepal (2011)	18.7	(16.5 - 21.1)	11.9	(10.1 - 13.9)	6.8	(5.4 - 8.4)	63.6
Nepal (2016)	15.3	(13.5 - 17.3)	7.5	(6.2 - 9.0)	7.8	(6.5 - 9.3)	48.9
Nepal (2022)	17.0	(15.0 - 19.2)	8.5	(7.1 - 10.2)	8.5	(7.0 - 10.2)	50.0
Pakistan (2012/13)	35.2	(31.1 - 39.4)	21.6	(18.2 - 25.2)	13.6	(10.8 - 16.8)	61.3
Pakistan (2017/18)	26.9	(22.5 - 31.5)	19.0	(15.3 - 23.2)	7.8	(5.4 - 10.9)	70.8
Philippines (1993)	17.2	(15.2 - 19.2)	11.7	(10.1 - 13.5)	5.4	(4.3 - 6.7)	68.4
Philippines (1998)	21.3	(19.2 - 23.5)	11.2	(9.6 - 12.8)	10.2	(8.7 - 11.8)	52.4
Philippines (2003)	22.7	(20.8 - 24.7)	12.7	(11.2 - 14.3)	10.0	(8.7 - 11.4)	56.0

Philippines (2022)	18.5	(17.2 - 19.9)	11.3	(10.3 - 12.5)	7.2	(6.3 - 8.1)	61.3
Tajikistan (2012)	9.8	(6.2 - 14.4)	4.0	(2.0 - 7.3)	5.8	(3.1 - 9.6)	41.2
Tajikistan (2017)	18.7	(13.5 - 24.5)	12.9	(8.6 - 18.2)	5.7	(3.1 - 9.5)	69.4
Vietnam (1997)	16.5	(12.4 - 21.1)	7.3	(4.6 - 10.7)	9.2	(6.2 - 12.9)	44.3
Vietnam (2002)	19.0	(15.6 - 22.7)	3.8	(2.3 - 5.8)	15.2	(12.1 - 18.6)	20.0
**Latin America & Caribbean**							
Bolivia (1994)	44.3	(39.9 - 48.5)	17.2	(14.1 - 20.6)	27.0	(23.3 - 30.9)	38.9
Brazil (1996)	16.8	(15.6 - 18.0)	8.3	(7.4 - 9.2)	8.5	(7.6 - 9.4)	49.5
Colombia (1990)	21.4	(19.4 - 23.5)	10.2	(8.7 - 11.7)	11.3	(9.7 - 12.9)	47.4
Colombia (1995)	30.8	(28.9 - 32.7)	11.4	(10.2 - 12.7)	19.3	(17.8 - 21.0)	37.1
Colombia (2000)	26.5	(24.6 - 28.3)	9.8	(8.6 - 11.1)	16.7	(15.1 - 18.2)	37.1
Colombia (2005)	29.4	(28.2 - 30.5)	12.7	(11.9 - 13.5)	16.7	(15.7 - 17.6)	43.2
Colombia (2010)	29.8	(28.7 - 31.0)	12.7	(11.9 - 13.5)	17.2	(16.2 - 18.1)	42.5
Colombia (2015/16)	26.3	(24.8 - 27.7)	11.2	(10.1 - 12.3)	15.1	(13.9 - 16.3)	42.5
Dominican Republic (1991)	28.6	(26.4 - 30.9)	22.7	(20.6 - 24.7)	6.0	(4.9 - 7.2)	79.1
Dominican Republic (1996)	27.9	(26.0 - 29.9)	19.5	(17.8 - 21.2)	8.4	(7.3 - 9.7)	69.8
Dominican Republic (2002)	26.0	(24.9 - 27.1)	17.6	(16.7 - 18.6)	8.4	(7.7 - 9.1)	67.9
Guatemala (1995)	26.3	(23.2 - 29.6)	19.0	(16.2 - 21.9)	7.3	(5.6 - 9.4)	72.1
Guatemala (1998/99)	30.8	(26.7 - 35.0)	18.6	(15.3 - 22.2)	12.2	(9.4 - 15.4)	60.4
Guatemala (2014/15)	20.9	(18.4 - 23.6)	11.1	(9.2 - 13.2)	9.8	(8.1 - 11.8)	53.0
Honduras (2011/12)	34.9	(33.5 - 36.4)	15.1	(14.0 - 16.2)	19.9	(18.7 - 21.0)	43.2
Nicaragua (1998)	24.2	(22.7 - 25.9)	13.3	(12.1 - 14.6)	10.9	(9.8 - 12.1)	55.0
Paraguay (1990)	36.2	(33.6 - 38.7)	8.5	(7.1 - 10.1)	27.6	(25.3 - 30.0)	23.6
Peru (1991/92)	34.7	(32.3 - 37.1)	11.8	(10.3 - 13.5)	22.9	(20.8 - 25.0)	34.0
Peru (1996)	34.9	(33.2 - 36.6)	10.3	(9.3 - 11.5)	24.6	(23.0 - 26.1)	29.6
Peru (2000)	41.4	(39.6 - 43.2)	13.4	(12.2 - 14.7)	28.0	(26.3 - 29.6)	32.5
Peru (2004/6)	47.3	(45.1 - 49.4)	13.8	(12.4 - 15.3)	33.4	(31.4 - 35.4)	29.3
Peru (2007/8)	46.8	(45.1 - 48.5)	13.7	(12.5 - 14.9)	33.1	(31.5 - 34.7)	29.2
Peru (2009)	48.5	(46.8 - 50.1)	11.6	(10.6 - 12.7)	36.9	(35.3 - 38.5)	23.9
Peru (2010)	48.3	(46.6 - 50.0)	11.2	(10.2 - 12.3)	37.1	(35.5 - 38.7)	23.2
Peru (2011)	46.4	(44.7 - 48.1)	9.3	(8.4 - 10.4)	37.0	(35.4 - 38.7)	20.1
Peru (2012)	46.0	(44.4 - 47.7)	10.3	(9.4 - 11.3)	35.7	(34.2 - 37.3)	22.4

CI= Confidence Interval

S14.2 Table: 12-month cumulative incidence of abandonment following method-related discontinuation per 100 episodes of use

IUD

	Method-related		Abandoned after method-related discontinuation				Percentage abandoned
			Yes		No		
	Rate	95%CI	Rate	95%CI	Rate	95%CI	
**Sub-Saharan Africa**							
Benin (2017/18)	3.90663	1.87598 - 7.10564	2.4	(0.9 - 5.2)	1.5	(0.5 - 3.9)	60.6
Burkina Faso (2021)	11.0328	7.52597 - 15.2842	7.8	(4.9 - 11.6)	3.2	(1.5 - 6.0)	70.8
Burundi (2010/11)	7.68677	3.452 - 14.1493	4.8	(1.5 - 10.9)	2.9	(0.9 - 6.9)	62.0
Ethiopia (2016)	8.69309	5.24858 - 13.2066	5.2	(2.7 - 8.9)	3.4	(1.4 - 6.9)	60.4
Guinea (2018)	66.9	(60.9 - 72.2)	45.9	(39.9 - 51.8)	21.0	(16.4 - 26.1)	68.6
Kenya (1998)	17.1	(10.6 - 24.8)	8.6	(4.2 - 14.9)	8.5	(4.2 - 14.7)	50.3
Kenya (2003)	8.2	(3.8 - 14.8)	5.0	(1.8 - 10.8)	3.2	(0.9 - 8.2)	61.0
Kenya (2014)	5.8	(3.4 - 9.1)	2.0	(0.8 - 4.2)	3.8	(1.9 - 6.7)	33.8
Kenya (2022)	8.0	(5.7 - 11.0)	3.5	(2.0 - 5.7)	4.5	(2.8 - 6.8)	43.8
Madagascar (2021)	12.8	(7.1 - 20.1)	6.8	(3.0 - 12.7)	6.0	(2.4 - 11.9)	53.1
Malawi (2015/16)	5.7	(2.9 - 9.9)	3.5	(1.5 - 7.0)	2.2	(0.7 - 5.5)	61.3
Mali (2018)	4.8	(1.8 - 10.2)	3.2	(0.9 - 8.1)	1.7	(0.3 - 5.4)	65.2
Mozambique (2022/23)	13.2	(8.2 - 19.5)	7.5	(3.9 - 12.7)	5.7	(2.6 - 10.5)	56.7
Nigeria (2013)	2.4	(1.0 - 4.9)	1.9	(0.7 - 4.2)	0.5	(0.1 - 2.2)	78.2
Nigeria (2018)	5.6	(3.1 - 9.1)	3.9	(1.9 - 7.1)	1.6	(0.5 - 4.0)	70.5
Rwanda (2014/15)	7.0	(3.1 - 13.3)	4.6	(1.6 - 10.0)	2.5	(0.6 - 7.2)	64.6
Rwanda (2019/20)	7.3	(4.0 - 11.9)	4.4	(2.0 - 8.2)	2.9	(1.0 - 6.5)	60.1
Senegal (2018)	5.8	(2.8 - 10.3)	4.4	(1.8 - 8.6)	1.5	(0.3 - 4.3)	74.9
Senegal (2019)	8.3	(4.3 - 14.0)	6.1	(2.7 - 11.4)	2.2	(0.6 - 5.8)	73.4
Senegal (2023)	11.7	(8.1 - 16.1)	9.3	(6.1 - 13.4)	2.4	(1.0 - 4.8)	79.8
Tanzania (2015/16)	11.0	(5.8 - 18.1)	7.6	(3.5 - 13.9)	3.4	(0.9 - 8.5)	69.4
Tanzania (2022)	13.0	(7.4 - 20.2)	8.7	(4.3 - 15.1)	4.2	(1.5 - 9.3)	67.3
Zambia (2013/14)	7.7	(4.1 - 13.0)	2.9	(1.0 - 6.6)	4.8	(2.1 - 9.4)	37.4
**North Africa Western Asia and Europe**							
Armenia (2000)	4.4	(2.4 - 7.4)	1.4	(0.4 - 3.5)	3.0	(1.4 - 5.6)	31.6
Armenia (2005)	5.7	(3.3 - 9.2)	2.0	(0.8 - 4.4)	3.7	(1.8 - 6.7)	35.2
Armenia (2010)	3.2	(1.3 - 6.3)	1.5	(0.4 - 4.1)	1.7	(0.5 - 4.2)	48.1
Armenia (2015/16)	0.4	(0.0 - 2.6)	0.0	(0.0 - 0.0)	0.4	(0.0 - 2.6)	0.0
Azerbaijan (2006)	3.2	(1.6 - 5.5)	2.9	(1.4 - 5.2)	0.3	(0.0 - 1.6)	91.1
Egypt (1992/93)	8.5	(7.5 - 9.6)	4.3	(3.5 - 5.1)	4.2	(3.5 - 5.1)	50.2
Egypt (1995/96)	9.1	(8.3 - 10.0)	4.3	(3.7 - 5.0)	4.8	(4.2 - 5.5)	47.5

Egypt (2000)	9.0	(8.3 - 9.9)	3.9	(3.3 - 4.4)	5.2	(4.6 - 5.8)	42.7
Egypt (2003)	8.6	(7.6 - 9.6)	3.6	(3.0 - 4.3)	5.0	(4.2 - 5.8)	42.0
Egypt (2005)	9.9	(9.1 - 10.6)	3.7	(3.3 - 4.2)	6.1	(5.6 - 6.7)	37.8
Egypt (2008)	6.8	(6.1 - 7.5)	3.0	(2.5 - 3.5)	3.8	(3.3 - 4.4)	43.5
Egypt (2014)	8.1	(7.4 - 8.9)	2.8	(2.4 - 3.3)	5.3	(4.7 - 5.9)	34.8
Jordan (1990)	12.1	(10.4 - 13.9)	6.9	(5.6 - 8.3)	5.3	(4.2 - 6.5)	56.6
Jordan (1997)	11.9	(10.4 - 13.6)	5.4	(4.3 - 6.6)	6.5	(5.4 - 7.9)	45.1
Jordan (2002)	8.9	(7.5 - 10.4)	3.8	(2.9 - 4.8)	5.1	(4.1 - 6.3)	42.5
Jordan (2007)	7.6	(6.6 - 8.8)	2.4	(1.9 - 3.1)	5.2	(4.4 - 6.1)	32.0
Jordan (2009)	9.1	(8.0 - 10.3)	2.9	(2.2 - 3.6)	6.3	(5.3 - 7.3)	31.2
Jordan (2012)	7.2	(6.2 - 8.3)	1.7	(1.2 - 2.3)	5.5	(4.7 - 6.5)	23.5
Jordan (2017/18)	5.4	(4.5 - 6.4)	2.6	(2.0 - 3.3)	2.8	(2.1 - 3.6)	48.0
Jordan (2023)	5.8	(4.7 - 7.1)	3.8	(2.9 - 4.9)	2.0	(1.4 - 2.8)	66.1
Moldova (2005)	4.3	(3.0 - 5.8)	1.5	(0.8 - 2.5)	2.8	(1.8 - 4.1)	34.5
Morocco (1992)	13.2	(9.2 - 17.9)	4.2	(2.2 - 7.3)	9.0	(5.7 - 13.1)	32.0
Morocco (2003/4)	12.1	(9.3 - 15.2)	1.6	(0.8 - 3.1)	10.4	(7.9 - 13.4)	13.6
Türkiye (1993)	6.8	(5.4 - 8.4)	2.4	(1.6 - 3.4)	4.5	(3.4 - 5.8)	34.8
Türkiye (1998)	6.6	(5.1 - 8.2)	1.9	(1.2 - 2.9)	4.6	(3.4 - 6.1)	29.2
Türkiye (2003/4)	6.8	(5.4 - 8.3)	1.5	(1.0 - 2.4)	5.2	(4.0 - 6.6)	22.9
Türkiye (2018/19)	6.6	(4.5 - 9.3)	3.7	(2.2 - 5.8)	2.9	(1.6 - 4.9)	55.9
Ukraine (2007)	0.6	(0.2 - 1.6)	0.0	(0.0 - 0.0)	0.6	(0.2 - 1.6)	0.0
Yemen (2013)	12.8	(10.8 - 15.0)	4.7	(3.5 - 6.2)	8.1	(6.5 - 9.9)	36.7
**Central, South & Southeast Asia**							
Bangladesh (1993/94)	32.3	(27.4 - 37.4)	11.9	(8.8 - 15.6)	20.4	(16.3 - 24.9)	36.9
Bangladesh (1996/97)	35.8	(29.7 - 41.9)	9.6	(6.2 - 13.7)	26.2	(20.9 - 31.9)	26.7
Bangladesh (1999/0)	28.5	(21.7 - 35.6)	10.0	(6.0 - 15.2)	18.5	(12.9 - 24.8)	35.1
Bangladesh (2004)	27.0	(18.6 - 36.0)	4.9	(1.7 - 10.6)	22.1	(14.5 - 30.7)	18.1
Bangladesh (2011)	18.8	(12.3 - 26.3)	2.5	(0.7 - 6.7)	16.3	(10.3 - 23.5)	13.3
Cambodia (2010/11)	5.4	(3.1 - 8.7)	1.5	(0.5 - 3.5)	3.9	(2.0 - 6.9)	27.6
Cambodia (2014)	8.5	(6.2 - 11.2)	3.0	(1.7 - 4.7)	5.5	(3.7 - 7.8)	34.9
Cambodia (2021/22)	9.4	(7.0 - 12.1)	4.9	(3.3 - 7.0)	4.5	(2.9 - 6.5)	52.3
India (2005/6)	15.0	(13.4 - 16.5)	8.9	(7.7 - 10.1)	6.1	(5.1 - 7.2)	59.3
India (2015/16)	14.4	(13.6 - 15.2)	9.9	(9.2 - 10.6)	4.5	(4.0 - 5.0)	68.8
India (2019/21)	19.4	(18.6 - 20.1)	12.1	(11.5 - 12.7)	7.2	(6.8 - 7.7)	62.7
Indonesia (1991)	12.2	(10.8 - 13.6)	5.2	(4.3 - 6.3)	6.9	(5.9 - 8.1)	43.1
Indonesia (1994)	11.6	(10.1 - 13.1)	3.5	(2.7 - 4.4)	8.1	(6.9 - 9.4)	30.1
Indonesia (1997)	9.4	(7.7 - 11.2)	2.6	(1.8 - 3.7)	6.8	(5.4 - 8.4)	27.7
Indonesia (2002/3)	7.3	(5.6 - 9.2)	1.6	(0.9 - 2.6)	5.7	(4.2 - 7.5)	21.7

Indonesia (2007)	8.5	(6.5 - 10.8)	2.4	(1.4 - 3.8)	6.1	(4.4 - 8.1)	28.2
Indonesia (2012)	4.0	(2.7 - 5.5)	1.4	(0.7 - 2.4)	2.6	(1.6 - 3.9)	34.8
Indonesia (2017)	7.4	(6.0 - 8.9)	1.8	(1.1 - 2.6)	5.6	(4.4 - 7.0)	24.1
Kazakhstan (1999)	6.6	(5.2 - 8.2)	3.6	(2.6 - 4.9)	2.9	(2.0 - 4.1)	55.2
Kyrgyz Republic (2012)	6.2	(4.8 - 7.9)	3.2	(2.2 - 4.6)	3.0	(2.0 - 4.2)	52.4
Myanmar (2015/16)	4.8	(2.3 - 8.8)	0.0	(0.0 - 0.0)	4.8	(2.3 - 8.8)	0.0
Nepal (2011)	20.9	(13.0 - 30.2)	8.8	(4.0 - 16.0)	12.1	(6.2 - 20.1)	42.3
Nepal (2016)	21.6	(15.2 - 28.6)	7.5	(3.9 - 12.6)	14.0	(9.0 - 20.2)	34.8
Nepal (2022)	18.3	(11.9 - 25.8)	12.3	(7.1 - 19.0)	6.0	(2.7 - 11.3)	67.0
Pakistan (2012/13)	22.9	(19.3 - 26.8)	11.8	(9.2 - 14.9)	11.1	(8.5 - 14.1)	51.6
Pakistan (2017/18)	18.9	(14.8 - 23.3)	11.9	(8.6 - 15.7)	7.0	(4.6 - 10.1)	62.9
Philippines (1993)	12.1	(8.4 - 16.5)	6.5	(3.9 - 10.0)	5.6	(3.2 - 9.0)	53.5
Philippines (1998)	11.8	(8.4 - 15.8)	3.7	(1.9 - 6.3)	8.1	(5.4 - 11.6)	31.0
Philippines (2003)	8.6	(5.7 - 12.3)	3.3	(1.6 - 5.9)	5.3	(3.1 - 8.4)	38.6
Philippines (2022)	13.2	(9.6 - 17.4)	6.8	(4.3 - 10.1)	6.4	(4.0 - 9.6)	51.5
Tajikistan (2012)	5.3	(3.9 - 6.8)	4.0	(2.9 - 5.4)	1.3	(0.7 - 2.2)	76.1
Tajikistan (2017)	8.9	(7.2 - 10.8)	7.3	(5.8 - 9.0)	1.6	(1.0 - 2.6)	81.7
Vietnam (1997)	7.7	(6.3 - 9.1)	3.4	(2.5 - 4.4)	4.3	(3.3 - 5.4)	44.4
Vietnam (2002)	9.1	(7.6 - 10.9)	3.1	(2.2 - 4.2)	6.1	(4.8 - 7.6)	33.5
**Latin America & Caribbean**							
Bolivia (1994)	8.7	(6.2 - 11.6)	3.9	(2.4 - 6.1)	4.7	(3.0 - 7.1)	45.3
Brazil (1996)	10.6	(5.8 - 17.1)	3.4	(1.1 - 8.0)	7.2	(3.4 - 13.0)	32.0
Colombia (1990)	11.8	(9.7 - 14.3)	4.2	(2.9 - 5.8)	7.6	(5.9 - 9.6)	35.6
Colombia (1995)	13.5	(11.3 - 15.9)	4.1	(2.9 - 5.5)	9.4	(7.6 - 11.5)	30.1
Colombia (2000)	10.7	(8.7 - 13.0)	1.8	(1.1 - 2.9)	8.9	(7.0 - 11.0)	17.1
Colombia (2005)	13.0	(11.8 - 14.4)	4.4	(3.7 - 5.2)	8.6	(7.6 - 9.8)	33.7
Colombia (2010)	14.7	(13.3 - 16.3)	4.2	(3.4 - 5.1)	10.5	(9.3 - 11.9)	28.6
Colombia (2015/16)	11.7	(9.6 - 14.0)	4.6	(3.3 - 6.2)	7.1	(5.5 - 9.0)	39.2
Dominican Republic (1991)	28.3	(22.2 - 34.7)	9.1	(5.7 - 13.6)	19.2	(14.1 - 24.9)	32.2
Dominican Republic (1996)	22.3	(17.5 - 27.5)	8.7	(5.7 - 12.5)	13.6	(9.8 - 18.0)	39.0
Dominican Republic (2002)	19.0	(16.0 - 22.2)	11.2	(8.9 - 13.8)	7.8	(5.9 - 10.1)	58.7
Guatemala (1995)	9.4	(5.9 - 13.7)	5.1	(2.7 - 8.7)	4.2	(2.1 - 7.5)	54.7
Guatemala (2014/15)	7.9	(4.9 - 11.8)	1.7	(0.6 - 4.1)	6.1	(3.5 - 9.7)	22.1
Honduras (2011/12)	20.1	(17.7 - 22.7)	6.2	(4.8 - 7.8)	13.9	(11.8 - 16.1)	30.9
Nicaragua (1998)	18.1	(15.9 - 20.3)	6.1	(4.9 - 7.6)	11.9	(10.1 - 13.8)	34.0
Paraguay (1990)	12.5	(8.7 - 17.1)	3.8	(1.9 - 6.8)	8.7	(5.5 - 12.7)	30.6
Peru (1991/92)	9.4	(7.9 - 11.0)	2.8	(2.0 - 3.8)	6.6	(5.4 - 8.0)	29.5
Peru (1996)	15.1	(13.7 - 16.5)	3.5	(2.9 - 4.3)	11.5	(10.3 - 12.8)	23.4

Peru (2000)	13.5	(11.8 - 15.2)	3.4	(2.6 - 4.4)	10.1	(8.7 - 11.7)	25.1
Peru (2004/6)	18.1	(14.9 - 21.6)	4.8	(3.2 - 6.9)	13.2	(10.5 - 16.4)	26.8
Peru (2007/8)	13.2	(10.4 - 16.3)	3.3	(2.0 - 5.1)	10.0	(7.5 - 12.8)	24.7
Peru (2009)	15.3	(12.3 - 18.6)	2.7	(1.5 - 4.5)	12.6	(9.9 - 15.7)	17.8
Peru (2010)	16.0	(12.7 - 19.6)	4.5	(2.9 - 6.8)	11.5	(8.7 - 14.7)	28.4
Peru (2011)	19.1	(15.1 - 23.4)	2.4	(1.2 - 4.5)	16.7	(12.9 - 20.8)	12.7
Peru (2012)	14.5	(11.2 - 18.3)	2.8	(1.5 - 4.8)	11.7	(8.7 - 15.2)	19.4

CI= Confidence Interval

S14.3 Table: 12-month cumulative incidence of abandonment following method-related discontinuation per 100 episodes of use

Injectables

	Method-related		Abandoned after method-related discontinuation: Yes		Abandoned after method-related discontinuation: No		Percentage abandoned
	Rate	95%CI	Rate	95%CI	Rate	95%CI	
**Sub-Saharan Africa**							
Angola (2015/16)	24.2	(20.5 - 28.1)	21.7	(18.2 - 25.5)	2.5	(1.4 - 4.2)	89.7
Benin (2017/18)	24.1	(20.7 - 27.7)	20.6	(17.4 - 24.1)	3.5	(2.2 - 5.2)	85.6
Burkina Faso (2010)	13.9	(11.9 - 16.0)	11.7	(9.9 - 13.7)	2.1	(1.4 - 3.1)	84.6
Burkina Faso (2021)	19.6	(17.8 - 21.4)	14.5	(12.9 - 16.2)	5.1	(4.1 - 6.1)	74.1
Burundi (2010/11)	22.2	(19.6 - 25.0)	16.6	(14.3 - 19.1)	5.7	(4.3 - 7.3)	74.6
Comoros (2012)	15.7	(11.5 - 20.5)	14.7	(10.6 - 19.4)	1.0	(0.2 - 2.9)	93.7
Côte d'Ivoire (2021)	24.4	(21.3 - 27.6)	19.1	(16.3 - 22.1)	5.3	(3.8 - 7.1)	78.2
Ethiopia (2005)	17.9	(15.8 - 20.2)	11.8	(10.0 - 13.7)	6.2	(4.9 - 7.7)	65.6
Ethiopia (2016)	16.7	(15.5 - 17.9)	12.2	(11.2 - 13.3)	4.4	(3.8 - 5.1)	73.5
Gabon (2019/21)	38.0	(28.4 - 47.5)	27.4	(19.0 - 36.4)	10.6	(5.5 - 17.6)	72.1
Gambia (2013)	13.6	(10.1 - 17.5)	10.2	(7.2 - 13.8)	3.4	(1.8 - 5.7)	75.1
Gambia (2019/20)	23.3	(20.9 - 25.7)	18.9	(16.7 - 21.1)	4.4	(3.4 - 5.7)	81.1
Ghana (2014)	17.5	(14.8 - 20.4)	13.9	(11.5 - 16.6)	3.6	(2.4 - 5.2)	79.4
Ghana (2022/23)	38.8	(36.4 - 41.1)	30.7	(28.5 - 33.0)	8.0	(6.8 - 9.4)	79.3
Guinea (2018)	46.6	(42.1 - 50.9)	34.0	(29.9 - 38.1)	12.6	(9.9 - 15.6)	73.0
Kenya (1998)	13.7	(11.3 - 16.3)	9.3	(7.4 - 11.5)	4.3	(3.0 - 6.0)	68.3
Kenya (2003)	23.3	(20.8 - 25.8)	15.7	(13.6 - 18.0)	7.6	(6.1 - 9.2)	67.5
Kenya (2014)	18.9	(17.6 - 20.2)	9.5	(8.6 - 10.5)	9.4	(8.4 - 10.3)	50.5
Kenya (2022)	27.6	(26.0 - 29.2)	17.6	(16.2 - 18.9)	10.0	(9.0 - 11.1)	63.6
Lesotho (2014)	16.3	(14.3 - 18.5)	8.1	(6.6 - 9.7)	8.3	(6.8 - 9.9)	49.4
Liberia (2013)	22.4	(19.9 - 24.9)	21.1	(18.7 - 23.5)	1.3	(0.7 - 2.1)	94.3
Liberia (2019/20)	31.0	(28.8 - 33.2)	28.5	(26.4 - 30.7)	2.5	(1.8 - 3.3)	92.0
Madagascar (2021)	15.9	(14.9 - 16.9)	10.0	(9.2 - 10.9)	5.8	(5.2 - 6.5)	63.2
Malawi (2004/5)	16.6	(15.2 - 18.0)	14.9	(13.6 - 16.3)	1.7	(1.2 - 2.2)	89.9
Malawi (2015/16)	22.0	(21.1 - 22.9)	16.7	(15.9 - 17.6)	5.3	(4.8 - 5.8)	76.1
Mali (2012/13)	18.8	(15.5 - 22.4)	15.4	(12.3 - 18.8)	3.4	(2.1 - 5.3)	81.7
Mali (2018)	22.0	(19.4 - 24.8)	20.4	(17.9 - 23.1)	1.6	(1.0 - 2.6)	92.7
Mozambique (2011)	21.0	(18.2 - 24.0)	17.5	(14.9 - 20.3)	3.5	(2.4 - 5.0)	83.1
Mozambique (2022/23)	15.1	(13.4 - 16.9)	12.2	(10.7 - 13.9)	2.9	(2.1 - 3.8)	81.1
Namibia (2013)	13.6	(12.1 - 15.1)	10.2	(8.9 - 11.6)	3.3	(2.6 - 4.2)	75.4
Niger (2012)	19.1	(15.6 - 22.8)	12.5	(9.7 - 15.8)	6.6	(4.5 - 9.1)	65.7
Nigeria (2013)	12.3	(10.4 - 14.4)	9.6	(8.0 - 11.5)	2.6	(1.8 - 3.8)	78.5
Nigeria (2018)	32.3	(30.0 - 34.7)	27.6	(25.4 - 29.9)	4.7	(3.7 - 5.8)	85.4

Rwanda (2010/11)	16.3	(14.9 - 17.7)	8.1	(7.1 - 9.1)	8.2	(7.2 - 9.3)	49.6
Rwanda (2014/15)	18.5	(17.1 - 19.9)	10.0	(8.9 - 11.1)	8.5	(7.5 - 9.5)	54.2
Rwanda (2019/20)	24.4	(22.6 - 26.2)	12.9	(11.6 - 14.4)	11.4	(10.1 - 12.8)	53.1
Senegal (2010/11)	30.6	(27.5 - 33.8)	22.1	(19.3 - 24.9)	8.6	(6.8 - 10.6)	72.0
Senegal (2015)	17.4	(14.9 - 20.1)	11.1	(9.1 - 13.3)	6.3	(4.8 - 8.1)	63.8
Senegal (2016)	23.4	(20.6 - 26.2)	12.9	(10.7 - 15.2)	10.5	(8.6 - 12.6)	55.1
Senegal (2018)	20.6	(18.3 - 23.1)	13.9	(11.9 - 16.0)	6.7	(5.4 - 8.3)	67.3
Senegal (2019)	22.2	(19.6 - 24.9)	17.2	(14.8 - 19.7)	5.0	(3.7 - 6.5)	77.6
Senegal (2023)	22.8	(20.6 - 25.0)	18.9	(16.9 - 21.0)	3.9	(2.9 - 5.0)	83.1
Sierra Leone (2013)	18.0	(16.2 - 19.8)	13.5	(11.9 - 15.1)	4.5	(3.6 - 5.6)	75.0
South Africa (2016)	15.6	(13.9 - 17.4)	12.3	(10.8 - 14.0)	3.2	(2.5 - 4.2)	79.2
Tanzania (2004/5)	26.6	(24.1 - 29.1)	17.8	(15.7 - 20.0)	8.8	(7.3 - 10.4)	66.9
Tanzania (2015/16)	21.3	(19.6 - 23.0)	15.6	(14.1 - 17.1)	5.7	(4.8 - 6.8)	73.2
Tanzania (2022)	29.7	(27.7 - 31.8)	22.8	(20.9 - 24.7)	7.0	(5.9 - 8.2)	76.6
Uganda (2011)	27.6	(25.4 - 29.8)	22.9	(20.9 - 25.0)	4.7	(3.7 - 5.8)	83.0
Zambia (2013/14)	14.8	(13.5 - 16.1)	10.9	(9.8 - 12.0)	3.9	(3.3 - 4.7)	73.4
Zambia (2018/19)	19.4	(18.1 - 20.7)	15.1	(13.9 - 16.3)	4.3	(3.7 - 5.0)	77.8
Zimbabwe (1994)	8.2	(4.0 - 14.4)	3.8	(1.3 - 8.7)	4.4	(1.5 - 9.5)	46.8
Zimbabwe (1999)	19.0	(15.9 - 22.4)	9.6	(7.4 - 12.2)	9.4	(7.1 - 12.0)	50.6
Zimbabwe (2005/6)	17.1	(14.6 - 19.8)	9.6	(7.7 - 11.8)	7.5	(5.8 - 9.4)	56.4
Zimbabwe (2010/11)	22.2	(19.2 - 25.4)	12.6	(10.2 - 15.2)	9.6	(7.6 - 12.0)	56.6
Zimbabwe (2015)	21.2	(18.7 - 23.9)	8.5	(6.9 - 10.4)	12.7	(10.7 - 14.8)	40.3
**North Africa Western Asia and Europe**							
Egypt (1992/93)	40.4	(31.0 - 49.5)	13.4	(7.8 - 20.6)	26.9	(18.9 - 35.6)	33.3
Egypt (1995/96)	39.8	(35.0 - 44.7)	19.0	(15.3 - 23.0)	20.9	(17.0 - 25.0)	47.6
Egypt (2000)	37.7	(35.0 - 40.4)	19.7	(17.6 - 22.0)	18.0	(15.9 - 20.2)	52.3
Egypt (2003)	32.3	(29.4 - 35.2)	14.0	(11.9 - 16.3)	18.3	(15.9 - 20.7)	43.4
Egypt (2005)	29.7	(27.9 - 31.6)	14.5	(13.1 - 16.0)	15.2	(13.8 - 16.7)	48.9
Egypt (2008)	23.7	(21.6 - 25.9)	12.2	(10.6 - 14.0)	11.5	(9.9 - 13.2)	51.6
Egypt (2014)	22.0	(20.1 - 23.9)	11.1	(9.7 - 12.6)	10.9	(9.5 - 12.4)	50.6
Jordan (2002)	46.6	(36.6 - 56.0)	18.9	(12.0 - 27.0)	27.7	(19.4 - 36.6)	40.6
Jordan (2007)	32.4	(25.4 - 39.5)	13.8	(9.1 - 19.5)	18.6	(13.1 - 24.8)	42.6
Jordan (2009)	54.6	(47.9 - 60.8)	20.6	(15.6 - 26.1)	34.0	(28.0 - 40.1)	37.8
Jordan (2012)	37.0	(30.7 - 43.4)	14.4	(10.2 - 19.4)	22.6	(17.4 - 28.3)	38.9
Jordan (2017/18)	38.7	(30.5 - 46.7)	13.8	(8.7 - 20.0)	24.9	(18.0 - 32.3)	35.7
Jordan (2023)	32.6	(25.8 - 39.5)	17.2	(12.1 - 23.2)	15.4	(10.6 - 21.0)	52.9
Morocco (2003/4)	42.2	(37.0 - 47.3)	12.8	(9.6 - 16.4)	29.5	(24.8 - 34.3)	30.2
Türkiye (2003/4)	66.3	(58.2 - 73.2)	16.6	(11.2 - 22.9)	49.8	(41.6 - 57.4)	25.0
Türkiye (2018/19)	43.8	(33.0 - 54.0)	22.0	(13.9 - 31.2)	21.8	(13.8 - 31.0)	50.2
Yemen (2013)	29.3	(26.7 - 32.0)	16.2	(14.1 - 18.4)	13.2	(11.3 - 15.2)	55.1
**Central, South & Southeast Asia**							

Bangladesh (1993/94)	47.4	(43.5 - 51.1)	20.8	(17.8 - 24.0)	26.5	(23.2 - 30.0)	44.0
Bangladesh (1996/97)	39.8	(36.5 - 43.1)	17.3	(14.8 - 19.9)	22.5	(19.8 - 25.4)	43.4
Bangladesh (1999/0)	41.4	(38.5 - 44.3)	16.6	(14.5 - 18.8)	24.8	(22.4 - 27.4)	40.0
Bangladesh (2004)	38.7	(36.2 - 41.1)	10.8	(9.3 - 12.4)	27.9	(25.6 - 30.1)	27.9
Bangladesh (2011)	27.4	(25.5 - 29.3)	8.6	(7.5 - 9.8)	18.8	(17.2 - 20.5)	31.3
Bangladesh (2014)	17.2	(15.6 - 18.9)	5.3	(4.3 - 6.3)	12.0	(10.6 - 13.4)	30.6
Bangladesh (2017/18)	24.7	(23.0 - 26.4)	5.5	(4.6 - 6.4)	19.2	(17.7 - 20.8)	22.1
Bangladesh (2022)	19.0	(17.3 - 20.8)	6.2	(5.2 - 7.3)	12.8	(11.4 - 14.3)	32.7
Cambodia (2010/11)	18.9	(16.7 - 21.1)	10.9	(9.2 - 12.7)	8.0	(6.5 - 9.6)	57.7
Cambodia (2014)	20.6	(18.5 - 22.8)	9.6	(8.1 - 11.2)	11.0	(9.4 - 12.8)	46.4
Cambodia (2021/22)	29.8	(26.9 - 32.7)	16.0	(13.7 - 18.4)	13.8	(11.7 - 16.1)	53.7
India (2005/6)	29.5	(22.5 - 36.8)	9.2	(5.3 - 14.3)	20.3	(14.3 - 27.0)	31.2
India (2015/16)	30.9	(28.3 - 33.5)	20.2	(18.0 - 22.5)	10.7	(9.0 - 12.5)	65.4
India (2019/21)	28.1	(26.8 - 29.4)	16.6	(15.6 - 17.7)	11.5	(10.6 - 12.4)	59.1
Indonesia (1991)	21.6	(20.3 - 23.0)	7.0	(6.2 - 7.9)	14.6	(13.4 - 15.8)	32.5
Indonesia (1994)	19.4	(18.4 - 20.6)	6.6	(5.9 - 7.3)	12.9	(11.9 - 13.8)	33.9
Indonesia (1997)	16.2	(15.3 - 17.1)	5.2	(4.7 - 5.8)	11.0	(10.2 - 11.8)	32.1
Indonesia (2002/3)	11.2	(10.5 - 11.9)	3.6	(3.2 - 4.0)	7.6	(7.0 - 8.2)	32.0
Indonesia (2007)	13.7	(13.0 - 14.4)	4.1	(3.7 - 4.5)	9.6	(9.0 - 10.2)	30.0
Indonesia (2012)	12.9	(12.3 - 13.5)	3.7	(3.4 - 4.1)	9.2	(8.7 - 9.7)	28.8
Indonesia (2017)	17.4	(16.7 - 18.1)	4.5	(4.2 - 4.9)	12.9	(12.3 - 13.5)	26.1
Maldives (2009)	32.6	(24.7 - 40.8)	14.7	(9.3 - 21.3)	17.9	(11.8 - 24.9)	45.2
Myanmar (2015/16)	24.9	(23.4 - 26.5)	12.5	(11.4 - 13.7)	12.4	(11.3 - 13.6)	50.3
Nepal (2011)	29.3	(27.1 - 31.7)	17.5	(15.7 - 19.5)	11.8	(10.2 - 13.5)	59.7
Nepal (2016)	23.2	(21.1 - 25.3)	12.7	(11.2 - 14.4)	10.4	(9.0 - 12.0)	55.0
Nepal (2022)	24.9	(22.9 - 26.8)	12.8	(11.4 - 14.4)	12.0	(10.6 - 13.6)	51.6
Pakistan (2012/13)	41.8	(38.5 - 45.0)	26.5	(23.6 - 29.4)	15.3	(13.0 - 17.8)	63.4
Pakistan (2017/18)	30.8	(27.1 - 34.6)	21.9	(18.7 - 25.3)	8.9	(6.8 - 11.4)	71.1
Philippines (1998)	37.8	(32.8 - 42.9)	19.1	(15.2 - 23.3)	18.8	(14.9 - 23.0)	50.4
Philippines (2003)	39.3	(35.3 - 43.2)	14.6	(11.9 - 17.6)	24.6	(21.2 - 28.2)	37.3
Philippines (2022)	37.0	(34.1 - 39.9)	16.3	(14.1 - 18.6)	20.7	(18.3 - 23.2)	44.0
Tajikistan (2012)	28.7	(21.9 - 35.8)	20.5	(14.7 - 27.0)	8.2	(4.6 - 13.2)	71.4
Tajikistan (2017)	27.1	(18.6 - 36.3)	24.6	(16.5 - 33.6)	2.5	(0.5 - 7.5)	90.9
**Latin America & Caribbean**							
Bolivia (1994)	52.5	(44.5 - 59.9)	17.7	(12.3 - 23.9)	34.8	(27.5 - 42.2)	33.7
Brazil (1996)	33.8	(28.8 - 38.8)	11.4	(8.3 - 15.0)	22.4	(18.2 - 26.9)	33.7
Colombia (1990)	39.0	(34.1 - 44.0)	8.6	(6.0 - 11.8)	30.4	(25.8 - 35.2)	22.0
Colombia (1995)	45.8	(41.8 - 49.6)	13.1	(10.6 - 15.8)	32.7	(29.1 - 36.4)	28.6
Colombia (2000)	41.1	(38.1 - 44.1)	11.5	(9.7 - 13.5)	29.6	(26.8 - 32.3)	28.1
Colombia (2005)	34.3	(32.8 - 35.7)	12.5	(11.5 - 13.5)	21.8	(20.6 - 23.0)	36.4
Colombia (2010)	33.1	(32.1 - 34.2)	11.9	(11.2 - 12.6)	21.3	(20.3 - 22.2)	35.9

Colombia (2015/16)	33.2	(32.1 - 34.2)	11.8	(11.1 - 12.6)	21.3	(20.4 - 22.3)	35.7
Dominican Republic (2002)	44.4	(41.4 - 47.4)	23.7	(21.2 - 26.4)	20.7	(18.3 - 23.2)	53.4
Guatemala (1995)	31.6	(26.6 - 36.8)	14.5	(10.9 - 18.6)	17.1	(13.2 - 21.4)	45.9
Guatemala (1998/99)	44.1	(39.1 - 49.0)	21.5	(17.6 - 25.7)	22.6	(18.6 - 26.9)	48.8
Guatemala (2014/15)	17.6	(16.5 - 18.8)	10.1	(9.2 - 11.0)	7.5	(6.8 - 8.3)	57.4
Honduras (2011/12)	32.3	(31.1 - 33.5)	11.5	(10.7 - 12.4)	20.8	(19.7 - 21.9)	35.6
Nicaragua (1998)	35.7	(32.6 - 38.9)	12.3	(10.3 - 14.5)	23.5	(20.7 - 26.3)	34.3
Paraguay (1990)	40.5	(36.9 - 44.0)	10.2	(8.1 - 12.5)	30.3	(27.0 - 33.6)	25.2
Peru (1991/92)	49.9	(45.9 - 53.7)	15.4	(12.7 - 18.3)	34.5	(30.8 - 38.2)	30.9
Peru (1996)	38.6	(36.4 - 40.7)	11.7	(10.4 - 13.1)	26.9	(25.0 - 28.8)	30.4
Peru (2000)	32.1	(30.7 - 33.6)	11.0	(10.1 - 12.0)	21.1	(19.9 - 22.4)	34.2
Peru (2004/6)	37.3	(35.6 - 39.0)	11.2	(10.1 - 12.3)	26.2	(24.6 - 27.7)	29.9
Peru (2007/8)	39.0	(37.6 - 40.4)	11.2	(10.4 - 12.1)	27.8	(26.5 - 29.1)	28.8
Peru (2009)	35.3	(34.0 - 36.5)	11.2	(10.4 - 12.0)	24.1	(23.0 - 25.2)	31.7
Peru (2010)	37.0	(35.8 - 38.3)	9.5	(8.8 - 10.3)	27.5	(26.3 - 28.7)	25.7
Peru (2011)	36.7	(35.4 - 38.0)	11.1	(10.3 - 12.0)	25.6	(24.4 - 26.8)	30.2
Peru (2012)	38.2	(37.0 - 39.5)	10.5	(9.7 - 11.3)	27.8	(26.6 - 28.9)	27.4

CI= Confidence Interval

S14.4 Table: 12-month cumulative incidence of abandonment following method-related discontinuation per 100 episodes of use

Condom

	Method-related		Abandoned after method-related discontinuation: Yes		Abandoned after method-related discontinuation: No		Percentage abandoned
	Rate	95%CI	Rate	95%CI	Rate	95%CI	
**Sub-Saharan Africa**							
Angola (2015/16)	9.0	(7.0 - 11.2)	7.1	(5.4 - 9.1)	1.9	(1.1 - 3.0)	78.9
Benin (2017/18)	3.1	(1.6 - 5.3)	1.7	(0.7 - 3.6)	1.3	(0.5 - 3.0)	56.4
Burkina Faso (2010)	2.2	(1.2 - 3.8)	1.2	(0.5 - 2.4)	1.1	(0.4 - 2.3)	51.8
Burkina Faso (2021)	5.2	(3.7 - 7.2)	2.8	(1.7 - 4.3)	2.5	(1.5 - 3.8)	52.9
Burundi (2010/11)	7.7	(3.0 - 15.4)	6.5	(2.3 - 13.8)	1.2	(0.1 - 6.3)	84.2
Comoros (2012)	7.2	(3.2 - 13.4)	4.0	(1.3 - 9.1)	3.2	(0.9 - 8.1)	55.2
Côte d'Ivoire (2021)	6.5	(4.9 - 8.4)	3.7	(2.6 - 5.3)	2.7	(1.7 - 4.0)	57.9
Ethiopia (2005)	12.8	(6.2 - 21.9)	3.7	(0.9 - 10.2)	9.1	(3.8 - 17.3)	29.1
Gabon (2019/21)	7.2	(6.0 - 8.5)	5.0	(4.0 - 6.1)	2.2	(1.6 - 3.0)	69.2
Ghana (2014)	6.0	(3.5 - 9.4)	4.1	(2.1 - 7.2)	1.8	(0.7 - 4.1)	69.5
Ghana (2022/23)	13.7	(10.8 - 16.9)	7.7	(5.6 - 10.2)	6.0	(4.1 - 8.4)	56.1
Guinea (2018)	8.2	(5.5 - 11.6)	8.0	(5.4 - 11.4)	0.2	(0.0 - 1.6)	97.9
Kenya (1998)	16.6	(12.5 - 21.1)	7.5	(4.9 - 10.9)	9.1	(6.1 - 12.8)	45.2
Kenya (2003)	14.7	(11.1 - 18.8)	9.1	(6.3 - 12.6)	5.6	(3.4 - 8.5)	61.9
Kenya (2014)	4.7	(3.4 - 6.4)	2.4	(1.5 - 3.7)	2.3	(1.4 - 3.6)	51.2
Kenya (2022)	11.0	(8.8 - 13.5)	5.2	(3.7 - 7.1)	5.8	(4.2 - 7.7)	47.6
Lesotho (2014)	7.0	(5.7 - 8.5)	3.5	(2.6 - 4.6)	3.5	(2.6 - 4.6)	49.7
Liberia (2019/20)	21.8	(15.6 - 28.8)	8.6	(4.8 - 13.9)	13.2	(8.4 - 19.1)	39.6
Madagascar (2021)	16.0	(10.5 - 22.4)	8.7	(4.8 - 14.0)	7.3	(3.8 - 12.3)	54.3
Malawi (2004/5)	18.1	(14.7 - 21.9)	10.4	(7.9 - 13.5)	7.7	(5.4 - 10.5)	57.6
Malawi (2015/16)	23.2	(20.8 - 25.6)	11.7	(10.0 - 13.6)	11.5	(9.7 - 13.4)	50.6
Mozambique (2011)	8.6	(6.3 - 11.3)	5.5	(3.6 - 7.8)	3.1	(1.9 - 4.9)	63.4
Mozambique (2022/23)	11.2	(8.5 - 14.4)	4.9	(3.2 - 7.3)	6.3	(4.3 - 8.8)	44.1
Namibia (2013)	3.6	(2.7 - 4.6)	1.1	(0.7 - 1.7)	2.5	(1.8 - 3.3)	30.8
Nigeria (2013)	2.4	(1.8 - 3.2)	1.0	(0.7 - 1.6)	1.4	(0.9 - 2.1)	42.2
Nigeria (2018)	7.1	(5.8 - 8.5)	4.7	(3.6 - 5.9)	2.4	(1.7 - 3.3)	66.2
Rwanda (2010/11)	8.9	(5.5 - 13.2)	2.1	(0.7 - 4.7)	6.8	(3.9 - 10.8)	23.2
Rwanda (2014/15)	13.2	(9.7 - 17.2)	2.5	(1.2 - 4.7)	10.6	(7.5 - 14.4)	19.3
Rwanda (2019/20)	21.4	(17.9 - 25.3)	8.0	(5.8 - 10.7)	13.5	(10.6 - 16.7)	37.2
Senegal (2010/11)	13.7	(9.2 - 19.1)	8.9	(5.3 - 13.6)	4.8	(2.4 - 8.5)	64.9
Sierra Leone (2013)	17.6	(11.7 - 24.5)	3.3	(1.1 - 7.4)	14.3	(9.0 - 20.8)	18.7
South Africa (2016)	9.9	(8.0 - 11.9)	6.5	(5.0 - 8.3)	3.4	(2.4 - 4.6)	66.0
Tanzania (2004/5)	15.6	(12.7 - 18.8)	7.2	(5.3 - 9.6)	8.4	(6.3 - 10.9)	46.3

Tanzania (2015/16)	10.4	(8.0 - 13.3)	3.3	(2.0 - 5.1)	7.1	(5.1 - 9.6)	31.5
Tanzania (2022)	10.3	(7.1 - 14.1)	5.2	(3.0 - 8.2)	5.1	(3.0 - 8.0)	50.4
Uganda (2011)	6.5	(4.4 - 9.3)	4.5	(2.7 - 6.9)	2.1	(1.0 - 3.9)	68.1
Zambia (2013/14)	11.6	(9.4 - 14.1)	2.4	(1.5 - 3.8)	9.2	(7.2 - 11.4)	21.0
Zambia (2018/19)	24.5	(21.1 - 28.1)	10.8	(8.4 - 13.6)	13.7	(11.1 - 16.6)	44.2
Zimbabwe (1994)	11.6	(8.3 - 15.6)	4.2	(2.3 - 7.0)	7.4	(4.8 - 10.7)	36.5
Zimbabwe (1999)	12.2	(8.2 - 17.1)	5.7	(3.1 - 9.5)	6.5	(3.7 - 10.3)	47.0
Zimbabwe (2005/6)	11.3	(8.4 - 14.8)	4.1	(2.4 - 6.6)	7.2	(4.9 - 10.1)	36.6
Zimbabwe (2010/11)	13.8	(10.8 - 17.1)	5.6	(3.7 - 7.9)	8.2	(6.0 - 10.9)	40.5
Zimbabwe (2015)	12.4	(9.7 - 15.3)	3.8	(2.4 - 5.7)	8.6	(6.4 - 11.1)	30.8
**North Africa Western Asia and Europe**							
Albania (2017/18)	15.9	(10.5 - 22.3)	8.9	(5.0 - 14.3)	7.0	(3.6 - 11.7)	56.2
Armenia (2000)	12.5	(9.5 - 16.0)	5.6	(3.6 - 8.2)	6.9	(4.7 - 9.7)	44.5
Armenia (2005)	10.5	(7.6 - 13.9)	4.1	(2.4 - 6.5)	6.4	(4.2 - 9.2)	39.4
Armenia (2010)	2.2	(1.1 - 3.9)	0.5	(0.1 - 1.6)	1.7	(0.8 - 3.3)	22.6
Armenia (2015/16)	2.4	(1.3 - 4.1)	0.4	(0.1 - 1.4)	2.0	(1.0 - 3.6)	18.2
Azerbaijan (2006)	13.3	(8.5 - 19.2)	4.2	(1.8 - 8.3)	9.0	(5.2 - 14.2)	31.9
Egypt (1992/93)	30.1	(25.0 - 35.4)	7.4	(4.8 - 10.8)	22.7	(18.2 - 27.6)	24.5
Egypt (1995/96)	23.1	(18.9 - 27.5)	7.5	(5.1 - 10.4)	15.6	(12.2 - 19.5)	32.3
Egypt (2000)	22.8	(17.0 - 29.2)	4.6	(2.2 - 8.4)	18.2	(13.0 - 24.1)	20.3
Egypt (2003)	23.5	(15.7 - 32.3)	3.5	(1.0 - 8.5)	20.0	(12.8 - 28.4)	14.8
Egypt (2005)	19.8	(14.7 - 25.5)	2.6	(1.0 - 5.5)	17.2	(12.4 - 22.6)	13.2
Egypt (2008)	9.9	(5.4 - 16.1)	1.1	(0.1 - 4.4)	8.9	(4.6 - 14.8)	10.8
Egypt (2014)	13.2	(7.6 - 20.5)	0.4	(0.0 - 4.3)	12.8	(7.3 - 20.0)	3.1
Jordan (1990)	26.0	(18.7 - 33.8)	1.8	(0.4 - 5.4)	24.2	(17.2 - 31.9)	6.8
Jordan (1997)	17.4	(13.7 - 21.5)	4.2	(2.5 - 6.6)	13.2	(10.0 - 16.9)	24.1
Jordan (2002)	22.5	(18.8 - 26.5)	5.1	(3.4 - 7.4)	17.4	(14.1 - 21.1)	22.7
Jordan (2007)	12.2	(10.2 - 14.4)	2.8	(1.9 - 4.0)	9.4	(7.7 - 11.4)	22.9
Jordan (2009)	17.8	(15.7 - 20.1)	4.6	(3.5 - 5.9)	13.2	(11.4 - 15.2)	25.9
Jordan (2012)	12.4	(10.7 - 14.3)	1.6	(1.0 - 2.4)	10.9	(9.2 - 12.6)	12.7
Jordan (2017/18)	10.6	(8.5 - 12.9)	4.7	(3.3 - 6.4)	5.9	(4.4 - 7.7)	44.3
Jordan (2023)	6.2	(4.4 - 8.3)	3.0	(1.8 - 4.7)	3.1	(2.0 - 4.8)	48.9
Moldova (2005)	13.8	(11.4 - 16.3)	2.0	(1.2 - 3.2)	11.7	(9.6 - 14.1)	14.8
Morocco (1992)	10.7	(5.7 - 17.6)	2.0	(0.4 - 6.3)	8.8	(4.3 - 15.2)	18.3
Morocco (2003/4)	33.0	(27.0 - 39.1)	3.5	(1.7 - 6.6)	29.5	(23.7 - 35.4)	10.7
Türkiye (1993)	14.3	(11.8 - 17.0)	2.0	(1.1 - 3.2)	12.3	(10.0 - 14.9)	13.7
Türkiye (1998)	10.7	(8.5 - 13.2)	1.0	(0.4 - 2.0)	9.7	(7.6 - 12.1)	9.1
Türkiye (2003/4)	14.6	(12.6 - 16.7)	1.1	(0.6 - 1.9)	13.5	(11.6 - 15.5)	7.7
Türkiye (2018/19)	6.5	(5.0 - 8.3)	2.3	(1.5 - 3.5)	4.2	(3.0 - 5.7)	35.3
Ukraine (2007)	5.3	(4.2 - 6.5)	0.3	(0.1 - 0.7)	5.0	(4.0 - 6.2)	5.6
Yemen (2013)	16.6	(10.9 - 23.3)	5.3	(2.4 - 9.8)	11.3	(6.7 - 17.4)	31.8

**Central, South & Southeast Asia**							
Bangladesh (1993/94)	22.8	(19.8 - 25.9)	5.3	(3.8 - 7.1)	17.5	(14.8 - 20.3)	23.3
Bangladesh (1996/97)	26.8	(23.5 - 30.1)	5.2	(3.7 - 7.1)	21.6	(18.6 - 24.7)	19.5
Bangladesh (1999/0)	23.2	(20.7 - 25.9)	4.2	(3.1 - 5.6)	19.0	(16.7 - 21.5)	18.2
Bangladesh (2004)	26.8	(24.3 - 29.3)	3.7	(2.8 - 4.9)	23.1	(20.7 - 25.4)	13.9
Bangladesh (2011)	22.6	(20.4 - 24.9)	3.6	(2.7 - 4.7)	19.0	(17.0 - 21.1)	15.9
Bangladesh (2014)	14.7	(12.9 - 16.6)	2.7	(1.9 - 3.7)	12.0	(10.3 - 13.8)	18.3
Bangladesh (2017/18)	21.9	(20.1 - 23.7)	3.1	(2.5 - 4.0)	18.8	(17.1 - 20.5)	14.3
Bangladesh (2022)	12.6	(11.1 - 14.3)	3.0	(2.3 - 3.9)	9.6	(8.3 - 11.1)	23.9
Cambodia (2010/11)	12.3	(8.8 - 16.3)	4.2	(2.3 - 7.1)	8.0	(5.3 - 11.5)	34.4
Cambodia (2014)	14.9	(11.1 - 19.3)	4.3	(2.5 - 6.9)	10.6	(7.4 - 14.5)	28.9
Cambodia (2021/22)	12.5	(8.6 - 17.2)	8.7	(5.5 - 12.8)	3.8	(1.9 - 6.9)	69.3
India (2005/6)	14.9	(14.1 - 15.8)	7.7	(7.0 - 8.3)	7.3	(6.7 - 7.9)	51.2
India (2015/16)	13.6	(13.3 - 14.0)	7.8	(7.5 - 8.0)	5.9	(5.6 - 6.1)	57.0
India (2019/21)	19.6	(19.3 - 19.9)	10.6	(10.3 - 10.8)	9.1	(8.9 - 9.3)	53.8
Indonesia (1991)	25.1	(20.9 - 29.4)	4.7	(2.9 - 7.1)	20.4	(16.5 - 24.5)	18.6
Indonesia (1994)	23.8	(19.3 - 28.7)	6.7	(4.3 - 9.7)	17.2	(13.2 - 21.6)	28.0
Indonesia (1997)	16.5	(11.9 - 21.8)	5.8	(3.2 - 9.4)	10.7	(7.0 - 15.3)	35.0
Indonesia (2002/3)	19.7	(14.8 - 25.1)	2.0	(0.7 - 4.6)	17.7	(13.0 - 22.9)	10.3
Indonesia (2007)	14.3	(11.2 - 17.7)	4.8	(3.1 - 7.2)	9.4	(7.0 - 12.4)	33.8
Indonesia (2012)	17.5	(14.5 - 20.6)	2.1	(1.1 - 3.5)	15.4	(12.6 - 18.4)	11.8
Indonesia (2017)	11.8	(9.9 - 14.0)	2.1	(1.4 - 3.2)	9.7	(7.9 - 11.7)	18.1
Kazakhstan (1999)	27.2	(23.4 - 31.2)	5.3	(3.6 - 7.5)	21.9	(18.4 - 25.6)	19.6
Kyrgyz Republic (2012)	4.3	(2.9 - 6.1)	2.0	(1.1 - 3.4)	2.2	(1.3 - 3.6)	47.8
Maldives (2009)	9.8	(7.9 - 11.8)	6.5	(5.0 - 8.2)	3.3	(2.3 - 4.6)	66.3
Nepal (2011)	11.4	(9.4 - 13.7)	3.2	(2.2 - 4.6)	8.2	(6.5 - 10.2)	28.0
Nepal (2016)	11.5	(9.6 - 13.6)	4.4	(3.2 - 5.8)	7.1	(5.6 - 8.8)	38.3
Nepal (2022)	10.2	(8.3 - 12.4)	3.5	(2.4 - 4.9)	6.7	(5.2 - 8.5)	34.1
Pakistan (2012/13)	10.2	(8.8 - 11.6)	6.5	(5.4 - 7.7)	3.7	(2.9 - 4.7)	63.6
Pakistan (2017/18)	5.2	(4.2 - 6.5)	3.4	(2.6 - 4.4)	1.9	(1.3 - 2.7)	64.6
Philippines (1993)	24.6	(18.7 - 31.0)	8.5	(5.1 - 13.0)	16.1	(11.2 - 21.8)	34.6
Philippines (1998)	29.5	(24.7 - 34.4)	10.3	(7.3 - 13.8)	19.2	(15.2 - 23.6)	34.9
Philippines (2003)	37.2	(32.0 - 42.5)	11.8	(8.6 - 15.5)	25.4	(20.9 - 30.2)	31.7
Philippines (2022)	15.3	(12.1 - 18.8)	6.4	(4.4 - 8.9)	8.9	(6.5 - 11.7)	41.8
Tajikistan (2012)	9.4	(5.9 - 13.9)	6.2	(3.4 - 10.1)	3.2	(1.4 - 6.3)	65.9
Tajikistan (2017)	11.7	(8.5 - 15.4)	9.2	(6.4 - 12.6)	2.5	(1.2 - 4.6)	78.8
Vietnam (1997)	12.6	(9.3 - 16.3)	2.2	(1.1 - 4.2)	10.3	(7.4 - 13.9)	17.8
Vietnam (2002)	16.1	(12.5 - 20.0)	2.9	(1.5 - 4.9)	13.2	(10.0 - 16.9)	17.9
**Latin America & Caribbean**							
Bolivia (1994)	29.4	(23.1 - 36.0)	4.9	(2.4 - 8.5)	24.6	(18.7 - 30.8)	16.5
Brazil (1996)	26.1	(23.6 - 28.6)	4.8	(3.7 - 6.1)	21.2	(18.9 - 23.7)	18.4

Colombia (1990)	23.6	(18.5 - 29.0)	5.5	(3.1 - 8.9)	18.0	(13.6 - 23.0)	23.5
Colombia (1995)	31.2	(28.2 - 34.2)	6.0	(4.6 - 7.7)	25.2	(22.4 - 28.0)	19.3
Colombia (2000)	28.9	(26.8 - 31.2)	4.7	(3.8 - 5.8)	24.2	(22.1 - 26.3)	16.4
Colombia (2005)	25.2	(24.0 - 26.4)	6.6	(6.0 - 7.3)	18.6	(17.5 - 19.6)	26.4
Colombia (2010)	21.9	(21.0 - 22.8)	6.8	(6.2 - 7.3)	15.2	(14.4 - 16.0)	30.8
Colombia (2015/16)	20.5	(19.4 - 21.8)	5.9	(5.3 - 6.7)	14.6	(13.6 - 15.7)	28.9
Dominican Republic (1991)	37.6	(32.2 - 43.0)	16.3	(12.4 - 20.6)	21.3	(16.9 - 26.0)	43.4
Dominican Republic (1996)	31.5	(27.5 - 35.6)	7.4	(5.4 - 10.0)	24.0	(20.4 - 27.9)	23.6
Dominican Republic (2002)	23.4	(20.9 - 26.0)	8.0	(6.4 - 9.8)	15.4	(13.3 - 17.7)	34.2
Guatemala (1995)	22.4	(18.4 - 26.6)	9.4	(6.7 - 12.5)	13.0	(9.9 - 16.6)	41.8
Guatemala (1998/99)	25.4	(20.1 - 31.1)	3.5	(1.7 - 6.5)	21.9	(16.9 - 27.3)	13.9
Guatemala (2014/15)	10.9	(9.2 - 12.8)	3.9	(2.9 - 5.1)	7.0	(5.7 - 8.6)	35.6
Honduras (2011/12)	25.2	(23.7 - 26.7)	8.2	(7.2 - 9.1)	17.0	(15.8 - 18.4)	32.3
Nicaragua (1998)	26.0	(22.5 - 29.7)	7.8	(5.8 - 10.2)	18.2	(15.2 - 21.5)	29.9
Paraguay (1990)	24.9	(19.9 - 30.3)	3.4	(1.6 - 6.2)	21.5	(16.8 - 26.7)	13.6
Peru (1991/92)	33.0	(29.6 - 36.5)	7.9	(6.1 - 10.0)	25.1	(22.0 - 28.4)	23.9
Peru (1996)	22.5	(20.6 - 24.5)	3.2	(2.5 - 4.1)	19.3	(17.5 - 21.2)	14.4
Peru (2000)	23.3	(21.6 - 25.1)	5.0	(4.2 - 6.0)	18.3	(16.7 - 20.0)	21.5
Peru (2004/6)	21.3	(19.7 - 23.0)	3.7	(3.0 - 4.5)	17.6	(16.2 - 19.2)	17.3
Peru (2007/8)	21.1	(19.9 - 22.4)	4.0	(3.4 - 4.6)	17.1	(16.0 - 18.3)	18.9
Peru (2009)	22.0	(20.8 - 23.2)	4.1	(3.5 - 4.7)	17.9	(16.9 - 19.0)	18.6
Peru (2010)	21.1	(20.0 - 22.3)	4.0	(3.4 - 4.5)	17.2	(16.1 - 18.2)	18.8
Peru (2011)	19.7	(18.6 - 20.8)	3.7	(3.2 - 4.3)	16.0	(15.0 - 17.0)	18.9
Peru (2012)	21.1	(20.0 - 22.2)	4.4	(3.9 - 5.0)	16.7	(15.7 - 17.7)	21.0

CI= Confidence Interval

S14.5 Table: 12-month cumulative incidence of abandonment following method-related discontinuation per 100 episodes of use

Implant

	Method-related		Abandoned after method-related discontinuation: Yes		Abandoned after method-related discontinuation: No		Percentage abandoned
	Rate	95%CI	Rate	95%CI	Rate	95%CI	
**Sub-Saharan Africa**							
Benin (2017/18)	8.5	(6.7 - 10.5)	7.5	(5.9 - 9.4)	1.0	(0.5 - 1.8)	88.7
Burkina Faso (2010)	1.3	(0.6 - 2.8)	0.9	(0.3 - 2.2)	0.5	(0.1 - 1.6)	66.2
Burkina Faso (2021)	6.9	(6.0 - 7.9)	5.3	(4.5 - 6.2)	1.7	(1.2 - 2.2)	76.1
Côte d'Ivoire (2021)	9.5	(7.5 - 11.8)	8.4	(6.5 - 10.6)	1.1	(0.6 - 2.1)	88.0
Ethiopia (2016)	5.5	(4.1 - 7.1)	2.9	(2.0 - 4.1)	2.6	(1.7 - 3.7)	53.2
Gambia (2019/20)	7.2	(5.3 - 9.3)	6.0	(4.4 - 8.1)	1.1	(0.5 - 2.2)	84.5
Ghana (2014)	5.7	(3.5 - 8.7)	4.2	(2.4 - 6.9)	1.5	(0.5 - 3.5)	73.9
Ghana (2022/23)	17.8	(15.6 - 20.1)	14.1	(12.1 - 16.2)	3.7	(2.7 - 4.9)	79.4
Guinea (2018)	39.8	(35.1 - 44.4)	27.6	(23.4 - 32.0)	12.2	(9.3 - 15.5)	69.4
Kenya (2014)	7.1	(5.6 - 8.8)	3.6	(2.5 - 4.9)	3.5	(2.5 - 4.8)	50.3
Kenya (2022)	9.9	(8.8 - 11.1)	6.3	(5.4 - 7.3)	3.6	(2.9 - 4.4)	63.8
Liberia (2013)	2.5	(0.7 - 6.4)	2.5	(0.7 - 6.4)	0.0	(0.0 - 0.0)	100.0
Liberia (2019/20)	9.4	(6.7 - 12.6)	7.7	(5.3 - 10.7)	1.7	(0.7 - 3.4)	81.9
Madagascar (2021)	7.6	(6.4 - 9.0)	5.6	(4.6 - 6.8)	2.0	(1.4 - 2.8)	73.4
Malawi (2015/16)	5.3	(4.4 - 6.3)	4.2	(3.4 - 5.1)	1.1	(0.7 - 1.6)	79.8
Mali (2012/13)	7.1	(4.2 - 11.1)	4.9	(2.5 - 8.4)	2.3	(0.8 - 4.9)	68.3
Mali (2018)	7.7	(5.9 - 9.8)	6.2	(4.6 - 8.1)	1.5	(0.8 - 2.6)	80.4
Mozambique (2022/23)	8.3	(6.5 - 10.4)	6.1	(4.6 - 8.0)	2.2	(1.3 - 3.4)	73.8
Nigeria (2018)	10.0	(8.2 - 11.9)	8.3	(6.7 - 10.2)	1.6	(1.0 - 2.5)	83.8
Rwanda (2010/11)	3.9	(2.4 - 6.0)	2.2	(1.1 - 3.9)	1.7	(0.8 - 3.2)	56.8
Rwanda (2014/15)	2.1	(1.2 - 3.5)	1.4	(0.7 - 2.6)	0.7	(0.3 - 1.7)	64.9
Rwanda (2019/20)	5.0	(4.1 - 6.0)	3.1	(2.4 - 3.9)	1.9	(1.3 - 2.5)	62.6
Senegal (2010/11)	10.0	(5.9 - 15.3)	5.6	(2.8 - 9.8)	4.4	(1.8 - 8.7)	56.0
Senegal (2015)	5.5	(3.3 - 8.5)	2.8	(1.3 - 5.3)	2.7	(1.3 - 4.9)	51.5
Senegal (2016)	4.1	(2.6 - 6.2)	3.2	(1.9 - 5.0)	1.0	(0.4 - 2.2)	76.8
Senegal (2018)	6.7	(4.9 - 8.7)	3.9	(2.6 - 5.5)	2.8	(1.7 - 4.3)	58.1
Senegal (2019)	6.3	(4.5 - 8.5)	4.4	(2.9 - 6.4)	1.9	(1.0 - 3.2)	70.1
Senegal (2023)	9.5	(8.0 - 11.1)	7.0	(5.7 - 8.4)	2.5	(1.8 - 3.4)	73.4
Sierra Leone (2013)	7.1	(5.2 - 9.3)	5.7	(4.1 - 7.8)	1.4	(0.7 - 2.5)	80.9
South Africa (2016)	11.3	(8.0 - 15.2)	9.8	(6.7 - 13.6)	1.5	(0.5 - 3.4)	86.7
Tanzania (2015/16)	7.1	(5.4 - 9.0)	5.1	(3.7 - 6.8)	2.0	(1.2 - 3.1)	71.8
Tanzania (2022)	13.7	(12.3 - 15.2)	11.3	(10.0 - 12.6)	2.5	(1.9 - 3.2)	82.1
Uganda (2011)	11.2	(6.4 - 17.4)	9.3	(5.0 - 15.4)	1.9	(0.6 - 4.7)	83.4

Zambia (2013/14)	3.8	(2.5 - 5.5)	2.8	(1.7 - 4.4)	1.0	(0.4 - 2.1)	74.2
Zambia (2018/19)	8.6	(6.8 - 10.5)	5.5	(4.1 - 7.2)	3.1	(2.1 - 4.3)	64.3
Zimbabwe (2010/11)	5.5	(2.5 - 10.4)	1.7	(0.3 - 5.4)	3.8	(1.4 - 8.0)	31.4
Zimbabwe (2015)	4.8	(3.4 - 6.6)	2.7	(1.7 - 4.1)	2.1	(1.3 - 3.4)	55.8
**North Africa Western Asia and Europe**							
Egypt (2005)	17.9	(12.4 - 24.2)	4.9	(2.3 - 8.9)	13.0	(8.4 - 18.8)	27.2
Egypt (2008)	19.9	(13.2 - 27.6)	6.7	(3.0 - 12.4)	13.2	(7.8 - 20.0)	33.7
Egypt (2014)	11.0	(6.8 - 16.3)	3.7	(1.5 - 7.4)	7.3	(4.0 - 11.9)	33.6
Jordan (2023)	15.0	(8.9 - 22.6)	2.7	(0.7 - 7.3)	12.3	(6.8 - 19.4)	17.9
Yemen (2013)	21.8	(15.6 - 28.7)	10.6	(6.4 - 16.1)	11.2	(6.8 - 16.8)	48.7
**Central, South & Southeast Asia**							
Bangladesh (2011)	5.9	(2.8 - 10.5)	1.0	(0.1 - 4.4)	4.9	(2.2 - 9.1)	17.7
Bangladesh (2014)	5.1	(2.9 - 8.3)	2.5	(1.1 - 4.9)	2.6	(1.1 - 5.2)	49.4
Bangladesh (2017/18)	12.2	(9.4 - 15.4)	4.3	(2.7 - 6.4)	7.9	(5.7 - 10.6)	35.0
Bangladesh (2022)	10.8	(7.7 - 14.5)	2.9	(1.5 - 5.1)	7.9	(5.3 - 11.2)	26.6
Cambodia (2014)	5.5	(3.1 - 9.0)	2.3	(0.9 - 4.9)	3.2	(1.4 - 6.1)	42.2
Cambodia (2021/22)	11.6	(8.2 - 15.5)	5.5	(3.3 - 8.5)	6.0	(3.7 - 9.1)	47.9
Indonesia (1991)	2.2	(1.2 - 3.6)	1.3	(0.6 - 2.4)	0.9	(0.4 - 2.0)	57.4
Indonesia (1994)	4.1	(3.0 - 5.3)	2.1	(1.4 - 3.1)	1.9	(1.3 - 2.9)	52.1
Indonesia (1997)	2.5	(1.7 - 3.4)	1.3	(0.8 - 2.0)	1.2	(0.7 - 2.0)	50.9
Indonesia (2002/3)	2.3	(1.5 - 3.4)	0.6	(0.2 - 1.2)	1.7	(1.0 - 2.7)	25.0
Indonesia (2007)	4.6	(3.2 - 6.4)	2.6	(1.6 - 4.1)	2.0	(1.1 - 3.3)	57.1
Indonesia (2012)	5.4	(4.1 - 6.9)	1.7	(1.0 - 2.6)	3.7	(2.6 - 5.0)	31.2
Indonesia (2017)	5.7	(4.6 - 7.0)	2.4	(1.7 - 3.2)	3.3	(2.5 - 4.3)	41.5
Nepal (2011)	7.8	(3.2 - 15.0)	2.3	(0.4 - 7.2)	5.5	(1.9 - 12.1)	29.1
Nepal (2016)	7.1	(4.4 - 10.6)	2.3	(1.0 - 4.6)	4.8	(2.6 - 7.9)	32.5
Nepal (2022)	8.6	(6.6 - 10.9)	4.5	(3.1 - 6.3)	4.1	(2.8 - 5.8)	52.1
Philippines (2022)	7.9	(5.8 - 10.4)	3.6	(2.3 - 5.5)	4.3	(2.8 - 6.2)	46.0
**Latin America & Caribbean**							
Colombia (2005)	7.8	(3.0 - 15.7)	1.3	(0.1 - 6.1)	6.5	(2.2 - 14.1)	16.8
Colombia (2010)	5.5	(4.3 - 7.0)	2.8	(1.9 - 3.9)	2.8	(1.9 - 3.9)	50.0
Colombia (2015/16)	6.2	(5.1 - 7.5)	2.6	(1.9 - 3.5)	3.6	(2.7 - 4.6)	42.2
Dominican Republic (2002)	26.5	(19.1 - 34.6)	13.8	(8.3 - 20.6)	12.8	(7.7 - 19.2)	51.9
Guatemala (2014/15)	8.5	(5.8 - 11.9)	2.5	(1.2 - 4.7)	6.0	(3.7 - 9.0)	29.9

CI= Confidence Interval

S14.6 Table: 12-month cumulative incidence of abandonment following method-related discontinuation per 100 episodes of use

Periodic abstinence

	Method-related		Abandoned after method-related discontinuation				Percentage abandoned
			Yes		No		
	Rate	95%CI	Rate	95%CI	Rate	95%CI	
**Sub-Saharan Africa**							
Benin (2017/18)	6.4	(4.3 - 8.9)	5.1	(3.3 - 7.4)	1.3	(0.5 - 2.7)	79.7
Burkina Faso (2010)	2.7	(1.0 - 5.8)	1.3	(0.3 - 3.8)	1.4	(0.3 - 4.0)	48.9
Burkina Faso (2021)	4.7	(3.1 - 6.9)	2.7	(1.5 - 4.5)	2.0	(1.0 - 3.5)	57.8
Burundi (2010/11)	3.5	(1.3 - 7.7)	0.6	(0.0 - 3.4)	2.9	(0.9 - 6.9)	16.4
Comoros (2012)	0.4	(0.0 - 4.6)	0.4	(0.0 - 4.6)	0.0	(0.0 - 0.0)	100.0
Côte d'Ivoire (2021)	2.5	(1.2 - 4.4)	1.8	(0.8 - 3.5)	0.7	(0.2 - 2.0)	72.0
Ethiopia (2005)	12.2	(6.9 - 19.1)	6.1	(2.6 - 11.8)	6.1	(2.6 - 11.5)	50.3
Ethiopia (2016)	5.6	(2.1 - 11.6)	0.5	(0.0 - 5.2)	5.1	(1.8 - 10.9)	9.4
Gabon (2019/21)	6.1	(3.9 - 9.1)	3.4	(1.8 - 5.8)	2.7	(1.3 - 5.0)	55.2
Ghana (2014)	0.8	(0.2 - 2.4)	0.4	(0.0 - 1.7)	0.5	(0.1 - 1.9)	46.2
Ghana (2022/23)	6.5	(5.1 - 8.2)	2.4	(1.6 - 3.5)	4.1	(3.0 - 5.5)	36.9
Kenya (1998)	4.9	(3.5 - 6.8)	1.6	(0.8 - 2.7)	3.4	(2.2 - 5.0)	31.3
Kenya (2003)	3.2	(2.0 - 4.7)	0.7	(0.2 - 1.6)	2.5	(1.5 - 3.9)	21.4
Kenya (2014)	6.6	(4.7 - 8.8)	0.2	(0.0 - 1.0)	6.3	(4.5 - 8.5)	3.6
Kenya (2022)	9.7	(7.4 - 12.3)	3.6	(2.2 - 5.4)	6.1	(4.4 - 8.2)	37.0
Madagascar (2021)	6.2	(4.9 - 7.7)	1.6	(1.0 - 2.5)	4.6	(3.4 - 5.9)	26.3
Nigeria (2013)	1.3	(0.7 - 2.3)	0.0	(0.0 - 0.0)	1.3	(0.7 - 2.3)	0.0
Nigeria (2018)	3.5	(2.3 - 5.1)	1.8	(1.0 - 3.1)	1.7	(0.9 - 2.9)	51.4
Rwanda (2010/11)	5.8	(3.3 - 9.2)	0.3	(0.0 - 1.9)	5.5	(3.1 - 8.9)	4.4
Rwanda (2014/15)	6.1	(3.4 - 9.9)	1.1	(0.3 - 3.4)	5.0	(2.6 - 8.5)	18.4
Rwanda (2019/20)	7.4	(4.8 - 10.6)	3.6	(1.9 - 6.1)	3.8	(2.1 - 6.2)	48.9
Tanzania (2004/5)	11.2	(7.9 - 15.0)	3.8	(2.1 - 6.3)	7.4	(4.8 - 10.7)	33.9
Tanzania (2015/16)	4.4	(2.9 - 6.4)	0.6	(0.2 - 1.6)	3.9	(2.5 - 5.7)	13.0
Tanzania (2022)	4.9	(3.4 - 6.8)	3.7	(2.4 - 5.4)	1.2	(0.6 - 2.3)	75.5
Uganda (2011)	2.5	(0.7 - 6.5)	0.7	(0.0 - 3.8)	1.9	(0.4 - 5.5)	25.9
Zambia (2013/14)	9.2	(5.1 - 14.8)	1.5	(0.3 - 4.8)	7.7	(4.0 - 13.0)	16.5
**North Africa Western Asia and Europe**							
Armenia (2000)	9.6	(6.4 - 13.6)	1.7	(0.6 - 3.8)	8.0	(5.1 - 11.7)	17.4
Armenia (2005)	3.7	(1.5 - 7.5)	0.0	(0.0 - 0.0)	3.7	(1.5 - 7.5)	0.0
Armenia (2010)	3.0	(0.7 - 8.1)	1.1	(0.1 - 5.4)	1.9	(0.3 - 6.5)	37.1
Armenia (2015/16)	2.5	(0.7 - 6.7)	0.0	(0.0 0.0)	2.5	(0.7 - 6.7)	0.0
Azerbaijan (2006)	1.4	(0.3 - 3.9)	0.2	(0.0 - 2.4)	1.2	(0.3 - 3.6)	13.1
Egypt (1992/93)	11.3	(6.5 - 17.7)	2.7	(0.8 - 6.7)	8.6	(4.5 - 14.4)	23.9

Egypt (1995/96)	11.9	(6.9 - 18.2)	1.4	(0.2 - 4.7)	10.5	(5.9 - 16.6)	11.5
Egypt (2005)	6.8	(3.4 - 12.0)	0.8	(0.1 - 3.7)	6.0	(2.8 - 11.0)	11.6
Jordan (1990)	7.2	(5.4 - 9.3)	2.5	(1.5 - 4.0)	4.7	(3.2 - 6.5)	35.1
Jordan (1997)	14.7	(12.4 - 17.2)	1.8	(1.1 - 2.9)	12.9	(10.7 - 15.3)	12.4
Jordan (2002)	6.8	(4.9 - 9.1)	1.8	(0.9 - 3.2)	5.0	(3.4 - 7.0)	26.7
Jordan (2007)	6.4	(4.8 - 8.4)	1.4	(0.8 - 2.5)	5.0	(3.5 - 6.8)	22.4
Jordan (2009)	12.2	(10.0 - 14.7)	1.1	(0.5 - 2.1)	11.1	(8.9 - 13.5)	9.3
Jordan (2012)	8.9	(6.8 - 11.3)	1.2	(0.5 - 2.3)	7.7	(5.7 - 10.0)	13.5
Jordan (2017/18)	13.5	(8.8 - 19.1)	2.9	(1.1 - 6.2)	10.6	(6.5 - 15.8)	21.3
Jordan (2023)	8.5	(4.6 - 13.9)	1.9	(0.5 - 5.2)	6.6	(3.2 - 11.6)	22.3
Moldova (2005)	11.3	(7.3 - 16.1)	0.6	(0.1 - 2.6)	10.7	(6.8 - 15.5)	5.2
Morocco (1992)	9.2	(6.4 - 12.6)	0.9	(0.2 - 2.4)	8.3	(5.7 - 11.6)	9.4
Morocco (2003/4)	14.6	(11.9 - 17.5)	0.8	(0.3 - 1.8)	13.8	(11.2 - 16.7)	5.3
Türkiye (1993)	11.2	(6.4 - 17.6)	0.8	(0.1 - 4.2)	10.4	(5.8 - 16.6)	7.4
Türkiye (2003/4)	14.7	(9.1 - 21.7)	2.5	(0.6 - 7.0)	12.2	(7.2 - 18.7)	17.3
Ukraine (2007)	11.3	(8.3 - 14.8)	0.4	(0.0 - 1.6)	10.9	(7.9 - 14.4)	3.3
Yemen (2013)	7.5	(5.1 - 10.4)	0.6	(0.1 - 1.8)	6.9	(4.7 - 9.7)	7.5
**Central, South & Southeast Asia**							
Bangladesh (1993/94)	10.3	(8.2 - 12.7)	2.1	(1.2 - 3.4)	8.2	(6.3 - 10.4)	20.5
Bangladesh (1996/97)	9.4	(7.1 - 12.0)	0.8	(0.3 - 1.8)	8.6	(6.4 - 11.1)	8.4
Bangladesh (1999/0)	14.5	(12.0 - 17.2)	1.5	(0.8 - 2.7)	12.9	(10.6 - 15.5)	10.6
Bangladesh (2004)	13.1	(10.9 - 15.4)	1.9	(1.1 - 3.0)	11.2	(9.2 - 13.4)	14.5
Bangladesh (2011)	10.1	(8.2 - 12.1)	0.9	(0.4 - 1.7)	9.2	(7.4 - 11.1)	9.0
Bangladesh (2014)	8.1	(6.2 - 10.4)	0.6	(0.2 - 1.5)	7.5	(5.6 - 9.7)	7.6
Bangladesh (2017/18)	14.1	(12.1 - 16.3)	1.4	(0.9 - 2.3)	12.7	(10.8 - 14.8)	10.2
Bangladesh (2022)	7.3	(5.6 - 9.2)	0.8	(0.4 - 1.7)	6.4	(4.9 - 8.3)	11.5
Cambodia (2010/11)	4.1	(2.3 - 6.6)	0.5	(0.1 - 1.8)	3.6	(1.9 - 5.9)	12.6
Cambodia (2014)	4.4	(2.5 - 7.0)	1.0	(0.3 - 2.7)	3.3	(1.8 - 5.7)	23.6
Cambodia (2021/22)	29.2	(23.0 - 35.7)	6.3	(3.5 - 10.4)	22.9	(17.3 - 28.9)	21.7
India (2005/6)	5.4	(4.9 - 6.0)	1.6	(1.3 - 1.9)	3.9	(3.4 - 4.4)	28.5
India (2015/16)	11.1	(10.7 - 11.5)	5.1	(4.8 - 5.4)	6.0	(5.7 - 6.4)	45.8
India (2019/21)	12.3	(12.0 - 12.6)	5.9	(5.7 - 6.1)	6.4	(6.2 - 6.6)	48.1
Indonesia (1991)	10.1	(7.0 - 13.7)	1.2	(0.4 - 2.8)	8.9	(6.0 - 12.4)	11.5
Indonesia (1994)	8.5	(5.9 - 11.6)	2.8	(1.4 - 4.9)	5.7	(3.6 - 8.4)	32.7
Indonesia (1997)	6.8	(4.3 - 10.1)	0.0	(0.0 - 0.0)	6.8	(4.3 - 10.1)	0.0
Indonesia (2002/3)	6.1	(3.9 - 9.0)	1.0	(0.3 - 2.6)	5.1	(3.1 - 7.8)	15.9
Indonesia (2007)	7.4	(5.1 - 10.2)	1.2	(0.5 - 2.7)	6.1	(4.1 - 8.8)	16.8
Indonesia (2012)	4.4	(2.7 - 6.8)	0.4	(0.1 - 1.5)	4.0	(2.4 - 6.3)	9.0
Indonesia (2017)	5.5	(4.0 - 7.5)	1.0	(0.5 - 2.1)	4.5	(3.1 - 6.3)	18.8
Kazakhstan (1999)	21.2	(16.5 - 26.3)	2.3	(1.0 - 4.7)	18.9	(14.4 - 23.8)	11.0
Maldives (2009)	1.7	(0.5 - 4.4)	0.0	(0.0 0.0)	1.7	(0.5 - 4.4)	0.0

Nepal (2011)	7.1	(2.9 - 13.7)	0.6	(0.0 - 5.1)	6.4	(2.5 - 12.9)	8.8
Nepal (2016)	5.3	(2.8 - 9.1)	1.9	(0.6 - 4.6)	3.4	(1.5 - 6.7)	35.5
Nepal (2022)	3.6	(1.6 - 7.0)	0.6	(0.1 - 2.6)	3.1	(1.2 - 6.3)	15.6
Pakistan (2012/13)	1.1	(0.2 - 4.1)	0.4	(0.0 - 3.3)	0.7	(0.1 - 3.6)	33.7
Pakistan (2017/18)	2.1	(0.6 - 5.7)	0.0	(0.0 - 0.0)	2.1	(0.6 - 5.7)	0.0
Philippines (1993)	4.3	(3.1 - 5.8)	1.8	(1.1 - 2.9)	2.4	(1.5 - 3.6)	43.2
Philippines (1998)	6.8	(5.3 - 8.5)	1.9	(1.2 - 2.8)	4.9	(3.7 - 6.4)	27.3
Philippines (2003)	7.9	(6.1 - 10.0)	2.0	(1.1 - 3.2)	5.9	(4.3 - 7.8)	25.1
Philippines (2022)	2.8	(1.4 - 5.0)	1.5	(0.6 - 3.3)	1.3	(0.5 - 2.9)	53.9
Vietnam (1997)	8.1	(5.5 - 11.4)	0.2	(0.0 - 1.4)	7.9	(5.3 - 11.2)	2.4
Vietnam (2002)	11.6	(8.7 - 15.0)	1.2	(0.5 - 2.7)	10.4	(7.7 - 13.7)	10.5
**Latin America & Caribbean**							
Bolivia (1994)	7.2	(6.1 - 8.4)	1.1	(0.7 - 1.6)	6.1	(5.1 - 7.3)	15.1
Brazil (1996)	19.7	(16.5 - 23.0)	1.5	(0.7 - 2.8)	18.2	(15.1 - 21.4)	7.6
Colombia (1990)	13.3	(10.7 - 16.3)	3.0	(1.8 - 4.6)	10.4	(8.0 - 13.0)	22.2
Colombia (1995)	21.0	(18.7 - 23.4)	3.0	(2.2 - 4.2)	18.0	(15.8 - 20.3)	14.5
Colombia (2000)	22.6	(20.2 - 25.0)	3.0	(2.2 - 4.1)	19.5	(17.3 - 21.8)	13.4
Colombia (2005)	18.2	(16.6 - 19.9)	5.7	(4.8 - 6.8)	12.4	(11.1 - 13.9)	31.6
Colombia (2010)	17.3	(15.3 - 19.4)	3.9	(3.0 - 5.1)	13.4	(11.6 - 15.3)	22.6
Colombia (2015/16)	11.4	(8.9 - 14.2)	2.7	(1.6 - 4.2)	8.7	(6.6 - 11.2)	23.5
Dominican Republic (1991)	13.9	(10.6 - 17.6)	1.2	(0.4 - 2.9)	12.6	(9.5 - 16.2)	8.9
Dominican Republic (1996)	14.8	(11.7 - 18.3)	1.7	(0.8 - 3.3)	13.1	(10.2 - 16.4)	11.7
Dominican Republic (2002)	16.1	(13.6 - 18.7)	3.0	(2.0 - 4.4)	13.1	(10.8 - 15.5)	18.8
Guatemala (1995)	4.1	(2.5 - 6.4)	1.9	(0.9 - 3.5)	2.3	(1.1 - 4.1)	44.9
Guatemala (1998/99)	8.1	(5.5 - 11.3)	4.7	(2.8 - 7.4)	3.3	(1.8 - 5.6)	58.9
Guatemala (2014/15)	3.4	(2.4 - 4.6)	0.4	(0.2 - 1.0)	3.0	(2.1 - 4.1)	12.2
Honduras (2011/12)	22.7	(20.1 - 25.5)	8.2	(6.5 - 10.1)	14.5	(12.3 - 16.8)	36.1
Nicaragua (1998)	12.1	(8.6 - 16.2)	1.7	(0.7 - 3.8)	10.4	(7.1 - 14.3)	14.4
Paraguay (1990)	14.9	(12.0 - 18.2)	1.5	(0.7 - 2.8)	13.5	(10.6 - 16.6)	9.8
Peru (1991/92)	8.2	(7.4 - 9.1)	1.0	(0.7 - 1.4)	7.2	(6.4 - 8.1)	12.4
Peru (1996)	10.0	(9.3 - 10.8)	1.6	(1.3 - 1.9)	8.4	(7.7 - 9.1)	15.7
Peru (2000)	10.8	(9.8 - 11.8)	1.9	(1.5 - 2.4)	8.8	(8.0 - 9.7)	18.1
Peru (2004/6)	12.6	(11.5 - 13.9)	1.8	(1.4 - 2.4)	10.8	(9.7 - 12.0)	14.4
Peru (2007/8)	13.8	(12.8 - 14.9)	2.5	(2.1 - 3.1)	11.3	(10.3 - 12.3)	18.3
Peru (2009)	37.8	(31.8 - 43.9)	0.8	(0.2 - 2.7)	37.1	(31.0 - 43.1)	2.1
Peru (2010)	14.4	(13.3 - 15.6)	2.8	(2.3 - 3.4)	11.6	(10.6 - 12.7)	19.3
Peru (2011)	11.9	(10.8 - 13.1)	2.4	(1.9 - 3.0)	9.5	(8.5 - 10.5)	20.4
Peru (2012)	12.1	(11.0 - 13.2)	2.1	(1.6 - 2.6)	10.0	(9.1 - 11.0)	17.1

CI= Confidence Interval

S14.7 Table: 12-month cumulative incidence of abandonment following method-related discontinuation per 100 episodes of use

Withdrawal

	Method-related		Abandoned after method-related discontinuation				Percentage abandoned
			Yes		No		
	Rate	95%CI	Rate	95%CI	Rate	95%CI	
**Sub-Saharan Africa**							
Angola (2015/16)	7.6	(3.2 - 14.5)	6.7	(2.7 - 13.5)	0.8	(0.0 - 5.2)	89.2
Benin (2017/18)	10.2	(7.0 - 14.2)	2.2	(0.9 - 4.4)	8.1	(5.2 - 11.7)	21.2
Burundi (2010/11)	4.7	(2.1 - 9.1)	0.8	(0.1 - 3.6)	3.9	(1.6 - 8.0)	16.7
Comoros (2012)	0.9	(0.0 - 5.4)	0.0	(0.0 - 0.0)	0.9	(0.0 - 5.4)	0.0
Gabon (2019/21)	13.4	(10.3 - 17.0)	8.7	(6.2 - 11.8)	4.7	(3.0 - 7.0)	64.9
Ghana (2014)	4.9	(2.4 - 9.0)	0.0	(0.0 - 0.0)	4.9	(2.4 - 9.0)	0.0
Ghana (2022/23)	9.7	(7.3 - 12.5)	3.7	(2.3 - 5.6)	6.0	(4.2 - 8.3)	38.2
Kenya (2014)	8.2	(3.8 - 14.6)	2.5	(0.6 - 7.1)	5.7	(2.2 - 11.5)	30.5
Kenya (2022)	20.5	(15.4 - 26.1)	4.5	(2.4 - 7.7)	15.9	(11.4 - 21.2)	22.2
Madagascar (2021)	9.8	(6.4 - 14.2)	4.5	(2.3 - 7.8)	5.3	(2.8 - 8.8)	46.3
Malawi (2004/5)	10.3	(7.7 - 13.3)	3.1	(1.8 - 5.0)	7.2	(5.0 - 9.8)	30.0
Malawi (2015/16)	23.1	(17.9 - 28.7)	8.6	(5.5 - 12.6)	14.5	(10.3 - 19.3)	37.3
Nigeria (2013)	3.1	(2.1 - 4.3)	0.6	(0.3 - 1.2)	2.5	(1.6 - 3.5)	19.9
Nigeria (2018)	5.6	(4.4 - 6.9)	3.8	(2.9 - 4.9)	1.8	(1.2 - 2.6)	68.2
Rwanda (2010/11)	8.5	(5.6 - 12.2)	2.1	(0.9 - 4.4)	6.4	(3.9 - 9.7)	24.9
Rwanda (2014/15)	8.7	(5.6 - 12.7)	1.3	(0.4 - 3.4)	7.4	(4.5 - 11.2)	14.7
Rwanda (2019/20)	15.5	(12.1 - 19.3)	4.1	(2.4 - 6.4)	11.5	(8.5 - 14.9)	26.1
Tanzania (2004/5)	11.3	(8.7 - 14.2)	1.3	(0.6 - 2.6)	10.0	(7.6 - 12.7)	11.7
Tanzania (2015/16)	18.6	(14.3 - 23.4)	2.1	(0.8 - 4.4)	16.5	(12.4 - 21.2)	11.2
Tanzania (2022)	12.4	(8.8 - 16.7)	5.5	(3.2 - 8.7)	6.9	(4.2 - 10.4)	44.5
Uganda (2011)	5.0	(2.6 - 8.6)	0.9	(0.2 - 3.1)	4.1	(2.0 - 7.5)	17.2
Zambia (2013/14)	10.7	(8.5 - 13.2)	1.7	(0.9 - 2.9)	9.0	(7.0 - 11.3)	15.9
Zambia (2018/19)	25.5	(20.8 - 30.4)	7.1	(4.6 - 10.3)	18.4	(14.3 - 22.9)	27.9
Zimbabwe (1994)	8.3	(5.5 - 11.8)	2.0	(0.9 - 4.1)	6.2	(3.9 - 9.4)	24.5
Zimbabwe (1999)	3.6	(1.4 - 7.6)	1.1	(0.2 - 4.3)	2.5	(0.8 - 6.0)	30.9
Zimbabwe (2005/6)	7.3	(3.5 - 13.1)	3.4	(1.1 - 7.9)	3.9	(1.4 - 8.7)	46.3
Zimbabwe (2010/11)	14.2	(8.3 - 21.7)	3.0	(0.8 - 7.6)	11.2	(6.1 - 18.2)	21.1
Zimbabwe (2015)	9.9	(5.2 - 16.4)	3.1	(0.9 - 7.7)	6.7	(3.0 - 12.5)	31.8
**North Africa Western Asia and Europe**							
Albania (2017/18)	1.2	(0.7 - 1.8)	0.9	(0.5 - 1.5)	0.3	(0.1 - 0.7)	76.7
Armenia (2000)	3.5	(2.7 - 4.5)	1.1	(0.7 - 1.6)	2.5	(1.8 - 3.3)	30.0

Armenia (2005)	1.8	(1.2 - 2.8)	0.6	(0.2 - 1.2)	1.3	(0.7 - 2.1)	30.1
Armenia (2010)	1.6	(0.9 - 2.7)	0.1	(0.0 - 0.7)	1.5	(0.8 - 2.6)	8.2
Armenia (2015/16)	3.4	(2.3 - 4.7)	0.8	(0.4 - 1.6)	2.5	(1.6 - 3.8)	24.7
Azerbaijan (2006)	1.0	(0.6 - 1.6)	0.2	(0.0 - 0.5)	0.9	(0.5 - 1.4)	14.9
Egypt (1995/96)	11.0	(5.8 - 18.1)	0.5	(0.0 - 4.4)	10.5	(5.5 - 17.5)	4.2
Jordan (1990)	11.0	(8.6 - 13.7)	2.5	(1.5 - 4.1)	8.4	(6.3 - 10.9)	23.1
Jordan (1997)	12.7	(10.8 - 14.9)	2.1	(1.3 - 3.1)	10.7	(8.9 - 12.7)	16.2
Jordan (2002)	12.7	(10.9 - 14.8)	2.2	(1.5 - 3.2)	10.5	(8.8 - 12.4)	17.4
Jordan (2007)	8.5	(7.3 - 9.8)	1.2	(0.8 - 1.7)	7.3	(6.2 - 8.6)	13.6
Jordan (2009)	11.5	(10.2 - 12.9)	1.1	(0.8 - 1.6)	10.3	(9.1 - 11.7)	9.9
Jordan (2012)	11.7	(10.5 - 12.9)	0.9	(0.6 - 1.4)	10.8	(9.6 - 12.0)	8.1
Jordan (2017/18)	7.8	(6.7 - 9.0)	2.7	(2.1 - 3.5)	5.0	(4.2 - 6.0)	35.2
Jordan (2023)	5.4	(4.6 - 6.3)	2.6	(2.0 - 3.2)	2.9	(2.3 - 3.6)	47.2
Moldova (2005)	10.5	(8.8 - 12.3)	1.1	(0.6 - 1.8)	9.4	(7.8 - 11.2)	10.3
Morocco (1992)	7.4	(4.5 - 11.2)	1.6	(0.5 - 3.8)	5.8	(3.3 - 9.3)	21.5
Morocco (2003/4)	19.8	(16.6 - 23.1)	3.3	(2.1 - 5.0)	16.4	(13.5 - 19.6)	16.9
Türkiye (1993)	10.3	(9.0 - 11.6)	1.5	(1.0 - 2.1)	8.8	(7.6 - 10.1)	14.2
Türkiye (1998)	8.7	(7.5 - 10.0)	1.2	(0.8 - 1.7)	7.5	(6.4 - 8.8)	13.5
Türkiye (2003/4)	12.9	(11.6 - 14.2)	1.1	(0.7 - 1.5)	11.8	(10.6 - 13.0)	8.4
Türkiye (2018/19)	5.3	(4.0 - 6.8)	1.1	(0.6 - 1.8)	4.2	(3.1 - 5.6)	19.8
Ukraine (2007)	11.6	(9.3 - 14.2)	0.3	(0.1 - 1.0)	11.3	(9.0 - 13.9)	2.5
Yemen (2013)	8.9	(6.7 - 11.3)	0.7	(0.2 - 1.6)	8.2	(6.2 - 10.6)	7.4
**Central, South & Southeast Asia**							
Bangladesh (1993/94)	12.1	(9.0 - 15.8)	4.2	(2.4 - 6.6)	8.0	(5.4 - 11.1)	34.3
Bangladesh (1996/97)	26.8	(21.9 - 31.9)	1.7	(0.6 - 3.7)	25.1	(20.3 - 30.1)	6.3
Bangladesh (1999/0)	14.2	(11.4 - 17.2)	3.3	(2.1 - 5.1)	10.8	(8.4 - 13.6)	23.6
Bangladesh (2004)	23.1	(19.8 - 26.4)	1.5	(0.7 - 2.7)	21.6	(18.4 - 24.9)	6.5
Bangladesh (2011)	11.6	(8.2 - 15.5)	2.0	(0.8 - 4.2)	9.5	(6.5 - 13.2)	17.7
Bangladesh (2014)	12.3	(8.5 - 16.9)	0.5	(0.1 - 2.4)	11.8	(8.1 - 16.3)	4.0
Bangladesh (2017/18)	10.2	(7.8 - 13.1)	2.2	(1.1 - 3.7)	8.1	(5.9 - 10.7)	21.1
Bangladesh (2022)	10.4	(8.1 - 13.1)	2.6	(1.5 - 4.1)	7.9	(5.8 - 10.3)	24.6
Cambodia (2010/11)	4.5	(3.4 - 5.7)	0.7	(0.4 - 1.3)	3.8	(2.8 - 4.9)	16.4
Cambodia (2014)	5.0	(4.1 - 6.0)	0.9	(0.6 - 1.5)	4.1	(3.2 - 5.0)	18.8
Cambodia (2021/22)	7.6	(6.5 - 8.8)	2.5	(1.9 - 3.3)	5.1	(4.2 - 6.1)	33.3
India (2005/6)	7.9	(7.0 - 9.0)	1.7	(1.3 - 2.3)	6.2	(5.4 - 7.2)	21.6
India (2015/16)	13.9	(13.3 - 14.4)	5.4	(5.0 - 5.7)	8.5	(8.1 - 8.9)	38.6
India (2019/21)	13.4	(13.0 - 13.8)	6.0	(5.7 - 6.3)	7.4	(7.1 - 7.7)	44.8
Indonesia (1991)	12.9	(9.0 - 17.5)	0.6	(0.1 - 2.4)	12.3	(8.5 - 16.8)	4.5

Indonesia (1994)	11.4	(8.0 - 15.6)	2.9	(1.3 - 5.4)	8.6	(5.6 - 12.3)	25.3
Indonesia (1997)	13.3	(9.3 - 18.0)	2.1	(0.8 - 4.4)	11.2	(7.6 - 15.7)	15.5
Indonesia (2002/3)	4.2	(2.4 - 6.8)	0.7	(0.2 - 2.1)	3.6	(1.9 - 6.0)	15.9
Indonesia (2007)	8.6	(6.5 - 10.9)	3.0	(1.8 - 4.6)	5.6	(4.0 - 7.5)	34.9
Indonesia (2012)	8.5	(6.6 - 10.6)	2.8	(1.8 - 4.2)	5.7	(4.1 - 7.5)	33.0
Indonesia (2017)	8.5	(7.2 - 10.0)	1.2	(0.8 - 1.9)	7.3	(6.1 - 8.7)	14.3
Kazakhstan (1999)	24.7	(19.8 - 30.0)	4.3	(2.3 - 7.2)	20.5	(15.9 - 25.4)	17.2
Kyrgyz Republic (2012)	9.9	(5.9 - 15.0)	1.0	(0.2 - 3.6)	8.9	(5.2 - 13.8)	9.8
Maldives (2009)	3.6	(1.9 - 6.2)	0.6	(0.1 - 2.1)	3.1	(1.5 - 5.4)	15.8
Nepal (2011)	4.0	(2.8 - 5.6)	1.1	(0.5 - 2.1)	2.9	(1.9 - 4.2)	27.6
Nepal (2016)	6.4	(5.2 - 7.7)	1.7	(1.2 - 2.5)	4.6	(3.6 - 5.8)	27.2
Nepal (2022)	5.2	(4.3 - 6.3)	1.0	(0.7 - 1.6)	4.2	(3.4 - 5.2)	19.8
Pakistan (2012/13)	4.3	(3.3 - 5.5)	1.8	(1.2 - 2.7)	2.5	(1.7 - 3.4)	42.4
Pakistan (2017/18)	2.9	(2.0 - 4.1)	0.9	(0.4 - 1.6)	2.1	(1.3 - 3.1)	29.8
Philippines (1993)	7.4	(6.0 - 9.1)	2.3	(1.5 - 3.2)	5.2	(4.0 - 6.6)	30.2
Philippines (1998)	11.0	(9.3 - 12.7)	3.3	(2.5 - 4.4)	7.6	(6.3 - 9.2)	30.3
Philippines (2003)	10.8	(9.1 - 12.7)	3.0	(2.1 - 4.1)	7.8	(6.4 - 9.5)	27.5
Philippines (2022)	11.6	(10.3 - 13.0)	5.6	(4.7 - 6.6)	6.0	(5.1 - 7.1)	48.1
Tajikistan (2012)	5.5	(2.6 - 10.1)	0.4	(0.0 - 2.9)	5.1	(2.3 - 9.6)	7.0
Tajikistan (2017)	7.2	(3.5 - 12.7)	1.3	(0.2 - 4.6)	6.0	(2.7 - 11.1)	17.3
Vietnam (1997)	8.5	(6.4 - 10.9)	1.5	(0.7 - 2.7)	7.0	(5.1 - 9.2)	17.6
Vietnam (2002)	10.7	(8.7 - 13.0)	0.9	(0.4 - 1.8)	9.8	(7.9 - 12.0)	8.4
**Latin America & Caribbean**							
Bolivia (1994)	15.6	(11.2 - 20.7)	1.4	(0.4 - 3.7)	14.2	(10.0 - 19.2)	9.0
Brazil (1996)	25.0	(21.8 - 28.4)	3.8	(2.6 - 5.5)	21.2	(18.1 - 24.4)	15.3
Colombia (1990)	10.3	(7.4 - 13.7)	1.9	(0.9 - 3.8)	8.3	(5.8 - 11.5)	19.0
Colombia (1995)	24.9	(22.3 - 27.6)	4.4	(3.3 - 5.8)	20.5	(18.0 - 23.0)	17.8
Colombia (2000)	24.7	(22.5 - 27.0)	2.6	(1.8 - 3.5)	22.1	(20.0 - 24.4)	10.4
Colombia (2005)	20.1	(18.7 - 21.6)	3.1	(2.5 - 3.7)	17.1	(15.8 - 18.5)	15.2
Colombia (2010)	20.5	(19.0 - 22.1)	5.0	(4.2 - 5.9)	15.5	(14.2 - 16.9)	24.5
Colombia (2015/16)	16.4	(14.3 - 18.6)	1.6	(1.0 - 2.5)	14.8	(12.7 - 16.9)	9.8
Dominican Republic (1991)	19.5	(15.6 - 23.7)	4.9	(3.0 - 7.5)	14.6	(11.2 - 18.3)	25.2
Dominican Republic (1996)	18.5	(15.2 - 22.1)	3.6	(2.2 - 5.5)	14.9	(11.9 - 18.3)	19.3
Dominican Republic (2002)	26.3	(23.5 - 29.1)	4.4	(3.2 - 5.8)	21.9	(19.3 - 24.6)	16.7
Guatemala (2014/15)	4.9	(3.7 - 6.2)	0.8	(0.4 - 1.5)	4.0	(3.0 - 5.3)	16.9
Honduras (2011/12)	23.5	(21.8 - 25.2)	7.0	(6.1 - 8.1)	16.5	(15.0 - 18.0)	29.9
Nicaragua (1998)	13.3	(8.8 - 18.8)	1.8	(0.5 - 4.7)	11.5	(7.3 - 16.7)	13.8
Paraguay (1990)	16.9	(11.8 - 22.9)	4.7	(2.2 - 8.6)	12.3	(7.9 - 17.7)	27.6

Peru (1991/92)	16.8	(14.2 - 19.6)	1.5	(0.8 - 2.5)	15.3	(12.9 - 18.0)	8.8
Peru (1996)	19.8	(17.6 - 22.0)	2.2	(1.5 - 3.1)	17.5	(15.5 - 19.7)	11.2
Peru (2000)	15.9	(13.6 - 18.3)	2.8	(1.9 - 4.0)	13.1	(11.0 - 15.4)	17.6
Peru (2004/6)	21.2	(18.6 - 23.9)	3.6	(2.5 - 5.0)	17.5	(15.1 - 20.0)	17.2
Peru (2007/8)	22.3	(20.3 - 24.4)	2.4	(1.7 - 3.2)	20.0	(18.1 - 21.9)	10.6
Peru (2009)	12.8	(11.8 - 13.9)	2.0	(1.6 - 2.4)	10.8	(9.9 - 11.8)	15.4
Peru (2010)	20.1	(18.4 - 21.7)	3.4	(2.7 - 4.2)	16.7	(15.1 - 18.2)	16.9
Peru (2011)	18.0	(16.4 - 19.7)	2.2	(1.6 - 2.9)	15.8	(14.3 - 17.4)	12.1
Peru (2012)	22.4	(20.7 - 24.1)	2.8	(2.2 - 3.5)	19.6	(18.0 - 21.3)	12.5

CI= Confidence Interval
