# Supplementary material for: Contraceptive discontinuation, switching, abandonment and their reproductive consequences: An analysis of 1,539,071 episodes of reversible method use contributed from 61 countries that participated in DHS: Population base-analysis
Source: PLOS Glob Public Health. 2025 Oct 31;5(10):e0005174. doi: 10.1371/journal.pgph.0005174 (PMC12578211; doi:10.1371/journal.pgph.0005174)
Supplement: S15 Table — (PDF) [file pgph.0005174.s026.pdf]

S15 Table: Trends in 12-month method abandonment

	Declining trends		Increasing trends		No of countries
	Significant	Not significant	Not significant	Significant	
Contraceptive method					
Oral contraceptive	8	5	4	1	18
IUD	5	1	6	3	15
Injectables	6	4	2	4	16
Condom	7	6	2	3	18
Implants		1	4	2	7
Periodic abstinence	1	7	4	4	16
Withdrawal		3	8	4	15
**Total**	27	27	30	21	105
