# Supplementary material for: Contraceptive discontinuation, switching, abandonment and their reproductive consequences: An analysis of 1,539,071 episodes of reversible method use contributed from 61 countries that participated in DHS: Population base-analysis
Source: PLOS Glob Public Health. 2025 Oct 31;5(10):e0005174. doi: 10.1371/journal.pgph.0005174 (PMC12578211; doi:10.1371/journal.pgph.0005174)
Supplement: S16 Table — (PDF) [file pgph.0005174.s027.pdf]

S16 Table: Cumulative incidence of wanted and unwanted conceptions at 12 months following discontinuation

	Method-related		Wanted pregnancy		No Further need		Other reasons/Don't Know	
Reason for discontinuation	Wanted	Unwanted	Wanted	Unwanted	Wanted	Unwanted	Wanted	Unwanted
**Sub-Saharan Africa**								
Angola (2015/16)	23.6	3.8	49.0	1.6	10.7	1.5	16.4	2.8
Benin (2017/18)	16.3	4.1	53.7	7.4	18.5	5.7	23.7	7.1
Burkina Faso (2010)	16.7	2.3	62.5	6.4	4.3	0.0	33.6	2.7
Burkina Faso (2021)	18.8	1.9	53.8	2.4	12.4	1.9	24.9	0.3
Burundi (2010/11)	19.9	10.0	52.3	17.5	7.9	4.9	35.9	11.6
Comoros (2012)	34.7	1.6	65.2	0.0	57.5	0.0	30.0	4.5
Côte d'Ivoire (2021)	14.9	1.4	51.2	4.1	11.0	0.4	27.2	0.5
Ethiopia (2005)	15.2	4.7	59.7	17.4	2.6	2.5	14.2	10.3
Ethiopia (2016)	17.4	6.2	60.9	8.2	4.9	0.3	21.0	8.1
Gabon (2019/21)	14.6	5.3	48.8	9.6	10.9	1.7	16.0	5.7
Gambia (2013)	30.1	6.0	62.2	11.1	12.2	0.0	38.3	1.3
Gambia (2019/20)	20.4	3.0	55.4	3.4	7.1	0.9	26.0	2.9
Ghana (2014)	18.0	5.3	65.0	7.7	6.3	0.0	32.5	2.4
Ghana (2022/23)	19.7	2.8	57.7	6.1	8.8	1.7	18.9	1.1
Guinea (2018)	11.5	0.9	54.4	0.0	7.9	1.1	10.1	0.6
Kenya (1998)	15.5	9.1	72.6	10.9	13.2	1.2	36.3	10.7
Kenya (2003)	18.3	7.0	59.9	13.6	5.9	0.8	25.6	11.5
Kenya (2014)	10.1	3.9	64.3	11.6	4.5	1.3	22.6	7.2
Kenya (2022)	11.2	3.4	66.9	8.2	10.0	1.7	39.2	6.9
Lesotho (2014)	9.0	4.6	50.3	14.6	6.7	1.9	17.6	6.8
Liberia (2013)	24.3	4.1	51.4	4.3	0.0	0.0	31.7	1.5
Liberia (2019/20)	20.5	2.0	34.4	4.4	2.5	0.7	23.2	2.7
Madagascar (2021)	10.2	1.5	66.9	4.7	6.1	0.0	16.9	1.4
Malawi (2004/5)	25.6	6.5	64.5	8.0	8.3	2.7	39.6	5.8
Malawi (2015/16)	19.2	6.1	49.5	9.9	7.8	2.7	21.0	6.3
Mali (2012/13)	15.2	7.0	46.3	4.0	16.4	3.0	31.3	1.1
Mali (2018)	23.8	4.4	56.2	3.8	16.9	3.5	37.7	5.0
Mozambique (2011)	16.5	3.7	47.5	6.0	12.7	0.4	25.7	3.7
Mozambique (2022/23)	11.6	3.4	45.6	3.8	9.7	2.0	15.5	3.4
Namibia (2013)	13.3	4.7	51.8	10.1	7.4	1.2	26.6	6.6
Niger (2012)	17.8	1.2	67.0	2.1	26.0	0.9	50.2	1.2
Nigeria (2013)	9.9	1.6	78.5	5.4	12.1	0.1	32.2	2.3
Nigeria (2018)	18.6	2.2	66.2	5.9	14.1	1.5	21.8	3.9
Rwanda (2010/11)	11.0	6.2	68.2	13.7	6.8	3.4	24.4	14.8
Rwanda (2014/15)	14.9	8.0	68.0	10.4	10.2	2.5	24.2	13.3
Rwanda (2019/20)	14.3	7.2	64.5	12.9	7.9	5.6	21.4	13.0

Senegal (2010/11)	16.2	3.6	54.1	3.7	2.6	0.0	24.5	4.8
Senegal (2015)	21.5	4.4	57.2	3.7	4.3	0.0	32.3	5.6
Senegal (2016)	17.9	1.2	56.6	3.1	7.1	0.7	40.6	3.1
Senegal (2018)	19.1	3.0	55.0	1.8	8.9	0.8	32.8	3.7
Senegal (2019)	21.3	3.4	52.4	2.4	7.3	0.0	44.2	4.5
Senegal (2023)	19.2	2.0	44.7	3.6	8.3	0.0	22.7	0.3
Sierra Leone (2013)	18.3	2.5	62.4	4.5	14.3	0.0	34.3	3.2
South Africa (2016)	9.4	5.1	46.2	6.1	11.2	1.1	18.0	3.0
Tanzania (2004/5)	15.3	3.1	73.2	4.8	7.3	0.7	27.6	3.4
Tanzania (2015/16)	13.4	2.6	67.7	6.8	11.7	0.9	25.6	4.5
Tanzania (2022)	21.3	2.5	56.3	2.5	14.3	0.6	21.4	0.9
Uganda (2011)	25.9	8.8	67.1	8.1	6.7	1.3	38.4	7.5
Zambia (2013/14)	16.9	4.3	74.8	8.3	14.1	0.4	27.4	6.1
Zambia (2018/19)	20.5	3.6	64.5	7.3	10.4	3.7	29.2	3.3
Zimbabwe (1994)	23.5	7.0	71.1	8.8	11.6	2.8	39.6	8.4
Zimbabwe (1999)	14.7	5.0	70.4	4.7	11.0	2.3	30.8	3.7
Zimbabwe (2005/6)	14.8	4.3	73.4	8.4	5.5	0.7	30.7	5.5
Zimbabwe (2010/11)	17.7	3.8	63.8	8.7	9.2	1.3	22.4	2.3
Zimbabwe (2015)	14.7	3.0	69.7	6.7	9.8	1.5	29.3	2.5
**North Africa Western Asia and Europe**								
Albania (2017/18)	7.0	0.0	62.9	7.0	1.2	1.6	3.1	0.6
Armenia (2000)	8.6	8.9	74.4	13.1	0.5	1.0	19.8	12.1
Armenia (2005)	5.8	10.3	80.8	4.9	0.6	2.5	15.3	16.5
Armenia (2010)	2.4	0.0	79.8	9.1	0.0	0.0	21.6	5.5
Armenia (2015/16)	4.2	0.2	75.4	4.6	1.2	1.1	20.4	18.1
Azerbaijan (2006)	3.5	7.1	68.7	11.0	4.3	0.9	28.8	20.0
Egypt (1992/93)	13.7	14.3	64.3	19.7	4.7	2.5	19.5	10.9
Egypt (1995/96)	14.6	13.5	65.4	16.2	3.0	2.5	18.3	13.7
Egypt (2000)	14.2	5.4	70.7	9.1	2.6	1.6	24.9	8.7
Egypt (2003)	13.3	5.4	68.7	10.8	2.8	1.3	23.4	16.7
Egypt (2005)	11.8	6.6	67.3	12.1	3.3	0.7	10.2	8.0
Egypt (2008)	14.4	5.8	66.4	11.7	3.0	0.8	29.2	8.6
Egypt (2014)	11.5	5.4	62.4	11.3	4.4	1.4	27.6	24.0
Jordan (1990)	19.1	11.5	66.4	18.4	12.7	4.1	23.4	12.8
Jordan (1997)	11.8	7.7	68.1	16.5	6.2	0.0	21.4	11.3
Jordan (2002)	11.6	7.9	64.6	19.4	3.7	1.0	12.3	9.4
Jordan (2007)	11.2	4.6	68.0	14.6	2.9	3.3	17.3	6.8
Jordan (2009)	7.6	4.8	68.7	15.8	2.6	0.2	21.6	10.1
Jordan (2012)	4.9	2.5	65.1	19.2	3.0	1.3	9.4	4.9
Jordan (2017/18)	12.6	3.3	53.6	15.8	9.1	3.4	20.0	7.9
Jordan (2023)	11.7	8.7	52.3	22.0	7.1	9.0	17.6	10.6

Morocco (1992)	14.5	14.5	66.8	13.5	2.3	0.8	20.6	10.5
Morocco (2003/4)	4.1	1.9	67.4	9.1	1.9	1.5	17.0	6.8
Türkiye (1993)	3.1	5.4	69.5	11.9	2.7	3.1	9.0	10.1
Türkiye (1998)	4.8	4.6	70.7	8.9	2.5	0.0	21.6	11.4
Türkiye (2018/19)	5.8	6.0	74.0	4.0	2.8	1.1	20.0	4.7
Ukraine (2007)	0.5	0.0	66.4	2.9	0.3	0.0	9.8	4.3
Yemen (2013)	13.8	7.1	58.7	17.0	8.6	2.1	25.6	7.9
**Central, South & Southeast Asia**								
Kazakhstan (1999)	9.5	1.9	66.9	8.7	2.4	0.0	23.0	4.4
Kyrgyz Republic (2012)	26.2	3.4	70.1	6.2	1.0	1.0	30.2	1.4
Tajikistan (2012)	11.3	11.5	66.9	18.7	3.0	0.0	24.1	8.8
Tajikistan (2017)	16.1	6.4	58.2	5.2	11.0	5.2	12.9	7.9
Bangladesh (1993/94)	7.9	7.8	57.6	14.9	2.1	1.7	9.8	6.6
Bangladesh (1996/97)	7.1	9.4	56.7	13.9	1.6	1.8	11.5	9.3
Bangladesh (1999/0)	7.8	6.8	61.8	12.9	3.1	0.9	12.7	5.3
Bangladesh (2004)	5.8	4.6	56.7	11.2	2.8	0.0	6.9	4.3
Bangladesh (2011)	3.4	3.7	58.6	10.8	1.0	0.3	8.3	3.3
Bangladesh (2014)	3.5	2.7	63.0	8.9	1.4	0.7	13.7	5.2
Bangladesh (2017/18)	1.6	1.6	58.7	9.4	0.8	0.1	10.8	5.6
Bangladesh (2022)	3.1	1.6	59.8	8.0	2.9	0.6	5.2	1.3
Cambodia (2010/11)	14.5	5.4	68.5	6.2	7.1	0.4	30.3	9.3
Cambodia (2014)	13.0	3.5	67.2	3.4	6.5	2.4	20.1	5.8
Cambodia (2021/22)	10.2	4.6	63.2	7.1	5.5	2.7	12.2	7.1
India (2005/6)	10.0	8.0	65.2	18.0	3.0	3.8	18.0	12.7
India (2015/16)	6.5	3.2	58.2	13.2	6.6	2.6	9.0	4.0
India (2019/21)	6.4	2.8	47.3	10.9	7.5	3.1	8.4	3.1
Indonesia (1991)	8.7	4.9	60.0	8.4	0.8	1.1	20.5	6.8
Indonesia (1994)	8.7	3.4	61.2	5.4	1.8	0.5	11.0	5.0
Indonesia (1997)	5.9	2.5	57.7	5.8	3.3	2.1	13.0	5.7
Indonesia (2002/3)	4.9	1.8	58.8	5.8	2.0	1.2	18.6	5.8
Indonesia (2007)	7.7	2.1	56.9	8.3	2.0	0.6	12.5	3.2
Indonesia (2012)	3.7	1.3	53.6	7.5	1.9	0.3	14.0	5.0
Indonesia (2017)	3.7	1.3	53.1	5.9	2.0	0.5	15.6	5.8
Maldives (2009)	12.2	2.2	60.7	3.9	9.1	0.0	33.8	1.3
Myanmar (2015/16)	5.8	1.6	56.0	5.0	2.8	0.2	6.1	1.2
Nepal (2011)	6.7	9.4	59.1	13.2	2.1	1.4	6.6	10.9
Nepal (2016)	5.2	5.8	60.5	17.0	2.2	1.7	20.8	15.6
Nepal (2022)	5.6	4.6	61.4	14.5	2.4	1.1	12.1	13.6
Pakistan (2012/13)	14.2	9.1	64.4	17.9	10.0	3.5	23.7	11.0
Pakistan (2017/18)	17.4	7.9	63.3	17.7	3.6	0.0	29.6	26.5
Philippines (1993)	17.8	11.3	62.4	19.1	5.3	0.7	28.8	14.0

Philippines (1998)	11.0	5.4	66.5	15.1	5.5	2.6	20.1	8.4
Philippines (2003)	13.1	7.9	59.7	17.7	3.7	1.9	20.0	12.6
Philippines (2022)	7.2	5.2	50.5	12.9	5.4	3.4	10.8	5.5
Vietnam (1997)	7.5	11.9	69.2	18.1	0.0	9.7	7.4	21.1
Vietnam (2002)	4.7	5.0	77.1	10.4	2.4	0.0	6.2	16.6
**Latin America & Caribbean**								
Bolivia (1994)	8.0	5.3	68.7	19.4	3.5	3.0	17.6	15.6
Brazil (1996)	10.4	5.7	68.9	7.9	4.8	2.4	9.2	3.1
Colombia (1990)	11.3	5.3	72.9	7.9	8.2	2.2	9.2	3.1
Colombia (1995)	7.8	3.7	71.3	8.2	4.1	1.8	12.3	5.3
Colombia (2000)	6.2	3.5	70.5	13.5	2.1	0.9	8.1	4.7
Colombia (2005)	9.9	5.1	66.9	8.5	4.0	0.8	14.3	5.9
Colombia (2010)	9.7	3.6	64.2	8.5	3.4	1.2	13.5	4.8
Colombia (2015/16)	7.9	2.8	58.3	7.4	3.2	1.5	11.6	3.7
Dominican Republic (1991)	24.1	4.7	75.2	6.9	10.2	3.2	24.1	5.0
Dominican Republic (1996)	23.9	7.3	75.0	7.0	8.5	3.6	23.1	7.0
Dominican Republic (2002)	22.1	6.8	74.3	9.5	8.8	2.2	25.1	5.3
Guatemala (1995)	21.6	6.3	73.9	7.8	12.8	1.6	25.8	7.2
Guatemala (1998/99)	14.1	4.1	68.1	12.5	11.9	0.0	20.5	1.8
Guatemala (2014/15)	18.3	3.7	73.4	8.1	2.0	0.2	30.5	5.0
Honduras (2011/12)	13.9	5.4	71.0	8.0	4.7	0.8	16.8	4.2
Nicaragua (1998)	12.9	5.9	67.9	11.6	4.4	2.2	21.9	7.0
Paraguay (1990)	8.2	1.4	75.3	4.4	3.4	0.7	7.8	1.3
Peru (1991/92)	5.7	5.7	73.0	17.1	1.1	2.2	12.2	9.8
Peru (1996)	4.7	3.7	72.1	15.7	3.7	1.5	10.5	5.8
Peru (2000)	5.4	3.9	69.1	10.5	3.7	1.5	10.8	4.6
Peru (2004/6)	4.8	3.9	70.5	9.7	4.4	1.2	3.9	3.3
Peru (2007/8)	5.2	3.1	70.2	9.7	2.2	1.0	4.3	1.9
Peru (2009)	4.6	3.1	64.9	11.4	3.8	0.9	4.5	1.3
Peru (2010)	5.1	3.2	68.2	10.7	2.7	1.6	3.2	1.4
Peru (2011)	5.3	3.1	68.7	9.0	2.3	1.4	3.5	1.5
Peru (2012)	5.0	3.2	67.3	10.6	3.1	0.7	5.2	1.2
