# Supplementary material for: Contraceptive discontinuation, switching, abandonment and their reproductive consequences: An analysis of 1,539,071 episodes of reversible method use contributed from 61 countries that participated in DHS: Population base-analysis
Source: PLOS Glob Public Health. 2025 Oct 31;5(10):e0005174. doi: 10.1371/journal.pgph.0005174 (PMC12578211; doi:10.1371/journal.pgph.0005174)
Supplement: S17 Table — (PDF) [file pgph.0005174.s028.pdf]

S17 Table: Conception rates at 12 and 24 month, by switchers and abandoners,
After method-related discontinuation within 12 months of use

Region<br>Country	Survey<br>round	at 12 months<br>Switchers	 abandoners	at 24 months<br>Switchers	 abandoners
**Sub-Saharan Africa**					
Angola	2015/16	2.0	40.5	2.0	52.7
Benin	2017/18	12.3	35.3	18.8	54.9
Burkina Faso	2021	6.5	35.6	22.0	51.4
Burundi	2010/11	9.0	66.1	30.9	85.6
Côte d'Ivoire	2021	1.7	21.8	20.3	33.8
Ethiopia	2016	6.6	49.1	16.2	67.5
Gabon	2019/21	8.2	34.9	16.8	56.0
Gambia	2019/20	2.3	37.0	16.2	54.8
Ghana	2022/23	8.3	41.7	24.8	59.2
Guinea	2018	13.9	21.5	29.6	31.2
Kenya	2022	6.1	36.6	13.9	49.5
Lesotho	2014	3.2	45.7	8.1	59.0
Liberia	2019/20	8.1	23.9	15.1	37.9
Madagascar	2021	3.2	28.1	15.9	43.4
Malawi	2015/16	7.3	40.6	18.1	62.2
Mali	2018	14.1	38.0	40.4	63.3
Mozambique	2022/23	4.4	22.5	13.6	35.2
Namibia	2013	5.1	34.7	22.1	50.9
Nigeria	2018	7.2	35.0	21.4	55.8
Rwanda	2019/20	7.8	60.1	20.8	72.8
Senegal	2023	26.4	30.7	36.1	48.2
Sierra Leone	2013	8.1	33.2	10.1	44.9
South Africa	2016	1.4	20.0	3.7	31.6
Tanzania	2022	8.1	36.8	17.0	52.2
Uganda	2011	11.5	60.2	44.5	80.8
Zambia	2018/19	10.3	46.2	29.8	62.6
Zimbabwe	2015	2.3	50.1	10.5	59.7
**North Africa Western Asia and Europe**					
Egypt	2014	8.7	56.7	24.9	66.5
Jordan	2023	7.4	50.5	13.4	58.0
Moldova	2005	9.5	51.8	21.5	51.8
Morocco	2003/4	9.8	52.2	24.2	60.7
Türkiye	2018/19	5.4	46.6	10.7	59.1
Ukraine	2007	5.3	0.0	10.0	0.0
Yemen	2013	10.4	62.2	27.9	75.0

**Central, South & Southeast Asia**					
Kyrgyz Republic	2012	10.3	62.0	24.3	67.6
Tajikistan	2017	0.0	25.0	23.8	44.1
Bangladesh	2022	4.9	24.8	11.3	35.4
Cambodia	2021/22	4.6	35.0	14.0	46.1
India	2019/21	4.9	19.0	12.5	28.3
Indonesia	2017	3.9	29.3	12.2	43.6
Maldives	2009	6.6	30.3	16.2	46.8
Myanmar	2015/16	8.6	20.7	16.9	34.8
Nepal	2022	1.9	30.2	7.2	47.1
Pakistan	2017/18	6.9	44.9	29.5	62.2
Philippines	2022	6.6	31.1	12.7	42.4
Vietnam	2002	9.0	85.1	25.4	88.6
**Latin America & Caribbean**					
Colombia	2015/16	5.3	43.5	13.3	51.3
Dominican Republic	2002	14.4	66.0	32.5	77.3
Guatemala	2014/15	8.1	62.4	20.2	74.1
Honduras	2011/12	10.6	69.7	26.3	78.4
Peru	2012	8.9	62.5	20.8	70.0
