# Supplementary material for: Contraceptive discontinuation, switching, abandonment and their reproductive consequences: An analysis of 1,539,071 episodes of reversible method use contributed from 61 countries that participated in DHS: Population base-analysis
Source: PLOS Glob Public Health. 2025 Oct 31;5(10):e0005174. doi: 10.1371/journal.pgph.0005174 (PMC12578211; doi:10.1371/journal.pgph.0005174)
Supplement: S18 Table — (PDF) [file pgph.0005174.s029.pdf]

S18 Table: Ranking countries by 12-month conception rates and 12-month discontinuation for method-related reasons

Country	ISO	Block	Rank
Ukraine	UA	1	1
Bangladesh	BD	1	2
Nepal	NP	1	3
Moldova	MD	1	4
South Africa	ZA	1	5
Mozambique	MZ	1	6
Côte d'Ivoire	CI	1	7
Vietnam	VN	1	8
Tajikistan	TJ	1	9
Maldives	MV	1	10
Cambodia	KH	1	11
Türkiye	TR	1	12
Sierra Leone	SL	1	13
Namibia	NA	2	14
Indonesia	ID	2	15
India	IN	2	16
Myanmar	MM	2	17
Madagascar	MG	2	18
Colombia	CO	2	19
Guinea	GN	2	20
Morocco	MA	2	21
Philippines	PH	2	22
Peru	PE	2	23
Liberia	LR	2	24
Kenya	KE	2	25
Lesotho	LS	3	26
Gabon	GA	3	27
Burkina Faso	BF	3	28
Zimbabwe	ZW	3	29
Egypt	EG	3	30
Nigeria	NG	3	31
Benin	BJ	3	32
Pakistan	PK	3	33
Jordan	JO	3	34
Guatemala	GT	3	35
Mali	ML	3	36
Kyrgyz Republic	KG	3	37

Senegal	SN	4	38
Tanzania	TZ	4	39
Gambia	GM	4	40
Honduras	HN	4	41
Ethiopia	ET	4	42
Ghana	GH	4	43
Malawi	MW	4	44
Zambia	ZM	4	45
Rwanda	RW	4	46
Angola	AO	4	47
Yemen	YE	4	48
Dominican Republic	DO	4	49
Uganda	UG	4	50
Burundi	BI	4	51

1= Conception rates < median and method-related discontinuation < median
2= Conception rates < median and method-related discontinuation >= median
3= Conception rates >= median and method-related discontinuation < median
4= Conception rates >= median and method-related discontinuation >= median
